# Supplementary material for: Evaluating the appropriateness of laboratory testing and antimicrobial use in South African children hospitalized for community-acquired infections
Source: PLoS One. 2022 Jul 28;17(7):e0272119. doi: 10.1371/journal.pone.0272119 (PMC9333228; doi:10.1371/journal.pone.0272119)
Supplement: S1 Appendix — (PDF) [file pone.0272119.s001.pdf]

Integrated Management of Childhood Illness

Chart Booklet

World Health Organization

March 2014

WHO Library Cataloguing-in-Publication Data:

Integrated Management of Childhood Illness: distance learning course.

15 booklets
Contents: - Introduction, self-study modules – Module 1: general danger signs for the sick child – Module 2: The sick young infant – Module 3: Cough or difficult breathing – Module 4: Diarrhoea – Module 5: Fever – Module 6: Malnutrition and anaemia – Module 7: Ear problems – Module 8: HIV/AIDS – Module 9: Care of the well child – Facilitator guide – Pediatric HIV: supplementary facilitator guide – Implementation: introduction and roll out – Logbook – Chart book

1.Child Health Services. 2.Child Care. 3.Child Mortality – prevention and control. 4.Delivery of Health Care, Integrated. 5.Disease Management. 6.Education, Distance. 7.Teaching Material. I.World Health Organization.

ISBN 978 92 4 150682 3 (NLM classification: WS 200)

© World Health Organization 2014

All rights reserved. Publications of the World Health Organization are available on the WHO website (www.who.int) or can be purchased from WHO Press, World Health Organization, 20 Avenue Appia, 1211 Geneva 27, Switzerland (tel.: +41 22 791 3264; fax: +41 22 791 4857; e-mail: bookorders@who.int).

Requests for permission to reproduce or translate WHO publications –whether for sale or for non-commercial distribution– should be addressed to WHO Press through the WHO website (www.who.int/about/licensing/copyright_form/en/index.html).

The designations employed and the presentation of the material in this publication do not imply the expression of any opinion whatsoever on the part of the World Health Organization concerning the legal status of any country, territory, city or area or of its authorities, or concerning the delimitation of its frontiers or boundaries. Dotted lines on maps represent approximate border lines for which there may not yet be full agreement.

The mention of specific companies or of certain manufacturers' products does not imply that they are endorsed or recommended by the World Health Organization in preference to others of a similar nature that are not mentioned. Errors and omissions excepted, the names of proprietary products are distinguished by initial capital letters.

All reasonable precautions have been taken by the World Health Organization to verify the information contained in this publication. However, the published material is being distributed without warranty of any kind, either expressed or implied. The responsibility for the interpretation and use of the material lies with the reader. In no event shall the World Health Organization be liable for damages arising from its use.

Printed in Switzerland

World Health Organization

Integrated Management of Childhood Illness

unicef

SICK CHILD AGE 2 MONTHS UP TO 5 YEARS

ASSESS AND CLASSIFY THE SICK CHILD

CHECK FOR GENERAL DANGER SIGNS 1
THEN ASK ABOUT MAIN SYMPTOMS: 2
Does the child have diarrhoea? 3
Does the child have fever? 4
Does the child have an ear problem? 5
THEN CHECK FOR ACUTE MALNUTRITION 6
THEN CHECK FOR ANAEMIA 7
THEN CHECK FOR HIV INFECTION 8
THEN CHECK THE CHILD'S IMMUNIZATION, VITAMIN A AND DEWORMING STATUS 9
ASSESS OTHER PROBLEMS: 9
HIV TESTING AND INTERPRENTING RESULTS 10
WHO PAEDIATRIC STAGING FOR HIV INFECTION 11

TREAT THE CHILD

TEACH THE MOTHER TO GIVE ORAL DRUGS AT HOME 12
Give an Appropriate Oral Antibiotic 12
Give Inhaled Salbutamol for Wheezing 13
Give Oral Antimalarial for MALARIA 13
Give Paracetamol for High Fever (> 38.5°C) or Ear Pain 13
Give Iron* 14
TEACH THE MOTHER TO TREAT LOCAL INFECTIONS AT HOME 15
Soothe the Throat, Relieve the Cough with a Safe Remedy 15
Treat Eye Infection with Tetracycline Eye Ointment 15
Clear the Ear by Dry Wicking and Give Eardrops* 15
Treat for Mouth Ulcers with Gentian Violet (GV) 15
Treat Thrush with Nystatin 15
GIVE VITAMIN A AND MEBENDAZOLE IN CLINIC 16
Give Vitamin A Supplementation and Treatment 16
Give Mebendazole 16
GIVE THESE TREATMENTS IN THE CLINIC ONLY 17
Give Intramuscular Antibiotics 17
Give Diazepam to Stop Convulsions 17
Give Artesunate Suppositories or Intramuscular Artesunate or Quinine for Severe Malaria 17
Treat the Child to Prevent Low Blood Sugar 18
GIVE EXTRA FLUID FOR DIARRHOEA AND CONTINUE FEEDING 19
PLAN A: TREAT DIARRHOEA AT HOME 19
PLAN B: TREAT SOME DEHYDRATION WITH ORS 19
PLAN C: TREAT SEVERE DEHYDRATION QUICKLY 20
GIVE READY-TO-USE THERAPEUTIC FOOD 21
Give Ready-to-Use Therapeutic Food for SEVERE ACUTE MALNUTRITION 21
TREAT THE HIV INFECTED CHILD 22
Steps when Initiating ART in Children 22
Preferred and Alternative ARV Regimens 23
Give Antiretroviral Drugs (Fixed Dose Combinations) 23
Give Antiretroviral Drugs 24
Side Effects ARV Drugs 25
Manage Side Effects of ARV Drugs 26
Give Pain Relief to HIV Infected Child 27
IMMUNIZE EVERY SICK CHILD AS NEEDED 27

FOLLOW-UP

GIVE FOLLOW-UP CARE FOR ACUTE CONDITIONS 28
PNEUMONIA 28
PERSISTENT DIARRHOEA 28
DYSENTERY 28
MALARIA 29
FEVER: NO MALARIA 29
MEASLES WITH EYE OR MOUTH COMPLICATIONS, GUM OR MOUTH ULCERS, OR THRUSH 29
EAR INFECTION 29
FEEDING PROBLEM 29
ANAEMIA 29
UNCOMPLICATED SEVERE ACUTE MALNUTRITION 30
MODERATE ACUTE MALNUTRITION 30
GIVE FOLLOW-UP CARE FOR HIV EXPOSED AND INFECTED CHILD 31
HIV EXPOSED 31
CONFIRMED HIV INFECTION NOT ON ART 31
CONFIRMED HIV INFECTION ON ART: THE FOUR STEPS OF FOLLOW-UP CARE 32

COUNSEL THE MOTHER

FEEDING COUNSELLING 33
Assess Child's Appetite 33
Assess Child's Feeding 34
Feeding Recommendations 35
Feeding Recommendations for HIV EXPOSED Child on Infant Formula 36
Stopping Breastfeeding 37
Feeding Recommendations For a Child Who Has PERSISTENT DIARRHOEA 37
EXTRA FLUIDS AND MOTHER'S HEALTH 38
Advise the Mother to Increase Fluid During Illness 38
Counsel the Mother about her Own Health 38
WHEN TO RETURN 39

Recording Form: Recording form 60

Recording Form: ART initiation steps 62

Recording Form: HIV on ART follow-up steps 64

SICK YOUNG INFANT AGE UP TO 2 MONTHS

ASSESS AND CLASSIFY THE SICK YOUNG INFANT

CHECK FOR VERY SEVERE DISEASE AND LOCAL BACTERIAL INFECTION 41
CHECK FOR JAUNDICE 42
THEN ASK: Does the young infant have diarrhoea*? 43
THEN CHECK FOR HIV INFECTION 43
THEN CHECK FOR FEEDING PROBLEM OR LOW WEIGHT FOR AGE 44
THEN CHECK FOR FEEDING PROBLEM OR LOW WEIGHT FOR AGE IN NON-BREASTFED INFANTS 45
THEN CHECK THE YOUNG INFANT'S IMMUNIZATION AND VITAMIN A STATUS: 46
ASSESS OTHER PROBLEMS 46
ASSESS THE MOTHER'S HEALTH NEEDS 46

TREAT AND COUNSEL

TREAT THE YOUNG INFANT 47
GIVE FIRST DOSE OF INTRAMUSCULAR ANTIBIOTICS 47
TREAT THE YOUNG INFANT TO PREVENT LOW BLOOD SUGAR 47
TEACH THE MOTHER HOW TO KEEP THE YOUNG INFANT WARM ON THE WAY TO THE HOSPITAL 48
GIVE AN APPROPRIATE ORAL ANTIBIOTIC FOR LOCAL BACTERIAL INFECTION 48
TEACH THE MOTHER TO TREAT LOCAL INFECTIONS AT HOME 48
To Treat Diarrhoea, See TREAT THE CHILD Chart. 48
Immunize Every Sick Young Infant, as Needed 49
GIVE ARV FOR PMTCT PROPHYLAXIS 49
COUNSEL THE MOTHER 50
TEACH CORRECT POSITIONING AND ATTACHMENT FOR BREASTFEEDING 50
TEACH THE MOTHER HOW TO EXPRESS BREAST MILK 50
TEACH THE MOTHER HOW TO FEED BY A CUP 50
TEACH THE MOTHER HOW TO KEEP THE LOW WEIGHT INFANT WARM AT HOME 50
ADVISE THE MOTHER TO GIVE HOME CARE FOR THE YOUNG INFANT 51

FOLLOW-UP

GIVE FOLLOW-UP CARE FOR THE YOUNG INFANT 52
ASSESS EVERY YOUNG INFANT FOR "VERY SEVERE DISEASE" DURING FOLLOW-UP VISIT 52
LOCAL BACTERIAL INFECTION 52
DIARRHOEA 52
JAUNDICE 53
FEEDING PROBLEM 53
LOW WEIGHT FOR AGE 53
THRUSH 54
CONFIRMED HIV INFECTION OR HIV EXPOSED 54

Recording Form: Young infant recording form 66

Annex:

Skin Problems

IDENTIFY SKIN PROBLEM 55
IF SKIN IS ITCHING 56
IF SKIN HAS BLISTERS/SORES/PUSTULES 57
NON-ITCHY 58
CLINICAL REACTION TO DRUGS 59
DRUG AND ALLERGIC REACTIONS 59

SICK CHILD AGE 2 MONTHS UP TO 5 YEARS

ASSESS AND CLASSIFY THE SICK CHILD

ASSESS	CLASSIFY	IDENTIFY TREATMENT

ASK THE MOTHER WHAT THE CHILD'S PROBLEMS ARE

- Determine if this is an initial or follow-up visit for this problem.
  - if follow-up visit, use the follow-up instructions on TREAT THE CHILD chart.
  - if initial visit, assess the child as follows:

USE ALL BOXES THAT MATCH THE CHILD'S SYMPTOMS AND PROBLEMS TO CLASSIFY THE ILLNESS

CHECK FOR GENERAL DANGER SIGNS

Ask:
- Is the child able to drink or breastfeed?
- Does the child vomit everything?
- Has the child had convulsions?

Look:
- See if the child is lethargic or unconscious.
- Is the child convulsing now?

URGENT attention

Signs	Classify	Identify treatment
• Any general danger sign	***Pink:*** **VERY SEVERE DISEASE**	■ **Give diazepam if convulsing now**<br>■ **Quickly complete the assessment**<br>■ **Give any pre-referal treatment immediately**<br>■ **Treat to prevent low blood sugar**<br>■ **Keep the child warm**<br>■ **Refer URGENTLY.**

A child with any general danger sign needs *URGENT* attention; complete the assessment and any pre-referral treatment immediately so referral is not delayed.

THEN ASK ABOUT MAIN SYMPTOMS:
Does the child have cough or difficult breathing?

Ask / Look, listen, feel	Classify	Signs	Classification	Treatment
If yes, ask:<br>• For how long?<br><br>***Look, listen, feel*:***<br>• Count the breaths in one minute.<br>• Look for chest indrawing.<br>• Look and listen for stridor.<br>• Look and listen for wheezing.<br>(CHILD MUST BE CALM)<br><br>**If wheezing with either fast breathing or chest indrawing:**<br>Give a trial of rapid acting inhaled bronchodilator for up to three times 15-20 minutes apart. Count the breaths and look for chest indrawing again, and then classify.<br><br>***If the child is:*** / ***Fast breathing is:***<br>2 months up to 12 months: **50** breaths per minute or more<br>12 Months up to 5 years: **40** breaths per minute or more	***Classify COUGH or DIFFICULT BREATHING***	• Any general danger sign or<br>• Stridor in calm child.	***Pink:***<br>**SEVERE PNEUMONIA OR VERY SEVERE DISEASE**	■ **Give first dose of an appropriate antibiotic**<br>■ **Refer URGENTLY to hospital****
		• Chest indrawing or<br>• Fast breathing.	***Yellow:***<br>**PNEUMONIA**	■ **Give oral Amoxicillin for 5 days*****<br>■ If wheezing (or disappeared after rapidly acting bronchodilator) give an inhaled bronchodilator for 5 days****<br>■ If chest indrawing in HIV exposed/infected child, give first dose of amoxicillin and refer.<br>■ Soothe the throat and relieve the cough with a safe remedy<br>■ If coughing for more than 14 days or recurrent wheeze, refer for possible TB or asthma assessment<br>■ Advise mother when to return immediately<br>■ Follow-up in 3 days
		• No signs of pneumonia or very severe disease.	***Green:***<br>**COUGH OR COLD**	■ If wheezing (or disappeared after rapidly acting bronchodilator) give an inhaled bronchodilator for 5 days****<br>■ Soothe the throat and relieve the cough with a safe remedy<br>■ If coughing for more than 14 days or recurrent wheezing, refer for possible TB or asthma assessment<br>■ Advise mother when to return immediately<br>■ Follow-up in 5 days if not improving

**If pulse oximeter is available, determine oxygen saturation and refer if < 90%.*

*** If referral is not possible, manage the child as described in the pneumonia section of the national referral guidelines or as in WHO Pocket Book for hospital care for children.*

****Oral Amoxicillin for 3 days could be used in patients with fast breathing but no chest indrawing in low HIV settings.*

***** In settings where inhaled bronchodilator is not available, oral salbutamol may be tried but not recommended for treatement of severe acute wheeze.*

Does the child have diarrhoea?

If yes, ask:

- For how long?
- Is there blood in the stool?

Look and feel:

- Look at the child's general condition. Is the child:
  - Lethargic or unconscious?
  - Restless and irritable?
- Look for sunken eyes.
- Offer the child fluid. Is the child:
  - Not able to drink or drinking poorly?
  - Drinking eagerly, thirsty?
- Pinch the skin of the abdomen. Does it go back:
  - Very slowly (longer than 2 seconds)?
  - Slowly?

Classify DIARRHOEA

	Signs	Classification	Treatment
**for DEHYDRATION**	Two of the following signs:<br>• Lethargic or unconscious<br>• Sunken eyes<br>• Not able to drink or drinking poorly<br>• Skin pinch goes back very slowly.	***Pink:***<br>**SEVERE DEHYDRATION**	■ If child has no other severe classification:<br>○ Give fluid for severe dehydration (Plan C)<br>OR<br>**If child also has another severe classification:**<br>○ **Refer URGENTLY to hospital with mother giving frequent sips of ORS on the way**<br>○ **Advise the mother to continue breastfeeding**<br>■ **If child is 2 years or older and there is cholera in your area, give antibiotic for cholera**
	Two of the following signs:<br>• Restless, irritable<br>• Sunken eyes<br>• Drinks eagerly, thirsty<br>• Skin pinch goes back slowly.	***Yellow:***<br>**SOME DEHYDRATION**	■ Give fluid, zinc supplements, and food for some dehydration (Plan B)<br>■ **If child also has a severe classification:**<br>○ **Refer URGENTLY to hospital with mother giving frequent sips of ORS on the way**<br>○ **Advise the mother to continue breastfeeding**<br>■ Advise mother when to return immediately<br>■ Follow-up in 5 days if not improving
	Not enough signs to classify as some or severe dehydration.	***Green:***<br>**NO DEHYDRATION**	■ Give fluid, zinc supplements, and food to treat diarrhoea at home (Plan A)<br>■ Advise mother when to return immediately<br>■ Follow-up in 5 days if not improving
**and if diarrhoea 14 days or more**	• Dehydration present.	***Pink:***<br>**SEVERE PERSISTENT DIARRHOEA**	■ Treat dehydration before referral unless the child has another severe classification<br>■ Refer to hospital
	• No dehydration.	***Yellow:***<br>**PERSISTENT DIARRHOEA**	■ Advise the mother on feeding a child who has PERSISTENT DIARRHOEA<br>■ Give multivitamins and minerals (including zinc) for 14 days<br>■ Follow-up in 5 days
**and if blood in stool**	• Blood in the stool.	***Yellow:***<br>**DYSENTERY**	■ **Give ciprofloxacin for 3 days**<br>■ Follow-up in 3 days

Does the child have fever?

(by history or feels hot or temperature 37.5°C* or above)

If yes:

Decide Malaria Risk: high or low

Then ask:

- For how long?
- If more than 7 days, has fever been present every day?
- Has the child had measles within the last 3 months?

Look and feel:

- Look or feel for stiff neck.
- Look for runny nose.
- Look for any bacterial cause of fever**.
- Look for signs of MEASLES.
  - Generalized rash and
  - One of these: cough, runny nose, or red eyes.

Do a malaria test: If NO severe classification***

- In all fever cases if High malaria risk.
- In Low malaria risk if no obvious cause of fever present.

Classify FEVER

High or Low Malaria Risk

Signs	Classify as	Treatment
• Any general danger sign or<br>• Stiff neck.	***Pink:*** **VERY SEVERE FEBRILE DISEASE**	■ **Give first dose of artesunate or quinine for severe malaria**<br>■ **Give first dose of an appropriate antibiotic**<br>■ **Treat the child to prevent low blood sugar**<br>■ **Give one dose of paracetamol in clinic for high fever (38.5°C or above)**<br>■ **Refer URGENTLY to hospital**
• Malaria test POSITIVE.	***Yellow:*** **MALARIA**	■ **Give recommended first line oral antimalarial**<br>■ **Give one dose of paracetamol in clinic for high fever (38.5°C or above)**<br>■ Give appropriate antibiotic treatment for an identified bacterial cause of fever<br>■ Advise mother when to return immediately<br>■ Follow-up in 3 days if fever persists<br>■ If fever is present every day for more than 7 days, refer for assessment
• Malaria test NEGATIVE<br>• Other cause of fever PRESENT.	***Green:*** **FEVER: NO MALARIA**	■ **Give one dose of paracetamol in clinic for high fever (38.5°C or above)**<br>■ **Give appropriate antibiotic treatment** for an identified bacterial cause of fever<br>■ Advise mother when to return immediately<br>■ Follow-up in 3 days if fever persists<br>■ If fever is present every day for more than 7 days, refer for assessment

No Malaria Risk and No Travel to Malaria Risk Area

Signs	Classify as	Treatment
• Any general danger sign<br>• Stiff neck.	***Pink:*** **VERY SEVERE FEBRILE DISEASE**	■ **Give first dose of an appropriate antibiotic.**<br>■ **Treat the child to prevent low blood sugar.**<br>■ **Give one dose of paracetamol in clinic for high fever (38.5°C or above).**<br>■ **Refer URGENTLY to hospital.**
• No general danger signs<br>• No stiff neck.	***Green:*** **FEVER**	■ **Give one dose of paracetamol in clinic for high fever (38.5°C or above)**<br>■ **Give appropriate antibiotic treatment** for any identified bacterial cause of fever<br>■ Advise mother when to return immediately<br>■ Follow-up in 2 days if fever persists<br>■ If fever is present every day for more than 7 days, refer for assessment

If the child has measles now or within the last 3 months:

- Look for mouth ulcers. Are they deep and extensive?
- Look for pus draining from the eye.
- Look for clouding of the cornea.

If MEASLES now or within last 3 months, Classify

Signs	Classify as	Treatment
• Any general danger sign or<br>• Clouding of cornea or<br>• Deep or extensive mouth ulcers.	***Pink:*** **SEVERE COMPLICATED MEASLES******	■ **Give Vitamin A treatment**<br>■ **Give first dose of an appropriate antibiotic**<br>■ **If clouding of the cornea or pus draining from the eye, apply tetracycline eye ointment**<br>■ **Refer URGENTLY to hospital**
• Pus draining from the eye or<br>• Mouth ulcers.	***Yellow:*** **MEASLES WITH EYE OR MOUTH COMPLICATIONS******	■ **Give Vitamin A treatment**<br>■ **If pus draining from the eye, treat eye infection with tetracycline eye ointment**<br>■ If mouth ulcers, treat with gentian violet<br>■ Follow-up in 3 days
• Measles now or within the last 3 months.	***Green:*** **MEASLES**	■ **Give Vitamin A treatment**

* These temperatures are based on axillary temperature. Rectal temperature readings are approximately 0.5°C higher.

**Look for local tenderness; oral sores; refusal to use a limb; hot tender swelling; red tender skin or boils; lower abdominal pain or pain on passing urine in older children.

*** If no malaria test available: High malaria risk - classify as MALARIA; Low malaria risk AND NO obvious cause of fever - classify as MALARIA.

**** *Other important complications of measles - pneumonia, stridor, diarrhoea, ear infection, and acute malnutrition - are classified in other tables.*

Does the child have an ear problem?

If yes, ask:

- Is there ear pain?
- Is there ear discharge?
  If yes, for how long?

Look and feel:

- Look for pus draining from the ear.
- Feel for tender swelling behind the ear.

Classify EAR PROBLEM

Signs	Classify as	Treatment
• Tender swelling behind the ear.	***Pink:*** **MASTOIDITIS**	■ **Give first dose of an appropriate antibiotic**<br>■ **Give first dose of paracetamol for pain**<br>■ **Refer URGENTLY to hospital**
• Pus is seen draining from the ear and discharge is reported for less than 14 days, or<br>• Ear pain.	***Yellow:*** **ACUTE EAR INFECTION**	■ **Give an antibiotic for 5 days**<br>■ **Give paracetamol for pain**<br>■ Dry the ear by wicking<br>■ Follow-up in 5 days
• Pus is seen draining from the ear and discharge is reported for 14 days or more.	***Yellow:*** **CHRONIC EAR INFECTION**	■ Dry the ear by wicking<br>■ Treat with topical quinolone eardrops for 14 days<br>■ Follow-up in 5 days
• No ear pain and<br>No pus seen draining from the ear.	***Green:*** **NO EAR INFECTION**	■ No treatment

THEN CHECK FOR ACUTE MALNUTRITION

CHECK FOR ACUTE MALNUTRITION

LOOK AND FEEL:

Look for signs of acute malnutrition

- Look for oedema of both feet.
- Determine WFH/L* ___ z-score.
- Measure MUAC**____ mm in a child 6 months or older.

If WFH/L less than -3 z-scores or MUAC less than 115 mm, then:

- **Check for any medical complication present:**
  - **Any general danger signs**
  - **Any severe classification**
  - **Pneumonia with chest indrawing**
- **If no medical complications present:**
  - **Child is 6 months or older, offer RUTF*** to eat. Is the child:**

    Not able to finish RUTF portion?

    Able to finish RUTF portion?
  - **Child is less than 6 months, assess breastfeeding:**

    Does the child have a breastfeeding problem?

Classify NUTRITIONAL STATUS

Signs	Classify as	Treatment
• Oedema of both feet<br>**OR**<br>• WFH/L less than -3 z-scores **OR** MUAC less than 115 mm **AND any one of the following:**<br>○ Medical complication present or<br>○ Not able to finish RUTF or<br>○ Breastfeeding problem.	***Pink:***<br>**COMPLICATED SEVERE ACUTE MALNUTRITION**	▪ **Give first dose appropriate antibiotic**<br>▪ **Treat the child to prevent low blood sugar**<br>▪ **Keep the child warm**<br>▪ **Refer URGENTLY to hospital**
• WFH/L less than -3 z-scores<br>**OR**<br>• MUAC less than 115 mm<br>**AND**<br>• Able to finish RUTF.	***Yellow:***<br>**UNCOMPLICATED SEVERE ACUTE MALNUTRITION**	▪ **Give oral antibiotics for 5 days**<br>▪ Give ready-to-use therapeutic food for a child aged 6 months or more<br>▪ Counsel the mother on how to feed the child.<br>▪ Assess for possible TB infection<br>▪ Advise mother when to return immediately<br>▪ Follow up in 7 days
• WFH/L between -3 and -2 z-scores<br>**OR**<br>• MUAC 115 up to 125 mm.	***Yellow:***<br>**MODERATE ACUTE MALNUTRITION**	▪ Assess the child's feeding and counsel the mother on the feeding recommendations<br>▪ If feeding problem, follow up in 7 days<br>▪ Assess for possible TB infection.<br>▪ Advise mother when to return immediately<br>▪ Follow-up in 30 days
• WFH/L - 2 z-scores or more<br>**OR**<br>• MUAC 125 mm or more.	***Green:***<br>**NO ACUTE MALNUTRITION**	▪ If child is less than 2 years old, assess the child's feeding and counsel the mother on feeding according to the feeding recommendations<br>▪ If feeding problem, follow-up in 7 days

*WFH/L is Weight-for-Height or Weight-for-Length determined by using the WHO growth standards charts.

** MUAC is Mid-Upper Arm Circumference measured using MUAC tape in all children 6 months or older.

***RUTF is Ready-to-Use Therapeutic Food for conducting the appetite test and feeding children with severe acute malanutrition.

THEN CHECK FOR ANAEMIA

Check for anaemia

- Look for palmar pallor. Is it:
  - Severe palmar pallor*?
  - Some palmar pallor?

Classify ANAEMIA **Classification arrow**

• Severe palmar pallor	***Pink:*** **SEVERE ANAEMIA**	■ **Refer URGENTLY to hopsital**
• Some pallor	***Yellow:*** **ANAEMIA**	■ Give iron**<br>■ Give mebendazole if child is 1 year or older and has not had a dose in the previous 6 months<br>■ Advise mother when to return immediately<br>■ Follow-up in 14 days
• No palmar pallor	***Green:*** **NO ANAEMIA**	■ • If child is less than 2 years old, assess the child's feeding and counsel the mother according to the feeding recommendations<br>○ If feeding problem, follow-up in 5 days

*Assess for sickle cell anaemia if common in your area.

**If child has severe acute malnutrition and is receiving RUTF, DO NOT give iron because there is already adequate amount of iron in RUTF.

THEN CHECK FOR HIV INFECTION

Use this chart if the child is **NOT** enrolled in HIV care.

ASK

Has the mother or child had an HIV test?

IF YES:

Decide HIV status:

- Mother: POSITIVE or NEGATIVE
- Child:
  - Virological test POSITIVE or NEGATIVE
  - Serological test POSITIVE or NEGATIVE

If mother is HIV positive and child is negative or unknown, ASK:

- Was the child breastfeeding at the time or 6 weeks before the test?
- Is the child breastfeeding now?
- If breastfeeding ASK: Is the mother and child on ARV prophylaxis?

IF NO, THEN TEST:

- Mother and child status unknown: TEST mother.
- Mother HIV positive and child status unknown: TEST child.

Classify HIV status

Signs	Classify as	Treatment
• Positive virological test in child<br>OR<br>• Positive serological test in a child 18 months or older	***Yellow:***<br>**CONFIRMED HIV INFECTION**	■ **Initiate ART treatment and HIV care**<br>■ **Give cotrimoxazole prophylaxis***<br>■ Assess the child's feeding and provide appropriate counselling to the mother<br>■ Advise the mother on home care<br>■ Asess or refer for TB assessment and INH preventive therapy<br>■ Follow-up regularly as per national guidelines
• Mother HIV-positive AND negative virological test in a breastfeeding child or only stopped less than 6 weeks ago<br>OR<br>• Mother HIV-positive, child not yet tested<br>OR<br>• Positive serological test in a child less than 18 months old	***Yellow:***<br>**HIV EXPOSED**	■ **Give cotrimoxazole prophylaxis**<br>■ **Start or continue ARV prophylaxis as recommended**<br>■ Do virological test to confirm HIV status**<br>■ Assess the child's feeding and provide appropriate counselling to the mother<br>■ Advise the mother on home care<br>■ Follow-up regularly as per national guidelines
• Negative HIV test in mother or child	***Green:***<br>**HIV INFECTION UNLIKELY**	■ Treat, counsel and follow-up existing infections

* Give cotrimoxazole prophylaxis to all HIV infected and HIV-exposed children utill confirmed negative after cessation of breastfeeding.

** If virological test is negative, repeat test 6 weeks after the breatfeeding has stopped; if serological test is positive, do a virological test as soon as possible.

THEN CHECK THE CHILD'S IMMUNIZATION, VITAMIN A AND DEWORMING STATUS

IMMUNIZATION SCHEDULE: Follow national guidelines

AGE	VACCINE				
**Birth**	BCG*	OPV-0	Hep B0		
**6 weeks**	DPT+HIB-1	OPV-1	Hep B1	RTV1	PCV1***
**10 weeks**	DPT+HIB-2	OPV-2	Hep B2	RTV2	PCV2
**14 weeks**	DPT+HIB-3	OPV-3	Hep B3	RTV3	PCV3
**9 months**	Measles **				
**18 months**	DPT				

VITAMIN A SUPPLEMENTATION

Give every child a dose of Vitamin A every six months from the age of 6 months. Record the dose on the child's chart.

ROUTINE WORM TREATMENT

Give every child mebendazole every 6 months from the age of one year. Record the dose on the child's card.

***Children who are HIV positive or unknown HIV status with symptoms consistent with HIV should not be vaccinated.*

***Second dose of measles vaccine may be given at any opportunistic moment during periodic supplementary immunization activities as early as one month following the first dose.*

****HIV-positive infants and pre-term neonates who have received 3 primary vaccine doses before 12 months of age may benefit from a booster dose in the second year of life.*

ASSESS OTHER PROBLEMS:

MAKE SURE CHILD WITH ANY GENERAL DANGER SIGN IS REFERRED after first dose of an appropriate antibiotic and other urgent treatments. Treat all children with a general danger sign to prevent low blood sugar.

HIV TESTING AND INTERPRENTING RESULTS

HIV testing is RECOMMENDED for:

- All children with unknown HIV status especially those born to HIV-positive mothers. (If you do not know the mother's status, test the mother first, if possible)

Types of HIV Tests		
	**What does the test detect?**	**How to interpret the test?**
**SEROLOGICAL TESTS** (Including rapid tests)	These tests **detect antibodies made by immune cells in response to HIV**.<br>They do not detect the HIV virus itself.	HIV antibodies pass from the mother to the child. Most antibodies have gone by 12 months of age, but in some instances they do not disappear until the child is 18 months of age.<br>This means that a positive serological test in children less than 18 months in **NOT** a reliable way to check for infection of the child.
**VIROLOGICAL TESTS** (Including DNA or RNA PCR)	These tests **directly detect the presence of the HIV virus** or products of the virus in the blood.	Positive virological (PCR) tests reliably detect HIV infection at any age, even before the child is 18 months old.<br>If the tests are negative and the child has been breastfeeding, this does not rule out infection. The baby may have just become infected.<br>Tests should be done six weeks or more after breastfeeding has completely stopped—only then do the tests reliably rule out infection.

For HIV exposed children 18 months or older, a positive HIV antibody test result means the child is infected.

For HIV exposed children less than 18 months of age:

- If PCR or other virological test is available, test from 4 - 6 weeks of age.
  - A positive result means the child is infected.
  - A negative result means the child is not infected, but could become infected if they are still breast feeding.
- If PCR or other virological test is not available, use HIV antibody test. A positive result is consistent with the fact that the child has been exposed to HIV, but does not tell us if the child is definitely infected.

Interpreting the HIV Antibody Test Results in a Child less than 18 Months of Age		
Breastfeeding status	POSITIVE (+) test	NEGATIVE (-) test
NOT BREASTFEEDING, and has not in last 6 weeks	HIV EXPOSED and/or HIV infected - Manage as if they could be infected. Repeat test at 18 months.	HIV negative Child is not HIV infected
BREASTFEEDING	HIV EXPOSED and/or HIV infected - Manage as if they could be infected. Repeat test at 18 months or once breastfeeding has been discontinued for more than 6 weeks.	Child can still be infected by breastfeeding. Repeat test once breastfeeding has been discontinued for more than 6 weeks.

WHO PAEDIATRIC STAGING FOR HIV INFECTION

This is used for monitoring children during follow up to determine clinical response to ARV treatment. Determine the clinical stage by assessing the child's signs and symptoms. Look at the classification for each stage. Decide what is the highest stage applicable to the child where one or more of the child's symptoms are represented.

	**Stage 1**<br>**Asymptomatic**	**Stage 2**<br>**Mild Disease**	**Stage 3**<br>**Moderate Disease**	**Stage 4**<br>**Severe Disease (AIDS)**
	-	-	Unexplained severe acute malnutrition not responding to standard therapy	Severe unexplained wasting/stunting/severe acute malnutrition not responding to standard therapy
Symptoms/Signs	No symptoms, or only:<br>Persistent generalized lymphadenopathy (PGL)	• Enlarged liver and/or spleen<br>• Enlarged parotid<br>• Skin conditions (prurigo, seborraic dermatitis, extensive molluscum contagiosum or warts, fungal nail infection herpes zoster)<br>• Mouth conditions recurrent mouth ulcerations, linea gingival Erythema)<br>• Recurrent or chronic upper respiratory tract infections (sinusitis, ear infection, tonsilitis, ortorrhea)	• Oral thrush (outside neonatal period).<br>• Oral hairy leukoplakia.<br>• Unexplained and unresponsive to standard therapy:<br>○ Diarhoea for over 14 days<br>○ Fever for over 1 month<br>○ Thrombocytopenia*(under 50,000/mm3 for 1month<br>○ Neutropenia* (under 500/mm3 for 1 month)<br>○ Anaemia for over 1 month (haemoglobin under 8 gm)*<br>• Recurrent severe bacterial pneumonia<br>• Pulmonary TB<br>• Lymp node TB<br>• Symptomatic lymphoid interstitial pneumonitis (LIP)*<br>• Acute necrotising ulcerative gingivitis/periodontitis<br>• Chronic HIV associated lung diseses including bronchiectasis*	• Oesophageal thrush<br>• More than one month of herpes simplex ulcerations.<br>• Severe multiple or recurrent bacteria infections > 2 episodes in a year (not including pneumonia) pneumocystis pneumonia (PCP)*<br>• Kaposi's sarcoma.<br>• Extrapulmonary tuberculosis.<br>• Toxoplasma brain abscess*<br>• Cryptococcal meningitis*<br>• Acquired HIVassociated rectal fistula<br>• HIV encephalopathy*

*Conditions requiring diagnosis by a doctor or medical officer - should be referred for appropriate diagnosis and treatment.

TREAT THE CHILD

CARRY OUT THE TREATMENT STEPS IDENTIFIED ON THE *ASSESS AND CLASSIFY* CHART

TEACH THE MOTHER TO GIVE ORAL DRUGS AT HOME

Follow the instructions below for every oral drug to be given at home.
Also follow the instructions listed with each drug's dosage table.

- **Determine the appropriate drugs and dosage for the child's age or weight.**
- **Tell the mother the reason for giving the drug to the child.**
- **Demonstrate how to measure a dose.**
- **Watch the mother practise measuring a dose by herself.**
- **Ask the mother to give the first dose to her child.**
- **Explain carefully how to give the drug, then label and package the drug.**
- **If more than one drug will be given, collect, count and package each drug separately.**
- **Explain that all the oral drug tablets or syrups must be used to finish the course of treatment, even if the child gets better.**
- **Check the mother's understanding before she leaves the clinic.**

Give an Appropriate Oral Antibiotic

- **FOR PNEUMONIA, ACUTE EAR INFECTION:**

FIRST-LINE ANTIBIOTIC: Oral Amoxicillin

AGE or WEIGHT	AMOXICILLIN* *Give two times daily for 5 days*	
	TABLET 250 mg	SYRUP 250mg/5 ml
2 months up to 12 months (4 - <10 kg)	1	5 ml
12 months up to 3 years (10 - <14 kg)	2	10 ml
3 years up to 5 years (14-19 kg)	3	15 ml

** Amoxicillin is the recommended first-line drug of choice in the treatment of pneumonia due to its efficacy and increasing high resistance to cotrimoxazole.*

- **FOR PROPHYLAXIS IN HIV CONFIRMED OR EXPOSED CHILD:**

ANTIBIOTIC FOR PROPHYLAXIS: Oral Cotrimoxazole

AGE	COTRIMOXAZOLE (trimethoprim + sulfamethoxazole) Give once a day starting at 4-6 weeks of age		
	Syrup (40/200 mg/5ml)	Paediatric tablet (Single strength 20/100 mg)	Adult tablet (Single strength 80/400 mg)
Less than 6 months	2.5 ml	1	-
6 months up to 5 years	5 ml	2	1/2

- **FOR DYSENTERY give Ciprofloxacine**

FIRST-LINE ANTIBIOTIC: Oral Ciprofloxacine

AGE	CIPROFLOXACINE Give 15mg/kg two times daily for 3 days	
	250 mg tablet	500 mg tablet
Less than 6 months	1/2	1/4
6 months up to 5 years	1	1/2

- **FOR CHOLERA:**

FIRST-LINE ANTIBIOTIC FOR CHOLERA: ____
SECOND-LINE ANTIBIOTIC FOR CHOLERA: ____

AGE or WEIGHT	ERYTHROMYCIN *Give four times daily for 3 days*	TETRACYCLINE *Give four times daily for 3 days*
	TABLET 250 mg	TABLET 250 mg
2 years up to 5 years (10 - 19 kg)	1	1

TEACH THE MOTHER TO GIVE ORAL DRUGS AT HOME

Follow the instructions below for every oral drug to be given at home.
Also follow the instructions listed with each drug's dosage table.

Give Inhaled Salbutamol for Wheezing

USE OF A SPACER*

A spacer is a way of delivering the bronchodilator drugs effectively into the lungs. No child under 5 years should be given an inhaler without a spacer. A spacer works as well as a nebuliser if correctly used.

- From salbutamol metered dose inhaler (100 μg/puff) give 2 puffs.
- Repeat up to 3 times every 15 minutes before classifying pneumonia.

Spacers can be made in the following way:

- Use a 500ml drink bottle or similar.
- Cut a hole in the bottle base in the same shape as the mouthpiece of the inhaler. This can be done using a sharp knife.
- Cut the bottle between the upper quarter and the lower 3/4 and disregard the upper quarter of the bottle.
- Cut a small V in the border of the large open part of the bottle to fit to the child's nose and be used as a mask.
- Flame the edge of the cut bottle with a candle or a lighter to soften it.
- In a small baby, a mask can be made by making a similar hole in a plastic (not polystyrene) cup.
- Alternatively commercial spacers can be used if available.

To use an inhaler with a spacer:

- Remove the inhaler cap. Shake the inhaler well.
- Insert mouthpiece of the inhaler through the hole in the bottle or plastic cup.
- The child should put the opening of the bottle into his mouth and breath in and out through the mouth.
- A carer then presses down the inhaler and sprays into the bottle while the child continues to breath normally.
- Wait for three to four breaths and repeat.
- For younger children place the cup over the child's mouth and use as a spacer in the same way.

** If a spacer is being used for the first time, it should be primed by 4-5 extra puffs from the inhaler.*

Give Oral Antimalarial for MALARIA

- **If Artemether-Lumefantrine (AL)**
  - Give the first dose of artemether-lumefantrine in the clinic and observe for one hour. If the child vomits within an hour repeat the dose.
  - Give second dose at home after 8 hours.
  - Then twice daily for further two days as shown below.
  - Artemether-lumefantrine should be taken with food.
- **If Artesunate Amodiaquine (AS+AQ)**
  - Give first dose in the clinic and observe for an hour, if a child vomits within an hour repeat the dose.
  - Then daily for two days as per table below using the fixed dose combination.

**WEIGHT (age)**	**Artemether-Lumefantrine tablets** (20 mg artemether and 120 mg lumefantrine) Give two times daily for 3 days			**Artesunate plus Amodiaquine tablets** Give Once a day for 3 days					
				(25 mg AS/67.5 mg AQ)			(50 mg AS/135 mg AQ)		
	Day 1	Day 2	day 3	Day 1	Day 2	Day 3	Day 1	Day 2	Day 3
5 - <10 kg (2 months up to 12 months)	1	1	1	1	1	1	-	-	-
10 - <14 kg (12 months up to 3 years)	1	1	1	-	-	-	1	1	1
14 - <19 kg (3 years up to 5 years)	2	2	2	-	-	-	1	1	1

Give Paracetamol for High Fever (> 38.5°C) or Ear Pain

- Give paracetamol every 6 hours until high fever or ear pain is gone.

**AGE or WEIGHT**	**PARACETAMOL**	
	**TABLET** (100 mg)	**TABLET** (500 mg)
2 months up to 3 years (4 - <14 kg)	1	1/4
3 years up to 5 years (14 - <19 kg)	1 1/2	1/2

TEACH THE MOTHER TO GIVE ORAL DRUGS AT HOME

Follow the instructions below for every oral drug to be given at home.
Also follow the instructions listed with each drug's dosage table.

*Give Iron**

- Give one dose daily for 14 days.

AGE or WEIGHT	IRON/FOLATE TABLET	IRON SYRUP
	Ferrous sulfate 200 mg + 250 µg Folate (60 mg elemental iron)	Ferrous fumarate 100 mg per 5 ml (20 mg elemental iron per ml)
2 months up to 4 months (4 - <6 kg)		1.00 ml (< 1/4 tsp.)
4 months up to 12 months (6 - <10 kg)		1.25 ml (1/4 tsp.)
12 months up to 3 years (10 - <14 kg)	1/2 tablet	2.00 ml (<1/2 tsp.)
3 years up to 5 years (14 - 19 kg)	1/2 tablet	2.5 ml (1/2 tsp.)

* Children with severe acute malnutrition who are receiving ready-to-use therapeutic food (RUTF) should not be given Iron.

TEACH THE MOTHER TO TREAT LOCAL INFECTIONS AT HOME

- **Explain to the mother what the treatment is and why it should be given.**
- **Describe the treatment steps listed in the appropriate box.**
- **Watch the mother as she does the first treatment in the clinic (except for remedy for cough or sore throat).**
- **Tell her how often to do the treatment at home.**
- **If needed for treatment at home, give mother the tube of tetracycline ointment or a small bottle of gentian violet.**
- **Check the mothers understanding before she leaves the clinic.**

Soothe the Throat, Relieve the Cough with a Safe Remedy

- **Safe remedies to recommend:**
  - Breast milk for a breastfed infant.

____

____

- **Harmful remedies to discourage:**

____

____

____

Treat Eye Infection with Tetracycline Eye Ointment

- **Clean both eyes 4 times daily**.
  - Wash hands.
  - Use clean cloth and water to gently wipe away pus.
- **Then apply tetracycline eye ointment in both eyes 4 times daily.**
  - Squirt a small amount of ointment on the inside of the lower lid.
  - Wash hands again.
- **Treat until there is no pus discharge.**
- **Do not put anything else in the eye.**

*Clear the Ear by Dry Wicking and Give Eardrops**

- **Dry the ear at least 3 times daily.**
  - Roll clean absorbent cloth or soft, strong tissue paper into a wick.
  - Place the wick in the child's ear.
  - Remove the wick when wet.
  - Replace the wick with a clean one and repeat these steps until the ear is dry.
  - Instill quinolone eardrops after dry wicking three times daily for two weeks.

** Quinolone eardrops may include ciprofloxacin, norfloxacin, or ofloxacin.*

Treat for Mouth Ulcers with Gentian Violet (GV)

- **Treat for mouth ulcers twice daily.**
  - Wash hands.
  - Wash the child's mouth with clean soft cloth wrapped around the finger and wet with salt water.
  - Paint the mouth with half-strength gentian violet (0.25% dilution).
  - Wash hands again.
  - Continue using GV for 48 hours after the ulcers have been cured.
  - Give paracetamol for pain relief.

Treat Thrush with Nystatin

Treat thrush four times daily for 7 days

- Wash hands
- Wet a clean soft cloth with salt water and use it to wash the child's mouth
- Instill nystatin 1ml four times a day
- Avoid feeding for 20 minutes after medication
- If breastfed check mother's breasts for thrush. If present treat with nystatin
- Advise mother to wash breasts after feeds. If bottle fed advise change to cup and spoon
- Give paracetamol if needed for pain

GIVE VITAMIN A AND MEBENDAZOLE IN CLINIC

- **Explain to the mother why the drug is given**
- **Determine the dose appropriate for the child's weight (or age)**
- **Measure the dose accurately**

Give Vitamin A Supplementation and Treatment

VITAMIN A SUPPLEMENTATION:

- Give first dose any time after 6 months of age to ALL CHILDREN
- Thereafter vitamin A **every six months** to ALL CHILDREN

VITAMIN A TREATMENT:

- Give an extra dose of Vitamin A (same dose as for supplementation) for ***treatment*** if the child has MEASLES or PERSISTENT DIARRHOEA. If the child has had a dose of vitamin A within the past month or is on RUTF for treatment of severe acute malnutrition, DO NOT GIVE VITAMIN A.
- Always record the dose of Vitamin A given on the child's card.

AGE	VITAMIN A DOSE
6 up to 12 months	100 000 IU
One year and older	200 000 IU

Give Mebendazole

- Give 500 mg mebendazole as a single dose in clinic if:
  - hookworm/whipworm are a problem in children in your area, and
  - the child is 1 years of age or older, and
  - the child has not had a dose in the previous 6 months.

GIVE THESE TREATMENTS IN THE CLINIC ONLY

- **Explain to the mother why the drug is given.**
- **Determine the dose appropriate for the child's weight (or age).**
- **Use a sterile needle and sterile syringe when giving an injection.**
- **Measure the dose accurately.**
- **Give the drug as an intramuscular injection.**
- **If child cannot be referred, follow the instructions provided.**

Give Intramuscular Antibiotics

GIVE TO CHILDREN BEING REFERRED URGENTLY

- Give Ampicillin (50 mg/kg) and Gentamicin (7.5 mg/kg).

AMPICILLIN

- Dilute 500mg vial with 2.1ml of sterile water (500mg/2.5ml).
- IF REFERRAL IS NOT POSSIBLE OR DELAYED, repeat the ampicillin injection every 6 hours.
- **Where there is a strong suspicion of meningitis, the dose of ampicillin can be increased 4 times.**

GENTAMICIN

- 7.5 mg/kg/day once daily

AGE or WEIGHT	**AMPICILLIN** 500 mg vial	**GENTAMICIN** 2ml/40 mg/ml vial
2 up to 4 months (4 - <6 kg)	1 m	0.5-1.0 ml
4 up to 12 months (6 - <10 kg)	2 ml	1.1-1.8 ml
12 months up to 3 years (10 - <14 kg)	3 ml	1.9-2.7 ml
3 years up to 5 years (14 - 19 kg)	5 m	2.8-3.5 ml

Give Diazepam to Stop Convulsions

- Turn the child to his/her side and clear the airway. Avoid putting things in the mouth.
- Give 0.5mg/kg diazepam injection solution per rectum using a small syringe without a needle (like a tuberculin syringe) or using a catheter.
- Check for low blood sugar, then treat or prevent.
- Give oxygen and REFER
- If convulsions have not stopped after 10 minutes repeat diazepam dose

**AGE or WEIGHT**	**DIAZEPAM** 10mg/2mls
2 months up to 6 months (5 - 7 kg)	0.5 ml
6 months up to 12months (7 - <10 kg)	1.0 ml
12 months up to 3 years (10 - <14 kg)	1.5 ml
3 years up to 5 years (14-19 kg)	2.0 ml

Give Artesunate Suppositories or Intramuscular Artesunate or Quinine for Severe Malaria

FOR CHILDREN BEING REFERRED WITH VERY SEVERE FEBRILE DISEASE:

- Check which pre-referral treatment is available in your clinic (rectal artesunate suppositories, artesunate injection or quinine).
- Artesunate suppository: Insert first dose of the suppository and refer child urgently
- Intramuscular artesunate or quinine: Give first dose and refer child urgently to hospital.

IF REFERRAL IS NOT POSSIBLE:

- For artesunate injection:
  - Give first dose of artesunate intramuscular injection
  - Repeat dose after 12 hrs and daily until the child can take orally
  - Give full dose of oral antimlarial as soon as the child is able to take orally.

  For artesunate suppository:
  - Give first dose of suppository
  - Repeat the same dose of suppository every 24 hours until the child can take oral antimalarial.
  - Give full dose of oral antimalarial as soon as the child is able to take orally
- For quinine:
  - Give first dose of intramuscular quinine.
  - The child should remain lying down for one hour.
  - Repeat the quinine injection at 4 and 8 hours later, and then every 12 hours until the child is able to take an oral antimalarial. Do not continue quinine injections for more than 1 week.

  If low risk of malaria, do not give quinine to a child less than 4 months of age.

**AGE or WEIGHT**	**RECTAL ARTESUNATE SUPPOSITORY**		**INTRAMUSCULAR ARTESUNATE**	**INTRAMUSCULAR QUININE**	
	50 mg suppositories Dosage 10 mg/kg	200 mg suppositories Dosage 10 mg/kg	60 mg vial (20mg/ml) 2.4 mg/kg	150 mg/ml* (in 2 ml ampoules)	300 mg/ml* (in 2 ml ampoules)
2 months up to 4 months (4 - <6 kg)	1		1/2 ml	0.4 ml	0.2 ml
4 months up to 12 months (6 - <10 kg)	2		1 ml	0.6 ml	0.3 ml
12 months up to 2 years (10 - <12 kg)	2	-	1.5 ml	0.8 ml	0.4 ml
2 years up to 3 years (12 - <14 kg)	3	1	1.5 ml	1.0 ml	0.5 ml
3 years up to 5 years (14 - 19 kg)	3	1	2 ml	1.2 ml	0.6 ml

** quinine salt*

GIVE THESE TREATMENTS IN THE CLINIC ONLY

Treat the Child to Prevent Low Blood Sugar

- **If the child is able to breastfeed:**
  - Ask the mother to breastfeed the child.
- **If the child is not able to breastfeed but is able to swallow:**
  - Give expressed breast milk or a breast-milk substitute.
  - If neither of these is available, give sugar water*.
  - Give 30 - 50 ml of milk or sugar water* before departure.
- **If the child is not able to swallow:**
  - Give 50 ml of milk or sugar water* by nasogastric tube.
  - If no nasogastric tube available, give 1 teaspoon of sugar moistened with 1-2 drops of water sublingually and repeat doses every 20 minutes to prevent relapse.
  - ** To make sugar water: Dissolve 4 level teaspoons of sugar (20 grams) in a 200-ml cup of clean water.*

GIVE EXTRA FLUID FOR DIARRHOEA AND CONTINUE FEEDING

(See FOOD advice on COUNSEL THE MOTHER chart)

PLAN A: TREAT DIARRHOEA AT HOME

Counsel the mother on the 4 Rules of Home Treatment:

1. **Give Extra Fluid**
2. **Give Zinc Supplements (age 2 months up to 5 years)**
3. **Continue Feeding**
4. **When to Return.**

1. ***GIVE EXTRA FLUID*** **(as much as the child will take)**
   - **TELL THE MOTHER:**
     - Breastfeed frequently and for longer at each feed.
     - If the child is exclusively breastfed, give ORS or clean water in addition to breast milk.
     - If the child is not exclusively breastfed, give one or more of the following: ORS solution, food-based fluids (such as soup, rice water, and yoghurt drinks), or clean water.
   - **It is especially important to give ORS at home when:**
     - *the child has been treated with Plan B or Plan C during this visit.*
     - *the child cannot return to a clinic if the diarrhoea gets worse.*
   - **TEACH THE MOTHER HOW TO MIX AND GIVE ORS. GIVE THE MOTHER 2 PACKETS OF ORS TO USE AT HOME.**
   - **SHOW THE MOTHER HOW MUCH FLUID TO GIVE IN ADDITION TO THE USUAL FLUID INTAKE:**

Up to 2 years	50 to 100 ml after each loose stool
2 years or more	100 to 200 ml after each loose stool

Tell the mother to:
- Give frequent small sips from a cup.
- If the child vomits, wait 10 minutes. Then continue, but more slowly.
- Continue giving extra fluid until the diarrhoea stops.

2. ***GIVE ZINC*** **(age 2 months up to 5 years)**
   - **TELL THE MOTHER HOW MUCH ZINC TO GIVE (20 mg tab):**

2 months up to 6 months	1/2 tablet daily for 14 days
6 months or more	1 tablet daily for 14 days

   - **SHOW THE MOTHER HOW TO GIVE ZINC SUPPLEMENTS**
     - Infants - dissolve tablet in a small amount of expressed breast milk, ORS or clean water in a cup.
     - Older children - tablets can be chewed or dissolved in a small amount of water.
3. ***CONTINUE FEEDING*** **(exclusive breastfeeding if age less than 6 months)**
4. ***WHEN TO RETURN***

PLAN B: TREAT SOME DEHYDRATION WITH ORS

In the clinic, give recommended amount of ORS over 4-hour period

- **DETERMINE AMOUNT OF ORS TO GIVE DURING FIRST 4 HOURS**

WEIGHT	< 6 kg	6 - <10 kg	10 - <12 kg	12 - 19 kg
**AGE***	**Up to 4 months**	**4 months up to 12 months**	**12 months up to 2 years**	**2 years up to 5 years**
**In ml**	200 - 450	450 - 800	800 - 960	960 - 1600

** Use the child's age only when you do not know the weight. The approximate amount of ORS required (in ml) can also be calculated by multiplying the child's weight (in kg) times 75.*

  - If the child wants more ORS than shown, give more.
  - For infants under 6 months who are not breastfed, also give 100 - 200 ml clean water during this period if you use standard ORS. This is not needed if you use new low osmolarity ORS.
- **SHOW THE MOTHER HOW TO GIVE ORS SOLUTION.**
  - Give frequent small sips from a cup.
  - If the child vomits, wait 10 minutes. Then continue, but more slowly.
  - Continue breastfeeding whenever the child wants.
- **AFTER 4 HOURS:**
  - Reassess the child and classify the child for dehydration.
  - Select the appropriate plan to continue treatment.
  - Begin feeding the child in clinic.
- **IF THE MOTHER MUST LEAVE BEFORE COMPLETING TREATMENT:**
  - Show her how to prepare ORS solution at home.
  - Show her how much ORS to give to finish 4-hour treatment at home.
  - Give her enough ORS packets to complete rehydration. Also give her 2 packets as recommended in **Plan A**.
  - Explain the 4 Rules of Home Treatment:
    1. **GIVE EXTRA FLUID**
    2. **GIVE ZINC (age 2 months up to 5 years)**
    3. **CONTINUE FEEDING (exclusive breastfeeding if age less than 6 months)**
    4. **WHEN TO RETURN**

↺

GIVE EXTRA FLUID FOR DIARRHOEA AND CONTINUE FEEDING

PLAN C: TREAT SEVERE DEHYDRATION QUICKLY

FOLLOW THE ARROWS. IF ANSWER IS "YES", GO ACROSS. IF "NO", GO DOWN.

START HERE

Can you give intravenous (IV) fluid immediately? YES→

- **Start IV fluid immediately.** If the child can drink, give ORS by mouth while the drip is set up. Give 100 ml/kg Ringer's Lactate Solution (or, if not available, normal saline), divided as follows

AGE	First give 30 ml/kg in:	Then give 70 ml/kg in:
Infants (under 12 months)	1 hour*	5 hours
Children (12 months up to 5 years)	30 minutes*	2 1/2 hours

* Repeat once if radial pulse is still very weak or not detectable.

- **Reassess the child every 1-2 hours.** If hydration status is not improving, give the IV drip more rapidly.
- Also give ORS (about 5 ml/kg/hour) as soon as the child can drink: usually after 3-4 hours (infants) or 1-2 hours (children).
- Reassess an infant after 6 hours and a child after 3 hours. Classify dehydration. Then choose the appropriate plan (A, B, or C) to continue treatment.

NO
↓

Is IV treatment available nearby (within 30 minutes)? YES→

- **Refer URGENTLY to hospital for IV treatment.**
- If the child can drink, provide the mother with ORS solution and show her how to give frequent sips during the trip or give ORS by naso-gastric tube.

NO
↓

Are you trained to use a naso-gastric (NG) tube for rehydration? YES→

NO
↓

Can the child drink? YES→

- **Start rehydration by tube (or mouth) with ORS solution:** give 20 ml/kg/hour for 6 hours (total of 120 ml/kg).
- **Reassess the child every 1-2 hours while waiting for transfer:**
  - If there is repeated vomiting or increasing abdominal distension, give the fluid more slowly.
  - If hydration status is not improving after 3 hours, send the child for IV therapy.
- After 6 hours, reassess the child. Classify dehydration. Then choose the appropriate plan (A, B or C) to continue treatment.

NO
↓

Refer URGENTLY to hospital for IV or NG treatment

NOTE:

- If the child is not referred to hospital, observe the child at least 6 hours after rehydration to be sure the mother can maintain hydration giving the child ORS solution by mouth.

GIVE READY-TO-USE THERAPEUTIC FOOD

Give Ready-to-Use Therapeutic Food for SEVERE ACUTE MALNUTRITION

- Wash hands before giving the ready-to-use therapeutic food (RUTF).
- Sit with the child on the lap and gently offer the ready-to-use therapeutic food.
- Encourage the child to eat the RUTF without forced feeding.
- Give small, regular meals of RUTF and encourage the child to eat often 5–6 meals per day.
- If still breastfeeding, continue by offering breast milk first before every RUTF feed.
- Give only the RUTF for at least two weeks, if breastfeeding continue to breast and gradually introduce foods recommended for the age (See Feeding recommendations in *COUNSEL THE MOTHER* chart).
- When introducing recommended foods, ensure that the child completes his daily ration of RUTF before giving other foods.
- Offer plenty of clean water, to drink from a cup, when the child is eating the ready-to-use therapeutic food**.**

Recommended Amounts of Ready-to-Use Therapeutic Food

CHILD'S WEIGHT (kg)	**Packets per day** (92 g Packets Containing 500 kcal)	**Packets per Week Supply**
4.0-4.9 kg	2.0	14
5.0-6.9 kg	2.5	18
7.0-8.4 kg	3.0	21
8.5-9.4 kg	3.5	25
9.5-10.4 kg	4.0	28
10.5-11.9 kg	4.5	32
>12.0 kg	5.0	35

TREAT THE HIV INFECTED CHILD

Steps when Initiating ART in Children

All children less than 5 years who are HIV infected should be initiated on ART irrespective of CD4 count or clinical stage.

Remember that if a child has any general danger sign or a severe classification, he or she needs URGENT REFERRAL. ART initiation is not urgent, and the child should be stabilized first.

**STEP 1: DECIDE IF THE CHILD HAS CONFIRMED HIV INFECTION**<br>**Child is under 18 months:**<br>**HIV infection is confirmed if virological test (PCR) is positive**<br>**Child is over 18 months:**<br>▪ **Two different serological tests are positive**<br>▪ **Send any further confirmatory tests required**<br>**If results are discordant, refer**<br>***If HIV infection is confirmed, and child is in stable condition, GO TO STEP 2***	**STEP 3: DECIDE IF ART CAN BE INITIATED IN YOUR FACILITY**<br>**If child is less than 3 kg or has TB, Refer for ART initiation.**<br>**If child weighs 3 kg or more and does not have TB, *GO TO STEP 4***
**STEP 2: DECIDE IF CAREGIVER IS ABLE TO GIVE ART**<br>**Check that the caregiver is willing and able to give ART. The caregiver should ideally have disclosed the child's HIV status to another adult who can assist with providing ART, or be part of a support group.**<br>▪ **Caregiver able to give ART: *GO TO STEP 3***<br>▪ **Caregiver not able: classify as CONFIRMED HIV INFECTION but NOT ON ART. Counsel and support the caregiver. Follow-up regularly. Move to the step 3 once the caregiver is willing and able to give ART.**	**STEP 4: RECORD BASELINE INFORMATION ON THE CHILD'S HIV TREATMENT CARD**<br>**Record the following information:**<br>▪ **Weight and height**<br>▪ **Pallor if present**<br>▪ **Feeding problem if present**<br>▪ **Laboratory results (if available): Hb, viral load, CD4 count and percentage. Send for any laboratory tests that are required. Do not wait for results. GO TO STEP 5**
**STEP 5: START ON ART, COTRIMOXAZOLE PROPHYLAXIS AND ROUTINE TREATMENTS**<br>▪ **Initiate ART treatement:**<br>• **Child up to 3 years: ABC or AZT +3TC+ LPV/R or recommended first-line regimen**<br>• **Child 3 years or older: ABC + 3TC + EFV, or recommended first-line regimen.**<br>▪ **Give co-trimoxazole prophylaxis**<br>▪ **Give other routine treatments, including Vitamin A and immunizations**<br>▪ **Follow-up regularly as per national guidelines**	

TREAT THE HIV INFECTED CHILD

Preferred and Alternative ARV Regimens

AGE	Preferred	Alternative	Children with TB/HIV Infection
Birth up to 3 YEARS	ABC or AZT + 3TC + LPV/r	ABC or AZT + 3TC + NVP	ABC or AZT + 3TC + NVP
			AZT + 3TC + ABC
3 years and older	ABC + 3TC + EFV	ABC or AZT + 3TC + EFV or NVP	ABC or AZT + 3TC + EFV
			AZT + 3TC + ABC

Give Antiretroviral Drugs (Fixed Dose Combinations)

WEIGHT (Kg)	AZT/3TC Twice daily		AZT/3TC/NVP Twice daily		ABC/AZT/3TC Twice daily		ABC/3TC Twice daily	
	60/30 mg tablet	300/150 mg tablet	60/30/50 mg tablet	300/150/200 mg tablet	60/60/30 mg tablet	300/300/150 mg tablet	60/30 mg tablet	600/300 mg tablet
3 - 5.9	1	-	1	-	1	-	1	-
6 - 9.9	1.5	-	1.5	-	1.5	-	1.5	-
10 - 13.9	2	-	2	-	2	-	2	-
14 - 19.9	2.5	-	2.5	-	2.5	-	2.5	-
20 - 24.9	3	-	3	-	3	-	3	-
25 - 34.9	-	1		1		1	-	0.5

TREAT THE HIV INFECTED CHILD

Give Antiretroviral Drugs

LOPINAVIR / RITONAVIR (LPV/r), NEVIRAPINE (NVP) & EFAVIRENZ (EFV)

WEIGHT (KG)	LOPINAVIR / RITONAVIR (LPV/r) Target dose 230-350mg/m^2 twice daily		NEVIRAPINE (NVP)			EFAVIRENZ (EFV) Target dose 15 mg/Kg once daily
	80/20 mg liquid	100/25 mg tablet	10 mg/ml liquid	50 mg tablet	200 mg tablet	200 mg tablet
	Twice daily	Twice daily	Twice daily	Twice daily	Twice daily	Once daily
**3 - 5.9**	**1 ml**	**-**	**5 ml**	**1**	**-**	**-**
**6 - 9.9**	**1.5 ml**	**-**	**8 ml**	**1.5**	**-**	**-**
**10 - 13.9**	**2 ml**	**2**	**10 ml**	**2**	**-**	**1**
**14 - 19.9**	**2.5 ml**	**2**	**-**	**2.5**	**-**	**1.5**
**20 - 24.9**	**3 ml**	**2**	**-**	**3**	**-**	**1.5**
**25 - 34.9**	**-**	**3**	**-**	**-**	**1**	**2**

ABACAVIR (ABC), ZIDOVUDINE (AZT or ZDV) & LAMIVUDINE (3TC)

WEIGHT (KG)	ABACAVIR (ABC) Target dose: 8mg/Kg/dose twice daily			ZIDOVUDINE (AZT or ZDV) Target dose 180-240mg/m^2 twice daily			LAMIVUDINE (3TC)		
	20 mg/ml liquid	60 mg dispersible tablet	300 mg tablet	10 mg/ml liquid	60 mg tablet	300 mg tablet	10 mg/ml liquid	30 mg tablet	150 mg tablet
	Twice daily	Twice daily	Twice daily	Twice daily	Twice daily	Twice daily	Twice daily	Twice daily	Twice daily
**3 - 5.9**	**3 ml**	**1**	**-**	**6 ml**	**1**	**-**	**3 ml**	**1**	**-**
**6 - 9.9**	**4 ml**	**1.5**	**-**	**9 ml**	**1.5**	**-**	**4 ml**	**1.5**	**-**
**10 - 13.9**	**6 ml**	**2**	**-**	**12 ml**	**2**	**-**	**6 ml**	**2**	**-**
**14 - 19.9**	**-**	**2.5**	**-**	**-**	**2.5**	**-**	**-**	**2.5**	**-**
**20 - 24.9**	**-**	**3**	**-**	**-**	**3**	**-**	**-**	**3**	**-**
**25 - 34.9**	**-**	**-**	**1**	**-**	**-**	**1**	**-**	**-**	**1**

TREAT THE HIV INFECTED CHILD

Side Effects ARV Drugs

	Very common side-effets:	Potentially serious side effects:	Side effects occurring later during treatment:
	■ warn patients and suggest ways patients can manage;<br>■ manage when patients seek care	■ warn patients and tell them to seek care	■ discuss with patients
Abacavir (ABC)		Seek care urgently:<br>Fever, vomiting, rash - this may indicate hypersensitivity to abacavir	
Lamivudine (3TC)	Nausea<br>Diarrhoea		
Lopinavir/ritonavir	Nausea<br>Vomiting<br>Diarrhoea		Changes in fat distribution:<br>Arms, legs, buttocks, cheeks become THIN<br>Breasts, tummy, back of neck become FAT<br>Elevated blood cholesterol and glucose
Nevirapine (NVP)	Nausea<br>Diarrhoea	Seek care urgently:<br>Yellow eyes<br>Severe skin rash<br>Fatigue AND shortness of breath<br>Fever	
Zidovudine<br>(ZDV or AZT)	Nausea<br>Diarrhoea<br>Headache<br>Fatigue<br>Muscle pain	Seek care urgently:<br>Pallor (anaemia)	
Efavirenz (EFV)	Nausea<br>Diarrhoea<br>Strange dreams<br>Difficulty sleeping<br>Memory problems<br>Headache<br>Dizziness	Seek care urgently:<br>Yellow eyes<br>Psychosis or confusion<br>Severe skin rash	

TREAT THE HIV INFECTED CHILD

Manage Side Effects of ARV Drugs

SIGNS or SYMPTOMS	APPROPRIATE CARE RESPONSE
**Yellow eyes (jaundice) or abdominal pain**	Stop drugs and REFER URGENTLY
**Rash**	If on **abacavir**, assess carefully. Is it a dry or wet lesion? Call for advice. If the rash is severe, generalized, or peeling, involves the mucosa or is associated with fever or vomiting: stop drugs and REFER URGENTLY
**Nausea**	Advise that the drug should be given with food. If persists for more than 2 weeks or worsens, call for advice or refer.
**Vomiting**	Children may commonly vomit medication. Repeat the dose if the medication is seen in the vomitus, or if vomiting occurred 30 minutes of the dose being given.<br>**If vomiting persists**, the caregiver should bring the child to clinic for evaluation.<br>**If vomiting everything, or vomiting associated with severe abdominal pain or difficulty breathing, REFER URGENTLY.**
**Diarrhoea**	Assess, classify, and treat using diarrhoea charts. Reassure mother that if due to ARV, it will improve in a few weeks. Follow-up as per chart booklet. If not improved after two weeks, call for advice or refer.
**Fever**	Assess, classify, and treat using feve chart.
**Headache**	Give paracetamol. If on efavirenz, reassure that this is common and usually self-limiting. If persists for more than 2 weeks or worsens, call for advice or refer.
**Sleep disturbances, nightmares, anxiety**	This may be due to **efavirenz**. Give at night and take on an empty stomach with low-fat foods. If persists for more than 2 weeks or worsens, call for advice or refer.
**Tingling, numb or painful feet or legs**	If new or worse on treatment, call for advice or refer.
**Changes in fat distribution**	Consider switching from stavudine to abacavir, consider to viral load. Refer if needed.

TREAT THE HIV INFECTED CHILD

Give Pain Relief to HIV Infected Child

- Give paracetamol or ibuprofen every 6 hours if pain persists.
- For severe pain, morphine syrup can be given.

AGE or WEIGHT	PARACETAMOL		ORAL MORPHINE (0.5 mg/5 ml)
	**TABLET** (100 mg)	**SYRUP** (120 mg/5ml)	
2 up to 4 months (4 - <6 kg)	-	2 ml	0.5 ml
4 up to 12 months (6 - <10 kg)	1	2.5 ml	2 ml
12 months up to 2 years (10 - <12 kg)	1 1/2	5 ml	3 ml
2 up to 3 years (12 - <14 kg)	2	7.5 ml	4 ml
3 up to 5 years (14 -<19 kg)	2	10 ml	5 ml

Recommended dosages for ibuprofen: 5-10 mg/kg orally, every 6-8h to a maximum of 500 mg per day i.e. ¼ of a 200 mg tablet below 15 kg , ½ tablet for 15 up to 20 kg of body weight. Avoid ibuprofen in children under the age of 3 months.

IMMUNIZE EVERY SICK CHILD AS NEEDED

FOLLOW-UP

GIVE FOLLOW-UP CARE FOR ACUTE CONDITIONS

- **Care for the child who returns for follow-up using all the boxes that match the child's previous classifications.**
- **If the child has any new problem, assess, classify and treat the new problem as on the *ASSESS AND CLASSIFY* chart.**

PNEUMONIA

After 3 days:

Check the child for general danger signs.
Assess the child for cough or difficult breathing.
Ask:

- Is the child breathing slower?
- Is there a chest indrawing?
- Is there less fever?
- Is the child eating better?

} See *ASSESS & CLASSIFY* chart.

Treatment:

- If ***any general danger sign or stridor,*** refer URGENTLY to hospital.
- If ***chest indrawing and/or breathing rate, fever and eating are the same or worse,*** refer URGENTLY to hospital.
- If ***breathing slower, no chest indrawing, less fever, and eating better***, complete the 5 days of antibiotic.

PERSISTENT DIARRHOEA

After 5 days:
Ask:

- Has the diarrhoea stopped?
- How many loose stools is the child having per day?

Treatment:

- If ***the diarrhoea has not stopped*** (child is still having 3 or more loose stools per day), do a full reassessment of the child. Treat for dehydration if present. Then refer to hospital.
- If ***the diarrhoea has stopped*** (child having less than 3 loose stools per day), tell the mother to follow the usual feeding recommendations for the child's age.

DYSENTERY

After 3 days:
Assess the child for diarrhoea. > See *ASSESS & CLASSIFY* chart.

Ask:

- Are there fewer stools?
- Is there less blood in the stool?
- Is there less fever?
- Is there less abdominal pain?
- Is the child eating better?

Treatment:

- If the child is ***dehydrated***, treat dehydration.
- If ***number of stools, amount of blood in stools, fever, abdominal pain, or eating are worse or the same***:
  - Change to second-line oral antibiotic recommended for dysentery in your area. Give it for 5 days. Advise the mother to return in 3 days. If you do not have the second line antibiotic, REFER to hospital.

Exceptions - if the child:

- is less than 12 months old, or
- was dehydrated on the first visit, or
- if he had measles within the last 3 months

} REFER to hospital.

- If ***fewer stools, less blood in the stools, less fever, less abdominal pain, and eating better***, continue giving ciprofloxacin until finished.

Ensure that mother understands the oral rehydration method fully and that she also understands the need for an extra meal each day for a week.

MALARIA

If fever persists after 3 days:
Do a full reassessment of the child. > See *ASSESS & CLASSIFY* chart.

DO NOT REPEAT the Rapid Diagnostic Test if it was positive on the initial visit.

Treatment:

- If the child has ***any general danger sign or stiff neck***, treat as VERY SEVERE FEBRILE DISEASE.
- If the child has any **other*cause of fever other than malaria***, provide appropriate treatment.
- If there is ***no other apparent cause of fever***:
  - If fever has been present for 7 days, refer for assessment.
  - Do microscopy to look for malaria parasites. If parasites are present and the child has finished a full course of the first line antimalarial, give the second-line antimalarial, if available, or refer the child to a hospital.
  - If there is no other apparent cause of fever and you do not have a microscopy to check for parasites, refer the child to a hospital.

GIVE FOLLOW-UP CARE FOR ACUTE CONDITIONS

FEVER: NO MALARIA

If fever persists after 3 days:
Do a full reassessment of the child. > See *ASSESS & CLASSIFY* chart.
Repeat the malaria test.

Treatment:
- If the child has ***any general danger sign or stiff neck***, treat as VERY SEVERE FEBRILE DISEASE.
- If a child has a ***positive malaria test***, give first-line oral antimalarial. Advise the mother to return in 3 days if the fever persists.
- If the child has any **other *cause of fever other than malaria***, provide treatment.
- If there is ***no other apparent cause*** of fever:
  - If the fever has been present for 7 days, refer for assessment.

MEASLES WITH EYE OR MOUTH COMPLICATIONS, GUM OR MOUTH ULCERS, OR THRUSH

After 3 days:
Look for red eyes and pus draining from the eyes.
Look at mouth ulcers or white patches in the mouth (thrush).
Smell the mouth.

Treatment for eye infection:
- If ***pus is draining from the eye***, ask the mother to describe how she has treated the eye infection. If treatment has been correct, refer to hospital. If treatment has not been correct, teach mother correct treatment.
- If ***the pus is gone but redness remains***, continue the treatment.
- If ***no pus or redness***, stop the treatment.

Treatment for mouth ulcers:
- If ***mouth ulcers are worse, or there is a very foul smell from the mouth***, refer to hospital.
- If ***mouth ulcers are the same or better***, continue using half-strength gentian violet for a total of 5 days.

Treatment for thrush:
- If ***thrush is worse*** check that treatment is being given correctly.
- If the child has ***problems with swallowing***, refer to hospital.
- If ***thrush is the same or better***, and the child is feeding well, continue nystatine for a total of 7 days.

EAR INFECTION

After 5 days:
Reassess for ear problem. > See *ASSESS & CLASSIFY* chart.
Measure the child's temperature.

Treatment:
- If there is ***tender swelling behind the ear or high fever (38.5°C or above)***, refer URGENTLY to hospital.
- ***Acute ear infection:***
  - If ***ear pain or discharge*** persists, treat with 5 more days of the same antibiotic. Continue wicking to dry the ear. Follow-up in 5 days.
  - If ***no ear pain or discharge***, praise the mother for her careful treatment. If she has not yet finished the 5 days of antibiotic, tell her to use all of it before stopping.
- ***Chronic ear infection:***
  - Check that the mother is wicking the ear correctly and giving quinolone drops tree times a day. Encourage her to continue.

FEEDING PROBLEM

After 7 days:
Reassess feeding. > See questions in the *COUNSEL THE MOTHER* chart.
Ask about any feeding problems found on the initial visit.

- Counsel the mother about any new or continuing feeding problems. If you counsel the mother to make significant changes in feeding, ask her to bring the child back again.
- If the child is classified as MODERATE ACUTE MALNUTRITION, ask the mother to return 30 days after the initial visit to measure the child's WFH/L, MUAC.

ANAEMIA

After 14 days:
- Give iron. Advise mother to return in 14 days for more iron.
- Continue giving iron every 14 days for 2 months.
- If the child has palmar pallor after 2 months, refer for assessment.

GIVE FOLLOW-UP CARE FOR ACUTE CONDITIONS

UNCOMPLICATED SEVERE ACUTE MALNUTRITION

After 14 days or during regular follow up:

Do a full reassessment of the child. > See *ASSESS & CLASSIFY* chart.

Assess child with the same measurements (WFH/L, MUAC) as on the initial visit.

Check for oedema of both feet.

Check the child's appetite by offering ready-to use therapeutic food if the child is 6 months or older.

Treatment:

- If the child has **COMPLICATED SEVERE ACUTE MALNUTRITION** (WFH/L less than -3 z-scores or MUAC is less than 115 mm or oedema of both feet AND has developed a medical complication or oedema, or fails the appetite test), refer URGENTLY to hospital.
- If the child has **UNCOMPLICATED SEVERE ACUTE MALNUTRITION** (WFH/L less than -3 z-scores or MUAC is less than 115 mm or oedema of both feet but NO medical complication and passes appetite test), counsel the mother and encourage her to continue with appropriate RUTF feeding. Ask mother to return again in 14 days.
- If the child has **MODERATE ACUTE MALNUTRITION** (WFH/L between -3 and -2 z-scores or MUAC between 115 and 125 mm), advise the mother to continue RUTF. Counsel her to start other foods according to the age appropriate feeding recommendations (see COUNSEL THE MOTHER chart). Tell her to return again in 14 days. Continue to see the child every 14 days until the child's WFH/L is -2 z-scores or more, and/or MUAC is 125 mm or more.
- If the child has **NO ACUTE MALNUTRITION** (WFH/L is -2 z-scores or more, or MUAC is 125 mm or more), praise the mother, STOP RUTF and counsel her about the age appropriate feeding recommendations (see COUNSEL THE MOTHER chart).

MODERATE ACUTE MALNUTRITION

After 30 days:

Assess the child using the same measurement (WFH/L or MUAC) used on the initial visit:

- If WFH/L, weigh the child, measure height or length and determine if WFH/L.
- If MUAC, measure using MUAC tape.
- Check the child for oedema of both feet.

Reassess feeding. See questions in the COUNSEL THE MOTHER chart.

Treatment:

- If the child is no longer classified as **MODERATE ACUTE MALNUTRITION**, praise the mother and encourage her to continue.
- If the child is still classified as **MODERATE ACUTE MALNUTRITION**, counsel the mother about any feeding problem found. Ask the mother to return again in one month. Continue to see the child monthly until the child is feeding well and gaining weight regularly or his or her WFH/L is -2 z-scores or more or MUAC is 125 mm. or more.

Exception:

If you do not think that feeding will improve, or if the child has lost weight or his or her MUAC has diminished, refer the child.

GIVE FOLLOW-UP CARE FOR HIV EXPOSED AND INFECTED CHILD

HIV EXPOSED

Follow up regularly as per national guidelines.

At each follow-up visit follow these instructions:

- Ask the mother: Does the child have any problems?
- Do a full assessment including checking for mouth or gum problems, treat, counsel and follow up any new problem
- Provide routine child health care: Vitamin A, deworming, immunization, and feeding assessment and counselling
- Continue cotrimoxazole prophylaxis
- Continue ARV prophylaxis if ARV drugs and breastfeeding are recommended; check adherence: How often, if ever, does the child/mother miss a dose?
- Ask about the mother's health. Provide HIV counselling and testing and referral if necessary
- Plan for the next follow-up visit

HIV testing:

- If new HIV test result became available since the last visit, reclassify the child for HIV according to the test result.
- Recheck child's HIV status six weeks after cessation of breastfeeding. Reclassify the child according to the test result.

If child is confirmed HIV infected

- Start on ART and enrol in chronic HIV care.
- Continue follow-up as for CONFIRMED HIV INFECTION ON ART

If child is confirmed uninfected

- Continue with co-trimoxazole prophylaxis if breastfeeding or stop if the test resuls are after 6 weeks of cessation of breastfeeding.
- Counsel mother on preventing HIV infection through breastfeeding and about her own health

CONFIRMED HIV INFECTION NOT ON ART

Follow up regularly as per national guidelines.

At each follow-up visit follow these instructions:

- Ask the mother: Does the child have any problems?
- Do a full assessment including checking for mouth or gum problems, treat, counsel and follow up any new problem
- Counsel and check if mother able or willing now to initiate ART for the child.
- Provide routine child health care: Vitamin A, deworming, immunization, and feeding assessment and counselling
- Continue cotrimoxazole prophylaxis if indicated.
- Initiate or continue isoniazid preventive therapy if indicated.
- If no acute illness and mother is willing, initiate ART (See Box Steps when Initiating ART in children)
- Monitor CD4 count and percentage.
- Ask about the mother's health, provide HIV counselling and testing.
- Home care:
  - Counsel the mother about any new or continuing problems
  - If appropriate, put the family in touch with organizations or people who could provide support
  - Advise the mother about hygiene in the home, in particular when preparing food
- Plan for the next follow-up visit

GIVE FOLLOW-UP CARE FOR HIV EXPOSED AND INFECTED CHILD

CONFIRMED HIV INFECTION ON ART: THE FOUR STEPS OF FOLLOW-UP CARE

Follow up regularly as per national guidelines.

STEP 1: ASSESS AND CLASSIFY

- **ASK: Does the child have any problems?**

Has the child received care at another health facility since the last visit?

- **CHECK: for general danger signs -** If present, complete assessment, give pre-referral treatment, **REFER URGENTLY.**
- ASSESS, CLASSIFY, TREAT and COUNSEL any sick child as appropriate.
- CHECK for ART severe side effects
  - Severe skin rash
  - Difficulty breathing and severe abdominal pain
  - Yellow eyes
  - Fever, vomiting, rash (only if on Abacavir)

  } If present, give any pre-referral treatment, REFER URGENTLY
- Check for other ART side effects

STEP 2: MONITOR PROGRESS ON ART

- **IF ANY OF FOLLOWING PRESENT, REFER NON-URGENTLY:**

- **Record the Child's weight and height**
- Assess adherence
  - Ask about adherence: how often, if ever, does the child miss a dose? Record your assessment.
- **Assess and record clinical stage**
  - Assess clinical stage. Compare with the child's stage at previous visits.
- **Monitor laboratory results**
  - Record results of tests that have been sent.

If any of these present, refer NON-URGENTLY:

- Not gaining weight for 3 months
- Loss of milestones
- Poor adherence
- Stage worse than before
- CD4 count lower than before
- LDL higher than 3.5 mmol/L
- TG higher than 5.6 mmol/L

- **Manage side effects**
- **Send tests that are due**

STEP 3: PROVIDE ART, COTRIMOXAZOLE AND ROUTINE TREATMENTS

- **If child is stable:** continue with the ART regimen and cotrimoxazole doses.
- **Check for appropriate doses:** remember these will need to increase as the child grows
- **Give routine care:** Vitamin A supplementation, deworming, and immunization as needed

STEP 4: COUNSEL THE MOTHER OR CAREGIVER

Use every visit to educate and provide support to the mother or caregiver

- **Key issues to discuss include:**

How the child is progressing, feeding, adherence, side-effects and correct management, disclosure (to others and the child), support for the caregiver

- **Remember to check that the mother and other family members are receiving the care that they need**
- **Set a follow-up visit:** if well, follow-up as per nastional guidelines. If problems, follow-up as indicated.

COUNSEL THE MOTHER

FEEDING COUNSELLING

Assess Child's Appetite

All children aged 6 months or more with SEVERE ACUTE MALNUTRITION (oedema of both feet or WFH/L less than -3 z-scores or MUAC less than 115 mm) and no medical complication should be assessed for appetite.

Appetite is assessed on the initial visit and at each follow-up visit to the health facility. Arrange a quiet corner where the child and mother can take their time to get accustomed to eating the RUTF. Usually the child eats the RUTF portion in 30 minutes.

Explain to the mother:

- The purpose of assessing the child's appetite.
- What is ready-to-use-therapeutic food (RUTF).
- How to give RUTF:
  - Wash hands before giving the RUTF.
  - Sit with the child on the lap and gently offer the child RUTF to eat.
  - Encourage the child to eat the RUTF without feeding by force.
  - Offer plenty of clean water to drink from a cup when the child is eating the RUTF.

Offer appropriate amount of RUTF to the child to eat:

- After 30 minutes check if the child was able to finish or not able to finish the amount of RUTF given and decide:
  - Child **ABLE** to finish at least one-third of a packet of RUTF portion (92 g) or 3 teaspoons from a pot within 30 minutes.
  - Child **NOT ABLE** to eat one-third of a packet of RUTF portion (92 g) or 3 teaspoons from a pot within 30 minutes.

FEEDING COUNSELLING

Assess Child's Feeding

Assess feeding if child is Less Than 2 Years Old, Has MODERATE ACUTE MALNUTRITION, ANAEMIA, CONFIRMED HIV INFECTION, or is HIV EXPOSED. Ask questions about the child's usual feeding and feeding during this illness. Compare the mother's answers to the ***Feeding Recommendations*** for the child's age.

ASK - How are you feeding your child?

- **If the child is receiving *any* breast milk, ASK**:
  - How many times during the day?
  - Do you also breastfeed during the night?

- **Does the child take any other food or fluids?**
  - What food or fluids?
  - How many times per day?
  - What do you use to feed the child?
- **If MODERATE ACUTE MALNUTRITION or if a child with CONFIRMED HIV INFECTION fails to gain weight or loses weight between monthly measurements, ASK:**
  - How large are servings?
  - Does the child receive his own serving?
  - Who feeds the child and how?
  - What foods are available in the home?
- **During this illness, has the child's feeding changed?**
  - If yes, how?

In addition, for HIV EXPOSED child:

- **If mother and child are on ARV treatment or prophylaxis and child breastfeeding, ASK:**
  - **Do you take ARV drugs? Do you take all doses, miss doses, do not take medication?**
  - **Does the child take ARV drugs (If the policy is to take ARV prophylaxis until 1 week after breastfeeding has stopped)? Does he or she take all doses, missed doses, does not take medication?**
- **If child not breastfeeding, ASK:**
  - **What milk are you giving?**
  - **How many times during the day and night?**
  - **How much is given at each feed?**
  - **How are you preparing the milk?**
    - **Let the mother demonstrate or explain how a feed is prepared, and how it is given to the infant.**
  - **Are you giving any breast milk at all?**
  - **Are you able to get new supplies of milk before you run out?**
  - **How is the milk being given? Cup or bottle?**
  - **How are you cleaning the feeding utensils?**

FEEDING COUNSELLING

Feeding Recommendations

Feeding recommendations FOR ALL CHILDREN during sickness and health, and including HIV EXPOSED children on ARV prophylaxis

Newborn, birth up to 1 week	1 week up to 6 months	6 up to 9 months	9 up to 12 months	12 months up to 2 years	2 years and older
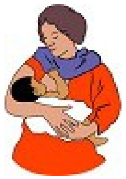	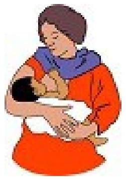	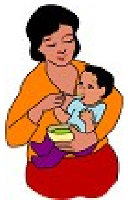	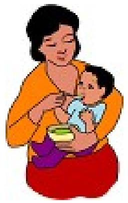	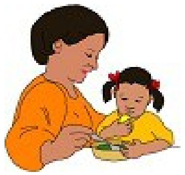	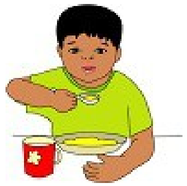
▪ Immediately after birth, put your baby in skin to skin contact with you.<br>▪ Allow your baby to take the breast within the first hour. Give your baby colostrum, the first yellowish, thick milk. It protects the baby from many Illnesses.<br>▪ Breastfeed day and night, as often as your baby wants, at least 8 times In 24 hours. Frequent feeding produces more milk.<br>▪ If your baby is small (low birth weight), feed at least every 2 to 3 hours. Wake the baby for feeding after 3 hours, if baby does not wake self.<br>▪ DO NOT give other foods or fluids. Breast milk is all your baby needs. This is especially important for infants of HIV-positive mothers. Mixed feeding increases the risk of HIV mother-to-child transmission when compared to exclusive breastfeeding.	▪ Breastfeed as often as your child wants. Look for signs of hunger, such as beginning to fuss, sucking fingers, or moving lips.<br>▪ Breastfeed day and night whenever your baby wants, at least 8 times in 24 hours. Frequent feeding produces more milk.<br>▪ Do not give other foods or fluids. Breast milk is all your baby needs.	▪ Breastfeed as often as your child wants.<br>▪ Also give thick porridge or well-mashed foods, including animal-source foods and vitamin A-rich fruits and vegetables.<br>▪ Start by giving 2 to 3 tablespoons of food. Gradually increase to 1/2 cups (1 cup = 250 ml).<br>▪ Give 2 to 3 meals each day.<br>▪ Offer 1 or 2 snacks each day between meals when the child seems hungry.	▪ Breastfeed as often as your child wants.<br>▪ Also give a variety of mashed or finely chopped family food, including animal-source foods and vitamin A-rich fruits and vegetables.<br>▪ Give 1/2 cup at each meal(1 cup = 250 ml).<br>▪ Give 3 to 4 meals each day.<br>▪ Offer 1 or 2 snacks between meals. The child will eat if hungry.<br>▪ For snacks, give small chewable items that the child can hold. Let your child try to eat the snack, but provide help if needed.	▪ Breastfeed as often as your child wants.<br>▪ Also give a variety of mashed or finely chopped family food, including animal-source foods and vitamin A-rich fruits and vegetables.<br>▪ Give 3/4 cup at each meal (1 cup = 250 ml).<br>▪ Give 3 to 4 meals each day.<br>▪ Offer 1 to 2 snacks between meals.<br>▪ Continue to feed your child slowly, patiently. Encourage—but do not force—your child to eat.	▪ Give a variety of family foods to your child, including animal-source foods and vitamin A-rich fruits and vegetables.<br>▪ Give at least 1 full cup (250 ml) at each meal.<br>▪ Give 3 to 4 meals each day.<br>▪ Offer 1 or 2 snacks between meals.<br>▪ If your child refuses a new food, offer "tastes" several times. Show that you like the food. Be patient.<br>▪ Talk with your child during a meal, and keep eye contact.

A good daily diet should be adequate in quantity and include an energy-rich food (for example, thick cereal with added oil); meat, fish, eggs, or pulses; and fruits and vegetables.

FEEDING COUNSELLING

Feeding Recommendations for HIV EXPOSED Child on Infant Formula

These feeding recommendations are for HIV EXPOSED children in setting where the national authorities recommend to avoid all breastfeeding or when the mother has chosen formula feeding.

PMTCT: If the baby is on AZT for prophylaxis, continue until 4 to 6 weeks of age.

Up to 6 months

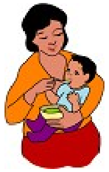

- **FORMULA FEED** exclusively. Do not give any breast milk. Other foods or fluids are not necessary.
- Prepare correct strength and amount just before use. Use milk within two hours. Discard any left over—a fridge can store formula for 24 hours.
- Cup feeding is safer than bottle feeding. Clean the cup and utensils with hot soapy water.

Give the following amounts of formula 8 to 6 times per day:

Age in months	Approx. amount and times per day
0 up to 1	60 ml x 8
1 up to 2	90 ml x 7
2 up to 4	120 ml x 6
4 up to 6	150 ml x 6

6 up to 12 monts

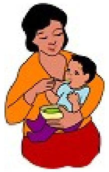

- Give 1-2 cups (250 - 500 ml) of infant formula or boiled, then cooled, full cream milk. Give milk with a cup, not a bottle.
- Give:

  ____

  ____

  ____

  ______________________________ *

- Start by giving 2-3 tablespoons of food 2 - 3 times a day. Gradually increase to 1/2 cup (1 cup = 250 ml) at each meal and to giving meals 3-4 times a day.
- Offer 1-2 snacks each day when the child seems hungry.
- For snacks give small chewable items that the child can hold. Let your child try to eat the snack, but provide help if needed.

12 months up to 2 years

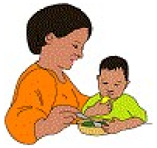

- Give 1-2 cups (250 - 500 ml) of boiled, then cooled, full cream milk or infant formula.
- Give milk with a cup, not a bottle.
- Give:

  ____

  ____

  ____

  ______________________________ *

or family foods 3 or 4 times per day. Give 3/4 cup (1 cup = 250 ml) at each meal.

- Offer 1-2 snacks between meals.
- Continue to feed your child slowly, patiently.
- Encourage - but do not force - your child to eat.

Safe preparation of replacement feeding

Infant formula

- Always use a marked cup or glass and spoon to measure water and the scoop to measure the formula powder.
- Wash your hands before preparing a feed.
- Bring the water to boil and then let it cool. Keep it covered while it cools.
- Measure the formula powder into a marked cup or glass. Make the scoops level. Put in one scoop for every 25 ml of water.
- Add a small amount of the cooled boiled water and stir. Fill the cup or glass to the mark with the water. Stir well.
- Feed the infant using a cup.
- Wash the utensils.

Cow's milk

- Cow' s or other animal milks are not suitable for infants below 6 months of age (even modified).
- For a child between 6 and 12 month of age: boil the milk and let it cool (even if pasteurized).
- Feed the baby using a cup.

** A good daily diet should be adequate in quantity and include an energy-rich food (for example, thick cereal with added oil); meat, fish, eggs, or pulses; and fruits and vegetables.*

FEEDING COUNSELLING

Stopping Breastfeeding

STOPPING BREASTFEEDING means changing from all breast milk to no breast milk.

This should happen gradually over one month. Plan in advance for a safe transition.

1. HELP MOTHER PREPARE:

- Mother should discuss and plan in advance with her family, if possible
- Express milk and give by cup
- Find a regular supply or formula or other milk (e.g. full cream cow's milk)
- Learn how to prepare a store milk safely at home

2. HELP MOTHER MAKE TRANSITION:

- Teach mother to cup feed (See chart booklet Counsel part in Assess, classify and treat the sick young infant aged up to 2 months)
- Clean all utensils with soap and water
- Start giving only formula or cow's milk once baby takes all feeds by cup

3. STOP BREASTFEEDING COMPLETELY:

- Express and discard enough breast milk to keep comfortable until lactation stops

Feeding Recommendations For a Child Who Has PERSISTENT DIARRHOEA

- If still breastfeeding, give more frequent, longer breastfeeds, day and night.
- If taking other milk:
  - replace with increased breastfeeding OR
  - replace with fermented milk products, such as yoghurt OR
  - replace half the milk with nutrient-rich semisolid food.
- For other foods, follow feeding recommendations for the child's age.

EXTRA FLUIDS AND MOTHER'S HEALTH

Advise the Mother to Increase Fluid During Illness

- **FOR ANY SICK CHILD:**
  - Breastfeed more frequently and for longer at each feed. If child is taking breast-milk substitutes, increase the amount of milk given.
  - Increase other fluids. For example, give soup, rice water, yoghurt drinks or clean water.

- **FOR CHILD WITH DIARRHOEA:**
  - Giving extra fluid can be lifesaving. Give fluid according to Plan A or Plan B on *TREAT THE CHILD* chart.

Counsel the Mother about her Own Health

- If the mother is sick, provide care for her, or refer her for help.
- If she has a breast problem (such as engorgement, sore nipples, breast infection), provide care for her or refer her for help.
- Advise her to eat well to keep up her own strength and health.
- Check the mother's immunization status and give her tetanus toxoid if needed.
- Make sure she has access to:
  - Family planning
  - Counselling on STD and AIDS prevention.

Give additional counselling if the mother is HIV-positive

- Reassure her that with regular follow-up, much can be done to prevent serious illness, and maintain her and the child’s health
- Emphasize good hygiene, and early treatment of illnesses

WHEN TO RETURN

Advise the Mother When to Return to Health Worker

FOLLOW-UP VISIT: Advise the mother to come for follow-up at the earliest time listed for the child's problems.

If the child has:	Return for follow-up in:
■ PNEUMONIA<br>■ DYSENTERY<br>■ MALARIA, if fever persists<br>● FEVER: NO MALARIA, if fever persists<br>■ MEASLES WITH EYE OR MOUTH COMPLICATIONS<br>■ MOUTH OR GUM ULCERS OR THRUSH	3 days
■ PERSISTENT DIARRHOEA<br>■ ACUTE EAR INFECTION<br>■ CHRONIC EAR INFECTION<br>■ COUGH OR COLD, if not improving	5 days
■ UNCOMPLICATED SEVERE ACUTE MALNUTRITION<br>■ FEEDING PROBLEM	14 days
■ ANAEMIA	14 days
■ MODERATE ACUTE MALNUTRITION	30 days
■ CONFIRMED HIV INFECTION<br>■ HIV EXPOSED	According to national recommendations

NEXT WELL-CHILD VISIT: Advise the mother to return for next immunization according to immunization schedule.

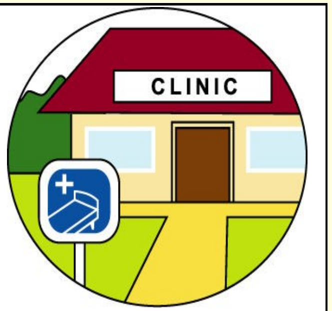

WHEN TO RETURN IMMEDIATELY

Advise mother to return immediately if the child has any of these signs:	
Any sick child	■ Not able to drink or breastfeed<br>■ Becomes sicker<br>■ Develops a fever
If child has COUGH OR COLD, also return if:	■ Fast breathing<br>■ Difficult breathing
If child has diarrhoea, also return if:	■ Blood in stool<br>■ Drinking poorly

SICK YOUNG INFANT AGE UP TO 2 MONTHS

ASSESS AND CLASSIFY THE SICK YOUNG INFANT

ASSESS

DO A RAPID APRAISAL OF ALL WAITING INFANTS

ASK THE MOTHER WHAT THE YOUNG INFANT'S PROBLEMS ARE

- Determine if this is an initial or follow-up visit for this problem.
  - if follow-up visit, use the follow-up instructions.
  - if initial visit, assess the child as follows:

CLASSIFY

USE ALL BOXES THAT MATCH THE INFANT'S SYMPTOMS AND PROBLEMS TO CLASSIFY THE ILLNESS

IDENTIFY TREATMENT

CHECK FOR VERY SEVERE DISEASE AND LOCAL BACTERIAL INFECTION

ASK:

- Is the infant having difficulty in feeding?
- Has the infant had convulsions (fits)?

LOOK, LISTEN, FEEL:

- Count the breaths in one minute. Repeat the count if more than 60 breaths per minute.
- Look for severe chest indrawing.

} YOUNG INFANT MUST BE CALM

- Measure axillary temperature.
- Look at the umbilicus. Is it red or draining pus?
- Look for skin pustules.
- Look at the young infant's movements.
  If infant is sleeping, ask the mother to wake him/her.
  - Does the infant move on his/her own?

  If the young infant is not moving, gently stimulate him/her.
  - Does the infant not move at all?

Classify ALL YOUNG INFANTS

Signs	Classify	Treatment
**Any one of the following signs**<br>• Not feeding well or<br>• Convulsions or<br>• Fast breathing (60 breaths per minute or more) or<br>• Severe chest indrawing or<br>• Fever (37.5°C* or above) or<br>• Low body temperature (less than 35.5°C*) or<br>• Movement only when stimulated or no movement at all.	***Pink:***<br>**VERY SEVERE DISEASE**	■ **Give first dose of intramuscular antibiotics**<br>■ **Treat to prevent low blood sugar**<br>■ **Refer URGENTLY to hospital ****<br>■ **Advise mother how to keep the infant warm on the way to the hospital**
• Umbilicus red or draining pus<br>• Skin pustules	***Yellow:***<br>**LOCAL BACTERIAL INFECTION**	■ **Give an appropriate oral antibiotic**<br>■ Teach the mother to treat local infections at home<br>■ Advise mother to give home care for the young infant<br>■ Follow up in 2 days
• None of the signs of very severe disease or local bacterial infection	***Green:***<br>**SEVERE DISEASE OR LOCAL INFECTION UNLIKELY**	■ Advise mother to give home care.

** These thresholds are based on axillary temperature. The thresholds for rectal temperature readings are approximately 0.5°C higher.*

*** If referral is not possible, management the sick young infant as described in the national referral care guidelines or WHO Pocket Book for hospital care for children.*

CHECK FOR JAUNDICE

If jaundice present, ASK:
- When did the jaundice appear first?

LOOK AND FEEL:
- Look for jaundice (yellow eyes or skin)
- Look at the young infant's palms and soles. Are they yellow?

CLASSIFY **JAUNDICE**

Signs	Classification	Treatment
• Any jaundice if age less than 24 hours or<br>• Yellow palms and soles at any age	***Pink:*** **SEVERE JAUNDICE**	■ ***Treat to prevent low blood sugar***<br>■ ***Refer URGENTLY to hospital*** or<br>■ ***Advise mother how to keep the infant warm on the way to the hospital***
• Jaundice appearing after 24 hours of age and<br>• Palms and soles not yellow	***Yellow:*** **JAUNDICE**	■ Advise the mother to give home care for the young infant<br>■ Advise mother to return immediately if palms and soles appear yellow.<br>■ If the young infant is older than 14 days, refer to a hospital for assessment<br>Follow-up in 1 day
• No jaundice	***Green:*** **NO JAUNDICE**	■ Advise the mother to give home care for the young infant

THEN ASK: Does the young infant have diarrhoea*?

IF YES, LOOK AND FEEL:
- Look at the young infant's general condition:
  Infant's movements
  - Does the infant move on his/her own?
  - Does the infant not move even when stimulated but then stops?
  - Does the infant not move at all?
  - Is the infant restless and irritable?
- Look for sunken eyes.
- Pinch the skin of the abdomen. Does it go back:
  - Very slowly (longer than 2 seconds)?
  - or slowly?

Classify DIARRHOEA **for DEHYDRATION**

Signs	Classification	Treatment
Two of the following signs:<br>• Movement only when stimulated or no movement at all<br>• Sunken eyes<br>• Skin pinch goes back very slowly.	***Pink:*** **SEVERE DEHYDRATION**	■ If infant has no other severe classification:<br>○ Give fluid for severe dehydration (Plan C)<br>OR<br>***If infant also has another severe classification:***<br>○ ***Refer URGENTLY to hospital with mother giving frequent sips of ORS on the way***<br>***Advise the mother to continue breastfeeding***
Two of the following signs:<br>• Restless and irritable<br>• Sunken eyes<br>• Skin pinch goes back slowly.	***Yellow:*** **SOME DEHYDRATION**	■ Give fluid and breast milk for some dehydration (Plan B)<br>■ ***If infant has any severe classification:***<br>○ ***Refer URGENTLY to hospital with mother giving frequent sips of ORS on the way***<br>○ ***Advise the mother to continue breastfeeding***<br>■ Advise mother when to return immediately<br>■ Follow-up in 2 days if not improving
Not enough signs to classify as some or severe dehydration.	***Green:*** **NO DEHYDRATION**	■ Give fluids to treat diarrhoea at home and continue breastfeeding (Plan A)<br>■ Advise mother when to return immediately<br>■ Follow-up in 2 days if not improving

*** What is diarrhoea in a young infant?**

A young infant has diarrhoea if the stools have changed from usual pattern and are many and watery (more water than faecal matter).

The normally frequent or semi-solid stools of a breastfed baby are not diarrhoea.

THEN CHECK FOR HIV INFECTION

ASK

- Has the mother and/or young infant had an HIV test?

IF YES:

- What is the mother's HIV status?:
  - Serological test POSITIVE or NEGATIVE
- What is the young infant's HIV status?:
  - Virological test POSITIVE or NEGATIVE
  - Serological test POSITIVE or NEGATIVE

If mother is HIV positive and NO positive virological test in child ASK:

- Is the young infant breastfeeding now?
- Was the young infant breastfeeding at the time of test or before it?
- Is the mother and young infant on PMTCT ARV prophylaxis?*

IF NO test: Mother and young infant status unknown

- Perform HIV test for the mother; if positive, perform virological test for the young infant

Classify HIV status

Signs	Classify	Treatment
• Positive virological test in young infant	***Yellow:*** **CONFIRMED HIV INFECTION**	■ Give cotrimoxazole prophylaxis from age 4-6 weeks<br>■ Give HIV ART and care<br>■ Advise the mother on home care<br>■ Follow-up regularly as per national guidelines
• Mother HIV positive AND negative virological test in young infant breastfeeding or if only stopped less than 6 weeks ago.<br>OR<br>• Mother HIV positive, young infant not yet tested<br>OR<br>• Positive serological test in young infant	***Yellow:*** **HIV EXPOSED**	■ Give cotrimoxazole prophylaxis from age 4-6 weeks<br>■ Start or continue PMTCT ARV prophylaxis as per national recommendations**<br>■ Do virological test at age 4-6 weeks or repeat 6 weeks after the child stops breastfeeding<br>■ Advise the mother on home care<br>■ Follow-up regularly as per national guidelines
• Negative HIV test in mother or young infant	***Green:*** **HIV INFECTION UNLIKELY**	■ Treat, counsel and follow-up existing infections

*** Prevention of Maternal-To-Child-Transmission (PMTCT) ART prophylaxis.**

****Initiate triple ART for all pregnant and lactating women with HIV infection, and put their infants on ART prophylaxis from birth for 6 weeks if breastfeeding or 4-6 weeks if on replacement feeding.**

THEN CHECK FOR FEEDING PROBLEM OR LOW WEIGHT FOR AGE

Use this table to assess feeding of all young infants except HIV-exposed young infants not breastfed. For HIV-exposed non-breastfed young infants see chart "THEN CHECK FOR FEEDING PROBLEM OR LOW WEIGHT FOR AGE IN NON-BREASTFED INFANTS"

If an infant has no indications to refer urgently to hospital:

Ask:
- Is the infant breastfed? If yes, how many times in 24 hours?
- Does the infant usually receive any other foods or drinks? If yes, how often?
- If yes, what do you use to feed the infant?

LOOK, LISTEN, FEEL:
- Determine weight for age.
- Look for ulcers or white patches in the mouth (thrush).

Classify FEEDING

Signs	Classify	Treatment
• Not well attached to breast <u>or</u><br>• Not suckling effectively <u>or</u><br>• Less than 8 breastfeeds in 24 hours <u>or</u><br>• Receives other foods or drinks <u>or</u><br>• Low weight for age <u>or</u><br>• Thrush (ulcers or white patches in mouth).	***Yellow:***<br>**FEEDING PROBLEM OR LOW WEIGHT**	■ If not well attached or not suckling effectively, teach correct positioning and attachment<br>○ If not able to attach well immediately, teach the mother to express breast milk and feed by a cup<br>■ If breastfeeding less than 8 times in 24 hours, advise to increase frequency of feeding. Advise the mother to breastfeed as often and as long as the infant wants, day and night<br>■ If receiving other foods or drinks, counsel the mother about breastfeeding more, reducing other foods or drinks, and using a cup<br>■ If not breastfeeding at all*:<br>○ Refer for breastfeeding counselling and possible relactation*<br>○ Advise about correctly preparing breast-milk substitutes and using a cup<br>■ Advise the mother how to feed and keep the low weight infant warm at home<br>■ If thrush, teach the mother to treat thrush at home<br>■ Advise mother to give home care for the young infant<br>■ Follow-up any feeding problem or thrush in 2 days<br>■ Follow-up low weight for age in 14 days
• Not low weight for age and no other signs of inadequate feeding.	***Green:***<br>**NO FEEDING PROBLEM**	■ Advise mother to give home care for the young infant<br>■ Praise the mother for feeding the infant well

ASSESS BREASTFEEDING:
- **Has the infant breastfed in the previous hour?**

  If the infant has not fed in the previous hour, ask the mother to put her infant to the breast. Observe the breastfeed for 4 minutes.

  (If the infant was fed during the last hour, ask the mother if she can wait and tell you when the infant is willing to feed again.)
  - Is the infant well attached?

    not well attached   *good attachment*
- **TO CHECK ATTACHMENT, LOOK FOR:**
  - *Chin touching breast*
  - *Mouth wide open*
  - *Lower lip turned outwards*
  - *More areola visible above than below the mouth*

  (All of these signs should be present if the attachment is good.)
- Is the infant suckling effectively (that is, slow deep sucks, sometimes pausing)?

  not suckling effectively   *suckling effectively*

Clear a blocked nose if it interferes with breastfeeding.

* Unless not breastfeeding because the mother is HIV positive.

THEN CHECK FOR FEEDING PROBLEM OR LOW WEIGHT FOR AGE IN NON-BREASTFED INFANTS

Use this chart for HIV EXPOSED infants not breastfeeding AND the infant has no indications to refer urgently to hospital:

Ask:

- What milk are you giving?
- How many times during the day and night?
- How much is given at each feed?
- How are you preparing the milk?
- Let mother demonstrate or explain how a feed is prepared, and how it is given to the infant.
- Are you giving any breast milk at all?
- What foods and fluids in addition to replacement feeds is given?
- How is the milk being given?
- Cup or bottle?
- How are you cleaning the feeding utensils?

LOOK, LISTEN, FEEL:

- Determine weight for age.
- Look for ulcers or white patches in the mouth (thrush).

Classify FEEDING

Signs	Classification	Treatment
• Milk incorrectly or unhygienically prepared <u>or</u><br>Giving inappropriate replacement feeds <u>or</u><br>Giving insufficient replacement feeds <u>or</u><br>• An HIV positive mother mixing breast and other feeds before 6 months <u>or</u><br>• Using a feeding bottle <u>or</u><br>• Low weight for age <u>or</u><br>• Thrush (ulcers or white patches in mouth).	***Yellow:***<br>**FEEDING PROBLEM OR LOW WEIGHT**	▪ Counsel about feeding<br>▪ Explain the guidelines for safe replacement feeding<br>▪ Identify concerns of mother and family about feeding.<br>▪ If mother is using a bottle, teach cup feeding<br>▪ Advise the mother how to feed and keep the low weight infant warm at home<br>▪ If thrush, teach the mother to treat thrush at home<br>▪ Advise mother to give home care for the young infant<br>▪ Follow-up any feeding problem or thrush in 2 days<br>▪ Follow-up low weight for age in 14 days
• Not low weight for age and no other signs of inadequate feeding.	***Green:***<br>**NO FEEDING PROBLEM**	▪ Advise mother to give home care for the young infant<br>▪ Praise the mother for feeding the infant well

THEN CHECK THE YOUNG INFANT'S IMMUNIZATION AND VITAMIN A STATUS:

IMMUNIZATION SCHEDULE:

AGE	VACCINE					VITAMIN A
**Birth**	BCG	OPV-0	Hep B0			200 000 IU to the mother within 6 weeks of delivery
**6 weeks**	DPT+HIB-1	OPV-1	Hep B1	RTV1	PCV1	

- **Give all missed doses on this visit.**
- Include sick infants unless being referred.
- Advise the caretaker when to return for the next dose.

ASSESS OTHER PROBLEMS

ASSESS THE MOTHER'S HEALTH NEEDS

Nutritional status and anaemia, contraception. Check hygienic practices.

TREAT THE YOUNG INFANT

GIVE FIRST DOSE OF INTRAMUSCULAR ANTIBIOTICS

- Give first dose of both ampicillin <u>and</u> gentamicin intramuscularly.

WEIGHT	**AMPICILLIN**<br>Dose: 50 mg per kg<br>To a vial of 250 mg	**GENTAMICIN**	
	Add 1.3 ml sterile water = 250 mg/1.5ml	Undiluted 2 ml vial containing 20 mg = 2 ml at 10 mg/ml OR Add 6 ml sterile water to 2 ml vial containing 80 mg* = 8 ml at 10 mg/ml	
		**AGE <7 days**<br>Dose: 5 mg per kg	**AGE >= 7 days**<br>Dose: 7.5 mg per kg
1-<1.5 kg	0.4 ml	0.6 ml*	0.9 ml*
1.5-<2 kg	0.5 ml	0.9 ml*	1.3 ml*
2-<2.5 kg	0.7 ml	1.1 ml*	1.7 ml*
2.5-<3 kg	0.8 ml	1.4 ml*	2.0 ml*
3-<3.5 kg	1.0 ml	1.6 ml*	2.4 ml*
3.5-<4 kg	1.1 ml	1.9 ml*	2.8 ml*
4-<4.5 kg	1.3 ml	2.1 ml*	3.2 ml*

* *Avoid using undiluted 40 mg/ml gentamicin.*

- Referral is the best option for a young infant classified with VERY SEVERE DISEASE. If referral is not possible, continue to give ampicillin <u>and</u> gentamicin for at least 5 days. Give ampicillin two times daily to infants less than one week of age and 3 times daily to infants one week or older. Give gentamicin once daily.

TREAT THE YOUNG INFANT TO PREVENT LOW BLOOD SUGAR

- ***If the young infant is able to breastfeed:***
  Ask the mother to breastfeed the young infant.
- ***If the young infant is not able to breastfeed but is able to swallow:***
  Give 20-50 ml (10 ml/kg) expressed breast milk before departure. If not possible to give expressed breast milk, give 20-50 ml (10 ml/kg) sugar water ***(To make sugar water: Dissolve 4 level teaspoons of sugar (20 grams) in a 200-ml cup of clean water)***.
- ***If the young infant is not able to swallow:***
  Give 20-50 ml (10 ml/kg) of expressed breast milk or sugar water by nasogastric tube.

TREAT THE YOUNG INFANT

TEACH THE MOTHER HOW TO KEEP THE YOUNG INFANT WARM ON THE WAY TO THE HOSPITAL

- Provide skin to skin contact

  OR
- Keep the young infant clothed or covered as much as possible all the time. Dress the young infant with extra clothing including hat, gloves, socks and wrap the infant in a soft dry cloth and cover with a blanket.

GIVE AN APPROPRIATE ORAL ANTIBIOTIC FOR LOCAL BACTERIAL INFECTION

- First-line antibiotic: ____
- Second-line antibiotic:____

AGE or WEIGHT	**AMOXICILLIN** Give 2 times daily for 5 days	
	Tablet 250 mg	Syrup 125 mg in 5 ml
Birth up to 1 month (<4 kg)	1/4	2.5 ml
1 month up to 2 months (4-<6 kg)	1/2	5 ml

.

TEACH THE MOTHER TO TREAT LOCAL INFECTIONS AT HOME

- Explain how the treatment is given.
- Watch her as she does the first treatment in the clinic.
- Tell her to return to the clinic if the infection worsens.

To Treat Skin Pustules or Umbilical Infection	To Treat Thrush (ulcers or white patches in mouth)
The mother should do the treatment twice daily for 5 days:<br>• Wash hands<br>• Gently wash off pus and crusts with soap and water<br>• Dry the area<br>• Paint the skin or umbilicus/cord with full strength gentian violet (0.5%)<br>• Wash hands	The mother should do the treatment four times daily for 7 days:<br>• Wash hands<br>• Paint the mouth with half-strength gentian violet (0.25%) using a soft cloth wrapped around the finger<br>• Wash hands

To Treat Diarrhoea, See TREAT THE CHILD Chart.

TREAT THE YOUNG INFANT

Immunize Every Sick Young Infant, as Needed

GIVE ARV FOR PMTCT PROPHYLAXIS

Initiate triple ART for all pregnant and lactating women with HIV infection, and put their infants on ART prophylaxis*:

Nevirapine or zidovudine are provided to young infant classified as HIV EXPOSED to minimize the risk of mother-to-child HIV transmission (PMTCT).

- ***If breast feeding:*** Give NVP for 6 weeks beginning at birth or when HIV exposure is recognized.
- ***If not breast feeding:*** Give NVP or ZDV for 4-6 weeks beginning at birth or when HIV exposure is recognized.

AGE	**NEVIRAPINE** Give once daily.	**ZIDOVUDINE (AZT)** Give once daily
**Birth up to 6 weeks:**		
▪ Birth weight 2000 - 2499 g	10 mg	10 mg
▪ Birth weight > 2500 g	15 mg	15 mg
**Over 6 weeks:**	20 mg	-

*** PREVENTION OF MATERNAL-TO-CHILD-TRANSMISSION (PMTCT) ART PROPHYLAXIS:**

OPTION B+: MOTHER ON LIFELONG TRIPLE ART REGIMEN, YOUNG INFANT ON NVP PROPHYLAXIS FROM BIRTH FOR 6 WEEKS IF BREASTFEEDING OR NVP OR AZT FOR 4-6 WEEKS IF ON REPLACEMENT FEEDING.

OPTION B: MOTHER ON TRIPLE ART REGIMEN TO BE DISCONTINUED ONE WEEK AFTER CESSATION OF BREASTFEEDING, YOUNG INFANT ON NVP PROPHYLAXIS FROM BIRTH FOR 6 WEEKS OR NVP OR AZT FOR 4-6 WEEKS IF ON REPLACEMENT FEEDING.

COUNSEL THE MOTHER

TEACH CORRECT POSITIONING AND ATTACHMENT FOR BREASTFEEDING

- Show the mother how to hold her infant.
  - with the infant's head and body in line.
  - with the infant approaching breast with nose opposite to the nipple.
  - with the infant held close to the mother's body.
  - with the infant's whole body supported, not just neck and shoulders.

- Show her how to help the infant to attach. She should:
  - touch her infant's lips with her nipple
  - wait until her infant's mouth is opening wide
  - move her infant quickly onto her breast, aiming the infant's lower lip well below the nipple.

- Look for signs of good attachment and effective suckling. If the attachment or suckling is not good, try again.

TEACH THE MOTHER HOW TO EXPRESS BREAST MILK

Ask the mother to:

- Wash her hands thoroughly.
- Make herself comfortable.
- Hold a wide necked container under her nipple and areola.
- Place her thumb on top of the breast and the first finger on the under side of the breast so they are opposite each other (at least 4 cm from the tip of the nipple).
- Compress and release the breast tissue between her finger and thumb a few times.
- If the milk does not appear she should re-position her thumb and finger closer to the nipple and compress and release the breast as before.
- Compress and release all the way around the breast, keeping her fingers the same distance from the nipple. Be careful not to squeeze the nipple or to rub the skin or move her thumb or finger on the skin.
- Express one breast until the milk just drips, then express the other breast until the milk just drips.
- Alternate between breasts 5 or 6 times, for at least 20 to 30 minutes.
- Stop expressing when the milk no longer flows but drips from the start.

TEACH THE MOTHER HOW TO FEED BY A CUP

- Put a cloth on the infant's front to protect his clothes as some milk can spill.
- Hold the infant semi-upright on the lap.
- Put a measured amount of milk in the cup.
- Hold the cup so that it rests lightly on the infant's lower lip.
- Tip the cup so that the milk just reaches the infant's lips.
- Allow the infant to take the milk himself. DO NOT pour the milk into the infant's mouth.

TEACH THE MOTHER HOW TO KEEP THE LOW WEIGHT INFANT WARM AT HOME

- Keep the young infant in the same bed with the mother.
- Keep the room warm (at least 25°C) with home heating device and make sure that there is no draught of cold air.
- Avoid bathing the low weight infant. When washing or bathing, do it in a very warm room with warm water, dry immediately and thoroughly after bathing and clothe the young infant immediately.
- Change clothes (e.g. nappies) whenever they are wet.
- Provide skin to skin contact as much as possible, day and night. For skin to skin contact:
  - Dress the infant in a warm shirt open at the front, a nappy, hat and socks.
  - Place the infant in skin to skin contact on the mother's chest between her breasts. Keep the infat's head turned to one side.
  - Cover the infant with mother's clothes (and an additional warm blanket in cold weather).
- When not in skin to skin contact, keep the young infant clothed or covered as much as possible at all times. Dress the young infant with extra clothing including hat and socks, loosely wrap the young infant in a soft dry cloth and cover with a blanket.
- Check frequently if the hands and feet are warm. If cold, re-warm the baby using skin to skin contact.
- Breastfeed the infant frequently (or give expressed breast milk by cup).

COUNSEL THE MOTHER

ADVISE THE MOTHER TO GIVE HOME CARE FOR THE YOUNG INFANT

1. **EXCLUSIVELY BREASTFEED THE YOUNG INFANT**
   Give only breastfeeds to the young infant. Breastfeed frequently, as often and for as long as the infant wants.
2. **MAKE SURE THAT THE YOUNG INFANT IS KEPT WARM AT ALL TIMES.**
   In cool weather cover the infant's head and feet and dress the infant with extra clothing.
3. **WHEN TO RETURN:**

**Follow up visit**	
**If the infant has:**	**Return for first follow-up in:**
▪ JAUNDICE	1 day
▪ LOCAL BACTERIAL INFECTION<br>▪ FEEDING PROBLEM<br>▪ THRUSH<br>▪ DIARRHOEA	2 days
▪ LOW WEIGHT FOR AGE	14 days
▪ CONFIRMED HIV INFECTION<br>▪ HIV EXPOSED	According to national recommendations

WHEN TO RETURN IMMEDIATELY:

**Advise the mother to return immediately if the young infant has any of these signs:**
▪ Breastfeeding poorly<br>▪ Reduced activity<br>▪ Becomes sicker<br>▪ Develops a fever<br>▪ Feels unusually cold<br>▪ Fast breathing<br>▪ Difficult breathing<br>▪ Palms and soles appear yellow

FOLLOW-UP

GIVE FOLLOW-UP CARE FOR THE YOUNG INFANT

ASSESS EVERY YOUNG INFANT FOR "VERY SEVERE DISEASE" DURING FOLLOW-UP VISIT

LOCAL BACTERIAL INFECTION

After 2 days:

- Look at the umbilicus. Is it red or draining pus?
- Look at the skin pustules.

Treatment:

- If umbilical ***pus or redness remains same or is worse***, refer to hospital. If ***pus and redness are improved***, tell the mother to continue giving the 5 days of antibiotic and continue treating the local infection at home.
- If skin pustules are ***same or worse***, refer to hospital. If ***improved***, tell the mother to continue giving the 5 days of antibiotic and continue treating the local infection at home.

DIARRHOEA

After 2 days:

Ask: Has the diarrhoea stopped?

Treatment

- If the diarrhoea has not stopped, assess and treat the young infant for diarrhoea. >SEE "Does the Young Infant Have Diarrhoea?"
- If the diarrhoea has stopped, tell the mother to continue exclusive breastfeeding.

GIVE FOLLOW-UP CARE FOR THE YOUNG INFANT

JAUNDICE

After 1 day:

- Look for jaundice. Are palms and soles yellow?

Treatment:

- If palms and soles are yellow, refer to hospital.
- If palms and soles are not yellow, but jaundice has not decreased, advise the mother home care and ask her to return for follow up in 1 day.
- If jaundice has started decreasing, reassure the mother and ask her to continue home care. Ask her to return for follow up at 2 weeks of age. If jaundice continues beyond two weeks of age, refer the young infant to a hospital for further assessment.

FEEDING PROBLEM

After 2 days:
Reassess feeding. > See "Then Check for Feeding Problem or Low Weight".
Ask about any feeding problems found on the initial visit.

- Counsel the mother about any new or continuing feeding problems. If you counsel the mother to make significant changes in feeding, ask her to bring the young infant back again.
- If the young infant is low weight for age, ask the mother to return 14 days of this follow up visit. Continue follow-up until the infant is gaining weight well.

Exception:
If you do not think that feeding will improve, or if the young infant has ***lost weight***, refer the child.

LOW WEIGHT FOR AGE

After 14 days:

Weigh the young infant and determine if the infant is still low weight for age.

Reassess feeding. > See "Then Check for Feeding Problem or Low Weight".

- If the infant is ***no longer low weight for age***, praise the mother and encourage her to continue.
- If the infant is ***still low weight for age, but is feeding well***, praise the mother. Ask her to have her infant weighed again within 14 days or when she returns for immunization, whichever is the earlier.
- If the infant is ***still low weight for age and still has a feeding problem***, counsel the mother about the feeding problem. Ask the mother to return again in 14 days (or when she returns for immunization, if this is within 14 days). Continue to see the young infant every few weeks until the infant is feeding well and gaining weight regularly and is no longer low weight for age.

Exception:
If you do not think that feeding will improve, or if the young infant has ***lost weight***, refer to hospital.

GIVE FOLLOW-UP CARE FOR THE YOUNG INFANT

THRUSH

After 2 days:

Look for ulcers or white patches in the mouth (thrush).

Reassess feeding. > See "Then Check for Feeding Problem or Low Weight".

- If ***thrush is worse*** check that treatment is being given correctly.
- If the infant has ***problems with attachment or suckling***, refer to hospital.
- If ***thrush is the same or better***, and if the infant is ***feeding well***, continue half-stregth gentian violet for a total of 7 days.

CONFIRMED HIV INFECTION OR HIV EXPOSED

A young infant classified as CONFIRMED HIV INFECTION or HIV EXPOSED should return for follow-up visits regularly as per national guidelines.

Follow the instructions for follow-up care for child aged 2 months up to 5 years.

Annex:

Skin Problems

IDENTIFY SKIN PROBLEM

IDENTIFY SKIN PROBLEM

IF SKIN IS ITCHING

	SIGNS	CLASSIFY AS:	TREATMENT	UNIQUE FEATURES IN HIV
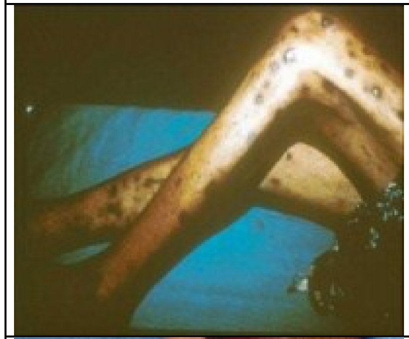	Itching rash with small papules and scratch marks. Dark spots with pale centres	**PAPULAR ITCHING RASH (PRURIGO)**	**Treat itching:**<br>■ Calamine lotion<br>■ Antihistamine oral<br>■ If not improves 1% hydrocortisone<br>Can be early sign of HIV and needs assessment for HIV	**Is a clinical stage 2 defining case**
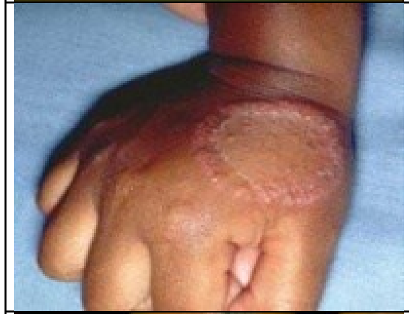	An itchy circular lesion with a raised edge and fine scaly area in the centre with loss of hair. May also be found on body or web on feet	**RING WORM (TINEA)**	Whitfield ointment or other antifungal cream if few patches<br>If extensive refer, if not give:<br>Ketoconazole<br>■ for 2 up to 12 months(6-10 kg) 40mg per day<br>■ for 12 months up to 5 years give 60 mg per day or give griseofulvin 10mg/kg/day<br>if in hair shave hair treat itching as above	Extensive: There is a high incidence of co existing nail infection which has to be treated adequately to prevent recurrence of tinea infections of skin.<br>**Fungal nail infection is a clinical stage 2 defining disease**
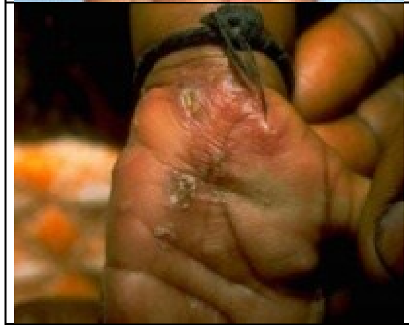	Rash and excoriations on torso; burrows in web space and wrists. face spared	**SCABIES**	Treat itching as above manage with anti scabies: 25% topical Benzyl Benzoate at night, repeat for 3 days after washing and or 1% lindane cream or lotion once wash off after 12 hours	In HIV positive individuals scabies may manifest as crust scabies.<br>Crusted scabies presents as extensive areas of crusting mainly on the scalp, face back and feet. Patients may not complain of itching. The scales will teeming with mites

IDENTIFY SKIN PROBLEM

IF SKIN HAS BLISTERS/SORES/PUSTULES

	SIGNS	CLASSIFY AS:	TREATMENT	UNIQUE FEATURES IN HIV
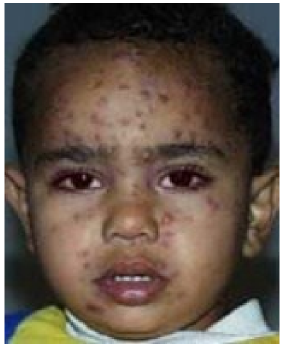	Vesicles over body. Vesicles appear progressively over days and form scabs after they rupture	**CHIKEN POX**	Treat itching as above<br>Refer URGENTLY if pneumonia or jaundice appear	Presentation atypical only if child is immunocompromised<br>Duration of disease longer<br>Complications more frequent<br>Chronic infection with continued appearance of new lesions for >1 month; typical vesicles evolve into nonhealing ulcers that become necrotic, crusted, and hyperkeratotic.
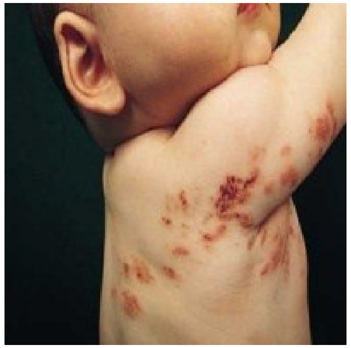	Vesicles in one area on one side of body with intense pain or scars plus shooting pain. Herpes zoster is uncommon in children except where they are immuno-compromised, for example if infected with HIV	**HERPES ZOSTER**	▪ Keep lesions clean and dry. Use local antiseptic<br>▪ If eye involved give acyclovir 20 mg /kg 4 times daily for 5 days<br>▪ Give pain relief<br>▪ Follow-up in 7 days	Duration of disease longer<br>Haemorrhagic vesicles, necrotic ulceration<br>Rarely recurrent, disseminated or multi-dermatomal<br>**Is a Clinical stage 2 defining disease**
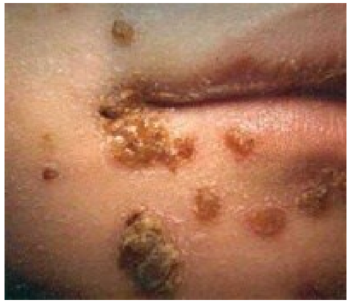	Red, tender, warm crusts or small lesions	**IMPETIGO OR FOLLICULITIS**	Clean sores with antiseptic<br>Drain pus if fluctuant<br>Start cloxacillin if size >4cm or red streaks or tender nodes or multiple abscesses for 5 days (25-50 mg/kg every 6 hours)<br>Refer URGENTLY if child has fever and /<br>or if infection extends to the muscle.	

IDENTIFY SKIN PROBLEM

NON-ITCHY

	SIGNS	CLASSIFY AS:	TREATMENT	UNIQUE FEATURES IN HIV
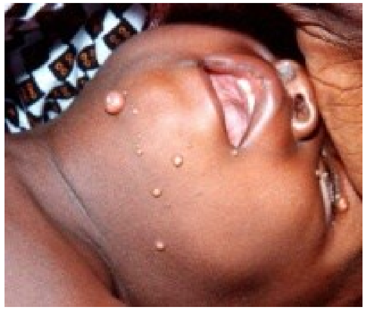	Skin coloured pearly white papules with a central umblication. It is most commonly seen on the face and trunk in children.	**MOLLUSCUM CONTAGIOSUM**	Can be treated by various modalities:<br>Leave them alone unless superinfected<br>Use of phenol: Pricking each lesion with a needle or sharpened orange stick and dabbing the lesion with phenol<br>Electrodesiccation<br>Liquid nitrogen application (using orange stick)<br>Curettage	Incidence is higher<br>Giant molluscum (>1cm in size), or coalescent<br>Pouble or triple lesions may be seen<br>More than 100 lesions may be seen.<br>Lesions often chronic and difficult to eradicate<br>Extensive molluscum contagiosum is a Clinical stage 2 defining disease
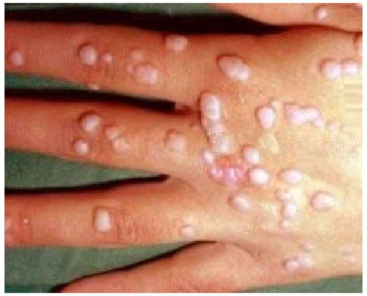	The common wart appears as papules or nodules with a rough (verrucous) surface	**WARTS**	Treatment:<br>Topical salicylic acid preparations (eg. Duofilm)<br>Liquid nitrogen cryotherapy.<br>Electrocautery	Lesions more numerous and recalcitrant to therapy<br>Extensive viral warts is a Clinical stage 2 defining disease
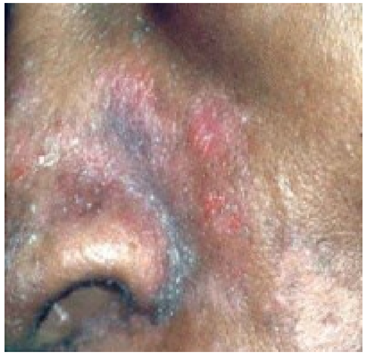	Greasy scales and redness on central face, body folds	**SEBBHORREA**	Ketoconazole shampoo<br>If severe, refer or provide tropical steroids<br>For seborrheic dermatitis: 1% hydrocortisone cream X 2 daily<br>If severe, refer	Seborrheic dermatitis may be severe in HIV infection.<br>Secondary infection may be common

CLINICAL REACTION TO DRUGS

DRUG AND ALLERGIC REACTIONS

	SIGNS	CLASSIFY AS:	TREATMENT	UNIQUE FEATURES IN HIV
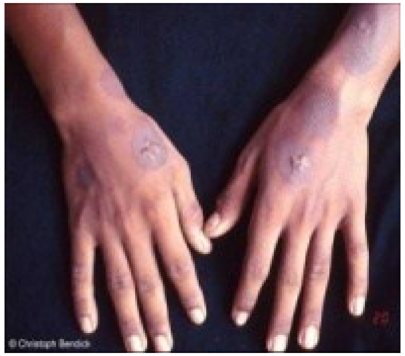	Generalized red, wide spread with small bumps or blisters; or one or more dark skin areas (fixed drug reactions)	**FIXED DRUG REACTIONS**	Stop medications give oral antihistamines, if pealing rash refer	Could be a sign of reactions to ARVs
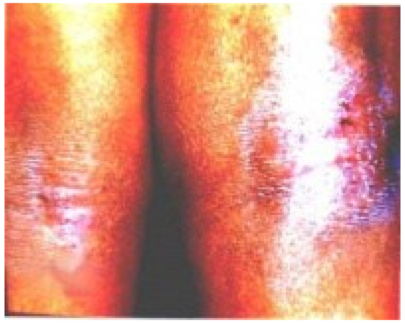	Wet, oozing sores or excoriated, thick patches	**ECZEMA**	Soak sores with clean water to remove crusts(no soap)<br>Dry skin gently<br>Short time use of topical steroid cream not on face.<br>Treat itching	
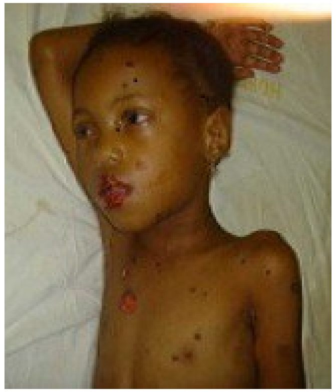	Severe reaction due to cotrimoxazole or NVP involving the skin as well as the eyes and the mouth. Might cause difficulty in breathing	**STEVEN JOHNSON SYNDROME**	Stop medication refer urgently	**The most lethal reaction to NVP, Cotrimoxazole or even Efavirens**

MANAGEMENT OF THE SICK CHILD AGED 2 MONTHS UP TO 5 YEARS

Name: Age: Weight (kg): Height/Length (cm): Temperature (°C):

Ask: What are the child's problems? Initial Visit? Follow-up Visit?

ASSESS (Circle all signs present) — **CLASSIFY**

CHECK FOR GENERAL DANGER SIGN

- NOT ABLE TO DRINK OR BREASTFEED
- VOMITS EVERYTHING
- CONVULSIONS
- LETHARGIC OR UNCONSCIOUS
- CONVULSING NOW

Classify: General danger sign present? Yes ___ No ___

Remember to use Danger sign when selecting classifications

DOES THE CHILD HAVE COUGH OR DIFFICULT BREATHING?

Classify: Yes __ No __

- For how long? ___ Days
- Count the breaths in one minute: ___ breaths per minute. Fast breathing?
- Look for chest indrawing
- Look and listen for stridor
- Look and listen for wheezing

DOES THE CHILD HAVE DIARRHOEA?

Classify: Yes __ No __

- For how long? ___ Days
- Is there blood in the stool?
- Look at the childs general condition. Is the child:
  - Lethargic or unconscious? Restless and irritable?
- Look for sunken eyes.
- Offer the child fluid. Is the child:
  - Not able to drink or drinking poorly? Drinking eagerly, thirsty?
- Pinch the skin of the abdomen. Does it go back:
  - Very slowly (longer then 2 seconds)? Slowly?

DOES THE CHILD HAVE FEVER? (by history/feels hot/temperature 37.5°C or above)

Classify: Yes __ No __

Decide malaria risk: High ___ Low ___ No___

- For how long? ___ Days
- If more than 7 days, has fever been present every day?
- Has child had measles within the last 3 months?

Do a malaria test, if NO general danger sign in all cases in high malaria risk or NO obvious cause of fever in low malaria risk:

Test POSITIVE? P. falciparum P. vivax *NEGATIVE?*

- Look or feel for stiff neck
- Look for runny nose
- Look for signs of MEASLES:
  - Generalized rash and
  - One of these: cough, runny nose, or red eyes
- Look for any other cause of fever.

If the child has measles now or within the last 3 months:

- Look for mouth ulcers. If yes, are they deep and extensive?
- Look for pus draining from the eye.
- Look for clouding of the cornea.

DOES THE CHILD HAVE AN EAR PROBLEM?

Classify: Yes __ No __

- Is there ear pain?
- Is there ear discharge? If Yes, for how long? ___ Days
- Look for pus draining from the ear
- Feel for tender swelling behind the ear

THEN CHECK FOR ACUTE MALNUTRITION AND ANAEMIA

- Look for oedema of both feet.
- Determine WFH/L z-score:
  - Less than -3? Between -3 and -2? -2 or more ?
- Child 6 months or older measure MUAC ____ mm.
- Look for palmar pallor.
  - Severe palmar pallor? Some palmar pallor?

If child has MUAC less than 115 mm or WFH/L less than -3 Z scores:

- Is there any medical complication: General danger sign?
  Any severe classification? Pneumonia with chest indrawing?
- Child 6 months or older: Offer RUTF to eat. Is the child:
  - Not able to finish? Able to finish?
- Child less than 6 months: Is there a breastfeeding problem?

CHECK FOR HIV INFECTION

- Note mother's and/or child's HIV status
  - Mother's HIV test: NEGATIVE POSITIVE NOT DONE/KNOWN
  - Child's virological test: NEGATIVE POSITIVE NOT DONE
  - Child's serological test: NEGATIVE POSITIVE NOT DONE
- If mother is HIV-positive and NO positive virological test in child:
  - Is the child breastfeeding now?
  - Was the child breastfeeding at the time of test or 6 weeks before it?
  - If breastfeeding: Is the mother and child on ARV prophylaxis?

CHECK THE CHILD'S IMMUNIZATION STATUS (Circle immunizations needed today)

BCG	DPT+HIB-1	DPT+HIB-2	DPT+HIB-3	Measles1	Measles 2	Vitamin A
OPV-0	OPV-1	OPV-2	OPV-3			Mebendazole
Hep B0	Hep B1	Hep B2	Hep B3			
	RTV-1	RTV-2	RTV-3			
	PCV-1	PCV-2	PCV-3			

Classify: Return for next immunization on: ________________ (Date)

ASSESS FEEDING if the child is less than 2 years old, has MODERATE ACUTE MALNUTRITION, ANAEMIA, or is HIV exposed or infected

Classify: FEEDING PROBLEMS

- Do you breastfeed your child? Yes ___ No ___
  - If yes, how many times in 24 hours? ___ times. Do you breastfeed during the night? Yes ___ No ___
- Does the child take any other foods or fluids? Yes ___ No ___
  - If Yes, what food or fluids?
  - How many times per day? ___ times. What do you use to feed the child?
  - If MODERATE ACUTE MALNUTRITION: How large are servings?
  - Does the child receive his own serving? ___ Who feeds the child and how?
- During this illness, has the child's feeding changed? Yes ___ No ___
  - If Yes, how?

ASSESS OTHER PROBLEMS:

Ask about mother's own health

TREAT

Remember to refer any child who has a danger sign and no other severe classification

Return for follow-up in ... days. Advise mother when to return immediately. Give any immunization and feeding advice needed today.

ART INITIATION RECORDING FORM

FOLLOW THESE STEPS TO INITIATE ART IF CHILD DOES NOT NEED URGENT REFERRAL

Name: Age: Weight (kg): Temperature (°C): Date:

ASSESS (Circle all findings)	TREAT	
**STEP 1: CONFIRM HIV INFECTION**<br>• Child under 18 months: Virological test positive<br>*Check that child has not breastfed for at least 6 weeks*<br>• Child 18 months and over: Serological test positive<br>Second serological test positive<br>*Check that child has not breastfed for at least 6 weeks*	• Send tests that are required<br>• Send confirmation test<br>***If HIV infection confirmed, and child is in stable condition, GO TO STEP 2***	YES ____ NO ____
**STEP 2: CAREGIVER ABLE TO GIVE ART**<br>• Caregiver available and willing to give medication<br>• Caregiver has disclosed to another adult, or is part of a support group	**If yes: GO TO STEP 3.**<br>**If no: COUNSEL AND SUPPORT THE CAREGIVER.**	YES ____ NO ____
**STEP 3: DECIDE IF ART CAN BE INITIATED AT FIRST LEVEL**<br>• Weight under 3 kg<br>• Child has TB	***If any present: REFER***<br>***If none present: GO TO STEP 4***	YES ____ NO ____
**STEP 4: RECORD BASELINE INFORMATION**<br>• Weight: _____ kg<br>• Height/length _____ cm<br>• Feeding problem<br>• WHO clinical stage today: _____<br>• CD4 count: _____ cells/mm3 CD4%: _____<br>• VL (if available): _____<br>• Hb: _____ g/dl	• Send tests that are required and ***GO TO STEP 5***	
**STEP 5: START ART AND COTRIMOXAZOLE PROPHYLAXIS**<br>• Less than 3 years: initiate ABC +3TC+LPV/r, or other recommended first-line regimen<br>• 3 years and older: initiate ABC+3TC+ EFV, or other recommended first-line	**RECORD ARVS & DOSAGES HERE:**<br>1. ____________________<br>2. ____________________<br>3. ____________________	
**PROVIDE FOLLOW-UP CARE**	• Follow-up according to national guidelines	NEXT FOLLOW-UP DATE: _______

RECORD ACTIONS AND TREATMENTS HERE:
ALWAYS REMEMBER TO COUNSEL THE MOTHER AND PROVIDE ROUTINE CARE

FOLLOW-UP CARE FOR CONFIRMED HIV INFECTION ON ART: SIX STEPS

Name: Age: Weight (kg): Height/legth (cm): Temperature (°C): Date:

Circle all findings

<table>
<tr><td>

STEP 1: ASSESS AND CLASSIFY

ASK: does the child have any problems?

ASK: has the child received care at another health facility since the last visit?

- **Check for general danger signs:**
  - NOT ABLE TO DRINK OR BREASTFEED
  - VOMITS EVERYTHING
  - CONVULSIONS
  - LETHARGIC OR UNCONSCIOUS
  - CONVULSING NOW
- **Check for ART severe side effects:**
  - Severe skin rash
  - Yellow eyes
  - Difficulty breathing and severe abdominal pain
  - Fever, vomiting, rash (only if on Abacavir)
- **Check for main symptoms:**
  - Cough or difficulty breathing
  - Diarrhoea
  - Fever
  - Ear problem
  - Other problems

</td><td>

If yes, record here: ____________________

YES ____ NO ____

If general danger signs or ART severe side effects, provide pre-referral treatment and REFER URGENTLY

Assess, classify, treat, and follow-up main symptoms according to IMCI guidelines. Refer if necessary.

</td><td>

RECORD ACTIONS TAKEN:

</td></tr>
<tr><td>

STEP 2: MONITOR ARV TREATMENT

Assess adherence:

- Takes all doses - Frequently misses doses - Occasionally misses a dose -

Not taking medication

- **Assess side-effects**

Nausea - Tingling, numb, or painful hands, feet, or legs - Sleep disturbances -

Diarrhoea - Dizziness - Abnormal distribution of fat - Rash - Other

- **Assess clinical condition:**

Progressed to higher stage

Stage when ART initiated: 1 - 2 - 3 - 4 - Unknown

- **Monitor blood results:** Tests should be sent after 6 months on ARVs, then yearly. Record latest results here:

DATE: _____ CD4 COUNT:________cells/mm3 CD4%: __________

Viral load: _________

If on LPV/r: LDL Cholesterol: _________ TGs: ____________

</td><td>

1. REFER NON-URGENTLY IF ANY OF THE FOLLOWING ARE PRESENT:

- Not gaining weight for 3 months
- Loss of milestones
- Poor adherence despite adherence counselling
- Significant side-effects despite appropriate management
- Higher clinical stage than before
- CD4 count significantly lower than before
- LDL higher than 3.5 mmol/L
- Triglycerides (TGs) higher than 5.6 mmol/L

2. MANAGE MILD SIDE-EFFECTS

3. SEND TESTS THAT ARE DUE

- CD4 count
- Viral load, if available
- LDL cholesterol and triglycerides

OTHERWISE, GO TO STEP 3

</td><td>

RECORD ACTIONS TAKEN:

</td></tr>
<tr><td>

STEP 3: PROVIDE ART AND OTHER MEDICATION

ABC+3TC+LPV/r

ABC+3TC+EFV

Cotrimoxazole

Vitamin A

Other Medication

</td><td>

RECORD ART DOSAGES:

1. ____________________
2. ____________________
3. ____________________

- **COTRIMOXAZOLE DOSAGE**:____________________
- **VITAMIN A DOSAGE**: ____________________
- OTHER MEDICATION DOSAGE:
  1. ____________________
  2. ____________________
  3. ____________________

</td><td></td></tr>
<tr><td>

STEP 4: COUNSEL

Use every visit to educate the caregiver and provide support, key issues include:

How is child progressing - Adherence - Support to caregiver - Disclosure (to others & child) - Side-effects and correct management

</td><td>

RECORD ISSUES DISCUSSED:

</td><td>

DATE OF NEXT VISIT:

</td></tr>
</table>

RECORD ACTIONS TAKEN:

MANAGEMENT OF THE SICK YOUNG INFANT AGED UP TO 2 MONTHS

Name: Age: Weight (kg): Temperature (°C):
Ask: What are the infant's problems?: Initial Visit? Follow-up Visit?
ASSESS (Circle all signs present) **CLASSIFY**

CHECK FOR SEVERE DISEASE AND LOCAL BACTERIAL INFECTION

- Is the infant having difficulty in feeding?
- Has the infant had convulsions?

- Count the breaths in one minute. ___ breaths per minute
  Repeat if elevated: ___ Fast breathing?
- Look for severe chest indrawing.
- Look and listen for grunting.
- Look at the umbiculus. Is it red or draining pus?
- Fever (temperature 38°C or above fells hot) or
  low body temperature (below 35.5°C or feels cool)
- Look for skin pustules. Are there many or severe pustules?
- Movement only when stimulated or no movement even when stimulated?

THEN CHECK FOR JAUNDICE

- When did the jaundice appear first?

- Look for jaundice (yellow eyes or skin)
- Look at the young infant's palms and soles. Are they yellow?

DOES THE YOUNG INFANT HAVE DIARRHOEA?

Yes ___ No ___

- Look at the young infant's general condition. Does the infant:
  - move only when stimulated?
  - not move even when stimulated?

  Is the infant restless and irritable?
- Look for sunken eyes.
- Pinch the skin of the abdomen. Does it go back:
  - Very slowly?
  - Slowly?

THEN CHECK FOR FEEDING PROBLEM OR LOW WEIGHT

If the infant has no indication to refer urgently to hospital

- Is there any difficulty feeding? Yes ___ No ___
- Is the infant breastfed? Yes ___ No ___
  If yes, how many times in 24 hours? ___ times
- Does the infant usually receive any other foods or drinks? Yes ___ No ___
  If yes, how often?
- What do you use to feed the child?

- Determine weight for age. Low ___ Not low ___
- Look for ulcers or white patches in the mouth (thrush).

CHECK FOR HIV INFECTION

- Note mother's and/or child's HIV status:
  - Mother's HIV test: NEGATIVE POSITIVE NOT DONE/KNOWN
  - Child's virological test: NEGATIVE POSITIVE NOT DONE
  - Child's serological test: NEGATIVE POSITIVE NOT DONE
- If mother is HIV positive and and NO positive virological test in young infant:
  - Is the infant breastfeeding now?
  - Was the infant breastfeeding at the time of test or 6 weeks before it?
  - If breastfeeding: Is the mother and infant on ARV prophylaxis?

ASSESS BREASTFEEDING

- Has the infant breastfed in the previous hour?

If the infant has not fed in the previous hour, ask the mother to put her infant to the breast. Observe the breastfeed for 4 minutes.

- Is the infant able to attach? To check attachment, look for:
  - Chin touching breast: Yes ___ No ___
  - Mouth wide open: Yes ___ No ___
  - Lower lip turned outward: Yes ___ No ___
  - More areola above than below the mouth: Yes ___ No ___

    not well attached *good attachment*
- Is the infant sucking effectively (that is, slow deep sucks, sometimes pausing)?

  not sucking effectively *sucking effectively*

CHECK THE CHILD'S IMMUNIZATION STATUS (Circle immunizations needed today)

BCG	DPT+HIB-1	DPT+HIB-2	Hep B 1	Hep B 2	200,000 I.U vitamin A to mother
OPV-0	OPV-1	OPV-2			

Return for next immunization on:

____
(Date)

ASSESS OTHER PROBLEMS:

Ask about mother's own health

TREAT

Return for follow-up in ... days. Advise mother when to return immediately. Give any immunization and feeding advice needed today.

Weight-for-age GIRLS

World Health Organization

Birth to 6 months (z-scores)

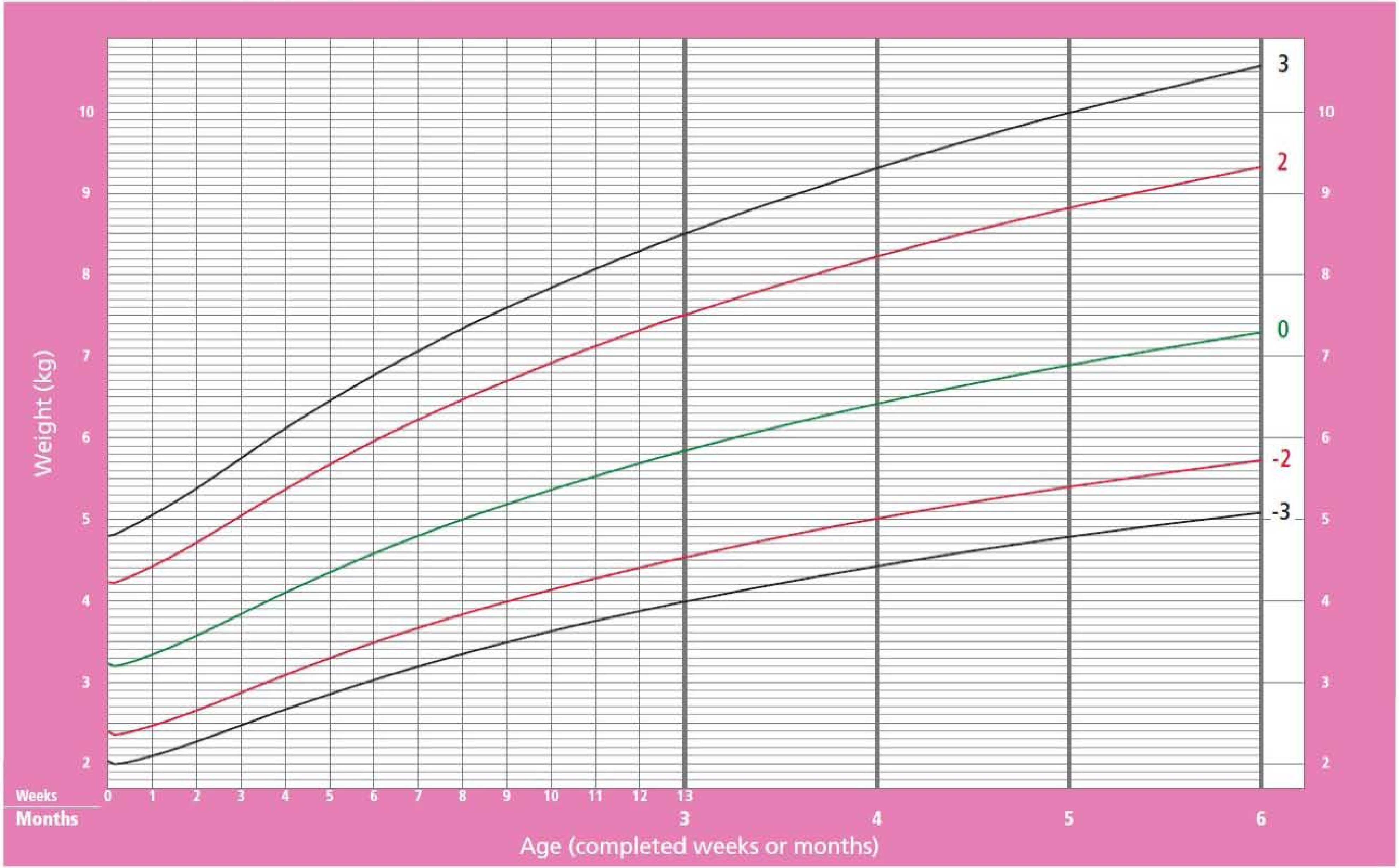

WHO Child Growth Standards

Weight-for-age BOYS

Birth to 6 months (z-scores)

World Health Organization

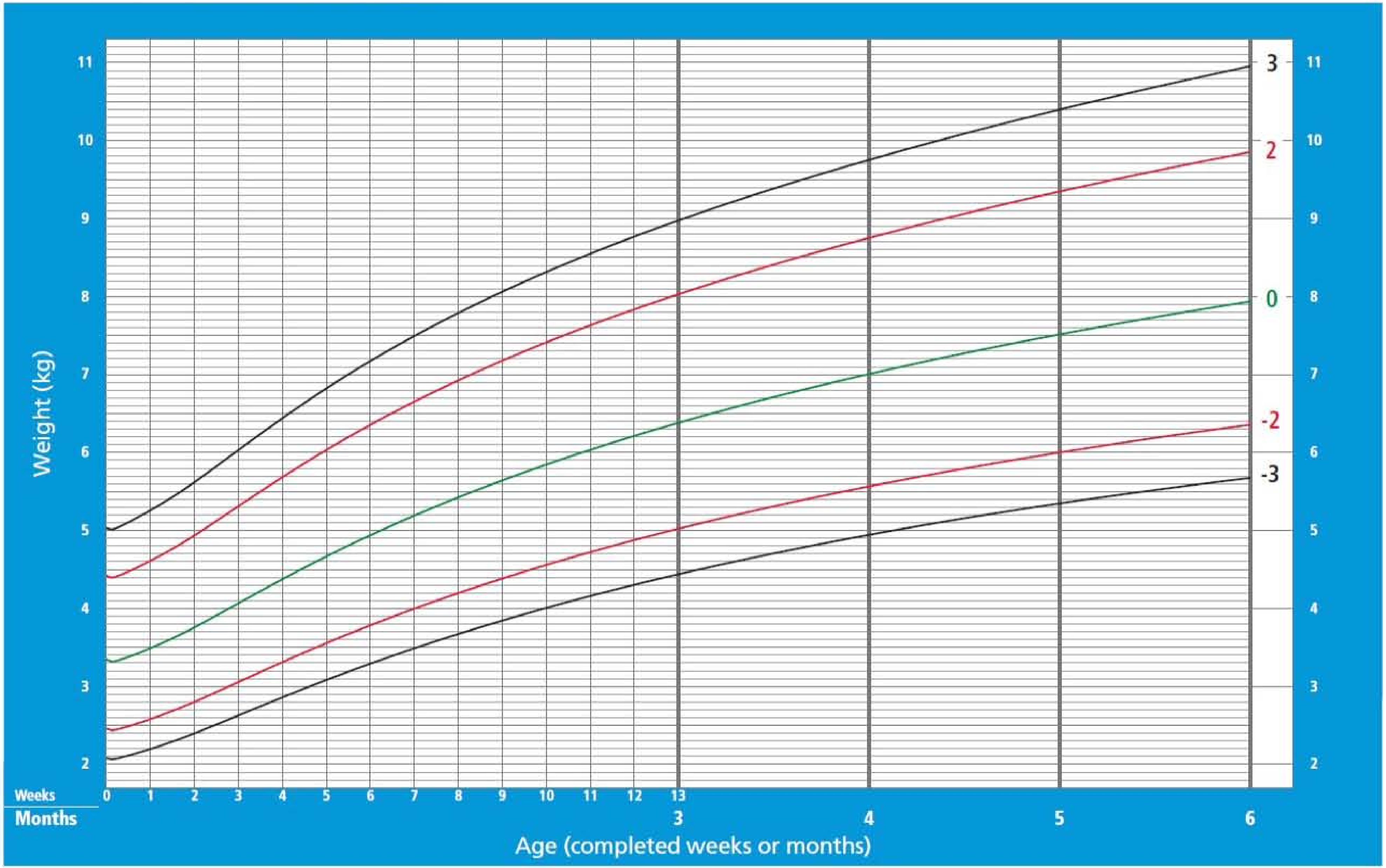

WHO Child Growth Standards

Weight-for-length GIRLS

Birth to 2 years (z-scores)

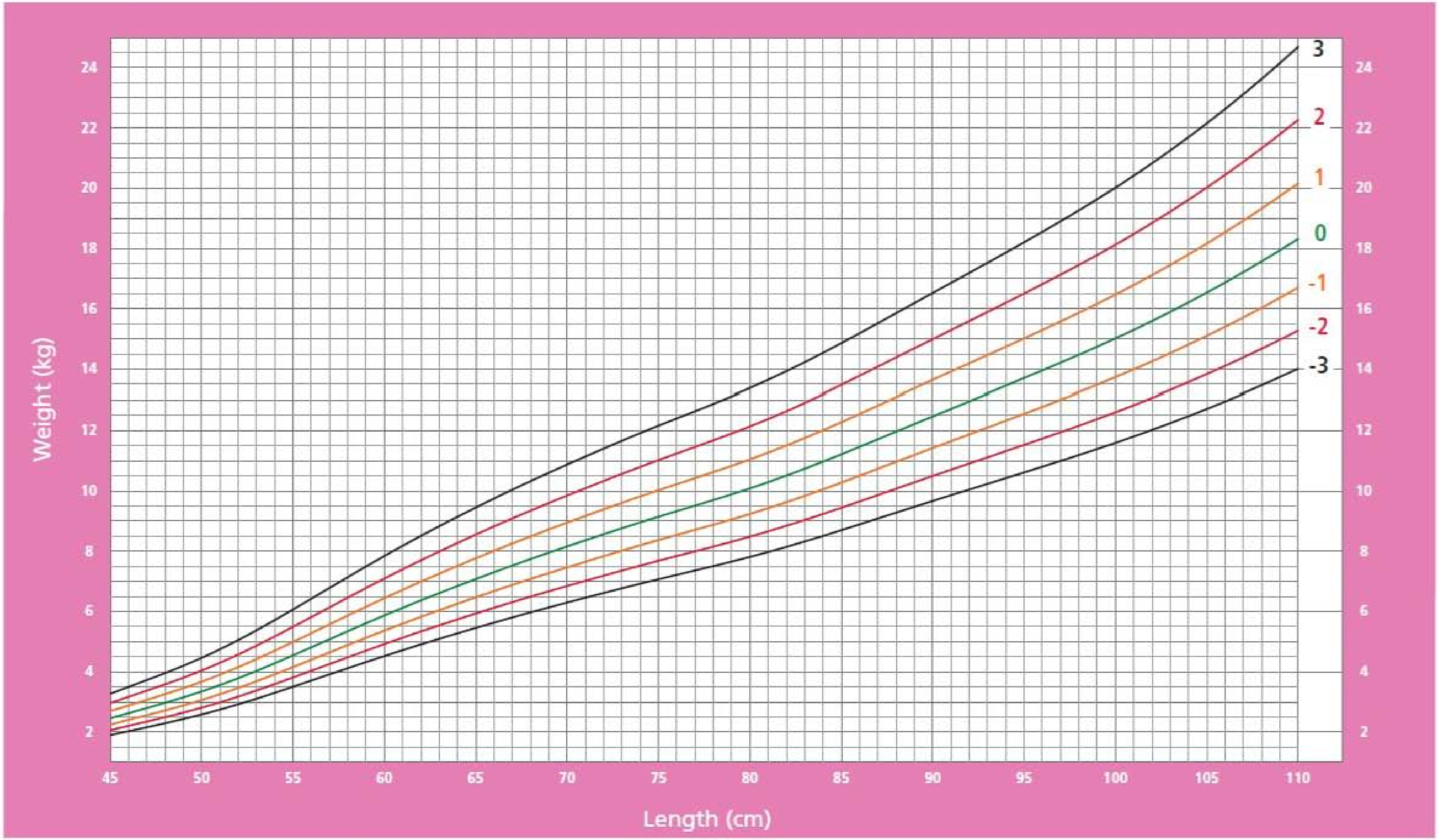

WHO Child Growth Standards

Weight-for-length BOYS

Birth to 2 years (z-scores)

World Health Organization

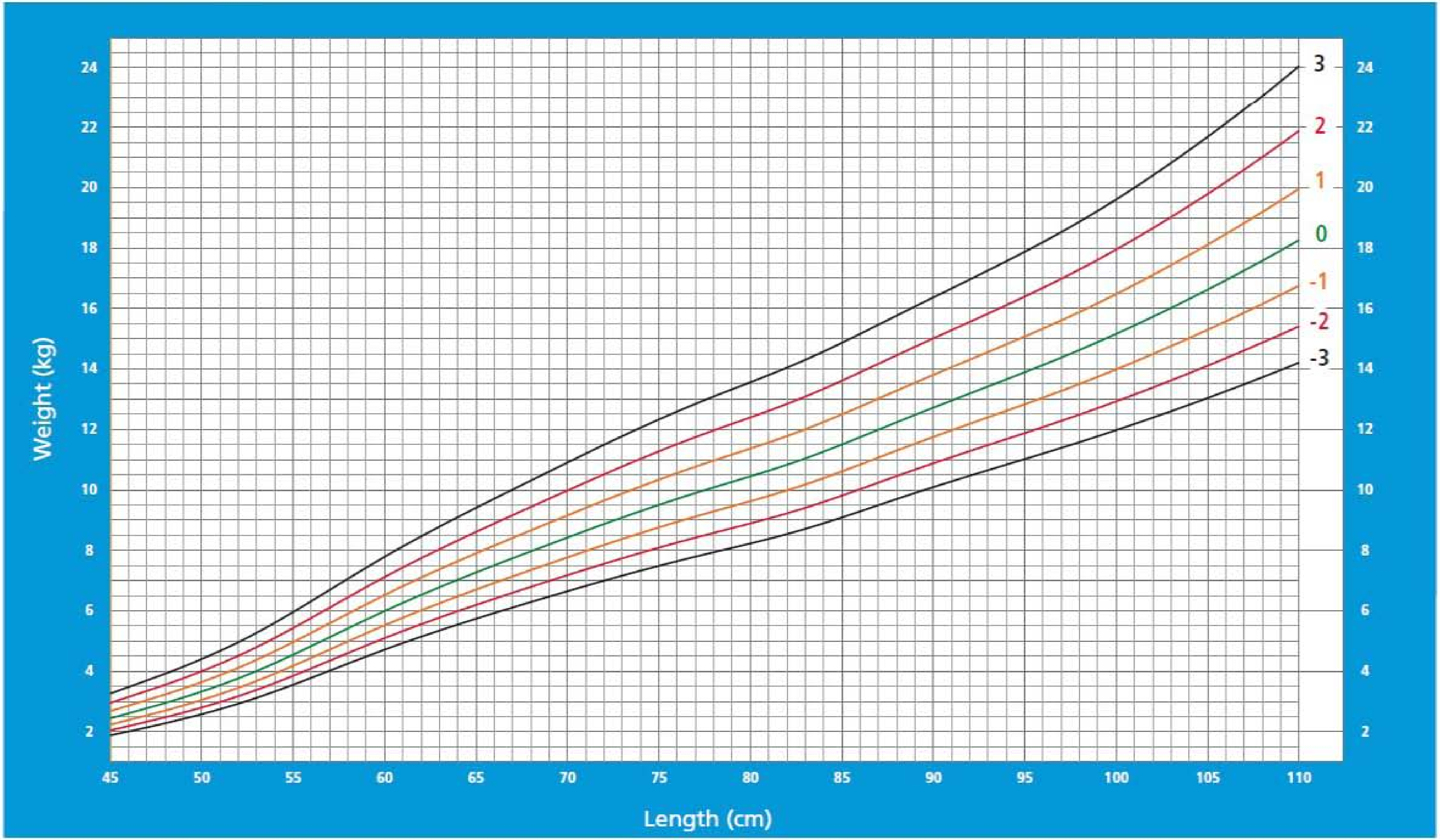

WHO Child Growth Standards

Weight-for-Height GIRLS

2 to 5 years (z-scores)

World Health Organization

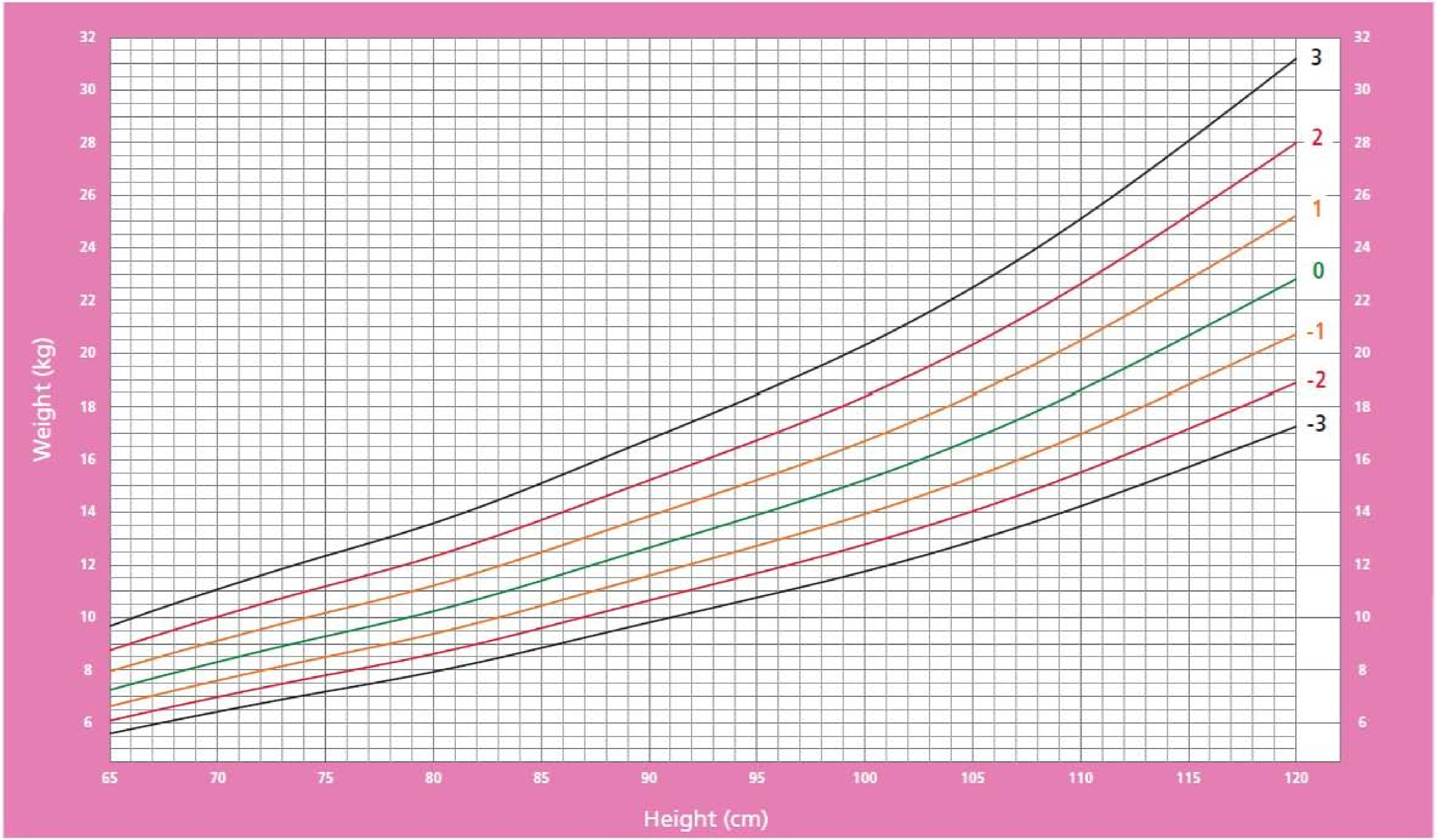

WHO Child Growth Standards

Weight-for-height BOYS

2 to 5 years (z-scores)

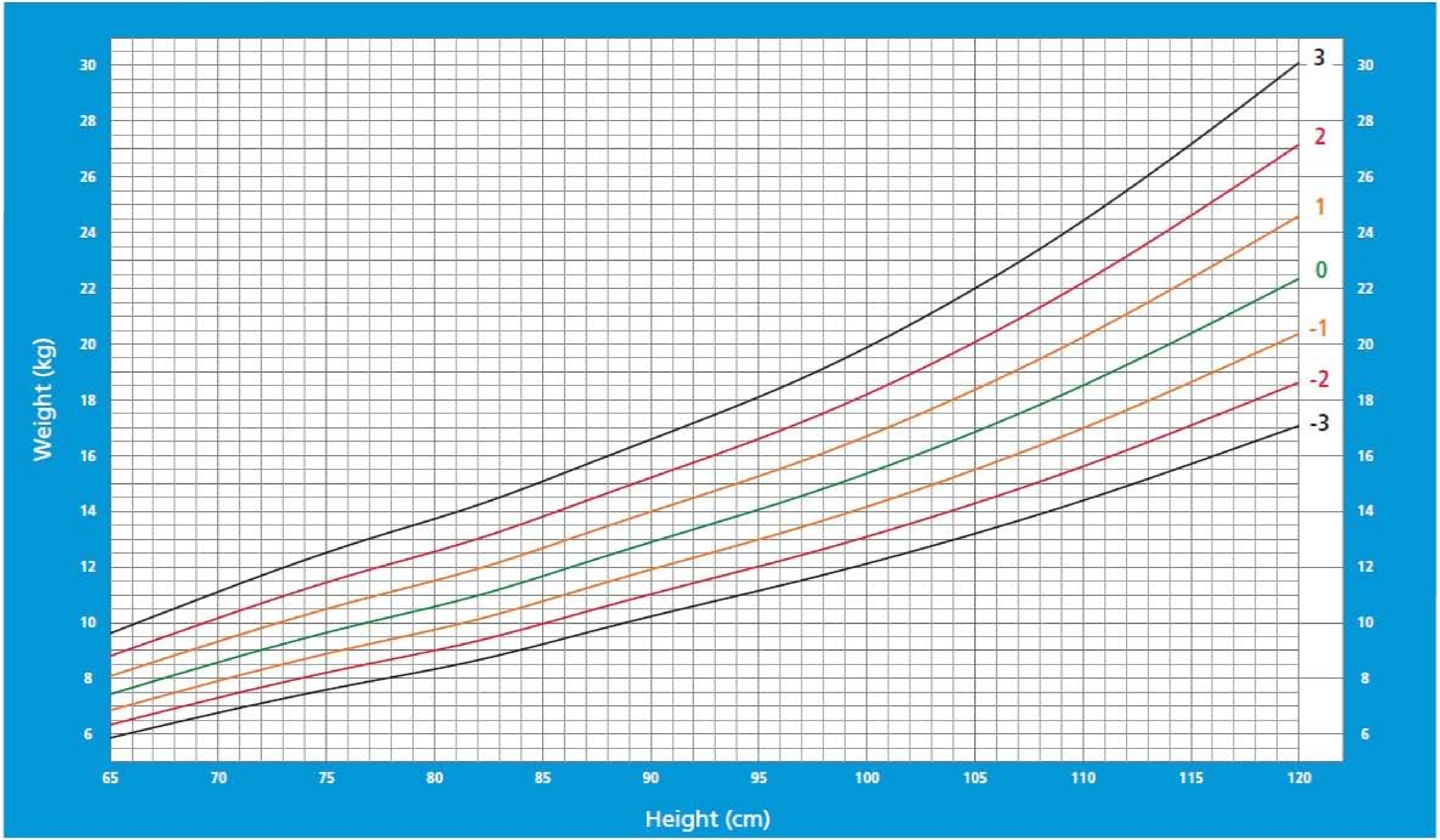

WHO Child Growth Standards

WHEN TO RETURN IMMEDIATELY

BRING ANY SICK CHILD IF

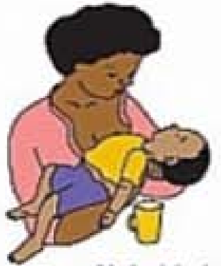

- Not able to drink or breastfeed

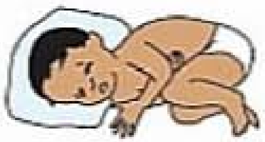

- Becomes sicker

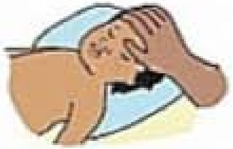

- Develops fever

BRING CHILD WITH COUGH IF

- Fast breathing

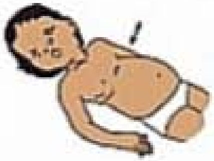

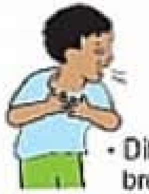

- Difficult breathing

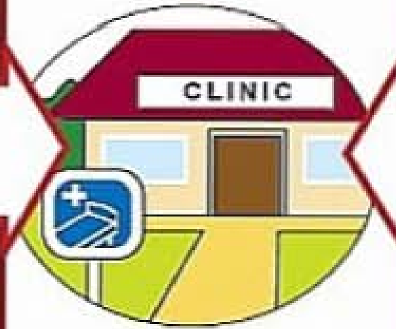

BRING CHILD WITH DIARRHOEA IF

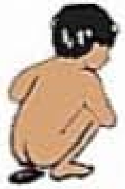

- Blood in stool
- Drinking poorly

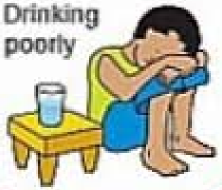

BRING YOUNG INFANT TO CLINIC IF ANY OF ABOVE SIGNS OR

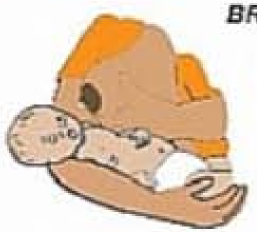

- Breastfeeding poorly

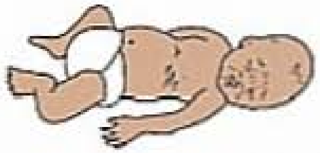

- Feels unusually cold

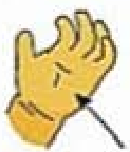

- Palms and soles appear yellow

GIVE GOOD HOME CARE FOR YOUR CHILD

FOR ANY SICK CHILD :

- If child is breastfed, breastfeed more frequently and for longer at each feed.
- If child is taking breastmilk substitutes, increase the amount of milk given
- Increase other fluids. You may give soup, rice water, yoghurt drinks or clean water.

  Give these fluids as much as the child will take. Give frequent small sips from a cup.
- If the child vomits, wait 10 minutes then continue – but more slowly

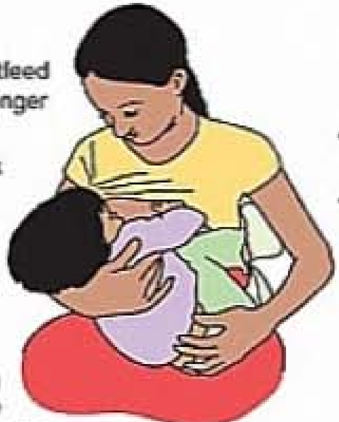

EXCLUSIVELY BREASTFEED THE YOUNG INFANT

- Give only breastfeeds to the young infant
- Breastfeed frequently, as often and for as long as the infant wants

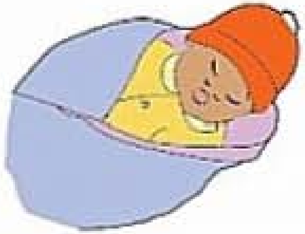

MAKE SURE THAT THE YOUNG INFANT IS KEPT WARM AT ALL TIMES

- In cool weather cover the infant's head and feet and dress the infant with extra clothing

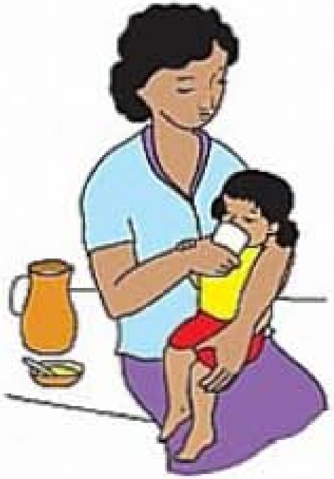

FOR CHILD WITH DIARRHOEA:

- Breastfeed frequently and for longer at each feed
- Give fluids:
  - ☐ ORS
  - ☐ Food based fluids, such as soup, rice water, yogurt drinks
  - ☐ Clean water
- Give zinc supplement, if the child aged more than 2 months and if zinc is given
- Continue giving extra fluid until the diarrhoea stops

PRINCIPLES OF THE INTEGRATED CLINICAL CASE MANAGEMENT

IMCI clinical guidelines are based on the following principles:

❶ **Examining all sick children aged up to five years** of age for **general danger signs** and all young infants for signs of **very severe disease**. These signs indicate severe illness and the need for immediate referral or admission to hospital.

❷ The children and infants are then **assessed for main symptoms:**

- ♦ In older children the main symptoms include:
  - Cough or difficulty breathing,
  - Diarrhoea,
  - Fever, and
  - Ear infection.
- ♦ In young infants, the main symptoms include:
  - Local bacterial infection,
  - Diarrhoea, and
  - Jaundice.

❸ Then in addition, all sick children are **routinely checked** for:

- Nutritional and immunization status,
- HIV status in high HIV settings, and
- Other potential problems.

❹ Only a **limited number of clinical signs** are used, selected on the basis of their sensitivity and specificity to detect disease through classification.

A combination of individual signs leads to a **child's classification** within one or more symptom groups rather than a diagnosis. The classification of illness is based on a colour-coded triage system:

- ♦ **"PINK"** indicates urgent hospital referral or admission,
- ♦ **"YELLOW"** indicates initiation of specific outpatient treatment,
- ♦ **"GREEN"** indicates supportive home care.

❺ IMCI management procedures use **a limited number of essential drugs** and encourage active participation of caregivers in the treatment of their children.

❻ An essential component of IMCI is the **counselling of caregivers** regarding home care:

- ♦ Appropriate feeding and fluids,
- ♦ When to return to the clinic immediately, and
- ♦ When to return for follow-up

IMCI Chart Booklet

This IMCI chart booklet is for use by nurses, clinicians and other health professionals who see young infants and children less than five years old. It facilitates the use of the IMCI case management process and the charts describe the sequence of all the case management steps. The chart booklet should be used by all health professionals providing care to sick children to help them apply the IMCI case management guidelines. Health professionals should always use the chart booklet for easy reference during the process of clinical care.

The chart booklet is divided into two main parts because clinical signs in sick young infants and older children are somewhat different and the case management procedures also differ between these age groups:

- **SICK CHILD AGED 2 MONTHS TO 5 YEARS.** This part contains all the necessary clinical algorithms, information and instructions on how to provide care to sick children aged 2 months to 5 years.

and

- **SICK YOUNG INFANT AGED UP TO 2 MONTHS.** This part includes case management clinical algorithms for the care of a young infant aged up to 2 months

Each of these parts contains IMCI charts corresponding to the main steps of the IMCI case management process.

For further information contact:
Maternal, Newborn, Child and Adolescent Health (MCA)
World Health Organization
20 Avenue Appia, 1211 Geneva 27, Switzerland
Tel +41-22 791 3281 • E-mail mncah@who.int
Website www.who.int/maternal_child_adolescent/en

ISBN 978 92 4 150682 3
