# Supplementary material for: Evaluating the appropriateness of laboratory testing and antimicrobial use in South African children hospitalized for community-acquired infections
Source: PLoS One. 2022 Jul 28;17(7):e0272119. doi: 10.1371/journal.pone.0272119 (PMC9333228; doi:10.1371/journal.pone.0272119)
Supplement: S2 Appendix — (PDF) [file pone.0272119.s002.pdf]

STANDARD TREATMENT GUIDELINES

AND

ESSENTIAL MEDICINES LIST

FOR

SOUTH AFRICA

HOSPITAL LEVEL
PAEDIATRICS

2013 EDITION

Copies may be obtained from:
The Directorate: Affordable Medicines
Private Bag X828
Pretoria
0001
OR
Department of Health Website: www.doh.gov.za

First printed 1998
Second edition 2006
Third edition 2013

ISBN: 978-1-920031-77-0

NOTE:
The information presented in these guidelines conforms to the current medical, nursing and pharmaceutical practice. Contributors and editors cannot be held responsible for errors, individual responses to drugs and other consequences.

© Copyright 2013, The National Department of Health.
Any part of this material may be reproduced, copied or adapted to meet local needs, without permission from the Committee or the Department of Health, provided that the parts reproduced are distributed free of charge or at no cost – **not for profit.**

Published by:
The National Department of Health, Pretoria, South Africa

FOREWORD

“*There can be no keener revelation of a society's soul than the way in which it treats its children.”*

Nelson R Mandela

Our children’s health and wellbeing are amongst our highest priorities. This should, therefore, be manifested in our actions and policies which should express care, love and respect for our future generations.

In order to achieve the Millennium Development Goal of reducing child mortality, available resources will need to be efficiently deployed towards improving the health of our children and their quality of life. The Standard Treatment Guidelines (STGs) and Essential Medicines List (EML) for Hospital Level, Paediatrics (2013), is a valuable tool to improve the quality of care that we, as healthcare professionals, strive to provide to our children.

In keeping with previous editions, the STGs and EML for Hospital Level, Paediatrics (2013), was developed and reviewed on the principles of evidence-based medicine. While we are aware of the paucity of comparative randomised evidence in paediatrics, the best available clinical evidence was used to formulate recommendations. A solid foundation for the rational selection of medicines has been laid and this should be used to allocate available resources in a reasonable manner.

We are grateful to everyone who actively participated in the peer review process and contributed by way of comment and submission of appropriate evidence. The quality of this edition was enhanced by your continued enthusiasm, participation and dedication. For this, I thank you.

Let us all work towards achieving the goal of a long and healthy life for all our citizens, and especially our children.

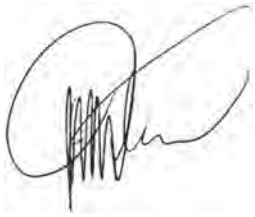

DR A MOTSOALEDI, MP
MINISTER OF HEALTH
DATE: 23/1/2014

INTRODUCTION

All of South Africa's children deserve access to care of the highest quality. To this end, I am pleased to introduce the Standard Treatment Guidelines and Essential Medicines List for Hospital Level (Paediatrics) 2013 edition.

The Standard Treatment Guidelines (STG) and Essential Medicines List (EML) for Hospital Level (Paediatrics) is a tool that, if actively embraced by clinicians, will propel us toward meeting our targets in terms of the Millennium Development Goals. It will guide us closer to our vision of quality, efficient and equitable health care provision as we strive to save and protect the lives of our babies and children.

Once again, this edition was reviewed following extensive consultation and collaboration. Besides consultation with various paediatricians in the field, there was also extensive collaboration with all the major programmes within the Department including, HIV and AIDS and Child Health Clusters, and in particular, IMCI, TB and Nutrition Directorates.

Recommendations regarding therapeutic interventions were guided by available, relevant clinical evidence. Additionally, the cost and incremental effectiveness of alternative medicines were considered, ensuring that our selections are affordable and sustainable within a publicly financed health system. In this way we can contribute to a health system that provides the greatest possible health benefits with the available resources.

In this edition, substantial changes have been made to the STG with the addition of new conditions and, in particular, the development of two new chapters relating to the care of adolescents and the management of drug allergies.

We are grateful to all experts and clinicians who enthusiastically participated in the review process. The final publication reflects the commitment of both the Expert Review Committee and all the many contributors who have the best interests of our children at heart.

It is our sincere wish that the Paediatric Hospital Level STGs and EML will be used actively to improve the quality of care provided to our children.

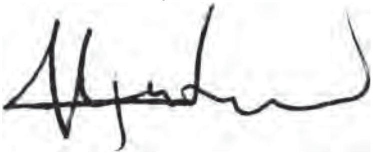

MS MP MATSOSO
DIRECTOR-GENERAL: HEALTH
DATE: 23/1/2014

ACKNOWLEDGEMENTS

Without the continued dedication of the members of the Paediatric Expert Review Committee for the Hospital Level Essential Medicines List, this edition of the Standard Treatment Guidelines and Essential Medicines List would not have been possible.

The quality of this edition was further enhanced by the contribution of many doctors, pharmacists, professional societies and other health care professionals. Although some of the submissions made may not be reflected in the STGs, we are humbled by your willingness to participate in the consultative peer review process. We hope that, with renewed enthusiasm, future editions will benefit from your contributions.

NATIONAL ESSENTIAL DRUGS LIST COMMITTEE (2009-2012)

Ms H Zeeman (Chairperson)
Dr F Benson
Prof GPG Boon
Mr V Dlamini
Prof BB Hoek
Ms Y Johnson
Prof B Maharaj
Dr T Mbengashe
Ms M Ndwandwe
Prof AG Parrish
Dr T Pillay
Mr C Shabalala (retired)
Ms N Thipa
Prof L Bamford
Prof M Blockman
Prof H Brits
Prof M Freeman
Prof PM Jeena
Prof G Maartens
Ms HM Marais
Mr HT Mphaka
Ms MNM Ntshangase
Dr L Pein
Dr H Saeed
Mr GS Steel
Prof BW van de Wal

NATIONAL ESSENTIAL MEDICINES LIST COMMITTEE (2013–2015)

Ms GS Steel (Chairperson)
Prof L Bamford
Dr E Bera
Prof M Blockman
Dr C Clark
Mrs D du Plessis
Mr A Gray
Prof B Hoek
Ms Y Johnson
Dr N Khaole
Prof G Maartens
Dr P Mntla
Dr L Mvusi
Ms M Ndwande
Dr L Pein
Dr G Reubenson
Dr W Seaketso
Prof BW van de Wal
Ms H Zeeman
Dr F Benson
Prof GPG Boon
Prof H Brits
Dr N Dlamini
Prof M Freeman
Dr G Grobler
Prof PM Jeena
Dr S Joubert
Dr J Lamprecht
Dr T Mbengashe
Mr HT Mphaka
Ms MNM Ntshangase
Prof A Parrish
Ms R Reddy
Dr C Scott
Ms N Thipa

PAEDIATRIC EXPERT COMMITTEE

Prof PM Jeena (Chairman)
Prof B Hoek (Vice Chair)
Prof L Bamford
Prof GPG Boon
Dr S Mda
Dr A Ndondo
Dr K Parbhoo
Prof HS Schaaf
Dr C Sutton
Prof I van Biljon

CONSULTANTS

Prof AM Cilliers
Prof M Cotton
Dr T de Maayer
Dr FG Goosen
Dr S Kauchali
Dr J Kriel
Prof D Morrel
Dr A Myburgh
Dr R Nassen
Dr G Reubenson
Prof H Saloojee
Dr C Scott
Prof W. Sinclair
Prof FF Takawira

COMMENTS AND CONTRIBUTIONS

Dr M Archary
Dr A Argent
Prof M Adhikari
Dr RP Benson
Dr S Bhardwaj
Ms M Bodenstein
Prof JK Burns
Dr I Couper
Dr J Cloete
Dr S Coetzee
Prof P Cooper
Prof A Cilliers
Dr E de Vries
Dr T de Maayer
Ms L Deysel
Ms SC Daniels
Prof PR Donald
Dr AL Engelbrecht
Dr P Ekermans
Prof B Eley
Dr L Fairlie
Dr U Fecht
Ms LB Foehr
Dr LN Goldstein
Dr Y Ganie
Prof RJ Green
Dr A Horn
Dr EGM Hoosen
Dr S Hawkridge
Dr T Hardcastle
Dr U Hallbauer
Dr V Henge-Daweti
Dr L Jenkins
Dr A Krug
Dr C Kelly
Dr J Kriel
Dr N Khan
Dr ME Levin
Dr P Lück
Dr CA Lee
Dr A Mahomed
Dr J Mouton
Dr R Masekela
Dr M Meiring
Dr W Marais
Dr D Moore
Ms L Moeng
Dr C Nel
Dr J Nuttal
Dr P Nourse
Ms A Ngqaka
Prof J Poole
Prof PC Potter
Dr K Pillay
Dr A Philotheou
Dr KL Petersen
Dr JC Roberts
Ms S Raiman

Prof J Rodda
Dr B Rossouw
Mr S Salie
Dr E Scriba
Dr C Tinley
Dr L Visser
Dr J Wilmshurst
Dr A Whitelaw
Prof NR Rhoda
Dr B Saunders
Dr D Segal
Ms L Terreblanche
Prof S Velaphi
Dr E von Delft
Ms J Whittal
Prof E Zöllner

EDITORIAL

Dr D Diale
Dr J Munsamy
Ms K Jamaloodien
Prof D Wittenberg (Clinical editor)

Assistance was provided by:
Mr V Jugathpal
Ms T Leong
Dr J Riddin
Dr R Lancaster
Mr P Msimanga
Dr S Singh

SECRETARIAT

Dr D Diale
Ms K Jamaloodien
Ms T Leong

LOGISTICS

Mr M Molewa
Ms P Ngobese
Mr S Sello

Mr GS Steel
Cluster Manager: Sector Wide Procurement

TABLE OF CONTENTS

Foreword			iii
Introduction			iv
Acknowledgements			v
The Essential Medicines Concept			xx
How to use this book			xxii
A guide to patient adherence in chronic diseases			xxvi
**CHAPTER 1 - EMERGENCIES AND TRAUMA**			**1.1**
1.1		Paediatric emergencies	1.1
	1.1.1	Rapid triage of the child presenting with acute conditions in casualty/out patients	1.1
	1.1.2	Approach to the resuscitation of the child	1.3
	1.1.3	Anaphylaxis/anaphylactic reactions	1.5
	1.1.4	Cardiorespiratory arrest	1.7
	1.1.5	Convulsions, not febrile convulsions	1.10
	1.1.6	Inhalation, foreign body	1.10
	1.1.7	Shock	1.11
	1.1.8	Intra-osseous infusion in emergencies	1.16
1.2		Trauma	1.17
	1.2.1	Burns	1.17
**CHAPTER 2 - ALIMENTARY TRACT**			**2.1**
2.1		Dental and oral disorders	2.1
	2.1.1	Gingivitis, uncomplicated	2.1
	2.1.2	Periodontitis	2.1
	2.1.3	Necrotising periodontitis	2.2
	2.1.4	Candidiasis, oral	2.3
	2.1.5	Aphthous ulcers	2.3
	2.1.6	Herpes gingivostomatitis	2.3
2.2		Gastrointestinal disorders	2.5
	2.2.1	Cholera	2.5
	2.2.2	Constipation/faecal loading	2.6
	2.2.3	Cystic fibrosis	2.7
	2.2.4	Diarrhoea, acute	2.9
	2.2.5	Diarrhoea, persistent	2.19

	2.2.6	Dysentery	2.23
	2.2.7	Gastro-oesophageal reflux disease (GORD)	2.24
	2.2.8	Peptic ulcer disease	2.26
	2.2.9	Inflammatory bowel disease (IBD)	2.27
2.3		Hepatic disorders	2.28
	2.3.1	Bleeding oesophageal varices	2.28
	2.3.2	Cirrhosis	2.29
		2.3.2.1 Ascites, due to hypoalbuminaemia and/or portal hypertension	2.30
	2.3.3	Portal hypertension	2.30
	2.3.4	Hepatitis, viral, acute	2.31
	2.3.5	Hepatitis, toxin induced, acute	2.32
	2.3.6	Hepatitis, chronic, autoimmune	2.33
	2.3.7	Hepatitis B, chronic	2.34
	2.3.8	Liver failure, acute	2.35
2.4		Malnutrition	2.38
	2.4.1	Malnutrition, severe acute	2.38
2.5		Rickets	2.49
2.6		Worm bolus	2.50
2.7		Recurrent abdominal pain	2.51
**CHAPTER 3 - BLOOD AND BLOOD-FORMING ORGANS**			**3.1**
3.1		Anaemia, aplastic	3.1
3.2		Anaemia, haemolytic	3.2
3.3		Anaemia, megaloblastic	3.4
3.4		Anaemia, iron deficiency	3.5
3.5		Anaemia of chronic disorders (infection or disease)	3.7
3.6		Anaemia, sickle cell	3.8
3.7		Haemophilia A and B	3.10
3.8		Von Willebrand disease	3.13
3.9		Haemorrhagic disease of the newborn	3.14
3.10		Idiopathic thrombocytopaenic purpura (ITP)	3.14
3.11		Venous thrombo-embolic disease	3.17
3.12		Special considerations in HIV infected children	3.19
	3.12.1	Thrombocytopaenia	3.19

CHAPTER 4 - CARDIOVASCULAR SYSTEM 4.1

4.1 Cardiac dysrhythmias 4.1
4.2 Cyanotic congenital heart disease with hypoxaemic attacks/spells (hypercyanotic spells) 4.6
4.2.1 Tetralogy of fallot 4.7
4.3 Endocarditis, infective 4.8
4.4 Rheumatic fever, acute 4.13
4.5 Myocarditis 4.15
4.6 Dilated cardiomyopathy 4.16
4.7 Pericardial effusion 4.17
4.8 Pericarditis 4.19
4.9 Heart failure 4.20
4.9.1 Heart failure, acute severe with pulmonary oedema and shock 4.22
4.9.2 Heart failure, acute 4.23
4.10 Dyslipidaemia 4.24
4.11 Hypertension in children 4.27
4.11.1 Hypertension, acute severe 4.37
4.11.2 Hypertension, chronic 4.38
4.12 Children with prosthetic heart valves 4.41

CHAPTER 5 - DERMATOLOGY **5.1**

5.1 Bullae 5.1
5.1.1 Epidermolysis bullosa 5.1
5.1.2 Staphylococcal scalded skin syndrome 5.1
5.1.3 Chronic bullous disease of childhood 5.2
5.2 Erythema and desquamation 5.2
5.2.1 Erythema Multiforme/Stevens-Johnson syndrome 5.2
5.3 Macules and papules 5.4
5.3.1 Drug reactions 5.4
5.3.2 Acne 5.5
5.3.3 Cellulitis and erysipelas 5.6
5.3.4 Eczema 5.7
5.3.5 Candidiasis 5.8
5.3.6 Psoriasis 5.9
5.3.7 Urticaria 5.10

5.4		Purpura	5.11
	5.4.1	Meningococcaemia	5.11
	5.4.2	Henoch-schönlein purpura	5.11
	5.4.3	Idiopathic thrombocytopenic purpura (ITP)	5.11
5.5		Vesicles and pustules	5.11
	5.5.1	Infections	5.11
	5.5.2	Skin and mucosal disorders in HIV	5.11
		5.5.2.1 HIV papular pruritic eruption	5.12
		5.5.2.2 Kaposi's sarcoma	5.12
		5.5.2.3 Warts	5.13
	5.5.3	Impetigo	5.13
**CHAPTER 6 - RENAL CONDITIONS**			**6.1**
6.1		Nephrological/urological disorders	6.1
	6.1.1	Post streptococcal glomerulonephritis	6.1
	6.1.2	Urinary tract infection (UTI)	6.4
	6.1.3	Nephrotic syndrome (NS)	6.8
	6.1.4	Acute kidney injury (renal failure, acute)	6.14
	6.1.5	Chronic kidney disease (renal failure, chronic)	6.19
	6.1.6	Enuresis	6.25
**CHAPTER 7 - ENDOCRINE SYSTEM**			**7.1**
7.1		Disorders of sexual development (DSD)	7.1
7.2		Adrenal hyperplasia, congenital	7.2
7.3		Adrenal insufficiency, acute	7.3
7.4		Diabetes insipidus	7.4
7.5		Diabetes mellitus	7.6
	7.5.1	Diabetes mellitus, insulin dependent (type 1)	7.6
		7.5.1.1 Guidelines for management of diabetics on sick days	7.17
	7.5.2	Diabetes mellitus, insulin dependent: acute complications	7.19
		7.5.2.1 Cerebral oedema in diabetic coma	7.19
		7.5.2.2 Hyperglycaemic ketoacidosis	7.20
		7.5.2.3 Hypoglycaemia in diabetics	7.26
		7.5.2.4 Nephropathy	7.28
	7.5.3	Diabetes mellitus in adolescents	7.28

	7.5.4	Diabetes mellitus, type 2	7.29
7.6		Hypoglycaemia in children	7.30
7.7		Growth disorders	7.32
7.8		Hypocalcaemia in children	7.33
7.9		Hyperkalaemia	7.34
7.10		Hypokalaemia	7.34
7.11		Hypopituitarism	7.35
7.12		Hypothyroidism, neonatal	7.36
7.13		Hypothyroidism in older children and adolescents	7.37
7.14		Hyperthyroidism, Graves disease	7.38
7.15		Obesity	7.38
7.16		Disorders of puberty	7.40
**CHAPTER 8 - INFECTIVE/INFECTIOUS DISEASES**			**8.1**
8.1		Helminthiasis, intestinal	8.1
8.2		Amoebiasis (entamoeba histolytica)	8.2
8.3		Cutaneous larva migrans/ancylostoma braziliense (dog hookworm)	8.3
8.4		Hydatid disease	8.3
8.5		Schistosomiasis (bilharzia)	8.4
8.6		Candidiasis, systemic and other	8.5
8.7		Cytomegalovirus (CMV) infection	8.7
8.8		Diphtheria	8.8
8.9		Malaria	8.10
	8.9.1	*P. falciparum* malaria, non-severe, uncomplicated	8.11
	8.9.2	*P. falciparum* malaria, severe, complicated (or if repeated vomiting)	8.12
	8.9.3	*P. ovale*, *p vivax* and *p. malariae*	8.14
	8.9.4	Malaria prophylaxis - self provided care	8.14
8.10		Measles	8.15
8.11		Meningitis, acute bacterial	8.17
8.12		Meningitis, cryptococcal	8.21
8.13		Meningitis, tuberculous (TBM)	8.22
8.14		Meningo-encephalitis/encephalitis, acute viral	8.26
8.15		Mumps	8.28
8.16		Mycobacterium avium complex (MAC) infection	8.29

8.17	Pertussis	8.30
8.18	Pneumocystis jiroveci pneumonia (PCP)	8.31
8.19	Poliomyelitis (acute flaccid paralysis)	8.31
8.20	Rabies	8.32
8.21	Tetanus	8.36
8.22	Tick bite fever	8.38
8.23	Toxoplasmosis	8.39
8.24	Typhoid	8.39
8.25	Non-typhoid salmonella (NTS)	8.40
8.26	Varicella (chickenpox)	8.42
8.27	Zoster	8.44
8.28	Sepsis (outside the neonatal period)	8.45
8.29	Staphylococcal septicaemia	8.46
**CHAPTER 9 - HUMAN IMMUNODEFICIENCY VIRUS INFECTION**		**9.1**
9.1	Human immunodeficiency virus infections	9.1
9.1.1	The HIV exposed infant	9.4
9.1.2	The HIV infected infant/child	9.8
9.2	Tuberculosis and HIV	9.21
9.3	Specific adverse events and complications	9.22
9.3.1	Lactic acidosis	9.22
9.3.2	Lipodystrophy	9.24
9.3.3	Immune reconstitution inflammatory syndrome (IRIS)	9.25
9.3.4	Wasting syndrome	9.26
9.4	Post exposure prophylaxis following alleged penetrative sexual abuse	9.26
9.5	HIV in adolescence	9.26
**CHAPTER 10 - SURGICAL PROPHYLAXIS**		**10.1**
**CHAPTER 11 - MUSCULOSKELETAL SYSTEM**		**11.1**
11.1	Arthritis, septic (pyogenic)	11.1
11.2	Arthritis, juvenile idiopathic	11.3
11.3	Osteitis/osteomyelitis, acute	11.3
**CHAPTER 12 - CONNECTIVE TISSUE DISORDERS**		12.1
12.1	Henoch Schönlein purpura (HSP)	12.1
12.2	Juvenile idiopathic arthritis (JIA)	12.2

12.3	Kawasaki syndrome/mucocutaneous lymph node syndrome	12.7
12.4	Systemic lupus erythematosus (SLE)	12.9
12.5	Takayasu arteritis	12.11
**CHAPTER 13 - THE NERVOUS SYSTEM**		13.1
13.1	Lumbar puncture	13.1
13.2	Seizures	13.2
13.3	Epilepsy	13.5
13.4	Seizures, febrile	13.10
13.5	Status epilepticus (convulsive)	13.11
13.6	Antiretroviral therapy and anticonvulsants	13.15
13.7	Headaches	13.16
13.8	Neurocysticercosis	13.18
13.9	Neuromuscular disorders	13.20
13.9.1	Inflammatory polyneuropathy (Guillain-Barré syndrome)	13.20
13.9.2	Myasthenia gravis	13.23
13.10	Sydenham's chorea	13.24
13.11	Cerebrovascular disease/stroke	13.25
**CHAPTER 14 - PAEDIATRIC PSYCHIATRY**		14.1
14.1	Sedation of acutely disturbed child or adolescent awaiting psychiatric evaluation	14.3
14.2	Attention deficit hyperactivity disorder (ADHD)	14.3
14.3	Mood disorders	14.7
14.3.1	Depression in childhood and adolescence	14.7
14.3.2	Disruptive mood dysregulation disorder (DMDD)	14.10
14.4	Anxiety disorders	14.11
14.4.1	Post traumatic stress disorder (PTSD)	14.12
14.4.2	Generalised anxiety disorder (GAD)	14.12
14.4.3	Obsessive compulsive disorder (OCD)	14.13
14.5	Childhood psychosis	14.14
14.5.1	Schizophrenia	14.15
14.5.2	Tic disorders	14.17
14.6	Other situations or contexts in which psychotropic drugs may be prescribed	14.18
14.6.1	Psychiatric presentations in HIV infected children and adolescents	14.18

14.6.2	Pervasive developmental disorders (PDDS)	14.19
14.6.3	Substance abuse	14.19
14.6.4	Behavioural problems associated with intellectual disability	14.27
**CHAPTER 15 - RESPIRATORY SYSTEM**		15.1
15.1	Chronic lung infections	15.1
15.1.1	Bronchiectasis	15.1
15.1.2	Lung abscess	15.3
15.1.3	Tuberculosis, perinatal	15.4
15.1.4	Tuberculosis, pulmonary	15.5
15.2	Conditions with predominant wheeze	15.15
15.2.1	Asthma attack, acute	15.15
15.2.2	Asthma, chronic	15.20
	15.2.2.1 Infrequent asthma	15.22
	15.2.2.2 Persistent asthma	15.23
15.2.3	Bronchiolitis	15.26
15.3	Cough with predominant fever and tachypnoea	15.28
15.3.1	Pneumonia	15.28
15.3.2	Predisposing conditions and modification of antimicrobial therapy	15.31
	15.3.2.1 Pneumonia, viral infection	15.31
	15.3.2.2 Fungal infection	15.32
	15.3.2.3 Pneumonia due to anaerobic infection	15.33
	15.3.2.4 Pneumonia, atypical due to mycoplasma or chlamydial infection	15.33
	15.3.2.5 Pneumonia, staphylococcal	15.34
	15.3.2.6 Pneumonia in HIV exposed or infected children	15.35
	15.3.2.5 Pneumonia, nosocomial	15.37
15.4	Pleural disease	15.38
15.4.1	Effusion and empyema	15.38
15.5	Upper airway diseases	15.39
15.5.1	Epiglottitis	15.39
15.5.2	Laryngotracheobronchitis, acute viral (croup)	15.41

**CHAPTER 16 - EYE CONDITIONS**		16.1
16.1	Eye infection, complicated (severe eye infection)	16.1
16.2	Conjuctivitis, allergic	16.2
16.3	Herpes keratitis and conjuctivitis	16.2
16.4	Cytomegalovirus (CMV) retinitis	16.3
16.5	Chemical burn to the eye	16.4
16.6	Penetrating eye injury with/without a foreign body	16.5
16.7	Non-penetrating eye injury	16.6
16.8	Retinopathy of prematurity (ROP)	16.7
16.9	Congenital glaucoma	16.8
16.10	Leucocoria	16.9
16.11	Strabismus	16.9
16.12	Loss of vision	16.10
**CHAPTER 17 - EAR, NOSE AND THROAT**		17.1
17.1	Abscess, retropharyngeal	17.1
17.2	Tonsilitis, complicated (peritonsillar cellulitis, peritonsillar abscess)	17.2
17.2.1	Acute bacterial tracheitis	17.3
17.3	Epistaxis (nose bleed)	17.4
17.4	Mastoiditis	17.6
17.5	Otitis externa	17.7
17.6	Otitis media, acute	17.7
17.7	Otitis media, subacute with effusion	17.8
17.8	Otitis media, chronic, suppurative	17.8
17.9	Rhinitis, allergic	17.9
17.10	Sinusitis, acute	17.10
17.11	Sinusitis, chronic	17.10
17.12	Sinusitis, complicated	17.11
17.13	Sinusitis, uncomplicated	17.12
**CHAPTER 18 - POISONING**		18.1
18.1	Poisoning	18.1
18.1.1	Anticholinergic poisoning	18.3
18.1.2	Anticoagulant poisoning	18.4
18.1.3	Antidepressant (tricyclic) poisoning	18.5

18.1.4	Caustic or corrosive agents, ingestion	18.7
18.1.5	Volatile solvents	18.8
18.1.6	Ethanol poisoning	18.8
18.1.7	Iron poisoning	18.9
18.1.8	Neuroleptic poisoning	18.11
18.1.9	Organophosphate poisoning	18.12
18.1.10	Opioid poisonong	18.13
18.1.11	Paracetamol poisoning	18.14
18.1.12	Petrochemical poisoning	18.17
18.1.13	Salicylate poisoning	18.17
18.1.14	Sedative-hypnotic poisoning	18.19
18.1.15	Sulfonylurea	18.19
18.1.16	Sympathomimetic agent poisoning	18.20
18.2	Envenomation	18.21
18.2.1	Scorpion stings	18.21
18.2.2	Snakebite	18.22
18.2.3	Spider bites (widow spiders)	18.25
	18.2.3.1 Spider bites, necrotic arachnidism	18.26
**CHAPTER 19 - PREMATURITY AND NEONATAL CONDITIONS**		19.1
19.1	Apnoea, neonatal	19.1
19.2	Cyanotic heart disease in the newborn	19.3
19.3	Enterocolitis, necrotising	19.5
19.4	Haemorrhagic disease of the newborn	19.7
19.5	Heart failure in neonates	19.9
19.6	Hypocalcaemia, neonatal	19.12
19.7	Hypoglycaemia, neonatal	19.13
19.8	Hypoxia/ischaemia of the newborn (perinatal hypoxia/hypoxic-ischaemic encephalopathy)	19.15
19.9	Jaundice, neonatal	19.20
19.9.1	Hyperbilirubinaemia, unconjugated	19.21
19.9.2	Hyperbilirubinaemia, conjugated	19.22
19.10	Jaundice, neonatal, prolonged	19.23
19.11	Meningitis bacterial, neonatal	19.28
19.12	Patent ductus arteriosus (PDA) in the newborn	19.30

19.13	Prematurity/preterm neonate	19.31
19.14	Respiratory distress in the newborn	19.34
19.15	Resuscitation of the newborn	19.38
19.16	Seizures, neonatal	19.41
19.17	Septicaemia of the newborn	19.44
19.18	Syphilis, early congenital	19.46
19.19	Tetanus, neonatal	19.48
**CHAPTER 20 - PAIN CONTROL & PALLIATIVE CARE IN PAEDIATRICS**		20.1
20.1	Management of pain	20.1
20.2	Palliative care	20.6
**CHAPTER 21 - INTENSIVE CARE AND ANAESTHETICS**		21.1
21.1	Sedation for intensive care procedures	21.1
21.1.1	ICU sedation, neonate	21.1
21.1.2	ICU sedation, infant and child	21.2
21.2	Parenteral nutrition	21.4
21.2.1	Parenteral nutrition, neonatal	21.5
21.2.2	Parenteral nutrition, paediatrics	21.6
21.3	Anaesthetic and post anaesthetic care of children	21.7
21.3.1	Local and regional anaesthesia	21.7
21.3.2	General anaesthesia	21.8
	21.3.2.1 Preparation	21.8
	21.3.2.2 Induction	21.9
	21.3.2.3 Maintenance	21.11
21.3.3	Post operative care	21.12
21.3.4	Management of anaesthetic and post anaesthetic complications	21.13
**CHAPTER 22 - ADOLESCENCE**		22.1
22.1	Adolescent chronic disease: transition of care	22.2
22.2	Contraception, teenage pregnancy and teratogenicity risks	22.4
22.3	Hypogonadism in chronic disease	22.4
22.4	Metabolic syndrome associated cardio-metabolic risk	22.6
22.5	Acne	22.7
22.6	Diabetes in adolescence	22.7
22.7	Obesity in adolescence	22.9

22.8 Anorexia nervosa, bulimia nervosa (eating disorders) 22.9

CHAPTER 23 - DRUG ALLERGIES 23.1

23.1 Drug allergies 23.1

23.2 Immediate hypersensitivity reactions 23.2

23.2.1 Drug related anaphylaxis 23.2

23.2.2 Drug related urticaria 23.2

23.2.3 Drug related angioedema 23.2

23.3 Delayed hypersensitivity reactions 23.3

23.4 Specific allergies 23.3

23.4.1 Allergies to penicillins 23.3

23.4.2 Allergies to sulphonamides 23.5

Guideline for the motivation of a new medicine on the National Essential Medicines List xxxii

Guidelines for adverse drug reaction reporting xxxvi

Disease notification procedures xlv

Using the Road to Health booklet xlix

Index of conditions lv

Index of medicines lxvi

Abbreviations lxxix

THE ESSENTIAL MEDICINES CONCEPT

The WHO describes Essential medicines as those that satisfy the priority health care needs of the population. Essential medicines are intended to be available within the context of functioning health systems at all times in adequate quantities, in the appropriate dosage forms, with assured quality and adequate information, and at a price the individual and the community can afford.

The concept of essential medicines is forward-looking. It incorporates the need to regularly update medicines selections to:

» reflect new therapeutic options and changing therapeutic needs;
» the need to ensure medicine quality; and
» the need for continued development of better medicines, medicines for emerging diseases, and medicines to meet changing resistance patterns.

Effective health care requires a judicious balance between preventive and curative services. A crucial and often deficient element in curative services is an adequate supply of appropriate medicines. In the health objectives of the National Drug Policy, the government of South Africa clearly outlines its commitment to ensuring availability and accessibility of medicines for all people. These are as follows:

» To ensure the availability and accessibility of essential medicines to all citizens.
» To ensure the safety, efficacy and quality of drugs.
» To ensure good prescribing and dispensing practices.
» To promote the rational use of drugs by prescribers, dispensers and patients through provision of the necessary training, education and information.
» To promote the concept of individual responsibility for health, preventive care and informed decision-making.

Achieving these objectives requires a comprehensive strategy that not only includes improved supply and distribution, but also appropriate and extensive human resource development. The implementation of an Essential Drugs Programme (EDP) forms an integral part of this strategy, with continued rationalisation of the variety of medicines available in the public sector as a first priority. The private sector is encouraged to use these guidelines and drug list wherever appropriate.

The criteria for the selection of essential drugs for Primary Health Care in South Africa were based on the WHO guidelines for drawing up a national EDL. Essential medicines are selected with due regard to disease prevalence, evidence on efficacy and safety, and comparative cost.

The implementation of the concept of essential medicines is intended to be flexible and adaptable to many different situations. It remains a national responsibility to determine which medicines are regarded as essential.

HOW TO USE THIS BOOK

Principles

The National Drug Policy makes provision for an Essential Drugs Program (EDP) which is a key component in promoting rational medicines use.

Each treatment guideline in the Paediatric Hospital Level Standard Treatment Guidelines (STGs) and Essential Medicines List (EML) has been designed as a progression in care from the current Primary Health Care (PHC) STGs and EML. In addition, where a referral is recommended, the relevant medicines have either been reviewed and included in the tertiary level EML, or is in the process of being reviewed. Given that the PHC STGs and EML are reviewed prior to the Paediatric Hospital Level technical consideration may dictate that there is a period when the two STGs are not always perfectly aligned.

All reasonable steps have been taken to align the STGs with Department of Health guidelines that were available at the time of review.
A medicine is included or removed from the list using an evidence based medicine review of safety and effectiveness, followed by consideration of cost and other relevant practice factors.

The EML has been developed down to generic or International Non-propriety Name (INN) level. It is anticipated that each Province will review the EML and prevailing tenders to compile a formulary which:

» lists formulations and pack sizes that will facilitate care in alignment with the STG;
» selects the preferred member of the therapeutic class based on cost;
» Implements formulary restrictions consistent with the local environment; and
» provides information regarding the prices of medicines.

Therapeutic classes are designated in the “Medicine treatment” section of the STGs which provides a class of medicines followed by example such as, HMGCoA reductase inhibitors (statins) e.g. simvastatin. These therapeutic classes have been designated where none of the members of the class offer a significant benefit over the other registered members of the class. It is anticipated that by limiting the listing to a class there is increased competition and hence an improved chance of obtaining the best possible price in the tender process. In circumstances where you encounter such a class always consult the local formulary to identify the example that has been approved for use in your facility.

The perspective adopted is that of a competent medical officer practicing in a public sector hospital. As such, the STGs serve as a standard for practice but do not replace sound clinical judgment.

Navigating the book

It is important that you become familiar with the contents and layout of the book in order to use the STGs effectively.

Where relevant this book is consistent with the Standard Treatment Guidelines for Primary Health Care, Integrated Management of Childhood Illness Strategy (IMCI) guidelines and other National Programme treatment guidelines.

The ICD-10 number, included with the conditions, refers to an international classification method used when describing certain diseases and conditions. A brief description and diagnostic criteria are included to assist the medical officer to make a diagnosis. These guidelines also make provision for referral of patients with more complex and uncommon conditions to facilities with the resources for further investigation and management. The dosing regimens provide the recommended doses used in usual circumstances however the final dose should take into consideration capacity to eliminate the medicine, interactions and comorbid states.

It is important to remember that the recommended treatments provided in this book are guidelines only and are based on the assumption that prescribers are competent to handle patients' health conditions presented at their facilities.

The STGs are arranged into chapters according to the organ systems of the body. Conditions and medicines are cross referenced in two separate indexes of the book. In some therapeutic areas that are not easily amenable to the development of a STG, the section is limited to a list of medicines.

This edition of the Paediatric Hospital Level STG and EML provides additional information regarding Patient Adherence in Chronic Conditions, Measuring Medication Level and Prescription Writing.

Finally, the guidelines make provision for referral of patients with more complex and uncommon conditions to facilities with the resources for further investigation and management.

The section on Patient Education in Chronic Conditions aims to assist health workers to improve patient adherence and health, generally.

Medicines Safety

Provincial and local Pharmaceutical and Therapeutics Committees (PTCs) should develop medicines safety systems to obtain information regarding medication errors, prevalence and importance of adverse medicine events, interactions and medicines quality. These systems should not only support the regulatory pharmacovigilance plan but should also provide pharmacoepidemiology data that will be required to inform future essential medicines decisions as well as local interventions that may be required to improve safety.

In accordance with the Medicines Control Council's guidance on reporting adverse drug reactions in South Africa, the medical officer with the support of the PTC should report the relevant adverse reactions to the National Adverse Drug Event Monitoring Centre (NADEMC). To facilitate reporting a copy of the form and guidance on its use has been provided at the back of the book.

Feedback
Comments that aim to improve these treatment guidelines will be appreciated. The submission form and guidelines for completing the form are included in the book. Motivations will only be accepted from the Provincial PTC.

MEASURING MEDICATION LEVELS

Potentially toxic medicines, medicines with narrow therapeutic indices and those with variable pharmacokinetics should be monitored regularly to optimise dosing, obtain maximum therapeutic effect, limit toxicity and assess compliance.

Routine measurement is rarely warranted, but rather should be tailored to answering a specific clinical question, and is of most value in medicines with a narrow therapeutic index or where there is considerable individual variation in pharmacokinetics.

Lithium
Measure serum levels at about 12 hours after the last dose – e.g. in the morning before that day's first dose. Levels should be less than 1 mmol/L and should be checked regularly while on therapy, with more frequent monitoring in the elderly and frail.

Aminoglycosides
Peak levels will be adequate if dosing is adequate (5 mg/kg/day in a single daily dose); trough levels taken immediately before the next dose are valuable in identifying potential toxicity before it manifests as deafness or renal impairment.
Aminoglycosides are contraindicated in renal impairment.

Anti-epileptics
Levels may be helpful to confirm poor adherence or to confirm a clinical suspicion of toxicity. Routine measurement in patients with well controlled seizures and no clinical evidence of toxicity is not appropriate. Individual levels may be difficult to interpret – if in doubt, seek assistance from a clinical pharmacokineticist.

PRESCRIPTION WRITING

Medicines should be prescribed only when they are necessary for treatments following clear diagnosis. Not all patients or conditions need prescriptions for

medicine. In certain conditions simple advice and general and supportive measures may be more suitable.

In all cases carefully consider the expected benefit of a prescribed medication against potential risks. This is important during pregnancy where the risk to both mother and foetus must be considered.

All prescriptions should:

- » be written legibly in ink by the prescriber with the full name and address of the patient, and signed with the date on the prescription form;
- » specify the age and, in the case of children, weight of the patient;
- » have contact details of the prescriber e.g. name and telephone number.

In all prescription writing the following should be noted:

- » The name of the medicine or preparation should be written in full using the generic name.
- » No abbreviations should be used due to the risk of misinterpretation. Avoid the Greek mu (ų): write mcg as an abbreviation for micrograms.
- » Avoid unnecessary use of decimal points and only use where decimal points are unavoidable. A zero should be written in front of the decimal point where there is no other figure, e.g. 2 mg not 2.0 mg or 0.5 mL and not .5 mL.
- » Frequency. Avoid Greek and Roman frequency abbreviations that cause considerable confusion – qid, qod, tds, tid, etc. Instead either state the frequency in terms of hours (e.g. 8 hourly) or times per day in numerals (e.g. 3x/d)
- » State the treatment regimen in full:
  - o medicine name and strength,
  - o dose or dosage,
  - o dose frequency,
  - o duration of treatment,

    e.g. amoxicillin 250 mg 8 hourly for 5 days.
- » In the case of "as required", a minimum dose interval should be specified, e.g. every 4 hours as required.
- » Most monthly outpatient scripts for chronic medication are for 28 days; check that the patient will be able to access a repeat before the 28 days are completed.
- » After writing a script, check that the dose, dose units, route, frequency, and duration for each item is stated. Consider whether the number of items is too great to be practical for the patient, and check that there are no redundant items or potentially important drug interactions. Check that the script is dated and that the patient's name and folder number are on the prescription form. Only then sign the script, and as well as signing provide some other way for the pharmacy staff to identify you if there are problems (print your name, use a stamp, or use a prescriber number from your institution's pharmacy.)

A GUIDE TO PATIENT ADHERENCE IN CHRONIC CONDITIONS

Achieving health goals for chronic conditions such as asthma, diabetes, HIV and AIDS, epilepsy, hypertension, mental health disorders and TB requires attention to:

» Adherence to long term pharmacotherapy – incomplete or non-adherence can lead to failure of an otherwise sound pharmacotherapeutic regimen.
» Organisation of health care services, which includes consideration of access to medicines and continuity of care

Patient Adherence

Adherence is the extent to which a person's behaviour – taking medication, following a diet and/or executing lifestyle changes, corresponds with agreed recommendations from a health care provider.

Poor adherence results in less than optimal management and control of the illness and is often the primary reason for suboptimal clinical benefit. It can result in medical and psychosocial complications of disease, reduced quality of life of patients, and wasted health care resources.

Poor adherence can fall into one of the following patterns where the patient:

» takes the medication very rarely (once a week or once a month);
» alternates between long periods of taking and not taking their medication e.g. after a seizure or BP reading;
» skips entire days of medication;
» skips doses of the medication;
» skips one type of medication;
» takes the medication several hours late;
» does not stick to the eating or drinking requirements of the medication;
» adheres to a purposely modified regimen; and
» adheres to an unknowingly incorrect regimen.

Adherence should be assessed on a regular basis. Although there is no gold standard, the current consensus is that a multi method approach that includes self report be adopted such as that below. .

Barriers that contribute toward poor adherence

BARRIER	RECOMMENDED SUPPORT
**Life style**	
» It is often difficult to take multiple medications.	» Create a treatment plan with information on how and when to take the medications.
» A busy schedule makes it difficult to remember to take the medication.	» Use reminders such as cues that form part of the daily routine.

BARRIER	RECOMMENDED SUPPORT
**Attitudes and beliefs**	
» The condition is misunderstood or denied.	» Remind patients that they have a long term illness that requires their involvement.
» Treatment may not seem to be necessary.	» Use change techniques such as motivational interviewing.
» May have low expectations about treatment.	» Identify goals to demonstrate improvement/stabilisation.
**Social and economic**	
» May lack support at home or in the community	» Encourage participation in treatment support programs.
» May not have the economic resources to attend appointments.	» Consider down referral or reschedule appointment to fit in with other commitments.
**Healthcare team related**	
» Little or no time during the visit to provide information.	» Encourage patient to ask questions.
» Information maybe provided in a way that is not understood.	» Use patient literacy materials in the patient's language of choice.
» Relationship with the patient may not promote understanding and self management.	» Engage active listening.
**Treatment related**	
» Complex medication regimens (multiple medications and doses) can be hard to follow.	» If possible reduce treatment complexity
» May be discouraged if they don't feel better right away.	» Help the patient understand the condition and the role of their medication
» May be concerned about adverse effects.	» Discus treatment goals in relation to potential adverse effects.

Although many of these recommendations require longer consultation time, this investment is rewarded many times over during the subsequent years of management.

For a patient to consistently adhere to long term pharmacotherapy requires integration of the regimen into his or her daily life style. The successful integration of the regimen is informed by the extent to which the regimen differs from his or her established daily routine. Where the pharmacological proprieties of the medication permits it, the pharmacotherapy dosing regimen should be adapted to the patient's daily routine. For example, a shift worker may need to take a sedating medicine in the morning when working night shifts, and at night, when working day shifts. If the intrusion into life style is too great alternative agents should be considered if they are available. This would include situations such as a lunchtime dose in a school-going

child who remains at school for extramural activity and is unlikely to adhere to a three times a regimen but may very well succeed with a twice daily regimen.

Towards concordance when prescribing

Establish the patient's:

» occupation,
» daily routine,
» recreational activities,
» past experiences with other medicines, and
» expectations of therapeutic outcome.

Balance these against the therapeutic alternatives identified based on clinical findings. Any clashes between the established routine and life style with the chosen therapy should be discussed with the patient in such a manner that the patient will be motivated to a change their lifestyle.

Note:

Education that focuses on these identified problems is more likely to be successful than a generic approach toward the condition/medicine.

Education points to consider

» Focus on the positive aspects of therapy whilst being encouraging regarding the impact of the negative aspects and offer support to deal with them if they occur.
» Provide realistic expectations regarding:
  – normal progression of the illness - especially important in those diseases where therapy merely controls the progression and those that are asymptomatic;
  – the improvement that therapy and non-drug treatment can add to the quality of life.
» Establish therapeutic goals and discuss them openly with the patient.
» Any action to be taken with loss of control or when side effects develop.
» In conditions that are asymptomatic or where symptoms have been controlled, reassure the patient that this reflects therapeutic success, and not that the condition has resolved.
» Where a patient raises concern regarding anticipated side effects, attempt to place this in the correct context with respect to incidence, the risks vs. the benefits, and whether or not the side effects will disappear after continued use.

Note

Some patient's lifestyles make certain adverse responses acceptable which others may find intolerable. Sedation is unlikely to be acceptable to a student but an older patient with insomnia may welcome this side effect. This is where concordance plays a vital role.

Notes on prescribing in chronic conditions.

» Don't change doses without good reason.
» Never blame anyone or anything for non-adherence before fully investigating the cause.

- If the clinical outcome is unsatisfactory - investigate adherence (remember side effects may be a problem here).
- Always think about side effects and screen for them from time to time.
- When prescribing a new medicine for an additional health related problem ask yourself whether or not this medicine is being used to manage a side effect.
- Adherence with a once daily dose is best. Twice daily regimens show agreeable adherence. However once the interval is decreased to 3 times a day there is a sharp drop in adherence with poor adherence to 4 times a day regimens.
- Keep the total number of tablets to an absolute minimum as too many may lead to medication dosing errors and may influence adherence

Improving Continuity of Therapy

- Make clear and concise records.
- Involvement the patient in the care plan.
- Every patient on chronic therapy should know:
  - his/her diagnosis
  - the name of every medicine
  - the dose and interval of the regimen
  - his/her BP or other readings

  Note: The prescriber should reinforce this only once management of the condition has been established.
- When the patient seeks medical attention for any other complaints such as a cold or headache he/she must inform that person about any other condition/disease and its management
- If a patient indicates that he/she is unable to comply with a prescribed regimen, consider an alternative - not to treat might be one option, but be aware of the consequences e.g. ethical

Patient Adherence Record

Folder No.	Date (dd/mm/yyyy) / /

Self-Reporting

**Question**	**Yes**	**No**
Do you sometimes find it difficult to remember to take your medicine?		
When you feel better, do you sometimes stop taking your medication?		
Thinking back over the past four days, have you missed any of your doses?		
Sometimes if you feel worse when you take the medicine, do you stop taking it?		

Visual Analogue Scale (VAS)

0 1 2 3 4 5 6 7 8 9 10

Score ____%

Pill Identification Test (PIT)

Medication	Knows the name (Y/N)	Knows the number of pills per dose (Y/N)	Time the medication is taken			Knows any additional instruction
			Morning (hour)	Evening (hour)	Considered Acceptable (Y/N)	

Pill Count

Did the client return the medication containers? ☐ Yes* ☐ No

***If yes,** check that the client only used medication from this container since the date of their last visit. If leftover medication had been used or an emergency prescription obtained, then the calculation will be invalid – skip to adherence assessment.

$$\textbf{\% Adherence} = \frac{\textbf{Dispensed} - \textbf{Returned}}{\textbf{Expected to be taken}} \times 100 = \frac{\square - \square}{\square} \times 100 = \square\ \%$$

Adherence Assessment

**Self-reporting**	Answered 'No' to all questions	Answered 'Yes' to 1 question	Answered 'Yes' to 2 or more questions
**VAS**	> 95%	75–94%	Less than 75%
**PIT—***Client knows the…*	Dose, Time, and Instructions	Dose and Time	Dose only or confused
**Pill count**	> 95%	75–94%	Less than 75%
**Overall Adherence**	High	Moderate	Low

CHAPTER 1

EMERGENCIES AND TRAUMA

1.1 PAEDIATRIC EMERGENCIES

Certain emergencies of the airway, breathing, circulation and neurological systems are dealt with in the chapters on respiratory, cardiac and nervous system, respectively. This section deals only with the approach to the severely ill child and selected conditions (cardiorespiratory arrest), anaphylaxis, shock, foreign body inhalation and burns. All doctors should ensure that they have received appropriate training to provide at least the basic (and preferably advanced) life support to children.

The most experienced clinician present should take control of the resuscitation.

1.1.1 RAPID TRIAGE OF THE CHILD PRESENTING WITH ACUTE CONDITIONS IN CASUALTY/OUT PATIENTS

Many deaths in hospital/health centres occur at or early after presentation. Some of these deaths can be prevented if very sick children are quickly identified upon arrival and treatment is started without delay.

The word "triage" means sorting. The idea of triage is to identify very sick children who will benefit from immediate emergency care from those who should receive priority care (be placed ahead of the normal queue) or those who can wait to be seen in the normal order of arrival. Triage is the process of rapidly examining all sick children when they first arrive in hospital in order to place them in one of these categories and should be reassessed regularly while awaiting care.

Categories

1. Emergencies: Conditions which cannot wait and require immediate treatment.
2. Priority signs (place ahead of normal queue).
3. Non-urgent (queue).

Emergencies: conditions which cannot wait and require immediate treatment

If any emergency sign positive: give emergency treatment(s), call for help, and draw blood for emergency laboratory investigations.

(A&B) Airway and breathing

» Not breathing.

or

» Obstructed breathing.

or

» Central cyanosis.

or

» Severe respiratory distress.

(C) Circulation

» Cold hands.

and

» Capillary refill $\geq$ 3 seconds.

and

» Weak and fast pulse.

(C) Coma/convulsing

» Coma.

or

» Convulsing (now).

(D) Severe dehydration (only in child with diarrhoea)

Diarrhoea plus any two of these:

» Lethargy.
» Sunken eyes.
» Very slow skin pinch.

Priority signs

These children need prompt assessment and treatment:

» tiny baby (< 3 months),
» temperature very high or very low,
» trauma or other urgent surgical condition,
» pallor (severe),
» poisoning (history of),
» pain (severe),
» respiratory distress,
» restless, continuously irritable, or lethargic,
» referral urgent (from another health professional),
» malnutrition: visible severe wasting,
» oedema of both feet,
» burns (major).

Non-urgent (queue)
Proceed with assessment and further treatment according to the child's priority.

A number of different triage processes exist and the above is taken from the South African Emergency Triage Assessment and Treatment (ETAT) course.

Other systems may include, in addition, the use of clinical markers such as respiratory rate, blood pressure and pulse rate to add precision to the triage, especially in more resourced settings.

Other important conditions are sometimes added to the ETAT guidelines to suit particular local conditions such as identifying infectious diseases that need immediate isolation, dehydration (not severe), facial or inhalational burns, evidence of meningococcal septicaemia, inconsolable crying, etc.

The ETAT triage presented above should be a minimum standard of triage in community health centres, district or regional hospitals in South Africa. Additional items may be added suitable to local conditions and resources.

1.1.2 APPROACH TO THE RESUSCITATION OF THE CHILD

In approaching a child with potential severe illness or injury a structured approach will improve the child's chances of a best possible outcome in the shortest possible time. The following is a diagrammatic overview derived from an advanced Paediatric life support approach.

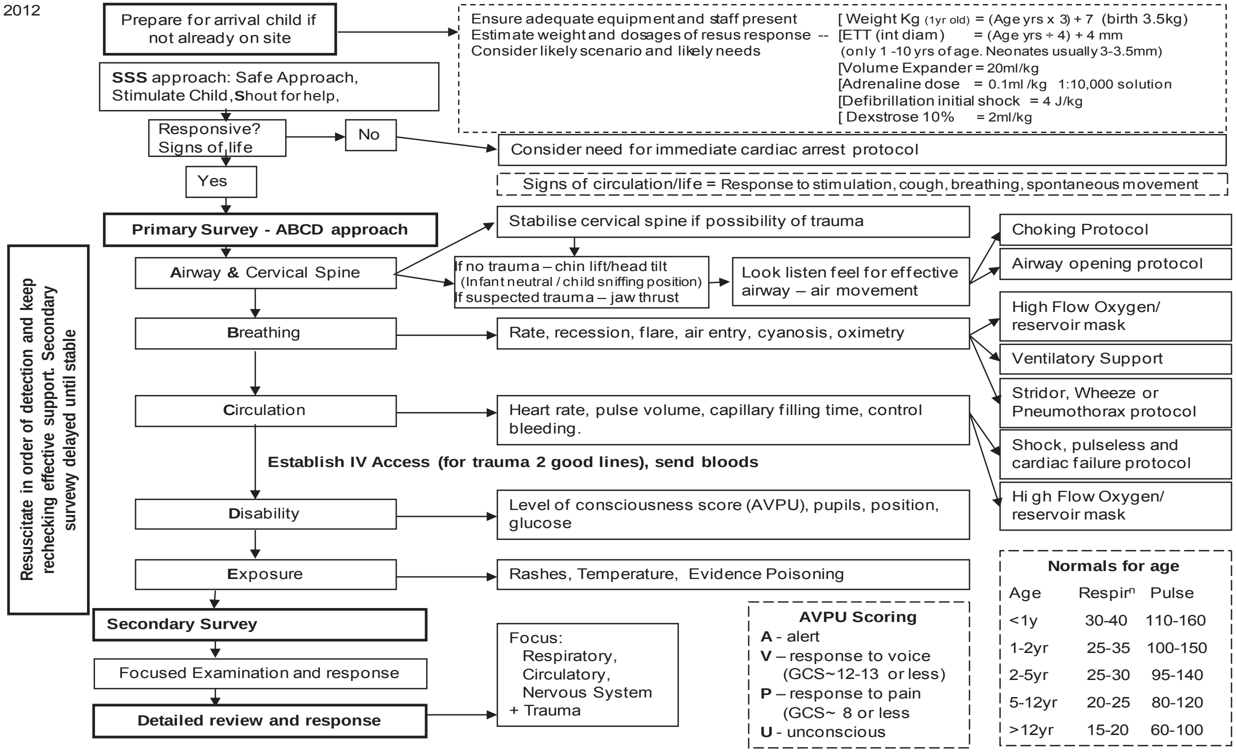

Normals for age

Age	Respir^{n}	Pulse
<1y	30-40	110-160
1-2yr	25-35	100-150
2-5yr	25-30	95-140
5-12yr	20-25	80-120
>12yr	15-20	60-100

A brief summary of the approach and primary survey adapted from paediatric life support documentation – for comprehensive competence an advanced paediatric life support course should be attended.

To optimise oxygen delivery to the tissue:

- Oxygen, high flow, 15 L/minute via facemask with reservoir bag **or** 6–10 L/minute via head box.
  - o If oxygen saturation < 92% or PaO_2 < 80 mmHg in spite of oxygen supply consider need for ventilation and continue respiratory support.

1.1.3 ANAPHYLAXIS/ANAPHYLACTIC REACTIONS

T78.2

DESCRIPTION

An acute, potentially life-threatening hypersensitivity reaction starting within seconds to minutes after administration of, or exposure to, a substance to which the individual is sensitised. Clinical manifestations range from mild urticaria and angioedema to upper airway obstruction, bronchospasm, hypotension, shock and death.

The reaction can be short-lived, protracted or biphasic, i.e. acute with recurrence several hours later. Immediate reactions are usually the most severe and/or life threatening.

DIAGNOSTIC CRITERIA

Clinical

» Acute onset of signs and symptoms.
» Dizziness, paraesthesia, syncope, sweating, flushing, dysrrhythmias.
» Swelling of eyes, lips and tongue (angioedema).
» Angioedema with upper airway obstruction and stridor.
» Hypotension and shock.
» Bronchospasm, wheezing, dyspnoea, chest tightness.
» Gastrointestinal symptoms such as nausea, vomiting, diarrhoea.

A life-threatening anaphylactic reaction requires **<u>immediate</u>** treatment.
Facilities to initiate treatment must be available at all health centres.

GENERAL AND SUPPORTIVE MEASURES

» Place hypotensive or shocked patient in horizontal position. Do not place patient in a sitting position.
» Secure an open airway. If necessary, bag via mask or intubate.
» Observe for 24 hours.

MEDICINE TREATMENT

To maintain arterial oxygen saturation ≥ 95% and to abolish cyanosis:

- Oxygen, 100%, at least 1–2 L/minute by nasal prong.

In severe anaphylaxis nasal oxygen is unlikely to be adequate:
- Oxygen, 100%, 15 L/minute by face mask.

- Epinephrine (adrenaline) 1:1 000, IM, 0.01 mL/kg. (i.e. 10 mcg/kg).
  - Can be repeated every 5 minutes, if necessary.
  - Maximum dose: 0.5 mL.
  - Do not administer IV unless there is failure to respond to several doses of IM.
  - Monitor urine output.

Intravenous fluids
Crystalloid solutions e.g.:
- Sodium chloride 0.9%, IV, 20 mL/kg as a bolus.
  - Repeat if necessary until circulation, tissue perfusion and blood pressure improve (up to 40 mL/kg).

- Promethazine, IV/IM, 0.25–0.5 mg/kg/dose.

Then continue with:
- Chlorphenamine, oral, 0.1 mg/kg/dose 6 hourly for 24–48 hours, if necessary.

If associated bronchospasm:
- Salbutamol, nebulised, 1 mL salbutamol respirator solution in 3 mL sodium chloride 0.9%.
  - Nebulise at 20-minute intervals.

- Hydrocortisone, IV, 5 mg/kg, 4–6 hourly for 12–24 hours.
  - Note: Steroids are adjunctive therapy and are not first line treatment, and should never be the sole treatment of anaphylaxis.

If associated stridor:
- Epinephrine (adrenaline), 1:1000, nebulise with oxygen, every 15–30 minutes until expiratory obstruction is abolished.
  - 1 mL epinephrine 1:1 000 diluted in 1 mL sodium chloride 0.9%.

PREVENTATIVE MEASURES AND HOME BASED TREATMENT
» Obtain a history of allergies/anaphylaxis on all patients before administering medication/immunisation.
» Identify offending agent and avoid further exposure.
» See patient wears allergy identification disc/bracelet.
» Train patients to self-administer epinephrine pre-filled auto injecting device. Specialist initiated for patients who have clear documented anaphylactic reactions.
» Educate patient and parent/caregiver on allergy and anaphylaxis.

REFERRAL

Caution
Do not refer the patient during the acute phase.
Transfer can only be done once patient is stable.
Patients supplied with self administered epinephrine must be informed of the shelf life of epinephrine and when they must come in to get a replacement for these.

» Bee sting anaphylaxis for desensitisation.

1.1.4 CARDIORESPIRATORY ARREST

I46.9

DESCRIPTION

Cardiorespiratory arrest in children is usually the end result of a period of circulatory or respiratory insufficiency and is seldom due to a sudden precipitous cardiac event. It is therefore important to pre-empt cardiorespiratory arrest in children by recognising and urgently treating respiratory or circulatory failure.

Cardiorespiratory arrest is diagnosed clinically in the unresponsive child who displays no respiratory effort and/or in whom there is no palpable pulse and no signs of life, i.e. cough or spontaneous movement.

GENERAL AND SUPPORTIVE MEASURES

Always call immediately for help from your colleagues on site.

Ensure an open airway.

If there is still no respiration, then commence with artificial breathing using a self-inflating bag, with a reservoir and an appropriate mask. Connect the bag to a high flow oxygen source (15 L/minute).
Movement of the chest in response to artificial breaths should be evident.

If there is no, or inadequate, air movement with bag-valve-mask ventilation, re-assess the airway with appropriate chin-lift/jaw-thrust manoeuvres. If necessary, place an appropriate sized endotracheal tube. In the event of an unexpected arrest or an arrest where there are no witnesses, consider the possibility of a foreign body obstruction. See section 1.1.6: Inhalation, foreign body.

Once effective breathing has been established, provide chest compressions at a rate of 100–120/minute for all children excluding neonates. Provide artificial breaths at a ratio of 15 compressions to 2 breaths in children (15:2).
Attach a cardiac monitor to the child and insert an intravenous line. If failure to insert IV line, insert intra-osseous line. See section 1.1.8: Intra-Osseous Infusion in Emergencies.

MEDICINE TREATMENT

Asystole or pulseless electrical activity (ie no palpable pulse even if normal electrical pattern)

- Epinephrine (adrenaline) **1:10 000,** IV/ intra-osseous, 0.1 mL/kg. (Follow each dose with a small bolus of sodium chloride 0.9%).
    - 0.1 mL of 1: 10 000 solution = 10 mcg.
    - Dilute a 1 mL ampoule of epinephrine (adrenaline) 1:1 000 in 9 mL of sodium chloride 0.9% or sterile water to give a 1: 10 000 solution.

OR (less ideally)

- Epinephrine (adrenaline) 1:1 000, endotracheal, undiluted 0.1 mL/kg down an endotracheal tube. (This is a higher dose due to the route of administration).

Repeat the dose of epinephrine (adrenaline) every 4 minutes if asystole persists while CPR continues.
When an ECG sinus rhythm trace is present continue CPR until an effective pulse and circulation is present.

If the arrest was preceded by circulatory shock:

- Sodium chloride 0.9%, IV, 20 mL/kg as a bolus.

During the resuscitation consider if any of the following correctable conditions are present (and if present correct them):

» Hypoxia.
» Hypovolaemia.
» Hyperkalaemia, hypokalaemia, hypocalcaemia.
» Hypothermia.
» Tension pneumothorax.
» Tamponade (cardiac).
» Toxins (e.g. tricyclic antidepressants).
» Thrombo-embolic event.

Note:
There is no evidence to support the **routine** use of any of the following in cardiac arrest:

» sodium bicarbonate,
» calcium,
» high dose IV epinephrine (adrenaline) (100 mcg/kg/dose).

Ventricular fibrillation or pulseless ventricular tachycardia

Proceed to immediate defibrillation but during this process cardiorespiratory resuscitation (compressions and ventilation) must continue, except during the actual administration of each shock. Continue until adequate circulation can be demonstrated.

For pulseless ventricular tachycardia and ventricular fibrillation the defibrillator should be set to asynchronous mode and voltage at 4 J/kg.

Do not increase voltage, give 4 J/kg repeatedly, if needed.

After each shock continue CPR for 2 minutes and only re-assess the ECG rhythm thereafter.
If fibrillation/ventricular tachycardia has changed back to sinus rhythm, stop shock cycle, but continue CPR until good stable circulation and adequate spontaneous breathing is evident.
If fibrillation/ventricular tachycardia is still present, give further shocks for 3 x 2-minute cycles of shocks.

Thereafter, if necessary, the 2-minute shock cycles should continue but, in addition, give the following after the 3rd shock:

- Epinephrine (adrenaline) **1:10 000,** IV, 0.1 mL/kg and then repeat after every 2nd shock, i.e. every 4 minutes. (Follow each dose with a small bolus of sodium chloride 0.9%).
  - 0.1 mL of 1: 10 000 solution = 10 mcg.
  - Dilute a 1 mL ampoule of epinephrine (adrenaline) 1:1 000 in 9 mL of sodium chloride 0.9% or sterile water to give a 1: 10 000 solution.

After the 3rd and 5th shocks, if normal rhythm has not returned:

- Amiodarone, IV/IO, 5 mg/kg bolus administered over 3–5 minutes.

Allow one minute of cardiopulmonary resuscitation between the administration of any medicine and a repeat cycle of shocks.

If ventricular fibrillation or pulseless ventricular tachycardia persists consider the following (and if present correct):

» Hypoxia.
» Hypovolaemia
» Hyperkalaemia, hypokalaemia, hypocalcaemia.
» Hypothermia.
» Tension pneumothorax.
» Tamponade (cardiac).
» Toxins (e.g. tricyclic antidepressants).
» Thrombo-embolic event.

REFERRAL

» To an intensive care unit after recovery from an arrest.

1.1.5 CONVULSIONS, NOT FEBRILE CONVULSIONS

See section 13.2: Seizures.

1.1.6 INHALATION, FOREIGN BODY

T17.9

DESCRIPTION

Accidental inhalation of solid object that may obstruct the airway at any level.

DIAGNOSTIC CRITERIA

Ask specifically about a possible choking episode if there is any suspicion of a foreign body aspiration.

» Initial symptom is frequently a sudden onset of choking followed by persistent unilateral wheeze (may be bilateral), chronic cough, stridor or the child may die suddenly even up to a few days later.
» Segmental or lobar pneumonia failing to respond to standard therapy.
» Signs of shift of the mediastinum.
» Chest X-ray on full expiration and full inspiration may show hyperinflation and/or collapse or sometimes, radio-opaque foreign body.

GENERAL AND SUPPORTIVE MEASURES

ACUTE EPISODE

» If coughing effectively and moving air adequately, provide oxygen and refer urgently for airway visualisation. Carry out transfer with a person who is able to manage the foreign body process accompanying the child.
» If the child is still breathing but unable to cough or breathe adequately, attempt to dislodge the foreign body by cycles of 5 back slaps followed by 5 chest compressions (infants), or 5 Heimlich manoeuvre (child) repeatedly.
» If the child is unconscious carry out standard cardiorespiratory resuscitation, i.e. cardiac compressions and ventilation (15:2).

Caution
Blind finger sweeps are dangerous and absolutely contra-indicated. All cases should have airway visualisation or be referred for airway visualisation.

Foreign body manoeuvres in Children who are not unconscious but unable to cough/breathe adequately.

Infants

» Check the mouth for any obstruction. If visualised, attempt removal under direct vision e.g. with Magills forceps.
» Lay the infant on an arm/thigh in the head down position and strike the back of the chest 5 times firmly with the heel of the hand.
» If no response, turn the infant over on its back and give 5 chest thrusts with 2 fingers in the midline to the lower ½ of the sternum compressing the chest by about 1/3 of its diameter.
» Repeat sequence, if necessary, checking in the mouth after each cycle to see if a removable foreign body can be seen.

Children

» Check the mouth for any obstruction. If visualised, attempt removal under direct vision e.g. with Magills forceps.
» Strike the back of the chest firmly 5 times while the child sits, lies prone or kneels.
» If no response, attempt Heimlich manoeuvre: standing behind the child, pass your arms around the body and form a fist just below the sternum. Thrust upwards 5 times.
» Repeat sequence, if necessary, checking in the mouth after each cycle to see if a removable foreign body can be seen.

REFERRAL

» All cases for the removal of retained foreign bodies.
» Unresolved respiratory complications.

1.1.7 SHOCK

R57.9

DESCRIPTION

An acute syndrome that reflects the inability of the pulmonary and circulatory system to provide adequate perfusion, oxygen and nutrients to meet the physiological and metabolic demands of organs, tissues and cells.

In more common usage it refers to circulatory inadequacy.

Compensation is achieved by increased pulse rate, and peripheral vascular constriction. The blood pressure is relatively well maintained but the patient still requires urgent resuscitation.

Depending on the nature and the intrinsic aetiology, shock can be divided into:

» **Hypovolaemic shock:** loss of intravascular fluid, e.g. dehydration, haemorrhage or fluid shifts.
» **Distributive shock:** e.g. septicaemia and anaphylaxis.
» **Cardiogenic shock:** e.g. cardiac dysfunction.
» **Dissociative shock:** e.g. profound anaemia and carbon monoxide poisoning.
» **Obstructive shock:** e.g. pneumothorax and cardiac tamponade.
» **Septic shock:** many mechanisms are operative in septic shock.
» **Neurogenic shock:** e.g. spinal cord trauma.

Complications of shock include multi-organ dysfunction and/or failure.

DIAGNOSTIC CRITERIA

Evidence of compensated shock include:

» cold peripheries,
» weak pulse pressure especially peripheral pulse weaker than central pulses,
» prolonged capillary filling, i.e. > 3 seconds,
» agitation/confusion/decreased level of consciousness,
» skin pallor,
» increased heart rate,
» signs and symptoms of underlying conditions.

In uncompensated shock, falling BP and failure to act urgently will result in irreversible shock and death.

> Facilities to start treatment of shock must be available at all health centres.

GENERAL AND SUPPORTIVE MEASURES

» Follow the ABCD's algorithm at the beginning of the chapter.
» Identify and treat the underlying cause.
» Ensure good intravenous or intra-osseous access. In trauma, two large bore lines for access are important. See section 1.1.8: Intra-Osseous Infusion in Emergencies.
» Take <u>appropriate</u> bloods, e.g. cross match, urea and electrolytes, coagulation studies, full blood count and blood cultures.
» Monitor:
  > and maintain vital signs,
  > and correct metabolic parameters,
  > urinary output – aim for at least 1 mL/kg/hour.

MEDICINE TREATMENT

To optimise oxygen delivery to the tissue, administer:

- Oxygen, high flow, 15 L/minute via facemask with reservoir bag or 6–10 L/minute via head box.

If oxygen saturation < 92% or PaO_2 < 80 mmHg consider need to intubate and continue respiratory support.

1. Hypovolaemic shock

> Response to each step of management must be reviewed every 15 minutes.
> **If after administration of a total of 40ml/kg of sodium chloride 0.9% fluid, shock has not resolved consider other causes and the need for inotropes.**

<u>For fluid deficit (vs. blood loss):</u>
IV fluids to correct the intravascular fluid deficit and improve circulation:

- Sodium chloride 0.9%, IV, 20 mL/kg rapidly.
  - o Review after each bolus to see if shock has resolved.

In children with **severe malnutrition:**

- Sodium chloride 0.9%, IV, 10 mL/kg administered over 10 minutes.
  - o Review after each bolus to see if shock has resolved.

With each re-assessment, if:

» Shock has resolved (capillary filling time < 3 seconds, good pulse, normal blood pressure), do not repeat bolus. Proceed to other care.
» Shock is better but still present, repeat bolus (up to 40 mL/kg). After this further care should be in an ICU setting. Consider CVP monitoring.
» Monitor for persistence of shock, i.e.
  > Non-responding or decreasing BP.
  > Non-responding or increasing pulse rate/decreasing volume.
  > Non-responding or increasing capillary filling time.
» Monitor for fluid or circulatory overload, i.e.
  > Increasing respiratory rate.
  > Increasing basal crepitations.
  > Increasing pulse rate.
  > Increasing liver size/tenderness.
  > Increasing JVP.

After stabilisation of the circulation, continue with maintenance fluid volumes according to the age of the patient.

<u>For blood loss:</u>

- Packed red cells, 10 mL/kg **or** whole blood, 20 mL/kg.

While awaiting blood for replacement begin volume resuscitation with:

- Sodium chloride 0.9%, IV.

2. Cardiogenic shock

Ideally children receiving treatment for cardiogenic shock should be in high care or ICU.

Inotropic support:
When perfusion is poor and blood pressure response is unsatisfactory, despite adequate fluid replacement.

- Dobutamine, IV, 5–15 mcg/kg/minute.

Chronotropic/inotropic plus vascular tone support:

If tissue perfusion and blood pressure do not improve satisfactorily on adequate fluid volume replacement and inotropic support consider:
- Epinephrine (adrenaline), IV infusion, 0.01–1 mcg/kg/minute.

If poor ventricular contractility and increased afterload are considered as the primary problem, do not give epinephrine (adrenaline) but consider adding an afterload reducing agent to the dobutamine infusion but only with specialist advice.

3. Septic shock

Children receiving treatment for septic shock should be in an ICU.
The resuscitation and treatment of these children must be immediate.

Response to each step of management must be reviewed every 15 minutes.

IV fluids:
- Sodium chloride 0.9%, IV, 20 mL/kg rapidly.
  - o Review after each bolus to see if shock has resolved.

In children with **severe malnutrition:**
- Sodium chloride 0.9%, IV, 10 mL/kg administered over 10 minutes.
  - o Review after each bolus to see if shock has resolved.

With each reassessment, if:
- » Shock has not resolved after 20 ml/kg of sodium chloride 0.9% fluid, consider inotropes.
- » Shock has resolved (capillary filling time < 3 seconds, good pulse, normal blood pressure), do not repeat bolus. Proceed to other care.
- » Shock is better but still present, repeat bolus (up to 40 mL/kg). After this further care should be in an ICU setting.
- » Monitor for persistence of shock, i.e.:
  - > Non-responding or decreasing BP.
  - > Non-responding or increasing pulse rate/decreasing volume.
  - > Non-responding or increasing capillary filling time.

» Monitor for fluid or circulatory overload, i.e.:
  > Increasing respiratory rate.
  > Increasing basal crepitations.
  > Increasing pulse rate.
  > Increasing liver size/tenderness.
  > Increasing JVP.

Chronotropic/Inotropic plus vascular tone support
If tissue perfusion and blood pressure do not improve satisfactorily on adequate fluid volume replacement.
Titrate inotropes against the response, and add additional agent if poor response.

- Epinephrine (adrenaline), IV infusion, 0.01–1 mcg/kg/minute.

If inadequate response:
ADD
- Dobutamine, IV, 5–15 mcg/kg/minute.

Unresponsive septicaemic shock:
- Hydrocortisone, IV, 1 mg /kg/dose, 6 hourly until shock has resolved.

Antibiotic therapy
Start antibiotics early.
Before initiating antibiotic therapy, take blood and urine specimens, if appropriate, for culture and sensitivity testing.
Reconsider antibiotic and/or antifungal therapy when culture and sensitivity results become available.

▪ 3rd generation cephalosporins, e.g.:
- Cefotaxime, IV, 75 mg/kg/dose, 8 hourly (neonates).

OR
Children > 2 months:
- Ceftriaxone, IV, 50 mg/kg/dose, 12 hourly.

Caution
Patients must be resuscitated and stabilised before referral.

1.1.8 INTRA-OSSEOUS INFUSION IN EMERGENCIES

If an intravenous drip cannot be set up within 5 minutes, set up an intra-osseous infusion.

1. Use an intra-osseous needle or if not available FG18 x 1.5 cm (or less ideally FG20 x 1.5 cm) or lumbar puncture needle.
2. Grasp the thigh and knee above and lateral to the insertion site with the palm of the left hand (if right-handed). Wrap the fingers around the knee to stabilise the proximal tibia. Do not allow any portion of your hand to rest behind the insertion site.
3. Find the site of insertion i.e. feel the tibial tuberosity. The site of insertion is about 2 cm below this tuberosity on the broad flat medial surface of the tibia.
4. Careful surgical preparation of the injection site as for lumbar punctures.
5. Insert the needle through the skin over the flat surface of the tibia.
6. Holding the needle low down near the skin, advance the needle through the bony cortex of the tibia, directing the needle perpendicular, i.e. 90° to the long axis, using a gentle but firm twisting or drilling motion.
7. Stop advancing the needle when a sudden decrease in resistance to forward motion of the needle is felt.
8. Remove the stylet from the needle.
9. Slowly inject a small amount of sodium chloride 0.9% through the needle. Check for any signs of increased resistance to injection, increased circumference of the soft tissues of the calf, or increased firmness of the tissue.
10. If the test injection is successful, disconnect the syringe and join an infusion set to the needle. Secure the needle and tubing with tape and support it with a bulky dressing.
11. If the test injection is unsuccessful, i.e. infiltration of the sodium chloride 0.9% into the leg tissue is observed, remove the needle and try again on the other leg.
12. The flow rate should rapidly increase after flushing through. If flow is poor consider the use of a 3 way tap and syringe.

Signs of successful insertion:

- » Sudden decrease in resistance to insertion as the needle passes through the bony cortex.
- » The needle remains upright without support.
- » Fluid flows freely through the needle without evidence of subcutaneous infiltration.

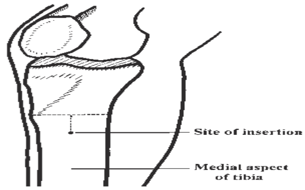

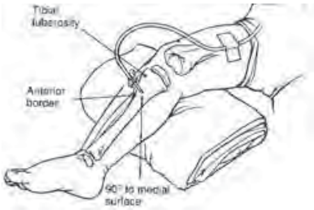

1.2 TRAUMA

1.2.1 BURNS

T30.0

DESCRIPTION

Burns lead to skin and soft tissue injury and may be caused by:

» heat, e.g. open flame, hot liquids, hot steam;
» chemical compounds;
» physical agents, e.g. electrical/lightning;
» radiation.

GENERAL AND SUPPORTIVE MEASURES

Emergency treatment

» Remove smouldering or hot clothing.
» Remove constrictive clothing/rings.
» To limit the extent of the burn, soak the affected area generously in cold water for not more than 10 minutes.
» In all burns > 10% or where carbon monoxide poisoning is possible (enclosed fire, decreased level of consciousness, disorientation) administer high flow oxygen (15 L/minute).
» Examine carefully to determine the extent and depth of the burn wounds.
» Respiratory obstruction due to thermal injury or soot inhalation, production of black coloured sputum, shortness of breath, hoarse voice and stridor are serious signals and may rapidly proceed to respiratory compromise. Consider early endotracheal airway placement.

<u>Further assessment and care</u>

Assessment:

The extent and depth may vary from superficial (epidermis) to full-thickness burns of the skin and underlying tissues.
Initially, burns are usually sterile.

Depth of burn wound	Surface/Colour	Pain sensation/healing
Superficial or epidermal	Dry, minor blisters, erythema	» Painful<br>» Heals within 7 days
Partial thickness superficial or superficial dermal	Blisters, moist	» Painful<br>» Heals within 10–14 days
Partial thickness deep or deep dermal	Moist white or yellow slough, red mottled	» Less painful<br>» Heals within a month or more<br>» Generally needs surgical debridement and skin graft
Full thickness (complete loss of skin)	Dry, charred whitish, brown or black	» Painless, firm to touch<br>» Healing by contraction of the margins (generally needs surgical debridement and skin graft)

Burns are classified as minor or major burns.

Major burns:

- Partial thickness burns of > 10% body surface area.
- Full thickness burn of > 3% body surface area.
- Any burn involving the head and face, hands, feet and perineum.
- Inhalation injuries.
- Circumferential burns.
- Electrical burn injuries.
- Burns in neonates.
- Burns in patients with serious pre-existing or concomitant injuries.

Minor burns:

- Partial thickness burns of < 10% body surface area in a child > 1 year of age.

Estimation of burn surface area

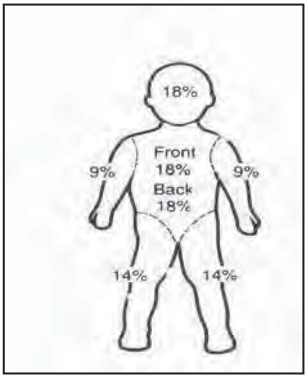

Published with kind permission from SAMJ.
South African Burn Society burn stabilisation protocol.
JS Karpelowsky, L Wallis, A Maderee and H Rode. 2007. SAMJ Vol 9, No 8 Page 574–7.

The figure above is used to calculate body surface area %, and indicates percentages for the whole leg/arm/head (and neck in adults) not the front or back.

» In children the palm of the hand is 1%.
» The following adjustments are made in children up to the age of 8 years old after which adult percentages are used for the head, neck and each leg.
» **Less than 1 year**
  > Head and neck are 18% of BSA.
  > Each leg is 14% of BSA.
» **After the first birthday (> 1 year)**

For each year of life:
  > Head and neck decrease by 1% of BSA until 10% (adult value).
  > Leg gains $^1/_2$ % of BSA until 18% (adult value).

Age Years	Head + neck Front + back	Torso Front	Torso Back	Leg + foot Front + back	Arm+ hand Front+ back
< 1 year	18%	18%	18%	14%	9%
1 to < 2 years	17%	18%	18%	14.5%	9%
2 to < 3 years	16%	18%	18%	15%	9%
3 to < 4 years	15%	18%	18%	15.5%	9%
4 to < 5 years	14%	18%	18%	16%	9%
5 to < 6 years	13%	18%	18%	16.5%	9%
6 to < 7 years	12%	18%	18%	17%	9%
7 to < 8 years	11%	18%	18%	17.5%	9%
8 years and older	10%	18%	18%	18%	9%

Care

Inhalation injury

In addition to other treatment, the degree of inhalation injury may warrant:

- monitoring of blood gases,
- warm humidified oxygen and/or intubation,
- positive pressure ventilation.

Ensure adequate airway in the presence of inhalational burns.
Children with burns may present with delayed onset of airway obstruction.
Consider early intubation.

Suspect carbon monoxide poisoning in all fire victims.

- Obtain carboxyhaemoglobin level.
- Treat by administering 100% oxygen.

Prevent heat loss

Nurse all major burns in a warm room (26°C).

Nasogastric drainage

Use a nasogastric tube on free drainage in all burns > 10% (especially during transfer).
After the 1st 24 hours, commence nasogastric feeds in children who had been started on nasogastric drainage where ileus is not suspected.

Nutritional support

Consult a dietician as children with burns require a higher than usual intake of nutrients.
Start enteral feeds within 6 hours in burns < 10%.

Estimate daily energy and protein needs using the formulae:

Energy (kJ):	250 kJ/kg body mass + (150 kJ x % burned BSA)
Protein:	3 g/kg body mass + (1 g x % burned BSA)
Maximum % burn area used for calculation should not exceed 50%	

Give iron and vitamins routinely until burn wounds are healed and/or skin grafting has successfully been completed.

Note:
Do not supplement iron during sepsis or infection.

In addition, provide:

- psychological support,
- physiotherapy,
- occupational therapy,
- waterbeds and cradles.

MEDICINE TREATMENT

Fluid replacement

Burns < 10% of total body surface area:

- Oral fluids.

Burns > 10% of total body surface area:

- IV fluid for resuscitation.

If in shock first treat shock. See section: 1.1.7: Shock.

As in all fluid administration in sick children volumes are estimates and response must be constantly re-evaluated and rates adjusted appropriately.

CALCULATION OF INITIAL FLUID REPLACEMENT (AFTER SHOCK HAS BEEN TREATED)

First 24 hours:

Replacement fluids for burns

- Sodium chloride 0.9%, IV.
  - o Calculate total fluid requirement in 24 hours:
    [Total % burn ____x weight (kg) ____x 4 mL] as sodium chloride 0.9%.
    Give half of this volume in the 1st 8 hours.
    Administer remaining fluid volume in next 16 hours.

Note:
If urine output not adequate, increase fluids for the next hour by 50% (continue at higher rate until urine output is adequate then resume normal calculated rate).

PLUS

Maintenance fluids in children

In children, give oral or intravenous maintenance fluid in addition to above calculated volume.

Child maintenance fluid requirement volumes	
≤1 year	120 mL/kg/24 hours
All children > 1 year – the sum of the following:	
» for each kg of body weight up to 10 kg	100 mL/kg/24 hours
» for each additional kg of body weight more than 10 kg	50 mL/kg/24 hours
» for each additional kg of body weight more than 20 kg	20 ml/kg/24 hours

Example: 24 kg child with 10% burns	
**1st 24 hours**	
» replacement for expected losses:<br>4 mL/kg x 24kg x 10%	**= 960 mL**
» maintenance:<br>first 10 kg = 10 kg x 100 mL/kg/24 hours<br>second 10 kg = 10 kg x 50 mL/kg/24 hours<br>remaining 4kg = 4 kg x 20 mL/kg/24 hours<br><br>Total maintenance:	 = 1 000 mL+<br>= 500 mL+<br>= 80 mL<br><br>**= 1 580 mL**
**Thus**	
1st 8 hours<br>= ½ resuscitation fluids + ⅓ maintenance fluids	480 mL sodium chloride 0.9%<br>+ 527 mL ½ Darrows/ dextrose 5%
Next 16 hours<br>= ½ resuscitation fluids + ⅔ maintenance fluids	480 mL sodium chloride 0.9%<br>+ 1053 mL ½ Darrows/ dextrose 5%

The above are guidelines, need regular review to maintain urine output 1–2 mL/kg/hour.

Avoid circumferential taping when securing infusion lines, as oedema under the eschar may decrease the venous return.

Second 24 hours:
If urine output is adequate, continue resuscitation:
- Sodium chloride 0.9%, IV, 1.5 mL/kg/% burn/ 24 hours.

PLUS
Maintenance:
- ½ Darrows/dextrose 5%, as per maintenance requirement above.

Part of this volume may be replaced by enteral feeds.

Thereafter progressively decrease IV fluids and increase enteral fluids according to response over time.

Anaemia
If haemoglobin < 7 g/dL:
- Packed red cells, 10 mL/kg over 3 hours.

Hypoalbuminaemia
If indicated by symptomatic hypoalbuminaemia:
- Albumin 20%, IV, 2 g/kg/day. (2 g = 10 mL).

For pain
- Paracetamol, oral, 15 mg/kg/dose 6 hourly as required.

Children with large burns need effective pain relief.
See section 20.1: Management of pain.

Change of dressing
Provide analgesic cover at each dressing change.
In major burns, change dressings under procedural sedation or g eneral anaesthesia.

Administer medicines half an hour before dressing change:
- Paracetamol, oral, 15 mg/kg/dose 6 hourly.

PLUS
- Tilidine, oral, 1 drop per 2.5kg of body weight (i.e. 1 mg/kg/dose).

PLUS
For anxiolysis:
- Midazolam, oral, 0.5 mg/kg/dose.

For severe pain/procedure:
ADD
- Ketamine, oral, 2–5 mg/kg at least 30 minutes before procedure (works best if given in sweet soft drink).

Gastric erosions
Preventative medication treatment is not given. Effective early resuscitation and early feeding decrease the incidence of gastric erosion.

If gastric erosion is suspected due to haematemesis or brownish gastric aspirates.

- Omeprazole, oral, 0.4–0.8 mg/kg/dose 12 hourly. Specialist initiated.
  - Maximum dose: 20–40 mg/dose.
  - If 1 month–2 years: 2.5 mg 12 hourly.
  - If > 2–6 years: 5 mg 12 hourly.
  - If > 7–12 years: 10 mg 12 hourly.

If unable to take orally:
- Ranitidine, IV, 1 mg/kg 6 hourly.

Local treatment of burns
Gently clean the wounds with running water.
Remove loose skin and debride dead tissue and dress with topical antiseptic cream and non-adherent dressing.
Thereafter, daily rinse with running water and dress with topical antiseptic cream and non-adherent dressing.

In < 20% body surface area burns:
- Povidone-iodine 0.5% with occlusive dressings.

In > 20% body surface area burns:

- Silver-sulphadiazine 1%, on non-adhesive dressings.
  - o Cover with paraffin gauze.
  - o Change dressings daily.

Excise and graft all full thickness or deep dermal burns as soon as the patient is stable.
Consider skin grafting in wounds not healed in two weeks.

Antibiotics

Consider if signs of infection are present as these may be subtle:

- » pyrexia/hypothermia,
- » shock (compensated or not compensated),
- » rising pulse or respiratory rate,
- » petechiae.
- » leucocytosis/thrombocytopaenia,
- » looks ill /toxic/altered level of consciousness,
- » local inflammatory changes,

The choice of antibiotics is based on the culture and sensitivity results of wound, urine and blood cultures once available.
Positive wound cultures alone do not indicate systemic infections requiring antibiotic treatment.

- Ceftriaxone, IV, 50 mg/kg/dose 24 hourly for 5 days.

If MRSA is suspected or confirmed, replace with:

- Vancomycin, IV, 15 mg/kg/dose 6 hourly for 5–14 days.

AND

- Amikacin, IV, for 5–14 days if renal function is satisfactory.
  - o 1 week to < 10 years: 25 mg stat then 18 mg/kg once daily.
  - o 10 years and older: 20 mg stat then 15 mg/kg.

Tetanus prevention

Patients with no previous immunisation in the last 5 years:

- Tetanus toxoid, IM, 0.5 mL.

Complete course in previously unvaccinated patients.

Where deep necrotic lesions are part of the burn and if the immunological status is not known:

- Tetanus immunoglobulin, IM, 500 IU.

Prior to transport/referral

- Commence resuscitative measures, if necessary.
- Administer 100% humidified oxygen by facemask for inhalation injuries, if necessary.
- Cover wounds with clean dressings after hot or smouldering clothing have been removed.

REFERRAL

- Major burn injuries.

CHAPTER 2
ALIMENTARY TRACT

2.1 DENTAL AND ORAL DISORDERS

2.1.1 GINGIVITIS, UNCOMPLICATED

K05.1

DESCRIPTION

Inflammation of the gum margin causing the gums to separate from the teeth.
Pockets form between the gums and the teeth, where pus and bacteria can collect, eventually causing periodontitis, a disease in the tissue that surround and supports the teeth – See section 2.1.2: Periodontitis.
Characteristics of uncomplicated gingivitis:

- » change in the normal gum contour,
- » redness,
- » watery exudate/bleeding,
- » may be recurrent.
- » may be painful,
- » swollen gums,
- » gum recession may occur,

GENERAL AND SUPPORTIVE MEASURES

Oral hygiene is usually adequate to prevent superficial mouth and gum infection:

- » Oral hygiene after each meal to remove plaque and food debris.
- » Frequent thorough brushing of teeth, at least twice daily.
- » Dental flossing at least once a day.
- » Homemade warm saline rinse. Dissolve ½ teaspoon of table salt (sodium chloride) in ± 200 mL warm water. Rinse mouth for one minute twice daily but do not swallow.

MEDICINE TREATMENT

- Paracetamol, oral, 15 mg/kg/dose 6 hourly when required to a maximum of 4 doses per 24 hours.
- Chlorhexidine 0.2%, 15 mL as a mouthwash, 2–4 times daily for 5 days use after brushing and flossing.

2.1.2 PERIODONTITIS

K05.4

DESCRIPTION

Progressive gingivitis to the point where the underlying bone is eroded, is characterised by teeth becoming loose in their sockets.
It is a cause of tooth loss in adults.

GENERAL AND SUPPORTIVE MEASURES

» Advice on improving and maintaining oral hygiene. See section 2.1.1: Gingivitis, uncomplicated. General and supportive measures.

MEDICINE TREATMENT

- Chlorhexidine 0.2%, 15 mL as a mouthwash, 2–4 times daily for 5 days.

REFERRAL

» All cases.

2.1.3 NECROTISING PERIODONTITIS

K05.6

DESCRIPTION

An acute very painful infection of the gingival margin characterised by:

» foul smelling breath,
» loss of gingiva and supporting bone around teeth, and
» presence of underlying disease, e.g. HIV

May lead to loss of surrounding lips and cheeks if not adequately treated.

GENERAL AND SUPPORTIVE MEASURES

» Advice on improving and maintaining oral hygiene. See section 2.1.1: Gingivitis, uncomplicated. General and supportive measures.

MEDICINE TREATMENT

- Amoxicillin/clavulanic acid, oral, 25 mg/kg/dose of the amoxicillin component 8 hourly for 5 days.

- Chlorhexidine 0.2%, 15 mL as a mouthwash, 2–4 times daily 30 minutes after brushing and flossing.
    - o Continue for 5 days.

For pain:

- Paracetamol, oral, 15 mg/kg/dose 6 hourly when required to a maximum of 4 doses per 24 hours.

REFERRAL

For dental treatment:

» No improvement within 5 days.

2.1.4 CANDIDIASIS, ORAL

B37.0

See section 8.6: Candidiasis, systemic and other.

2.1.5 APHTHOUS ULCERS

K12.0

DESCRIPTION

Painful ulcers in the oropharynx. Minor ulcers (< 1 cm diameter) usually heal within 2 weeks. Major ulcers (> 1 cm diameter) are very painful, often very deep and persist.

GENERAL AND SUPPORTIVE MEASURES

» Maintain adequate nutrition and hydration by encouraging fluid and food intake – use bland foods and fluids as they are less painful.
» For minor aphthous ulcers, use homemade warm saline rinse. Dissolve ½ teaspoon of table salt (sodium chloride) in ± 200 mL warm water. Rinse mouth but do not swallow.

MEDICINE TREATMENT

For pain:

- Paracetamol, oral, 15 mg/kg/dose 6 hourly when required to a maximum of 4 doses per 24 hours.

REFERRAL

» Major aphthous ulcers for further diagnostic evaluation.
» Aphthous ulcers not resolving in 3 weeks for further evaluation.

2.1.6 HERPES GINGIVOSTOMATITIS

B00.2

DESCRIPTION

Inflammation of the mouth structures with ulcers (which may be of various numbers and sizes), caused by *Herpes simplex* virus infection. The normal course of the disease is 7–10 days.

DIAGNOSTIC CRITERIA

Clinical

» General inflammation of the mouth with multiple small ulcers on the buccal mucosa, palate, anterior tonsillar pillars, tongue, inner lips and gingival margins.
» Fever, malaise and dysphagia.
» Tender, enlarged cervical lymph nodes.

GENERAL AND SUPPORTIVE MEASURES

» Maintain adequate nutrition and hydration by encouraging fluid and food intake – use bland foods and fluids as they are less painful.
» If oral nutrition cannot be maintained use oral/nasogastric and/or IV fluids, if necessary.

MEDICINE TREATMENT

- Chlorhexidine 0.2%, 10 mL as a mouthwash or gargle, 12 hourly.
    - o Do not swallow.

For pain:

- Paracetamol, oral, 15 mg/kg/dose 6 hourly.

OR

- Ibuprofen, oral, 5–10 mg/kg/dose 6 hourly after meals.

If more than minor fever blisters:

- Aciclovir, oral, 250 mg/m^2/dose 8 hourly for 7 days (or per kg dose equivalent below)
    - o If > 1month to 1 year old: 12.5 mg/kg/dose.
    - o If > 1 year to 6 years old: 10 mg/kg/dose.
    - o If > 6 years to 12 years old: 6 mg/kg/dose.

If very severe infection, consider:

- Aciclovir, IV, 250 mg/m^2/dose 8 hourly for 7 days (per kg dose equivalent below)
    - o If > 1 month to 1 year old: 12.5 mg/kg/dose.
    - o If > 1 year to 6 years old: 10 mg/kg/dose.
    - o If > 6 years to 12 years old 6 mg/kg/dose.
      Change to oral as soon as possible.

For very painful oral herpes in children > 2 years:

- Lidocaine (lignocaine) 2% gel applied every 3 to 4 hours.
    - o Apply a thin layer on the affected areas only.
    - o Do not exceed 3 mg/kg dose, i.e. maximum 0.15 mL/kg of 2% gel.

REFERRAL

» Herpes gingivostomatitis not responding to therapy.
» Disseminating disease, especially if associated with encephalopathy or increasing liver span.

2.2 GASTROINTESTINAL DISORDERS

2.2.1 CHOLERA

A00.9

***** Notifiable condition.

DEFINITION

An acute diarrhoeal disease caused by *V. cholerae*.

DIAGNOSTIC CRITERIA

Clinical

- » Sudden onset of severe, watery diarrhoea, i.e. 'rice water' diarrhoea.
- » Low-grade or no fever.
- » Persistent vomiting not associated with nausea.
- » Rapid fluid and electrolyte losses with dehydration, acidosis and hypovolaemic shock with/without renal failure.
- » History of contact with a cholera case or the presence of cholera in the community.

Investigations

- » Positive stool culture.
- » Agglutinating or toxin-neutralising antibodies in the serum.

GENERAL AND SUPPORTIVE MEASURES

- » Isolate patient and institute barrier nursing.
- » Ensure adequate hydration and nutrition.
- » Check blood glucose in patients with decreased level of consciousness.

The management of the fluid requirements is the most critical element of treating a patient with cholera.

MEDICINE TREATMENT

First treat shock.

Once shock has resolved, manage as acute diarrhoea. See section 2.2.4 Diarrhoea, acute.

For the management of shock during recognised cholera outbreaks, there may be benefit to replace sodium chloride 0.9% with:

- Modified Ringers–Lactate, IV.

Antibiotic treatment

Recommended antibiotics may vary according to sensitivities in epidemics. Consult the NICD for the latest recommendations. Current recommendations for severe dehydration are:

- Ciprofloxacin, oral, 15 mg/kg/dose 12 hourly for 3 days.

<u>In all children who are able to take oral medication</u>

- Zinc (elemental), oral for 14 days:
  - o If < 10 kg: 10 mg/day.
  - o If > 10 kg: 20 mg/day.

REFERRAL

» Cholera with complications, e.g. persistent shock, renal failure and severe electrolyte disturbances.

2.2.2 CONSTIPATION / FAECAL LOADING

K59.0

DESCRIPTION

Constipation: the infrequent passage of hard stools. This is often due to behavioural retention following previous painful episodes of defaecation, but may also be due to somatic causes or overuse of certain medications.

Faecal soiling: the involuntary leakage of small amounts of soft or watery stools secondary to faecal loading.

Causes include:

» psychogenic disorders,
» incorrect diet,
» lack of exercise,
» chronic use of enemas,
» certain medicines,
» metabolic, endocrine, neurogenic and lower bowel abnormalities.

DIAGNOSTIC CRITERIA

» Non-tender deformable faecal masses palpable.
» Confirm on a straight abdominal X-ray.

GENERAL AND SUPPORTIVE MEASURES

» Determine and treat the underlying cause.
» Treatment involves 3 steps:
  > initial clearance of stools,
  > prevent re-accumulation of hardened retained stool, and
  > retraining of the gut to achieve regular toilet habits.
» Management is long-term and requires the active involvement of the parents.

MEDICINE TREATMENT

Initial therapy

Faecal clearance if faecal loading:

- Phosphate-containing enema (sodium phosphate 6 mg/mL/ sodium acid phosphate 16 mg/mL).
  - o Age 2–5 years: 32 mL.
  - o Age 5–11 years: 64 mL.
  - o Repeat once, if necessary.

OR

Add-on therapy after failed treatment with phosphate enema:

- Polyethylene glycol 59 g/L solution with sodium sulphate and electrolytes , oral/ nasogastric tube, 10–25 mL/kg/hour until clear fluid is passed rectally.

Do not use sweeteners containing sugar.
Confirm the bowel is empty.

Maintenance therapy

Bowel re-training

Diet change with additional natural fibre from fruit, vegetables and bran.

Additional fibre:

- Ispaghula husk, oral, 1.75–3.5 g, stirred in water with breakfast.

AND/OR

- Liquid paraffin, oral, 2 mL/kg/day.

In refractory cases:

- Lactulose, oral, 0.5 mL/kg/dose 12 hourly.

If faecal loading, maintenance therapy should be continued for months to years.

REFERRAL

» Suspected organic cause e.g. constipation from birth in a breast-fed baby.

» Inadequate response to therapy.

2.2.3 CYSTIC FIBROSIS

E84.9

DESCRIPTION

An autosomal recessive disorder of exocrine glands, mainly affecting the gut, pancreas and lungs.

DIAGNOSTIC CRITERIA

Clinical

- » Recurrent infections of the respiratory tract with later bronchiectasis, respiratory failure and cor pulmonale.
- » Bulky, greasy and foul-smelling stools.
- » Occasionally presents with constipation.
- » Malabsorption with weight loss and failure to thrive.
- » Meconium ileus.
- » Family history, rare unless in a sibling.

Investigations

- » Sweat test:
  - > Quantitative analysis of sodium and chloride concentrations in sweat collected after stimulation by pilocarpine iontophoresis with chloride > 60 mmol/L.
- » DNA analysis for delta F508 and a few other mutations.

GENERAL AND SUPPORTIVE MEASURES

- » Nutritional support:
  - > well balanced diet,
  - > oral intake of at least 120% of recommended daily allowance,
  - > nutritional supplements,
  - > occasionally nocturnal supplemental feeding by nasogastric or gastrostomy tube.
- » Physiotherapy and postural drainage.
- » Psychosocial support.
- » Genetic counselling.

MEDICINE TREATMENT

Medicinal treatment is specialised and individualised and should be under the supervision of a subspecialist.

- Pancreatic enzymes (lipase/amylase/protease), with meals according to clinical response.
  - o Maximum dose 10 000 lipase units/kg/day.

REFERRAL

- » All to a recognised cystic fibrosis centre and/or specialist health facility for confirmation of diagnosis and initiation of treatment.
- » Management of exacerbations.

2.2.4 DIARRHOEA, ACUTE

A09.0

DESCRIPTION

Diarrhoea is a serious common childhood illness evidenced by the passing of frequent profuse loose watery stools. Vomiting may or may not be present.
Diarrhoeal disease is often caused by viral infection but may be due to bacterial infection, dietary or other causes.
Dehydration and metabolic disturbances are common if treatment is not instituted early and may result in severe disease, irreversible organ damage and death in children.
Malnutrition is a serious co-morbidity and/or result of diarrhoeal disease and must be managed correctly employing ongoing feeding. Feeding, minerals, micronutrients and vitamins are continued except during ileus or shock. See section 2.4: Malnutrition.
In severe malnutrition or in the young infant (< 2 months of age) bacterial co-infection is common.

DIAGNOSTIC CRITERIA

Clinical

> The assessment of shock and dehydration in children is not always simple. A good initial assessment and frequent re-assessments (4-hourly if dehydration is present. In the presence of shock continuous reassessment with appropriate adjustment of care are vital in the care of these children.

Shock is shown by one or more of the following:
Compensated shock:
- » delayed capillary refilling time (> 3 seconds);
- » rapid, weak pulse rate;
- » cool peripheries.

Late (Preterminal):
- » decreased level of consciousness,
- » decreased blood pressure,
- » decreased pulse volume.

Dehydration **is treated <u>after</u> shock is dealt with:**

**Severe dehydration**	**Some dehydration**
Sunken eyes<br>Very slow skin pinch (≥ 2 sec)<br>Drinking poorly	Sunken eyes<br>Slow skin pinch (< 2 sec)<br>Drinks eagerly<br>Irritable/restless

Other indicators of dehydration may be sought but do not add substantially to assessment, e.g.: depressed fontanelle, absent tears, decreased passage of urine.

Also assess for signs of metabolic, nutritional and other co-morbidities:

- » severe malnutrition,
- » decreased level of consciousness,
- » abnormal tone or floppiness,
- » abdominal distension,
- » decreased bowel sounds,
- » increased respiratory rate and chest indrawing,
- » persistent or bile stained vomiting,
- » urine for leucocytes or nitrites.

Investigations

- » After resuscitation, in children with severe dehydration, shock or other signs of metabolic, nutritional or other co-morbidities:
  - > sodium, potassium, urea, creatinine, blood acid-base assessment.
- » Stool culture, especially if at a sentinel site for infectious GIT disease, or suspected dysentery, typhoid, cholera.
- » Urine test strip on fresh/clean urine specimen for leucocytes, nitrites and blood. Ascertain HIV status with consent in every child.

GENERAL AND SUPPORTIVE MEASURES

- » Adequate initial assessment and fre quent re-assessment, including weight, is vital.
- » Re-assess the patient continuously while shock persists.
- » If dehydration is present, re-assess the patient 4-hourly and immediately correct shock or deterioration.
- » Monitor and maintain:
  - > hydration and circulation,
  - > blood pressure,
  - > acid-base status.
  - > normal blood glucose,
  - > blood electrolytes,
- » Monitor urine output, should be at least 1 mL/kg/hour. This may be difficult in small children with diarrhoea, especially in female infants.
- » Monitor body mass regularly. Weigh daily, 6-hourly if unsure of hydration status and child is very ill or sm all. This can be used to indicate response of hydration.
- » Continue oral feeds during period of diarrhoea:
  - > if the child is breastfed, continue breastfeeds and encourage the child to feed longer at each feed;
  - > if the child is exclusively breastfed, give oral rehydration solution (ORS) in addition to each feed;
  - > if the child is not exclusively breastfed, give ORS and other appropriate feeds, e.g. breast milk substitutes or food based fluids;
  - > if the child is severely dehydrated or shocked, withhold feeding until stable, usually a few hours only.

MEDICINE TREATMENT

> There is no place for antidiarrhoeal medications, i.e. kaolin and pectin, atropine and diphenoxylate, loperamide, or antiemetics in the routine management of acute diarrhoea.

OUTLINE OF PRACTICAL FLUID THERAPY OF DEHYDRATING WATERY DIARRHOEA

With severe malnutrition the assessment of dehydration is more difficult. Avoid intravenous infusions, if possible.
Treatment of dehydration requires more care/more frequent assessments.

1. First treat shock, if present (If no shock, proceed to section 2 below)

> If an IV infusion cannot be set up within 5 minutes use an intra-osseus infusion. See section 1.1.8: Intra-Osseous Infusion in Emergencies. During treatment of shock administer oxygen.

- Sodium chloride 0.9%, IV, 20 mL/kg given as a bolus rapidly.
  - After each bolus reassess for persistence of shock, or evidence of circulatory overload.
  - Repeat the fluid bolus up to 3 times if shock still persists, provided that evidence of circulatory overload is not present.
  - If after the second bolus, i.e. total of 40 mL/kg has been given, the response is inadequate, a third bolus can be started. Move the patient to ICU for CVP monitoring and inotropic support.

<u>Treatment of shock in severe malnutrition</u>
Shock treatment should be more cautious in patients with severe malnutrition due to poor cardiac reserve and high prevalence of gram negative septicaemia.

- Sodium chloride 0.9%, IV, 10 mL/kg administered over 10 minutes.
  - Up to 4 boluses may be given. However, deterioration may be due to fluid overload and shock may be due to septicaemia, not always hypovolaemia.
  - After 4 boluses (40 mL/kg) further treatment should be in a high care unit.
  - Re-assess frequently during treatment of shock. Patients response should guide further fluid therapy.

If pulse and respiratory rate increases, increasing liver span and gallop rhythm are found suspect fluid overload/ cardiac dysfunction and manage appropriately. See section 1.1.7: Shock.

When shock has been treated proceed to the management of dehydration.

2. Severe dehydration or some dehydration

2a) If the child has not failed oral rehydration and was not in shock:

- Oral rehydration solution (ORS), oral, 80 mL/kg over 4 hours using frequent small sips (i.e. 5 mL/kg every 15 minutes for 4 hours).
  - Give more if the child wants more.
  - Show the caregiver how to give ORS with a cup and spoon.
  - If child vomits wait 10 minutes and then continue more slowly.
  - Encourage caregiver to continue feeding the child, especially breastfeeding.

Review after 4 hours:

- » general condition,
- » capillary filling time,
- » level of consciousness,
- » skin turgor,
- » sunken eyes.
- » respiratory rate,
- » abdomen (liver span),
- » if passing urine,
- » number/quality of stools, and

Appropriate response at 4 hourly re-assessment:

Shock	• Treat for shock as under 1 above.
No improvement or more dehydrated	• Increase drip rate by 25%.
Improving (e.g. increase in weight) but still dehydrated	• Continue current drip rate.
No visible dehydration	• Decrease drip rate by 50%.<br>• If remains well hydrated after a further 4 hours stop IV rehydration fluids and move to ORS.<br>• For prevention of dehydration see under 3 below.

2b) If child fails the above oral treatment, was in shock or has already failed at primary health care level then:

IV fluid*

- ½ Darrows/dextrose 5%, IV, 10 mL/kg/hour administered for 4 hours, then re-assess.

*(This rate is in line with current safety evidence but the need for regular reassessment 4-hourly remains).

PLUS

Oral rehydration solution

- Oral rehydration solution (ORS), oral, 80 mL/kg over 4 hours using frequent small sips (i.e. 5 mL/kg every 15 minutes for 4 hours).

PLUS

Oral feeds at normal feed volumes and times.

Review after 4 hours:

» general condition,
» capillary filling time,
» level of consciousness,
» skin turgor,
» sunken eyes.
» respiratory rate,
» abdomen (liver span),
» if passing urine,
» number/quality of stools, and

Appropriate response at 4-hourly re-assessment:

Shock	• Treat for shock as under 1 above.
No improvement or more dehydrated	• Increase drip rate by 25%.
Improving (e.g. increase in weight) but still dehydrated	• Continue current drip rate.
No visible dehydration	• Decrease drip rate by 50%.<br>• If remains well hydrated after a further 4 hours stop IV rehydration fluids and move to ORS.<br>• For prevention of dehydration see under 3 below.

Give oral feeds if:

» the level of consciousness is normal,
» the child is not in severe distress,
» not shocked and,
» has no surgical abdomen.

3. No visible signs of dehydration on presentation or a child stable with no dehydration after treatment of dehydration.

Show the caregiver how to give ORS with a cup and spoon using frequent small sips.

Encourage caregiver to give 10 mL/kg after each diarrhoeal stool until diarrhoea stops.

Instruct the caregiver on how to make and use ORS/SSS at home.

Home made sugar and salt solution may be used if oral rehydration formula is not available.

HOMEMADE SUGAR AND SALT SOLUTION (SSS)
½ level medicine measure of table salt
plus
8 level medicine measures of sugar
dissolved in 1 litre of boiled (if possible) then cooled water
(1 level medicine measure = approximately 1 level 5 mL teaspoon)

Encourage the caregiver to continue feeding the child, especially breastfeeding.

Instruct the caregiver to give the child extra feeds after the diarrhoea has stopped to make up for the period of inadequate intake.

Child should return to hospital immediately if:

- » no improvement,
- » condition deteriorates,
- » poor drinking or feeding,
- » slow skin pinch.
- » blood in stool,
- » fever develops,
- » sunken eyes,

Educate caregivers about hygiene, oral rehydration solution and danger signs of diarrhoea.

Figure 2 Summary flow chart for correction of dehydration in diarrhoeal disease

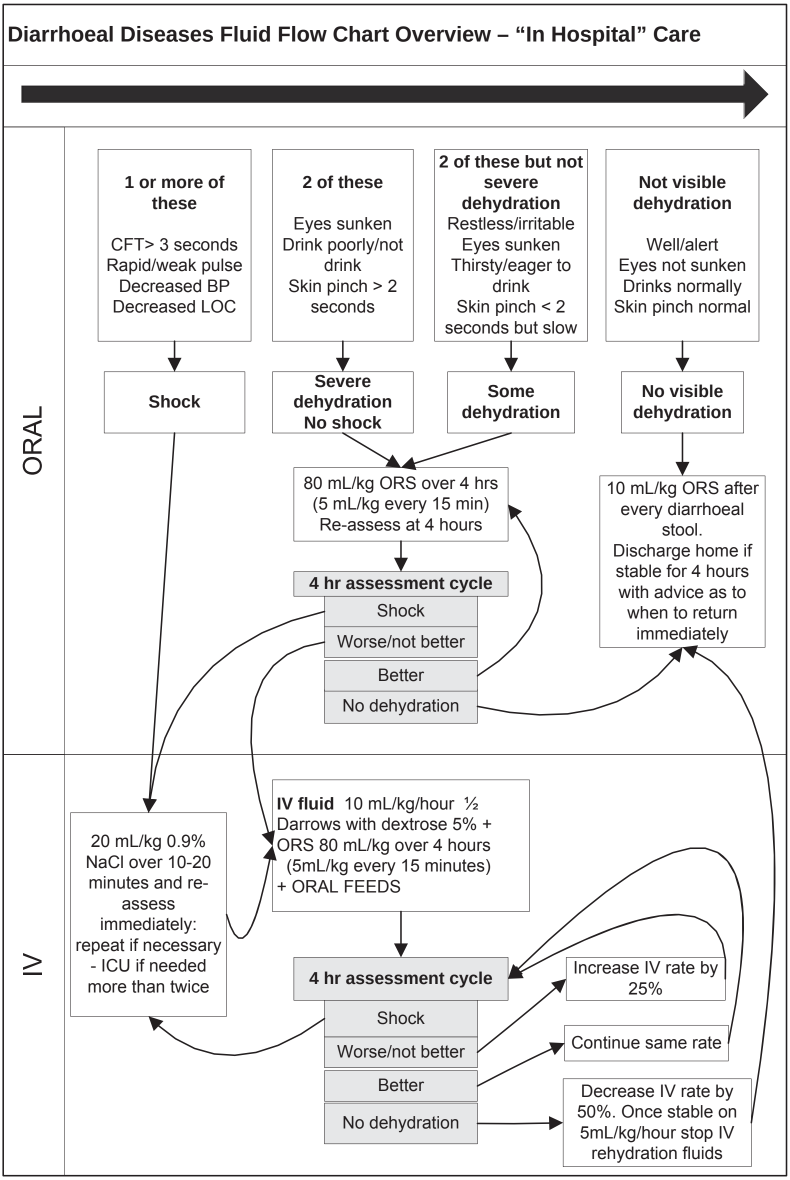

Metabolic disturbances

Acidosis

Metabolic acidosis does not require correction unless extremely severe, i.e. pH < 7.1, or if the body is unable to correct the deficit, e.g. salicylate poisoning or renal failure.

Correction should only be considered with expert supervision.

Correcting the renal circulation and shock will lead to self-correction in almost all cases.

If correction is necessary: volume of sodium bicarbonate 4.2% required is:

- Sodium bicarbonate 4.2% in mL = 0.3 x base deficit x weight in kg. Review response to assess the need for further correction.

Hypokalaemia

Note: Potassium levels are affected by the degree of acidosis.

If potassium is < 3.5 mmol/L but > 2.5 mmol/L:

- Potassium chloride, oral, 25–50 mg/kg/dose 8 hourly.

If potassium is < 2.5 mmol/L:

- ½ Darrows/dextrose 5%, 200 mL **plus** potassium chloride 15%, 2 mL, into the vacoliter:
  - o 1 mL potassium chloride 15% = 2 mmol in the above dilution, gives combined K^+ of 37 meq/L – do not exceed this amount.
  - o Mix well before administration.
  - o Run at normal rehydration rate (as above).

Oral potassium may also be given during this period:

- Potassium chloride, oral, 25–50 mg/kg/dose 8 hourly.

Monitor serum potassium 8–12 hourly. Once above 3.0 meq/L, stop IV potassium and continue with oral.

Hypernatraemia (> 150 mmol/L)

Oral rehydration is preferable to IV rehydration.

If oral rehydration fails, rehydrate using IV over 48 hours but continue giving oral rehydration.

IV Fluid rate

Rate:

- o If 2–10 kg: 6 mL/kg/hour
- o If > 10–20 kg: 5 mL/kg/hour
- o If > 20–40 kg: 4 mL/kg/hour

Serum sodium ≤ 160 mmol/L

- ½ Darrows/dextrose 5%, IV.

Serum sodium > 160 mmoL

Sodium chloride 0.9%/dextrose 5% plus potassium chloride (see below) is used to correct clinical dehydration for the first 48 hours. After changing to maintenance rates use oral rehydration, or if IV fluids are required change to ½ Darrows/dextrose 5%.

- Sodium chloride 0.9%/dextrose 5% **plus** potassium chloride (to 20 mmol/L), IV.

Repeat serum sodium every 8–12 hours to monitor progress.
Failure to decrease sodium levels usually means the rehydration rate is too slow.
Fall of sodium levels more than 1 mmol/L/hour on average means the rehydration rate should be reduced.

Frequent clinical reassessment is the key to the safe management of this situation. Serum sodium levels may be done more frequently where this is possible. Adjust the drip rate according to response.

If convulsions are considered likely, (decreased level of consciousness, hyper-irritable child), in the setting of high serum sodium, consider the use of prophylactic anticonvulsants:

- Phenobarbitone, IV, 20 mg/kg as a single dose.

OR

If IV phenobarbitone not available:

- Phenobarbitone, oral, 20–30 mg/kg as a single dose.

Hyponatraemia

The correction of hyponatraemia is usually only necessary where the serum sodium is significantly decreased (i.e. < 120 mmol/L), or if the patient is symptomatic.

Use sodium chloride 0.9% and add potassium chloride and dextrose as indicated below.
Give at the rate in dicated for dehydration and ex pect correction to hav e occurred after the following estimated volume:

Volume of sodium chloride 0.9% (mL) = (130–Na^+) x body weight in kg x 4.

- Administer sodium chloride 0.9%, 200 mL **plus** potassium chloride 15%, 2 mL **plus** dextrose 50%, 20 mL into the vacoliter.
  - Mix well before administration.

After the calculated volume has been given, resume with:

- ½ Darrows/dextrose 5%, IV, at the required rate.
  - Recheck the serum electrolytes.

Antibiotic therapy

Note:

» Antibiotics are not routinely used for diarrhoeal disease.
» During diarrhoea, absorption of antibiotics may be impaired due to intestinal hurry. Give antibiotics orally if administered for intra-luminal effect.
» Other antibiotics for systemic action are best administered parenterally.
» Consider urinary tract infectio n, or septicaemia in children with severe malnutrition, the immunocompromised and infants < 2 months old.

Dysentery

Treat initially as shigella dysentery:

- Ceftriaxone, IV, 50 mg/kg as a single daily dose for 5 days.

OR

- Ciprofloxacin, oral, 15 mg/kg/dose 12 hourly for 3 days.

CAUTION: USE OF CEFTRIAXONE

After 28 days of age IV ceftriaxone and IV calcium containing fluids may be given but only sequentially with the giving set flushed well between the two products.

Note: Do not use ceftriaxone in neonates.

For entamoeba histolytica (if demonstrated on stool microscopy, or strongly suspected - this is now a relatively uncommon condition in children in South Africa).

- Metronidazole, oral, 15 mg/kg/dose 8 hourly for 7 days.
  - o Severe disease: treat for 10 days.

Cholera

Treat according to current sensitivities of the organism during epidemic.
See section 2.2.1: Cholera.

Typhoid

- Ceftriaxone, IV, 50 mg/kg once daily for 10–14 days.

CAUTION: USE OF CEFTRIAXONE

After 28 days of age IV ceftriaxone and IV calcium containing fluids may be given but only sequentially with the giving set flushed well between the two products.

Note: Do not use ceftriaxone in neonates.

Severe malnutrition
See section 2.4.1: Malnutrition, severe acute.

- Ampicillin, IV, 50 mg/kg/dose 6 hourly for 5 days.

PLUS

- Gentamicin, IV, 6 mg/kg as a single daily dose for 5 days.
  - o Confirm normal renal function before second dose.

Very young infants , 28 days old

- Ampicillin, IV, 25–50 mg/kg/dose 6 hourly for 5 days.

PLUS

- Gentamicin, IV, 6 mg/kg as a single daily dose for 5 days.
  - o Confirm normal renal function before second dose.

Mineral and micronutrient supplementation
All children with diarrhoea.

- Zinc (elemental), oral,
  - o If < 10 kg: 10 mg/day.
  - o If > 10 kg: 20 mg/day.

- Potassium chloride, oral, 8 hourly.
  - o If < 6 months: 125 mg.
  - o If > 6 months: 250 mg.

  Unless: hyperkalaemic or anuric.

REFERRAL

» Inability to correct/treat shock/dehydration.
» Metabolic complications.
» Unresolving diarrhoea.

2.2.5 DIARRHOEA, PERSISTENT

K52.9

DESCRIPTION

Persistent diarrhoea: diarrhoea for longer than two weeks.

Persistent diarrhoea results in significant morbidity and mortality associated with poor nutrition.

Persistent diarrhoea is most frequently due to:

» temporary loss of disaccharidase activity in the intestinal microvillous brush border, e.g. lactase loss; or
» luminal infection/infestation, which may be non-specific bacterial overgrowth.

Rare causes include food allergies, cystic fibrosis and coeliac disease.

DIAGNOSTIC CRITERIA

Clinical

- » Persistent diarrhoea without weight loss or dehydration – consider Toddler's diarrhoea.
- » Persistent diarrhoea with weight loss and dehydration – consider small bowel mucosal injury, e.g. lactose intolerance or small bowel bacterial overgrowth.
- » Persistent diarrhoea with weight loss but no dehydration – consider a malabsorption syndrome, e.g. coeliac disease, allergic enteropathy, cystic fibrosis, etc.
- » Consider the possibility of HIV infection.

Investigations

Where weight gain falters, dehydration recurs, the child is ill or the diarrhoea continues:

- » full blood count,
- » serum proteins,
- » urine and stool microscopy,
- » culture and sensitivity tests (MCS),
- » stool-reducing substances > 0.5% reducing sugar is abnormal if on a lactose-containing diet.

GENERAL AND SUPPORTIVE MEASURES

Treatment strategy includes a stepwise approach with modification of the diet, which are not mutually exclusive and are applied according to local resources.

- » Monitor hydration, stools, nutritional status, weight gain, growth and other nutritional parameters such as serum proteins.
- » Nutritional support:
  - > Aim to provide at least 110 kcal/kg/day orally within three days to protect nutritional state.
  - > Where the stepwise approach is not possible:

    Under 4 months:

    Encourage exclusive breastfeeding if lactose intolerance is not severe. If not exclusive breastfeeding, use breast milk substitutes that are low in lactose, e.g. yoghurt or amasi or specialised formulae or lactose-free milk formula.

    Children aged 4 months and older:

    Feeding should be restarted as soon as the child can eat, with small meals 6 times a day.
  - > Nasogastric feeding may be required in children who eat poorly.

    If the response is good, give additional fruit and well-cooked vegetables to children who are responding well.

    After 7 days of treatment with an effective diet, resume an appropriate diet for age, including milk, which provides at least 110 calories/kg/day.

    Follow up regularly to ensure recovery from diarrhoea, continued weight gain and adherence to feeding advice.

MEDICINE TREATMENT

> **CAUTION**
> Antidiarrhoeal and anti-emetic agents are NOT recommended.

Antibiotic therapy
Antibiotics are only indicated when specific infections are suspected or where they are used in the Step-Wise Drug Based Empiric Protocol for Management of Diarrhoea.

All persistent diarrhoea with blood in stool should be treated as dysentery. See section 2.2.6: Dysentery.

For campylobacter:
- Erythromycin, oral, 10 mg/kg/dose 6 hourly for 7 days.

For *G. lamblia*:
- Metronidazole, oral, 7.5 mg/kg/dose 8 hourly for 5–7 days.

For *Y. enterocolitica*:
- Ceftriaxone, IV, 50 mg/kg/dose once daily.

OR
- Cefotaxime, IV, 50 mg/kg/dose 6 hourly.

> **CAUTION: USE OF CEFTRIAXONE**
> After 28 days of age IV ceftriaxone and IV calcium containing fluids may be given but only sequentially with the giving set flushed well between the two products.
>
> **Note: Do not use ceftriaxone in neonates.**

For *Cryptosporidium*:
No effective treatment available in the presence of HIV related immunosuppression.

For *Isospora belli*:
- Co-trimoxazole, oral, 5 mg/kg/dose of trimethoprim component 6 hourly for 10 days then 12 hourly for 3 weeks.

For *Cyclospora cayetanensis:*
- Co-trimoxazole, oral, 5 mg/kg/dose of trimethoprim component 6 hourly for 5 days.

For *Microsporidia*:
- Albendazole, oral. (Specialist supervision)

STEP-WISE EMPIRIC PROTOCOL FOR MANAGEMENT OF DIARRHOEA

Commence management at the most appropriate step according to previous management – many infants with persistent diarrhoea will already have failed the “day 1-2” stage and will commence management on “day 3-5”.

Day 0

Rehydration: Recommence breast or full-strength formula feeds within 12–24 hours.
Additional oral rehydration solution (ORS) to maintain hydration.

Day 1–2

Continue full-strength feeds with additional ORS as required.

Day 3–5

Change to lactose-free feeds if not breastfed.
Continue additional fluids as required.
If diarrhoea resolves, discharge, but continue with lactose-free feeds for 2 weeks.

Day 6–8

- Gentamicin, **ORAL**, 8 mg/kg/dose 4 hourly for 3 days only. Specialist initiated.
- Consider change to lactose-free feeds if not breastfed.

Day 9–11

Semi-elemental formula, sucrose- and lactose-free, protein hydrolysate, medium chain triglyceride.
Continue additional fluids as required.
If diarrhoea resolves, discharge if possible on semi-elemental feeds for at least 2 weeks. If this is not possible a trial of lactose free feeds before discharge is sometimes successful and, if so, the child can be discharged on the lactose free feeds.

If giardia is not excluded:

- Metronidazole, oral, 7.5 mg/kg/dose 8 hourly for 5 days.

In HIV infected children: *Isospora belli* and *Cyclospora*:

- Co-trimoxazole, oral, 5mg/kg/dose of trimethoprim 12 hourly for 10 days.

Day 14+

Consider total parenteral nutrition until diarrhoea has stopped. Thereafter gradually reintroduce semi-elemental feeds.

After success as indicated by weight gain, return of appetite and decrease of diarrhoea, less elemental diets can be judiciously and slowly re-introduced.

Mineral and micronutrient deficiencies

- Zinc (elemental), oral,
  - o If < 10 kg: 10 mg/day.
  - o If > 10 kg: 20 mg/day.

Provide nutritional support.

REFERRAL

» Inability to maintain hydration.
» Seriously compromised nutrition.
» Lack of local resources to support the stepwise protocol at any step.
» All cases not responding by day 12–13 of the stepwise protocol.
» Recurrent diarrhoea.

2.2.6 DYSENTERY

A03.9

DESCRIPTION

Passage of blood and mucus in the stools.
Shigella infection is the most common serious cause in children in South Africa.

Complications include:

» dehydration,
» shock,
» acidosis,
» renal failure, and
» convulsions,
» toxic megacolon,
» rectal prolapse,
» haemolytic uraemic syndrome.

DIAGNOSTIC CRITERIA

Clinical

» Sudden onset.
» Abdominal cramps, peritonism, urgency, fever and diarrhoea with blood and mucus in the stools.
» Meningismus and convulsions may occur.
» Exclude intussusception. Evidence of intussusception includes:
  > pain or abdominal tenderness,
  > bile-stained vomitus,
  > red currant jelly-like mucus in stool,
  > appearance of the intussusceptum through the anus.

Investigations

» Stool culture to confirm diagnosis of Shigellosis.
» Polymorphs and blood on stool microscopy.
» Immediate microscopy of warm stool to diagnose amoebic dysentery.

GENERAL AND SUPPORTIVE MEASURES

» Monitor fluid and electrolyte balance.
» Ensure adequate nutrition and hydration.

MEDICINE TREATMENT

Fluid and electrolyte replacement

See section 2.2.4: Diarrhoea, acute.

Antibiotic therapy

Treat as Shigella during an epidemic of Shigellosis, or if the child is febrile, "toxic"-looking, has seizures or if Shigella is cultured from the stool and the child is still ill.

- Ciprofloxacin, oral, 15 mg/kg/dose 12 hourly for 3 days.

Where oral medication cannot be used:

- Cefotaxime, IV, 75 mg/kg/dose 8 hourly for 5 days.

OR

- Ceftriaxone, IV, 50 mg/kg as a single daily dose for 5 days.

CAUTION: USE OF CEFTRIAXONE

After 28 days of age IV ceftriaxone and IV calcium containing fluids may be given but only sequentially with the giving set flushed well between the two products.

Note: Do not use ceftriaxone in neonates.

For entamoeba histolytica (only if demonstrated on stool microscopy, or strongly suspected):

- Metronidazole, oral, 15 mg/kg/dose 8 hourly for 7 days.

REFERRAL

» Dysentery with complications, e.g. persistent shock, haemolytic uraemic syndrome and toxic megacolon.

2.2.7 GASTRO-OESOPHAGEAL REFLUX DISEASE (GORD)

K21

DESCRIPTION

Gastro-oesophageal reflux is rep etitive regurgitation/reflux of gastri c contents into the oesophagus.

It is termed “Uncomplicated GOR” if the only symptom is frequent small vomits, in which case no further investigation or treatment is needed.
It is termed “Complicated GOR” or “GORD” if associated with the diagnostic criteria below.

DIAGNOSTIC CRITERIA

Clinical

» Recurrent vomiting or regurgitation and any of the following:
  > respiratory symptoms, i.e. recurrent wheeze or cough, chronic obstructive airway disease, recurrent aspiration/pneumonia, stridor, apnoea and apparent life-threatening event;
  > failure to thrive; and
  > abnormal posturing or opisthotonus (Sandifer syndrome).

Investigations

» 24-hour oesophageal pH monitoring is the most accurate method of assessing significant reflux. A negative test does not totally exclude reflux.
» Endoscopy to confirm oesophagitis may be carried out.
» Barium swallow – easy and accessible, but not very sensitive.
» Isotope studies ‘milk scan’ – oesophageal and gastric scintiscanning.

GENERAL AND SUPPORTIVE MEASURES

» Postural treatment: lying on the left side is currently recommended.
» Dietary measures such as feed thickeners. If not breastfeeding, frequent small volume feeds and early introduction of solids.

MEDICINE TREATMENT

Note:
Evidence in support of the following recommendations is weak:

- Omeprazole, oral, 0.4–0.8 mg/kg/dose 12 hourly. Specialist initiated.
  - o Maximum dose: 20–40 mg/dose.

If 1 month–2 years:	2.5 mg 12 hourly.
If > 2–6 years:	5 mg 12 hourly.
If > 7–12 years:	10 mg 12 hourly.

REFERRAL

» For diagnostic investigations, if not available locally.
» GORD not responding to treatment.

2.2.8 PEPTIC ULCER DISEASE

K27

DESCRIPTION

Varying degrees of gastritis or frank ulceration of the stomach or duodenum due to acid and pepsin-laden stomach contents on the gastric and duodenal mucosa in the face of inability of mucosal defence mechanisms to prevent these effects.

Peptic ulcers may be primary or secondary, e.g. stress related or associated with NSAID use.

DIAGNOSTIC CRITERIA

Clinical

» Haematemesis or melaena is a relatively common presentation in children (up to 50%).
» Epigastric pain. Pain is often poorly localised in children, described as dull and aching and frequently does not appear to respond to antacids.

Investigations

» Endoscopy to confirm diagnosis.

GENERAL AND SUPPORTIVE MEASURES

» Manage circulation and anaemia, as required.
» Stop all non steroidal anti-inflammatory agents.
» Remove all stressors identified.

MEDICINE TREATMENT

■ Proton pump inhibitor, e.g.:

- Omeprazole, oral, 0.4–0.8 mg/kg/dose 12 hourly. Specialist initiated.
  - o Maximum dose: 20–40 mg/dose.

If 1 month–2 years:	2.5 mg 12 hourly.
If > 2–6 years:	5 mg 12 hourly.
If > 7–12 years:	10 mg 12 hourly.

PLUS

If *Helicobacter pylori* positive. (Not routine)

- Amoxicillin, oral, 25–30 mg/kg 12 hourly for 7 days.

PLUS

- Metronidazole, oral, 7.5 mg/kg 12 hourly for 7 days.

REFERRAL

» Poor response to treatment.
» Suspicion of underlying cause.

2.2.9 INFLAMMATORY BOWEL DISEASES (IBD)
K58

DESCRIPTION
Chronic incurable inflammatory diseases of the intestine that are of unknown aetiology. IBD is sub-classified into:
» ulcerative colitis,
» Crohn's disease, and
» indeterminate colitis.

DIAGNOSTIC CRITERIA
Clinical
» Ulcerative colitis:
  > abdominal pain,
  > chronic diarrhoea with blood in stools,
  > urgency and tenesmus,
  > fever,
  > weight loss, and
  > arthritis/arthralgia.
» Crohn's disease:
  > postprandial pain,
  > diarrhoea,
  > weight loss,
  > fever,
  > abscess,
  > perioral disease,
  > uveitis/conjunctivitis,
  > arthralgia/arthritis, and
  > entero-enteric/enterocutaneous fistulae.

Investigations
» Blood tests may show moderate anaemia, leucocytosis, raised ESR and decreased serum proteins.
» Stool microscopy and culture, including mycobacteria.
» Ulcerative colitis: colonoscopy and biopsy.
» Crohn's disease: barium studies.

GENERAL AND SUPPORTIVE MEASURES
» Enteral nutrition to achieve optimal growth.
» Elemental enteral or parenteral nutrition may be required in some patients under sub-specialist supervision.

REFERRAL
» All patients with suspected inflammatory bowel disease for assessment and initiation of therapy.

2.3 HEPATIC DISORDERS

2.3.1 BLEEDING OESOPHAGEAL VARICES

I85.0

DESCRIPTION

Presentation with haematemesis (fresh blood) or malaena in a patient who has a spontaneous bleed from varices at the oesophageal-gastric junction. The patient may or may not have been known to have chronic liver disease and portal hypertension. This bleeding may be hard to control and be life threatening.

GENERAL AND SUPPORTIVE MEASURES

» Resuscitation and blood transfusion as required.
» For local control of acute bleeds that are not controlled with medicine treatment: Sengstaken tube.
» For secondary prophylaxis after a bleed: consider endoscopic injection sclerotherapy or variceal banding every 2 weeks until eradicated.
» If either or both treatments fail: surgical over-sewing.

MEDICINE TREATMENT

- Octreotide, IV, bolus, 1–2 mcg then 1–5 mcg/kg/hour by infusion.
  (Specialist initiated)

Post bleed prophylactic management

■ Proton pump inhibitor, e.g.:

- Omeprazole, oral, 0.4–0.8 mg/kg/dose 12 hourly. Specialist initiated.
  - o Maximum dose: 20–40 mg/dose.

If 1 month–2 years:	2.5 mg 12 hourly.
If > 2–6 years:	5 mg 12 hourly.
If > 7–12 years:	10 mg 12 hourly.

AND

- Propranolol, oral, 2 mg/kg/24 hours in 3 divided doses.
  - o If needed, increase dose to 8 mg/kg/24 hours.
  - o Aim to reduce the resting pulse rate by 25%.

REFERRAL

» All to establish diagnosis and initiate treatment.
» Bleeding varices: only after commencement of resuscitation and octreotide, if available.

2.3.2 CIRRHOSIS

K74.6

DESCRIPTION

The end result of irreversible damage to the liver tissue, causing a widespread, diffuse process of fibrosis with regenerating nodule formation. The fibrosis and abnormal portosystemic vascular connections that result cause ongoing damage. The progression rate is variable, but ultimately results in liver failure.

Causes are divided into biliary cirrhosis due to bile duct obstruction and post necrotic cirrhosis where the lesion is hepatocellular.

Complications include:

- » fat malabsorbtion,
- » liver failure,
- » portal hypertension,
- » ascites secondary to hypoalbuminaemia or portal hypertension.

DIAGNOSTIC CRITERIA

Clinical

- » Clubbing.
- » Jaundice.
- » Hepatomegaly and/or splenomegaly and/or ascites.
- » Signs and symptoms of complications.

Investigations

- » Liver enzymes may be normal.
- » FBC shows signs of hypersplenism with reduced circulating red cells, white cells and platelets.
- » Prolonged prothrombin time.
- » Hypo-albuminaemia.
- » Ultrasound of the liver and spleen may be abnormal.
- » Liver biopsy confirms cirrhosis.

GENERAL AND SUPPORTIVE MEASURES

- » Ensure adequate nutrition:
  - > Consult dietician, if available.
- » If not encephalopathic:
  - > High protein diet, i.e. 3 g/kg/day and medium chain triglyceride supplementation.
  - > High carbohydrate diet, supplement with glucose polymers.
  - > If high serum cholesterol or if xanthelasma: low cholesterol diet.

MEDICINE TREATMENT

- Multivitamin, oral, 5 mL as a single daily dose.

If INR is abnormal, consider a trial of vitamin K and if no response stop.

- Vitamin K_1 (phytomenodione), oral, 5 mg daily.

2.3.2.1 ASCITES, DUE TO HYPOALBUMINAEMIA AND/OR PORTAL HYPERTENSION

R18

GENERAL AND SUPPORTIVE MEASURES

» Restrict sodium intake, 1–2 mmol/kg/24 hours.
» Restrict fluids.
» If respiratory efforts are compromised by abdominal distension, careful removal of fluid by abdominal paracentesis may be necessary.

MEDICINE TREATMENT

- Spironolactone, oral, 1–3 mg/kg as a single daily dose.
  - o Continue for as long as needed to control ascites.
  - o Monitor serum potassium.
  - o Maximum dose: 25 mg.

If insufficient response, add:

- Furosemide, oral, 1–3 mg/kg as a single daily dose.

OR (do not give furosemide and hydrochlorothiazide together)

- Hydrochlorothiazide, oral, 1 mg/kg/dose 12–24 hourly.
  - o Maximum dose: 25 mg daily.

REFERRAL

» **Urgent:** Refractory ascites interfering with respiration.
» For determination of the underlying cause of the cirrhosis, portal hypertension and initiation of treatment.
» Cirrhosis, portal hypertension and/or liver failure not responding to adequate therapy.
» Hepatic encephalopathy.

2.3.3 PORTAL HYPERTENSION

K76.6

DESCRIPTION

Increased portal venous pressure above vena cava pressure. Most commonly secondary to cirrhosis, but causes without cirrhosis may be divided into:

» prehepatic portal vein obstruction,
» intrahepatic presinusoidal (e.g. bilharzia),
» intrahepatic post-sinusoidal.

DIAGNOSTIC CRITERIA

Clinical

- » Splenomegaly with recurrent ascites, variceal haemorrhage or hypersplenism.

Investigations

- » FBC shows hypersplenism.
- » Doppler assisted ultrasound and angiography.
- » Venacavagram.

GENERAL AND SUPPORTIVE MEASURES

- » Determine and manage underlying cause.

REFERRAL

- » All with symptoms.
- » All with acute cause and not presentation.

2.3.4 HEPATITIS, VIRAL, ACUTE

B17.9

* Notifiable condition

DESCRIPTION

Acute inflammation of the li ver with varying degrees of hepatocellular necrosis caused by hepatitis A, B and less commonly C, D and E viruses.

DIAGNOSTIC CRITERIA

Clinical

- » Prodromal phase:
  - > nausea,
  - > vomiting,
  - > fever, and
  - > malaise,
  - > anorexia,
  - > right upper quadrant abdominal pain.
- » Jaundice, tender hepatomegaly and dark urine.

Investigations

- » Raised transaminases and bilirubin.
- » Serological evidence of hepatitis virus infection. See section 2.3.7 Hepatitis B, chronic for Hepatitis B interpretation chart

GENERAL AND SUPPORTIVE MEASURES

- » Isolate patient if Hepatitis A for 7–10 after the onset of jaundice.
- » Inform patient of infectivity risk if hepatitis B, C or D.
- » Low fat, high carbohydrate diet or any diet that the patient tolerates.
- » Bed rest does not alter the course of the disease.

MEDICINE TREATMENT

Prophylaxis

- Hepatitis B vaccine, IM, 0.5 mL.
  - o If < 1 year: outer side of the right thigh.
  - o If > 1 year: upper arm.

  Use opposite side to that for the DPT/Td injection.
  Give at 6, 10 and 14 weeks.

Neonatal transmission:

Babies born to mothers with acute hepatitis B infection at the time of delivery or to mothers who are HBsAg-positive or HBeAg-positive:

- Hepatitis B immunoglobulin, IM, 0.5 mL within 12 hours of delivery.

PLUS

- Hepatitis B vaccine, IM, first dose within 12 hours of delivery.
  - o Continue hepatitis B immunisation according to the recommended immunisation schedule.

REFERRAL

» Acute hepatitis with bleeding tendency and altered level of consciousness – isolation recommended.
» Prolonged jaundice or raised transaminases.
» Chronic hepatitis with/without cirrhosis.

2.3.5 HEPATITIS, TOXIN INDUCED, ACUTE

K71.6

DESCRIPTION

Liver damage attributed to a toxin or medicine. The most common herbal toxin in South Africa is atractyloside (*Impila)*, which causes a Reye's-like syndrome, with liver failure. *Senecio* ingestion is also seen but this causes endothelial damage in hepatic veins, resulting in veno-occlusive disease with secondary cirrhosis and portal hypertension.

There are many medicines that are hepatotoxic. The commonest are:

» anticonvulsants,
» cytotoxics,
» analgesics,
» antiretrovirals.
» immunosuppressants,
» anti-inflammatories,
» antituberculous medication,

DIAGNOSTIC CRITERIA

» Depends on the toxin, but the history is usually diagnostic.
» *Impila* poisoning, given orally or rectally, may result in anicteric hepatic encephalopathy.

» Presents with onset of severe vomiting, followed by anuria then rapid depression of level of consciousness, progressing to seizures and/or coma within a day.

GENERAL AND SUPPORTIVE MEASURES

» Stop all potential hepatotoxic medication including paracetamol.
» Education regarding herbal toxins, if appropriate.

MEDICINE TREATMENT

For paracetamol poisoning
See section 18.1.11: Paracetamol poisoning.

Acute liver failure/Hepatic encephalopathy:
See section 2.3.8: Liver failure, acute.

REFERRAL

» All cases of hepatic encephalopathy due to toxin ingestion.
» All cases in which rechallenge of medication is considered.

2.3.6 HEPATITIS, CHRONIC, AUTOIMMUNE

K75.4

DESCRIPTION

Autoimmune induced hepatitis.

DIAGNOSTIC CRITERIA

Clinical

» Jaundice.
» Hepatosplenomegaly.
» Cutaneous features of chronic liver disease.
» Extrahepatic manifestations of the autoimmune process.

Investigations

» Elevated bilirubin and transaminases.
» Hypoalbuminaemia and prolonged prothrombin time.
» Auto-immune marker screen.
» Protein electrophoresis shows increased gammaglobulin > 25 g/L.
» Diagnosis confirmed on liver biopsy.

MEDICINE TREATMENT

■ Corticosteroids. Specialist initiated.

AND/OR

• Azathioprine. Specialist initiated.

REFERRAL

» All for confirmation of diagnosis and initiation of treatment.

2.3.7 HEPATITIS B, CHRONIC

B18.1

DESCRIPTION

Persistently elevated transaminases after hepatitis B infection.

DIAGNOSTIC CRITERIA

» Liver biopsy is characteristic.
» Hepatitis B serology positive.

Interpretation of Hepatitis B Serololgical Test Results

> Susceptible:

HBsAg	negative
Anti-HBc	negative
anti-HBs	negative
IgM anti-HBc	negative

> Immune due to vaccination:

HBsAg	negative
Anti-HBc	negative
anti-HBs	positive > 10 milli-unit/mL

> Immune from natural infection:

HBsAg	negative
Anti-HBc	positive
anti-HBs	positive

> Acute infection:

HBsAg	positive
Anti-HBc	positive
anti-HBs	negative
IgM anti-HBc	positive

> Chronic infection:

HBsAg	positive
Anti-HBc	positive
anti-HBs	negative
IgM anti-HBc	negative

> Four possible interpretations:

1. Recovering from acute HBV infection
2. Distantly immune anti-HBs level to low to detect
3. Susceptible with false positive anti-HBc
4. Chronic infection with HBsAg levels to low to detect

HBsAg	negative
Anti-HBc	positive
anti-HBs	negative

» Transaminases are double upper limit of normal.

REFERRAL

» For confirmation of diagnosis and initiation of treatment.

2.3.8 LIVER FAILURE, ACUTE

K72.0

DESCRIPTION

Acute liver failure is a devastating clinical syndrome which has a high mortality. It results from massive necrosis of liver cells leading to the development of hepatic encephalopathy. The clinical appearance can be deceptive and it is easy to under-estimate how critically ill these patients are. Refer patients early to secondary or tertiary hospital.

The following complications can occur:

- coagulopathy,
- cerebral oedema,
- encephalopathy,
- metabolic acidosis, and
- hypoglycaemia,
- renal failure,
- cardiorespiratory failure,
- sepsis.

DIAGNOSTIC CRITERIA

Clinical

Appears deceptively well in the early stages. Progressive features include:

- malaise,
- stupor,
- encephalopathy,
- bleeding tendency,
- vomiting,
- anorexia,
- foetor hepaticus,
- ascites, and
- jaundice. The absence of jaundice suggests another process, such as Reye's syndrome, which also leads to hepatic encephalopathy.

Investigations

- Raised or low liver enzymes, low serum albumin, raised bilirubin, raised blood ammonia, hypoglycaemia.
- Prolonged prothrombin time.
- Low fibrinogen.

GENERAL AND SUPPORTIVE MEASURES

- Admit to high care or intensive care unit.
- Monitor:
  - blood pressure,
  - heart rate,
  - respiration,
  - haematocrit,
  - acid-base status,
  - urine output,
  - neurological state,
  - gastro-intestinal bleeding,
  - blood glucose – 3 hourly if comatose,
  - liver and renal functions,
  - co-agulation competence (INR),
  - electrolytes: sodium, potassium, calcium and phosphate, magnesium.
- Maintain hydration.
- With encephalopathy, aim to reduce ammonia production by the gut and optimise renal excretion.

» Withdraw protein completely initially followed by restricted intake if level of consciousness improves, i.e. 0.5–1 g/kg/24 hours.
» Stop medium chain triglyceride supplements but maintain an adequate energy intake.
» Stop sedatives, diuretics and hepatotoxic medicines, if possible.

MEDICINE TREATMENT

To reduce intestinal protein absorption:

- Lactulose, oral, 1 g/kg/dose 4–8 hourly via nasogastric tube, then adjust dose to produce frequent soft stools daily.

OR

- Polyethylene glycol 59 g/L solution with sodium sulphate and electrolytes, oral/ nasogastric tube, 10–25 mL/kg/hour until clear fluid is passed rectally (about 4–6 hours).
  - o Do not use sweeteners containing sugar.
  - o Confirm the bowel is empty.
  - o Follow with regular lactulose to keep stool loose.

- Gentamicin, oral, 12.5 mg/kg/dose 6 hourly for 5 days.
  - o The intravenous formulation can be given orally.

Cerebral oedema:
For management of cerebral oedema, see section 13.5: Status epilepticus (convulsive).

For pre-operative use or with active bleeding:

- Fresh frozen plasma, IV, 20 mL/kg administered over 2 hours.

OR

- Lyophilised plasma, IV, 20 mL/kg administered over 2 hours.

- Vitamin K_1 (phytomenodione), IV/oral, 2.5–10 mg daily.
  - o Monitor response to vitamin K_1 with INR and PTT.

If platelet count < 10 x 10^9/L or if < 50 and with active bleeding:

- Platelet transfusion.

For gastrointestinal bleeding:

- Omeprazole, oral, 0.4–0.8 mg/kg/dose 12 hourly. (Specialist initiated)
  - o Maximum dose: 20–40 mg/dose.

If neonate:	0.5–1 mg/kg 12– 24 hourly.
If 1 month–2 years:	2.5 mg 12 hourly.
If > 2–6 years:	5 mg 12 hourly.
If > 7–12 years:	10 mg 12 hourly.

For hypoglycaemia:

- Dextrose 10%, IV bolus 5 mL/kg.
    - o Administer maintenance as below.

Maintenance of fluids until enteral feeding resumed:

- ½ Darrows/dextrose 5%, IV, 60–80 mL/kg/day.
    - o Ensure a minimum of 3–6 mmol/kg/day of potassium.
    - o Avoid diuretics.

For anaemia:

- Packed red cells, 10 mL/kg over 3 hours if haemoglobin < 7 g/dL.

For shock:
See Section 1.1.7: Shock.

For sedation, if essential:

- Midazolam, IV, 0.1 mg/kg.
    - o Benzodiazepines are poorly metabolised in liver failure and may result in prolonged sedation.
    - o Do not repeat without clinical indication.

Seizures are often subclinical or subtle. For seizures:

- Diazepam, IV, 0.2 mg/kg
    - o Repeat dose if not controlled in 5 minutes.
    - o Benzodiazepines are poorly metabolised in liver failure and may result in prolonged sedation.
    - o Do not repeat without clinical indication.

Amelioration of liver injury, in idiopathic/toxin cases: treat as for paracetamol poisoning. See section 18.1.11: Paracetamol poisoning.

Antibiotic therapy
Where sepsis is suspected, prevent and treat aggressively with intravenous broad spectrum antibiotics. Empiric antibiotic therapy until cultures is known.

- Ampicillin, IV, 50 mg/kg/dose, 6 hourly.

PLUS

- Cefotaxime, IV, 75 mg/kg/dose, 8 hourly.

REFERRAL

» All for determination of the underlying cause and initiation of treatment.
» Hepatic encephalopathy.
» Combined hepato-renal failure.
» Failure to contain bleeding.

2.4 MALNUTRITION

E40–E46

2.4.1 MALNUTRITION, SEVERE ACUTE

E40–E43

Z-scores

» For practical purposes a "z-score" is the number of standard deviations (SD) below or above the mean.
» 2 SD or 2 z-scores above the mean (+2) equates fairly closely to the 97th percentile and 2 SD or 2 z -scores below the mean (–2) equates fairly closely to the 3rd percentile.
» 3 SD or 3 z -scores above or below the mean would be regarded as severe deviation from normal.
» In deviation below normal consider if a reasonable explanation exists, e.g. severe low birth weight with adequate growth profile subsequently.

Admit all cases with severe complicated acute malnutrition.

Uncomplicated cases may be managed with "ready to use therapeutic food (RUTF)" in ambulatory setting where this service is established.

DESCRIPTION

Severe Acute Malnutrition (SAM)

A multi-deficiency state of severe undernutrition of essential nutrients exacerbated by acute/chronic infection and metabolic disturbances. Severe Acute Malnutrition (SAM) includes but is not restricted to the clinical entities of bilateral pitting oedema (Kwashiorkor), severe wasting (Marasmus), and combination of wasting and bilateral pitting oedema (Marasmic-Kwashiorkor). It is associated with a high but significantly modifiable mortality.

Criteria for ambulatory treatment of severe acute malnutrition,
all of the following must apply:

» Children over the age of 6 months with no pitting oedema.

PLUS

» Alert and feeding well.

PLUS

» None of the IMCI danger signs/nor those listed below.

PLUS

» Exclusion of other morbidity, TB and HIV infection.

DIAGNOSTIC CRITERIA

SAM in children aged 6–60 months:

Indicator	Measure	Cut-off
Severe wasting	Weight-for-height	z–score less than –3
	Mid upper arm circumference (MUAC)	Less than 11.5 cm
Bilateral pedal oedema	Clinical sign	

Where a suitable measuring device is not available the following less sensitive findings would also indicate the need to manage as severe acute malnutrition:

- **Severe underweight**
  - weight for age z-score less than –3 (usually clinically reflective of marasmus) where no other reasonable explanation is present, and/or
  - clinically visible severe wasting (usually clinically reflective of marasmus – thin arms, thin legs, "old man" appearance, baggy pants folds around buttocks, wasted buttocks).
- **Nutritional oedema** (usually clinically reflective of kwashiorkor – bilateral pedal oedema usually supported by findings of skin changes, fine pale sparse hair, enlarged smooth soft liver, moon face).

Danger signs:

- Lethargy (not alert).
- Shock.
- Refusing feeds.
- Weeping skin lesions.
- Hypothermia.
- Convulsions.
- Hypoglycaemia.
- Jaundice.
- Dehydration.
- Respiratory distress.
- Bleeding.
- Vomiting everything.

Note:
Any of these danger signs indicates the need for more intensive inpatient management.

Time frame for inpatient management of severe acute malnutrition

		stabilisation		rehabilitation
**Step**		**Days 1–2**	**Days 3–7**	**Weeks 2–6**
1.	Hypoglycaemia	→		
2.	Hypothermia	→		
3.	Dehydration	→		
4.	Electrolytes	→	→	→
5.	Infection	→	→	
6.	Micronutrients	No Iron →	→	Add Iron →
7.	Stabilisation feeding			
8.	Catch up growth			→
9.	Sensory stimulation	→	→	→
10.	Prepare for follow up			→

The general approach to the inpatient management of severe acute malnutrition is encapsulated in the 10 step approach illustrated above.
Within this approach the first days are involved in achieving metabolic and physical stability and th is phase usually moves to the re habilitation phase somewhere between the 3rd and 7th day of admission.

Stabilisation phase:

» feeding,
» preventing/treating hypoglycaemia,
» preventing/treating hypothermia,
» treating infections,
» giving minerals, vitamins and trace elements, and
» preventing/treating dehydration.

Rehabilitation phase:

» continued feeding,
» catch up growth,
» management chronic infections/infestations,
» continued administration of minerals and vitamins (including commencing iron),
» play and love; stimulation, and
» preparation for discharge.

Step 1: Hypoglycaemia (Blood glucose <3 mmol/L)

Prevention

Feed child with severe acute malnutrition immediately (within 30 minutes of presentation) and then ensure every feed is given by day and at night. See step 7: Stabilisation feeding.

Keep the child warm. See step 2: Hypothermia.

Detection and treatment

Test blood glucose level 3 hourly in severely ill child for first 24 hours and until stable (longer if the child is very ill).

Asymptomatic hypoglycaemia:

If blood glucose < 3 mmol/L in asymptomatic child, give immediately (oral bolus):

- Stabilisation/F75 formula, oral, 15 mL/kg.

OR

- Dextrose, 10%, oral, 10 mL/kg.
  - Dextrose 10% = Dextrose 50% 2 mL/kg with water for injection 8 mL//kg.

OR

- Sugar solution, oral, 10 mL/kg
  - 1 rounded teaspoon sugar in 50 mL or 3 ½ tablespoons of water.

Check blood glucose after 30 minutes and maintain it above 3 mmol/L.

Continue feeds.

If symptomatic or persistent hypoglycaemia:

- Dextrose, 10%, IV, 5 mL/kg.

OR

- Neonatal maintenance solution, IV, 5 mL/kg.

Continue feeds once responsive.

- Change feeds to 2 hourly if hypoglycaemia has occurred. See step 7: Stabilisation feeds.

These children have poor cardiac reserves and are easily volume overloaded. Do not start or maintain IV infusions unless absolutely necessary.

Step 2: Hypothermia (Axillary temperature <35ºC)

Prevent hypothermia

Care for child in a warm area, i.e. 25–30ºC room temperature.

Ensure child's body, especially the head, is covered at all times particularly at night.

Avoid drafts and change wet napkins/clothing.

Avoid exposure e.g. bathing.

Feed immediately and 2–3 hourly as this provides energy to generate heat.

Allow child to sleep with mother/carer at night for warmth.

Treat hypothermia

Check axillary (underarm) temperature, 3 hourly.

Axillary temperature <36ºC indicates an urgent need to warm child.

Allow child to sleep with mother/carer at night for warmth. Use mother-child skin-skin contact, i.e. Kangaroo care, to keep child warm and wrap both with blankets.

Place heater nearby. If a radiant heater is use d for warming check temperature at least every ½ hour.

If severely hypothermic and not improving use other heating measures but do not apply direct heat to the skin as this may burn the child.

Check temperature 2-hourly until > 36.5ºC.

Consider and treat for infection and sepsis. See step 5: Infection.

Step 3: Dehydration

See section 2.2.4: Diarrhoea, acute.

Continue feeds and other care of severe malnutrition.

Step 4: Electrolytes (hypokalaemia/hypomagnesaemia and hypernatraemia)

All severely malnourished children have excess body sodium even though the plasma sodium may be low. Oedema is partly due to these imbalances, not fluid overload.

Giving high sodium load fluids is dangerous.<br>Do **NOT** treat oedema with a diuretic.

Potassium

Serum potassium does not indicate total body potassium status. Potassium supplementation is required unless frank hyperkalaemia.

Feeds made with combined mineral and vitamin complex contains potassium. When this is used, do not add further potassium.

If the formula is made without combined mineral and vitamin complex**, add** potassium:

- Potassium chloride solution, 25–50 mg/kg/dose, oral, 8 hourly until oedema subsides:
  - If < 10 kg: 250 mg.
  - If > 10 kg: 500 mg.

Magnesium
Feeds made with combined mineral and vitamin complex or trace element mix contains magnesium. If formula is made without either of these additives, **add** magnesium:

- Trace element mix, oral, daily.
  - If < 10 kg: 2.5 mL.
  - If > 10 kg: 5 mL.

OR

- Magnesium sulphate 50%, oral, 0.2 mL/kg as a once daily dose for at least 2 weeks. The IV preparation can be given orally.

Step 5: Infection

Antibiotics
Start antibiotics on the first day at admission.

If the child has no danger signs, is alert and feeding well:

- Amoxicillin, oral, 30 mg/kg/dose 8 hourly for 5 days.

All other children:

- Ampicillin, IV/IM, 50 mg/kg 6 hourly for 7 days.
  - Avoid IV infusions, if possible. Use a heparin lock to avoid fluid overload because of poor cardiac reserves.

PLUS

- Gentamicin, IV, 6 mg/kg once daily for 7 days.

As soon as there is a response and patient can tolerate oral medication change ampicillin to amoxicillin and continue with gentamicin:

- Amoxicillin, oral, 30 mg/kg/dose 8 hourly for a further 5 days.

PLUS

- Gentamicin, IV/IM, 6 mg/kg once daily for 7 days.

If the child is severely ill or fails to improve after 48 hours:

- ■ Third generation cephalosporin, e.g.:
- Ceftriaxone, IV/IM, 50 mg/kg/dose once daily.
  - If meningitis suspected: use 80 mg/kg/dose.

CAUTION: USE OF CEFTRIAXONE

After 28 days of age IV ceftriaxone and IV calcium containing fluids may be given but only sequentially with the giving set flushed well between the two products.

Note: Do not use ceftriaxone in neonates.

If child does not improve after 5 days, or deteriorates:

- Refer to higher level of care.

Intestinal worm infestation
Treat after the acute phase:
Children 1–2 years of age:
- Mebendazole, oral, 100 mg 12 hourly for three days.

Children > 2 years:
- Mebendazole, oral, 500 mg as a single dose immediately.

HIV and TB
In children with HIV and TB, good recovery from malnutrition is possible but may take longer. Treatment failure of malnutrition may be more common.

Actively investigate for TB and HIV as soon as possible.

TB is difficult to diagnose and confirm.
Ask about contacts, symptoms, do tuberculin skin test (TST) and chest X-ray. If TST negative, repeat just before discharge.
If TB is clinically likely, presumptive TB treatment is often reasonable, but once begun should be completed. See section 15.1.4: Tuberculosis, pulmonary.

HIV is relatively simple to diagnose and confirm.
Children < 18 months: PCR and confirm with viral load > 10 000 copies/mL.
Children ≥ 18 months: rapid test/ELISA and confirm with different rapid test/ELISA.
ART is the same as for HIV negative children.
Once the child enters the rehabilitative phase, commence antiretroviral therapy without delay if HIV positive. See section 9.1: Human immunodeficiency virus infections.

Step 6: Micronutrients
Vitamins
- Vitamin A, oral, as a single dose:

Age	Dose	No. of capsules
Infants < 6 months:	50 000 IU	1 capsule
Infants 6–11 months:	100 000 IU	1 capsule
Children 12 months to 5 years:	200 000 IU	1 capsule

Record doses in the Road-to-Health booklet.

All children with clinical signs of severe vitamin A deficiency (eye changes: xerophthalmia, corneal ulceration, Bitots spots, corneal clouding) **and** severe measles:
- Vitamin A, oral, 3 doses.
  - First dose, immediately; second dose on day 2 and third dose after 14 days.
  - Record the dose given in prescription and the Road to Health Book.

If on feeds with combined mineral and vitamin complex:

- Folic acid, oral, 2.5 mg as a single dose.

If not on feeds with combined mineral and vitamin complex:

- Folic acid, oral, 2.5 mg as a single daily dose.

PLUS

- Multivitamin, oral, 5 mL as a single daily dose.

<u>Anaemia in malnourished children</u>
Non-acute management:
Although anaemia is common, do NOT give iron initially but wait until the child has a good appetite and starts gaining weight (usually by the second week).

Treat severe anaemia with blood transfusion, if:

» Symptomatic anaemia (Hb usually below 4 g/dL).

OR

» If there is respiratory distress with a low Hb.

- Packed red cells, IV, 5 mL/kg administered over 3 hours.

PLUS

- Furosemide, IV, 1 mg/kg at the start of the transfusion.

Repeat only if severe anaemia or respiratory distress persists and the haemoglobin is still low.

Once gaining weight and oedema has resolved:

- Iron, oral, 2 mg/kg elemental iron per dose 8 hourly with meals.
  - o Continue for at least 2 months to replace iron stores.

Step 7: Stabilisation feeding
<u>Immediate: stabilisation phase:</u>
Begin feeding immediately – do not miss feeds.
Give "F75/stabilising feed" at 130 mL/kg/day divided into 3 hourly feeds, i.e. 8 times daily. Give all feeds including that at 03h00.
If child has gross oedema i.e. if the oedema is up to or beyond the knee or anasarca, give 100 mL/kg initially and increase progressively.
Monitor and record intake carefully.

**F75 formula/Stabilisation**	
Fresh cow's milk	300 mL
Sugar	100 g
Vegetable oil	20 g
Combined mineral and vitamin complex*	indicated by insert
Water to make up to:	1 000 mL

* If no combined mineral and vitamin complex:

- Trace element mix, oral, 20 mL daily.

If danger signs, hypothermia or hypoglycaemia present, feed the same daily volume but divided into 2 hourly feeds, i.e. 12 times daily. Give all feeds including those at 02h00 and 04h00.
Give from a cup. Very weak children may be fed by spoon, dropper or syringe.
If feeds refused/not finished (i.e. less than 80% of daily amount taken) give all feeds via nasogastric tube.
Weigh daily and plot weight gain.

Readiness to enter the rehabilitation phase is signalled by a return of appetite, usually about one week after admission.

Step 8: Transition feeding and catch up growth

Feeding (rehabilitation phase)

» Transition
  For the first two days replace the initial feeds with equal amounts of "rebuilding/catch-up/F100 formula". Gradually increase the volume by 10 mL/feed until some formula remains unfinished, usually ± 200 mL/kg/day.
  When appetite returns introduce a modified diet. Balance the intake by giving 3 modified meals and 5 feeds of F100. Prepare food without adding salt.

**F100 formula/Rebuilding formula (catch-up)**	
Fresh cow's milk	880 mL
Sugar	75 g
Vegetable oil	20 mL
Combined mineral and vitamin complex *	as indicated by insert
Water to make up to:	1 000 mL

* If no combined mineral and vitamin complex:
- Trace element mix, oral, 20 mL.

Monitor progress after the transition by assessing the rate of weight gain.
Weigh child each morning before feeding and plot the weight.
Each week calculate and record weight gain as g/kg/day.

If weight gain is:
» poor (< 5 g/kg/day) – child requires full reassessment.
» moderate (5–10 g/kg/day) – check whether intake targets are being met, or if infection has been overlooked.
» good (>10 g/kg/day) – continue to praise staff and mothers.

Step 9: Sensory stimulation

Stimulation and loving care

» Provide tender loving care.
» Help and encourage mothers to comfort, feed and play with their children.
» Involve occupational therapist, if available, for structured play otherwise arrange this as best possible in the ward.
» Provide a stimulation program in the ward.

Step 10: Prepare for follow up

Preparation for discharge

» Obtain information on household food security, family background and socio-economic status and refer appropriately.
» Instruct mothers how to modify family foods, how often to feed, what and how much to give.
» Ready to Use Therapeutic Foods (RUTF) may be supplied to facilitate earlier discharge where this is indicated and available.
» Involve mother in discharge planning and follow up plans.
» Social assessment. Before discharge, ensure parent/caregiver is able to access food for the child, ensure all financial supports and grants have been accessed. A social worker may assist in ensuring this. The social worker should also assess for other social risks.
» Make follow-up arrangements. Link patient to PHC systems and Family Health Teams/CCG Workers for close follow-up and monitoring of feeding and compliance with therapeutic feeding program.
» Ensure all immunisations are up to date.
» Do not discharge any malnourished child without having adequately investigated for TB and HIV infection. Repeat TST before discharge as immunity may have returned to normal.
» Write full clinical summary in Road to Health book.

Discharge criteria

» good appetite,
» no infection,
» no oedema,
» continuous good weight gain for last 5 days,
» playful and alert, and
» all preparation in place for discharge.

Feed volume charts

Initial stabilisation /F75 formula volumes at 130 mL/kg/day

Use 2 hourly if child very sick or has hypoglycaemia or hypothermia

Childs Weight (kilograms)	Amount feed		If total volume taken in a day is less than the below figure change to nasogastric feeding
	Every 3 hours 8 times a day	Every 2 hours 12 times a day	
2	35	25	210
2.1	35	25	220
2.2	35	25	230
2.3	40	25	240
2.4	40	25	250
2.5	40	25	260
2.6	40	30	270
2.8	45	30	290
3	50	30	310
3.2	50	35	330
3.4	55	35	350
3.6	60	40	370
3.8	60	40	400
4	65	45	420
4.2	70	45	440
4.4	70	50	460
4.6	75	50	480
4.8	80	50	500
5	80	55	520
5.2	85	55	540
5.4	90	60	560
5.6	90	60	580
5.8	95	65	600
6	100	65	620
6.5	105	70	670
7	115	75	730
7.5	120	80	780
8	130	90	830
8.5	140	90	880
9	150	100	940
9.5	150	100	990
10	160	110	1050

If severe oedema decrease volume by 25% per feed initially and then increase progressively to above volumes.

2.5 RICKETS
E55.0

DESCRIPTION
Failure to calcify osteoid tissue in a growing child, usually due to deficiency of vitamin D, its ac tive metabolites, calcium, phosphorus or other ra re causes. This leads to bone deformity.

Occurs in ex-premature babies during infancy and in children with developmental disability, on anticonvulsants or not exposed to sunlight. In older children it is caused by renal tubulopathy and other rare conditions.

DIAGNOSTIC CRITERIA
Clinical
» Bowing of long bones, widening of metaphyses and cranial bossing.
» Occasionally convulsions or tetany due to hypocalcaemia.

Investigations
» Elevated alkaline phosphatase.
» Serum calcium and/or phosphate abnormalities.
» X-ray of wrists.

GENERAL AND SUPPORTIVE MEASURES
» Prevent vitamin D deficiency.
» Exposure to sunlight, at least 3 hours a week.
  Note:
  Breast milk does not contain adequate vitamin D to prevent deficiency.
  Ensure adequate sunlight exposure of infant or provide vitamin D until weaning.
» Normal vitamin D-containing diet for lactating mothers.

MEDICINE TREATMENT
Prophylaxis
For premature babies:
- Vitamin D, oral, 800 IU, once daily.

Infants who are exclusively breastfed or not on adequate volume of commercial milk formula:
- Vitamin D, oral, 400 IU, once daily.

Treatment of active rickets
Treat only after confirmation of active rickets on X–ray.
- Vitamin D, oral, 5 000 IU, once daily, in addition to milk in the diet.
  - Repeat X–ray after 6–8 weeks.
  - If no radiological improvement, further investigation is required.
  - If healing occurs, continue for 3 months. Confirm complete healing and adequate diet for the future.

REFERRAL

» Rickets presenting in children older than 2 years.
» No radiological response to treatment after 6–8 weeks.
» Incomplete radiological response.
» Rickets secondary to other disease processes.

2.6 WORM BOLUS

B77

DESCRIPTION

Partial or complete obstruction of t he bowel by a “k not” of *Ascaris lumbricoides* curled around each other. Usually presents with cramping abdominal pain with/without other evidence of obstruction. May occasionally lead to local necrosis and perforation of the small bowel.

DIAGNOSTIC CRITERIA

Clinical

Cramping abdominal pain associated with/without a palpable worm mass which may also be identified on X-ray abdomen straight or with contrast (when considered safe).
Exclusion of other cause of acute abdomen or acute abdominal pain.

GENERAL AND SUPPORTIVE MEASURES

» Maintain fluid, electrolyte and nutritional needs, IV ro ute may be needed.
» Nil per mouth and free drainage, where clinically indicated.
» Observe for fai lure of resolution, complete obstruction or evidence of necrosis/perforation.
» Surgery for complete obstruction, evidence of necrosis or perforation.
» Identify possible iron deficiency.

MEDICINE TREATMENT

Once the bolus resolve treat the ascaris:
Children 1–2 years of age:

- Mebendazole, oral, 100 mg 12 hourly for three days.

Children > 2 years:

- Mebendazole, oral, 500 mg as a single dose immediately.

REFERRAL

» Inability to manage surgical problems, if present.
» Prolonged obstruction.

2.7 RECURRENT ABDOMINAL PAIN

R10.4

DESCRIPTION

Recurrent abdominal pain for which no cause can be found occurring at least monthly for 3 consecutive months with severity that interferes with routine function of the child.

DIAGNOSTIC CRITERIA

Clinical

» Peri-umbilical pain associated with belching, bloating with negative findings on clinical evaluation and no response to acid-blocking medication **OR p**ain below the umbilicus accompanied by abdominal cramps, bloating and distension and with an altered bowel pattern that are consistent with Irritable Bowel Syndrome in adults.
» Either of the above syndromes with the exclusion of organic disease with appropriate investigation.
» Avoid excessive investigation where the diagnosis is strongly suspected in the presence of a normal clinical evaluation.
» Exclude the following:
  > Urinary tract infections, urinary tract anomalies, renal disease.
  > GIT infection, infestation or inflammation.
  > Chronic abdominal conditions such as tumours or infections, e.g. TB abdomen.
  > Gall bladder disease.
  > Pancreatic disease.

GENERAL AND SUPPORTIVE MEASURES

» Manage psychological stressors, anxiety or depression, where present, appropriately.
» Reassure child and family.
» Counselling to avoid the re-inforcement of the symptoms with secondary gain.
» Adequate dietary fibre in children with irritable bowel syndrome-type condition.

MEDICINE TREATMENT

Manage constipation, where present. See section 2.2.2: Constipation/faecal loading.

Manage comorbid anxiety or depression appropriately. See section 14.3.1: Depression in Childhood and Adolescence and section 14.4: Anxiety disorders.

REFERRAL

» Failure to respond to management
» For appropriate psychiatric / psychological management, if not locally available.

CHAPTER 3

BLOOD AND BLOOD-FORMING ORGANS

3.1 ANAEMIA, APLASTIC

D61.0

DESCRIPTION

Pancytopaenia caused by bone marrow failure with a hypocellular bone marrow without infiltration or fibrosis. May be acquired or inherited. Inherited bone marrow failure syndromes include Fanconi anaemia which has specific associated phenotypic features and chromosomal abnormalities.

DIAGNOSTIC CRITERIA

Clinical

- » Pallor, petechiae, purpura, bleeding, with frequent and/or severe infections.
- » Phenotypic features of Fanconi anaemia include:
  - > café au lait spots,
  - > skin pigmentary changes,
  - > short stature and dysmorphic faces,
  - > hypoplasia/absence of radius, fingerised thumb,
  - > microcephaly, small eyes, hyperreflexia,
  - > renal tract and cardiac abnormalities,
  - > hypogonadism.

Investigations

- » Full blood count shows pancytopaenia, with anaemia (may be macrocytic), leucopaenia and thrombocytopaenia.
- » Hypoplastic bone marrow on trephine biopsy.

GENERAL AND SUPPORTIVE MEASURES

Limit the use of blood products as the patient may be sensitised and jeopardise a future bone marrow transplant.

Avoid contact sport.

MEDICINE TREATMENT

For symptomatic anaemia (usually Hb < 7 g/dL):

- Packed red cells, IV.
  - o Use leukocyte depleted products.

For active bleeding:

- Platelets, IV, 20 mL/kg, administered immediately and rapidly over 15–30 minutes through a platelet giving set.
  - o If transplant is a possibility, use single donor apheresis platelets rather than pooled random donor platelets; preferably group specific.

For fever (T > 38ºC), broad spectrum antibiotics:
Take blood cultures first.

- Ceftriaxone, IV, 50–80 mg/kg once daily.

OR
If < 1 month old:

- Cefotaxime, IV, 25–50 mg/kg/dose, 6 hourly.

AND

- Amikacin, IV, 25 mg/kg as a loading dose, then 18 mg/kg/dose once daily.

REFERRAL

» All cases of suspected aplastic anaemia.
» Stabilise patient before transport with blood and/or platelet transfusions, if necessary, after consultation with a paediatrician or paediatric haematologist.
» All cases for consideration for bone marrow transplant or immunosuppressive therapy in the case of acquired aplastic anaemia.

3.2 ANAEMIA, HAEMOLYTIC

D55–59

DESCRIPTION

Anaemia caused by excessive destruction of red blood cells.
Destruction may be due to:

» Corpuscular defects:
  > abnormalities of the cell membrane (e.g. hereditary spherocytosis),
  > enzyme abnormalities (e.g. G6PD deficiency), or
  > abnormal haemoglobin (e.g. sickle cell anaemia, thalassaemia).

» Extracorpuscular defects:
  > Auto-immune or isoimmune:
  idiopathic warm or cold antibodies,
  infection triggered e.g. Mycoplasma pneumonia,
  medicine related e.g. penicillin,
  secondary to auto-immune disorders e.g. SLE, rheumatoid arthritis,
  secondary to tumours e.g. lymphoma, thymoma.
  > Non-immune:
  secondary to microangiopathy e.g.: haemolytic uraemic syndrome,
  infections causing haemolysis e.g. malaria,
  miscellaneous causes including hypersplenism.

DIAGNOSTIC CRITERIA

Clinical

- » Pallor, jaundice, fatigue.
- » Splenomegaly.

Investigations

- » Anaemia.
- » Evidence of haemolysis:
  - > anaemia,
  - > reticulocytosis,
  - > increased lactate dehydrogenase (LDH),
  - > urobilinogen in the urine.
  - > decreased haptoglobin,
  - > unconjugated hyperbilirubinaemia,
- » Direct Coombs test (direct antiglobulin) is positive with autoimmune haemolysis.
- » Renal function is abnormal in haemolytic uraemic syndrome.
- » Exclude other autoimmune disorders.
- » Consider underlying neoplasms.
- » In patients receiving recurrent transfusions (e.g. thalassaemia) monitor ferritin levels 3-monthly and discuss with your referral centre if elevated > 1000 mcg/L.

GENERAL AND SUPPORTIVE MEASURES

- » After appropriate investigations, transfuse the patient and then discuss with a paediatrician or paediatric haematologist.
- » Coombs-positive auto-immune haemolytic anaemia may require transfusion with the least incompatible blood (if cross-matching yields no compatible units).
- » In G6PD deficiency, avoid medicines known to cause haemolysis (e.g. aspirin, sulphonamides and primaquine) **and** be sure to give the patient a list of such medicines at discharge.

MEDICINE TREATMENT

Warm antibody autoimmune haemolytic anaemia

Under specialist supervision:

- Prednisone, oral, 2 mg/kg/24 hours until a satisfactory response is obtained.
  - o Continue treatment for a minimum of 4 weeks.
  - o Taper dose slowly over several weeks while monitoring for relapse.

Chronic haemolytic anaemia

All patients indefinitely:

- Folic acid, oral, 2.5–5 mg daily (depending on the size of the child).

SURGICAL TREATMENT

Splenectomy for hereditary spherocytosis with Hb < 10 g/dL **only** after the child’s fifth birthday.

Pre-splenectomy
Children who have been v accinated with PCV 7 as part of their ro utine should receive PCV 10 or 13 eight weeks before polysaccharide vaccine.
- Pneumococcal vaccine (polysaccharide), IM, 0.5 mL as a single dose.

Post splenectomy
- Phenoxymethylpenicillin, oral, 12 hourly.
  - If < 5 years: 125 mg.
  - If > 5 years: 250 mg.
  - Give indefinitely until at least until 18 years.

- Pneumococcal polysaccharide vaccine. Repeat vaccination as a booster 5 years after the initial dose.

- Annual influenza vaccine.

- Catch up conjugate pneumococcal vaccine
  - < 12 months of age: 3 dose series.
  - 12 months of age and older: 2 doses 8 weeks apart.

REFERRAL
» Any child with haemolytic anaemia who has received more than 10 transfusions for assessment for chelation therapy.
» All cases associated with evidence of haemolysis as above should be managed in consultation with a paediatrician or paediatric haematologist.

3.3 ANAEMIA, MEGALOBLASTIC
D53.1

DESCRIPTION
Anaemia caused by a deficiency of folate and/or vitamin B_{12}.

DIAGNOSTIC CRITERIA
Clinical
» Pallor and fatigue.
» Chronic diarrhoea.
Investigations
» Megaloblastic anaemia: elevated MCV (mean corpuscular volume) and MCH (mean corpuscular haemoglobin).
» Macro-ovalocytes on blood smear, hypersegmentation of neutrophils.
» Decreased serum vitamin B_{12} or red blood cell folate.
» Investigations to identify reason for folate or B_{12} deficiency, e.g. malabsorption.
» Pancytopaenia in severe cases.
» Actively exclude leukaemia and aplastic anaemia which may cause macrocytosis.

GENERAL AND SUPPORTIVE MEASURES

» Dietary modifications to ensure adequate intake of folate and vitamin B_{12}.
» Packed red blood cell transfusion for symptomatic anaemia. Try to avoid blood transfusion until all investigations have been done.

MEDICINE TREATMENT

Folic acid deficiency:

- Folic acid, oral, 5 mg daily until haemoglobin returns to normal value for age. Prolonged treatment may be needed for malabsorption states and congenital deficiencies.

Vitamin B_{12} deficiency:

- Vitamin B_{12}, IM, 500 mcg monthly. Prolonged treatment may be needed.

REFERRAL

» All cases of megaloblastic anaemia, except clear nutritional folate deficiency.

3.4 ANAEMIA, IRON DEFICIENCY

D50.9

DESCRIPTION

Iron deficiency is the most common cause of anaemia. The commonest causes of iron deficiency anaemia are poor nutritional intake, excessive milk ingestion and blood loss due to parasites (whipworm and hookworm).

Lower limits of normal haemoglobin:

Age	Hb (g/dL)
Birth	13.5
6 weeks	9.5
3 months	10.0
6–12 months	10.5
12–18 months	10.5
18 months–4 years	11.0
4–7 years	11.0
7–12 years	11.5
12 years and older	12 (F) : 13 (M)

Practically: Ferrous gluconate syrup 1 mL/kg/day in 2 doses.

DIAGNOSTIC CRITERIA

Clinical

Symptoms and signs vary with the severity of the deficiency:

- pallor,
- fatigue,
- irritability,
- behavioural and cognitive effects.
- delayed motor development,
- pica,
- soft ejection systolic murmur,

Investigations

- Haemoglobin below normal for age.
- Hypochromic microcytic anaemia.
- Low MCV (mean corpuscular volume) and MCH (mean corpuscular haemoglobin), increased red cell distribution width.
- Decreased serum iron, ferritin and transferrin saturation.
- Elevated total iron binding capacity.
- Stool examination to identify intestinal parasites or to confirm occult blood loss.
- Iron studies are not necessary if nutritional iron deficiency is strongly suspected. Document a response to a trial of iron therapy to confirm the diagnosis.

Note:
Chronic infections may also cause microcytic hypochromic anaemia.
See section 3.5: Anaemia of chronic disorders (infection or disease).

GENERAL AND SUPPORTIVE MEASURES

- Dietary adjustment.
- Counselling.

MEDICINE TREATMENT

Treatment

- Iron (elemental), oral, 3 mg/kg/dose 12 hourly with meals.

Elemental iron per preparation

Ferrous gluconate elixir	350 mg/5 mL	40 mg elemental iron/5 mL	8 mg elemental iron per mL
Ferrous gluconate syrup	250 mg/5 mL	30 mg elemental iron per 5 ml	6 mg elemental iron per mL
Ferrous lactate drops	25 mg/mL	25 mg elemental iron/ mL	1 mg elemental iron in 0.04 mL
Ferrous sulphate compound tablets	170 mg	± 65 mg elemental iron per table	± 65 mg elemental iron per tablet

Follow up at monthly intervals.
The expected response is an increase in Hb of 2 g/dL or more in 3 weeks.
Continue for 3–4 weeks after Hb is normal to replenish body iron stores.
The reticulocyte count will increase if there is a positive response and may be useful where the diagnosis is in doubt, if done within 1–2 weeks after iron therapy is started.

Treat for worms.
Children 1–2 years of age:
- Mebendazole, oral, 100 mg 12 hourly for three days.

Children > 2 years:
- Mebendazole, oral, 500 mg as a single dose immediately

CAUTION
Iron is extremely toxic in overdose, particularly in children<br>All medication should be stored out of reach of children

Prophylaxis
All premature babies, day 15 to 1 year:
- Iron (elemental), oral, 2 mg/kg daily.
- Multivitamin, drops, oral, 0.3 mL daily.
  - o Increase dose according to age.

Full term babies after 2 months:
- Iron (elemental), oral, 1 mg/kg daily for one year.
- Multivitamin, drops, oral, 0.6 mL daily

REFERRAL
» Patients not responding to adequate therapy.
» Patients in whom easily treatable causes for non-response have been excluded, e.g.:
  > non-adherence to therapy,
  > ongoing blood loss,
  > ongoing infection.

3.5 ANAEMIA OF CHRONIC DISORDERS (INFECTION OR DISEASE)
D63

DESCRIPTION
Anaemia caused by chronic infection or disease. This may be due to interference with nutrient supply or suppression of haemopoiesis. Iron may be trapped in the reticuloendothelial system resulting in relative iron deficiency.

Symptomatic anaemia may manifest with tachypnoea, tachycardia not attributable to other causes and heart failure.

DIAGNOSTIC CRITERIA

Clinical

» Pallor, fatigue;
» Features of malnutrition or chronic infection e.g. TB, HIV, chronic renal failure; or
» Auto-immune disease may be present.

Investigations

» Haemoglobin low with usually normocytic, normochromic red cells (may be microcytic).
» TST, chest X-ray and renal function tests.

GENERAL AND SUPPORTIVE MEASURES

» Emphasise a nutritionally balanced diet that is adequate in protein, vitamins and minerals for nutritional rehabilitation.
» Transfuse for symptomatic anaemia only.

MEDICINE TREATMENT

» Treat underlying infection, e.g. TB.
» Defer iron treatment until acute diseases are controlled, then provide extra iron (see above) and multivitamins.

REFERRAL

» All cases with unresolving anaemia and no cause found.

3.6 ANAEMIA, SICKLE CELL

D57

DESCRIPTION

Haemolytic anaemia due to homozygous inheritance of the sickle cell gene.
Patients may experience complications:

» Painful vaso-occlusive crises.
» Haemolytic crises (usually secondary to infection).
» Aplastic crises.
» Thrombotic crises, e.g. acute chest syndrome, priapism or stroke.
» Splenic sequestration.
» Severe infections.

DIAGNOSTIC CRITERIA

Clinical

» Pallor, jaundice, fatigue all of which may worsen abruptly (sequestration crisis, aplastic crisis).
» Features of complications:
  > painful swelling of the hands and feet (dactylitis);
  > bone pain, abdominal pain;
  > chest pain, fever, dyspnoea (acute chest syndrome);
  > convulsions, hemiparesis;
  > priapism.

Investigations

» Laboratory features of haemolytic anaemia. See section 3.2: Anaemia, haemolytic.
» Haemoglobin electrophoresis shows an SS pattern (both parents will be AS).

GENERAL AND SUPPORTIVE MEASURES

» Avoid exposure to cold, dehydration and stress.
» Increase fluid intake during painful crises.
» Heat and/or massage for pain.

MEDICINE TREATMENT

For sequestration crisis or aplastic crisis:

- Packed red cells, IV, 15 mL/kg.
  - o Avoid over-transfusing patients since the increase in viscosity may aggravate the vasculopathy associated with sickle cell disease (a Hb threshold of 13 g/dL has been recommended).

If hypoxic:

- Oxygen, by face mask.

Exchange transfusions may be used to treat severe complications (see referral criteria).

Prophylaxis against infection

Given to all children because functional asplenia is present by 1–2 years of age.

- Routine vaccinations during infancy.

- Catch up conjugate pneumococcal vaccine
  - o If < 12 months of age: 3 dose series.
  - o If 12 months of age and older: 2 doses 8 weeks apart.
- o Pneumococcal polysaccharide vaccine at 2 years (at least 8 weeks after conjugate vaccine). Repeat vaccination as a booster 5 years after the initial dose.

- Annual influenza vaccination.

- Phenoxymethylpenicillin, oral, 12 hourly.
  - o < 5 years: 125 mg
  - o > 5 years: 250 mg
  - o Give indefinitely.

Treatment
Analgesia as required:
- Paracetamol, oral, 15 mg/kg 6 hourly.

AND
- Ibuprofen, oral, 10 mg/kg 8 hourly.

AND
- Codeine phosphate, oral, 0.5–1 mg/kg 4 hourly.

If this is inadequate, see section 20.1: Management of pain.

- Hydroxyurea, oral, 15 mg/kg.
  - o Increase by 5 mg/kg every12 weeks.
  - o Maximum dose: 35 mg/kg daily.

Infections:
All children < 6 months with axillary temperature ≥ 38°C **and** all children > 6 months:
- ▪ 3rd generation cephalosporins, e.g.:
- Cefotaxime, IV, 25–50 mg/kg/dose, 6 hourly (neonates).

OR
Children > 2 months:
- Ceftriaxone, IV, 50–80 mg/kg/dose once daily.

Acute chest syndrome:
Consult a paediatrician.

REFERRAL
» All children with sickle cell anaemia should be managed in consultation with a paediatric haematologist or paediatrician.
» All children with severe complications that may benefit from exchange transfusion or intensive care, e.g. stroke, severe vaso-occlusive disease and acute chest syndrome.

3.7 HAEMOPHILIA A AND B
D66.7/D66.8

DESCRIPTION
Haemophilia A and h aemophilia B are ch ronic bleeding disorders caused, respectively, by a lack of clotting factor VIII or clotting factor IX.

Sub classification of severity

Class	Clotting factor	% of normal	Signs
Mild	VIII or IX	5–25%	Occasional bleeds
Moderate	VIII or IX	1–5%	Less frequent bleeds post trauma/dental extraction
Severe	VIII or IX	<1%	Spontaneous joint and muscle bleeds

DIAGNOSTIC CRITERIA

Clinical

» Major bleeds:
- > CNS,
- > severe injury,
- > forearm compartment,
- > gastrointestinal tract, airway,
- > advanced joint and soft tissue,
- > hip and ilio-psoas.

» Minor bleeds:
- > early joint bleed,
- > soft tissue,
- > mouth and gum,
- > muscle,
- > epistaxis,
- > haematuria.

» Pain/tingling in a joint suggests bleeding in a known haemophiliac.

Investigations

» Prolonged partial thromboplastin time (PTT).
» Factor VIII or factor IX concentration < 25% of normal activity.

GENERAL AND SUPPORTIVE MEASURES

» Haemophilia register.
» Alert bracelet.
» Dental care (see below for management of tooth extraction).

Acute bleeds into joints

» Apply ice packs: 5 minutes on and 10 minutes off.
» Rest the affected joint/limb until pain free and no further bleeding.
» No weight bearing.
» Splint. Do not use circumferential casts.
» **Do not** aspirate affected joints.

MEDICINE TREATMENT

For pain (as required):
Do not use NSAIDs and aspirin.

- Paracetamol, oral, 15 mg/kg 6 hourly.

If needed:
ADD

- Codeine phosphate, oral, 0.5-1 mg/kg 4 hourly.

For bleeds
Emergency treatment while awaiting transfer, if indicated.
If serious bleeding in known haemophiliac, and no factor available:
- Lyophilised plasma, IV, 20 mL/kg. Lyophilised plasma contains a minimum of 0.4 units/mL of each coagulation factor.

OR
- Fresh frozen plasma, IV, 20 mL/kg.

Factor VIII deficiency (with no inhibitor present)
Give until patient is pain free and has full movement of joint/limb. Administration should be 12 hourly for major bleeds, but may be daily for minor bleeds.

Minor bleeds:
- Factor VIII, intravenous, 20 units/kg.

Major bleeds:
- Factor VIII, intravenous, 40 units/kg

Use the entire contents of the appropriate volume ampoule.

For intracranial bleeds:
- Factor VIII, intravenous**,** 40 units/kg 6 hourly.

Decrease frequency if trough level is > 60%, if possible.

Factor IX deficiency (with no inhibitor present)
Give daily until patient is pain free and has full movement of the joint/limb.

Minor bleeds
- Factor IX, intravenous, 20 units/kg.

Major bleeds:
- Factor IX, intravenous, 60 units/kg.

Available product is factor IX complex and also contains factors II, VII and X.

Home treatment
Home treatment of bleeds is promoted by haemophilia treatment centres. Patients or caregivers are educated on the storage, reconstitution and administration of factor and provided with a supply of factor to be kept at home for use in the event of a bleed. Factor use and bleeding episodes are monitored through the use of an appropriate chart which can be reviewed at consultations and medication collection.

Haemophilia with inhibitors
Refer for assessment and planning with a haematologist for use of factor VIII or IX at twice the usual dose or factor VIII inhibitor-bypassing activity (FEIBA) or recombinant Factor VIIa.

For dental extraction:
Check that inhibitors are absent.
Admit for procedure and post-procedure care and observation.

Haemophilia A:
- Factor VIII, intravenous, 40 units/kg, immediately before extraction.

AND
- Tranexamic acid, oral, 25 mg/kg/dose 6–8 hourly for 5 days.

Haemophilia B:
- Factor IX, intravenous, 40 units/kg, immediately before extraction.

For mucous membrane bleeds
- Tranexamic acid, oral, 25 mg/kg/dose 6–8 hourly.
  - o Contra-indicated in haematuria.
  - o Use with caution with factor IX complex or factor VIII inhibitor-bypassing activity and preferably only 12 hours after administration of the factor.

REFERRAL
» All cases with **suspected** or established haemophilia (prolonged PTT and normal INR), for assessment, genetic counselling and planning of management to a haemophilia treatment centre.

3.8 VON WILLEBRAND DISEASE
D68.0

DESCRIPTION
Von Willebrand disease is the most common congenital bleeding disorder and is due to reduced amounts or abnormal forms of von Willebrand factor in the circulation.

DIAGNOSTIC CRITERIA
Clinical
» Recurrent epistaxis, prolonged bleeding from lacerations, easy bruising or gum bleeds.

Investigations
» Reduction in one or more of the following:
  > von Willebrand factor antigen,
  > ristocetin co-factor and/or collagen binding activity,
  > factor VIII coagulant activity.

GENERAL AND SUPPORTIVE MEASURES

» Apply pressure to the bleeding site.
» For tooth socket bleeds bite down on a piece of gauze.
» For epistaxis, see section 17.3: Epistaxis (Nose bleeds).

Avoid aspirin and NSAIDS

MEDICINE TREATMENT

For mild bleeds:

- Desmopressin, IV, 0.3 mcg/kg in 5 0 mL sodium chloride 0.9% administered over 30 minutes.
    - o Repeat at 12 and 24 hours, if needed.
    - o Maximum: 3 doses.
    - o Only after a therapeutic trial has demonstrated efficacy in th e patient.

For severe bleeds:

- Factor VIII, intravenous (Factor VIII containing von Willebrand factor).
    - o Initial dose: 30 units/kg.

For mucous membrane bleeds:

- Tranexamic acid, oral, 25 mg/kg/dose 6–8 hourly.

For menorrhagia:

- Combined oral contraceptive, low dose.

REFERRAL

» All suspected cases of v on Willebrand disease to a h aemophilia treatment centre for assessment.

3.9 HAEMORRHAGIC DISEASE OF THE NEWBORN

P53

See section 19.4: Haemorrhagic disease of the newborn.

3.10 IDIOPATHIC THROMBOCYTOPAENIC PURPURA (ITP)

D69.3

DESCRIPTION

Common bleeding disorder of childhood due to the auto-immune destruction of platelets.

It occurs most frequently in children aged 2 to 5 years and often follows infection with viruses. Chronic ITP (more than 6 months duration) occurs in 10 to 20% of children with ITP.

Complications include severe haemorrhage and bleeding into vital organs.

DIAGNOSTIC CRITERIA

Clinical

» Sudden onset of bruising and bleeding, either spontaneously or after minor trauma, into the skin and mucous membranes and rarely into the organs in an otherwise well child.
» The lesions may range from pinpoint petechial bleedings to large ecchymoses, and are often increased on pressure points.
» Epistaxis is common.
» Exclude child abuse.
» The presence of the following makes the diagnosis of ITP unlikely:
  > splenomegaly,
  > hepatomegaly,
  > lymphadenopathy,
  > masses,
  > joint swelling,
  > bone pain,
  > rashes present other than petechiae or ecchymoses.

Investigations

» Thrombocytopaenia with normal white cell count and differential, and normal haemoglobin and red cell morphology, other than the effects of blood loss.
» Normal INR (PT) and partial thromboplastin time (PTT).
» Abundant megakaryocytes on bone marrow aspiration with normal erythroid and myeloid cellularity.
» A normal LDH and uric acid help to rule out leukaemia.
» Indications for bone marrow biopsy/aspiration: Prior to starting steroids, or any other abnormality on FBC or any atypical cells on differential count.
» Test all newly-diagnosed cases for HIV.

Follow up patients with diagnosis of ITP not confirmed with bone marrow aspiration for development of new clinical signs and abnormalities on laboratory investigations.

GENERAL AND SUPPORTIVE MEASURES

» Avoid:
  > platelet transfusions unless bleeds are life-threatening,
  > contact sport, injury and trauma, and
  > dental procedures in acute phase.
» Re-assure patient and family that resolution usually occurs.

MEDICINE TREATMENT

Avoid medication that affects platelet function, e.g. NSAIDs and aspirin.

Acute ITP

Active bleeding:

- Prednisone, oral, 4 mg/kg/dose as a single daily dose for 4 days.
  - Stop after 4 days without tapering dose.

Chronic ITP

Intermittent treatment if platelets ≤ 10 x 10^9/L **and** significant bleeding episodes:

- Prednisone, oral, 4 mg/kg/dose as a single daily dose for 4 days.
  - Stop after 4 days without tapering dose.

Acute life-threatening bleeds (e.g. intracranial bleeding): (acute or chronic ITP)

- Methylprednisolone, IV, 30 mg/kg/dose administered over 30–60 minutes as a single daily dose for 3 days.
  - Maximum dose: 1 g.
  - Beware of arrhythmias, hypertension, etc.
  - Check BP daily.

AND

- Immunoglobulin, human normal, IV, 1 g/kg/day for 2 days.

Note:

Immunoglobulins are reserved for the management of acute life threatening bleeds.

AND

After administration of methylprednisolone:

- Platelets, IV.

Consider emergency splenectomy.

SURGICAL TREATMENT

Consider splenectomy in children 5 years and older with chronic ITP for more than one year plus significant bleeding or substantial limitation in activities as a result of the ITP.

<u>Pre-splenectomy</u>

Children who have been vaccinated with PCV 7 as part of their routine should receive PCV 10 or 13 eight weeks before polysaccharide vaccine.

- Pneumococcal vaccine (polysaccharide), IM, 0.5 mL as a single dose.

Post splenectomy

- Phenoxymethylpenicillin, oral, 12 hourly.
  - If < 5 years of age: 125 mg.
  - If > 5 years of age: 250 mg.
  - Give indefinitely until at least until 18 years.
- Pneumococcal polysaccharide vaccine. Repeat vaccination as a booster 5 years after the initial dose.
- Annual influenza vaccine.
- Catch up conjugate pneumococcal vaccine:
  - If < 12 months of age: 3 dose series.
  - 12 months of age and older: 2 doses 8 weeks apart.

REFERRAL

» Suspected ITP with unusual features such as splenomegaly or lymphadenopathy.
» ITP complicated by severe haemorrhage, bleeding into vital organs or an intracranial haemorrhage.
» ITP that fails to resolve in 6–12 months on adequate treatment (chronic ITP).
» If there is no local capacity to fully investigate the condition.

3.11 VENOUS THROMBO-EMBOLIC DISEASE

I82

DESCRIPTION

The occurrence of an occlusive or non-occlusive thrombus in the venous circulation and/or consequent pulmonary embolus. It is usually associated with risk factors such as the presence of central venous catheters, venous stasis, endothelial damage and hypercoagulable states due to various causes, e.g. nephrotic syndrome.

DIAGNOSTIC CRITERIA

Clinical

Depends on the site of thrombosis but may be silent.

» Deep venous thrombosis of an extremity presents with unilateral limb swelling.
» Upper extremity thrombus may present with associated facial and neck oedema.
» Pulmonary embolus presents with sudden onset of shortness of breath and chest pain.
» Cerebral sinus venous thrombosis presents with seizures or other neurological symptoms and signs.
» Renal vein thrombosis presents with haematuria and thrombocytopenia and oliguria and renal failure if bilateral.

Investigations

» Doppler ultrasonography, CT scan or MRI demonstrate thrombosis or embolus.
» D-dimer, antithrombin III, protein C, protein S, Factor V Leiden and antiphospholipid antibody testing may reveal underlying thrombophilia.

GENERAL AND SUPPORTIVE MEASURES

» Appropriate fluid restriction and electrolyte management if renal failure.

MEDICINE TREATMENT

If hypoxic:

- Oxygen by face mask.

- Heparin, unfractionated, IV, administered over 10 minutes as a bolus followed by an initial maintenance dose as a continuous infusion.

	**Bolus**	**Initial maintenance dose**
Preterm neonates	25–50 units/kg	15 units/kg/hour
Term neonates	75–100 units/kg	28 units/kg/hour
Children	75–100 units/kg	20 units/kg/hour

Neonates need a higher maintenance dose per body weight compared with older children.

<u>Target levels</u>

PTT: 60–85 seconds or 2–3 times the baseline value (if normal for age).
Monitor PTT 4 hours after bolus injection and adjust the continuous IV dose according to the result (See table below)

Nomogram for adjusting UFH dose*

PTT (seconds)	Bolus (units/kg)	Hold infusion (minutes)	Dose change	Repeat PTT (hours)
<50	50	0	Increase by 20%	4
50–59	0	0	Increase by 10%	4
60–85	0	0	No change	24
86–95	0	0	Decrease by 10%	4
69–120	0	30	Decrease by 10%	4
>120	0	60	Decrease by 15%	4

*the sensitivity of the PTT towards UFH depends on the reagent used.
Maintain PTT 2.5–3.5 times the control.
Discontinue heparin once therapeutic INR is achieved with warfarin.

Table reproduced from Venous thromboembolism: Prophylactic and therapeutic practice guideline. S Afr Med J 2013;103(4):260-267
Published with kind permission.

AND

- Warfarin, oral, 0.1 mg/kg daily from day 1.
  - o Target INR: 2–3.
  - o Continue warfarin therapy for 3–6 months if no underlying severe thrombophilia.
  - o Inherited thrombophilic conditions may need lifelong therapy.
  - o Beware of drug interactions

Weight	Starting dose of warfarin
10–20 kg	2.5 mg alternate days
20–35 kg	2.5 mg daily
35–50 kg	2.5 mg alternating with 5 mg daily
50 kg+	5 mg daily

Adjust schedules using combinations of 2.5 mg and 5 mg **or** 5 mg and 7.5 mg **or** 7.5 mg and 10 mg if a standard daily dose does not provide a therapeutic INR. For example: 2.5 mg Monday, Wednesday, Friday and 5 mg Tuesday, Thursday, Saturday, Sunday.

REFERRAL

» All patients to an appropriate centre for diagnostic imaging.

» Long term management of thrombophilic states should be in consultation with a paediatric haematologist or paediatrician.

3.12 SPECIAL CONSIDERATIONS IN HIV INFECTED CHILDREN

In addition to the usual causes of blood disorders in childhood, HIV infected children are at increased risk of developing anaemia, thrombocytopaenia and neutropaenia secondary to drugs (especially zidovudine in the case of anaemia), opportunistic infections or neoplasms. They are also at increased risk of thrombo-embolic disease secondary to vasculopathy or the induction of a thrombophilic state.

3.12.1 THROMBOCYTOPAENIA

D69.6

DESCRIPTION

Most cases of thrombocytopaenia in children with HIV infection are due to immune thrombocytopaenic purpura

Exclude other causes of thrombocytopaenia if the diagnosis is made clinically.

DIAGNOSTIC CRITERIA

Clinical

- » Bleeding tendency in a child with HIV infection.
- » Asymptomatic finding on full blood count.

Investigations

- » Thrombocytopaenia with normal white cell count and red cell indices, apart from the effects of blood loss.
- » Normal INR (PT) and partial thromboplastin time (PTT).
- » Abundant megakaryocytes on bone ma rrow aspiration with normal erythroid and myeloid cellularity.
- » Indications for bone marrow investigation: Prior to starting steroids or any other abnormality on FBC or any atypical cells on differential count.

GENERAL AND SUPPORTIVE MEASURES

- » As for the HIV uninfected child.
- » Avoid:
  - > platelet transfusions, unless life-threatening bleeds;
  - > contact sport, injury and trauma;
  - > dental procedures in acute phase;
  - > medication that affects platelet function, e.g. NSAIDs and aspirin.
- » Check for interactions with ARTs.

MEDICINE TREATMENT

As for the HIV uninfected child.

Initiate ART if not already initiated.

Acute ITP

Active bleeding:

- Prednisone, oral, 4 mg/kg/24 hours as a single daily dose for 4 days.

REFERRAL

- » All children with refractory symptomatic thrombocytopaenia.

CHAPTER 4

CARDIOVASCULAR SYSTEM

4.1 CARDIAC DYSRHYTHMIAS

I49.9

DESCRIPTION

A heart rate that is abnormally slow or fast for age or irregular.

Normal heart rate/minute for age:

Newborn	100–160
< 1 year	110–160
1–2 years	100–150
2–5 years	95–140
5–12 years	80–120
> 12 years	60–100

DIAGNOSTIC CRITERIA

Clinical

» Presenting features may vary with the age of the patient:
- > infants:
  - colour changes (pale, mottled),
  - irritability,
  - feeding difficulties,
  - sweating,
  - tachypnoeic/apnoeic spells.
  - irregular pulse,
  - tachycardia,
  - bradycardia,
  - signs of cardiac failure,
- > children:
  - dizziness,
  - palpitations,
  - fatigue,
  - chest pain,
  - tachycardia,
  - bradycardia,
  - syncope,
  - signs of cardiac failure.

Investigations

» ECG is essential for diagnosis, preferably a 12 lead ECG.
Monitors are inadequate to diagnose most dysrhythmias.

TACHYDYSRHYTHMIA

Sinus tachycardia

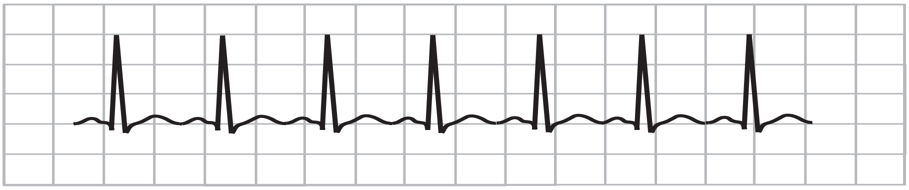

ECG Criteria

Rate: > upper limit for age

Rhythm: regular

P wave: present and normal

QRS: normal

Supraventricular tachycardia

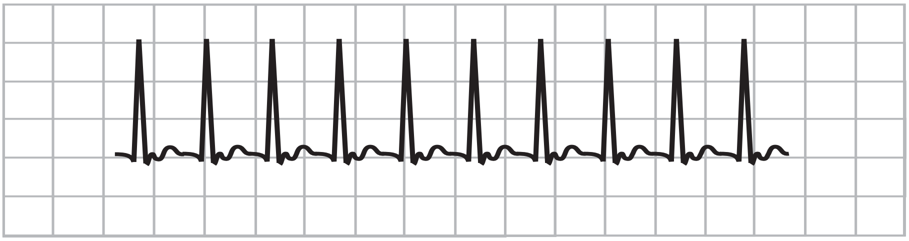

ECG Criteria

Rate: usually > 200 beats per minute

Rhythm: regular

P wave: abnormal

QRS: normal

Ventricular tachycardia

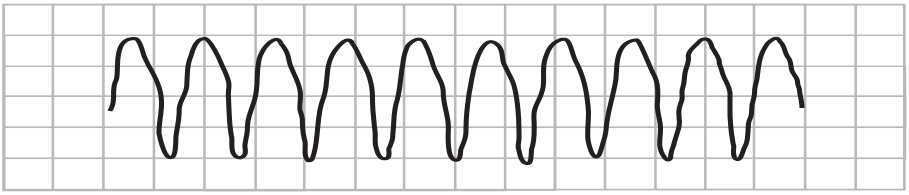

ECG Criteria

Rate: generally 100–220 beats per minute

Rhythm: generally regular

P wave: mostly not seen

QRS: abnormal, width of QRS > 120 millisecond

BRADYDYSRHYTHMIA

Common causes:

- hypoxia
- congenital
- drug ingestion
- excessive vagal stimulation

Sinus bradycardia

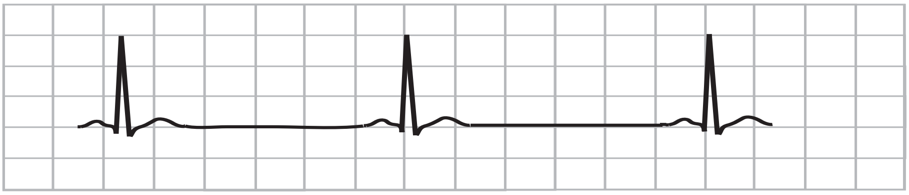

ECG Criteria

Rate: < lower limit for age

Rhythm: regular

P wave: present, all look the same

QRS: normal, 80–120 millisecond

Heart block (Complete)

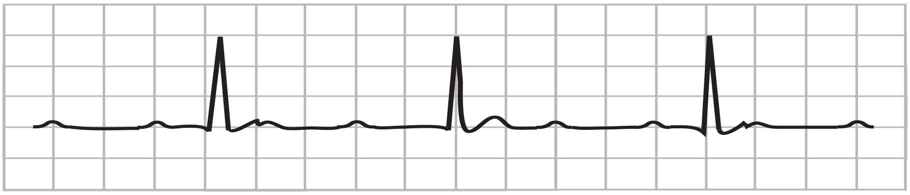

ECG Criteria

Rate: low, usually < 60 beats per minute

Rhythm: regular

P wave: independent P waves and QRS's with no relationship between the two (AV dissociation)

QRS: can be normal or wide, depending on escape rhythm

GENERAL AND SUPPORTIVE MEASURES

» Sinus tachycardia usually requires management of the underlying condition.
» Apply ABC of resuscitation.
» Admit to high care or intensive care unit.
» Monitor:
  > ECG,
  > blood pressure,
  > heart rate,
  > respiratory rate,
  > oxygen saturation,
  > haemoglobin,
  > acid–base status,
  > blood gases.
» Maintain adequate nutrition and hydration.
» Treat pyrexia.

MEDICINE TREATMENT

TACHYDYSRHYTHMIAS

Emergency treatment.

Narrow complex tachycardia

Commonly due to supraventricular tachycardia.

Stable patient:

Attempt vagal stimulation.

Place ice bag on face, or

Infants: immerse face in ice-cold water for a few seconds.

Older children: try a Valsalva manoeuvre.

Eye-ball pressure and carotid massage is contraindicated in children.

- Adenosine, IV, 0.1 mg/kg rapid IV push (within seconds).
  - Follow immediately with a rapid flush of at least 5 mL sodium chloride 0.9%.
  - Increase dose in 0.1 mg/kg increments every 2 minutes until return of sinus rhythm. Follow each dose with a rapid flush of sodium chloride 0.9%.
  - Maximum dose: 0.5 mg/kg. Do not exceed 12 mg in total.
  - Consult with a cardiologist/paediatrician before administration.

  Because adenosine is rapidly metabolised, inject adenosine in a good drip, followed with a rapid flush of a fluid bolus. It is sometimes helpful to have both the syringe with adenosine and the fluid bolus connected to the giving set and having as short as possible line between the syringes and the patient.

Unstable patient – heart failure / shocked:

DC synchronised cardioversion in increments of 1–2 J/kg.

If possible, empty the stomach before cardioversion is attempted.

Resuscitation facilities must be available.

Midazolam for sedation, if necessary.

Broad complex tachycardia

Commonly due to ventricular tachycardia.

Causes include electrolyte disturbances and drug ingestion.

Stable patient (rare):

Send ECG immediately to paediatric cardiologist.

If unsure whether narrow or wide angle tachycardia, attempt adenosine as in narrow complex tachycardia.

<u>Unstable patient – heart failure/shock:</u>

Pulseless treat as ventricular fibrillation.
DC asynchronised cardioversion in increments of 1–2 J/kg.
Resuscitation facilities must be available.
Midazolam for sedation, if necessary.

Monitor and correct blood gases and acid-base status.

If DC cardioversion fails:

- Amiodarone, IV, 5 mg/kg slowly over 20 minutes – **never** as a rapid infusion.

And continue with DC cardioversion.

BRADYDYSRHYTHMIAS

Try and correct underlying causes.

<u>Stable patient:</u>
Observe.

Bradydysrhythmia due to vagal stimulation:

- Atropine, IV/IO, 0.02 mg/kg.
  - o If no response, repeat in 5 minutes.

<u>Unstable patient:</u>
Treat as impending arrest:

- Epinephrine (adrenaline), IV/IO, 0.01 mg/kg.
  - o Repeat if necessary conferring with referral institution.

If no sustained response, consider:

- Epinephrine (adrenaline), IV infusion, 0.05–2 mcg/kg/minute.

REFERRAL

» All children with tachydysrhythmias after acute treatment, excluding sinus tachycardia due to other causes.
» Bradycardia unresponsive to medical treatment, or heart block.

4.2 CYANOTIC CONGENITAL HEART DISEASE WITH HYPOXAEMIC ATTACKS/SPELLS (HYPERCYANOTIC SPELLS)

Q24.9

DESCRIPTION

Acute worsening of central cyanosis in patients with a co nfirmed or suspected underlying cyanotic congenital heart disease such as Tetralogy of Fallot after the neonatal period.

DIAGNOSTIC CRITERIA

Clinical

» Rapid worsening of central cyanosis, tachypnoea/dyspnoea, anxiety and alteration in consciousness in the presence of congenital cyanotic heart disease.
» Restless and crying in the presence of congenital cyanotic heart disease.
» Decrease in intensity or disappearance of the systolic murmur in Tetralogy of Fallot during crying.

GENERAL AND SUPPORTIVE MEASURES

» Exclude and treat precipitants such as fever, infection and gastroenteritis.
» Calm patient and keep on mother’s lap, if possible.
» Place patient in knee-chest position to raise systemic blood pressure and increase systemic venous return.
» Monitor SaO_2, heart rate, respiratory rate and acid-base status.
» Ensure adequate hydration.

MEDICINE TREATMENT

- Oxygen, 100%, by facemask or by nasal cannula
- Volume expander e.g. sodium chloride 0.9%, IV bolus, 20mL/kg administered over 5 minutes.
- Morphine, IV, 0.1–0.2 mg/kg as a single dose.
  - o May cause impairment of airway reflexes and respiratory depression.

If clinically acidotic or pH < 7.2:

- Sodium bicarbonate 4.2%, IV, 2 mL/kg.

If failure to improve the cyanotic spell, consider in consultation with specialist:

- Ketamine, IV, 0.5–1 mg/kg.

Note: IV ketamine is a general anaesthetic.<br>Take standard precautions for respiratory arrest.

After resolution of spell:
If Hb < 10 g/dL, child is anaemic:

- Packed red cells, 10 mL/kg administered over 3 hours.

- Propranolol, oral, 0.5–1 mg/kg/dose 6 hourly.
  - o Do not exceed 5 mg/kg/day.

REFERRAL

» If above measures do not work refer urgently for consideration of phenylephrine, ventilation and emergency surgery.
» All cases for assessment.

4.2.1 TETRALOGY OF FALLOT

Q21.3

DESCRIPTION

Suspect tetralogy of Fallot in a child with cyanosis after the neonatal period.

DIAGNOSTIC CRITERIA

Clinical

» Child with central cyanosis.
» May be plethoric due to polycythemia – normal haemoglobin represents relative anaemia.
» May have clubbing.
» Possible history of cyanotic spells.
» Heart not clinically enlarged.
» Right ventricular hypertrophy usually not palpable.
» Single second heart sound.
» Coarse, ejection systolic murmur over right ventricular outflow tract.
» Chest X-ray:
  > normal/small heart,
  > boot shaped/pulmonary bay – concavity where pulmonary artery should be,
  > oligaemic lung fields.
» ECG:
  > right axis deviation and right ventricular hypertrophy.

GENERAL AND SUPPORTIVE MEASURES

» Good dental hygiene.

MEDICINE TREATMENT

- Iron (elemental), oral, 1 mg/kg/dose 8 hourly.

- Folic acid, oral, 2.5– 5 mg/day.

- Propranolol, oral, 0.5–1 mg/kg/dose 6 hourly.
  - o Do not exceed 5 mg/kg/day.

Endocarditis prophylaxis:
See section 4.3: Endocarditis, infective.

REFERRAL

» All children with cyanotic heart defects.

4.3 ENDOCARDITIS, INFECTIVE

I33.0

DESCRIPTION

Infection of the endothelial surface of the heart.
Suspect infective endocarditis in all children with persistent fever and underlying heart disease.

DIAGNOSTIC CRITERIA

Clinical

» An underlying heart defect and a persistent low grade fever without an obvious underlying cause.
» Associated other findings include: fatigue, joint pain, new murmurs, clubbing, splenomegaly and haematuria.
» Must be differentiated from acute carditis due to rheumatic fever.
» The Duke criteria have been suggested as a guide to diagnosis, but have definite limitations as they were developed for use in adult patients.

Table 1: Major and minor clinical criteria used in the modified Duke criteria for diagnosis of infective endocarditis (IE)

MAJOR CRITERIA	MINOR CRITERIA
» Positive blood culture:<br>> typical micro-organisms from two separate blood cultures: *S. viridans*, including nutritional variant strains, *S. bovis,* *HACEK group, *S. aureus,* or<br>> Enterococci, in the absence of a primary focus, or<br>> persistently positive blood culture with a micro-organism consistent with IE from blood cultures drawn > 12 hours apart, or<br>> all 3 or a majority of 4 or more separate blood cultures, with the first and last drawn at least one hour apart, or<br>> positive serology for Q fever<br>> single positive blood culture for *Coxiella burnetti* or anti-phase 1 IgG antibody titre > 1:800.<br><br>» Evidence of endocardial involvement:<br>> positive echocardiogram for IE (transoesophageal echocardiography is recommended for patients with prosthetic valves): oscillating intracardiac mass, on valve or supporting structures, or in the path of regurgitant jets, or on implanted materials, in the absence of an alternative anatomic explanation, or<br>> abscess, or<br>> new partial dehiscence of prosthetic valve, or<br>> new valvular regurgitation.	» Predisposing heart condition or IV drug use<br><br>» Fever ≥ 38ºC.<br><br>» Vascular phenomena:<br>> major arterial emboli,<br>> septic pulmonary infarcts,<br>> mycotic aneurysm,<br>> intracranial haemorrhage,<br>> conjunctival haemorrhages,<br>> Janeway lesions.<br><br>» Immunologic phenomena:<br>> Osler's nodes,<br>> Roth spots,<br>> glomerulonephritis,<br>> rheumatoid factor.<br><br>» Microbiologic evidence:<br>> positive blood culture but not meeting major criterion, or<br>> serologic evidence of active infection with organism consistent with IE.

*A group of fastidious gram negative organisms originating in the mouth.

Table 2: Modified Duke criteria for diagnosis of infective endocarditis (IE)

DEFINITE IE	POSSIBLE IE	REJECTED
Pathological criteria<br>» Micro-organisms<br>> by culture or histology in a vegetation, or<br>> in a vegetation that has embolised, or<br>> in an intracardiac abscess, or<br><br>Lesions<br>» Vegetation or intracardiac abscess present – confirmed by histology showing active IE.<br><br>Clinical criteria – see Table 1<br>» 2 major criteria,<br>» 1 major and 3 minor, or<br>» 5 minor.	» At least one major and one minor criterion, or<br>» 3 minor.	» Alternative diagnosis for manifestation of endocarditis, or<br>» resolution of manifestations, with antibiotic therapy ≤ 4 days, or<br>» no pathologic evidence of IE at surgery or autopsy, after antibiotic therapy for ≤ 4 days.

<u>Limitations of the Duke Criteria in children</u>
The clinical criteria rely heavily on relatively rare clinical features.
In contrast, common clinical features like splenomegaly, clubbing and haematuria have not been included.
Investigations like CRP or ESR, which may be of value, have also not been included.

Investigations

» Blood cultures:
  > Sterile blood culture technique is essential.
  > Take three blood cultures (venous) from different sites within 2 hours if very ill, otherwise over 24 hours. There is little benefit of doing more than five blood cultures.
  > Child does not necessarily have a temperature as patients are mostly constantly bacteraemic.
» Urine test strips – haematuria.
» CRP/ESR may be helpful.

GENERAL AND SUPPORTIVE MEASURES

» Bed rest/limit physical activity.
» Ensure adequate nutrition.
» Maintain haemoglobin > 10 g/dL.
» Measures to reduce fever.

MEDICINE TREATMENT

See section 4.9: Heart failure.

For fever:

- Paracetamol, oral, 15 mg/kg/dose, 6 hourly as required.

Antibiotic therapy

Antibiotics are **always** given IV, according to culture and sensitivity.
If culture is available treat according to sensitivities.

Empiric treatment

If culture is not yet available or is negative:

- Benzylpenicillin (Penicillin G), IV, 50 000 units/kg/dose, 6 hourly for 4 weeks.

PLUS

- Cloxacillin, IV, 50 mg/kg/dose 6 hourly for 4 weeks.

PLUS

- Gentamicin, IV, 3 mg/kg/day for 4 weeks.

If culture available:

S. viridans

- Benzylpenicillin (Penicillin G), IV, 50 000 units/kg/dose, 6 hourly for 4 weeks.

PLUS

- Gentamicin, IV, 3 mg/kg/day for 2 weeks.

Enterococci

- Benzylpenicillin (Penicillin G), IV, 75 000 units/kg/dose, 6 hourly for 4–6 weeks.

PLUS

- Gentamicin, IV, 3 mg/kg/day for 4–6 weeks.

Cloxacillin sensitive staphylococcus

- Cloxacillin, IV, 50 mg/kg/dose 6 hourly for 4–6 weeks.

If the organism is gentamicin sensitive:

ADD

- Gentamicin, IV, 3 mg/kg/day for 3–5 days.

<u>Multi-Resistant Staphylococcus Aureus (MRSA)</u>

- Vancomycin, IV, 10 mg/kg/dose infused over 1 hour, 6 hourly for 6 weeks.

<u>HACEK organisms</u>

- Ceftriaxone, IV, 100 mg/kg once daily **or** ampicillin, IV, 50 mg/kg/dose 6 hourly for 4 weeks.

PLUS

- Gentamicin, IV, 3 mg/kg/day for 4 weeks.

<u>Enteric bacilli, e.g. Klebsiella</u>

- Piperacillin, IV, 50 mg/kg/dose **or** ceftazidime, IV, 50 mg/kg/dose 6 hourly for 6 weeks.

PLUS

- Gentamicin, IV, 1 mg/kg/dose 8 hourly for 6 weeks.

Prophylaxis

The use of prophylaxis is controversial but still recommended.

For children with the following cardiac conditions:

» rheumatic heart disease;
» prosthetic cardiac valve or prosthetic material used in valve repair;
» previous infective endocarditis;
» unrepaired cyanotic heart disease, including palliative shunts;
» during the first 6 months after complete repair of congenital heart defect with prosthetic material or device (complete endothelialisation of prosthesis after 6 months);
» repaired cyanotic heart disease with residual defect at or adjacent to prosthetic patch or device; or
» cardiac transplant recipients who develop cardiac valvulopathy.

Children with the above cardiac conditions should receive prophylaxis when undergoing the following procedures:

» All dental procedures that involve manipulation of gingival tissues or periapical region of teeth or trauma to oral mucosa.
» Procedures on respiratory tract or infected skin, skin structures or musculoskeletal tissue.

Regimens for dental procedures

- Amoxicillin, oral, 50 mg/kg (maximum 2 g) 1 hour before the procedure.

Patients unable to take oral medication:

- Ampicillin, IV, 50 mg/kg (maximum 2 g) ½ hour before the procedure.

REFERRAL

» All patients with suspected and confirmed infective endocarditis as soon as possible.

4.4 RHEUMATIC FEVER, ACUTE

I01.9

* Notifiable condition.

DESCRIPTION

Rheumatic fever is a common cause of acquired heart disease with significant morbidity and mortality rates, both in the acute phase of the disease and as a result of chronic valvular sequelae.

DIAGNOSTIC CRITERIA

Revised Jones criteria:

» Evidence of recent streptococcal infection:
  > Elevated ASO-titre or other streptococcal antibody titres.
  > Positive throat culture for group A beta haemolytic streptococcus.

  PLUS

» Two major manifestations, **or** one major and two minor manifestations, justifies the presumptive diagnosis of acute rheumatic fever.

Major manifestations	Minor manifestations
▪ polyarthritis<br>▪ carditis<br>▪ erythema marginatum<br>▪ subcutaneous nodules<br>▪ Sydenham's chorea	▪ polyarthralgia<br>▪ fever<br>▪ acute phase reactants: increased erythrocyte sedimentation rate (ESR) or C-reactive protein (CRP)<br>▪ ECG: prolonged PR-interval, $\geq$ 0.18 seconds in the absence of carditis

> Chorea for which other causes have been excluded, provides adequate evidence of rheumatic fever without the other criteria for diagnosis being required.
> In children with rheumatic heart disease with fever, it is critical to differentiate recurrence of acute rheumatic fever from infective endocarditis.

For children with rheumatic heart disease, recurrence of some of the above criteria would suggest a recurrence of rheumatic fever but other causes such as IE should be excluded.

GENERAL AND SUPPORTIVE MEASURES

» Hospitalise with bed rest until sleeping pulse is normal and signs of rheumatic activity have resolved.
» Restrict physical activity for at least 2 weeks after acute phase reactants have normalised.
» Keep a record of the patients on rheumatic fever prophylaxis so that attendance can be monitored.

MEDICINE TREATMENT

Antibiotic therapy

To eradicate any streptococci:

- Benzathine benzylpenicillin (depot formulation), IM, as a single dose.
  - o If < 30 kg: 600 000 IU.
  - o If ≥ 30 kg: 1.2 MU.

OR

- Phenoxymethylpenicillin, oral, 250–500 mg 12 hourly for 10 days.

Anti-inflammatory therapy

Do not start until a definite diagnosis is made.

Severe arthritis:

- Aspirin soluble, oral, 20 mg/kg/dose 6 hourly until the arthritis resolves.

OR

If aspirin cannot be tolerated:

- Ibuprofen, oral, 5 mg/kg/dose, 6 hourly.

Cardiac failure: See section 4.9: Heart failure.

Chorea: See section 13.10: Sydenham's Chorea.

Prevention of repeated attacks

Any patient with documented rheumatic fever must receive prophylaxis.
Intramuscular penicillin is superior to other forms of prophylaxis.

- Benzathine benzylpenicillin (depot formulation), IM, every 21 days.
  - o If < 30 kg: 600 000 IU.
  - o If > 30 kg: 1.2 MU.

OR

- Phenoxymethylpenicillin, oral, 250 mg 12 hourly.

Continue therapy until patients reach 35 years of age.

REFERRAL

Rheumatic fever:

» with residual valvular damage electively for planning of care,
» with symptomatic valvular damage,
» unresponsive to treatment.

4.5 MYOCARDITIS

I40

DESCRIPTION

Myocarditis is an acute inflammation of the cardiac muscle. The majority of paediatric myocarditis cases are secondary to a viral infection. Viral myocarditis should be suspected whenever a child presents with unexplained shortness of breath, dysrhythmia or acute heart failure following a viral illness.

DIAGNOSTIC CRITERIA

Clinical

- » Tachycardia.
- » Clinical signs of biventricular heart failure.
- » May present with cardiogenic shock.

Investigations

- » ECG changes are non-specific but ST elevation, T wave inversion, prolonged QTc, small complexes, dysrhythmias or extra-systole may be seen.
- » Chest X-ray:
  - > pulmonary congestion,
  - > cardiomegaly,
  - > possible pleural effusion.
- » Elevated cardiac troponin T levels are a good indicator of myocarditis.

GENERAL AND SUPPORTIVE MEASURES

- » Restrict fluid (75% of daily requirements) – not at expense of adequate caloric intake.
- » Ensure adequate nutrition, tube-feeding may be necessary.

MEDICINE TREATMENT OF VIRAL MYOCARDITIS

To prevent hypoxia:

- Oxygen via face mask, nasal cannula or head box.

- Furosemide, oral/ IV, 1 mg/kg/dose 12 hourly.

Consider low dose inotropic support – Refer to ICU.

REFERRAL

- » Viral myocarditis not responding to treatment.

4.6 DILATED CARDIOMYOPATHY

I42.0

DESCRIPTION

Dilated cardiomyopathy refers to a group of conditions of diverse aetiology in which both ventricles are dilated with reduced contractility. It is difficult and sometimes impossible to distinguish myocarditis from dilated cardiomyopathy. A wide variety of disease can mimic dilated cardiomyopathy, but in the majority of cases no identifiable cause is found. One of the common causes of cardiomyopathy is HIV disease.

DIAGNOSTIC CRITERIA

Clinical

- » Cardiomegaly with clinical signs of heart failure and poor apical impulse.
- » May present with cardiogenic shock.

Investigations

- » Chest X-ray:
  - > pulmonary congestion,
  - > cardiomegaly,
  - > there may be pleural effusion.
- » ECG:
  - > Mostly non-specific.
  - > Dysrhythmias or extra-systoles may occur.

GENERAL AND SUPPORTIVE MEASURES

- » Fluid restriction (75% of daily requirements) – not at expense of adequate caloric intake.
- » Ensure adequate nutrition, tube-feeding may be necessary.
- » Advise bed rest.

MEDICINE TREATMENT

To prevent hypoxia:

- Oxygen via face mask, nasal cannula or head box.

- Furosemide, IV/oral, 1–3 mg/kg/24 hours in 2–3 divided doses.

AND/OR

- Spironolactone, oral, 2–4 mg/kg/24 hours in 2 divided doses.

- Digoxin, oral, 0.005 mg/kg/dose 12 hourly.
  - o Syrup: 0.005 mg = 0.1 mL, therefore 0.1 mL/kg/dose 12 hourly.
  - o In older children a once daily dose can be given, i.e. 0.01 mg/kg/day.

Persistent cardiac failure:

- Captopril, oral.
  - o Initial dose: 0.5 mg/kg/24 hours in 3 divided doses (8 hourly) for 24–48 hours.
  - o Increase by 0.5 mg/kg/24 hours every 24–48 hours until maintenance dose of 3–5 mg/kg/24 hours is reached.

Inotropic support. – Refer to ICU.

REFERRAL

» **Urgent:** To ICU for inotropic support.
» All patients for assessment and consideration of underlying disorders.

4.7 PERICARDIAL EFFUSION

I30

DESCRIPTION

Accumulation of fluid in the pericardial space, usually secondary to pericarditis.

DIAGNOSTIC CRITERIA

Clinical

» Most patients present with a prolonged history of:
  > low cardiac output,
  > distended neck veins,
  > muffled or diminished heart sounds.
» Patients with HIV may be a symptomatic and incidentally diagnosed when having a chest X-ray.
» Often associated with TB.
» Acute septic pericarditis may occur in patients with septicaemia.

Investigations

» Exclude TB in all cases. Tuberculin skin test.
» ECG:
  > small complexes tachycardia,
  > diffuse T wave changes,
» Chest X-ray:
  > in pericardial effusion – “water bottle” large globular heart or cardiac shadow with smoothed out borders.
» Ultrasound of heart and pericardium.
» Diagnostic pericardiocentesis:
  > in all patients with suspected bacterial or neoplastic pericarditis, and in all others in whom the diagnosis is not readily obtained;
  > include cell count and differential, culture and gram stain;
  > an elevated adenosine deaminase (ADA) may be helpful in diagnosing TB.

CARDIAC TAMPONADE

Cardiac tamponade is the accumulation of pericardial fluid that restricts filling and ejection of blood. The child usually presents with a tachycardia, pulsus paradoxus, elevated JVP, hypotension, shock or pulseless electric activity.

Features on ECG include electrical alternans, low voltage QRS.
Diagnosis is confirmed on sonar.

GENERAL AND SUPPORTIVE MEASURES

Urgent pericardiocentesis under ultrasound guidance by an experienced person.

Pericardiocentesis

- Do a coagulation screen if coagulation problems are suspected.
- Preferably under ultrasound guidance by an experienced person.
- In an emergency, drainage by using a large bore intravenous cannula.
- Technique:
  - Ensure that full resuscitation equipment is available as well as an IV line and cardiac monitor.
  - Administer oxygen via face mask, nasal cannula or head box.
  - If the patient is restless, it may be necessary to sedate the patient. In an emergency situation, this is unnecessary.
  - Position the patient in a 30° sitting-up position.
  - Prepare the preferred site just to the left of the xiphoid process, 1 cm inferior to the costal margin.
  - Infiltrate this area with 1% lidocaine (lignocaine).
  - Maintaining negative pressure on the syringe, insert the needle at a 45° angle to the skin, advancing in the direction of the patient's left shoulder.
  - While advancing the needle, observe closely on ECG for ventricular ectopic beats, a sign of myocardial contact, while advancing the needle. If this is noted, gradually withdraw the needle 1–2 cm.
  - Once air (pneumopericardium) or fluid begins to fill the syringe, advance the intravenous cannula, withdraw the needle, attach the syringe to the hub of the cannula and slowly aspirate the pericardial fluid.
  - Potential complications include: haemopericardium (from laceration of the heart wall or coronary artery), cardiac dysrythmias, pneumothorax, and pneumopericardium.

In an emergency, drainage by using a large bore intravenous cannula may be undertaken without sedation.

MEDICINE TREATMENT

Exclude TB in all cases with pericardial disease, and if suspected TB, give antituberculosis drugs for 6 months plus steroids:

- Prednisone, oral, 2 mg/kg/day for 4 weeks.

Pain management
See section 20.1: Management of pain.

Antibiotic therapy
Empiric antibiotic treatment until culture and sensitivity results are available. Antibiotic therapy should be continued for 3–4 weeks.

In case of purulent pericarditis:

- Cloxacillin, IV, 50 mg/kg/dose 6 hourly.

PLUS

- Ceftriaxone, IV, 100 mg/kg as a single daily dose.

Cardiac failure:
See section 4.9: Heart failure.

REFERRAL

» All after stabilisation of patient.

4.8 PERICARDITIS

I31.9

DESCRIPTION

Causes can be viral or bacterial. The commonest cause is TB.

Inflammation of the pericardium:

» Classical presentation of viral pericarditis, loud pericardial rub and chest pain that is relieved by sitting up. Children often do not complain of chest pain.
» Acute septic pericarditis may occur in patients with septicaemia.

TB pericarditis
TB pericarditis may present as pericardial effusion (most cases), effusive constrictive pericarditis or constrictive pericarditis.
Clinical features include:

» chronic cough,
» chest pain,
» night sweats,
» and weight loss.
» dyspnoea,
» fever,
» orthopnoea,

Severe pericardial pain is uncommon.

Investigations

» Exclude TB.
» Echocardiogram.

MEDICINE TREATMENT

Treat the cause.
Exclude TB in all pericardial disease cases. If TB is suspected, give antituberculosis drugs for 6 months.

▪ Corticosteroids:
- Prednisone, oral, 2 mg/kg/day for 4 weeks.

PLUS

▪ NSAID, e.g.:
- Ibuprofen, oral, 5 mg/kg per dose 6 hourly.

As soon as cultures are proved negative for organisms that can cause purulent pericarditis, TB treatment for 6 months.

Pain management:
See section 20.1: Management of pain.

Antibiotic therapy

Empiric therapy should be provided until culture and sensitivity results available.
Antibiotic therapy should be continued for 3–4 weeks.

Empiric therapy in case of purulent pericarditis:
- Cloxacillin, IV, 50 mg/kg/dose 6 hourly.

PLUS

- Ceftriaxone, IV, 100 mg/kg as a single daily dose.

Adjust antibiotic based on culture results.

REFERRAL

» All patients.

4.9 HEART FAILURE

I50.9

DESCRIPTION

Clinical syndrome reflecting the inability of the myocardium to meet the oxygen and nutritional/metabolic requirements of the body.

Causes include:

- » volume overload:
  - L-R shunt lesions
  - mitral/aortic regurgitation
- » pump failure:
  - myocarditis/cardiomyopathy
- » high output failure:
  - septicaemia
  - severe anaemia

DIAGNOSTIC CRITERIA

Clinical

- » Acute cardiac failure may present with shock. See section1.1.7: Shock.
- » History of recent onset of:
  - > poor feeding,
  - > tachypnoea,
  - > sweating,
  - > poor or excessive weight gain,
  - > breathlessness,
  - > cough.
- » Physical findings:
  - > tachycardia,
  - > hypotension,
  - > weak pulses,
  - > cardiomegaly,
  - > cold extremities,
  - > reduced urinary output,
  - > gallop rhythm with/without a cardiac murmur,
  - > pulmonary venous congestion and fluid retention:
    - ▪ tachypnoea,
    - ▪ dyspnoea,
    - ▪ orthopnoea,
    - ▪ recession,
    - ▪ wheezing,
    - ▪ coarse crepitations,
    - ▪ cyanosis;
  - > systemic venous congestion:
    - ▪ hepatomegaly,
    - ▪ periorbital oedema - not seen in infants,
    - ▪ abnormal weight gain;
  - > signs and symptoms of underlying condition/disease.

Investigations as appropriate for the possible underlying cause

- » Chest X-ray: cardiomegaly is almost always present.
- » Electrocardiogram may show evidence of hypertrophy/enlargement of one or more heart chambers and/or dysrhythmias.

4.9.1 HEART FAILURE, ACUTE SEVERE WITH PULMONARY OEDEMA AND SHOCK

I50.9

GENERAL AND SUPPORTIVE MEASURES

» Treat the underlying disorder/condition. Where the primary cause of acute pulmonary oedema is renal failure treat as under renal failure.
» Restrict fluids, beware of IV fluids.
» Place patient in an upright or semi-upright sitting position.
» Intubation and ventilation may be required in an ICU setting.

MEDICINE TREATMENT

- Oxygen 100%, administered via face mask or nasal cannula.

Treat the underlying condition:

- Furosemide, IV, 1–3 mg/kg as a single dose immediately.

For patients not responding to furosemide:

- Morphine, IV, 0.1 mg/kg.

Manage severe hypotensive or refractory failure in an ICU setting.
Inotropic support may help to stabilise patients with severe myocardial dysfunction, hypotension or low cardiac output.
May be lifesaving in severe myocarditis or cardiogenic shock.

- Dobutamine, IV infusion, 2–15 mcg/kg/minute.
  - o Continue until myocardial function and blood pressure improve.

If no response to dobutamine, consider epinephrine (adrenaline) infusion.
Ensure adequate renal function.

Once patient stable and maintaining blood pressure:

▪ ACE inhibitor, e.g.:

- Captopril, oral.
  - o Initial dose: 0.5 mg/kg/24 hours in 3 divided doses (8 hourly) for 24–48 hours.
  - o Increase by 0.5 mg/kg/24 hours every 24–48 hours until maintenance dose of 3–5 mg/kg/24 hours is reached.
  - o Continue for as long as needed to control the cardiac failure.

4.9.2 HEART FAILURE, ACUTE

I50.9

GENERAL AND SUPPORTIVE MEASURES

» Recognise and treat the underlying condition, e.g. infection, hypertension, cardiac tamponade, fluid overload.
» Fluid restriction (75% of daily requirements) – not at the expense of adequate caloric intake.
» Ensure adequate nutrition, tube-feeding may be necessary.
» Monitor blood potassium levels, urea and electrolytes.

MEDICINE TREATMENT

- Oxygen 100%, administered via face mask or nasal cannula.

Combination drug therapy is usually indicated, i.e. start with diuretic, then add digoxin then add ACE inhibitor.

Diuretic therapy

- Furosemide, IV/oral, 1 mg/kg/dose 12 hourly.
  Potassium supplements are necessary if furosemide is used without an aldosterone antagonist, i.e. spironolactone.

AND/OR

In refractory failure:

- Spironolactone, oral, 1–2 mg/kg/dose once daily.

ACE inhibitor

Note:
ACE inhibitors are contraindicated in bilateral renal artery stenosis, coarctation of the aorta and aortic stenosis.

- Captopril, oral.
  - o Initial dose: 0.5 mg/kg/24 hours in 3 divided doses (8 hourly) for 24–48 hours.
  - o Increase by 0.5 mg/kg/24 hours every 24–48 hours until maintenance dose of 3–5 mg/kg/24 hours is reached. If < 1 year do not exceed 4 mg/kg/day.
  - o Continue as long as needed to control the cardiac failure.

OR

- Enalapril, oral, 0.2–1 mg/kg/day as a single dose or 2 divided doses.

Digoxin

Use in consultation with a cardiologist.
Digoxin is contraindicated in bradycardia, heart block, cardiac tamponade or hypertrophic cardiomyopathy. Monitor serum potassium and digoxin blood levels and ECG.
Intravenous digoxin is dangerous and inappropriate.

Because of the potential confusion with digitalising the dose of digoxin. Start with a maintenance dose.

- Digoxin, oral, 0.005 mg/kg/dose 12 hourly.
  - o 0.005 mg = 0.1 mL.
  - o In older children a once daily dose can be given, i.e. 0.01 mg/kg/day.

REFERRAL

» For determination of the underlying cause, where this is not known and initiation of treatment after stabilisation.

» Deterioration despite adequate treatment.

4.10 DYSLIPIDAEMIA

E78.9

DESCRIPTION

Dyslipidaemia is a broad term used to describe disorders of lipid metabolism. Hypercholesterolaemia associated with increased levels of apolipoprotein **Lp(a)** B100-containing lipoproteins (low density lipoprotein (LDL), intermediate density lipoprotein (IDL)) and hy pertriglyceridaemia (chylomicrons, very low density lipoproteins VLDL) have the most serious clinical implications.

Hypercholesterolaemia promotes atherosclerosis and hypertriglyceridaemia is a major component of the metabolic syndrome.

Clinical features depend on the type of hyperlipidaemia.

» **Increased chylomicrons** are associated with eruptive xanthomas and hepatosplenomegaly.

» **Hypertriglyceridaemia** is associated with pancreatitis.

» **Increased levels of VLDL** are associated with familial hypercholesterolaemia (FH).

» **Heterozygous FH phenotype** lacks physical signs.

» **Homozygous FH phenotype** displays physical signs e.g. cutaneous xanthoma.

» **Increased LDL** is associated with glucose intolerance and hyperuricaemia.

Increased levels of HDL cholesterol protects against coronary heart disease.

DIAGNOSTIC CRITERIA

Clinical

» **Hyperlipidaemia** may be secondary to chronic kidney/liver disease, diabetes mellitus, hypothyroidism and medicine treatment e.g. calcineurin inhibitors (transplant patients) and ARV treatment.

- » Screening for hyperlipidaemia is indicated for children at risk of developing premature atherosclerosis, including:
  - > positive family history in parent/grandparent of any of the following conditions presenting <55 years of age:
    - ▪ familial hypercholesterolaemia,
    - ▪ cardiovascular disease,
    - ▪ metabolic syndrome.
  - > overweight or obese children, See section 7.16: Obesity.
- » A high-risk **familial hypercholesteraemia** is perceived to be the concomitant occurrence of 3 or more risk factors:
  - > LDL > 4.9 mmol/L (TChol > 5.2 mmol/L), or
  - > HDL < 1.3 mmol/L, and
  - > positive family history of premature coronary heart disease (myocardial infarct in non-smoking parent < 35 years of age),
  - > male gender,
  - > Lp(a) > 0.3 g/L,
  - > thickened carotid intima (CIMT).

Investigations

- » Exclude causes of secondary hyperlipidaemia
- » In most cases non-fasting total cholesterol is determined in children at risk.

  If level is higher than upper limit, lipid profile is done after 12 hours of fasting.
  - > Upper limit of S-cholesterol and triglycerides: Total cholesterol 5.2 mmol/L.
  - > Triglycerides (after 12 hours of fasting)
    - ▪ influenced by lifestyle – needs attention if > 1.68 mmol/L,
    - ▪ pancreatitis risk if > 10 mmol/L.

GENERAL AND SUPPORTIVE MEASURES

Schedule for integrated cardiovascular health promotion in children.

- » **Obesity**
  - > See section 7.15: Obesity.
- » **Blood pressure**
  - > With family history of hypertension < 55 years of age: routine BP measurement from 3 years of age once a year.
  - > If BP ≥ 95th percentile for sex, age, and height percentile, follow up and investigate if persistently elevated.
- » **Diet**
  - > Hypertriglyceridaemias needs a dietary intervention to restrict triglyceride intake.
  - > Refer to a dietician.
  - > Learning healthy eating habits is an important preventative measure.
  - > Moderate salt intake.

» **Physical activity**
  > Encourage active child-parent play.
  > Limit child's sedentary behaviour such as time watching television and playing video computer games to a maximum of 2 hours per day or 14 hours per week.
  > Children should not be allowed to eat while watching television, i.e. "no grazing".
  > Organised sport 5 times per week for at least 20–30 minute periods.

» **Smoking**
  > Encourage members of the household who smoke to stop.

MEDICINE TREATMENT

Consider medicine treatment only after failure of general and supportive measures to lower the cholesterol over 6-12 months.
Children should be at least 8 years of age for consideration of pharmacological intervention.
If LDL-C remains above 4.1 mmol/L in children with multiple risk factors, or above 4.9 mmol/L in those without a family history of premature heart disease, refer for consideration of statins:

▪ Statins, e.g.:
- Simvastatin, oral, 10 mg at night.

Secondary hypercholesterolaemia due to nephrotic syndrome

For persistent nephrotic range proteinuria (i.e. protein creatinine ratio > 0.2 g/mmol):

▪ ACE inhibitor, e.g.:
- Captopril, oral.
  - o Initial dose: 0.5 mg/kg/24 hours in 3 divided doses (8 hourly) for 24–48 hours.
  - o Increase by 0.5 mg/kg/24 hours every 24–48 hours until maintenance dose of 3–5 mg/kg/24 hours is reached.
    - o Maximum dose: 50 mg 8 hourly.
  - o Continue as long as needed to control the cardiac failure or as long as proteinuria persists.

OR

- Enalapril, oral, 0.2–1 mg/kg/day as single dose or 2 divided doses.

REFERRAL

» Children with familial hypercholesterolaemia or pri mary underlying metabolic disorder.
» LDL > 4.1 mmol/L in children with multiple risk factors.

4.11 HYPERTENSION IN CHILDREN

I10

DESCRIPTION

Hypertension is defined as systolic and/or diastolic blood pressure ≥ the 95th percentile for gender, age and height percentile on at least three consecutive occasions. A sustained blood pressure of >115/80 mmHg is abnormal in children between 6 weeks and 6 years of age. Measure blood pressure with the child in a sitting or supine position with the entire arm in line with the level of the heart.

In children, it is easier to monitor the systolic blood pressure because of better correlation and less technical pitfalls than diastolic blood pressure.

In the majority of children hypertension is due to an identifiable cause. Severe hypertension suggests renal disease.
Hypertensive emergency/crisis exists when CNS signs of hypertension appear such as encephalopathy, convulsions, retinal haemorrhages or blindness. Great care is required to reduce the blood pressure in a controlled manner to avoid potentially serious consequences of impaired auto-regulation of cerebral blood flow.

Hypertensive urgency is defined as a significant elevation of blood pressure without accompanying end organ damage. Patients are generally symptomatic with complaints of headache, blurred vision and nausea, despite the lack of end organ involvement.

A valid assessment of the blood pressure is of extreme importance.
The blood pressure is measured by standard auscultation technique in children > 1 year of age.

In children < 1 year of age, a flush technique is usually used, although Doppler measurement would be preferable.

Measure the BP in at least one limb, preferably the right upper arm. If hypertension is present, measure BP on all four limbs.

One should use the widest cuff that can be applied to the upper arm. The cuff bladder must encircle at least 80% of the upper arm and should cover at least 75% of the distance between the acromion and the olecranon. It is better to use a cuff that is slightly too large than one that is too small. Large cuffs, if covered with linen-like material, can be folded to the appropriate size in smaller infants as long as the bladder encompasses the arm.

DIAGNOSTIC CRITERIA

Clinical

» Symptoms and signs of any of the following systems:
  > central nervous,
  > cardiovascular,
  > respiratory,
  > urogenital.

» The most common associated features are:
  > oedema, haematuria, proteinuria,
  > skin sores (impetigo),
  > convulsions, coma and visual symptoms,
  > acute heart failure and pulmonary oedema,
  > acute respiratory distress, cyanosis and apnoea.

» Some children may be asymptomatic.

» Blood pressure in children correlates with body size and increases with age.

Categories of hypertension

» Normal: below 90th percentile.

» Prehypertension: 90th–95th percentile or BP > 120/80 mmHg.

» Stage 1 hypertension: > 95th–99th percentile plus 5 mmHg.

» Stage 2 hypertension: > 99th percentile plus 5 mmHg.

Age of child	95th Percentile of systolic and diastolic blood pressure	
	First 12 hours	First week
newborn premature	65/45 mmHg	80/50 mmHg
newborn fullterm	80/50 mmHg	100/70 mmHg

Blood pressure levels for Boys by age and height percentile

Age (year)	BP Percentile	Systolic BP (mmHg)							Diastolic BP (mmHg)						
		Percentile of Height							Percentile of Height						
		5th	10th	25th	50th	75th	90th	95th	5th	10th	25th	50th	75th	90th	95th
1	50th	80	81 83 85			87	88	89	34 35 36			37	38 39 39		
	90th	94	95 97 99			100	102	103	49 50 51			52	53 53 54		
	95th	98	99	101	103	104	106	106	54 54 55			56	57 58 58		
	99th	105	106	108	110	112	113	114	61 62 63			64	65 66 66		
2	50th	84	85 87 88			90	92	92	39 40 41			42	43 44 44		
	90th	97	99	100	102	104	105	106	54 55 56			57	58 58 59		
	95th	101	102	104	106	108	109	110	59 59 60			61	62 63 63		
	99th	109	110	111	113	115	117	117	66 67 68			69	70 71 71		
3	50th	86	87 89 91			93	94	95	44 44 45			46	47 48 48		
	90th	100	101	103	105	107	108	109	59 59 60			61	62 63 63		
	95th	104	105	107	109	110	112	113	63 63 64			65	66 67 67		
	99th	111	112	114	116	118	119	120	71 71 72			73	74 75 75		
4	50th	88	89 91 93			95	96	97	47 48 49			50	51 51 52		
	90th	102	103	105	107	109	110	111	62 63 64			65	66 66 67		
	95th	106	107	109	111	112	114	115	66 67 68			69	70 71 71		
	99th	113	114	116	118	120	121	122	74 75 76			77	78 78 79		

Age (year)	BP Percentile	Systolic BP (mmHg) Percentile of Height							Diastolic BP (mmHg) Percentile of Height						
		5th	10th	25th	50th	75th	90th	95th	5th	10th	25th	50th	75th	90th	95th
5	50th	90	91 93 95			96	98	98	50 51 52			53	54 55 55		
	90th	104	105	106	108	110	111	112	65 66 67			68	69 69 70		
	95th	108	109	110	112	114	115	116	69 70 71			72	73 74 74		
	99th	115	116	118	120	121	123	123	77 78 79			80	81 81 82		
6	50th	91	92 94 96			98	99	100	53 53 54			55	56 57 57		
	90th	105	106	108	110	111	113	113	68 68 69			70	71 72 72		
	95th	109	110	112	114	115	117	117	72 72 73			74	75 76 76		
	99th	116	117	119	121	123	124	125	80 80 81			82	83 84 84		
7	50th	92	94 95 97			99	100	101	55 55 56			57	58 59 59		
	90th	106	107	109	111	113	114	115	70 70 71			72	73 74 74		
	95th	110	111	113	115	117	118	119	74 74 75			76	77 78 78		
	99th	117	118	120	122	124	125	126	82 82 83			84	85 86 86		
8	50th	94	95 97 99			100	102	102	56 57 58			59	60 60 61		
	90th	107	109	110	112	114	115	116	71 72 72			73	74 75 76		
	95th	111	112	114	116	118	119	120	75 76 77			78	79 79 80		
	99th	119	120	122	123	125	127	127	83 84 85			86	87 87 88		
9	50th	95	96 98		100	102	103	104	57 58 59			60	61 61 62		
	90th	109	110	112	114	115	117	118	72 73 74			75	76 76 77		
	95th	113	114	116	118	119	121	121	76 77 78			79	80 81 81		
	99th	120	121	123	125	127	128	129	84 85 86			87	88 88 89		

Age (year)	BP Percentile	Systolic BP (mmHg)							Diastolic BP (mmHg)						
		Percentile of Height							Percentile of Height						
		5th	10th	25th	50th	75th	90th	95th	5th	10th	25th	50th	75th	90th	95th
10	50th	97	98	100	102	103	105	106	58	59	60	61	61	62	63
	90th	111	112	114	115	117	119	119	73	73	74	75	76	77	78
	95th	115	116	117	119	121	122	123	77	78	79	80	81	81	82
	99th	122	123	125	127	128	130	130	85	86	86	88	88	89	90
11	50th	99	100	102	104	105	107	107	59	59	60	61	62	63	63
	90th	113	114	115	117	119	120	121	74	74	75	76	77	78	78
	95th	117	118	119	121	123	124	125	78	78	79	80	81	82	82
	99th	124	125	127	129	130	132	132	86	86	87	88	89	90	90
12	50th	101	102	104	106	108	109	110	59	60	61	62	63	63	64
	90th	115	116	118	120	121	123	123	74	75	75	76	77	78	79
	95th	119	120	122	123	125	127	127	78	79	80	81	82	82	83
	99th	126	127	129	131	133	134	135	86	87	88	89	90	90	91
13	50th	104	105	106	108	110	111	112	60	60	61	62	63	64	64
	90th	117	118	120	122	124	125	126	75	75	76	77	78	79	79
	95th	121	122	124	126	128	129	130	79	79	80	81	82	83	83
	99th	128	130	131	133	135	136	137	87	87	88	89	90	91	91
14	50th	106	107	109	111	113	114	115	60	61	62	63	64	65	65
	90th	120	121	123	125	126	128	128	75	76	77	78	79	79	80
	95th	124	125	127	128	130	132	132	80	80	81	82	83	84	84
	99th	131	132	134	136	138	139	140	87	88	89	90	91	92	92

Age (year)	BP Percentile	Systolic BP (mmHg)							Diastolic BP (mmHg)						
		Percentile of Height							Percentile of Height						
		5th	10th	25th	50th	75th	90th	95th	5th	10th	25th	50th	75th	90th	95th
15	50th	109	110	112	113	115	117	117	61 62 63			64	65 66 66		
	90th	122	124	125	127	129	130	131	76 77 78			79	80 80 81		
	95th	126	127	129	131	133	134	135	81 81 82			83	84 85 85		
	99th	134	135	136	138	140	142	142	88 89 90			91	92 93 93		
16	50th	111	112	114	116	118	119	120	63 63 64			65	66 67 67		
	90th	125	126	128	130	131	133	134	78 78 79			80	81 82 82		
	95th	129	130	132	134	135	137	137	82 83 83			84	85 86 87		
	99th	136	137	139	141	143	144	145	90 90 91			92	93 94 94		
17	50th	114	115	116	118	120	121	122	65 66 66			67	68 69 70		
	90th	127	128	130	132	134	135	136	80 80 81			82	83 84 84		
	95th	131	132	134	136	138	139	140	84 85 86			87	87 88 89		
	99th	139	140	141	143	145	146	147	92 93 93			94	95 96 97		

Blood pressure levels for Girls by age and height percentile

Age (year)	BP Percentile	Systolic BP (mmHg)							Diastolic BP (mmHg)						
		Percentile of Height							Percentile of Height						
		5th	10th	25th	50th	75th	90th	95th	5th	10th	25th	50th	75th	90th	95th
1	50th	83	84	85	86 88 89			90	38 39 39			40	41 41 42		
	90th	97	97	98	100	101	102	103	52 53 53			54	55 55 56		
	95th	100	101	102	104	105	106	107	56 57 57			58	59 59 60		
	99th	108	108	109	111	112	113	114	64 64 65			65	66 67 67		
2	50th	85	85	87	88 89 91			91	43 44 44			45	46 46 47		
	90th	98	99	100	101	103	104	105	57 58 58			59	60 61 61		
	95th	102	103	104	105	107	108	109	61 62 62			63	64 65 65		
	99th	109	110	111	112	114	115	116	69 69 70			70	71 72 72		
3	50th	86	87	88	89 91 92			93	47 48 48			49	50 50 51		
	90th	100	100	102	103	104	106	106	61 62 62			63	64 64 65		
	95th	104	104	105	107	108	109	110	65 66 66			67	68 68 69		
	99th	111	111	113	114	115	116	117	73 73 74			74	75 76 76		
4	50th	88	88	90	91 92 94			94	50 50 51			52	52 53 54		
	90th	101	102	103	104	106	107	108	64 64 65			66	67 67 68		
	95th	105	106	107	108	110	111	112	68 68 69			70	71 71 72		
	99th	112	113	114	115	117	118	119	76 76 76			77	78 79 79		

Age (year)	BP Percentile	Systolic BP (mmHg) Percentile of Height							Diastolic BP (mmHg) Percentile of Height						
		5th	10th	25th	50th	75th	90th	95th	5th	10th	25th	50th	75th	90th	95th
5	50th	89	90	91	93 94 95			96	52 53 53			54	55 55 56		
	90th	103	103	105	106	107	109	109	66 67 67			68	69 69 70		
	95th	107	107	108	110	111	112	113	70 71 71			72	73 73 74		
	99th	114	114	116	117	118	120	120	78 78 79			79	80 81 81		
6	50th	91	92	93	94 96 97			98	54 54 55			56	56 57 58		
	90th	104	105	106	108	109	110	111	68 68 69			70	70 71 72		
	95th	108	109	110	111	113	114	115	72 72 73			74	74 75 76		
	99th	115	116	117	119	120	121	122	80 80 80			81	82 83 83		
7	50th	93	93	95	96 97 99			99	55 56 56			57	58 58 59		
	90th	106	107	108	109	111	112	113	69 70 70			71	72 72 73		
	95th	110	111	112	113	115	116	116	73 74 74			75	76 76 77		
	99th	117	118	119	120	122	123	124	81 81 82			82	83 84 84		
8	50th	95	95	96	98 99		100	101	57 57 57			58	59 60 60		
	90th	108	109	110	111	113	114	114	71 71 71			72	73 74 74		
	95th	112	112	114	115	116	118	118	75 75 75			76	77 78 78		
	99th	119	120	121	122	123	125	125	82 82 83			83	84 85 86		
9	50th	96	97	98	100	101	102	103	58 58 58			59	60 61 61		
	90th	110	110	112	113	114	116	116	72 72 72			73	74 75 75		
	95th	114	114	115	117	118	119	120	76 76 76			77	78 79 79		
	99th	121	121	123	124	125	127	127	83 83 84			84	85 86 87		

Age (year)	BP Percentile	Systolic BP (mmHg) Percentile of Height							Diastolic BP (mmHg) Percentile of Height						
		5th	10th	25th	50th	75th	90th	95th	5th	10th	25th	50th	75th	90th	95th
10	50th	98	99	100	102	103	104	105	59	59	59	60	61	62	62
	90th	112	112	114	115	116	118	118	73	73	73	74	75	76	76
	95th	116	116	117	119	120	121	122	77	77	77	78	79	80	80
	99th	123	123	125	126	127	129	129	84	84	85	86	86	87	88
11	50th	100	101	102	103	105	106	107	60	60	60	61	62	63	63
	90th	114	114	116	117	118	119	120	74	74	74	75	76	77	77
	95th	118	118	119	121	122	123	124	78	78	78	79	80	81	81
	99th	125	125	126	128	129	130	131	85	85	86	87	87	88	89
12	50th	102	103	104	105	107	108	109	61	61	61	62	63	64	64
	90th	116	116	117	119	120	121	122	75	75	75	76	77	78	78
	95th	119	120	121	123	124	125	126	79	79	79	80	81	82	82
	99th	127	127	128	130	131	132	133	86	86	87	88	88	89	90
13	50th	104	105	106	107	109	110	110	62	62	62	63	64	65	65
	90th	117	118	119	121	122	123	124	76	76	76	77	78	79	79
	95th	121	122	123	124	126	127	128	80	80	80	81	82	83	83
	99th	128	129	130	132	133	134	135	87	87	88	89	89	90	91
14	50th	106	106	107	109	110	111	112	63	63	63	64	65	66	66
	90th	119	120	121	122	124	125	125	77	77	77	78	79	80	80
	95th	123	123	125	126	127	129	129	81	81	81	82	83	84	84
	99th	130	131	132	133	135	136	136	88	88	89	90	90	91	92

Age (year)	BP Percentile	Systolic BP (mmHg) Percentile of Height							Diastolic BP (mmHg) Percentile of Height						
		5th	10th	25th	50th	75th	90th	95th	5th	10th	25th	50th	75th	90th	95th
15	50th	107	108	109	110	111	113	113	64 64 64			65	66 67 67		
	90th	120	121	122	123	125	126	127	78 78 78			79	80 81 81		
	95th	124	125	126	127	129	130	131	82 82 82			83	84 85 85		
	99th	131	132	133	134	136	137	138	89 89 90			91	91 92 93		
16	50th	108	108	110	111	112	114	114	64 64 65			66	66 67 68		
	90th	121	122	123	124	126	127	128	78 78 79			80	81 81 82		
	95th	125	126	127	128	130	131	132	82 82 83			84	85 85 86		
	99th	132	133	134	135	137	138	139	90 90 90			91	92 93 93		
17	50th	108	109	110	111	113	114	115	64 65 65			66	67 67 68		
	90th	122	122	123	125	126	127	128	78 79 79			80	81 81 82		
	95th	125	126	127	129	130	131	132	82 83 83			84	85 85 86		
	99th	133	133	134	136	137	138	139	90 90 91			91	92 93 93		

GENERAL AND SUPPORTIVE MEASURES

- » There is a strong association with overweight patients and high blood pressure.
- » The majority of these patients have mild hypertension and usually only need lifestyle modification.
- » Acute hypertension:
  - > Bed rest – fowler's position.
  - > Control fluid intake and output (restriction).
  - > Restrict dietary sodium.
  - > Manage end organ effects.
- » Chronic hypertension:
  - > Advise a change in lifestyle.
  - > Institute and monitor a weight reduction programme for obese individuals.
  - > Regular aerobic exercise is recommended in essential hypertension.
- » Dietary advice:
  - > Limit salt and saturated fat intake.
  - > Increase dietary fibre intake.

4.11.1 HYPERTENSION, ACUTE SEVERE

For acute on chronic hypertension blood pressure needs to be lowered cautiously.

Initiate medicines for sustained control as soon as possible to maintain the effect when the emergency measures are discontinued.

Rate of BP reduction depends upon starting BP and age of the child.
In the absence of central nervous system signs, acute hypertension can be rapidly controlled over 24 hours. If in doubt about the duration of hypertension, reduce BP slower over 48 hours.

Aim to reduce the systolic BP with not more than ⅓ of the interval between the patient's systolic blood pressure and the 95th percentile for that age or height in the first 8 hours, then a further gradual decline over the next 24–48 hours.
Do not decrease BP to < 95th percentile in first 24 hours.

GENERAL AND SUPPORTIVE MEASURES

- » Admit patient to paediatric intensive care unit, if possible.
- » Monitor BP every 10 minutes until stable, thereafter every 30 minutes for 24 hours.
- » Set up two peripheral intravenous drips.

MEDICINE TREATMENT

Do not combine medicines of the same class.

- Furosemide, IV, 1–2 mg/kg as a bolus slowly over 5 minutes.
  - If oliguric, maximum dose: 5 mg/kg/dose.
  - Repeat appropriately for fluid overload.

AND

- Labetalol, IV, 0.5–3 mg/kg/hour.
  - 100 mg labetolol in 80 mL sodium chloride 0.45% = 1 mg/mL.
  - Infuse with infusion pump.
  - Give bolus of 0.5 mg/kg and then titrate the dose slowly upwards until the desired blood pressure is achieved.
  - Repeat based on BP response.

If there is an inadequate response:

ADD

- Amlodipine, oral, 0.2 mg/kg/dose.
  - May be repeated after 12 hours.
  - Thereafter every 24 hours.

If phaeochromocytoma suspected use an alpha blocker instead of amlodipine while tapering labetalol, e.g.:

- Prazosin, oral, 12 hourly.
  - 1 month to 18 years: 0.5–1 mg/kg adjusted according to response.

In patients with hypertension due to a neurosecretory tumour (phaeochromocytoma or neuroblastoma), use an α-blocker either as single medicine or in combination with β-adrenergic blocker.

Once blood pressure is controlled, taper to oral treatment.
See section 4.11.2 Hypertension, chronic.

URGENT REFERRAL

» Severe hypertension for specific diagnosis and treatment.

4.11.2 HYPERTENSION, CHRONIC

I10

DESCRIPTION

Primary/Essential hypertension

Occurs most commonly in adolescents.
The patient is often asymptomatic and well.
It is diagnosed by excluding underlying causes of hypertension.
Hypertension is confirmed by sustained high blood pressure measured on 3 follow-up occasions.

Chronic secondary hypertension
All children with incurable forms of persistent secondary hypertension require medicine treatment over and above general and supportive measures.

DIAGNOSTIC CRITERIA

Investigations

» Urine dipstick test.
» Urine MCS.
» Blood urea, calcium, creatinine and electrolytes.
» Chest X-ray, ECG and abdominal sonar.

If all tests are negative, start lifestyle intervention.

GENERAL AND SUPPORTIVE MEASURES

» Introduce physical activity, diet management and weight reduction, if obese.
» Advise teenagers against smoking.
» Follow up to monitor blood pressure and educate patient on hypertension:
  > if blood pressure decreases, continue with non-drug management and follow up;
  > if BP is increasing progressively, reinvestigate to exclude secondary causes or refer;
  > if BP is stable but persistently > 95th percentile and secondary causes have been excluded, start medicine treatment after failed non-drug management for 6 months.
» Consider earlier initiation of medicine treatment if positive family history for cardiovascular disease, essential hypertension or diabetes mellitus.

MEDICINE TREATMENT

Goal of treatment in uncomplicated primary hypertension with no target-organ damage is to achieve BP < 95th percentile. For chronic renal disease, diabetes or hypertension with target-organ damage, target is BP < 90th percentile.
Medicine treatment is initiated for Stage 2 hypertension. Consider therapy in Stage 1 hypertension if there is a family history of cardiovascular disease, hypertension or diabetes.

Aim to achieve control of BP over 48–72 hours in symptomatic patients.

For ambulatory patients start at the lowest dose of the preferred medicine and increase the dose until control is achieved.

Once the highest recommended dose is reached or if the patient experiences adverse effects from the medicine, add a second medicine from a different class.
For patients with persistent hypertension despite the use of first line medicine, add a second/third medicine. There is no specific order in which medicine should be added.

Use specific classes of antihypertensive medicine according to the underlying pathogenesis or illness.

SYSTEMIC HYPERTENSION

Predominantly volume overload HT
(e.g. chronic kidney disease)

↓

Diuretics (e.g. furosemide)

↓

If unsatisfactory control add
β-blocker (e.g. atenolol)

Predominantly renin induced HT
(e.g. nephrotic syndrome, diabetic patient hypertension and persistent proteinuria)

↓

ACE inhibitor
(e.g. enalapril)

↓

If unsatisfactory response add diuretic (e.g. hydrochlorothiazide)

If BP remains uncontrolled, add sequentially as indicated

↓

long-acting calcium channel blocker
(e.g. amlodipine)

↓

β-blocker (e.g. atenolol)

↓

If unsatisfactory response add long-acting calcium channel blocker (e.g. amlodpine)

ACE inhibitor

Monitor renal function when an ACE inhibitor is prescribed because it may cause a decline in GFR resulting in deterioration of renal function and hyperkalaemia.
Adverse effects include hyperkalaemia and decreased GFR.
Check renal function and serum potassium periodically.
Contraindicated in bilateral renal artery stenosis.

- Enalapril, oral, 0.04 mg/kg/dose 12 hourly.
  - Maximum 0.3 mg/kg/dose up to 40 mg/day.

OR

For young children less than 10 kg body weight:

- Captopril, oral.
  - Initial dose: 0.1 mg/kg/dose 8 hourly.
  - Maximum 2 mg/kg/dose.

β-blocker

- Atenolol, oral, 0.5–1 mg/kg/dose once daily.
  - Maximum dose: 2 mg/kg/day.
  - Contraindicated in severe heart failure and asthma.

OR

If child less than 10 kg body weight:

- Propranolol, oral, 0.25–1 mg/kg/dose 8–12 hourly.
  - o Maximum dose: 1.5 mg/kg/dose.

Calcium channel blocker

- Amlodipine, oral, 0.1–0.2 mg/kg/dose once daily.

Diuretic

- Hydrochlorothiazide, oral, 0.5–1 mg/kg/dose once daily.
  - o May cause hypokalaemia.

OR

- Furosemide, oral, 0.5–1.5 mg/kg/dose 12–24 hourly.
  - o Maximum dose: 6 mg/kg/day.
  - o May cause hypokalaemia.

α-blocker, e.g:

Also indicated in patients with phaeochromocytoma-associated hypertension.

- Prazosin, oral, 12 hourly.
  - o 1 month to 18 years: 0.5–1 mg/kg adjusted according to response.

REFERRAL

» All children with chronic hypertension for specific diagnosis, planning of treatment and long-term follow-up.

» Persistent cough on treatment with ACE inhibitor.

4.12 CHILDREN WITH PROSTHETIC HEART VALVES

Z95.2

DESCRIPTION

Valve replacement may be required for severe valvular disease when valve repair is not feasible or advisable.

The valves may be mechanical valves or bioprosthetic valves or preserved human tissue valves.

In children bioprosthetic valves tend to degenerate, calcify and have structural deterioration more frequently and more rapidly compared with adults.

Mechanical valves are more commonly used in children.

Complications include:

» Valve failure. May be abrupt (teari ng of components) or gradua l (with calcification and stiffening of leaflets).

» Prosthetic valve thrombosis.

» Prosthetic valve endocarditis.

» Haemolytic anaemia.

MEDICINE TREATMENT

After mechanical valve replacement warfarin therapy is indicated to achieve an INR of 2.5 (range 2.0–3.0):

- Warfarin, oral, 0.1 mg/kg/daily.
  - o Adjust the dose depending on INR.
  - o Beware of haemorrhage.

Warfarin dose adjustment based on INR

INR < 1.5	Verify adherence.<br>If non-adherent resume at previous dose.<br>If dosage adjustments needed increase dose by 20% and review in 3 – 7 days.
INR 1.5–1.9	Verify adherence first.<br>Increase maintenance dose by 10%.
INR 2.0–3.0	No change needed.<br>In mitral valve, INR is closer to 3.0.
INR 3.1–4.0	Consider withholding one dose, and decrease by 10%.
INR 4.1–4.5	Decrease dose by 20%.
INR > 4.5	Withhold dose, evaluate INR daily until <4.5, then restart at 20% below previous dose.

The half-life of warfarin is 40 hours, dose adjustments may thus be calculated over a 48 hour period. The 10% and 20% dose adjustments may not be precisely achieved, approximate doses are acceptable.
If warfarin of 1 mg per tablet is not available and dosage adjustments are problematic discuss with paediatric cardiologist.
Some medicines and foods interfere with the warfarin effect.

Medicines that enhance anticoagulant effect include:

» allopurinol,
» aspirin,
» NSAIDS,
» paracetamol (regular use),
» valproate,
» phenytoin,
» imidazoles,
» metronidazole,
» macrolides, and
» quinolones.

Medicines that diminish anticoagulant effect include:
- » carbamazepine,
- » phenobarbital,
- » phenytoin, (both diminished and enhanced effects have been reported)
- » nevirapine,
- » rifampicin.

Foods that contain high amounts of vitamin K and can decrease the effectiveness of warfarin, e.g.:
- » spinach,
- » parsley, and
- » brussel sprouts.

Certain drinks can increase the effect of warfarin e.g. cranberry juice and alcohol.

CHAPTER 5

DERMATOLOGY

Skin lesions are best characterised by their morphologic appearance which allows consideration of a suitable differential diagnosis.

5.1 BULLAE

5.1.1 EPIDERMOLYSIS BULLOSA

Q81.9

DESCRIPTION

Congenital, hereditary blistering skin lesions with onset in the newborn. Lesions do not have erythematous base. Loss of nails may occur.

GENERAL AND SUPPORTIVE MEASURES

- » May require care in high or intensive care unit.
- » Do not rupture bullae.
- » Prevent infection with appropriate wound care.
- » Attend to fluid and nutrition balance.

REFERRAL

- » All cases.

5.1.2 STAPHYLOCOCCAL SCALDED SKIN SYNDROME

L00

DESCRIPTION

Blistering skin condition that presents like scalded skin.

GENERAL AND SUPPORTIVE MEASURES

- » Appropriate wound care.

MEDICINE TREATMENT

- » Cloxacillin, IV, 50 mg/kg/dose 6 hourly for 5 days.
  - o Neonates
    - Week 1–2, administer 12 hourly.
    - Week 2–4, administer 8 hourly.

OR

- » Flucloxacillin, oral, 12.5–25 mg/kg/dose 6 hourly for 7 days.

REFERRAL
» Recalcitrant cases.

5.1.3 CHRONIC BULLOUS DISEASE OF CHILDHOOD
L12.2

DESCRIPTION
Tense blisters that lead to ulceration involving the groin, face and trunk.

DIAGNOSTIC CRITERIA
» Skin biopsy with immunofluorescence.

GENERAL AND SUPPORTIVE MEASURES
» Appropriate wound care.

REFERRAL
» All cases.

5.2 ERYTHEMA AND DESQUAMATION

It is a continuum ranging from Erythema Multiforme (EM) to Stevens-Johnson Syndrome and then to the potentially lethal Toxic Epidermal Necrolysis (TEN).

5.2.1 ERYTHEMA MULTIFORME/STEVENS-JOHNSON SYNDROME
L51.9/L51.1

DESCRIPTION
Acute, vesico-bullous disorder with numerous manifestations on the skin, mucous membranes and, occasionally, internal organs caused mainly by:
» medicines, e.g. sulphonamides, phenytoin, phenobarbitone,
» exposure to toxic substances, and
» infections, e.g. herpes simplex and mycoplasma.

Complications include:
» conjunctivitis,
» uveitis,
» corneal scarring,
» fluid loss,
» infections,
» anaemia, and
» oesophageal strictures.

DIAGNOSTIC CRITERIA

Iris or target lesions consisting of a dark centre, an inner pale ring and an erythematous outer border. In erythema multiforme these lesions are pathognomonic.

Erythematous macules evolve into papules, vesicles, bullae, urticarial plaques or patches of confluent erythema. The centre of the lesion may be vesicular, purpuric or necrotic.

Erythema multiforme minor

Prodromal symptoms are generally absent. Symmetric crops of skin lesions of diverse morphology, primarily on the extensor surfaces of the arms and legs and often including soles and palms with relative sparing of the mucous membranes and the trunk.

Erythema multiforme major (Stevens-Johnson syndrome)

A serious, systemic condition involving the skin and at least two mucous membranes.

Eruption may be preceded by non-specific prodromal symptoms like:

» malaise,
» fever,
» chills, or
» upper respiratory infection.

Cutaneous lesions tend to rupture, leaving the skin denuded, with fluid loss, anaemia and high risk of infection.

Involvement of oral mucosa is common.

GENERAL AND SUPPORTIVE MEASURES

» May require care in high or intensive care unit.
» Examine daily for systemic involvement, infection and ocular lesions. If infection is suspected, send blood and skin lesion specimens for culture and sensitivity before initiating antibiotic therapy.
» Do not puncture bullae or vesicles.
» Frequent mouth washes for oral lesions.
» Cool compresses and wet dressings.
» Eye care where eye cannot close. See section 16.1: Eye infection, complicated (Severe eye infection).
» Maintain fluid balance. Beware of shock.
» Nasogastric feeds if unable to eat, IV alimentation if enteral feeds are not possible.
» Stop all medicines.

MEDICINE TREATMENT

For pain:

These patients require effective pain control.

Change of dressing protocol: See Section 20.1: Management of pain.

Antibiotic therapy

For secondary infections

Reconsider choice of antibiotic when the results of cultures become available or the child does not improve.

Use IV antibiotics if the oral route cannot be used.

- Cloxacillin, IV, 50 mg/kg/dose 6 hourly.

OR

- Cephalexin, oral, 6.25–12.5 mg/kg/dose 6 hourly.

For oral lesions:

- Chlorhexidine 0.2%, 15 mL as a mouthwash.
  - o Use as needed.
  - o Do not swallow.

Note:

The use of systemic corticosteroids is not recommended.

REFERRAL

» Erythema multiforme not responding to adequate therapy.

» Erythema multiforme with ocular involvement.

5.3 MACULES AND PAPULES

5.3.1 DRUG REACTIONS

L27.0

Commonly associated with:

» sulphur containing agents,

» penicillin,

» anti-epileptics (e.g. carbamazepine, lamotrigine),

» NSAIDs,

» anti-tuberculosis drugs, and

» non-nucleoside reverse transcriptase inhibitors.

A variety of rashes o ccur, ranging from (worst) erythema multiforme with mucosal involvement, target lesions, blistering and fever, through itchy or painful urticarial eruptions, measles-like maculopapular rashes, to erythema and flat, sy mmetrical macular lesions (fixed drug reactio ns). These are commonly flat or slightly raised lesions of < 0.5 cm in size.

Lesions recur upon re-exposure to the causative agent.

GENERAL AND SUPPORTIVE MEASURES

» Stop causative agents.

MEDICINE TREATMENT

Antihistamines:

For children 3 years and older:

- Cetirizine, oral, as a single dose at night.
  - o Children 3–12 years: 5 mg.
  - o Children > 12 years: 10 mg.

Where the oral route can be used:

- Chlorphenamine, oral, 0.1mg/kg/dose as a single dose at night.

Where the oral route cannot be used:

- Promethazine, IV, 0.1 mg/kg/dose 8–12 hourly.

REFERRAL

» Systemic involvement with organ dysfunction.

5.3.2 ACNE

L70

DESCRIPTION

An inflammatory condition of hair follicles leading to comedone formation that can cause scarring and post inflammation hyper-pigmentation.

DIAGNOSTIC CRITERIA

» Black or white heads (comedones).

GENERAL AND SUPPORTIVE MEASURES

» Avoid greasy and oily topical products.
» Discourage excessive facial washing.

MEDICINE TREATMENT

- Benzoyl peroxide 5%, topical, applied to affected areas at night.

AND

- Doxycycline, oral, 100 mg once daily for a maximum of three months.

If ineffective

Stop benzoyl peroxide

To limit skin irritation, introduce topical retinoids, e.g. tretinoin gel/cream, gradually at night.

- Tretinoin cream 0.05%, topical, applied sparingly once daily at bedtime until substantial improvement.
  - o Avoid contact with eyes and mucous membranes.
  - o Limit exposure to sunlight, especially with concomitant use of doxycycline.

> Tretinoin is teratogenic.
> Do not use where pregnancy is a possibility.
> If used, ensure adequate contraception.

To avoid sun irritation:

- Sunscreen, topical, applied daily.

REFERRAL

- » Recalcitrant and/or fulminant acne.
- » Psychologically disturbed or depressed patient.
- » Young females with premenstrual flare or with clinical signs of hyperandrogenism for consideration of oral contraceptives.

5.3.3 CELLULITIS AND ERYSIPELAS

L03.9/A46

DESCRIPTION

Infection of the skin and subcutaneous tissue usually caused by streptococci, staphylococci or *H. influenzae*. In cellulitis, the border is indistinct.

Erysipelas

The affected area is:

- » well demarcated with firm borders,
- » very tender and warm,
- » bright red and swollen.

Erysipelas must be distinguished from necrotising fasciitis where there is infection and inflammation usually by a gas-forming organism that spreads rapidly along the fascial tissue.
Complications may lead to septicaemia.

DIAGNOSTIC CRITERIA

- » Acutely ill child with fever and malaise.
- » Involved area is swollen, indurated, erythematous and painful/tender with regional lymphadenopathy.

GENERAL AND SUPPORTIVE MEASURES

- » Ensure adequate nutrition and hydration.
- » Elevate the affected limb to reduce swelling.
- » Exclude eczema, immunocompromised state, diabetes and underlying osteomyelitis.

MEDICINE TREATMENT

Choice of intravenous or oral antibiotics depends on the severity of the condition.

Severe disease

- Cloxacillin, IV, 50 mg/kg/dose 6 hourly for 5 days.

For peri-orbital cellulitis:

- Ceftriaxone, IV, 50 mg/kg once daily.

OR

If one month old or younger:

- Cefotaxime, IV, 50 mg/kg/dose, 6–8 hourly.

Non-severe disease

- Cephalexin, oral, 12.5–25 mg/kg/dose 6 hourly for 7 days.
  - o Child < 2 years: 125 mg.
  - o Child 2–10 years: 250 mg.
  - o > 10 years: 500 mg.

<u>Penicillin allergy</u>

- Erythromycin, oral, 6.25–12.5 mg/kg/dose, 6 hourly for 5 days.

For pain:

- Paracetamol, oral, 15 mg/kg/dose, 6 hourly as required.

If needed **ADD**:

- Ibuprofen, oral, 5–10 mg/kg/dose, 6 hourly for 72 hours.
  - o Child < 30 kg, maximum dose: 500 mg/day.

REFERRAL

» **Urgent**: necrotising fasciitis.
» Poor response to therapy.
» Recurrent cellulitis.

5.3.4 ECZEMA

L20.9

DESCRIPTION

An inflammatory itchy skin condition characterised by:

» Vesicles, weeping and crusting in the acute stage.
» Scaling and lichenification during the chronic stage.

DIAGNOSTIC CRITERIA

» Family history of allergies.
» Reaction after exposure to allergens.
» Typical distribution: face, flexures of knees and elbows, and creases of neck.

GENERAL AND SUPPORTIVE MEASURES

» Avoidance measures: use neutral soaps and rinse clothes properly after wash.
» Keep fingernails short to prevent scratching.
» Wrap with dressings soaked in sodium chloride 0.9%.
» Avoid sunlight and use sunscreen.

MEDICINE TREATMENT

Antihistamine

- Cetirizine, oral, 12 hourly
  - o Children 6 months to 2 years: 0.25 mg/kg/dose
  - o Children 2–6 years: 2.5 mg.
  - o Children 6 -12 years: 5 mg.
  - o Children > 12 years: 10 mg.

To relieve skin dryness:

- Aqueous cream, topical.

For baths and prior to applying corticosteroids (after drip drying):

- Emulsifying ointment, topical.

For the face and skin folds:

- Hydrocortisone 1%, topical, 12 hourly.

For the body:

- Betamethasone 0.1%, topical, undiluted applied once daily for 7 days.
  - o Moisturise with emulsifying ointment during therapy and in subsequent weeks.

Secondary bacterial infection:

- Cephalexin, oral, 6.25–12.5 mg/kg/dose, 6 hourly for 14 days.

Note:
Short term use of topical steroids is recommended.
Oral corticosteroids do not have a role in the management of this condition.

REFERRAL

» Recalcitrant cases.
» Concomitant food allergy (allergy clinic).

5.3.5 CANDIDIASIS

B37.2

DESCRIPTION

Skin infection involving axillae, neck and perineum. Commonly occurs in immunocompromised individuals. Involvement of mouth and perineal regions suggests systemic disease.

DIAGNOSTIC CRITERIA

Clinical

» Red raw looking patches with satellite white pustular lesions on an erythematous base.

» Mucosal involvement.

Investigations

» Wet preparation with potassium hydroxide or biopsy and culture.

GENERAL AND SUPPORTIVE MEASURES

» Control underlying immunosuppressive state e.g. diabetes, HIV.

» Personal hygiene of mothers prior to breast feeding.

MEDICINE TREATMENT

- Imidazole cream 1%, e.g. clotrimazole, topical, applied 8 hourly for 14 days.

If no response:

- Fluconazole, oral, 3–6 mg/kg/day for 14 days.

REFERRAL

» Recalcitrant infection.

5.3.6 PSORIASIS

L40.9

DESCRIPTION

An inflammatory condition of the skin and joints.

DIAGNOSTIC CRITERIA

» Scaly, red itchy papules and plaques over scalp, perineum, and skin folds and extensor surfaces.

» Nails may be opaque, deformed and crumbling.

» Occasional pustules are seen.

GENERAL AND SUPPORTIVE MEASURES

» Avoid precipitants e.g. drugs.

MEDICINE TREATMENT

Local plaques

To remove scales in children 12 years and older:

- Salicylic acid 2% and coal tar in white soft paraffin, applied 8 hourly.

For scalp lesions:

- Mild coal tar shampoo.

Severe pustular psoriasis
- Hydrocortisone 1%, topical, applied 1–2 times daily.

OR
- Prednisone, oral, 1–2 mg/kg as a single daily dose for 7 days.

REFERRAL
» Severe psoriasis and recalcitrant cases.
» Intolerance to salicylic acid.
» No response to treatment.

5.3.7 URTICARIA
L50.9

DESCRIPTION
An itchy inflammatory skin and mucosal condition recognised by wheal and flare reaction that may be acute or chronic, often due to irritants, insect bites or allergens. Secondary infective features include excoriation, vesicles and pigmentary changes. Chronic papular eruptive urticaria is often seen in HIV infected individuals.

DIAGNOSTIC CRITERIA
» History of allergen exposure.
» Wheal and flare reaction (“hives”).
» Positive skin test if due to allergy.

GENERAL AND SUPPORTIVE MEASURES
» Limit exposure to precipitants, e.g. drugs, allergens and toxins.
» Limit exposure to insects by using topical insect repellent which contains more than 10% diethyltoluamide (DET).
» Wrap with dressings soaked in sodium chloride 0.9%.

MEDICINE TREATMENT
- Chlorphenamine, oral, 0.1 mg/kg/dose as a single dose at night.

- Hydrocortisone 1%, topical, applied twice daily as required.
  - Useful when applied immediately after insect bite.

Severe chronic urticaria
- Cetirizine, oral, as a single dose at night.
  - Child 3–12 years: 5 mg.
  - Child > 12 years: 10 mg.

REFERRAL
» Recalcitrant and chronic cases.

5.4. PURPURA
D69.9

5.4.1 MENINGOCOCCAEMIA
A39.2

DESCRIPTION
Palpable bleeding into skin caused by *N. meningitides* and is associated with rapid spread.

This is a medical emergency and can be fatal.
See section 8.25: Sepsis.

5.4.2 HENOCH-SCHÖNLEIN PURPURA
D69.0

See section 12.1: Henoch Schönlein Purpura (HSP)

5.4.3 IDIOPATHIC THROMBOCYTOPENIC PURPURA (ITP)
D69.3

See section 3.10: Idioptahic thrombocytopaenic purpura (ITP)

5.5. VESICLES AND PUSTULES

5.5.1 INFECTIONS
R23.8/L08.9

See section 8.26: Varicella (chickenpox) and section 8.27: Zoster.

5.5.2 SKIN AND MUCOSAL DISORDERS IN HIV

Skin and mucosal disorders are more severe in immune suppressed (HIV-infected) patients and may be worsened by IRIS. It may present initially with a skin or a mucosal lesion or skin and mucosal lesions may develop during the course of the HIV illness.

Lesions respond to antiretroviral therapy together with treatment for a specific skin and/or mucosal disorder. Skin eruptions or rashes are relatively common in HIV patients and relate to antiretroviral and other medicines.

Conditions that are frequently more common in patients with HIV or are more specific in their unusual presentation include:
» HIV papular pruritic eruption.
» Kaposi's sarcoma.

5.5.2.1 HIV PAPULAR PRURITIC ERUPTION

DESCRIPTION

Chronic itchy condition with a relapsing course due to an initial allergy to insect bites in a susceptible patient who developed an immune reaction to insect products. In HIV-infected patients the insect bite reactions are severe and recalcitrant with post inflammatory pigmentation and scarring.

DIAGNOSTIC CRITERIA

» Initial lesion is a pruritic urticarial spot with a central red punctum.
» Lesions progress to pruritic papules with or without blisters which, due to scratching, may present with inflammatory changes, erosions, crusts or scabs with secondary infection.
» Post inflammatory pigmentation and scarring are common.

GENERAL AND SUPPORTIVE MEASURES

» Prevent insect bites e.g. insect repellents, eradicate fleas and other insects.

MEDICINE TREATMENT

- Calamine lotion, topical, applied as needed.
- Chlorphenamine, oral, 0.1 mg/kg/dose 6 hourly.
- Betamethasone 0.1%, topical, applied 12 hourly for 3 days.

Then, until pruritus subsides:

- Hydrocortisone 1%, topical applied 12 hourly.

Treat secondary infection with an appropriate antibiotic, if indicated.

Treatment of HIV. See section 9.1: Human immunodeficiency virus infections.

REFERRAL

» No response to treatment.

5.5.2.2 KAPOSI'S SARCOMA

C46.9

DESCRIPTION

Kaposi's sarcoma is a vascular tumour that can present anywhere on the skin and also involve lymph nodes and internal organs, primarily lungs and gastrointestinal tract.

It is associated with human herpes virus 8 and occurs most commonly in immunocompromised HIV-infected patients.
It can be asymptomatic and indolent or aggressive, characterised by explosive growth and death.

DIAGNOSTIC CRITERIA

» Presents with skin lesions on the limbs particularly the lower leg and foot but skin lesions may occur anywhere on the body.
» Lesions (skin and mucosal) may be bruise-like patches, purple or purple-red plaques, subcutaneous papules or nodules.
» Lymphoedema, ulceration and secondary bacterial infection may occur.

GENERAL AND SUPPORTIVE MEASURES

» Counselling to assist patient in dealing with the condition.

MEDICINE TREATMENT

Manage in consultation with an oncologist.

Treat secondary infection with an appropriate antibiotic, if indicated.

Treatment of HIV. See section 9.1: Human immunodeficiency virus infections.

Supportive treatment e.g. pain, once condition is considered untreatable. See section 20.1: Management of pain.

REFERRAL

» All not responding to ART.
» Extensive progressive disease.

5.5.2.3 WARTS

MEDICINE TREATMENT

- Podophyllin resin 20% or salicylic acid 25% ointment, applied under plaster nightly.
  - o Protect surrounding skin with petroleum jelly.
  - o Repeat until the wart falls off.

REFERRAL

» Extensive warts involving the face.
» Genital warts: Refer to STI clinic.

5.5.3 IMPETIGO

See Standard Treatment Guidelines and Essential Medicines List for Primary Health Care.

CHAPTER 6

RENAL CONDITIONS

6.1 NEPHROLOGICAL/UROLOGICAL DISORDERS

6.1.1 POST STREPTOCOCCAL GLOMERULONEPHRITIS

N05.9

DESCRIPTION

Acute post-streptococcal glomerulonephritis is a disorder of th e kidneys caused by an immunological response of the kidney to nephritogenic strains of streptococci. It develops one to three weeks after a streptococcal throat or skin infection. Immune complexes are deposited in the glomerular basement membrane and/or mesangium of the glomeruli.

DIAGNOSTIC CRITERIA

Clinical

» Occurs predominantly in children 3–12 years old.
» Presents 1–3 weeks after streptococcal pharyngitis or skin infection (impetigo).
» Characteristic features include:
  > facial or generalised oedema,
  > painless macroscopic haematuria (smoky or tea coloured urine),
  > oliguria, and
  > hypertension.

Special investigations to confirm APSGN

**Urine analysis**	
Macroscopic appearance	smoky, brown, bloody
Urine test strips	1+ to 3+ haematuria; ± trace to 2+ proteinuria
Microscopic examination	dysmorphic red blood cells;<br>red blood cell and granular casts
**Blood investigations**	
**Streptococcus serology**<br>ASO or Anti-DNAseB titre	positive in the absence of prior antibiotic treatment<br>(ASO often negative in preceding skin infections)
**Complement study**<br>C_3<br>C_4	 decreased<br>normal
**S-biochemistry**	
Serum electrolytes	dilutional hyponatraemia, hyperchloraemic<br>hyperkalaemic metabolic acidosis is common
S-Urea & creatinine	mildly elevated in the acute phase
Full blood count	dilutional anaemia; thrombocyte count is normal

GENERAL AND SUPPORTIVE MEASURES

» Bed rest is necessary in children with severe hypertension or pulmonary oedema.
» Monitor fluid balance:
  > Weigh daily and record fluid intake and output strictly.
  > Allowed fluid intake is dependent on urine output.
  > In small children fluid balance is best monitored with regular weighing.
  > Never use a potassium-containing solution in an anuric patient.
  > Do not use parenteral fluids if oral intake is possible.
» Fluid management according to fluid status:
  > **Pulmonary oedema plus oliguria/anuria**: Do not give fluid.
  > **Hydrated anuric patient without extra-renal fluid losses**: Oral fluid to replace insensible water losses only.
    Insensible water loss is calculated as:
    ▪ 25 mL/kg/day(400 mL/m^2/day).
    In exceptional circumstances, if parenteral fluid is required, use an electrolyte free solution i.e. dextrose 5% or 10%, IV.
  > **Normally hydrated plus oliguria**: Oral fluid intake to replace insensible water loss and urine output of previous 24 hours.
  > **Normally hydrated plus normal urine output**: Give normal fluid intake.
» Dietary measures:
  > Restrict sodium intake in all patients.
  > Restrict potassium intake until result of serum electrolytes is available.
  > Restrict protein intake to 0.5 g/kg/day.
  > Bread and jam is a relatively safe option if no dietician is available.

MEDICINE TREATMENT

Eradication of streptococci

- Phenoxymethylpenicillin, oral, 50 mg/kg/24 hours in 4 divided doses (6 hourly) for 10 days.

OR

If unable to take oral medication:

- Benzathine benzylpenicillin (depot formulation), IM, 30 000 units/kg/dose, 2 doses given 5 days apart.
  - o Maximum dose: 1.2 million units.

Penicillin allergy

- Erythromycin, oral, 10 mg/kg/dose, 6 hourly for 10 days

Hypertension

Hypertension usually develops acutely due to fluid overload.

Hypertensive crisis: Patient with signs of hypertensive encephalopathy, i.e.:
» convulsions,
» retinal haemorrhages, visual loss and
» left heart failure.

Hypertensive urgency: Symptomatic patients with significant elevation of blood pressure with complaints of headache, blurred vision and nausea but lacks the above clinical manifestations.

Initiate treatment for acute hypertension: See section 4.11.1: Hypertension, acute severe.

For acute hypertensive emergency due to post streptococcal glomerulonephritis:
- Furosemide, IV, 1–2 mg/kg/dose.

If oliguric:
- Furosemide, IV, 5 mg/kg/dose.
  - o Administer IV bolus slowly over 5 minutes due to risk of ototoxicity.

AND
- Labetalol, IV, 0.2–1.0 mg/kg/dose as a bolus.
  - o Maximum bolus dose: 40 mg.
  - o Continue infusion: 0.25–3.0 mg/kg/hour.
  - o Taper infusion rate up or down according to response.

If no hypertensive crisis but persistent significant hypertension:
- Propranolol, oral, 1-2 mg/kg/dose, 6 hourly.
  - o Maximum dose: 8 mg/kg/24 hours.

If blood pressure is not adequately controlled:
ADD
- Amlodipine, oral, 0.2 mg/kg/dose.
  - o May be repeated 6 hours later, thereafter once every 24 hours.
  - o Maximum dose: 5 mg.
  - o Crush 5 mg tablet and disperse in 5 mL water: amlodipine 1 mg/mL.

Once blood pressure has normalised, taper and stop antihypertensive treatment. Monitor blood pressure over the next 48 hours to exclude rebound hypertension.

Volume overloaded and pulmonary oedema
See fluid management in general and supportive measures.

- Furosemide, slow IV, 2 mg/kg/dose.
  - o Maximum dose: 5 mg/kg/dose.
  - o Maximum cumulative daily dose: 8 mg/kg/24 hours.

For pulmonary oedema:
See fluid management in general and supportive measures.

- Morphine, IV, 0.1 mg/kg/dose.
  - o Repeat after 4 hours if required.

- Oxygen, 100%, 2–3 L/minute by nasal cannula.

REFERRAL
Urgent (as soon as possible)
» Anuric patient with acute volume overload and unresponsive to furosemide.
» Uncontrolled hypertension.
» Oliguric and progressive renal failure.
» Cardiac failure or pulmonary oedema not responding to treatment.

For specialist advice
» Macroscopic haematuria persisting for more than 4 weeks or persistent proteinuria.
» Family history of renal disease.
» Streptococcal aetiology unproven (ASOT and anti-DNAseB negative, normal C_3 levels, decreased C_4 levels).
» Decreased complement levels which persist for more than 6 weeks.
» Persistent renal failure after initial recovery.
» Persistent hypertension.

6.1.2 URINARY TRACT INFECTION (UTI)

N39.0

DESCRIPTION

Bacterial infection of the urinary tract.

Uncomplicated urinary tract infection (UTI) is an infection which is limited to the lower urinary tract and there are no associated urological anomalies. It is seen most commonly in girls over two years of age.

It presents with localising symptoms of dysuria, frequency, urgency, cloudy urine and lower abdominal discomfort. Urine dipstick test show positive leukocyte esterase, nitrites and haematuria.

See the Standard Treatment Guidelines and Essential Medicines List for Primary Health Care Level.

Complicated urinary tract infection (UTI) is an infection of the urinary tract involving the renal parenchyma (acute pyelonephritis) or which is associated with underlying congenital anomalies of the kidneys and urinary tract.

It is accompanied by fever and other systemic features as described below. It may result in significant short-term morbidity, including septicaemic shock and acute renal failure, especially in infants.

Permanent renal damage may occur in children who have recurring episodes of pyelonephritis.

DIAGNOSTIC CRITERIA

Clinical

» Signs and symptoms are related to the age of the child and are often non-specific.
» Uncomplicated urinary tract infections may cause very few signs and symptoms. Complicated infections may present with a wide range of signs and symptoms.
» Neonates may present with:
  > fever,
  > hypothermia,
  > poor feeding,
  > sepsis,
  > vomiting,
  > prolonged jaundice,
  > failure to thrive,
  > renal failure.
» Infants and children may present with:
  > failure to thrive,
  > persisting fever,
  > abdominal pain,
  > frequency,
  > dysuria,
  > enuresis or urgency.

Examine the urine in any child with fever of unknown origin.

Special investigations

» Urine bag specimens are used for screening purposes only.
  > When urine dipstick test of bag specimen reveals presence of leukocytes or nitrites, collect urine aseptically for urine MCS.
  > Urine specimen is collected aseptically:
    ▪ by suprapubic aspiration or transurethral bladder catheterisation in acutely ill children < 2 years of age or in smaller children who are unable to co-operate;

    or

    ▪ by mid-stream clean catch method in older children.
» Criteria for the diagnosis of UTI:
  > any culture from a suprapubic urine sample,
  > a culture of > 10^4 col/mL urine of a single organism from a catheter specimen,
  > a pure culture of > 10^5 col/mL in a mid-stream clean catch sample or consistent culture of a pure growth even with counts as low as 10^4 col/mL.

- » Ultrasound:
  - > Do a ren al ultrasound in **all** children with first UTI as soon as possible, unless a normal ultrasound was previously seen.
- » MCUG:
  - > in children who have abnormalities of the kidneys, ureter or bladder demonstrated by ultrasound.

GENERAL AND SUPPORTIVE MEASURES

- » Ensure adequate nutrition and hydration. Maintain hydration with oral and/or IV fluids if necessary.
- » For recurring infections:
  - > avoid irritant soaps and bubble baths,
  - > treat constipation, if present,
  - > treat pinworm,
  - > perineal hygiene,
  - > regular complete emptying of the bladder and/or double voiding, i.e. making an additional attempt at voiding after the initial flow of urine has ceased.

MEDICINE TREATMENT

Antibiotic therapy

Total duration of antibiotic therapy: <u>minimum of 7 days</u>.
Increase duration to 10–14 days in infants who had septicaemia.
The empiric choice of antibiotics depends on the expected sensitivity of the suspected organism.
Review antibiotic choice once culture and sensitivity results become available.

<u>Oral treatment:</u>
Children > 3 months old, who are unwell but not acutely ill and who are not vomiting:
Children with uncomplicated UTI:

- Amoxicillin/clavulanic acid, oral, 30 mg/kg/dose of amoxicillin component 8 hourly.

<u>Parenteral treatment:</u>
All neonates and acutely ill infants should preferably be treated parenterally for the first few days until temperature has normalised and they are able to tolerate feeds.

- Amoxicillin/clavulanic acid, IV, 25 mg/kg/dose 8 hourly.

OR

- Ceftriaxone, IV, 80 mg/kg daily.

If there is no improvement after 24 hours of IV amoxicillin/clavulanic acid treatment and culture result is pending, a resistant organism may be the cause.

ADD

- Gentamicin IV, 5 mg/kg/dose once daily.
  - o Do a trough blood level before the third dose.
  - o Consult a specialist if trough levels are high.

If there is evidence of good clinical response to amoxicillin/clavulanic acid alone, change to:

- Amoxicillin/clavulanic acid, oral, 30 mg/kg/dose of amoxicillin component 8 hourly.

Investigate all children with a structural or functional abnormality of the urinary tract and recurrent **symptomatic urinary tract infections** for infection.

If patient has temperature > 38.5˚C or symptoms of urinary tract infection, do urine dipstick test. If positive leucocytes and/or nitrites in fresh urine, collect urine aseptically for MCS.
Treat urinary tract infection empirically as above.

<u>Long-term prophylactic antibiotic therapy:</u>

Asymptomatic bacteriuria does not require treatment.
Use of long-term prophylactic antibiotic therapy for UTI is not recommended.

Prophylaxis may be indicated in specific risk groups, i.e. for children < 2 years of age and who have a structural or functional abnormality of the urinary tract associated with increased risk of recurrent infections, i.e. grade III or more vesico-ureteric reflux. In this setting consult nephrologist and microbiologist.

For pain:

- Paracetamol, oral, 15 mg/kg/dose, 6 hourly as required.

Avoid NSAIDs.

REFERRAL

» Poor response to adequate therapy, i.e. persistent positive culture and/or fever.
» If complicated urinary tract infection, i.e. obstruction is suspected or renal failure.
» If recurrent urinary tract infections or repeated positive pure culture of any micro-organism.

6.1.3 NEPHROTIC SYNDROME (NS)

N04

DESCRIPTION

Nephrotic syndrome (NS) is a clinical syndrome associated with massive proteinuria due to increased permeability of t he glomerular basement membrane. Most children have primary (idiopathic) nephrotic syndrome associated with minimal change nephrotic syndrome (MCNS) or foca l segmental glomerulosclerosis (FSGS). In an undefined proportion of patients the disease is caused by genetic mutations in podocyte specific genes. Main causes of secondary nephrotic syndrome include infections (HIV, Hepatitis C), Systemic lupus erythematosis (SLE) and reflux nephropathy.

Main complications:

» Increased risk of infections with encapsulated organisms, *S. pneumoniae, E coli.* Chicken pox and measles are the main major viral infections.
» Hypercoagulable state: increased risk of arterial and venous thrombosis. Aggressive investigation and treatment may be ne cessary to prevent fatal pulmonary embolism.

DIAGNOSTIC CRITERIA

Clinical

» Massive proteinuria.
» Hypo-albuminaemia.
» Oedema.
» Hyperlipidaemia (hypercholesterolaemia).
» Usually normal blood pressure.
» Transient microscopic haematuria and/or hypertension in 25% of children.
» Usually normal renal function.

Investigations

» Urine test strips: ≥ 3+ proteinuria; may have trace to 1+ haematuria.
» Spot random urine sample protein:creatinine ratio: > 0.2 g/mmol.
» Urine microscopy: hyaline and lipid casts. May have occasional red and white blood cells.
» Serum albumin: < 25 g/L.
» S-urea and creatinine and electrolytes usually normal.
» S-cholesterol: increased.
» Investigations to exclude secondary causes of nephrotic syndrome, including: ASO and Anti-DNAseB titre, hepatitis Bs antigen, hepatitis C antibody, RPR, HIV and CMV antibodies.
» S-complement.
» Antinuclear factor antibody and anti-dsDNA.

A presumptive diagnosis of MCNS can be made in children:

» who are 2–6 years old and who have:
  > normal blood pressure,
  > normal renal function,
  > only a trace/1+ haematuria, but no red cell casts,
  > normal complement levels, and
  > in whom secondary causes have been excluded.

GENERAL AND SUPPORTIVE MEASURES

» Monitor fluid balance.
» Monitor urine output strictly and weigh daily (1 kg = 1 L of fluid).
» Assess hydration status.
» Suspect hypovolaemia in the presence of hypotension, small pulse volume and cold extremities.
» Normovolaemic: normal moist mucosa and normal blood pressure with well perfused limbs.
» Replace ongoing extra-renal losses as for dehydrated child e.g. oral rehydration for gut losses, etc.

Continued weight gain or anuria is an indication for referral.

» Dietary measures:
  > Do not restrict oral fluid intake,
  > Restrict salt intake in all patients. No salt added during preparation of food, no salt on the table during meals and restrict all salt preserved foods.
  > Limit intake of saturated fat.
  > Normal energy intake.
  > Normal protein diet for all with normal renal function.

MEDICINE TREATMENT

Specific treatment of causative conditions:

Where a treatable underlying cause exists this should be treated e.g.:

» HIV infection.
» Syphilis infection.
» SLE.
» Streptococcal infection.

If hypovolaemic:

- Sodium chloride 0.9%, IV, 20 mL bolus, immediately over 10 minutes.

Replace ongoing extra-renal losses as for dehydrated child e.g. oral rehydration solution for gut losses, etc.

For short term symptomatic treatment of oedema in patients with anasarca, volume contraction and oliguria and for the short term management of patients with congenital nephrotic syndrome:

- Albumin, human 20% (salt free), IV, 1 g/kg (i .e. 5 mL/kg) administered over 5 hours on 2 consecutive days.

AND

- Furosemide, IV, 2 mg/kg, slow IV infusion over 5 hours, i.e. 0.4 mg/kg/hour.

For patients with oedema and hypervolaemia:

- Furosemide, oral, 2 mg/kg/dose, 12 hourly.

AND

- Potassium chloride, oral, 5 mL 8 hourly.
  - o Monitor serum potassium.

For patients with intractable oedema who fail to improve with furosemide treatment only:

ADD

- Hydrochlorothiazide oral, 1 mg/kg, once daily.
  - o Do not exceed 12.5 mg daily.

Severe ascites:

- Spironolactone, oral 1.5 – 2.5 mg/kg/dose, 12 hourly.

All children with non-remitting nephrotic syndrome:

- Multivitamin, oral, 5 mL daily.
  (Formulation to include pyridoxine, other B vitamins, vitamin C 30 mg and vitamin D 400 IU)
- Folic acid, oral, 5 mg daily.
- Calcium (elemental), oral, 10–15 mg/kg/dose, 12 hourly.
  - o Maximum dose: 1 000 mg (1 g) daily.
  - o Calcium carbonate 420 mg = 168 mg elemental calcium.

Give all children with non-remitting nephrotic syndrome renoprotective treatment as for patients with chronic renal failure

▪ **ACE inhibitor**

An ACE inhibitor is given to decrease proteinuria, irrespective of presence or absence of systemic hypertension.
Begin with low dosage and titrate against response and blood pressure.

- Enalapril, oral, 0.1 mg/kg once daily.
  - o Increase dose to 0.5 mg/kg/day, as a single dose or two divided doses.
  - o Monitor for adverse effects: hyperkalaemia (increased risk when potassium sparing diuretic is used simultaneously) and acute renal failure (increased risk in children with impaired renal function or volume depletion)
  - o Do not use if estimated CrCl < 30 mL/minute.

Cholesterol lowering drugs
Are not indicated in children with steroid responsive nephrotic syndrome. For these patients only provide dietary advice.

For children > 8 years who have non-remitting nephrotic range proteinuria and persistent cholesterol levels > 7 mmol/L:

- HMGCoA reductase inhibitors (statin), e.g.:
- Simvastatin, oral, 10 mg at night.

Immunisation

**Do not give live vaccines to patients receiving steroid and other immunosuppressive treatment.**

Give all other EPI vaccines according to the schedule.

<u>Once in remission:</u>
Provide all routine vaccinations.

In children > 2 years:

- Pneumococcal vaccine (polysaccharide), IM, 0.5 mL as a single dose.
- Varicella-zoster vaccine, SC, 2 doses 6 weeks apart.

Check immunity against Hepatitis B.
In the absence of any immunity, vaccinate as for any non-immune individual.

- Hepatitis B vaccine, IM, 1 mL, 3 doses one month apart.
    - o If the antibody level is considered non-protective or insufficient, give 2 booster doses one month apart.

Antibiotics
Patients with anasarca have increased risk of pneumococcal infections, particularly spontaneous pneumococcal peritonitis.

For patients with anasarca:

- Phenoxymethylpenicillin, oral, 125–250 mg, 12 hourly.

Corticosteroids
Initiate corticosteroid treatment only in consultation with a specialist. In the absence of a histological diagnosis empiric steroid treatment should only be given to children with presumed minimal change nephrotic syndrome.
A rapid response to steroid treatment is usually indicative of MCNS. Patients with clinical features and/or laboratory results which suggest a diagnosis other than MCNS should be referred for kidney biopsy before steroid treatment is given, i.e. patients with initial macroscopic haematuria, persistent hypertension, persistent low C_3 and renal function impairment.

Initial treatment (first course of steroid treatment)

Start with high dose prednisone which is continued for 4 weeks. If patient is in remission after 4 weeks, start tapering the dose over **the following 4 months**.

- Prednisone, oral, 2 mg/kg/dose as a single dose in the morning for 4 weeks.
  - o Maximum dose: 60 mg daily.
  - o Taper dose over next 12 weeks as follows:
    - 2 mg/kg/dose as a single dose on alternate mornings for 4 weeks.
    - 1.5 mg/kg/dose as a single dose on alternate mornings for 4 weeks.
    - 1 mg/kg/dose as a single dose on alternate mornings for 4 weeks.
    - 0.5 mg/kg/dose as a single dose on alternate mornings for 4 weeks.

A short initial course i.e. total course of 8 weeks vs. 20 weeks, is associated with more frequent relapses. Relapses occur in up to 85% of all children with MCNS.

If the patient fails to achieve remission after 4 weeks of treatment, continue with the high dose for another 4 weeks (maximum of 8 weeks). Patients who fail to go into remission after 8 weeks of steroid treatment are considered steroid resistant and should be referred for kidney biopsy.

Note:

» For practical reasons a dipstick test is usually performed on a spontaneously voided urine sample instead of a 24-hour urine sample.
» Test urine every morning during cortisone treatment.
» Dipstick test should be negative for minimum of 3 consecutive mornings before decreasing the dose.
» If proteinuria recurs, go back one step in the suggested dose for a few more days before again attempting to decrease the dose.
» Some patients do not understand alternate day treatment schedules in which case daily dose of prednisone is given instead of alternate days.

Definitions

» **Remission**: No/trace protein on urine dipstick test for 3 consecutive days (spot sample urine protein:creatinine ratio < 0.02 g/mmol).
» **Steroid-sensitive NS**: No/trace protein on dipstick test for 3 consecutive days within 4 weeks after start of standard oral prednisone therapy.
» **Steroid-resistant NS**: Failure to achieve remission in spite of maximum 8 weeks' treatment with prednisone 2 mg/kg/day. (Spot sample urine protein:creatinine ratio > 0.02 g/mmol).
» **Relapse of NS**: 3+ proteinuria on urine dipstick test or urine protein:creatinine ratio > 0.2 g/mmol for 3 consecutive days.
» **Frequently-relapsing NS**: two or more relapses per 6 months or ≥ 4 per 12 month period.
» **Steroid-dependent NS:** Relapse develops during tapering of steroid treatment or within 2 weeks after stopping treatment.

Long-term corticosteroid treatment suppresses adrenal function. Therefore additional steroids or steroid supplementation is necessary during periods of acute stress, e.g. surgery or septic shock.

Treatment of relapse

» Always start with high prednisone dose, given daily for one week.
» Dipstick test should be negative for minimum of 3 consecutive mornings before the dose is decreased.
» If proteinuria recurs, go back one step in the suggested dose for a few more days before again attempting to decrease the dose.
» Schedule is similar as initial course, but for shorter period of 8 weeks in total.

<u>Schedule for relapse</u>

- Prednisone, oral, 2 mg/kg/dose as a single daily dose for minimum of one week. Then taper dose as follows:
  - 2 mg/kg/dose as a single dose on alternate mornings for 2 weeks
  - 1.5 mg/kg/dose as a single dose on alternate mornings for 2 weeks
  - 1 mg/kg/dose as a single dose on alternate mornings for 2 weeks
  - 0.5 mg/kg/dose as a single dose on alternate mornings for 2 weeks

Second-line immunosuppressive treatment

» Second line immunosuppressive treatment is indicated in children with steroid sensitive nephrotic syndrome, i.e. frequently-relapsing NS, steroid-dependent NS and in those with steroid toxicity. Kidney biopsy is preferably done before second line immunosuppressive treatment is started due to the risks associated with this treatment.
» It should only be prescribed after consultation with a paediatrician. It remains the prescriber's responsibility to monitor the patient at regular intervals for side effects of treatment. Full blood count, urea, creatinine, electrolytes and albumin needs to be done every 10–14 days throughout the course of treatment.
» Second line immunosuppressive treatment for steroid sensitive nephrotic syndrome should only be started when the urine dipstick test is negative.
» It is always given in combination with steroid treatment.

- Cyclophosphamide, oral, 2 mg/kg/dose once daily for 12 weeks.
  - o Ensure adequate fluid intake to avoid haemorrhagic cystitis.

Children with steroid resistant nephrotic syndrome do not benefit from treatment with cyclophosphamide, but may respond to treatment with cyclosporine or tacrolimus.

REFERRAL

- » All with congenital nephrotic syndrome.
- » All with clinical features and/or laboratory results which suggest a diagnosis other than MCNS, e.g. i nitial macroscopic haematuria, persistent hypertension, persistent low C_3 and renal func tion impairment.
- » Patients with steroid resistant nephrotic syndrome.
- » All patients before second line immunosuppressive treatment is prescribed.

6.1.4 ACUTE KIDNEY INJURY (RENAL FAILURE, ACUTE)

N17.9

DESCRIPTION

Acute kidney injury (AKI) is a syndrome characterised by a rapid decline in glomerular filtration rate and retention of fluid and nitrogenous waste products. It often presents as a cont inuum of volume responsiveness "prerenal AKI" up to a point of volume unresponsiveness. AKI is classified as prerenal, renal and postrenal failure.

Levels of AKI is d efined by pRIFLE criteria (mnemonic p=paediatric, Risk, Injury, Failure, Loss and End Stage Renal Failure).

Paediatric modified RIFLE (pRIFLE) criteria

Level	Estimated creatinine clearance (eCrCl)*	Urine output
1	↓ eCrCl by 25 %	<0.5 mL/kg/hour for 8 hours
2	↓ eCrCl by 50 %	<0.5 mL /kg/hour for >16 hours
3	↓ eCrCl by 75%	<0.5 mL /kg/hour for >24 hours or anuria for 12 hours

The previous method of measuring creatinine clearance using 24 hour urine sample is not recommended due to the difficulty of obtaining an accurate 24 hour urine collection in children.

A calculated glomerular filtration rate can be ascertained using the height of the child in cm, the serum creatinine (micromol/L) and a factor "K" = 40 for all children older than 1 year and up to puberty. (**Counahan-Barratt formula**)

$$\text{eCrCl (mL/min/1.73 m}^2\text{)} = \frac{= [40 \times \text{height (cm)}]}{\text{S-creatinine (micromol/L)}}$$

Normal values for GFR in children:

Age	Mean GFR (ml/min/1.73/ m^2)	Range
Birth	20	
7 days	40	25–60
1 month	50	30–70
6 months	75	40–100
12 months	115	65–160
2–12 years	125	90–165

DIAGNOSTIC CRITERIA

Clinical

» In neonates exclude congenital abnormality of the urinary tract.
» Oliguria is the most common manifestation, i.e. :
  Neonates: output < 1 mL/kg/hour.
  Older children: output ≤ 0.3 mL/kg/hour.
» Prerenal: shock and dehydration.
» Postrenal: exclude obstruction, e.g. palpable bladder.
» Intrinsic kidney disease: oedema, volume overload, hypertension.
» Signs of underlying infection/septicaemia, e.g. fever, skin rash, etc.

Investigations

» Urine macroscopic appearance: brownish with acute tubular necrosis.
» Urine test strips: haematuria, proteinuria indicative of glomerular disease; leucocytes and nitrites in favour of pyelonephritis.
» Urine microscopy: red blood cell casts, leukocyte, hyaline and granular casts.
» Urine culture to exclude pyelonephritis.
» Urine biochemistry:

	Pre-renal failure	Intrinsic renal failure
U-Osmol (mOsmol/L)	↑ > 320	equal to serum Osmol
FeNa % *	< 1 %	≥ 3 %

$$\text{Fractional excretion of sodium\%} = \frac{\text{Urinary sodium}}{\text{Urinary creatinine}} \times \frac{\text{Serum creatinine}}{\text{Serum sodium}} \times 100$$

*FeNa % becomes an invalid test for pre-renal failure if the child has received furosemide.

Note:
Serum creatinine is measured in micromol/L and urine creatinine in millimol/L. To convert micromol/L to millimol/L ÷ by 1000.

» Ultrasound of kidneys and bladder.
» Serum urea, urate, creatinine, electrolytes and osmolarity, glucose, calcium, phosphate and albumin.
» Typical biochemistry: hyperkalaemic metabolic acidosis, hyponatraemia, hypocalcaemia, hyperphosphataemia.
» Full blood count, differential and platelet count.

» Clotting profile.
» Cultures and DIC workup as indicated.
» ECG on to exclude life threatening hyperkalaemia.
» Chest X-ray to evaluate cardiomegaly, pleural effusions and pulmonary oedema.

GENERAL AND SUPPORTIVE MEASURES

» Treat the underlying cause.
» Monitor fluid intake and output, blood pressure.
» Weigh daily.
» Nutritional support.
  > High-energy diet. Give supplementary nasogastric feeds, if required. Infants should preferably be given breast feeds or an infant milk formula.
  > Daily requirements:
    - protein: 1 g/kg maximum
    - carbohydrate: 2–3 g/kg
    - fat: 2 g/kg
» Restrict NaCl, potassium and phosphate intake.
» Restrict protein intake when S-urea > 25 mmol/L.

Avoid nephrotoxic or renally excreted medicines, e.g.
NSAIDs, aminoglycosides, vancomycin, cough and cold mixtures,
radiocontrast drugs.

» Fluid management:
  > Depends on volume status, urine output and extra renal losses.
  > Never use a potassium-containing solution in an anuric patient.
  > Only use parenteral fluids if oral intake is not possible.
» Fluid management according to fluid status:
  > **Pulmonary oedema plus oliguria/anuria**: Do not give fluid.
  > **Hydrated anuric patient without extra-renal fluid losses**: Oral fluid to replace insensible water losses only.

  Insensible water loss is calculated as:
    - Neonate and young baby: 30–40 mL/kg/day.
    - Older children: 25 mL/kg/day (400 mL/m^2/day).

  In exceptional circumstances, if parenteral fluid is required, use an electrolyte free solution i.e. dextrose 5% or 10%, IV.

  Normally hydrated plus oliguria: Oral fluid intake to replace insensible water loss plus urine output of previous 24 hours.

> **Dehydrated, oliguric and ongoing extra-renal fluid losses**: Volume for replacement according to losses.
>
> Replace fluid losses with an appropriate solution which mirrors losses e.g.:
> - for diarrhoea: ½ Darrows/dextrose 5%, IV or oral rehydration solution;
> - for vomiting/gastric fluid losses: sodium chloride 0.9%/dextrose 5%.

> **Normally hydrated plus normal urine output:** Give normal fluid intake.

> **Shock**: See section 1.1.7: Shock.

> **Polyuria**, (urine output > 4 mL/kg/hour): which usually occurs during the recovery (diuretic) phase of acute tubular necrosis: Replace fluid and electrolyte losses with ½ Darrows/dextrose 5%, IV.
>
> Volume to replace is equal to urine output of preceding 12 hours. Fluid balance is critical. Assess at the minimum every 12 hours to make appropriate changes to fluid prescription.

MEDICINE TREATMENT

Hyperkalaemia

Monitor ECG for signs of hyperkalaemia.
Discontinue all sources of intake of potassium.
Treat when serum potassium > 6.5 mmol/L.
Monitor response to treatment and adjust accordingly.

- Salbutamol, solution, 2.5–5 mg/dose, nebulise over 20 minutes.
  - 0.5–1 mL salbutamol in 1 mL sodium chloride 0.9%.

OR

- Salbutamol, IV, 4 mcg/kg in 5 mL water administered over 30 minutes.

- Sodium bicarbonate 4.2%, IV, 4 mL/kg administered over 4 hours.
  - Do not mix calcium and sodium bicarbonate-containing solutions.

- Sodium polystyrene sulphonate, oral/rectal, 1 g/kg in dextrose water.

- Calcium gluconate 10 %, IV, 0.5–1 mL/kg/dose slowly over 3–5 minutes.

- Dextrose 50%, IV, 2 mL/kg over 20 minutes **with/without** insulin, soluble, 0.1 units/kg.
  - Monitor for hypoglycaemia hourly if insulin is used.

If hyperkalaemia persists despite above treatment refer the patient urgently for dialysis.

Other complications

Metabolic acidosis: serum pH ≤ 7.1

- Sodium bicarbonate 4.2 %, IV, 4 mL/kg administered over 2–4 hours.
  - o Do not mix calcium and sodium bicarbonate containing solutions.

Hypertension

See section 4.11: Hypertension in children.

Infection

Avoid nephrotoxic antibiotics.

Uraemic convulsions

See section 13.4: Seizures, febrile.

Refer for urgent dialysis

Exclude specific causes of convulsions, e.g. hypoglycaemia, hyper- or hyponatraemia, hypocalcaemia or hypertension and treat accordingly.

Anaemia

For acute blood loss/active haemolysis and Hb < 7 g/dL:

- Packed red cells, IV, 10 mL/kg administered over 6 hours.

Pulmonary oedema, volume overload and hypertension

Do not give fluid to anuric patients with pulmonary oedema.

Pulmonary oedema is an indication for dialysis.

Intubate and initiate positive pressure ventilation as necessary.

- Furosemide, IV, 2–5 mg/kg administered over 5 minutes.
  - o Maximum daily dose: 8 mg/kg/24 hours.
- Morphine, IV, 0.1 mg/kg.
  - o Repeat after 4 hours, if required.
- Oxygen, 100%, 2–3 L/minute by nasal cannula.

REFERRAL

Urgent for dialysis when:

» Fluid overload is causing pulmonary oedema.
» Anuria > 24 hours.
» Central nervous system signs, e.g. convulsions or coma.
» Uraemic bleeding diathesis.
» Uraemic pericarditis.
» Hyperkalaemia or hyponatraemia not responding to conservative treatment.
» Persistent metabolic acidosis pH < 7.1 or serum bicarbonate < 10 mmol/L.
» Uncontrollable hypertension.
» Severe hyperphosphataemia and hypocalcaemia.

6.1.5 CHRONIC KIDNEY DISEASE (RENAL FAILURE, CHRONIC)

N18.9

DESCRIPTION

Chronic kidney disease (CKD) is defined as: "evidence of structural or functional kidney abnormalities (abnormal urinalysis, imaging studies or histology) that persist for at least 3 months, with or without a decreased glomerular filtration rate (GFR), as defined by a GFR of less than 60 mL/min/1.73 m^2"

It is characterised by a progressive decline in renal function to end stage renal failure due to progressive loss of functioning glomeruli and is accompanied by the onset or worsening of proteinuria. In children the glomerular filtration rate (estimated eGFR, estimated Creatinine Clearance eCrCl) is calculated using the Counahan-Barratt formula:

$$\text{eCrCl (mL/min/1.73 m}^2\text{)} = \frac{= [40 \times \text{height (cm)}]}{\text{S-creatinine (micromol/L)}}$$

Staging of chronic kidney disease (KDQOI definition)

Stage	*eGFR (mL/min/1.73 m^2)	Features
1	≥ 90	Renal parenchymal disease present.
2	60–89	Usually asymptomatic – biochemical abnormalities present.
3	30–59	Biochemical abnormalities and poor growth, poor appetite.
4	15–29	
5	<15 (ESRF)	End stage renal failure.

*eGFR: estimated glomerular filtration rate

DIAGNOSTIC CRITERIA

Renal function may deteriorate without clinical symptoms.

- » Children are likely to present with acute on chronic renal failure during episodes of acute intercurrent illness.
- » Poor weight gain and stunting.
- » Poor appetite, chronic constipation, polydipsia and polyuria.
- » Children with renal tubular disorders or bilateral renal dysplasia have obligatory salt wasting and are often unable to concentrate urine. This may result in severe dehydration and metabolic acidosis if they do not have free access to water.
- » May present with tachypnoea mimicking acute "respiratory distress" to compensate for metabolic acidosis.
- » Chronic anaemia.
- » Renal osteodystrophy, i.e. bone pain and skeletal deformities.

- » Volume overload: oedema, hypertension, heart failure, pulmonary oedema.
- » Uraemic symptoms and signs: nausea, vomiting, pruritis, brownish skin pigmentation, uraemic frost.
- » Bleeding tendency (mucosa).
- » Convulsions due to hyponatraemia, hypernatraemia, hypocalcaemia, uraemia or hypertension.

Investigations

- » Urine:
  - > Protein:creatinine ratio is usually increased (normal < 0.02 g/mmol).
  - > Iso-osmolar, i.e. urine Osmol ± 300–350 mOsm/L (normal maximal urine concentration > 1000 mOsmol/L).
- » Urine volume may be:
  - > normal, or
  - > increased (polyuria): > 4 mL/kg/hour, or
  - > decreased (oliguria): < 1.0 mL/kg/hour.
- » Urine test strips:
  - > May be normal or reveal proteinuria, haematuria, glycosuria.
  - > Nitrites and leukocytes may indicate UTI. Do urine MCS.
- » Urine microscopy
  - > May be normal or reveal casts.
  - > Pus cells, leukocyte casts and bacteria may indicate UTI. Do urine MCS.
- » Serum urea:
  - > Increased, depending on hydration, nutritional state and protein intake.
- » Serum creatinine is a better indicator of renal function than serum urea:
  - > Is influenced by age of child and muscle bulk.
  - > It may be only mildly increased in a malnourished child with little muscle bulk despite advanced renal failure (serum creatinine only starts increasing once renal function has fallen to less than half normal).
- » Serum electrolytes:
  - > Hyperkalaemia.
  - > Hyperchloraemia and decreased bicarbonate.
- » Calcium, phosphate and ALP:
  - > Decreased calcium.
  - > Increased phosphate.
  - > Increased ALP.
- » Plasma parathyroid hormone:
  - > Increased.
- » Renal sonar:
  - > To exclude obstruction.
  - > Small shrunken kidneys are indicative of chronic renal failure.

There is no place for renal biopsy in patients with end stage renal failure.

GENERAL AND SUPPORTIVE MEASURES

» Determine and treat the underlying cause of chronic renal failure.
» Monitor fluid intake and output, and blood pressure.
» Weigh daily.
» If in respiratory distress due to volume overload:
  > Place in sitting position.
  > Give oxygen, 100%, 2–3 L/minute by nasal prongs.
» Dietary management:
  > Monitor potassium closely.
  > Limit potassium intake if serum potassium > 5.5 mmol/L.
  > Restrict fruit juices, dried fruit, all citrus fruits, bananas, guavas and tomatoes.
  > All vegetables should either be soaked for 24 hours before cooking or water should be decanted twice during cooking.
  > Restrict phosphate once serum phosphate reaches or exceeds the upper limit of normal for age, usually >1.8 mmol/L and when GFR <70 mL/min/1.73m^2.
  > Limit dairy products and other foods with high phosphate content like grains and cereals, carbonated cool drinks, etc.
  > Do not limit protein intake.
  > Restrict salt intake. No salt added during preparation of food, no salt on the table during meals and restrict all salt preserved foods.
    Generally, salt is restricted for hypertensive, oedematous patients, but not for patients with salt losing nephropathies who are polyuric, unless they are hypertensive.
  > High-energy diet with supplementary nasogastric feeds or nocturnal fluids for children with poor appetite, polyuria/nocturia and with inadequate intake to maintain growth.
» Fluid management:
  > Depends on underlying kidney disease.
  > Use body weight to guide fluid prescription.
  > Only use parenteral fluids if oral intake is not possible.
  > Children with tubular abnormalities may be unable to concentrate their urine and therefore require free access to water.
  > **Anuric**: Fluid to replace insensible water losses only.
    Use an electrolyte free solution i.e. dextrose 5% or 10%, IV.
    Insensible water loss is calculated as:
    - Neonate and young baby: 30–40 mL/kg/day.
    - Older children: 25 mL/kg/day (400 mL/m^2/day).
  > **Oliguric with oedema and hypertension**: Total volume fluid allowed calculated as:
    Insensible water loss is calculated as:
    - Neonate and young baby: 30–40 mL/kg/day.
    - Older children: 25 mL/kg/day (400 mL/m^2/day).

Use an electrolyte free solution i.e. dextrose 5% or 10%, IV.
plus
50% of urine output.
plus
Extra-renal losses (volume for volume).
Use a potassium-free solution, e.g. sodium chloride 0.45%.
Once euvolaemic, give same fluids as above to replace 100% of urine output.

> **Dehydrated and hypotensive**: Give sodium chloride 0.9%, IV bolus immediately and re-assess.
Repeat bolus, if necessary.
Strictly monitor urine output and fluid losses.

MEDICINE TREATMENT

Avoid nephrotoxic agents and appropriately adjust renally excreted medicines, e.g.<br>NSAIDs, aminoglycosides, vancomycin, amphotericin B, radiocontrast drugs.

Vitamins and minerals

- Multivitamin, oral, 5 mL daily.
  (Formulation to include pyridoxine, other B vitamins, vitamin C 30 mg and vitamin D 400 IU).

AND

- Folic acid, oral, 5 mg daily.

For management of hyperphosphataemia/osteodystrophy and hyperparathyroidism:
In combination with restricted dietary intake of phosphate:

- Calcium carbonate, oral, 1–4 tablets chewed 8 hourly with meals.
  - o 1 tablet is equivalent to 0.168 g elemental calcium.
- Alfacalcidol oral, 0.25 mcg daily. Specialist initiated.
- If serum phosphate is > 2.5 mmol/L, treat the hyperphosphataemia first to decrease to below < 1.8 mmol/L before beginning the alfacalcidol (to avoid metastatic calcification).

In patients with serum calcium < 2.2 mmol/L start alfacalcidiol early:

- Alfacalcidol oral, 0.25 mcg, initially twice weekly. (Specialist initiated)
  - o Increase dose as necessary to maintain serum calcium in upper normal range.

Chronic metabolic acidosis

If serum bicarbonate < 18 mmol/L:

- Sodium bicarbonate, oral, 1 mmol/kg/dose 2–3 doses per day after meals.
  - Adjust according to response.

  Note:
  The intravenous formulation can be given orally.

Hyperkalaemia

Discontinue all medicines that may cause hyperkalaemia, e.g. potassium sparing diuretics, spironolactone, ACE inhibitors.
Exclude volume depletion as an underlying cause for hyperkalaemia.

If serum potassium remains > 5.5 mmol/L:

- Sodium polystyrene sulphonate, oral/rectal, 1 g/kg/dose in dextrose water, once or twice daily.
  - Treat accompanying metabolic acidosis.

Anaemia

Ensure adequate intake of haematinics.
Ensure adequate iron stores. Measure ferritin, transferrin, transferrin saturation and total iron binding capacity.
Avoid transfusions if possible due to risk of developing antibodies in a patient who may be a potential candidate for renal transplantation.

If a patient has symptomatic anaemia, haemoglobin usually < 7g/dL:

- Packed cells, IV, 10 mL/kg administered over 6 hours.

If the patient has a persisting haemoglobin level < 8g/dL despite correction of possible deficiencies of iron, folic acid or vitamin B_{12} treatment, start recombinant human erythropoietin (rHuEPO) in consultation with a paediatric nephrologist.
Note:
Blood pressure must be controlled before starting rHuEPO treatment.
Dose of erythropoietin is gradually increased according to increase in haemoglobin. Target haemoglobin is 10–12 g/dL.

- Erythropoietin, SC, 75 units/kg/week in divided doses 2–3 times a week.
  - Monitor Hb levels every 4 weeks.
  - Adjust dose until target haemoglobin level of 12 g/dL is reached. Continue with this dose.
  - If the Hb level is increasing, do not change dose.
  - If the Hb level remains unchanged, increase by 25% at 4-week intervals until maximum dose of 300 units/kg/week is reached.
  - If Hb level increases > 12 g/dL, stop treatment for one week. Thereafter continue with 25% less than previous dose per week.

For persistent anaemia:
Refer to tertiary centre for nephrologist assessment.

Hypertension
See section 4.11: Hypertension in children.

Dyslipidaemia
Dyslipidaemia may contribute to the progression of chronic kidney disease, particularly in children with nephrotic syndrome. Hypertriglyceridaemia and abnormal apolipoprotein metabolism is a feature of CRF. Dietary intervention is necessary, including limiting saturated fat and cholesterol intake.

For children > 8 years with persistent total cholesterol levels > 7 mmol/L:
- HMGCoA reductase inhibitors (statins), e.g.:
- Simvastatin, oral, 10 mg at night.
  - Maximum dose: 20 mg at night.

Refer for advice on management.

Renoprotective treatment
All children with persistent nephrotic range proteinuria and GFR > 30 mL/minute:
- ACE inhibitor. (with nephrologist supervision)
- Enalapril, oral, 0.1 mg/kg/dose, once daily.
  - Increase dose to 0.5 mg/kg/day, as a single dose or two divided doses.
  - Monitor for adverse effects: hyperkalaemia (increased risk when potassium sparing diuretic is used simultaneously) and acute renal failure (increased risk in children with impaired renal function or volume depletion).
  - May cause hyperkalaemia, worsening metabolic acidosis and declining renal function while reducing proteinuria.
  - Monitor serum urea and electrolytes, i.e. serum potassium and bicarbonate, and renal function within 7 days.
  - If serum creatinine has doubled, check hydration status, stop diuretics and halve the dose of ACE inhibitors.
  - If renal function does not improve, or hyperkalaemia > 5.5 mmol/L persists, stop ACE inhibitor treatment.

Immunisation
Give all EPI vaccines according to the schedule.
Provide all routine vaccinations or missing vaccinations in older children.
Check immunity against chicken pox and Hepatitis B.

In children > 2 years of age:
- Pneumococcal vaccine (polysaccharide), IM, 0.5 mL as a single dose.

In the absence of any immunity against chickenpox give:
- Varicella-zoster vaccine, SC, 2 doses 6 weeks apart.

In the absence of immunity against Hepatitis B, vaccinate as for any non-immune individual.

- Hepatitis B vaccine, IM, 1 mL, 3 doses one month apart.
  - o If the antibody level is considered non-protective or insufficient, give 2 booster doses one month apart.

REFERRAL

» All children with chronic kidney disease, including those with:
  > persistent proteinuria or haematuria,
  > inherited kidney diseases,
  > renal tubulopathies,
  > congenital malformation of kidneys,
  > chronic bilharziasis.

» Patients with dyslipidaemia or hypercholesterolaemia.

6.1.6 ENURESIS

R32

DESCRIPTION

Enuresis is bedwetting after the age of 5 years.
Primary monosymptomatic enuresis refers to incontinence during sleep only. It is of great importance to differentiate between monosymptomatic enuresis and enuresis with associated bladder dysfunction during daytime, because the treatment of these two conditions is totally different.

Enuresis is a benign condition with a spontaneous annual resolution rate. Intervention must carry no risk or have minimal side effects. The cure rate of "treatment" should be significantly greater than the spontaneous cure rate before it can be considered effective.

DIAGNOSTIC CRITERIA

Clinical

» Clinical evaluation of all children with enuresis should begin with a structured interview.

» Exclude symptoms of underlying systemic disease e.g.:
  > diabetes mellitus,
  > diabetes insipidus,
  > urinary tract infections,
  > neurological disturbances,
  > structural abnormalities.

Investigations

- » Urine dipstick test in all patients as a screening test.
- » Urine pH and concentration on first morning urine sample after 10 hour overnight fast.
- » If any of the two above is abnormal, further investigations are necessary to exclude organic causes.
- » Ultrasound investigation may be necessary to identify structural abnormalities of the kidneys, pelvis and ureters.

GENERAL AND SUPPORTIVE MEASURES

- » Motivate, counsel and re-assure child and parents.
- » Advise against punishment and scolding.
- » Regular fluid intake 6–8 glasses of water spread throughout the day.
- » Last drink 1 hour before going to bed.
- » Regular voiding 5–6 times per day.
- » Diapers should never be used as this will lower the self esteem.
- » Bell and pad system is effective but only use in children > 7 years and who are well motivated.
- » Consider behaviour modification and bladder training exercises in children with daytime wetting.

MEDICINE TREATMENT

If general measures have failed after 6 months, consult with a specialist for consideration of desmopressin.

For short term treatment only for a patient with enuresis and low self esteem:

- Desmopressin, oral, 200–400 mcg at night for 3 months. (Specialist consultation)
    - o Adverse effects include fluid retention, hyponatraemia and cerebral oedema.

In children over 5 years with voiding dysfunction and accompanying daytime wetting:

- Oxybutinin, oral, 0.1–0.3 mg/kg/dose 8 hourly.

REFERRAL

- » Suspected underlying systemic illness or chronic kidney disease.
- » Persistent enuresis in a child > 8 years.

CHAPTER 7

ENDOCRINE SYSTEM

7.1 DISORDERS OF SEXUAL DEVELOPMENT (DSD)

DESCRIPTION

The current terminology for neonates presenting with incomplete differentiation of the external genitalia is “disorder of sexual development”.

DIAGNOSTIC CRITERIA

Clinical

- » Severe forms of DSD present with one or more of the following:
  - > varying degrees of hypospadias,
  - > maldescent of one or both gonads, or
  - > atypical size of the phallus,
  - > scrotalisation of labia.
- » Simple hypospadias is easily recognised and is typically associated with palpable gonads. In this situation, assure the parents of male gender.
- » Suspect true hermaphroditism (XX, ovotesticular DSD) if one or both gonads are not palpable in association with hypospadias.
- » Suspect congenital adrenal hyperplasia in an infant with non-palpable gonads and genital ambiguity.

Investigations

- » Urgent urea/electrolytes and blood sugar to identify possible adrenal insufficiency.
- » Further investigations in the referral centre include:
  - > Genitourinary imaging (e.g. ultrasound).
  - > Chromosome studies for hermaphroditism.
  - > Elevated 17-hydroxyprogesterone level to confirm diagnosis of adrenal hyperplasia.

GENERAL AND SUPPORTIVE MEASURES

- » If assigning gender is unclear, refer parents to units experienced in managing these patients. Gender assignment in these infants should only be undertaken after extensive counselling and evaluation by a multidisciplinary team.
- » Urgently refer n eonates suspected of having congenital adrenal hyperplasia as a sa lt-losing crisis (characterised by a high potassium and a low sodium) may be life threatening.

MEDICINE TREATMENT

Congenital adrenal hyperplasia can present with an adrenal crisis. See section 7.3: Adrenal insufficiency, acute.

REFERRAL

» All cases for confirmation of the diagnosis, counselling and initiation and monitoring of treatment.
» Urgent all cases of congenital adrenal hyperplasia.

7.2 ADRENAL HYPERPLASIA, CONGENITAL

E25.0

DESCRIPTION

Autosomal recessive enzymatic defects of the cortisol biosynthetic pathways in the adrenal gland. The presentation depends on the severity and type of the enzyme defect.

DIAGNOSTIC CRITERIA

Clinical

» Neonates with disorder of sexual development (ambiguous genitalia).
» Adrenal insufficiency (e.g. acute circulatory collapse, hyponatraemia, hypoglycaemia) See section 7.3: Adrenal insufficiency, acute.
» Accelerated growth velocity or precocious pseudopuberty.

Investigations

See section 7.3: Adrenal insufficiency, acute.

» Elevated 17-hydroxyprogesterone in the serum.
» Elevated serum renin.

GENERAL AND SUPPORTIVE MEASURES

» Surgical correction of genital abnormalities after endocrine treatment.
» Psychological support for child and family.

MEDICINE TREATMENT

Glucocorticoid and mineralocorticoid replacement. To be initiated in consultation with subspecialist.

- Hydrocortisone, oral, 0.5 mg/kg/day in three divided doses. Specialist initiated.
    - o The morning dose should be given as early as possible.

- Fludrocortisone acetate, oral, 5 mcg/kg/day as single daily dose.
    - o Range: 50–200 mcg daily.

For salt losing patients:

- Sodium chloride, oral, 0.5–1 g for every 10 kg body weight per day.

Glucocorticoids are administered for life. Once growth is complete, prednisone may be given once or twice daily. Long-acting glucocorticoids are generally avoided in children because of potential growth suppression.
The dose is individualised by monitoring growth, bone age and hormonal levels.

Adolescent dose:

- Prednisone, oral, 10 mg/day in two divided doses.

REFERRAL

» All cases for confirmation of the diagnosis, counselling and initiation and monitoring of treatment.

7.3 ADRENAL INSUFFICIENCY, ACUTE

E27.4

DESCRIPTION

Acute failure of adrenal function, suspected when a patient presents with hypotension, hypoglycaemia, hyponatraemia, hyperkalaemia, and metabolic acidosis.

Patients on chronic steroid therapy are at risk for adrenal insufficiency.

Consider augmentation of the steroid dose during times of stress (fever, trauma, and surgery).

DIAGNOSTIC CRITERIA

Clinical

» Acute circulatory collapse. The features include:
  > tachycardia,
  > hypotension,
  > pallor,
  > poor peripheral perfusion,
  > cool clammy skin,
  > disturbed consciousness,
  > coma,
  > signs of dehydration, and
  > metabolic acidosis.
» A history of weakness, anorexia, vomiting, weight loss, salt craving, hyperpigmentation (primary adrenal insufficiency), auto-immune endocrinopathies and steroid-dependence.
» Ambiguous genitalia.
» Hyperkalaemia.
» Hypoglycaemia.
» Hyponatraemia.

Investigations

Take blood for estimation of

» Serum electrolytes and blood glucose.
» In all suspected cases, take a sample of clotted blood for estimation of plasma cortisol prior to treating the patient. Send this sample with the patient to the central hospital if laboratory facilities are not locally available.

MEDICINE TREATMENT

Stabilisation

For shock

- Sodium chloride 0.9%, IV, 20 mL/kg bolus as needed.

For hypoglycaemia

Dextrose 10%, IV, 2–5 mL/kg bolus as needed.

- Hydrocortisone, IV, 2 mg/kg immediately as a single dose.
  - o Follow with 0.5 mg /kg/dose every 6 hours.

Manage hyperkalaemia. See section 6.1.4: Acute kidney injury (Renal failure, acute).

Prevention

Patients on chronic steroid therapy are at risk for adrenal insufficiency during stressful situations e.g. sepsis, trauma, elective or emergency surgery. Augment the dose of steroids for the duration of stress.

For major stress:

- Hydrocortisone, IV, 2 mg/kg/day for the duration of the stress.

For minor stress, e.g. URTI:

- Hydrocortisone, IV, 1 mg/kg/day for 3 days.

**Adrenal insufficiency is a life threatening emergency**

REFERRAL

» All cases immediately after stabilisation.

7.4 DIABETES INSIPIDUS

E23.2/ N25.1

DESCRIPTION

Suspect diabetes insipidus in any child with polydipsia and polyuria. Infants may present with failure to thrive.

Central diabetes insipidus is due to deficiency of antidiuretic hormone.
Nephrogenic diabetes insipidus occurs if the kidney is unable to respond to antidiuretic hormone.

DIAGNOSTIC CRITERIA

» Pathological polyuria defined as excretion of > 1.5 L/m^2 of urine. In infants, the corresponding value is > 2.5 L/m^2.
» Serum osmolality > 300 mOsm/kg, with urine osmolality < 300 mOsm/kg is suggestive of diabetes insipidus.
» A positive water deprivation test. (Only conduct under specialist supervision)

MEDICINE TREATMENT

Central diabetes insipidus

- Desmopressin, oral, 50–300 mcg/day 12 hourly.

Older children:

- Desmopressin, oral, 50 mcg/day, starting dose.
  - o Titrate according to response. Use the lowest dose at which an antidiuretic effect is obtained.
  - o Maximum dose: 100 mcg/dose 8 hourly.

Infants or where oral administration is not feasible:

- Desmopressin, nasal solution, 5–10 mcg/day (0.05–0.1 mL), starting dose.
  - o Titrate according to response. Use the lowest dose at which an antidiuretic effect is obtained.
  - o Maximum daily dose: 30 mcg/day once or twice daily.

Note: The oral and nasal formulations have different absorption rates and therefore have different dosing

The patient must have a phase of urinary dilution or breakthrough urination before the next dose to ensure that water intoxication does not result.

Nephrogenic diabetes insipidus

If no response to desmopressin.

Treat the underlying cause.

- Hydrochlorothiazide, oral, 0.5–1 mg/kg/dose 12 hourly.
- Ibuprofen, oral, 5 mg/kg/dose 12 hourly.

REFERRAL

» All cases for evaluation.

7.5 DIABETES MELLITUS

DESCRIPTION

A syndrome of abnormal carbohydrate metabolism, associated with a relative or absolute impairment of insulin secretion with varying degrees of peripheral resistance to the action of insulin.

7.5.1 DIABETES MELLITUS, INSULIN DEPENDENT (TYPE 1)

E10

DESCRIPTION

Most diabetic children have type 1 diabetes, and:

- » have auto-immune destruction of the pancreatic beta cells as the underlying cause,
- » have an absolute requirement for insulin therapy,
- » will develop diabetic ketoacidosis (DKA) if not given insulin.

DIAGNOSTIC CRITERIA

- » Polydipsia.
- » Polyphagia.
- » Heavy glycosuria.
- » Weight loss or failure to gain weight.
- » Weakness or tiredness.
- » Recurrent protracted infections.
- » Random blood glucose of ≥ 11.1 mmol/L.
- » Polyuria – this can present as secondary enuresis in young children
- » Fasting blood glucose of ≥ 7.0 mmol/L – fasting is not usually needed for the diagnosis.
- » Ketonuria.
- » An oral glucose tolerance test is generally not needed.

GENERAL AND SUPPORTIVE MEASURES

- » Refer to a unit which is able to manage type 1 diabetic patients.
- » Educate child and caregiver about all aspects of the disease.
- » Medical alert bracelet should be worn at all times.
- » Follow-up by medical practitioner or at clinic/hospital at least every 3 months.
- » Monitor thyroid function annually.
- » Annual test for proteinuria in children > 12 years of age.
- » Annual eye examination in children > 12 years of age.

Diet: healthy lifelong eating habits

- » Refer a newly diagnosed patient and family to a dietician.
- » Principles of the prudent diet:
  - › Encourage children to reduce the intake of fats and salt and to increase dietary fibre content.
  - › Provide all diabetics with a meal plan, e.g. “constant carbohydrate meal plan” or “carbohydrates counting meal plan”. There is no one ‘diabetic’ diet. Individualise the diet giving consideration to usual eating habits and other lifestyle changes required.

> Six main nutrition factors contribute to better sugar control, i.e. lower HbA1c levels. These are:
  1. Following a meal plan. Keep day-to-day intake consistent.
  2. Avoiding extra snacks that are not part of the meal plan.
  3. Avoiding over-treatment of low blood sugars (hypoglycaemia).
  4. Prompt correction of high blood sugars.
  5. Adjusting insulin levels for meals in patients using the "carbohydrates counting meal plan".
  6. Consistency of night snacks.

CONSTANT CARBOHYDRATE MEAL PLAN

Consistency is the key. The amount of insulin, usually two or three doses per day, is kept relatively constant from day-to-day. Carbohydrates should be manipulated to match the relatively constant insulin dose. **If able to count carbohydrates, give 1 unit of insulin per 15 g of carbohydrate.**
The amount of carbohydrates (types can vary) is kept about the same for each meal and each snack from one day to the next.

As part of the educational process, the family must get used to reading food labels to know the grams (g) of carbohydrates being eaten. The dietician may suggest a range of carbohydrates for each meal.

Examples of carbohydrate content of some foods
The following foods have 15 g of carbohydrate per serving:
1 cup = 250 mL

FOOD	SERVING SIZE
Beans (cooked, canned)	½ cup
Bread (white, brown)	1 slice
Pap (cooked)	¼ cup
Soft maize porridge (cooked)	½ cup
Pasta (cooked)	½ cup
Potato (mashed)	½ cup
Rice (cooked)	⅓ cup
Apple (small)	1
Fruit juice	½ cup
Grapes	½ cup (12 medium grapes)
Orange (small)	1
Banana (small)	1
Milk	1cup
Yoghurt (low fat, unsweetened)	1 cup
Pizza (thin-crust, medium size)	1/8 of medium pizza
Potato slap chips (not crisps)	8–12

- Tailor the advice to the patients' lifestyle, economic circumstances and usual diet and, where possible, avoid drastic changes.
- Do not forbid any particular food as this may lead to disturbed attitudes to food, e.g. carbohydrates are not forbidden but can be taken before exercise, incorporated into a main meal or used as a source of energy during illness when children have a poor appetite.
- Diet should provide adequate nutrition for growth and development.

  Dietary composition

  It is recommended that:
  - approximately 35% of dietary energy should be derived from mono- and polyunsaturated fat,
  - 15% from protein,
  - 50% from carbohydrates. Carbohydrates should always provide at least 40% of the total calories.

  Timing of meals and snacks

  Children receiving twice daily injections of combined short and intermediate acting insulin regimens need three main meals and three snacks (mid morning, mid afternoon and prior to bed time).

  Eat meals and snacks at the same time each day. The timing of insulin injections may need to be adjusted according to the patients' own circumstances.

  Preschool aged children may have unpredictable eating habits and may require frequent small meals.

Exercise

- Regular exercise helps increase insulin sensitivity; maintains proper weight, blood pressure, blood glucose and blood fat levels.
- Exercise must be regular, i.e. daily. The same amount of exercise should ideally be done at the same time of the day.
- Some form of carbohydrate is necessary before and after intense exercise to reduce the risk of hypoglycaemia. Blood glucose monitoring may be necessary before and after intense exercise.

Blood glucose testing, record keeping and review of records

- Glucometers with compatible strips and bloodletting devices.
- Encourage children to perform their own finger-prick blood glucose testing.
- Finger prick should be performed at the side of the fingertips.
- Encourage the child to monitor his/her blood glucose prior to each main meal and at bedtime. A daily record of all testing performed should be recorded in a logbook. Review logbook frequently to ensure optimal adjustments in management.
- More frequent blood glucose testing is indicated if the child is unwell, partaking in unusual amounts of physical activity or feels hypoglycaemic.
- For a basal-bolus regimen, testing can be done up to 5 times a day and for other regimens, twice daily.

Glycaemic targets

- » Glycaemic targets for young children should not be as strict as for adults. Balance the ability of the family to avoid recurrent hypoglycaemia. A paediatrician should assist in setting practical goals. See table "Monitoring, control and adjustments".
- » Disabling hypoglycaemia is the presence of recurrent and unpredictable episodes of hypoglycaemia requiring third party assistance and leading to anxiety about repeated episodes and leads to a reduced quality of life
- » Ideally 80% of the pre-meal blood glucose values should fall within the target range during home monitoring, but targets may need to be altered based on the age of the child and the ability of the family.
- » Infants, toddlers, and preschoolers are unable to recognise or communicate signs and symptoms of low blood glucose. They also have unpredictable eating habits.
- » School-age children and some young adolescents have more predictable eating habits but may be lacking in judgement. They are able to recognise or communicate signs and symptoms of low blood glucose.
- » Most adolescents and young adults are able to recognize and treat low blood glucose reactions. They have predictable eating habits and are able to plan ahead.
- » Acceptable target range before meals:

	**Blood glucose levels**
> Infants and toddlers	6– 12 mmol/L
> School-age children and some young adolescents	4– 10 mmol/L
> Most adolescents and young adults	4– 8 mmol/L

- » Monitor HbA1c levels 3 monthly. The aim is to maintain HbA1C as close as possible to the recommended range, i.e. < 7.5%. Aim for a lower HbA1C in patients who are adherent with regard to home glucose monitoring.

Monitoring control and adjustments

**Level of control**	**Optimal**	**Suboptimal:** (need to take action)	**High risk** (refer patient to specialised diabetic clinic)
**Clinical assessment**			
Raised blood glucose	No symptoms	» polyuria,*<br>» polydypsia,* and<br>» enuresis.*	» blurred vision,<br>» poor weight gain,<br>» poor growth,<br>» delayed puberty,<br>» poor school attendance,<br>» skin or genital infections,<br>» signs of vascular compromise.
Low blood glucose	Few, mild and no severe hypoglycaemias.	Episodes of severe hypoglycaemia (unconsciousness and/or convulsions)**	
**Monitoring**<br>Biochemical assessment<br>Self monitoring finger prick glucose monitoring			
AM fasting (preprandial)	4–6	>8	>9
Postprandial	5–10	10–14	>14
Bed time	6.7–10	<6.7*** or 10–11	< 4.4*** or >11
Nocturnal	4.5–9	<4.2*** or >9	<4*** or >11
HbA_{1C}	<7.5	7.5–9.0	>9.0

*In situations with polyuria, polydypsia and enuresis, adjust the doses of the insulin upwards. Dose adjustments should usually not be greater than 10% of the daily dose at any one time.

** Identify and address the specific reasons for hypoglycaemia e.g. skipping meals or snacks.

In specific situations where the lifestyle cannot be modified or there are recurrent episodes of severe hypoglycaemia, consider referral to a tertiary centre.

*** Consider hypoglycaemia unawareness in situations where there are consistently low readings and the patient does not report symptoms.

- Hypoglycaemia unawareness is potentially dangerous. The (appropriate) insulin dose may need to be adjusted downwards if more than 30% of the readings (during a single week) are below the target values indicated.

Urine ketone testing

- Hyperglycaemia and substantial ketones (+++) indicates that DKA is present.
- Test urine for ketones in the following circumstances:
  - if vomiting occurs;
  - any time the blood glucose > 15 mmol/L, especially if the child is unwell and particularly if the blood glucose has been high for more than 24 hours;
  - if unusual drowsiness is present;
  - in the presence of high temperature, vomiting or diarrhoea, even when the glucose is < 15 mmol/L;
  - if abdominal pains occur;
  - if the breathing is deep and rapid or smells of acetone.

MEDICINE TREATMENT

Insulin therapy

Principles of insulin therapy:

- To provide sufficient insulin throughout the 24 hour period to cover basal requirements.
- To deliver higher boluses of insulin in an attempt to match the glycaemic effect of meals.
- The most suitable areas for insulin injection are:
  - the upper, outer area of the arms;
  - the front and side of the thigh;
  - the upper, outer surface of the buttocks; and
  - the abdomen, except the area close to the navel.
- Establish a pattern for injecting, i.e. horizontally or vertically. Vary the site of injection according to this pattern. When the area has been fully covered move to another area.
- Patients doing strenuous exercise should not inject into their legs.

Insulin injection technique

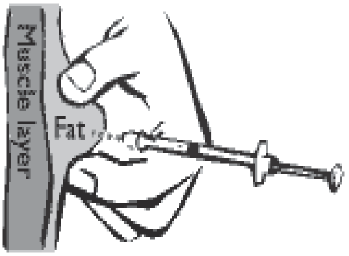

Pinching the skin to give an insulin injection. A small pinch with the finger and thumb is enough.

- Insulin injection by syringe is usually given into deep subcutaneous tissue through a two-finger pinch of skin at an angle of 45–90 degrees.
- The subcutaneous fat layer should be thicker than the needle length.
- There is significant risk of accidental intramuscular injections and hence more rapid absorption especially in lean individuals. This can be minimised by using a two-finger pinch technique, an injection angle of 45 degrees and 8 mm needles. Five or 6 mm needles may be appropriate in lean children or those using pens. Only withdraw the needle and release the skin fold on the count of ten.
- Disinfection of the skin is not necessary prior to insulin injections, however injections should be given through clean, healthy skin.
- Needles should not be used for more than 6 injections.
- Prefilled insulin syringes are recommended for children. Pen devices delivering less than 1 unit should be available for selected patients.
- Thoroughly mix all insulin suspensions before injection by rolling or inverting the vial ten times so that the cloudy suspension mixes thoroughly and uniformly.

Duration of action of standard insulins

Insulin	Onset of action	Peak action	Effective duration
Regular/short acting	30–60 minutes	2–3 hours	8–10 hours
Intermediate acting	2–4 hours	4–12 hours	12–20 hours

Choice of insulin regimen

- No insulin injection regimen satisfactorily mimics normal physiology. The choice of insulin regimen should be individualised and will depend on age, duration of diabetes, lifestyle (dietary patterns, exercise schedules, school, work commitments, etc), targets of metabolic control, and particularly, individual patient/family preferences.
- The choice of an insulin regimen is determined by the patient's circumstances. Depending on the patient's scope to undertake insulin therapy, a number of alternatives will allow insulin therapy to be tailored to their lifestyle. Discussion with parents should provide the basis for such important decisions.
- It is not possible to prescribe a single best regimen for preschool and primary school children. Individualise the choice of regimen according to family circumstances.
- Multiple daily injections provide for the best glycaemic control in young people with type 1 diabetes. If manageable, this should be the regimen of choice. Initially, a twice daily injection regimen may be more manageable.

Whichever insulin regimen is chosen should be supported by comprehensive education appropriate for the age, maturity and individual needs of the child and family.

Selecting an insulin regimen

<u>Total daily insulin dose</u>

This is individualised and varies according to age, puberty development, stress and individual variability. Usual range is 0.5–1 units/kg/day, but may be higher or less.

The aim is to select a regimen that allows the achievement of glycaemic control without disabling hypoglycaemia. This also requires a comprehensive support programme for the child and family enabling the implementation of an appropriate diet and other care strategies including home blood glucose monitoring and the ability to recognise and manage hypoglycaemic episodes. Where glycaemic control is not achieved despite an adequate support programme consider referral to a Tertiary centre**.**

Insulin regimens

Consult with a paediatric endocrinologist or paediatrician with experience in diabetes care. Repeated consultations are indicated when glycaemic control targets are not achieved.

The following regimen choices are listed in order of simplicity and flexibility.

Regimen 1: Two Injections daily

- A mixture (premixed combination) of short and intermediate acting insulins (before breakfast and the main evening meal).
- The total daily dose is divided so that $^{2}/_{3}$ is given in the morning and $^{1}/_{3}$ in the evening.
- The morning or evening dose is then again split between the intermediate-acting and the short-acting insulin in a 70:30 ratio which is pre-mixed
- This regimen is less flexible but easier to instruct.

**Regimen 1: Premixed 70/30**		
Breakfast	intermediate acting (70% of morning dose)<br>+<br>short acting insulin (30 % of morning dose)	$^{2}/_{3}$ of total daily dose in units
Supper	intermediate acting (70% of evening dose)<br>+<br>short acting insulin (30% of **evening** dose)	$^{1}/_{3}$ of total daily dose in units

OR

Regimen 2: Three injections daily

- A mixture of short and intermediate acting (premixed 70:30) insulin before breakfast; short acting insulin alone before an afternoon snack or main evening meal; intermediate acting insulin before bed; or variations of this regimen may be used at times.
- This requires that the caregiver is aware of three different insulin preparations and can differentiate between them.

**Regimen 2**		
Breakfast	short acting insulin (30% of morning dose)<br>+<br>intermediate acting (70% of morning dose)	$^{2}/_{3}$ of total daily dose
Supper	short acting insulin (1/3 of evening dose)	$^{1}/_{3}$ of total daily dose
At night (± 21h00)	intermediate acting (2/3 of evening dose)	

OR

Regimen 3: Basal-bolus regimen

- Short acting insulin 15–30 minutes before a meal or rapid acting insulin with main meals e.g. breakfast, lunch and main evening meal; intermediate acting insulin before bed.
- Normally, 30–40% of the total daily dose of insulin is given at bedtime as intermediate acting insulin. The remaining insulin is given prior to breakfast, lunch and evening meal in the form of short acting insulin.

**Regimen 3: Basal-bolus regimen**<br>Rapid acting insulin is indicated in the child (especially < 3 years of age) with erratic eating habits despite adequate education		
Breakfast	short acting insulin	20% of total daily dose (if able to count carbohydrates: give 1 unit per 15 g)
Lunch	short acting insulin	20% of total daily dose
Supper	short acting insulin	20% of total daily dose
At night (± 21h00)	intermediate acting (ideally this ought to be a basal insulin acting over 24 hours)	40% of total daily dose

Infants, toddlers, and preschoolers

Regimen 2.

OR

Regimen 3. (See meal planning)

Older children (> 5 or 6 years of age)

Regimen 2.

Teenagers

Regimen 3 (basal bolus).

Questions to be considered when choosing a regimen

What scope does the patient have for insulin therapy?

» Will the p atient be abl e to undert ake, financially and c ulturally, an advanced insulin regimen if necessary?
» Is a responsible person available to give insulin injections at all times of the day or only at certain times?
» How goal orientated is the patient/caregiver in terms of diabetes control?

What is the patient's eating pattern?

» What is the typical pattern of meals?
» What type of food do they typically eat at each meal, and how much?
» Is their eating pattern relatively constant, or does it vary?
» Can they or do they want to change their eating habits?

None of these regimens can be optimised without frequent assessment of blood glucose monitoring.

> Achieving a balance between food intake, insulin levels and energy expenditure is an essential pre-requisite for achieving glycaemic control.

Adjustment of insulin dosage for regimens 1 and 2
The insulin dose should not be changed after a single abnormal blood glucose reading.

Adjust the dose only once a pattern has been established. The dose which is to be adjusted depends on the time of abnormal glucose readings, as indicated in the table below:

<table>
<tr><th rowspan="2"></th><th colspan="4">Timing of the unsatisfactory blood glucose level</th></tr>
<tr><th>Before breakfast</th><th>Before Lunch</th><th>Before supper</th><th>At ± 21h00</th></tr>
<tr><td colspan="5">Regimens 1 and 2</td></tr>
<tr><td>Glucose too high
Which insulin dose to be increased</td><td rowspan="2">Supper or 21h00 dose: intermediate acting insulin</td><td rowspan="2">Breakfast dose: short acting insulin</td><td rowspan="2">Breakfast dose: intermediate acting insulin</td><td rowspan="2">Supper dose: short acting insulin</td></tr>
<tr><td>Glucose too low
Which insulin dose to be reduced</td></tr>
</table>

	**Timing of the unsatisfactory blood glucose level**			
	**Before breakfast**	**Before Lunch**	**Before supper**	**At ± 21h00**
**Regimen 3**				
**Glucose too high**<br>Which insulin dose to be increased<br>**Glucose too low**<br>Which Insulin dose to be reduced	21h00 dose: intermediate acting insulin	Breakfast dose: rapid (or short acting) insulin	Lunch dose: rapid (or short acting) insulin	Supper dose: rapid (or short acting) insulin

REFERRAL

- » Management of all children with diabetes should be supervised by a paediatrician with experience in managing diabetes in the young and under ideal circumstances should involve a multidisciplinary team, i.e. paediatrician, dietician, nurse educator, psychologist, ophthalmologist etc. at a district or regional hospital.
- » Complications.
- » Uncontrolled diabetics, such as children with unpredictable blood glucose control, nocturnal or frequent hypoglycaemic events or children who do not reach their therapeutic goals for consideration of analogue insulin.
- » Periodic screening of eyes by an ophthalmologist:
  - > prepubertal onset of diabetes: 5 years after onset or at age 11 years, or at puberty (whichever is earlier), and annually thereafter;
  - > pubertal onset of diabetes: 2 years after onset and annually thereafter.

7.5.1.1 GUIDELINES FOR MANAGEMENT OF DIABETICS ON SICK DAYS

DESCRIPTION

Illness associated with fever tends to raise blood glucose because of higher levels of stress hormones, gluconeogenesis and insulin resistance.

Illness associated with vomiting and/or diarrhoea may lower blood glucose, with the possibility of hypoglycaemia and the development of starvation ketones. Illness may result in ketone production.

DIAGNOSTIC CRITERIA

- » Unstable blood glucose measurements as a result of illness or stress or starvation.
- » Increased insulin requirements are induced by a catabolic state and stress.
- » Ketonuria may also indicate the following:
  - > ketonuria in the presence of hyperglycaemia is indicative of severe insulin deficiency and calls for urgent therapy to prevent progression into ketoacidosis;
  - > ketonuria in the presence of low blood glucose levels is indicative of a starvation state or is the result of a counter-regulatory response to hypoglycaemia.

GENERAL AND SUPPORTIVE MEASURES

- » Monitor glucose more frequently.
- » Test urine for ketones.
- » Ensure adequate intake of calories and fluids on sick days to prevent ketogenesis. If not enough calories are consumed, ketones will appear in the urine without the development of hyperglycaemia. In this circumstance encourage the patient to eat whatever he/she feels like.
- » Treat underlying intercurrent illness.
- » Special circumstances:
  - > Gastroenteritis:
    If hypoglycaemia occurs especially with gastroenteritis, and there is mild ketonuria, ensure that the child takes regular frequent amounts of carbohydrate using oral rehydration solution or intravenous fluids.
  - > Loss of appetite:
    Replace meals with easily digestible food and sugar-containing fluids.
  - > Vomiting:
    If the patient has difficulty eating or keeping food down and the blood glucose is < 10 mmol/L, encourage the patient to take sugar-containing liquids. Give small volumes. Some glucose will be absorbed. If there is no vomiting, increase the amount of liquid.

MEDICINE TREATMENT

Insulin therapy

Insulin must always be given each day. Do not skip an insulin injection because of sickness and/or vomiting. If vomiting occurs, IV fluids may be needed to avoid hypoglycaemia.

Generally the body will require more energy during illness. Insulin allows more glucose to enter into the cells, providing more energy to fight infection.

General guidelines when giving extra insulin:

» If the blood glucose is rising or if ketones in the urine, the patient must seek urgent medical attention.

Moderate urine ketones

» The extra dose of insulin is usually 10–20% of the total daily dose.
  This extra insulin is given as short (or rapid) acting insulin every three hours.
  If the blood glucose drops < 8.3 mmol/L, it may be necessary to sip regular juice or other sugar-containing drinks. This is done to raise the blood glucose before giving the next insulin injection.

Large amount of urine ketones

» Give 20% of the total daily insulin dose.
  Repeat as above if necessary.

Extra fluids

In addition to taking extra insulin, extra fluids, e.g. water and fruit juices are important to prevent acidosis. These fluids replace the fluids lost in the urine and prevent dehydration.

REFERRAL

» In a child with intercurrent illness **urgent** specialist medical or nursing advice must be obtained when:
  > patient is unable to carry out the advice regarding sick days;
  > the diagnosis is unclear;
  > vomiting is persistent, particularly in young children;
  > blood glucose continues to rise despite increased insulin;
  > hypoglycaemia is severe;
  > ketonuria is heavy or persistent;
  > the child is becoming exhausted, confused, hyperventilating, dehydrated or has severe abdominal pain.

7.5.2 DIABETES MELLITUS, INSULIN DEPENDENT: ACUTE COMPLICATIONS

E10

7.5.2.1 CEREBRAL OEDEMA IN DIABETIC COMA

G93.6

DESCRIPTION

A condition of brain swelling during the course of treatment of hyperglycaemic coma.

Cerebral oedema usually occurs 4–12 hours after the initiation of treatment and often follows an initial period of clinical and biochemical improvement.

Cerebral oedema causes significant neurological morbidity and has a mortality of approximately 80%.

The cause of cerebral oedema during treatment remains unclear. However, too rapid reduction in intravascular osmolality may aggravate the process. Therefore rehydration should occur more slowly in children with DKA than in other causes of dehydration.

DIAGNOSTIC CRITERIA

Clinical

» Signs and symptoms of cerebral oedema include:
  - headache,
  - confusion,
  - irritability,
  - reduced consciousness,
  - papilloedema,
  - hypoxaemia, and
  - specific neurological signs and raised intracranial pressure.

» The risk of cerebral oedema is increased if urea levels are increased or if the PCO_2 is persistently low, i.e. < 20 mmHg.

GENERAL AND SUPPORTIVE MEASURES

» Admit to ICU, if possible, or to a centre experienced with managing this condition.
» Restrict intravenous fluids to ⅔ maintenance and replace deficit over 72 hours rather than 48 hours pending ICU admission.
» Elevate head of bed.
» Exclude hypoglycaemia.
» Do not use bicarbonate.
» Exclude thrombosis, haemorrhage or infection.
» Do not delay treatment while waiting for a CT scan to confirm cerebral oedema.

MEDICINE TREATMENT

Within 10 minutes of diagnosis:

- Mannitol 20%, IV, 2.5 mL/kg, immediately over 15 minutes.
  - o If no initial response: repeat in 30 minutes to 2 hours.

7.5.2.2 HYPERGLYCAEMIC KETOACIDOSIS

E10.1

DESCRIPTION

Diabetic ketoacidosis (DKA) occurs with relative or absolute insulin deficiency, either caused by non-adherence to insulin regimens or by excessive secretion of counterregulatory hormones during stress, e.g. infection, trauma and surgery.

DIAGNOSTIC CRITERIA

» Heavy glycosuria.
» Hyperglycaemia, i.e. blood glucose usually > 15 mmol/L, ketonuria, and pH < 7.3.
» Bicarbonate < 15 mmol/L **and** patients who are clinically dehydrated.
» May/may not be vomiting.
» May/may not be drowsy.

Note:
In rare cases blood glucose is not elevated.
Children with mild dehydration and not clinically unwell usually tolerate oral rehydration and subcutaneous insulin.
See section 7.5.1.1: Guidelines for management of diabetics on sick days.

GENERAL AND SUPPORTIVE MEASURES

» Admit all children and adolescents to an ICU or ward experienced in the management of DKA in children and adolescents, if possible.
» Ensure patent airway.
» If the child is comatose, insert an artificial airway and a urinary catheter.
» If comatose or recurrent vomiting insert oro/nasogastric tube and apply free drainage.

MEDICINE TREATMENT

Seek specialist advice early in the management.

If hypoxaemic:

- Oxygen via facemask or airway.

The objectives of fluid and sodium replacement therapy in diabetic ketoacidosis are:

» Restoration of circulating volume.
» Replacement of sodium and extracellular fluid and intracellular fluid deficits of water.
» The restoration of glomerular filtration rate with enhanced clearance of glucose and ketones from the blood.
» To reduce the risk of cerebral oedema.

Fluids

<u>**a**: Fluids for resuscitation in shock:</u>

- Sodium chloride 0.9%, IV, 10–20 mL/kg over 10–30 minutes.
  - o Repeat if shock persists.

b: Fluid requirements after resuscitation

Calculation of fluid requirement subsequent phase of rehydration (see table below which has the calculations determined for different weights)
Fluid requirement = deficit + maintenance
Calculate deficit = estimated % dehydration x body weight (kg and equivalent in mL)
Calculate maintenance (mL):<br>≤1 year: 120 mL/kg/24 hours<br>All children older than 1 year – the sum of the following:<br>• first 10 kg body weight: 100 mL/kg/24 hours<br>• second 10 kg body weight: 50 mL/kg/24 hour<br>• additional weight > 20 kg body weight: 20 ml/kg/24 hours
Add deficit to 48 hour maintenance and replace this volume evenly over 48 hours with initially sodium chloride 0.9% When blood glucose falls to 12–15 mmol/L change the infusion to a dextrose-containing maintenance fluid, e.g. dextrose 5% in sodium chloride 0.45% .
Assess hydration status every 4–6 hourly

Examples of fluid volumes for **subsequent phase** of rehydration (i.e. maintenance + 5% of body weight/24 hours)

**Body weight kg**	**Maintenance mL/24 hour**	**Maintenance + 5% of body weight mL/24 hour**	**Maintenance + 5% of body weight mL/hour**
4	325	530	22
5	405	650	27
6	485	790	33
7	570	920	38
8	640	1040	43
9	710	1160	48
10	780	1280	53
11	840	1390	58
12	890	1490	62
13	940	1590	66
14	990	1690	70
15	1030	1780	74
16	1070	1870	78
17	1120	1970	82
18	1150	2050	85
19	1190	2140	89
20	1230	2230	93

Body weight kg	Maintenance mL/24 hour	Maintenance + 5% of body weight mL/24 hour	Maintenance + 5% of body weight mL/hour
22	1300	2400	100
24	1360	2560	107
26	1430	2730	114
28	1490	2890	120
30	1560	3060	128
32	1620	3220	134
34	1680	3360	140
36	1730	3460	144
38	1790	3580	149
40	1850	3700	154
45	1980	3960	165
50	2100	4200	175
55	2210	4420	184
60	2320	4640	193
65	2410	4820	201
70	2500	5000	208
75	2590	5180	216
80	2690	5380	224

Note:
Sodium chloride 0.9% is preferred for resuscitation and the initial phase of rehydration.
However, to prevent the occurrence of hyperchloraemic acidosis switch to sodium chloride 0.45%/dextrose 5% after blood glucose has fallen to 12 mmol/L or less.

Note:
One of the danger signals for cerebral oedema is a drop in the serum sodium

Bicarbonate
Bicarbonate use is associated with increased risk of cerebral oedema.

Caution
Bicarbonate should never be given without prior discussion with a specialist.

Potassium
Commence potassium replacement immediately unless anuria is present. If serum potassium is high wait for urination before replacement.
Early addition of potassium in the fluid regimen (15% kCl 20mL in 1L = 40 mmol/L.) is essential even if the serum concentration is normal as insulin will drive glucose and potassium into the cells.

DKA protocol: Two-bag system – Alternative fluid and electrolyte treatment
Under supervision of a specialist.
The two-bag system consists of 2 bags of identical electrolyte content but different dextrose concentrations, 0% and 10%, administered simultaneously into a single IV line. Variations in dextrose delivery are achieved through differential proportions of the 2 bags contributing to the total rate, which is determined by the patient's degree of dehydration.

- Sodium chloride 0.9%, IV, 10–20 mL/kg.
    - May be repeated if necessary.
    - Then switch to "two bag" system

**Bag 1 (dextrose 0%)**	**Bag 2 (dextrose 10%)**
• Sodium chloride 0.45%, 1 L<br>**PLUS**<br>• Potassium chloride, 20 mL	• Dextrose 10%, 1 L<br>**PLUS**<br>• Sodium chloride 5%, 90 mL<br>**PLUS**<br>• Potassium chloride, 20 mL

Run these two riders for easy titration of dextrose from dextrose 10% to dextrose 0%:

Fluid	Blood glucose >15	Blood glucose 10–15	Blood glucose <10
Bag 1	100%	50%	0%
Bag 2	0%	50%	100%

Insulin

- Insulin short-acting, 0.1 unit/kg/hour as a continuous IV infusion.
    - Add insulin, 50 units (0.5 mL) to 50 mL sodium chloride 0.9% in a syringe pump to get a solution of 1 unit/mL.
    - Attach this using a Y-connector to the IV fluids already being administered.
    - Do not add insulin directly to the fluid bags.
    - The solution should be administered at a rate of 0.1 mL/kg/hour. (0.1 unit/kg/hour)

  If the rate of blood glucose fall exceeds 5 mmol/ L/hour or the blood glucose falls to 14 mmol/L:
    - Add a dextrose-containing fluid.
    - Do not stop the insulin infusion while dextrose is being infused

  If the blood glucose falls below 4 mmol/L:
    - Give a bolus of 2 mL/kg of dextrose 10% and increase the concentration of dextrose in the infusion.

  Continue with IV insulin until:
    - base deficit is < 5 or bicarbonate is 15 mmol/L,
    - there is no ketonuria (or ketonemia if you can measure it),
    - blood glucose is 10 mmol/L.

Alternative to insulin infusion

Where there are no facilities for insulin infusion, e.g. no syringe pumps, staff constraints, etc.:

- Insulin short-acting, IV, 0.1 unit/kg, hourly.

Changing from intravenous to subcutaneous insulin

Continue with intravenous fluids until the child is drinking well and able to tolerate snacks.

When oral fluids are tolerated, reduce intravenous fluids.

Subcutaneous insulin can be started once the child is well hydrated and able to tolerate a normal diet.

The most convenient time to change to subcutaneous insulin is just before a mealtime. Administer the first dose of subcutaneous insulin 30 minutes before the meal and continue with the insulin infusion for 90 minutes after the subcutaneous injection to prevent rebound hyperglycaemia.

In newly diagnosed diabetics regimen 1 is chosen at a low range dose divided in the usual way:

o Prepubertal children: 0.7 units/kg.

o Pubertal children: 1 unit/kg.

In established diabetics, give usual insulin.

Give supplemental subcutaneous short acting insulin before meals if the blood glucose > 11 mmol/L:

Blood glucose mmol/L	Short-acting Insulin units/kg/dose
11–12	0.06
13–16	0.09
16	0.12

REFERRAL

» No improvement

» Deterioration of condition, i.e.:

  > pH <7.1,

  > hyperventilation,

  > shock,

  > depressed level of consciousness,

  > persistent vomiting,

  > age < 5 years.

» Rising blood glucose.

7.5.2.3 HYPOGLYCAEMIA IN DIABETICS
E16.0

DESCRIPTION
Autonomic symptoms (hunger, nausea, anxiety, pallor, palp itations, sweating, trembling) usually precede neuroglycopaenic symptoms (impaired thinking, change of mood, irritability, dizziness, headache, tiredness, confusion, and later convulsions and coma). A reversal in appearance of the symptoms is undesirable and is known as hypoglycaemia unawareness.

Causes of hypoglycaemia include:
- » A missed or delayed snack or meal.
- » Exercise without appropriate dietary preparation.
- » Alcohol.
- » Overdose of insulin.
- » Impaired food absorption e.g. gastro-enteritis.
- » Addison’s disease. Recurrent hypoglycaemia may necessitate investigation for this condition.

Nocturnal hypoglycaemia
Nightmares and headaches may be suggestive of nocturnal hypoglycaemia. Blood glucose concentrations fall to their lowest levels between 02h00 and 04h00.

DIAGNOSTIC CRITERIA
- » Blood glucose < 3. 5–4 mmol/L with symptoms in a k nown diabetic patient.
  Good glycaemic control is likely to be associated with occasional hypoglycaemic episodes.
- » Grading of severity:
  <u>Mild (Grade 1)</u>
  - > Child or adolescent is aware of, responds to and self-treats the hypoglycaemia.
  - > Children < 6 years of age can rarely be classified as grade 1 because they are unable to help themselves.

  <u>Moderate (Grade 2)</u>
  - > Child or adolescent cannot respond to hypoglycaemia and requires help from someone else, but oral treatment is successful.

  <u>Severe (Grade 3)</u>
  - > Child or adolescent is semiconscious or unconscious or in coma with/without convulsions and may require parenteral therapy with glucagon or intravenous glucose.

GENERAL AND SUPPORTIVE MEASURES

» Determine underlying cause.
» Patient education on diabetes and its complications.
» If patient is fully alert and conscious, give sugar-containing drink and/or snack (carbohydrate).
» Monitor blood glucose every 15 minutes until blood glucose is 6–8 mmol/L.

MEDICINE TREATMENT

Mild or moderate hypoglycaemia:
Immediate oral rapidly absorbed simple carbohydrate, e.g.:

- Glucose, oral, 5–15 g or 1-3 level teaspoons of sugar in a small amount of water.
    - o Wait 10–15 minutes.
    - o If no response, repeat above.
    - o As symptoms improve, the next meal or oral complex carbohydrate should be ingested, e.g. fruit, bread, cereal, milk, etc.

Severe hypoglycaemia
<u>Outside hospital</u>

- Glucagon, IM/SC, 0.1–0.2 mg/10 kg.
    - o If < 12 years of age: 0.5 mg.
    - o If > 12 years of age: 1.0 mg.

<u>In hospital</u>
If there is an unsatisfactory response or inability to take oral carbohydrate and signs of disorientation, stupor, convulsions, coma:

- Dextrose 10%, IV, 2–5 mL/kg.
    - o Dilute dextrose 50% solution before use to 10% strength.
    - o (1 mL/kg of dextrose 50% plus 4 mL/kg of water for injection, gives 10% dextrose solution).

If IV dextrose cannot be given:

- Glucagon, IM/SC, 10–30 mcg/kg.

Monitor blood glucose every 15 minutes until stable, then repeat 1–2 hourly.
Keep blood glucose between 6 and 8 mmol/L.

REFERRAL

» Recurrent episodes of hypoglycaemia.

7.5.2.4 NEPHROPATHY

N08.3

DIAGNOSTIC CRITERIA

» Persistent albuminuria:
  > 3 specimens over a 3–6 month period all show increased albumin:creatinine ratio on a spot urine:
    males: > 2.5 mg/mmol,
    females: > 3.5 mg/mmol.

» Screening for micro-albuminuria should start from:
  > prepubertal children: duration of 5 years.
  > pubertal children: duration of 2 years.

GENERAL AND SUPPORTIVE MEASURES

» Optimise diabetic control.
» Monitor blood pressure.

MEDICINE TREATMENT

If albumin:creatinine ratio is > 3.5 mg/mmol:

▪ ACE inhibitor, e.g.:

• Enalapril, oral, 0.1 mg/kg/dose as a single dose or two divided doses.
  o Maximum dose: 0.5 mg/kg or 40 mg/day.

  Note:
  Exclude non-diabetic nephropathy.

REFERRAL

» All patients with significant albuminuria.

7.5.3 DIABETES MELLITUS IN ADOLESCENTS

E10

See section 22.6: Diabetes in adolescence.

DESCRIPTION

Adolescence is that period between puberty and when the patient leaves school to join the workforce. The adolescent and the transition should be managed with special planning, i.e.:

» the admission policy of the hospital,
» observing the wishes of the adolescent,
» emotional and physical maturity considerations,
» presence of any co-existing medical, surgical or psychiatric disorder that may be more appropriately managed in the paediatric service.

GENERAL AND SUPPORTIVE MEASURES

Promote:

- » normal growth and pubertal development,
- » psychological development,
- » maintenance of glycaemic control and adherence,
- » normal lifestyle,
- » avoidance of risk taking behaviours (smoking, substance abuse),
- » sex education.

MEDICINE TREATMENT

Failure of current insulin regimens are attributed to the endocrine changes of puberty which results in poor glycaemic control.
Insulin resistance occurs during puberty, being maximal in late puberty.

Normal insulin requirements during puberty:

o 1.0–1.4 units/kg/day.

This may occasionally be higher, but as a general rule a higher requirement generally necessitates the search for other causes.
After puberty, the insulin requirements fall to prepubertal levels.
Failure to reduce insulin requirements in the late adolescent stages may result in excessive weight gain.

7.5.4 DIABETES MELLITUS, TYPE 2

E11

DESCRIPTION

Type 2 diabetes develops when insulin secretion cannot meet the increased demand posed by insulin resistance. Type 2 diabetes may be associated with hyperlipidemia, hypertension, acanthosis nigricans, ovarian hyperandrogenism and non-alcoholic fatty liver disease (features of insulin resistance).

DIAGNOSTIC CRITERIA

Clinical

- » Obesity.
- » Children 10 years and older with a strong family history of type 2 diabetes, usually in adolescents with BMI > 95% without auto-antibodies to islet cells and normal serum C-peptide levels.
- » Keto-acidosis is unusual in type 2 diabetes.
- » Symptoms of diabetes <u>plus</u> random plasma glucose > 11 mmol/L **or** a fasting glucose > than 7 mmol/L
- » Type 2 diabetics may have minimal symptoms or signs for months or even years before the diagnosis.

Investigations

To confirm diagnosis:

» Fasting plasma glucose ≥ 7.0 mmol/L confirms the diagnosis.

OR

» Oral glucose tolerance test.
  > Ingestion of 1 .75 g/kg (maximum 75 g) of glucose dissolved in water.
  > Plasma glucose > 11 mmol/L 2 hours post ingestion of oral glucose.

GENERAL AND SUPPORTIVE MEASURES

» Lifestyle modification:
  Manage patients who are not ill at diagnosis initially with advice on nutrition and exercise, but most will eventually require medicine therapy.
» Education on routine blood glucose monitoring. A daily record of all testing performed should be recorded in a logbook. Record prebreakfast fasting and 2-hour postprandial dinner levels which is sufficient in most cases.

MEDICINE TREATMENT

Refer for initiation of therapy.

7.6 HYPOGLYCAEMIA IN CHILDREN

E16.2

DESCRIPTION

Infants and small children have relatively limited glycogen stores with larger brain/body ratios than adults and are therefore at greater risk of hypoglycaemia during starvation.

The causes of hypoglycaemia (outside the neonatal period) include:

» hypopituitarism,
» adrenal insufficiency,
» growth hormone deficiency,
» hypothyroidism,
» hyperinsulinaemia,
» inborn errors of metabolism,
» malnutrition,
» sepsis,
» liver dysfunction,
» malaria,
» severe illness with poor intake,
» accelerated starvation (ketotic hypoglycaemia),
» medicine, e.g. insulin, alcohol, aspirin, beta-blockers, oral hypoglycaemic agents, quinine.

DIAGNOSTIC CRITERIA

Clinical

» Acute autonomic symptoms: hunger, nausea, anxiety, pallor, palpitations, sweating, trembling.
» Neuroglycopaenic symptoms: impaired thinking, change of moo d, irritability, dizziness, headache, tiredness, confusion, and l ater convulsions and coma.
» Patients are often asymptomatic especially younger children who may be completely asymptomatic or present only with a behaviour change.

Investigations

» Plasma glucose concentration < 2.6 mmol/L.
» Although hypoglycaemia is a clin ical emergency requiring prompt therapy, wherever possible, draw a blood sample for investigation prior to the administration of glucose. Collect 5 mL of blood in a plain tube at the earliest opportunity and send for separation and storage of plasma at –20°C. Such samples may provide clear biochemical evidence of the cause of the hypoglycaemic episode thus avoiding having to subject the child to further investigations.

MEDICINE TREATMENT

After collection of initial blood samples:

- Dextrose 10%, IV, 2–5 mL/kg.
  - o Dilute dextrose 50% solution before use to 10% strength.
    (1 mL/kg of dextrose 50% plus 4 mL/kg of water for injection, gives 10% dextrose solution).

If hypoglycaemia persists or the plasma glucose is difficult to maintain in the normal range, consider adrenal insufficiency:

ADD

- Hydrocortisone, IV, 2–3 mg/kg, immediately.

Stabilisation

- Sodium chloride 0.9%/dextrose 5%, IV, 20 mL/kg bolus as needed.

OR

- Dextrose 10%, IV, 2–3 mL/kg glucose as needed.

AND

- Hydrocortisone, IV, 2–3 mg/kg immediately.

If hypoglycaemia persists consider adrenal insufficiency.

Ongoing treatment

Intravenous fluid therapy as needed.
Start oral feeds as soon as possible.

REFERRAL

» All patients with confirmed hypoglycaemia not explained by intercurrent illness, drugs, persisting or recurrent hypoglycaemia.

7.7 GROWTH DISORDERS

R62

DESCRIPTION

Constitutional delay in growth is defined as bone age which is significantly delayed compared to chronological age. Familial short stature is bone age equivalent to chronological age.

Common features of both are:

» short for chronological age,
» normal rate of linear growth,
no decline in height percentile.
Pathological growth failure are defined by height is disproportionately short relative to the weight or abnormally low linear growth rate.

DIAGNOSTIC CRITERIA

» Measure and plot child's height and weight on growth charts.
Routine monitoring of height and w eight for growth assists in t he diagnosis of problems which would otherwise be missed or would come to light at a sta ge where the outc ome of treat ment may be less favourable.
» A child is regarded as short if his/her height for age z-score is below –2 for gender.
» To further evaluate short stature assess parental height. Target height:
  > for a boy = (father's height + (mother's height + 13 cm)) ÷ 2
  > for a girl = ((father's height – 13 cm) + mother's height) ÷ 2
» If the child's predicted final height is > 8.5 cm below the target height, monitor growth (height) over 6 months to 1 year.
» If the child's height for age z-score is below –3, refer immediately.
» Suspect endocrine causes when there height is disproportionately short relative to the weight.
» Growth failure occurs when the child's height deviates further from z-score of –2 over a period of 1 year.

GENERAL AND SUPPORTIVE MEASURES

» Identify and manage non-endocrine causes of stunted growth, e.g.:
  > intra-uterine growth retardation,
  > chronic disease,
  > psychosocial deprivation,
  > skeletal dysplasia and other dysmorphic syndromes.

REFERRAL

- » Height for age z-score below –3.
- » Height 8.5 cm or more below target height.
- » Growth failure (height deviates further from z-score of –2 over a period of 1 year).
- » Suspected endocrine causes.
- » Dysmorphic child with unidentified syndrome.
- » Untreated chronic disease.

7.8 HYPOCALCAEMIA IN CHILDREN

E83.5

DESCRIPTION

The main causes of hypocalcaemia in children are:

- » vitamin D deficiency,
- » calcium deficiency,
- » magnesium deficiency,
- » reduced parathyroid hormone production,
- » impaired renal function.

DIAGNOSTIC CRITERIA

Clinical

- » Signs and symptoms of tetany include:
  - > paraesthesia,
  - > cramps,
  - > carpopedal spasm,
  - > laryngospasm,
  - > positive Chvostek's sign,
  - > positive Trousseau's sign,
  - > weakness,
  - > lethargy,
  - > prolonged QT interval on the ECG.

Investigations

- » Blood level to establish cause:
  - > calcium,
  - > albumin,
  - > phosphate,
  - > magnesium,
  - > ALP.

MEDICINE TREATMENT

Acute hypocalcaemia

- Calcium gluconate 10%, IV, 1–2 mL/kg administered over 5–10 minutes, 6–8 hourly.
  - o Maximum dose: 10 mL.
  - o ECG monitoring is advised.

If hypomagnesaemic:
- Magnesium sulphate 50%, IV/IM, 0.2 mL/kg every 12–24 hours.

Chronic therapy
Long-term therapy depends on the cause.
Manage hypophosphataemia or hyperphosphatemia, depending on the cause of hypocalcaemia, before long-term calcium is initiated.

- Calcium, elemental, oral, 50 mg/kg/day until normal calcium level is achieved.
  - o Follow with 30 mg/kg/day.

If vitamin D deficient:
- Vitamin D, oral, 5 000 IU/day.

For hypoparathyroidism and pseudohypoparathyroidism:
- Calcitriol, oral, 0.01–0.04 mcg/kg/day.

OR
- Alfacalcidol, oral, 0.05 mcg/kg/day.
  - o If < 20 kg: 0.0 5 mcg/kg/day.
  - o If > 20kg: 1 mcg/day.

REFERRAL
» Chronic hypocalcaemia.

7.9 HYPERKALAEMIA
E87.5

See section 6.1.4: Acute kidney injury (Renal failure, acute).

7.10 HYPOKALAEMIA
E87.6

DESCRIPTION
Causes include:
» prolonged decreased intake and protein energy malnutrition;
» increased renal excretion: renal tubular acidosis, amphoteracin B and diuretics;
» increased extrarenal losses;
» transmembrane shifts: $ß_2$ stimulants, alkalosis; and
» mineralocorticoid excess.

DIAGNOSTIC CRITERIA

Clinical

» Cardiac arrhythmias, especially with digitalis.
» Neuromuscular dysfunction, e.g. muscle weakness.
» Renal: impairment of urine concentrating or diluting ability.

Investigations

» Serum potassium < 3.0 mmol/L.

MEDICINE TREATMENT

See section 2.2.4: Diarrhoea, acute.

Severe respiratory paralysis and or cardiac arrhythmias:

- Potassium chloride, IV, < 1 mEq/kg/hour.
    - o ECG monitoring.
    - o Potassium concentration should not be > 40 mmol/L/infusion.
    - o Never give potassium as an IV bolus.

Less critical situations to correct potassium deficit over 2–3 days:

- Potassium chloride, oral, 2–6 mEq/kg/day.
  Note: 1 g KCl = 13 mEq; 1 mL 15% KCl = 2 mmol; 1 mEq = 1 mmol.

7.11 HYPOPITUITARISM

E23.0

DESCRIPTION

Multiple or isol ated deficiencies of adre nocorticoid hormone (ACTH), luteinising hormone, thyroid stimulating hormone, and growth hormone manifesting as hypoglycaemia, abnormal body proportions and failure to grow and develop.

The deficiency may be due to:

» congenital abnormalities with/without midline structural abnormalities of the brain,
» central nervous system tumours,
» histiocytosis,
» complications of radiation therapy.

DIAGNOSTIC CRITERIA

Clinical

» Neonates with hypopituitarism may present with:
  > persistent hypoglycaemia,
  > cholestatic jaundice (related to low cortisol),
  > micropenis.
» Growth failure with immature body proportions.

Investigations

» Endocrine evaluation with pituitary function tests under specialist supervision.
» Confirm diagnosis in older children with stimulation tests.

MEDICINE TREATMENT

To correct hypoglycaemia:

- Hydrocortisone, IV, 2-3 mg/kg.

REFERRAL

» All patients after stabilisation of hypoglycaemia.

7.12 HYPOTHYROIDISM, NEONATAL

P72.2

DESCRIPTION

Congenital deficiency of thyroid hormone due to:

» aplasia/hypoplasia or ectopia of the thyroid gland,
» defects in thyroid hormone biosynthesis, or
» intrauterine exposure to antithyroid medicines.

Congenital hypothyroidism is one of the co mmon treatable causes of preventable mental retardation in children. Congenital hypothyroidism must be treated as early as possible to avoid intellectual impairment.
Symptoms and signs in neonates are unreliable.

DIAGNOSTIC CRITERIA

Clinical

» Prolonged unconjugated hyperbilirubinaemia.
» Feeding difficulties.
» Lethargy.
» Somnolence.
» Abdominal distension.
» Umbilical hernia.
» Subnormal temperature.
» Periorbital oedema.
» Delayed dentition.
» Broad hands.
» Hair coarse and scanty.
» Hoarse voice and goitre.
» Oedema of the extremities and genitals.
» Bradycardia.
» Anaemia.
» Apnoeic episodes.
» Coarse cry.
» Constipation.
» Wide open fontanelles.
» Enlarged tongue.
» Short and thick neck.
» Dry skin.
» Hypotonia.
» Delayed physical and mental development.

Investigations

» When suspected, perform TSH test.
  > If elevated perform a free T_4.

Delay in diagnosis and treatment is associated with irreversible neurodevelopmental damage**.**

GENERAL AND SUPPORTIVE MEASURES

» Growth and neurodevelopmental assessment.
» Regular follow up.

MEDICINE TREATMENT

Neonates and infants, started as soon as possible, ideally within the first three weeks after birth:

- Levothyroxine, oral, 10–15 mcg/kg as a single daily dose.
  - o Adjust dosage to blood levels of T_4 (in the upper half of the reference range) and normalise the TSH (between 0.5–2 mU/L), especially in the first 3 years of life. Check TSH only 6 weeks after adjusting the thyroxine dose.
  - o Continue treatment indefinitely.

REFERRAL

» All patients for confirmation of diagnosis and initiation of therapy.

7.13 HYPOTHYROIDISM IN OLDER CHILDREN AND ADOLESCENTS

E03.9

DESCRIPTION

Acquired hypothyroidism in childhood and adolescents may be due to:

» chronic lymphocytic thyroiditis,
» goitrogen induced,
» iodine deficiency,
» post surgery,
» radioactive iodine,
» infiltrations, or
» medicines, e.g. antiretrovirals.

DIAGNOSTIC CRITERIA

» Elevated TSH and low thyroxine levels.

MEDICINE TREATMENT

- Levothyroxine, oral, 100 mcg/m^2 once daily.

REFERRAL

» All cases for investigation and initiation of therapy.

7.14 HYPERTHYROIDISM, GRAVES DISEASE

E05.9/E05.0

DESCRIPTION

Hyperthyroidism is a pathological syndrome in which tissue is exposed to excessive amounts of circulating thyroid hormones.
The most common cause is Grave's disease, although thyroiditis may also present with thyrotoxicosis.

DIAGNOSTIC CRITERIA

Clinical

» Fatigue.
» Poor school performance.
» Warm moist hands.
» Thyromegaly.
» Tremor.
» Proptosis.
» Tachycardia.
» Nervousness or anxiety.
» Weight loss.
» Palpitations.
» Heat insensitivity.

Investigations

» Elevated thyroxine (T_4) and suppressed TSH.

MEDICINE TREATMENT

- Carbimazole, oral, 0.5 mg/kg once daily.

AND

To block sympathetic hyperactivity:

- Atenolol, oral, 1–2 mg/kg as a single daily dose.

For children less than 10 kg:

- Propranolol, oral, 0.2–0.5 mg/kg 6–12 hourly.
    - o Maximum dose: 1.5 mg/kg/dose 6–12 hourly.

REFERRAL

» All patients for confirmation of diagnosis, initiation and follow up of therapy

7.15 OBESITY

E66

DESCRIPTION

Most children with obesity do not have an underlying pathological cause and have so-called "simple obesity", i.e. both weight and height are increased.

In children with pathological obesity, the height is not usually increased when compared to parental height. Causes of pathological obesity include syndromes, hypothalamic damage, endocrine abnormalities, immobility, impaired skeletal growth or medicines.

There has been a dramatic increase in the prevalence of childhood overweight and its resultant comorbidities.

DIAGNOSTIC CRITERIA

Clinical

» Measurement of weight alone is inadequate given the influence of height on weight.
» Assess severity using body mass index (BMI):

$$\text{body mass index} = \frac{\text{weight (kg)}}{[\text{height (m)}]^2}$$

» The BMI varies with age. Use sex-specific BMI charts for accurate identification of obesity.
» In general obesity is likely if BMI:
  - > 19 kg/m^2 at age 5 years,
  - > 20 kg/m^2 at age 10 years, and
  - > 25 kg/m^2 at age 18 years.

Investigations

» Fasting glucose and lipid profile.
» ALT.

GENERAL AND SUPPORTIVE MEASURES

» Weight control by:
  > education about the nature of obesity and its longer term consequences;
  > healthy eating, e.g. regular meal times, avoidance of excessive "snacking", fried foods, added fats and sugars and high energy drinks while encouraging foods with high fibre content, with modest calorie restriction;
  > increasing physical activity;
  > reduce sedentary time, e.g. TV watching, computer games, videogames or time on the telephone;
  > psychological support.
» Weight loss down to an "ideal body weight for height" is unrealistic. Prevention of further weight gain may produce significant longer-term benefits. If the patient is over 7 years, or if complications are present, aim for a weight loss of 0.5 kg/month.

MEDICINE TREATMENT

Manage hyperlipidaemia
See section 4.10: Dyslipidaemia.

REFERRAL

» All cases of pathological obesity.
» Severe/progressive obesity < 2 years.
» Serious co-morbidity requiring weight loss.

7.16 DISORDERS OF PUBERTY

E30

DESCRIPTION

Abnormally early or abnormally late development of signs of puberty including the development of breasts (in girls) or enlargement of external genitalia (boys) and sexual hair growth.
Often associated abnormality of growth velocity.

DIAGNOSTIC CRITERIA

» Puberty begins after 9 years and usually not later than 14 years in males.
» Puberty begins after 8 years and usually not later than 13.5 years in females.
» Precocity or delay of puberty occurring outside these ages need investigation.

Investigations

» Puberty staging.
» Radiological bone age.
» Endocrine investigation.

GENERAL AND SUPPORTIVE MEASURES

» Psychological support.
» Treat the cause, e.g. tumours.

REFERRAL

» All.

CHAPTER 8

INFECTIVE/INFECTIOUS DISEASES

8.1 HELMINTHIASIS, INTESTINAL

B82.0

DESCRIPTION

Infestation of the intestine with adult worms. The following species are commonly encountered:

» *Ascaris lumbricoides* (round worm).
» *Enterobius vermicularis* (pin worm).
» *Trichuris trichiura* (whipworm).
» *Ancylostoma duodenale* and *Necator americanus* (hookworm).
» *Taenia saginatum* and T. *solium* (beef and pork tapeworms).

DIAGNOSTIC CRITERIA

» Most infestations are asymptomatic and become apparent with the passage of a worm rectally or orally.
» Signs and symptoms include:
  > vague abdominal pains,
  > diarrhoea,
  > rectal prolapse,
  > protein losing enteropathy.
  > perianal itch,
  > vaginitis,
  > iron deficiency anaemia, and
» Surgical complications of m echanical effects occur in the bowel, pancreatic duct or biliary tree m igration of w orm larvae may cause cutaneous, pulmonary or cerebral symptoms. See se ction 13.8: Neurocysticercosis.
» Migration of worm larvae may cause cutaneous, pulmonary or cerebral symptoms. See section 13.8: Neurocysticercosis
» Definitive diagnosis is based on recognition of the worm or identification of worm eggs or proglottids in stool.

GENERAL AND SUPPORTIVE MEASURES

Prevent infestation by:

» Hand washing.
» Careful preparation of foods by adequate washing and cooking.
» Wearing shoes (hookworm).
» Improved sanitation will protect the environment from contamination.

MEDICINE TREATMENT

All helminths excluding *Taenia* and *Enterobius*:

Children 1–2 years of age:

- Mebendazole, oral, 100 mg 12 hourly for three days.

Children > 2 years:

- Mebendazole, oral, 500 mg as a single dose immediately.

Enterobius

- Mebendazole, oral, 100 mg immediately as a single dose.
  - o Repeat after 2 weeks.

Taenia

- Albendazole, oral for three days.
  - o If 1–2 years of age: 200 mg.
  - o If > 2 years of age: 400 mg.

REFERRAL

» Abdominal complications requiring specialist assessment.

8.2 AMOEBIASIS (ENTAMOEBA HISTOLYTICA)

A06.9

DESCRIPTION

Amoebic colitis is caused by the parasite *Entamoeba histolytica*. It can cause localised intestinal disease or disseminated disease. Amoebiasis is n ow relatively uncommon in South Africa, but immunodeficiency is a risk factor.

DIAGNOSTIC CRITERIA

Clinical

» Diarrhoea with mucus, blood and pus (dysentery).

» Liver abscesses:
  - > presents with point tenderness over the liver area,
  - > pleuritic type pain,
  - > fever (often fever of unknown origin).

Laboratory diagnosis of colitis:

» Trophozoites or cysts in fresh stool.

» Trophozoites in rectal smear (danger of perforation if biopsy is done).

» Serological tests (ELISA and agar gel diffusion).

GENERAL AND SUPPORTIVE MEASURES

» Hand washing.

» Careful preparation of foods by adequate washing and cooking.

» Aspirate liver abscess if not responding to treatment in 5 days or if rupture is imminent.

MEDICINE TREATMENT

- Metronidazole, oral, 15 mg/kg/dose 8 hourly for 7 days.
  - o 10 days in severe disease.

8.3 CUTANEOUS LARVA MIGRANS/*ANCYLOSTOMA BRAZILIENSE* (DOG HOOKWORM)

B76.9/B76.0

DESCRIPTION

Infestation of the skin by dog hookworm larvae. Maturation of the larvae cannot occur. The infection is self-limiting.

DIAGNOSTIC CRITERIA

» Presents as an itchy “serpiginous” skin lesion.

GENERAL AND SUPPORTIVE MEASURES

» Regular deworming of dogs.
» Wearing shoes to protect against infection.

MEDICINE TREATMENT

- Albendazole, oral for three days.
  - o If 1–2 years of age: 200 mg.
  - o If > 2 years of age: 400 mg.

8.4 HYDATID DISEASE

B67

DESCRIPTION

The development of hy datid (*Echinococcus granulosus)* cysts follows ingestion of worm ova that are usually passed in the stools of dogs in sheep farming areas. Cysts may occur in any organ, but are most commonly found in the liver and lungs.

DIAGNOSTIC CRITERIA

» Typical radiological features.
» Diagnostic aspiration of an organ cyst should never be attempted.

GENERAL AND SUPPORTIVE MEASURES

» Prevent infestation by:
  > hand washing,
  > adequate food preparation,
  > surgical removal of cysts may be indicated.

MEDICINE TREATMENT

- Albendazole, oral, 7.5 mg/kg/dose 12 hourly for three 28 day cycles with a 14-day interval between each cycle.

REFERRAL

» All for consideration of PAIR (Percutaneous Puncture, Aspiration, Injection (of a scolecidal agent), re-aspiration) which should be carried out under expert supervision.

8.5 SCHISTOSOMIASIS (BILHARZIA)

B65.0/B65.1

DESCRIPTION

Disease manifestations caused by infestation by species of the genus Schistosoma.

Infestations with *S. haematobium* and *S. mansoni* are endemic in certain areas of South Africa.

Nematodes reside in venous plexus draining bladder wall (haematobium) or intestine (mansoni).

Complications include:

» haematuria,
» dysuria,
» cystitis,
» calcifications in the bladder wall,
» obstructive uropathy,
» bladder stones,
» intestinal perforation,
» strictures,
» hepatosplenomegaly,
» portal hypertension,
» cirrhosis,
» ascites,
» pulmonary hypertension,
» bladder cancer,
» fistulas,
» spinal cord granulomas with pressure effects.

DIAGNOSTIC CRITERIA

Clinical

» Transient pruritic papular rash (swimmers itch) after exposure to cercariae in the water.
» A few weeks after exposure:
  > fever,
  > chills,
  > headache,
  > urticaria,
  > cough, and
  > wheezing,
  > hepatosplenomegaly,
  > arthralgia,
  > lymphadenopathy,
  > eosinophilia.
» Haematuria and dysuria.
» Abdominal pain and diarrhoea often after ingestion of food.

Investigations

- » Positive serological tests for schistosomiasis.
- » Viable eggs in urine, stools or rectal biopsy specimens.

GENERAL AND SUPPORTIVE MEASURES

- » Educate patient/caregiver on preventative measures.
- » Symptomatic and supportive treatment.
- » Avoid exposure to water contaminated by schistosoma.
- » Surgical intervention to correct or prevent complications.

MEDICINE TREATMENT

- Praziquantel, oral, 40 mg/kg/24 hours as a single dose or in 2 divided doses on the same day.

REFERRAL

- » Schistosomiasis with suspected complications following adequate therapy.

8.6 CANDIDIASIS, SYSTEMIC AND OTHER

B37

DESCRIPTION

Superficial and/or disseminated (systemic) fungal infection caused by *C. albicans, C. Tropicalis* and other candida species.

Risk factors include:

- » Prolonged, broad-spectrum antibiotic therapy.
- » Compromised immune system, including patients infected with HIV or on cancer chemotherapy, and the premature baby.
- » Steroid therapy.
- » Diabetes mellitus.
- » IV hyperalimentation – may directly contaminate solution or as an associated risk factor.
- » Instrumentation, and central or peripheral vascular catheters.

DIAGNOSTIC CRITERIA

Clinical

- » Oral candidiasis (thrush):
  - > White plaque adheres to inner cheeks, lips, palate and tongue.
  - > Stomatitis with red mucosa and ulcers may also be present.
  - > In immunocompromised patients, the lesions may extend into the oesophagus.
- » Oesophageal candidiasis:
  - > Presents as difficulty swallowing, drooling or retrosternal pain (irritability in small children).

- » Skin lesions in the newborn:
  - > A red, maculopapular or pustular rash is seen in infants born to women with candida amnionitis.
- » Cutaneous dissemination:
  - > May be represented by scattered, red papules or nodules.
  - > Superficial infections of any moist area, such as axillae or neck folds, are common and may present as an erythematous, intertriginous rash with satellite lesions.
- » Vulvovaginitis:
  - > A thick cheesy vaginal discharge with intense pruritus, white plaques on the glans of the penis.
  - > Common in diabetics and patients on broad-spectrum antibiotics.
  - > In recurrent vulvovaginitis, exclude diabetes, foreign body or sexual abuse.
- » Systemic or disseminated candidiasis:
  - > Mimics bacterial sepsis but fails to respond to antibiotics.
  - > Thrombocytopaenia is common.
  - > Ophthalmitis with "cotton wool" retinal exudates may also occur.
  - > Is usually nosocomial.

Investigations

- » For oesophageal candidiasis:
  - > Oesophagoscopy or barium swallow. It is reasonable to initiate treatment on clinical grounds.
- » Systemic candidiasis:
  - > Urine and blood cultures are essential.
  - > Budding yeasts and pseudohyphae are seen on microscopy of biopsy specimens, fluid or scrapings of lesions.

GENERAL AND SUPPORTIVE MEASURES

- » Encourage cup feeding of formula-fed infants, as bottles are difficult to clean and predispose to candida infection.
- » Eradicate or minimise risk factors.
- » Avoid use of pacifiers (dummies), teats and bottles but if used, these should be sterilised.
- » Remove all invasive devices, drain abscesses and debride infected tissue.

MEDICINE TREATMENT

Oral candidiasis

- Nystatin suspension 100 000 IU/mL, oral, 1 mL 4 hourly.
  - o Keep in contact with affected areas for as long as possible.
  - o Suspect immunodeficiency if poor response to treatment.

If no response:
- ▪ Imidazole oral gel, e.g.:
- Miconazole gel 2%, oral, apply 8 hourly.

Oesophageal candidiasis
- Fluconazole, IV/oral, 12 mg/kg immediately as a single dose.
  - o Follow with 6 mg/kg/day for 3 weeks.

Vulvovaginitis
- Fluconazole, oral, 12 mg/kg as a single dose.
  - o Maximum dose: 150 mg.

OR
- ▪ Imidazole topical/vaginal, e.g.
- Clotrimazole **OR** miconazole, applied locally at night for 7–14 days.
  - o Do not use applicator in girls who are not sexually active.

Systemic candidiasis
- Amphotericin B, IV infusion, 0.5–1 mg/kg/dose once daily over 4 hours for at least 3 weeks after clinical improvement.
  - o Maximum dose if CNS involvement 1 mg/kg/day.
  - o Maximum cumulative dose: 30–35 mg/kg over 4–8 weeks.
  - o Adjust dosing interval in patients with renal impairment.
  - o Protect from light during infusion.
  - o Check serum potassium and magnesium at least 3 times a week.
  - o Do **not** use bacterial filter with amphotericin B.

Prehydration before administering amphotericin to prevent renal impairment:
- Sodium chloride 0.9%, IV, 20 mL/kg **plus** potassium chloride, 20 mmol/L infused over 2–4 hours.

REFERRAL
» Candidiasis not responding to adequate therapy.
» Patients with renal and hepatic failure.
» Confirmed azole resistance.

8.7 CYTOMEGALOVIRUS (CMV) INFECTION
B25.9

DESCRIPTION
Usually asymptomatic infections but may cause mononucleosis-like syndrome in children and adolescents.
Congenital infections vary from asymptomatic through isolated neural deafness, to severe disease including microcephaly.
Severe disease can occur in immunocompromised children especially HIV-infected children, e.g. pneumonia, encephalitis, retinitis and gastrointestinal infections.

DIAGNOSTIC CRITERIA

Diagnosis can be difficult as presence of antibodies to CMV does not imply active infection or causality.

» Quantitative CMV PCR (viral load).
» Intranuclear inclusion bodies may be seen in biopsy material.

MEDICINE TREATMENT

In severe disease:

- Ganciclovir, IV. (Specialist initiated)
  - o Initial dose: 5 mg/kg administered over 1 hour, 12 hourly for 21 days.
  - o Follow with: 5 mg/kg daily for 21 days.

REFERRAL

» All cases of severe organ-related disease or disseminated disease.
» For consideration of ganciclovir in patients also infected with PCP.

8.8 DIPHTHERIA

A36.9

***** Notifiable condition

TELEPHONE HOTLINE	
National Institute of Communicable Diseases	011 386 6337 or 011 386 6000
After hours	082 883 9920

DESCRIPTION

Diphtheria is an acute, communicable infection of the upper respiratory tract, caused by *Corynebacterium diphtheriae*. Disease is unlikely if the pat ient shows documented evidence of complete immunisation.

Cutaneous diphtheria can also occur.

Incubation period is between 2 and 7 days.

Complications include:

» In the first 2 weeks of the disease:
  > Cervical lymphadenopathy with peri-adenitis and with swelling of the neck (bull neck).
  > Upper airway obstruction by membranes.
  > Myocarditis.
» Usually after 3 weeks:
  > Neuritis resulting in paresis/paralysis of the soft palate and bulbar, eye, respiratory and limb muscles.

DIAGNOSTIC CRITERIA

Clinical

» Presents with upper airway obstruction and white to grey adherent pseudomembranes, myocarditis or peripheral neuritis.

Investigations

» Irregular staining Gram positive pleomorphic bacillus on throat swab.
» Culture of membrane or throat swab.

GENERAL AND SUPPORTIVE MEASURES

» Isolate patient in high or intensive care unit until 3 successive nose and throat cultures at 24-hour intervals are negative.
» Usually non-communicable within 4 days of antibiotics.
» Nutritional support.
» If respiratory failure develops, provide ventilatory support.
» Tracheostomy if life-threatening upper airway obstruction.
» Bed rest for 14 days.

MEDICINE TREATMENT

Note:

Do **not** withhold treatment pending culture results.

Antibiotic therapy

- Benzylpenicillin (Penicillin G), IV, 50 000 units/kg/dose, 6 hourly for 10 days.

<u>Penicillin allergy</u>

See section 23.4.1: Allergies to penicillins.

<u>Close contacts (household and regular visitors):</u>

Regardless of immunisation status, isolate contact and swab throat for culture. Keep under surveillance for 7 days.

All close contacts:

- Erythromycin, oral, 12.5 mg/kg/dose 6 hourly for 7 days.
  - Maximum dose: 1 000 mg/day.

OR

If contacts cannot be kept under surveillance:

- Benzathine benzylpenicillin (depot formulation), IM, single dose
  - If < 30 kg 600 000 units
  - If > 30 kg 1.2 million units

If 1st culture was positive, follow up throat culture after 2 weeks and treat again:

- Erythromycin, oral, 12.5 mg/kg/dose 6 hourly for 10 days.
  - Maximum dose: 1 000 mg/day.

REFERRAL

» All.

8.9 MALARIA

B54

* Notifiable disease.

DESCRIPTION

Malaria is transmitted by the bite of an infected female *Anopheles* mosquito. The incubation period varies with the species of the parasite, *Plasmodium falciparum* being shortest, usually 7–21 days, and *P. malariae* the longest. The incubation period may be prolonged by use of malaria prophylaxis or certain antibiotics.

Infection is caused by four species of protozoa of the genus Plasmodium, i.e. *P. falciparum, P. vivax, P. malariae and P. ovale.*
P. falciparum is the most common and causes the most severe disease.

The confirmation of the diagnosis and treatment of malaria is an emergency. Complications develop rapidly. Malaria can be missed outside transmission areas.

DIAGNOSTIC CRITERIA

Clinical

» A child living in, or with recent **travel history** to a malaria transmission area.
» Fever, which may be intermittent.
» Flu-like symptoms including sweating or rigors, i.e. cold shaking feeling.
» Body pains and headache.
» Occasionally diarrhoea, loss of appetite, nausea and vomiting, tachypnoea and cough.
» A young child may present with fever, poor feeding, lethargy, vomiting, diarrhoea or cough.
» Clinical features are non-specific and overlap with many other infections.

Investigations

» Testing is urgent. Obtain the result immediately.
  > Rapid diagnostic test.
  In areas where malaria transmission occurs, rapid tests should always be available for malaria screening but cannot be used for monitoring response to treatment as they may remain positive for over 4 weeks.

» Malaria parasites in blood smear – thick and thin smears.
  > One negative malaria test does not exclude the diagnosis.
  > Repeat smears if initially negative, and malaria suspected.
  > If severe malaria suspected, commence therapy and repeat smears after 6–12 hours.
  > Repeat smears after 48 hours and if no improvement in degree of parasitaemia, consider alternative therapy.

If severe malaria is suspected and diagnosis cannot be confirmed immediately, treat while awaiting laboratory results.

8.9.1 *P. FALCIPARUM* MALARIA, NON-SEVERE, UNCOMPLICATED

B50.9

DESCRIPTION

A child with uncomplicated malaria is alert, can tolerate oral medication, can sit, stand or walk unaided as appropriate for age and has no clinical or laboratory evidence of severe malaria.

Ideally treatment should be started in hospital. Initial doses should be directly observed. Observe for 1 hour to ensure dose is not vomited.

MEDICINE TREATMENT

Treat according to the National Malaria Guidelines.

Option 1:

Only for clearly uncomplicated, low risk malaria cases (> 5 kg):

- Artemether/lumefantrine 20/120 mg, oral, with fat-containing food/milk to ensure adequate absorption.
  - o Give first dose immediately.
  - o Follow with second dose 8 hours later.
  - o Then 12 hourly for another 2 days (total number of doses in 3 days = 6).

Weight	Dose	Total tablets per course
5– ≤15 kg	1 tablet	6
15–≤25 kg	2 tablets	12
25– ≤35kg	3 tablets	18
over 35 kg	4 tablets	24

OR

Option 2:

Manage children < 5 kg with uncomplicated malaria with quinine plus clindamycin:

- Quinine, oral, 10 mg/kg/dose 8 hourly for 7–10 days.

2–3 days after initiating treatment with quinine:

- Clindamycin, oral, 10 mg/kg/dose 12 hourly for 7 days.

Children who are vomiting but who have no other indications of severe malaria:

- Quinine, IV, 10 mg/kg/dose 8 hourly administered over 4–6 hours.
  - o ECG and heart rate monitoring.
  - o Monitor blood glucose levels regularly.
  - o Switch to oral medication, once able to do so.

8.9.2 *P. FALCIPARUM* MALARIA, SEVERE, COMPLICATED (OR IF REPEATED VOMITING)

B50.0/B50.8

DIAGNOSTIC CRITERIA

Clinical

» Unable to drink or breastfeed.
» Vomits everything.
» Renal failure.
» Cerebral malaria: manifests with convulsions, which may be subtle, and/or any change in mental state, ranging from irritability, lethargy to coma, stiff neck or bulging fontanelle.
» Respiratory distress and metabolic acidosis similar to pneumonia.
» Anaemia: can be severe and lead to cardiac failure and a depressed mental state.
» Shock: cold moist skin, low blood pressure and collapse.
» Hypoglycaemia: can present with convulsions and a depressed mental state.
» Jaundice, bleeding, acute renal failure and ARDS are less common in children than adults.

Investigations

» Hyperparasitaemia: > 5% of RBCs infected indicates severe malaria but a lower parasite density does not exclude severe malaria.
» Low Hb (< 6 g/dL).
» Test glucose immediately with a fingerprick test. Low blood glucose (< 2.2 mmol/L).
» Acidosis: serum lactate (venous) > 5 mmol/L or bicarbonate < 15 mmol/L.
» Severe thrombocytopaenia: < 50 x 10^9/L.
» In severe cases, repeat smear after 72 hours and after the completion of the course of treatment.

GENERAL AND SUPPORTIVE MEASURES

» Check airway, breathing, circulation (ABC).
» Admit to high care or intensive care unit.
» Review the child at least twice daily, including holidays.
» Avoid overhydration.

» Control convulsions.
» Ventilatory support, if necessary.
» Agitation and respiratory distress can be as a result of severe metabolic acidosis. Treat shock and acidosis. See section 1.1.7: Shock.
» Nutritional support.

MEDICINE TREATMENT
Urgent

- Quinine, IV infusion, diluted in 5–10 mL/kg dextrose 5% or sodium chloride 0.9%.
  - o Loading dose: 20 mg/kg over 4 hours (loading dose).
  - o Follow with 10 mg/kg over 4–6 hours at 8 hourly intervals until able to take oral therapy.
  - o ECG monitoring, if available.
  - o Monitor blood glucose levels.

2–3 days after initiating treatment with quinine and able to swallow, switch to any of the 2 regimens:
Children > 5 kg:

- Artemether/lumefantrine 20/120 mg, oral, with fat-containing food/milk to ensure adequate absorption.
  - o Give first dose immediately.
  - o Follow with second dose 8 hours later.
  - o Then 12 hourly for another 2 days (total number of doses in 3 days = 6).

Weight	Dose	Total tablets per course
5– ≤15 kg	1 tablet	6
15–≤25 kg	2 tablets	12
25– ≤35kg	3 tablets	18
over 35 kg	4 tablets	24

OR
Children < 5 kg

- Quinine, oral, 10 mg/kg/dose 8 hourly to complete 7–10 day course.

PLUS

- Clindamycin, oral, 10 mg/kg/dose 12 hourly for 7 days.

For concurrent bacterial sepsis:

- Ceftriaxone, IV, 100 mg/kg as a single daily dose once daily for 10 days.
  - o Maximum dose: 4 000 mg/24 hours.

For fever:

- Paracetamol, oral, 15 mg/kg/dose, 6 hourly as required.

For hypoglycaemia:
- Dextrose 10%, IV

If Hb < 7 g/dL:
- Packed red cells, IV, 10 mL/kg over 3 hours.

Note:
Fluid loss is often underestimated in a febrile, vomiting, sweating child.

8.9.3 P. OVALE, *P VIVAX* AND P*. MALARIAE*
B53.0/B51.9/B52.9

- Chloroquine, oral, 10 mg base/kg as a single dose,
  - Follow with 5 mg base/kg given 6, 24 and 48 hours after the first dose.

PLUS
To eradicate the organism:
- Primaquine, oral, 0.25 mg base /kg/day for 14 days. (obtained using section 21 approval)
  - Continue chloroquine once weekly until primaquine is obtained.

8.9.4 MALARIA PROPHYLAXIS – SELF PROVIDED CARE

In the high-risk malaria areas from September to May in South Africa, malaria prophylaxis should be used, together with preventive measures against mosquito bites. State facilities do not provide prophylactic therapy. It is recommended that persons intending to travel to high-risk areas take the relevant prophylactic therapy.

Preventative measures against mosquito bites include:
» Use of treated mosquito nets, screens, coils or pads.
» Application of insect repellent to exposed skin and clothing.
» Wearing long sleeves, long trousers and socks if outside between dusk and dawn, as mosquitoes are most active at this time.
» Visiting endemic areas only during the dry season.

!CAUTION!
Pregnant women and children under 5 years should avoid visiting malaria-endemic areas, as they are more prone to the serious complications of malaria

For chemoprophylaxis refer to National Malaria Guidelines.

REFERRAL

- **Urgent**: Severe or complicated malaria.
- High risk children under 2 years, splenectomised patients.
- Malaria not responding clinically to adequate treatment within 48–72 hours (possible resistance).

8.10 MEASLES

B05

* Notifiable condition

DESCRIPTION

The following case definition is an epidemiological and not a diagnostic tool:

- Fever and maculopapular rash with any one of the following:
  - cough,
  - coryza/runny nose,
  - conjunctivitis.

Suspect measles in any child fulfilling the case definition.

An acute, highly contagious, viral, childhood exanthem.
Incubation period: 8–14 days from exposure to 1st symptoms and 14 days between appearance of rash in source and contact.

Complications include:

- pneumonia,
- laryngotracheobronchitis (croup),
- encephalitis,
- stomatitis, and
- feeding difficulties,
- severe diarrhoea,
- otitis media,
- corneal ulceration.

Subacute sclerosing panencephalitis is a rare long-term complication.

DIAGNOSTIC CRITERIA

Clinical

- Prodromal (catarrhal) phase:
  - duration 3–5 days,
  - fever,
  - runny nose (coryza),
  - cough,
  - conjunctivitis.
- Koplik's spots, followed 3–5 days later with maculopapular rash.
- The rash begins to fade after 3 days in the order of its appearance leaving temporary darker staining.
- If fever is still present after the third day of the rash, a complication should be suspected.

Investigations

- Serum measles IgM antibodies for confirmation of diagnosis.

GENERAL AND SUPPORTIVE MEASURES

» Notify provincial EPI manager prior to confirmation.
» Only admit high risk patients:
  > children less than 6 months old,
  > immune compromised/suppressed children,
  > children with severe malnutrition,
  > children with complications.
» Minimal exposure to strong light, if patient is photophobic.
» Isolate the patient in a separate room, if possible away from other children.
» All entering the room to wear mask, gloves and gown.
» Patient is infectious for 4 days after onset of rash, longer if HIV-infected.
» Screen outpatient waiting areas for children with measles.
» If pneumonia with hypoxia, give humidified oxygen by means of nasal cannula.

MEDICINE TREATMENT

All patients

- Vitamin A, oral, as a single daily dose for 2 days.
  - o If < 6 months of age: 50 000 units.
  - o If 6–12 months of age: 100 000 units.
  - o If > 1 year of age: 200 000 units.

For fever

- Paracetamol, oral, 10–15 mg/kg/dose, 6 hourly as required until fever subsides.

Pneumonia

Antibiotics, empirical

To cover *S. pneumoniae* and Gram-negative infection.
Total duration of therapy: 5–7 days.

- Ampicillin, IV, 25–50 mg/kg/dose 6 hourly.

PLUS

- Gentamicin, IV, 7.5 mg/kg immediately as a single dose.
  - o Follow with 5 mg/kg once daily.

When child improves follow with oral therapy to complete 5–7 days treatment:

- Amoxicillin, oral, 30 mg/kg/dose 8 hourly.

<u>Penicillin allergy</u>
See section 23.4.1: Allergies to penicillins.

In very severe progressive or unresponsive pneumonia consider use of aciclovir for possible herpes infection.

Croup
See section 15.5.2: Laryngotracheobronchitis, acute viral (croup).

Diarrhoea
See section 2.2.4 Diarrhoea, acute.

Encephalitis
See section 8.14: Meningo-encephalitis/encephalitis, acute viral.

Convulsions
See section 13.5: Status epilepticus (convulsive).

Conjunctivitis
- Chloramphenicol ophthalmic ointment 1%, inserted 6 hourly for 5 days.

If corneal clouding/ulceration present obtain urgent ophthalmologic consultation.

Management of contacts
Immunise children older than 6 months if unvaccinated and less than 72 hours since exposure.
Between 3 and 6 days after exposure and for contacts less than 6 months old:
- Immunoglobulin, human normal, IM, 0.25 mL/kg.

If immunodeficient:
- Immunoglobulin, human normal, IM, 0.5 mL/kg.

Immunise all children > 6 months of age if outbreak occurs.

REFERRAL
» Children in need of intensive care unit.
» Children with depressed level of consciousness.
» Children with corneal ulceration/opacity.

8.11 MENINGITIS, ACUTE BACTERIAL
G00
* Notifiable condition. (*N. meningitidis and H. influenzae)*

This guideline applies to children > 60 days old. For the management of neonates, see section 19.11: Meningitis bacterial, neonatal.

DESCRIPTION
Bacterial meningitis most commonly results from haematogenous dissemination of micro-organisms from a distant site, e.g. the nasopharynx. In children, *S. pneumoniae* and *N. meningitides* are the usual pathogens.

Note:
Tuberculosis, cryptococcal and partially treated acute bacterial meningitis should be considered when the clinical and laboratory features are not typical of pyogenic meningitis, or when there is a slow onset of disease (> 2 days), especially in any high risk settings such as immune suppression, TB contact and malnourished children.
Differentiation of TB or cryptococcal meningitis from acute bacterial meningitis is not always easy on presentation.

Complications include:

- Raised intracranial pressure due to cerebral oedema, subdural effusion/empyema or hydrocephalus.
- Other acute complications include:
  - cerebral infarctions,
  - shock,
  - seizures,
  - metastatic infection, e.g. arthritis, pneumonia, pericarditis,
  - disseminated intravascular thrombosis,
  - inappropriate antidiuretic hormone (ADH) secretion.

Long-term neurological sequelae include deafness, blindness, mental retardation and motor paralysis, e.g. hemiparesis.

DIAGNOSTIC CRITERIA

Clinical

- Fever.
- Headache.
- Vomiting.
- Convulsions
- Feeding problems.
- Irritability.
- Lethargy.
- Photophobia.
- Signs of meningeal irritation. In young infants signs of meningism are often absent.
- Signs of increased intracranial pressure, e.g. bulging anterior fontanel.
- Papilloedema is not a useful sign in young children with meningitis. It is difficult to elicit and may be absent even with acutely raised ICP.

Investigations

- Lumbar puncture (LP) (all abnormal findings should lead to serious considerations of acute bacterial meningitis).
  Do not do a LP but initiate treatment immediately if clinical signs of severely raised intracranial pressure, i.e. impending cerebral herniation:
  - decreased or sudden deterioration of level of consciousness,
  - decerebrate or decorticate posturing,
  - neurogenic hyperventilation,
  - unequal dilated or poorly reactive pupils.
- Clinical meningococcaemia (septicaemia) with petechiae/purpura.
  - Confirm with skin scrape, Gram stain and blood culture.
- Bacterial antigen tests on CSF.

GENERAL AND SUPPORTIVE MEASURES

» Admit to high or intensive care unit, if appropriate.
» Monitor, where indicated:
  > neurological status,
  > heart rate,
  > blood pressure,
  > acid-base status,
  > blood glucose,
  > fluid balance, i.e. hydration,
  > respiration,
  > body temperature,
  > haematocrit,
  > electrolytes,
  > blood gases,
  > serum and urine osmolality.
» Ensure adequate nutrition by enteral feeding where possible.
  > Use a nasogastric tube if necessary.
  > If enteral feeding is not possible, give intravenous fluids: paediatric or neonatal maintenance solution with dextrose.

MEDICINE TREATMENT

Antibiotic therapy

Empiric treatment:

- Ceftriaxone, IV, 50 mg/kg/dose 12 hourly.

Adjust antimicrobial therapy according to culture and sensitivity.

Usual duration of treatment (minimum 5 days fever free):

N. meningitidis	7 days
S. pneumoniae	10 days
H. influenzae	10 days

In complicated cases, a longer duration of therapy may be required.
Re-assess antimicrobial therapy when blood and CSF culture and sensitivity results become available, or when improvement is not evident within 72–96 hours.

Seek immediate advice on what treatment to start with when ventriculo-peritoneal shunt infection, spread from sinuses, mastoids, or direct penetrating source of infection is present.

For shunts:

▪ 3rd generation cephalosporin, e.g.:
- Ceftriaxone, IV, 50 mg/kg/dose 12 hourly.

PLUS

- Vancomycin, IV, 15 mg/kg/dose, 6 hourly infused over 1 hour.

Steroid therapy

Empiric treatment before culture results are received.

- Dexamethasone, IV, 0.15 mg/kg 6 hourly for three days starting with or before initiation of antibiotic.
  o If an organism other than *H. Influenzae* is cultured, stop steroid therapy.
  o Do not delay antibiotic treatment if dexamethasone is not readily available.

Fever and headache:
- Paracetamol, oral, 15 mg/kg/dose, 6 hourly as required.

Convulsions
See section 13.5: Status epilepticus (convulsive).

Raised intracranial pressure or cerebral oedema
Elevate head of bed ± 20 degrees.
Maintain $PaCO_2$ at 4–5 kPa (30–35 mmHg); intubate and ventilate if necessary.
Avoid fluid overload.

- Mannitol, IV, 250 mg/kg administered over 30–60 minutes.

- Dexamethasone, IV, 0.5 mg/kg 12 hourly.

Chemoprophylaxis for close contacts
A close contact is defined as someone living in the same household or dormitory, or institution, or children in the same crèche, or any other “kissing” contact. Health care workers who have intimate contact should receive prophylaxis.

N. meningitidis
- Ciprofloxacin, oral, as a single dose.
  - If < 12 years of age: 10 mg/kg.
  - If > 12 years of age: 500 mg.

  Note:
  If < 12 years of age and able to swallow, use a a single 250 mg tablet.

OR
- Ceftriaxone, IM, single dose
  - If < 12 years of age: 125 mg.
  - If > 12 years of age: 250 mg.

Close contacts who are pregnant:
- Ceftriaxone, IM, 250 mg.

H. influenzae prophylaxis for **all** contacts under 5 years who are household contacts (including index case) or day care contacts:
- Rifampicin, oral, 20 mg/kg/dose, once daily for 4 days.
  - Maximum dose: 600 mg
  - Neonatal dose: 10 mg/kg/dose, once daily for 4 days.

Hib vaccination:
Update Hib vaccination in unimmunised or partially immunised children.
Give Hib booster to all children <5 years including index case.
Hib vaccination on its own is not protective in contact situation.

REFERRAL

- » Where lumbar puncture is deferred due to suspected raised intracranial pressure and/or localising signs start bacterial and tuberculous meningitis treatment immediately.
- » Meningitis with complications.
- » All cases of suspected shunt infection. Start treatment immediately before referral.

8.12 MENINGITIS, CRYPTOCOCCAL

G02.1

DESCRIPTION

An uncommon childhood meningitis that may occur in older HIV-infected children with severe CD4 T-cell depletion. Pulmonary and skin involvement can occur.

DIAGNOSTIC CRITERIA

Clinical

- » Acute or chronic headache in an older HIV-infected child. Meningism need not be present.
- » Often presents with cranial nerve palsy.
- » Can occur as result of Immune Reconstitution Inflammatory Syndrome (IRIS) after initiation of antiretroviral therapy.

Investigations

- » Test all cerebrospinal fluid (CSF) specimens from HIV-infected children with suspected meningitis.
- » India ink stain, and/or cryptococcal antigen test – more sensitive than India ink stain.
- » Fungal culture blood and urine.
- » Chest X-ray.
- » Ophthalmological assessment.

GENERAL AND SUPPORTIVE MEASURES

- » Admit to high or intensive care unit, if appropriate.
- » Monitor, where indicated:
  - > neurological status,
  - > heart rate,
  - > blood pressure,
  - > haematocrit,
  - > minerals,
  - > acid-base status,
  - > fluid balance, i.e. hydration.
  - > respiration,
  - > body temperature,
  - > electrolytes,
  - > blood glucose,
  - > blood gases,
  - > serum and urine osmolality, and
- » Ensure adequate nutrition by enteral feeding where possible. Use a nasogastric tube if necessary. If enteral feeding is not possible, give intravenous fluids: paediatric or neonatal maintenance solution with dextrose.

MEDICINE TREATMENT

Treatment

Initial treatment

- Amphotericin B, IV, 0.7–1.0 mg/kg/day as a daily infusion over 4 hours.

PLUS

- Fluconazole, IV, 12 mg/kg/day for 2 weeks.
  - o Maximum dose: 800mg.

Prehydration before administering amphotericin B t o prevent renal impairment:

- Sodium chloride 0.9%, IV, 20 mL/kg **plus** potassium chloride, 20 mmol/L infused over 2–4 hours.

Consolidation treatment

- Fluconazole, oral, 12 mg/kg/day for 8 weeks.
  - o Maximum dose: 800mg.

Secondary prophylaxis (maintenance treatment)

- Fluconazole, oral, 6 mg/kg/day
  - o Maximum dose: 400 mg.

Discontinue secondary prophylaxis:

» Children < 6 years on ART: CD 4 count > 25% for at least 6 months.
» Children > 6 year on ART: CD 4 count > 200 for at least 6 months. Adolescents on ART: CD4 count increases to between 100–200 cells/mm^3 for at least 6 months.
» Re-start prophylaxis if CD4 count drops below thresholds above.

For continued raised intracranial pressure:

» Daily therapeutic lumbar puncture is indicated if initial LP manometric pressure > 25 cm water in the lateral recumbent position.
» Continue until pressure stabilises below 25 cm water.
» Remove 10-20 mL daily and obtain a closing pressure.

REFERRAL

» All cases not responding to initial treatment.
» All patients with IRIS.

8.13 MENINGITIS, TUBERCULOUS (TBM)

G01

* Notifiable condition.

DESCRIPTION

Tuberculous meningitis is an infection of the meninges caused by *M. tuberculosis*. Early diagnosis and treatment improves the prognosis.

Differentiation from acute bacterial meningitis may be difficult. If in any doubt, treat for both conditions.

Complications may be acute or long term:

» Acute:
  > raised intracranial pressure,
  > hydrocephalus,
  > cerebral oedema,
  > brain infarcts,
  > hemi/quadriplegia,
  > convulsions,
  > hyponatraemia due to inappropriate antidiuretic hormone (ADH) secretion or cerebral salt wasting.

  Syndrome of inappropriate antidiuretic hormone secretion (SIADH) and cerebral salt wasting both present with hyponatraemia; the former responding to fluid restriction and the latter to fluid replacement, i.e. sodium chloride 0.9%.

  SIADH has lower serum uric acid and low urine output. Cerebral salt wasting has a normal serum uric acid and high urine output.
» Long term neurological sequelae include: mental handicap, blindness and deafness.

DIAGNOSTIC CRITERIA

Clinical

» History of contact with an infectious tuberculosis case.
» Onset may be gradual with vague complaints of drowsiness (or fatigue), vomiting, fever, weight loss, irritability and headache.
» Later symptoms such as convulsions and neurological fall-out may occur.
» Older children may present with behavioural changes.
» Examination may reveal signs of meningeal irritation and ra ised intracranial pressure, convulsions, cranial nerve palsies, localising signs (such as hemiparesis), altered level of consciousness or coma and choroidal tubercles.
» The degree of involvement is classified into 3 stages. Prognosis relates to the stage of the disease.

  Stage 1: non-specific signs, conscious, rational, no focal neurological signs, no hydrocephalus.

  Stage 2: signs of meningeal irritation, confusion and/or focal neurological signs.

  Stage 3: stupor, delirium, coma and/or neurological signs, i.e. hemiplegia.

Investigations

» CSF findings:
  > May vary depending on the stage.
  > Protein is usually raised.
  > Chloride and glucose are moderately low.
  > Lymphocytes usually predominate.
  > Gram stain is negative and acid-fast bacilli are seldom found.

> In selected cases TB PCR based test on CSF should be done, where available. It may be helpful where it is positive, negative PCR does not exclude TB.
> A negative result does not exclude TB and cultures must still be done. Bacilli may be cultured from the CSF but may take up to 4–6 weeks. If culture positive, also do drug susceptibility test. Always send for culture, do not perform stain as low diagnostic yield from low concentration of organisms wastes CSF sample.

» A Mantoux test and chest X-ray must be done, but are often unhelpful.
» If depressed level of consciousness or focal neurological signs are present, a CT scan is useful (do CT first before LP in such cases).
» Electrolytes: check for hyponatraemia.

GENERAL AND SUPPORTIVE MEASURES

» Monitor neurological status on a regular basis. If rapid deterioration in level of consciousness, consider ventriculoperitoneal shunt.
» Attend to nutritional status. Initially nasogastric feeding is usually needed.
» Rehabilitative measures: most patients need physiotherapy and occupational therapy.
» Surgical treatment for non-communicating hydrocephalus, diagnosed by air encephalogram (VP shunt).
» Communicating hydrocephalus with severely raised pressure may be managed with medicines once hydration status stable and/or with serial lumbar puncture with specialist consultation.

MEDICINE TREATMENT

Differentiation from acute bacterial meningitis may be difficult.
If in doubt, treat for both conditions.

Antituberculosis treatment

Requires therapy with a combination of 4 drugs as a special regimen.
All treatment should be directly observed therapy.
Single drugs may form part of the regimen to provide the total daily required dose for each medicine by supplementing the combination to give the necessary therapeutic dose per kilogram.

A 6-month regimen of all 4 the following drugs:

- Rifampicin, oral, 20 mg/kg as a single daily dose.

PLUS

- Isoniazid, oral, 20 mg/kg as a single daily dose.

PLUS

- Pyrazinamide, oral, 40 mg/kg as a single daily dose.
  - o Maximum daily dose: 2 000 mg.

PLUS

- Ethionamide, oral, 20 mg/kg as a single daily dose.
  - o Maximum daily dose: 1 000 mg.

Consider prolonging treatment for another 3 months if there are concerns about ongoing disease. Consult with a specialist.

In case of suspected/confirmed multidrug-resistant TBM, refer immediately for admission and treatment.

Steroid therapy

- Prednisone, oral, 2mg/kg as a single daily dose for 4 weeks.
  - Maximum daily dose: 60 mg.
  - Taper to stop over further 2 weeks.

Hydrocephalus

Avoid low sodium IV fluids in these patients, i.e. < 60 mmol/L.

To differentiate communicating from non-communicating hydrocephalus an air encephalogram is usually required. Communicating hydrocephalus is more common in this condition.

In children with a sudden deterioration of level of consciousness and other comatose children with TBM, inform the neurosurgeon before doing the air-encephalogram so that shunt surgery can immediately be done if the hydrocephalus is non-communicating. Air-encephalogram procedure: do a lumbar puncture, inject 5 ml of air with a syringe and do immediate lateral X-ray of the skull. Air in the lateral ventricles on skull X-ray indicates communicating hydrocephalus; air at base of brain (not in lateral ventricles), indicates non-communicating hydrocephalus.

<u>Communicating hydrocephalus</u>

If dehydrated, rehydrate with sodium chloride 0.9%, IV.

Start diuretics as soon as patient is well hydrated and serum electrolytes are within the normal range.

- Acetazolamide, oral, 20 mg/kg/dose 8 hourly.
  - Maximum daily dose: 1 000 mg.
  - Monitor for metabolic acidosis and serum potassium derangements.

PLUS

- Furosemide, oral, 0.3 mg/kg/dose 8 hourly for the first month of treatment.
  - Taper slowly over 2 weeks if the intracranial pressure has normalised, as indicated by clinical response or resolution of hydrocephalus on follow-up scan.
  - Do not restrict fluids once on diuretics.

Sudden deterioration of level of consciousness:

- Mannitol, IV, 250 mg/kg administered over 30–60 minutes.

REFERRAL

» TBM not responding to adequate therapy.
» TBM with complications.
» Suspicion of non-communicating hydrocephalus.
» Suspected drug-resistant TB (contact with drug-resistant TB case).

8.14 MENINGO-ENCEPHALITIS/ENCEPHALITIS, ACUTE VIRAL

A86

DESCRIPTION

A number of viruses cause infection of the brain and meninges. Herpes simplex is the most important because it is treatable. A high mortality and morbidity is associated with untreated disease.

Complications include:

» increased intracranial pressure,
» cerebral oedema,
» blindness,
» permanent neurological deficits,
» seizures,
» deafness,
» inappropriate antidiuretic hormone (ADH) secretion.

DIAGNOSTIC CRITERIA

Clinical

» Severe headache, fever, nausea, vomiting, lethargy and abnormal behaviour.
» Alteration in level of consciousness, i.e. drowsiness, confusion, stupor and coma.
» Generalised and/or focal convulsions.
» Focal neurological deficits.
» Abnormal movements, i.e. basal ganglia involvement.
» Cranial nerve palsies (brainstem involvement), loss of sphincter control, paresis of limbs and segmental sensory loss (spinal cord involvement).
» Some patients may have signs of meningeal irritation.
» Herpes encephalitis may have an acute and fulminant course. It can result from primary or recurrent infection.

Investigations

» Laboratory tests are mostly unhelpful.
» CSF may reveal:
  > slightly raised protein,
  > normal glucose level, and
  > mild pleocytosis, mostly lymphocytes.
  > A specific virus is sometimes isolated. PCR is helpful, if available.
  > Red cells are seen with Herpes encephalitis.

» In some instances, the CSF may be completely normal.
» A CT scan of the brain may reveal brain oedema.
  > CT findings may only be apparent after 3–5 days.
  > The herpes virus preferentially involves the temporal lobe and orbital surfaces of the frontal lobes.
» An EEG may demonstrate changes suggestive of herpes encephalitis.

GENERAL AND SUPPORTIVE MEASURES

» Admit to high or intensive care unit, if appropriate.
» Monitor, where indicated:
  > neurological status,
  > heart rate,
  > blood pressure,
  > haematocrit,
  > acid-base status,
  > fluid balance, i.e. hydration,
  > respiration,
  > body temperature,
  > electrolytes,
  > blood glucose,
  > blood gases,
  > serum and urine osmolality.
» Ensure adequate nutrition by enteral feeding where possible.
  > Use a nasogastric tube if necessary.
  > If enteral feeding is not possible, give intravenous fluids: paediatric or neonatal maintenance solution with dextrose.

MEDICINE TREATMENT

If herpes simplex virus or varicella zoster virus encephalitis:

- Aciclovir, IV, 8 hourly administered over 1 hour for 21 days.
  - o If 0–12 years of age: 20 mg/kg/dose.
  - o If > 12 years of age: 10 mg/kg/dose.

Acute convulsions

See section 13.5: Status epilepticus (convulsive).

For fever:

- Paracetamol, oral, 15 mg/kg/dose, 6 hourly until fever subsides.

Raised intracranial pressure or cerebral oedema

Elevate head of bed ± 20 degrees.
Maintain $PaCO_2$ at 4–5 kPa; intubate and ventilate, if necessary.
Avoid fluid overload.

- Mannitol, IV, 250 mg/kg administered over 30–60 minutes.
  - o Do not repeat without consultation with a paediatrician.

REFERRAL

» Deterioration of clinical condition despite adequate treatment.
» Meningo-encephalitis with complications or loss of consciousness.

8.15 MUMPS

B26

DESCRIPTION

Mumps is an acute, communicable, viral disease of childhood that commonly affects the salivary glands, chiefly the parotid gland, and frequently the central nervous system.

The incubation period is 2–3 weeks.

Complications include:

» meningo-encephalitis,
» pancreatitis,
» orchitis,
» facial nerve paresis,
» nephritis.
» oophoritis,
» thyroiditis,
» nerve deafness,
» myocarditis,

DIAGNOSTIC CRITERIA

Clinical

» A prodrome of 1–2 days may precede the salivary gland involvement and is characterised by fever, malaise, headache and pain in or behind the ear on chewing or swallowing.
» Painful enlargement of the parotid gland/s with the ear usually displaced upward and outward with the mandibular angle obliterated. The submaxillary and sublingual glands may also be involved.
» Pain may be referred to the ear.
» Papilla of Stensen’s duct opposite the upper second molar may be oedematous and red.
» Central nervous system involvement may occur alone or may precede, accompany or follow inflammation of the salivary glands.

Investigations

» Leucopaenia with relative lymphocytosis.

GENERAL AND SUPPORTIVE MEASURES

» Isolate patient until salivary gland enlargement subsides.
» Maintain adequate nutrition and hydration.
» Patient may return to school after swelling has subsided.

MEDICINE TREATMENT

Treat complications as appropriate.

For pain and fever:

• Paracetamol, oral, 15 mg/kg/dose, 6 hourly as required.

REFERRAL
» Mumps with complications not responding to adequate therapy.

8.16 MYCOBACTERIUM AVIUM COMPLEX (MAC) INFECTION
A31.0

DESCRIPTION
Atypical mycobacterium, causing disease in extremely immunocompromised patients.
MAC infection in HIV-infected children usually presents with disseminated disease, often enlarged intra-abdominal lymph nodes and pancytopaenia. Pulmonary, GIT or skin disease is less common.

DIAGNOSTIC CRITERIA
» MAC may be isolated from blood, bone marrow, lymph node, other sterile fluids and tissues.
» Confirm diagnosis with a biopsy for histology and culture **or** 2 culture-positive sputa or gastric aspirates.
» PCR line probe test can be used for diagnosis.

GENERAL AND SUPPORTIVE MEASURES
» If MAC infection is localised to a single enlarged peripheral lymph node, an excision of the lymph node is therapeutic.

MEDICINE TREATMENT
To be managed under specialist care.
Therapy consists of a combination of at least two medicines.

▪ New generation macrolide e.g.:
• Clarithromycin, oral, 7.5 mg/kg/dose 12 hourly.

PLUS
• Ethambutol, oral, 20–25 mg/kg once daily.

For extensive disease in severely immunodeficient child:
ADD
▪ Fluoroquinolone, e.g.:
• Levofloxacin, oral, 20 mg/kg once daily.

PLUS
• Amikacin, IV, 15 mg/kg once daily.

REFERRAL
» No response to treatment.
» Fibrocavitating pulmonary disease

8.17 PERTUSSIS

A37.9
* Notifiable condition

DESCRIPTION

A communicable respiratory infection usually recognised by a paroxysmal cough followed by an inspiratory whoop (absent in young infants) and associated vomiting. Subconjunctival haemorrhages may be present. The cough can persist for 3 months or longer with the infectious period between 2 weeks and 3 months. The disease is more severe in young infants where it may present with apnoea rather than the inspiratory whoop.
Incubation period: 7–10 days. Range: 6– 21 days.

DIAGNOSTIC CRITERIA

» Diagnosis is clinical.
» A definitive diagnosis often not possible with respect to viral pertussis-like syndrome.
» FBC usually very high WCC with > 50% lymphocytosis.
» Use naso-pharyngeal aspirates if possible for special cultures for *Bordetella pertussis* or identify by using PCR.

GENERAL AND SUPPORTIVE MEASURES

» Isolate patient during first 2 days whilst on antibiotic therapy.
» Clear airways by gentle suction taking care not to induce cough.
» Appropriate respiratory support for apnoea or respiratory distress/failure.
» Encourage oral feeding. If unsuccessful provide nasogastric feeds with small volumes.
» Immunise infant against pertussis even if diagnosis of pertussis was made.

MEDICINE TREATMENT

If hypoxic:

- Oxygen, 1–2 L/minute via nasal prongs.

▪ New generation macrolide e.g.:
- Clarithromycin, oral, 7.5 mg/kg/dose 12 hourly for 7 days.

For fever:

- Paracetamol, oral, 15 mg/kg/dose, 6 hourly as required.

<u>Management of contacts</u>

Prophylaxis only for unimmunised or partly immunised infants < 6 months of age:

- Macrolide e.g.:
- Clarithromycin, oral, 7.5 mg/kg/dose 12 hourly for 7 days.

REFERRAL

» Children with seizures or encephalopathy for further evaluation.
» Infants requiring intensive care, where none is available on site.

8.18 PNEUMOCYSTIS JIROVECI PNEUMONIA (PCP)

B20.6

See section 15.3.2.6: Pneumonia in HIV exposed or infected children.

8.19 POLIOMYELITIS (ACUTE FLACCID PARALYSIS)

A80.3
* Notifiable condition

Also see section 13.9.1: Inflammatory Polyneuropathy (Guillain-Barré Syndrome).

DESCRIPTION

Poliomyelitis is caused by polio virus types 1, 2 and 3. It mainly affects children under 5 years of age, but a person at any age who does not have immunity may be infected. Risk of paralysis increases with age.

Poliomyelitis is uncommon. Most cases of acute flaccid paralysis (AFP) are caused by Guillain-Barré Syndrome, but all cases of AFP should be notified. Humans are the only reservoir. The faecal-oral route is the major route of transmission, although droplet spread can also occur.
Incubation period is between 7–14 days.

DIAGNOSTIC CRITERIA

Clinical

» Polio virus infection is asymptomatic in 90–95% of cases.
» ± 4–8% will develop abortive polio with some or all of the following symptoms:
  > fever,
  > headache,
  > stiff neck,
  > muscle pain,
  > nausea,
  > vomiting and diarrhoea.
» ± 1% may present as viral meningitis.
» The remaining 1–2% will develop paralysis of sudden rapid onset reaching full development in hours, maximum 3 days.
» Paralysis is often asymmetrical and always flaccid. Reflexes are absent.
» Sensation usually not affected.
» Lower limbs are affected more than upper limbs and proximal more than distal muscles.

Investigations

» Send two stool specimens taken 24–48 hours apart to the National Institute of Communicable Diseases via the local laboratory.

GENERAL AND SUPPORTIVE MEASURES

» Isolate patient to prevent faecal-oral spread.
» Rehabilitative measures:
  > Most patients need physiotherapy and occupational therapy.

MEDICINE TREATMENT

Prevention

Immunise all children, including HIV-infected children, according to the EPI programme.
Vaccine associated/derived atypical paralytic polio is only associated with live oral polio vaccine (given in new EPI programme only at birth and 6 weeks) and not associated with inactivated polio vaccine (IPV), which is given from 6 weeks on.
IPV contraindicated if: previous anaphylactic reaction following IPV or if anaphylaxis with streptomycin, neomycin or polymyxin B.

REFERRAL

» Children requiring intensive care, if none is available on site.

8.20 RABIES

A82.9
* Notifiable condition
Inform state veterinarian or local veterinary official.

DESCRIPTION

A viral infection of the central nervous system following transmission of the rabies virus from the saliva of affected animals through bites or contamination of mucosa or skin lesions.
Incubation period 2–8 weeks.

DIAGNOSTIC CRITERIA

Clinical

» Signs and symptoms may begin with:
  > fever,
  > headache,
  > nausea,
  > diarrhoea,
  > irritability.
» Early signs include paraesthesia or itching at site of bite in ⅓ of cases.
» The acute neurologic phase interspersed with lucid periods manifests with:
  > agitation,
  > mania,
  > hyperactivity,
  > hallucinations.

- Seizures may be precipitated by auditory or tactile stimuli.
- Hypersalivation, hydrophobia or aerophobia may occur.
- Death is usually due to cardio-respiratory failure.

Investigations

- Virus specific fluorescent antigen in brain tissue confirms diagnosis in animals.
- Preserve brain tissue of the dead animal.

GENERAL AND SUPPORTIVE MEASURES

- Symptomatic and supportive treatment.
- Prompt cleansing of the bite wound.
- Do not suture puncture wounds.
- Seek advice.

TELEPHONE HOTLINE	
National Institute of Communicable Diseases	011 386 6337 or 011 386 6000
After hours	082 883 9920

Post exposure prophylaxis

Caution
Start post exposure prophylaxis immediately.
Do not wait for confirmatory laboratory tests in the animal.

Post exposure prophylaxis may be life saving and should always be given if there is the slightest suspicion that the animal may have been rabid.
The decision to give post exposure prophylaxis is based on the risk of rabies transmission, the species and behaviour of the animal and the nature of the bite. No laboratory test on t he human victim is p ossible or av ailable to confirm or exclude possible transmission.

MEDICINE TREATMENT TO PREVENT INFECTION

Treatment depends on the risk category.

Risk Category	Type of exposure	Action
1.	» touching or feeding animal<br>» licking intact skin	» none if reliable history
2.	» nibbling uncovered skin<br>» superficial scratch without bleeding<br>» licking broken skin	» wound treatment<br>» give rabies vaccine<br>» do not give rabies immunoglobulin (RIG)<br>Stop vaccination if laboratory tests of animal are negative for rabies or animal, i.e. dog or cat remains well after 10 days observation.
3.	» bites or scratches penetrating skin and drawing blood<br>» licking of mucous membranes	» wound treatment<br>» give rabies vaccine<br>» give rabies immunoglobulin (RIG)<br>» give tetanus toxoid vaccine and antibiotic<br>Stop vaccination if laboratory tests of animal are negative for rabies or animal, i.e. dog or cat, remains well after 10 days observation.

Wound treatment

Local wound care:

Flush wound thoroughly and clean with soap and water or sodium chloride 0.9% or chlorhexidine 0.05%.

- Povidone iodine 10%, topical.

For penetrating wounds:

- Tetanus toxoid (TT), IM, 0.5 mL.

Pre-emptive antibiotic only if hand is bitten or for extensive wounds or human bites. Data does not support the use of antibiotics in minor animal bites.

- Amoxicillin/clavulanic acid, oral, 30 mg/kg/dose of amoxicillin component 8 hourly.

Rabies Vaccine

Must be given for category 2 and 3 bites.

Vaccine is administered on days 0, 3, 7, 14, 28. Vaccine is ideally given as soon as possible after exposure, but should still be given if patient presents some time after the exposure.

If vaccine administration is delayed > 48 hours, a double dose should be given initially.

Rabies vaccine is given IM but never in the buttock. Give to deltoid muscle in adults and antero-lateral aspect of thigh in infants.

Adverse events are uncommon and include:

» Local reactions:
    > pain,
    > erythema,
    > swelling or itching at the injection site.
» Systemic reactions:
    > fever,
    > arthralgia,
    > arthritis,
    > angioedema,
    > nausea,
    > vomiting,
    > malaise.

Rabies Immunoglobulin (RIG)

Must be given for category 3 bites only. Always give the vaccine first.

Immunoglobulin must be given as soon as possible after exposure, but may be administered up to 7 days after the first vaccine is given.

Do not give RIG if the patient has previously received pre- or post-exposure prophylaxis.

- Rabies immunoglobulin, 20 units/kg.
    - o Infiltrate around wound with up to 50% of dose.
    - o Administer remaining immunoglobulin into deltoid muscle opposite to vaccine administration site.
    - o If multiple wounds, dilute in sodium chloride 0.9% to 2–3 times so that **all** wounds are infiltrated.
    - o **Do not** exceed maximum dose as antibody production to the vaccine is inhibited.
    - o If unavailable, **do not** delay active immunisation.

REFERRAL

» Where prophylactic treatment is not immediately available.
» All cases of human clinical rabies for appropriate palliative care.

8.21 TETANUS

A35
* Notifiable condition.

DESCRIPTION

Tetanus is an acute spastic paralytic illness caused by tetanospasmin, the neurotoxin produced by *Clostridium tetani*. The toxin prevents neurotransmitter release from spinal inhibitory neurons.

Complications include:

» asphyxia,
» dehydration,
» hyperpyrexia,
» inability to suck, chew and swallow.
» bronchopneumonia,
» respiratory failure,
» laryngospasm,

DIAGNOSTIC CRITERIA

The diagnosis is made on clinical grounds.

Clinical

» Unimmunised/incompletely immunised child.
» History of wound/trauma or unhygienic care of umbilical cord/stump.
» Trismus.
» Stiffness of the neck, back and abdominal muscles.
» Pharyngospasm, laryngospasm, dysphagia, inability to suck, chew and swallow which severely compromises feeding and eating activities.
» Spontaneous muscle contractions/spasms or muscle contractions/ spasms triggered by minimal stimuli such as touch, sound, light or movement.
» No involvement of sensorium, i.e. consciousness is not disturbed.
» Autonomic nervous system instability with hypertension, tachycardia and dysrhythmias.

GENERAL AND SUPPORTIVE MEASURES

» Admit to high or intensive care unit, if available.
» Ventilatory support, if needed.
» Monitor:
  > temperature,
  > respiration,
  > heart rate,
  > blood gases,
  > SaO_2.
  > blood pressure,
  > blood glucose,
  > electrolytes,
  > acid-base status,
» Protect the patient from all unnecessary sensory and other stimuli.
» Ensure adequate hydration and nutrition.
» Wound care and debridement/umbilical cord care.
» Educate parents/caregivers regarding prevention of tetanus by vaccination.

MEDICINE TREATMENT

For hypoxia:

- Oxygen 100% by nasal canula.

- Tetanus immunoglobulin, IM, 500–2 000 IU as a single dose.

- Tetanus toxoid (TT), IM, 0.5 mL
  - Not required in immunised patients who have received a booster within the past 5 years.

- Metronidazole, IV, 7.5 mg/kg/dose 8 hourly for 10 days duration.

For control of muscle spasms:

- Diazepam, IV, 0.1–0.2 mg/kg/dose 4–6 hourly, titrated according to response.
  - Do not exceed 10 mg/dose.
  - After improvement, use enteral form in high care setting.
  - For ventilation and muscle relaxants, see section 21.1.2: ICU sedation, infant and child.

After recovery from tetanus, patients should be actively immunised as the disease does not confer immunity.

Prevention of tetanus

Minor wounds

Children with clean minor wounds do not require tetanus immunoglobulin or antibiotics. Tetanus vaccine should be given, except in fully immunised patients who have received a booster within the past 5 years.

For more severe wounds

If child with penetrating wound not completely immunised:

- Tetanus immunoglobulin (TIG), IM.
  - If < 5 years of age: 75 IU.
  - If 5–10 years of age: 125 IU.
  - If > 10 years of age: 250 IU.

- Tetanus toxoid (TT), IM, 0.5 mL.
  - Not required in immunised patients who have received a booster within the past 5 years.

REFERRAL

» All severe cases.

8.22 TICK BITE FEVER

A79.9

DESCRIPTION

A febrile illness with exanthem caused by *Rickettsia conorii* with the tick as vector. Recently, other tick-borne Rickettsial diseases have been identified.

The rash appears on days 3–5 of the illness. It spreads from the extremities to the trunk, neck, face, palms, and soles within 36 hours.
The lesions progress from macular to maculopapular and may persist for 2–3 weeks.
Atypical cutaneous findings may occur in a few patients.

Complications include:

» vasculitis,
» thrombosis,
» myocarditis,
» thrombocytopaenia.
» encephalitis,
» renal failure,
» pneumonitis, and

DIAGNOSTIC CRITERIA

The diagnosis is made on clinical grounds.

Clinical

» Fever, headache, malaise, myalgia and arthralgia.
» Maculopapular rash which may involve the palms and soles.
» Eschar at the site of the tick bite is associated with regional lymphadenopathy and splenomegaly.

Investigations

» Diagnosis can be confirmed retrospectively by immunofluorescent antibody techniques.

GENERAL AND SUPPORTIVE MEASURES

» Remove tick as soon as possible after detection on the body.

MEDICINE TREATMENT

Antibiotic therapy

Treatment must be started before confirmation of diagnosis by serology.
Although not recommended for children < 8 years of age, doxycycline is still regarded as the drug of choice for children with tick-bite fever.

- Doxycycline, oral.
  - o If < 50 kg: 4 mg/kg/24 hours in 2 divided doses on the first day, then 2 mg/kg/24 hours in 2 divided doses for 7 days.
  - o If > 50 kg: 100 mg 12 hourly for 7 days.

If unable to take oral therapy:

- Clarithromycin, IV, 7.5 mg/kg/dose 12 hourly for 7 days.

For headache and fever:

- Paracetamol, oral, 15 mg/kg/dose, 6 hourly as required.

REFERRAL

» Patients not responding to adequate therapy.
» Patients with complications.

8.23 TOXOPLASMOSIS

B58.9

DESCRIPTION

Rarely occurs in children.
Usually presents as encephalitis, with focal neurological abnormalities occurring in association with headache.
Ocular and pulmonary disease is also seen.

DIAGNOSTIC CRITERIA

Investigations

» Diagnosis may be made on blood and CSF serology.
» CSF PCR for toxoplasmosis may also be helpful.
» CT scan usually reveals multiple bilateral, focal hypodense ring-enhancing lesions.

REFERRAL

» All cases.

8.24 TYPHOID

A01.1
* Notifiable condition.

DESCRIPTION

A systemic disease caused by *Salmonella typhi*.

DIAGNOSTIC CRITERIA

Clinical:

» fever,
» headache,
» diarrhoea or constipation,
» abdominal pain or tenderness,
» cough,
» delirium,
» meningismus,
» anorexia,
» vomiting,
» ileus,
» epistaxis,
» hepatomegaly and/or splenomegaly,
» stupor.

Investigations:

» Leucopaenia, anaemia and thrombocytopaenia.
» Positive cultures from blood (1st week), stool (after 1st week), urine and bone marrow.
» Serology may be helpful.

GENERAL AND SUPPORTIVE MEASURES

» Isolate patient until 3 consecutive stools are culture negative.
» Correct and maintain fluid and electrolyte status.

MEDICINE TREATMENT

Note:
Relapse and carrier state may occur despite adequate therapy.

If haemoglobin < 7 g/dL:

- Packed red cells, IV, 10 mL/kg.

▪ 3rd generation cephalosporin, e.g.:

- Ceftriaxone, IV, 50 mg/kg once daily for 7 days.
  - o In severe disease prolong treatment up to 14 days.

If cephalosporin resistance consider fluoroquinolones:

- Ciprofloxacin, oral, 10-20 mg/kg/dose 12 hourly for 7 days.

SURGICAL TREATMENT

Surgical intervention for bowel perforation, osteomyelitis, etc.

REFERRAL

» Inadequate response to treatment.
» Patients with complications.

8.25 NON-TYPHOID SALMONELLA (NTS)

A02.9

DESCRIPTION

Present as:

» gastroenteritis, or
» extraintestinal (invasive) disease.

DIAGNOSTIC CRITERIA

Clinical

» Self-limiting mucosal intestinal disease presenting with diarrhoea and vomiting in immunocompetent patients.
» Young infants (< 3 mon ths) and immunodeficient children (especially HIV-infected children) are prone to invasive, even recurrent disease.

» Invasive disease includes bacteraemia (fever), osteomyelitis and meningitis.
» There is also an association of invasive NTS with malaria and severe anaemia.

Investigations

» Positive cultures from blood, stool, urine and bone marrow.
» Serology may be helpful.

GENERAL AND SUPPORTIVE MEASURES

» Correct and maintain fluid and electrolyte status.

MEDICINE TREATMENT

Note:
Relapse may occur despite adequate therapy. Antibiotic therapy in NTS gastroenteritis may prolong excretion of Salmonella.

If haemoglobin < 7 g/dL:

- Packed red cells, IV, 10 mL/kg.

Gastroenteritis

Antibiotic therapy is **not** generally recommended. However, in infants < 3 months of age and severely immunocompromised children at high risk of developing invasive disease treat as for invasive disease.

Invasive disease

If < 1 month of age:

- Cefotaxime, IV/IM, 50–75 mg/kg/dose 8 hourly.

OR

If > 1 month of age:

- Ceftriaxone, IV, 50–80 mg/kg once daily.
  Duration:
  - Bacteraemia: 10–14 days.
  - Acute osteomyelitis: 4–6 weeks.
  - Meningitis: 4 weeks.

If cephalosporin resistance treat according to sensitivity.

SURGICAL TREATMENT

Surgical intervention for osteomyelitis, etc.

REFERRAL

» Inadequate response to treatment.
» Patients with complications.

8.26 VARICELLA (CHICKEN POX)

B01

DESCRIPTION

An acute, highly contagious, viral disease caused by herpes varicella-zoster. It spreads by infective droplets or fluid from vesicles. One attack confers permanent immunity. Varicella is contagious from about 5 days before the onset of the rash until the crusts begin to disappear.

Re-activation of the virus may appear later as herpes zoster or shingles (in children, consider immunosuppression if this occurs).
Incubation period is 2–3 weeks.

Complications are more common in immunocompromised patients and include:

» secondary skin infection,
» pneumonia,
» necrotising fasciitis,
» encephalitis,
» haemorrhagic varicella lesions with evidence of disseminated, intravascular coagulation.
» Two important bacteria causing complications are *Staphylococcus aureus and Streptococcus pyogenes*.

DIAGNOSTIC CRITERIA

Clinical

» Mild headache, fever and malaise.
» Characteristic rash.
» The lesions progress from macules to vesicles in 24–48 hours.
» Successive crops appear every few days.
» The vesicles, each on an erythematous base, are superficial, tense 'teardrops' filled with clear fluid which dry to form fine crusts.
» The rash is more profuse on the trunk and sparse at the periphery of extremities.
» At the height of eruption, all stages (macules, vesicles and crusts) are present at the same time.
» The rash lasts 8–10 days and heals without scarring, unless secondarily infected.
» Mucous membranes may be involved.
» Pruritus may be severe.
» Patients are most contagious from 1–2 days before onset of the rash until crusting of lesions.

GENERAL AND SUPPORTIVE MEASURES

» Isolate the patient.
» Isolate the neonate until the mother is regarded as non-contagious.
» Maintain adequate hydration.

MEDICINE TREATMENT

Antiviral therapy

Indicated for immunocompetent patients with varicella complications and for all immunocompromised patients.
Initiate as early as possible, preferably within 24 hours of the appearance of the rash.

Immunocompromised patients and all cases with severe chickenpox (not encephilits)

- Aciclovir, oral, 20 mg/kg/dose 6 hourly for 7days.
  - o Maximum dose: 800 mg/dose.

In severe cases or in cases where oral medicine cannot be given:

- Aciclovir, IV, 8 hourly administered over 1 hour for 21 days.
  - o If 0–12 years: 20 mg/kg/dose 8 hourly.
  - o If >12 years: 10 mg/kg/dose 8 hourly.

For encephalitis:
See section 8.14: Meningo-encephalitis/encephalitis, acute viral.

For fever:

- Paracetamol, oral, 15 mg/kg/dose, 6 hourly as required.

For mild pruritus:

- Calamine lotion, topical, applied 8 hourly.

For severe pruritus:

- Chlorphenamine, oral, 0.1 mg/kg 6–8 hourly for 24–48 hours.

Secondary skin infection

- Cephalexin, oral, 12.5 mg/kg/dose, 6 hourly for 5 days.

Prophylaxis

Post exposure prophylaxis must be given to:
Neonates whose mothers develop varicella from 5 days before delivery to 2 days after delivery and for an exposure:

- Varicella-zoster immunoglobulin, IM, 1 mL (100 units) given within 96 hours of exposure.

If varicella-zoster immunoglobulin is not available:

- Aciclovir, oral, 20 mg/kg/dose 6 hourly for 10 days.
  Note**:**
  In neonates, prophylaxis may not prevent disease.

Infants and children > 28 days

Immunocompromised children exposed to varicella:

- Aciclovir, oral, 20 mg/kg/dose 8 hourly for 10 days given in the second week after exposure.

Hospitalised immunocompetent children exposed to varicella (to limit spread).

- Varicella-zoster vaccine, IM, 0.5 mL given within 72 hours of exposure.

OR

- Aciclovir, oral, 20 mg/kg/dose 8 hourly for 10 days given in the second week after exposure.

REFERRAL

» Neonates with varicella.
» Patients with complications.

8.27 ZOSTER

B02

DESCRIPTION

A vesicular eruption in a dermatomal pattern, which does not cross the midline, due to reactivation of herpes varicella-zoster virus.
Occurs commonly in immunocompromised children and occasionally in immunocompetent children.

DIAGNOSTIC CRITERIA

Usually made on clinical grounds.

Investigations

» Confirm diagnosis by viral culture or Tzanck preparation.

GENERAL AND SUPPORTIVE MEASURES

» Isolate patient.

MEDICINE TREATMENT

Within 24 hours of the appearance of the rash for less severe cases:

- Aciclovir, oral, 20 mg/kg/dose 6 hourly for 7 days.
    - o Maximum dose: 800 mg/dose.

If oral treatment cannot be taken and for severe cases:

- Aciclovir, IV, 8 hourly administered over 1 hour for 21 days.
    - o 0–12 years 20 mg/kg/dose
    - o >12 years: 10 mg/kg/dose

In older children where pain may become a problem:

- Carbamazepine, oral, 5 mg/kg/dose every 8 hours.

REFERRAL

» Disseminated zoster.

8.28 SEPSIS (OUTSIDE THE NEONATAL PERIOD)

A41.9

DESCRIPTION

Severe sepsis is an uncontrolled inflammatory response as a result of suspected or proven infection. (Systemic Inflammatory Response Syndrome)

Clinical features include:

» raised cardiac output,
» decreased systemic resistance,
» warm extremities,
» a wide pulse pressure.

The hyperdynamic state is recognised by hyperpyrexia, hyperventilation, tachycardia, and mental confusion.
A widespread scarlatiniform rash with secondary desquamation, conjunctivitis, strawberry tongue, vomiting and watery diarrhoea may be present in cases of toxic shock. Haemorrhagic skin lesions are present in many children with meningococcaemia, but may also be present in other severe sepsis cases. Children 2–3 years of age may present with a history of poor feeding, mottled appearance of the skin, acidosis, and inconsolable crying.

DIAGNOSTIC CRITERIA

Clinical

» A systemic inflammatory response with at least two of the following four criteria, one of which must be abnormal temperature or leukocyte count:
  > core temperature of > 38.5ºC or < 36ºC,
  > tachycardia,
  > tachypnoea,
  > elevated leukocyte count,

  plus one of the following:
  > cardiovascular dysfunction,
  > acute respiratory distress syndrome, or
  > ≥ 2 other organ dysfunctions.

Investigations

» Blood culture and identify focus of infection e.g. osteomyelitis, abscess.
» Investigate for malaria especially in endemic areas or if there is a history of travel.
» Where meningitis due to meningococcus is suspected, i.e. with petechial rash, lumbar puncture is contra-indicated if the patient is shocked. Do petechial scrapes and blood culture to confirm diagnosis.

GENERAL AND SUPPORTIVE MEASURES

» Admit to high care area.
» Early recognition and treatment of septic shock.

MEDICINE TREATMENT

Empiric antibiotic therapy

Choice of antibiotic depends on the severity of the condition and predisposing factors.

Reconsider choice of antibiotic when the results of cultures become available or the child does not improve.

- Ceftriaxone, IV, 50 mg/kg/dose 12 hourly for 7days.

Suspected meningococcal septicaemia

- Ceftriaxone, IV, 80 mg/kg/dose 12 hourly.

Confirmed meningococcal septicaemia

- Benzylpenicillin (Penicillin G), IV, 100 0 00 units/kg/dose immediately, then 4 hourly for 7 days.

Suspected staphylococcal infection (e.g. osteomyelitis)

- Cloxacillin, IV, 50 mg/kg/dose 6 hourly.

PLUS

- Ceftriaxone, IV, 50 mg/kg/dose, 12 hourly.

Continue IV antibiotics until there is evidence of good clinical response and laboratory markers of in fection improve (usually about 2 weeks). Oral antibiotics may then be considered.

Nosocomial sepsis: manage according to the ba ckground microbiological flora within your institution.

Septic shock

See section 1.1.7: Shock.

REFERRAL

» Septicaemia with complications.
» Patients requiring intensive care.

8.29 STAPHYLOCOCCAL SEPTICAEMIA

A41.2

DESCRIPTION

Staphylococci cause disease by direct invasion of tissues w ith liberation of toxins. Septicaemia may occur when haematogenous dissemination occurs from a focus of infection.

DIAGNOSTIC CRITERIA

Clinical

Features of septicaemia should raise an index of suspicion of staphylococcal infection.

Suggestive features of staphylococcal infection include:

» presence of abscesses,
» erythema of palms and soles,
» osteomyelitis,
» septic arthritis, and
» endocarditis.

Investigations

» Send pus for culture and sensitivity.
» Blood cultures are frequently negative in serious staphylococcal infection, a finding that demonstrates the need for performing other cultures.

GENERAL AND SUPPORTIVE MEASURES

» Surgical drainage or aspiration of pus.
» If infection is associated with a foreign body, such as an intravenous catheter, remove catheter and swab for culture and sensitivity.

MEDICINE TREATMENT

When *S. aureus* isolates are likely to be the cause of infection, the most appropriate agents to administer for empiric treatment are based on the relative frequency of CA-MRSA isolates in the particular community.

Sensitive staphylococci:

- Cloxacillin, IV, 50 mg/kg/dose 6 hourly.

Staphylococcus (bone and joint)

- Cloxacillin, IV, 50 mg/kg/dose 6 hourly.

Methicillin resistant staphylococci (proven/suspected) and signs of serious illness:

- Vancomycin, IV, 15 mg/kg/dose, 6 hourly infused over 1 hour.

REFERRAL

» Severe sepsis with organ dysfunction.
» Septic shock after resuscitation.
» Staphylococci resistant to above antibiotics.

CHAPTER 9

HUMAN IMMUNODEFICIENCY VIRUS INFECTION

9.1 HUMAN IMMUNODEFICIENCY VIRUS INFECTIONS

B20–24

Comprehensive guidelines are available for ART and the care of children with HIV infection in the National Antiretroviral Treatment Guidelines and the PMTCT Guidelines

DESCRIPTION

Human Immunodeficiency Virus (HIV) is a retrovirus infecting immune cells, especially CD4 T lymphocytes. In advanced HIV disease the body loses its ability to fight infections and this stage is characterised by severe damage to organs, opportunistic infections, malignancies and very low CD4 counts.

In infants most infection is transmitted from mother to child, while in adolescents and adults, sexual transmission is the usual route.

Infants born of HIV infected mothers may be shown to be HIV infected, at risk of being/becoming HIV infected ("HIV exposed"), or known to not have HIV infection. To exclude HIV infection in these HIV exposed infants/children, a DNA PCR test (or if 18 months or older, a rapid test or ELISA) should be done in infants at least 6 weeks of age or at least 6 weeks following cessation of breastfeeding and should be negative.

DIAGNOSTIC CRITERIA

Suspect HIV infection in the following situations:

» Exposure of foetus/baby to a HIV infected mother.
» Sexual abuse.
» Adolescents having unprotected sexual encounters.
» Parents with tuberculosis or HIV.
» Any child with tuberculosis.
» Clinical features of symptomatic HIV infection.
» Unexplained severe dermatoses.
» Persistent/recurrent ear discharge.
» Severe progressive pneumonia especially in the first 6 months of life.
» *Pneumocystis jiroveci* (*carinii*) and/or Cytomegalovirus pneumonia.
» Low weight for age or unsatisfactory weight gain.
» Persistent or recurrent diarrhoea in the past three months.
» Enlarged lymph nodes in two or more of the following sites: neck, axilla or groin.

» Oral thrush outside the neonatal period.
» Parotid gland swelling.
» Liver enlargement.
» Spleen enlargement.
» Recurrent infections including pneumonia, ear infections, sinusitis, osteitis and arthritis.
» Digital clubbing.
» Progressive developmental delay.
» The combination of multiple problems.

Confirmation of HIV infection

Children < 18 months of age:

» Do HIV DNA PCR at 6 weeks of age in all HIV exposed infants.
» If positive, confirm with the second baseline DNA PCR test. In itiate treatment while awaiting the second PCR test result.
» If the child is breast fed and the 6-week HIV DNA PCR is negative, repeat testing 6 weeks after complete cessation of breastfeeding. (If the child is 18 months or older, do an ELISA or rapid test).
» If at any time the child has evidence suggesting HIV infection, even if this is before 6 weeks of age or the chi ld has had a prev ious negative PCR, the child should be tested for HIV infection.

In children ≥ 18 months of age:

» Do HIV rapid/ELISA test.
» If 1st test is positive, confirm the result with a second test using a kit of a different manufacturer, and preferably on different blood specimens.
  Note:
  Rapid tests may be le ss reliable in ch ildren with advanced disease. If clinical findings suggest HIV infection but the rapid test is negative, send a further specimen of blood to the laboratory for formal ELISA testing. If test results are still equivocal do an HIV DNA PCR test.

Note:

» Negative tests do not exclude infection until 6 weeks after birth and 6 weeks after exposure to other risk of HIV infection (including cessation of breastfeeding).
» Children with discordant HIV test re sults must be discussed with an expert.

Adapted WHO clinical staging of HIV and AIDS for infants and children

For persons aged under 15 years with confirmed laboratory evidence of HIV infection

Clinical Stage 1

» asymptomatic
» persistent generalised lymphadenopathy (PGL)

Clinical Stage 2

» hepatosplenomegaly
» papular pruritic eruptions
» seborrhoeic dermatitis
» extensive human papilloma virus infection
» extensive molluscum contagiosum
» fungal nail infections
» recurrent oral ulcerations
» lineal gingival erythema (LGE)
» angular cheilitis
» parotid enlargement
» herpes zoster
» recurrent or chronic RTIs, i.e.
  > otitis media
  > otorrhoea
  > sinusitis

Clinical Stage 3

» moderate unexplained malnutrition (weight for age/height between the 3rd percentile and 60% of expected weight) not adequately responding to standard therapy
» unexplained persistent diarrhoea (14 days or more)
» unexplained persistent fever (intermittent or constant, for longer than one month)
» oral candidiasis (outside neonatal period)
» oral hairy leukoplakia
» acute necrotising ulcerative gingivitis/periodontitis
» pulmonary TB
» severe recurrent presumed bacterial pneumonia
» chronic HIV-associated lung disease including bronchiectasis
» lymphoid interstitial pneumonitis (LIP)
» unexplained anaemia (< 8 g /dL), and or neutropaenia (< 500/mm^3) and/or thrombocytopaenia (< 50 000/mm^3) for more than one month

Clinical Stage 4

- » unexplained severe wasting or severe malnutrition not adequately responding to standard therapy
- » pneumocystis pneumonia
- » recurrent severe presumed bacterial infections, e.g.
  - > empyema
  - > pyomyositis
  - > bone or joint infection
  - > meningitis

  but excluding pneumonia
- » chronic herpes simplex infection; (oro-labial or cutaneous of more than one month's duration)
- » extrapulmonary TB
- » Kaposi's sarcoma
- » oesophageal candidiasis
- » CNS toxoplasmosis (outside the neonatal period)
- » HIV encephalopathy
- » CMV infection (CMV retinitis or infections of organs other than liver, spleen or lymph nodes; onset at age one month of more)
- » extrapulmonary cryptococcosis including meningitis
- » any disseminated endemic mycosis, e.g.
  - > extrapulmonary histoplasmosis
  - > coccidiomycosis
- » cryptosporidiosis
- » isosporiasis
- » disseminated non-tuberculous mycobacteria infection
- » candida of trachea, bronchi or lungs
- » visceral herpes simplex infection
- » acquired HIV associated rectal fistula
- » cerebral or B cell non-Hodgkin lymphoma
- » progressive multifocal leukoencephalopathy (PML)
- » HIV-associated cardiomyopathy or HIV-associated nephropathy

9.1.1 THE HIV EXPOSED INFANT

Z20.6

DESCRIPTION

An infant whose mother is HIV infected and in which infant HIV infection has neither been confirmed nor excluded.

Transmission of HIV infection from mother to child may occur during pregnancy, during delivery, or via breast feeding. Prevention of transmission of infection from mother to child can be effectively carried out with a very high success rate by means of fully suppressing the mother's viral load and giving antiretroviral therapy to the infant.

With the effective use of antiretrovirals, the risk of HIV transmission through breast feeding is minimised. Where the viral load of the mother cannot be suppressed the risk of breast milk transmission remains significant.

The PMTCT plan starts with the mother and may take one of the following routes once she is diagnosed as HIV infected:

1. Pregnant women with HIV infection receive combination antiretroviral therapy (cART) to suppress the HIV viral load to undetectable levels after which the risk of breast feeding transmission is negligible. cART may have been started before the woman became pregnant or may only be started during pregnancy as soon as the infection is detected.
   - » If the mother is started on cART early in pregnancy (i.e. > 4 weeks before delivery), and continues while breastfeeding then give antiretroviral prophylaxis (nevirapine (NVP)) to the infant until 6 weeks of age. If DNA PCR is positive in infant 6 weeks of age, start infant cART.
   - » If the mother is started on cART late during pregnancy (i.e. ≤ 4 weeks before delivery) or at delivery, then give antiretroviral prophylaxis (NVP) to the infant until 12 weeks of age. If DNA PCR is positive in infant 6 weeks of age, change infant prophylaxis to cART.
   - » Mothers who present late and have not received antiretrovirals:
     - o Initiate mother on cART, and give antiretroviral prophylaxis (NVP) to the infant until 12 weeks of age. If DNA PCR is positive in infant 6 weeks of age, change infant prophylaxis to cART.
     - o If the mother is unable to take cART (known renal disease or active psychiatric disorder) she should be referred for expert care and for the selection of an appropriate cART. If DNA PCR is positive in infant 6 weeks of age, change infant prophylaxis to cART.
   - » If the mother has been on cART for lifelong treatment before the pregnancy, she must have a viral load measurement at 1st booking.
     - o If the viral load is > 1000 copies/mL the cause could be problems with adherence, or viral resistance. Seek expert advice for the management of the mother and the child.
     - o If the viral load is < 1000 copies/mL and the mother is breastfeeding then give antiretroviral prophylaxis (NVP) to the infant until 6 weeks of age. If DNA PCR is positive in infant 6 weeks of age, change infant prophylaxis to cART.
2. Orphaned or abandoned infants with mother's HIV status unknown:
   - » See Infant Regimens in table below.

Perform DNA PCR testing on all HIV exposed infants at 6 weeks of age, and if negative, perform an ELISA/rapid test on HIV at 18 months of age.

Perform DNA PCR testing on all HIV-exposed low birth weight infants (< 2.5 kg) at birth and if positive, start cART. If negative, repeat DNA PCR at 6 weeks, and if negative again, perform an ELISA/rapid test on HIV at 18 months of age.

Perform DNA PCR testing at any other time if symptoms suggestive of HIV infection are present in a child not already known to be HIV infected, including prior to 6 weeks of age.

<table>
<tr><th colspan="3">Infant regimens</th></tr>
<tr><th>Infant</th><th>Regimen</th><th>Comment</th></tr>
<tr><td>Mother starting cART in pregnancy (including TDF + [3TC or FTC] + EFV)</td><td>Mother starts cART more than 4 weeks before delivery:
• NVP at birth and then daily for 6 weeks.

Mother starts cART less than 4 weeks before delivery, or at delivery:
• NVP at birth and then daily for 12 weeks.</td><td rowspan="4">If PCR is positive at any time including the 6 week test, stop NVP and urgently initiate, or refer the infant for cART.</td></tr>
<tr><td>Mother on lifelong cART and has started before pregnancy (including TDF + [3TC or FTC] + EFV)</td><td>• NVP at birth and then daily for 6 weeks –unless the mothers viral load is raised.

The viral load of mother should be done at booking if the viral load has not been shown to be fully suppressed within the last six months.
• If the viral load is not suppressed the mother (and child) must be managed by a practitioner experienced in ART and PMTCT.</td></tr>
<tr><td>Mother did not get any ART before or during delivery and tests HIV positive post delivery</td><td>Mother: initiate cART.

Baby: Give NVP immediately and then daily for 12 weeks.</td></tr>
<tr><td>Unknown maternal status because orphaned or abandoned</td><td>Baby: Give NVP immediately*.
Test infant with rapid HIV test.
- If positive, give NVP daily for 6 weeks. (Do infant HIV PCR at 6 weeks).
- If negative, discontinue NVP.</td></tr>
</table>

***** If rapid HIV test can be done within 2 hours, then wait for HIV result before commencing NVP.

Nevirapine (NVP) dose for infant on PMTCT

Daily NVP prophylaxis for 6 weeks or 12 weeks – see table above.

» Give 1st dose as soon as possible after birth.
» Only one dose per 24-hour period; repeat dose once only if baby vomits.
» If infant HIV PCR is positive at any time, stop NVP, confirm with 2nd DNA PCR test and initiate/refer for cART. Continue normal breastfeeding.

- Nevirapine, oral, daily. (Syrup 10 mg/mL)
  - o Newborns ≥ 2 kg and infants:

**Age**	**Weight:** mg of NVP	mL of NVP syrup 10 mg/mL
**Birth to 6 weeks:**	**2.0–2.5kg:** 10 mg	1 mL
	**> 2.5kg:** 15 mg	1.5 mL
**6 weeks to 6 months:**	20 mg	2 mL
If infant is severely underweight for age (3 SD or 3 z-scores below the mean) give NVP according to weight, (i.e. 4 mg/kg/dose daily) until in the normal weight for age range.		

  - o Premature newborn < 2 kg:

**Weight**	1st 2 weeks after birth mg of NVP	After 1st 2 weeks after birth mg of NVP
500 to < 625 g	1 mg	2 mg
625 to < 850 g	1.5 mg	3 mg
850 to < 1 200 g	2 mg	4 mg
1.2 to < 1.5 kg	3 mg	5 mg
1 5 to < 1.9 kg	3.5 mg	6 mg
If infant at time of discharge is severely underweight for age (3 SD or 3 z-scores below the mean) give NVP according to weight, (i.e. 4mg/kg/dose daily) until in the normal weight for age range.		

Feeding advice

» Exclusive breastfeeding is strongly recommended for the 1st 6 months, after which the nutritional requirements of the child will require the introduction of complementary foods.

» Except where a mother is shown to be failing cART, the advantages of breastfeeding exceed the risks of HIV transmission in a mother on cART and the mother should be encouraged to breast feed.
» The use of flash pasteurisation or Pretoria pasteurisation to reduce HIV transmission is supported but may pose significant barriers to successful breast milk feeding due to the effort involved.

Co-trimoxazole prophylaxis
Indications:
» All HIV exposed Infants starting from 4–6 weeks of age.
Discontinuation:
» If the child is shown to be HIV-uninfected **and** has not been breastfed for the last 6 weeks; **or**
» If HIV-infected, the immune system is fully reconstituted and > 1 year of age (i.e. child 1 to 5 years of age: CD4 > 25%, or child > 5 years of age: CD4 > 350 cells/mm^3 on 2 tests at least 3–6 months apart).

- Co-trimoxazole (sulfamethoxazole/trimethoprim), oral, once daily (everyday).

Recommended daily by weight band	Dose sulfamethoxazole/ trimethoprim	Suspension 200/40 mg per 5 mL	Single strength tablet 400/80 mg	Double strength tablet 800/160 mg
3 to 4.9 kg	100/20 mg	2.5 mL	¼ tablet	–
5 to 13.9 kg	200/40 mg	5 mL	½ tablet	–
14 to 29.9 kg	400/80 mg	10 mL	1 tablet	½ tablet
> 30 kg	800/160 mg	–	2 tablets	1 tablet

9.1.2 THE HIV INFECTED INFANT/CHILD
B20-24

DESCRIPTION
An infant or child in whom HIV infection has been confirmed with 2 appropriate tests.

For confirmation of HIV infection see section 9.1: Human immunodeficiency virus infections.

GENERAL AND SUPPORTIVE MEASURES
Counseling is a vital part of the successful care of children with HIV infection and their families. Specific matters requiring attention are:
» The implications of the disease to the family.
» Implications of treatment and understanding of the condition and its care.
» The disclosure process within the family and extended family should be encouraged. Besides the caregiver, help from the family is often useful.
» Disclosure to the child of appropriate age and maturity.

Treatment of mothers, caregivers and other family members:

» Always ask about the caregiver's health, and the health of other members of the family.
» Ensure that mothers and other family members have timeous access to medical care including cART.
» Encourage breastfeeding in all mothers with HIV infected children, with introduction of weaning foods from 6 months of age.
» Always ask at every visit about TB contacts and TB symptoms in all children and their caregivers.

STANDARDISED NATIONAL MONITORING FOR INFANTS AND CHILDREN WITH HIV

At initial diagnosis of HIV	Purpose
Verify HIV status	To ensure that national testing algorithm has been followed.
Document weight, height, head circumference (< 2 years of age) and development	To monitor growth and development.
Screen for TB symptoms	To identify TB and HIV co-infected.
Do CD4 count	Children < 5 years of age: Baseline. Do **not** wait for CD4 count to start ART.
	Children ≥ 5 years of age: To determine eligibility for ART.
Hb or FBC if available	To detect anaemia or neutropenia.
**At routine follow-up visits (patients not yet on ART)**	**Purpose**
Document weight, height, head circumference (< 2 years of age) and development.	To monitor growth and development.
Check that a CD4 count has been done in the last 6 months.	To determine if patient has become eligible for ART.
If > 5 years of age WHO clinical staging.	To determine if patient has become eligible for ART.
Screen for TB symptoms.	To identify TB/HIV co-infection.

At Initiation of ART (baseline)	Purpose
Hb or FBC	If less than 8 g/dL, manage appropriately.
CD4 count (if not performed in last 6 months).	Baseline assessment.
Cholesterol + triglyceride if starting PI-based regimen.	Baseline assessment.
If considering TDF-based regimen: creatinine + urine dipstick test.	If abnormal refer for specialist opinion.
ALT (if jaundiced or on TB treatment).	To assess for liver dysfunction at baseline.
**On ART**	**Purpose**
Height, weight, head circumference (< 2 years of age) and development.	To monitor growth and development stages.<br><br>Adjust dosing at each visit as necessary according to weight gain.
Clinical assessment including drug-related adverse events.	To monitor response to ART and exclude adverse effects.
CD4 count<br>1 year on ART, and then every 12 months thereafter.	To monitor response to ART, stop co-trimoxazole prophylaxis as indicated.
Viral load (VL)<br>< 5 years of age: at month 6, and month 12 on ART, then annually.<br><br>5 to 15 years of age: at month 6, then annually.	To monitor viral suppression response to ART.<br>To identify treatment failure and identify adherence problems.
Hb or FBC at month 1, 2, 3 and then annually if on AZT.	To identify AZT-related anaemia.
Cholesterol + triglyceride at 1 year of treatment and then every 12 months if on PI-based regimen.	To monitor for PI-related metabolic side effects.

MEDICINE TREATMENT

Co-trimoxazole prophylaxis

Indications:

» All HIV exposed Infants starting from 4–6 weeks of age.

Discontinuation:

» If the child is shown to be uninfected **and** has not been breastfed for the last 6 weeks; **or**

» If HIV-infected, the immune system is fully reconstituted on cART and child > 1 year of age (i.e. child 1 to 5 years of age: CD4 > 25%, or child > 5 years of age: CD4 > 350 cells/mm^3 on 2 tests at least 3–6 months apart).

- Co-trimoxazole (sulfamethoxazole/trimethoprim), oral, once daily (everyday).

Recommended daily dosage by weight band	Dose sulfamethoxazole/ trimethoprim	Suspension 200/40 mg per 5 mL	Single strength tablet 400/80 mg	Double strength tablet 800/160 mg
3 to 4.9 kg	100/20 mg	2.5 mL	¼ tablet	–
5 to 13.9 kg	200/40 mg	5 mL	½ tablet	–
14 to 29.9 kg	400/80 mg	10 mL	1 tablet	½ tablet
> 30 kg	800/160 mg	–	2 tablets	1 tablet

Immunisation, deworming and vitamin A program

Continue, deworming and vitamin A programme as in the HIV-negative child.
Continue immunisation as in the HIV-negative child except:

» Give an additional measles vaccination at 6 months of age.

» Do not give BCG to children with symptomatic HIV unless the child has been immune reconstituted on antiretroviral therapy.

See Immunisation in the Standard Treatment Guidelines and Essential Medicines List for Primary Health Care.

Nutritional support

Specific nutritional conditions should be treated appropriately.

Antiretroviral therapy (ART)

Initiation of ART in well uncomplicated infants shown to be PCR positive should be at PHC level – see national NIMART guidelines (IMCI) and Standard Treatment Guidelines and Essential Medicines List for Primary Health Care.

The preparation of the child and family to start ART is critical to the success of the treatment. Failure to achieve adherence and understanding may lead to resistance and adversely affect the prognosis of the child.

Eligibility for antiretroviral therapy

Clinical criteria

» Confirmation of diagnosis of HIV infection.

and

» Child < 5 years of age, irrespective of CD4 count or staging.
» Child ≥ 5 years with CD4 ≤ 350 cells/ mm^3 or WHO clinical stage III or IV.

and

» No medical contrai ndication (e.g. major o rgan dysfunction). If medical contraindications are present refer to hospital for rapid review and planning.

Children requiring fast track (i.e. to start ART within 7 days of being eligible with attention to social issues, counselling and adherence)

» Children < 1 year of age.
» WHO Clinical stage IV.
» MDR or XDR-TB infection.
» CD4 count < 200 cells/ mm^3 or < 15%.

Social issues that must be addressed to ensure successful treatment

These are extremely important for success and impact on adherence.
Social challenges should be overcome and not be barriers to care.
Disclosure to another adult living in the same house is encouraged so that there is someone else who can assist with the child's treatment.

» Mandatory component: at least one identifiable caregiver able to supervise the child and/or administer medication. All efforts should be made to ensure that the social circumstances of vulnerable children, e.g. orphans, be addressed to facilitate treatment.
» Adherence:
  > Very high levels of adherence (> 95%) should be attained for adequate virological response and prevention of viral resistance. This can be achieved with regular education and support.
  > All efforts to encourage this level of adherence should be made.
  > Viral load measurements are useful for monitoring adherence.
» Sensitive, age-appropriate disclosure may facilitate adherence.

Requirements before ART is used

The child's family (parents, caregivers) should understand:

» that antiretroviral therapy is lifelong;
» the prognosis of the condition (treated and untreated);
» adverse effects of the medicines, their mode of action, and the risk and implications of developing resistance, if incorrectly use;
» that all medications should be given as prescribed. If o ne or mo re antiretroviral is missing consultation with an expert is required.

ART Regimens

» Are chosen according to age, weight, expected adverse effects, efficacy and prior antiretroviral exposure.

» Adjust the dosage of antiretroviral therapy according to weight during follow up visits. Assess weight gain and need for adjustment at each visit.
» Do not change regimens or move to 2nd line therapy without clear guidance from an experienced practitioner in child ARV medicine as unnecessary loss of effective regimens can shorten life expectancy. Adherence problems need to be addressed thoroughly before switching to a 2nd or 3rd line regimen.
» Single drug substitution may only be made when drug-specific adverse effects are encountered, on condition that complete virological suppression is documented and the matter is discussed with an experienced practitioner in child ARV medicine first.

First Line Regimen	
All infants and children < 3 years of age (or < 10 kg)	ABC + 3TC + LPV/r<br>Note: Do **not** change regimen on reaching 3 years of age or 10 kg.
Children ≥ 3 years of age (and ≥ 10 kg)	ABC + 3TC + EFV. Do VL at 6 months on treatment to monitor viral suppression response to ART.
Currently on d4T-based regimen	Change d4T to ABC if the viral load is undetectable.<br>If detectable discuss with an experienced practitioner in child ARV medicine
**Second Line Regimen**	
**Failed first line protease inhibitor (PI)-based regimen**<br>(Consult with a specialist before changing)	
	**Recommended second line regimen**
ABC + 3TC + LPV/r	Consult with specialist for advice*<br><br><u>No previous daily NVP for PMTCT:</u><br>AZT + 3TC+ EFV* + LPV/r<br>* Use NVP if < 3 years of age or < 10 kg.<br><br><u>Previous daily NVP for PMTCT:</u><br>Refer for specialist management.
Previously on a regimen with <u>unboosted</u> PI (e.g. ritonavir alone), or with rifampicin while on LPV/r	Referral to a specialist for management.
**Failed first line NNRTI based regimen**<br>(Discuss with a specialist before changing)	
	**Recommended second line regimen**
ABC +3TC + EFV (or NVP)	AZT + 3TC +LPV/r
**Third line regimens**	
Failure of a 2nd line regimen	Refer to a specialist for further management.<br>Access to third line ART will be managed centrally by the National Department of Health.

General comments

Switch to tablets or capsules from syrups or solutions as soon as possible.
Use fixed dose combinations in preference to single agents.
If available, use daily dose regimens.

<table>
<tr><th>Weight
(kg)</th><th colspan="2">Abacavir
(ABC)</th><th colspan="2">Lamivudine
(3TC)</th><th>Efavirenz
(EFV)</th><th>Lopinavir/ritonavir
(LPV/r)</th></tr>
<tr><td>Target dose</td><td colspan="2">8 mg/kg 12 hourly
OR
≥10kg:
16 mg/kg once daily</td><td colspan="2">4 mg/kg 12 hourly
OR
≥10kg:
8 mg/kg once daily</td><td>By weight band
once daily</td><td>300/75mg/m^2/dose
LPV/r
12 hourly</td></tr>
<tr><td>Available formulations</td><td colspan="2">Sol. 20 mg/mL
Tabs 60 mg (scored, dispersible),
300 mg (not scored),
ABC/3TC 600/300mg</td><td colspan="2">Sol. 10 mg/mL
Tabs 150mg (scored),300mg;
ABC/3TC 600/300mg</td><td>Caps 50,200 mg
Tabs 50,200, 600 mg
(not scored)</td><td>Sol. 80/20 mg/mL
Adult Tabs 200/50 mg,
Paeds Tabs 100/25 mg</td></tr>
<tr><td colspan="7">Currently available tablet formulations of abacavir (except 60 mg), efavirenz, LPV/r and AZT must be swallowed whole and not chewed, divided or crushed.</td></tr>
<tr><td colspan="7">< 3 kg: Consult with a clinician experienced in paediatric ARV prescribing for neonates (< 28 days of age) and infants weighing < 3 kg.</td></tr>
<tr><td>3–4.9</td><td colspan="2">2 mL 12 hourly</td><td colspan="2">2 mL 12 hourly</td><td rowspan="3">Avoid using when
< 10 kg or
< 3 years:
dosing is not established</td><td>*1 mL 12 hourly</td></tr>
<tr><td>5–6.9</td><td colspan="2">3 mL 12 hourly</td><td colspan="2">3 mL 12 hourly</td><td rowspan="2">*1.5 mL 12 hourly</td></tr>
<tr><td>7–9.9</td><td colspan="2">4 mL 12 hourly</td><td colspan="2">4 mL 12 hourly</td></tr>
<tr><td></td><td colspan="2">Choose only one option</td><td colspan="2">Choose only one option</td><td></td><td></td></tr>
<tr><td>10–13.9</td><td>6 mL OR
2 x 60 mg tabs
12 hourly</td><td>12 mL
OR
4 x 60 mg
tabs daily</td><td>6 mL
12 hourly</td><td>12 mL daily</td><td>200 mg at night
(1x200 mg
cap/tab)</td><td>2 mL 12 hourly</td></tr>
</table>

<table>
<tr><th>Weight (kg)</th><th colspan="2">Abacavir (ABC)</th><th colspan="2">Lamivudine (3TC)</th><th>Efavirenz (EFV)</th><th>Lopinavir/ritonavir (LPV/r)</th></tr>
<tr><td></td><td colspan="2">Choose only one option</td><td colspan="2">Choose only one option</td><td></td><td>Choose one option</td></tr>
<tr><td>14–19.9</td><td>8 mL
12 hourly</td><td>1 300 mg tab
OR
15mL
daily</td><td>8 mL
OR
½x150 mg tab
12 hourly</td><td>1x150mg
tab
OR
15 mL daily</td><td>300mg at night:
(200mg cap/tab
+ 2x50mg cap/tab</td><td>2.5 mL
OR
2 x 100/25 mg paeds tabs
OR
1 x 200/50 mg adult tabs
12 hourly</td></tr>
<tr><td>20–24.9</td><td>10 mL
12 hourly</td><td>20 mL
daily</td><td>1x150 mg tab
OR
15 mL
12 hourly</td><td>30 mL
OR
1x300mg tab
OR
2x150mg tab
daily</td><td>300mg at night:
(200mg cap/tab
+ 2x50mg cap/tab</td><td>3 mL
OR
2 x 100/25 mg paeds tabs
OR
1 x 200/50 mg adult tabs
12 hourly</td></tr>
<tr><td>25–29.9</td><td rowspan="4">1x300mg tab
12 hourly</td><td rowspan="4">2x300mg
tabs daily

OR
1xABC/3TC
600/300 mg
tab
daily</td><td rowspan="4">1x150mg tab
12 hourly</td><td rowspan="4">2x150mg tabs

OR

1x300mg tab
daily

OR

1xABC/3TC
600/300 mg
tab
daily</td><td rowspan="3">400mg at night:
(2x200mg caps/tab)</td><td>3.5 mL
OR
3 x 100/25 mg paeds tabs
OR
#1 x 200/50 mg adult tabs
+ 1x100/25 mg paeds tab
12 hourly</td></tr>
<tr><td>30–34.9</td><td>4 mL
OR
3x100/25mg paeds tabs
OR
#1x200/50 mg adult tabs
+ 1x100/25 mg paeds tab
12 hourly</td></tr>
<tr><td>35–39.9</td><td rowspan="2">5 mL
OR
2x200/50 mg adult tabs
12 hourly</td></tr>
<tr><td>> 40</td><td>600mg tab at night</td></tr>
</table>

* Avoid LPV/r solution in any full term infant <14 days of age and any premature infant <14 days after their due date of delivery (40 weeks post conception) or obtain expert advice. [#]Children 25–34.9kg may also be dosed with LPV/r 200/50mg adult tabs: 2 tabs am; 1 tab pm

Weight (kg)	Ritonavir (r) boosting	Stavudine (d4T)	Nevirapine (NVP)	Zidovudine (AZT)
**Target dose**	**ONLY as booster for LPV/r when on rifampicin**<br>12 hourly<br>(0.75xLPV dose 12 hourly)	1 mg/kg/dose 12 hourly	160–200 mg/m^2 /dose 12 hourly (after once daily lead-in for 2 wks)	180–240mg/m^2/dose 12 hourly
**Available formulations**	Sol: 80mg/mL	Sol: 1mg/mL<br>Caps:15, 20, 30mg	Sol. 10mg/mL<br>Tabs 200mg(scored)	Sol. 10mg/mL<br>Caps 100mg<br>Tabs 300mg (not scored),<br>AZT/3TC 300/150mg
Currently available tablet formulations of AZT must be swallowed whole and **not** chewed, divided or crushed				
< 3 kg: Consult with a clinician experienced in paediatric ARV prescribing for neonates (< 28 days of age) and infants weighing < 3 kg				
3–4.9	1 mL 12 hourly	6 mL 12 hourly	5 mL12 hourly	6 mL12 hourly
5–5.9	1.5 mL 12 hourly	**7.5mg 12 hourly**: open 15mg cap into 5 mL water: give 2.5 mL		
6–6.9			8 mL 12 hourly	9 mL 12 hourly
7–7.9		**10mg 12 hourly**: open 20mg cap into 5 mL water: give 2.5 mL		
8–9.9				12 mL<br>**OR**<br>1 cap<br>12 hourly
10–10.9		**15mg 12 hourly**: open 15mg cap into 5 mL water: give 2.5 mL	10 mL 12 hourly	
11–13.9				
14–19.9	2 mL 12 hourly	**20mg 12 hourly**: open 20mg cap into 5 mL water (if child unable to swallow capsule)	15 mL 12 hourly<br>**OR**<br>1 tab morning<br>½ tab evening	15 mL 12 hourly<br>**OR**<br>2 cap morning<br>1 cap evening
20–24.9	2.5 mL 12 hourly			20 mL<br>**OR**<br>2 cap<br>12 hourly
25–34.9	3 mL 12 hourly	30mg 12 hourly	1 tab 12 hourly	1 tab 12 hourly<br>**OR**<br>1xAZT/3TC<br>300/150 mg tab 12 hourly
> 35	4 mL 12 hourly			

Specific information on ARVs		
	**Storage**	**Adverse effects**
**Nucleoside reverse transcriptase inhibitors (NRTIs)**		
Zidovudine (AZT)	room temperature	» haematological e.g. anaemia, neutropaenia
Stavudine (d4T)	refrigerate suspension	» lactic acidosis; peripheral neuropathy, lipoatrophy
Lamivudine (3TC)	room temperature	» uncommon
Abacavir (ABC)	room temperature	» Abacavir Hypersensitivity Reaction (HSR)<br>> usually occurs in 1st 6 weeks of initiation of therapy,<br>> symptoms and signs become worse with each subsequent dose,<br>> multi-system manifestations,<br>> fever, and rash common,<br>> other systems include gastrointestinal signs (nausea, vomiting, abdominal pain) and respiratory symptoms (dyspnoea, sore throat and cough).<br>» Laboratory abnormalities include raised transaminases and creatinine phosphokinase and lymphopaenia.<br>» **Do not continue or rechallenge with abacavir.**

Specific information on ARVs		
**Non-nucleoside reverse transcriptase inhibitors (NNRTIs)**		
	**Storage**	**Adverse effects**
Nevirapine (NVP)	room temperature	» skin rash usually occurs in 1st 6 weeks<br>Do not increase dosage until rash resolves.<br>» beware of liver toxicity
Efavirenz (EFV)		» give at night to avoid CNS side-effects:<br>> dysphoria > vivid dreams<br>> dizziness > distracted<br>» hepatotoxicity<br>For TB, maximise dose.<br>Possible teratogenicity
**Protease Inhibitors (PIs)**		
Ritonavir (r)		» bitter taste
Lopinavir/ritonavir (LPV/r)	Use tablets whole, without crushing, halving, biting or chewing	» nausea<br>» vomiting<br>» diarrhoea

Important side effects of ARVs

	**Continue ART with careful monitoring.**	**Stop treatment. Consult expert.**
Lactic acidosis	» lactate 2–5 mmol/L with no signs or symptoms	» lactate > 5 mmol/L, or<br>» with signs or symptoms or acidosis
Anaemia	» Hb: 7.0–9.9 g/dL	» Hb < 7g/dLor cardiac failure
Neutropaenia	» 0.4–1.2 x 10^9/L	» ≤0.399 x 10^9/L
Increase liver enzymes and hepatitis	» ≤ 9.9 x upper normal limit	» ≥ 10.0 x upper normal limit
Increased serum triglycerides	» 1.54–8.46 mmol/L	» ≥ 8.47mmol/L
Increased cholesterol	» 4.43–12.92 mmol/L	» ≥12.93 mmol/L
Severe skin reactions	» diffuse maculo- papular rash, or<br>» dry desquamation	» vesiculation, or<br>» ulcers, or<br>» exfoliative dermatitis, or<br>» Stevens-Johnson syndrome, or<br>» erythema multiforme, or<br>» moist desquamation, or<br>» with elevated ALT or AST
» peripheral neuropathy<br>» myopathy<br>» abdominal pain<br>» nausea and vomiting<br>» pancreatitis<br>» headache<br>» fatigue<br>» sedative effect<br>» sleep disturbance<br>» confusion<br>» abnormal thinking	clinical evaluation:<br>» Discuss all cases urgently with an HIV expert, before interrupting therapy	

Criteria for changing therapy

<u>Adverse effects</u>

Children may occasionally need to change a medicine from the first line regimen to one from the second line regimen because of intolerance or a serious adverse reaction. There is no need to change an entire regimen for a single adverse drug reaction.

Note: a single drug substitution <u>can only be made if</u> the viral load is undetectable or if the change is made in the first six months of starting a regimen.

The decision to swap must be made by a doctor with antiretroviral experience (this can be by telephonic consultation), as inappropriate choices of antiretrovirals may be ineffective or dangerous.

<u>Treatment failure</u>

The HIV viral load is the most sensitive method to detect failure of response to cART.

Virological failure can be defined as a measurable viral load despite optimal adherence and optimal dosage over a four month period. Treatment failure is defined primarily by viral loads, as waiting for clinical or immunological failure enhances the chances of increasing viral resistance to other available anti-retroviral agents.

The most common cause of failure of first (and subsequent) line therapy is poor adherence. There is no point in changing to second line therapy before adherence has been addressed.

Viral load (VL)	Response
Lower than detectable limits	» Praise the patient and caregiver(s) and continue 12 monthly VL monitoring.
< 400 copies/mL	» 12 monthly VL monitoring and adherence support
400–1 000 copies/mL	» Begin step up adherence package.<br>» Repeat VL in 6 months.
>1 000 copies/mL	» Begin step-up adherence package.<br>» Repeat VL in 3 months:<br>> If < 400: return to routine 6–12 monthly monitoring.<br>> If 400–1 000: continue step up adherence and repeat VL after 6 months.<br>> If > 1 000 despite stepped up adherence, **and** child on NNRTI based regimen: switch to second line therapy after adherence ensured.<br>> If the child is on a PI-based regimen and the VL is > 1 000 despite stepped up adherence:<br>▪ Where the HIV VL < 30 000 continue with same regimen while monitoring VL 3-monthly. Continue stepping up adherence and consult an expert.<br>▪ If the HIV VL is > 30 000 this requires referral to an expert for further management.

REFERRAL

» Complicated or very ill children should be referred to a practitioner skilled in the care of such children.
» Attempts should be made to refer patients to accredited primary health care sites once stable on ART.

9.2 TUBERCULOSIS AND HIV

B20.0

DESCRIPTION

TB and HIV are often co-morbid conditions. Exclude TB by clinical examination, chest X-ray, tuberculin skin test (TST), *M. tuberculosis* PCR test and mycobacterial culture (where TB disease is suspected on clinical or radiological grounds) in all patients before starting ART.

Re-evaluate the risk for TB and TB contact at each visit on history (including contact history) and clinical examination.

MEDICINE TREATMENT

TB prophylaxis

Give TB prophylaxis to all children exposed to a close contact with an infectious pulmonary TB case (sputum microscopy smear-positive, culture-positive or *M. tuberculosis* PCR test positive), or who are TST positive, **but** in whom no evidence of TB disease is present.

- Isoniazid, oral, 10 mg/kg/dose once daily for 6 months.
  - o Maximum dose: 300 mg daily.

Repeat course if an HIV-infected patient, irrespective of age, is re-exposed to a TB contact at any point after completing TB treatment or prophylaxis.

If patient has been exposed to a known MDR or XDR-TB source case or the contact case has failed standard TB treatment, refer for expert opinion. See section 15.1.4: Tuberculosis, pulmonary.

TB treatment

<u>If the child is not yet on ART:</u>

» Commence TB treatment first. Follow with cART, usually after 2–8 weeks.
  > After 2 weeks if CD 4 < 50 cells/mm^3.
  > After 8 weeks if CD 4 > 50 cells/mm^3.
» Check ALT before commencing cART. If the ALT is raised discuss this with an expert as it may not be an absolute contraindication to treatment.
» Be aware of the possibility of Immune Reconstitution Inflammatory Syndrome (IRIS).

If the child is already on ART:

» Commence TB treatment taking into consideration possible drug interactions.

If the child needs to take concomitant ART and rifampicin-containing treatment:

» Efavirenz: use the maximum dosage according to recommended range as per package insert of either drug for weight or body surface area.
» Abacavir and lamivudine: no adjustment of dosages.
» Lopinavir/ritonavir: provide additional ritonavir while on rifampicin-containing treatment (add sufficient ritonavir to ensure an equal dose in milligrams of lopinavir and ritonavir). For example for each mL of lopinavir/ritonavir solution (80/20 mg/mL) add 0.75 mL of ritonavir solution (80 mg/mL).
» Give pyridoxine (vitamin B_6) to all children on TB and ARV treatment, due to shared toxicities of the regimens.

9.3 SPECIFIC ADVERSE EVENTS AND COMPLICATIONS

9.3.1 LACTIC ACIDOSIS

E87.2

DESCRIPTION

All nucleoside analogues have been associated with lactic acidosis which is rare but life threatening. Initial symptoms are variable and usually occur between 1–20 months (median 4 months) after starting therapy. The propensity to develop lactic acidosis is highest with stavudine, followed by didanosine and then zidovudine.

DIAGNOSTIC CRITERIA

Clinical

Clinical prodromal syndrome:

» Generalised fatigue.
» Weakness and myalgia.
» Gastrointestinal symptoms:
  > nausea,
  > vomiting,
  > diarrhoea,
  > vague abdominal pain,
  > hepatomegaly,
  > anorexia,
  > and/or sudden unexplained weight loss.
» Respiratory symptoms: tachypnoea and dyspnoea.
» Neurologic symptoms, including motor weakness.

Investigations

» Laboratory abnormalities:
  > Hyperlactataemia:
    chronic, asymptomatic: 2.1–5 mmol/L.
    symptomatic severe: > 5 mmol/L.
  > Exclude other causes of hyperlactataemia/acidosis.
  > Lactic acidosis is defined by:
    lactate > 5 mmol/L,
    bicarbonate < 20 mmol/L,
    severe acidosis, i.e. pH < 7.3,
    increased anion gap,
    associated symptoms,
    precipitating events: (manage each appropriately)
    - dehydration,
    - sepsis,
    - cardiac failure,
    - severe anaemia.

TREATMENT

Lactate 2.1–5.0 mmol/L confirmed and asymptomatic

Consider replacing stavudine and didanosine if used with other ARVs.
Consider temporary discontinuation all ART while assessing. (When stopping ART, if r egimen contained nevirapine or efavirenz, use lopinavir/ritonavir (300 mg/m^2) for 2 weeks to cover the prolonged half life of NNRTIs).

Lactate > 5.0mmol/L confirmed, or a single test > 10mm0l/L

Stop all ARVs. (When stopping ART, if reg imen contained nevirapine or efavirenz, use lopinavir/ritonavir (300 mg/m^2) for 2 w eeks to cover the prolonged half life of NNRTI's, while getting expert advice.)
Provide supportive care, i.e. oxygen (if needed) and IV fluids.

In patient with symptomatic hyperlactataemia and/or a blood lactate of > 5 mmol/L:

» Stop all ARVs. (When stopping ART, if regimen contained nevirapine or efavirenz, use lopinavir/ritonavir (300 mg/m^2) for 2 weeks to cover the prolonged half life of NNRTIs).
» Consult a specialist for guidance.
» Treatment is primarily supportive.
» Ensure adequate fluid therapy.
» Consider use of bicarbonate to half correct acidosis if severe metabolic acidosis.
» Consider use of:
- Thiamine, oral, 100 mg daily.

REFERRAL

- » For adjustment of regimen.
- » For care if beyond capacity of local institution.

9.3.2 LIPODYSTROPHY

E88.1

DESCRIPTION

Both lipoatrophy and lipohypertrophy can occur as a complication or association of cART. The risk factors include virologic response to therapy and pubertal development during protease inhibitor therapy.

Lipodystrophy contributes to non-adherence to ART as a patient may be embarrassed by his/her physical appearance.

Stavudine, didanosine and zidovudine in decreasing order are the main causes of lipoatrophy.

The relationship between lipohypertrophy, hypercholesterolaemia, hypertriglyceridaemia, insulin resistance with puberty, body habitus and ART (especially protease inhibitors), is less clear but an association has been described.

DIAGNOSTIC CRITERIA

- » Lipoatrophy:
  - > Subcutaneous fat loss (lipoatrophy) of the face, extremities or buttocks.
- » Lipohypertrophy:
  - > Fat accumulation (lipohypertrophy) in the abdomen, or over the dorsocervical spine (buffalo hump) and breast enlargement.
  - > Excessive breast enlargement during puberty (lipomastia).
- » Insulin resistance may be suspected if there is:
  - > fasting hyperglycaemia,
  - > frank diabetes or acanthosis nigricans,
  - > biochemical features include an elevated fasting C-peptide or an abnormal glucose/insulin ratio.
- » Abnormal lipid profile: See section 4.10: Dyslipidaemia.
  - > hypercholesterolaemia, i.e. total cholesterol level > 5 mmol/L; and
  - > hypertriglyceridaemia, i.e. fasting triglyceride level > 1.7 mmol/L with possible consequences of premature atherosclerosis.

GENERAL AND SUPPORTIVE MEASURES

- » Dietary modification and exercise for lipohypertrophy, insulin resistance and abnormal lipid profile.

MEDICINE TREATMENT

Modification of ART, e.g. replace stavudine with abacavir in lipoatrophy.

Note:
Viral suppression must be present for a single drug substitution. If viral suppression is not present obtain expert advice.

If hyperlipidaemia is confirmed, use lipid lowering agents.

REFERRAL

» All patients for confirmation of diagnosis and initiation of therapy.

See section 7.16: Obesity.

9.3.3 IMMUNE RECONSTITUTION INFLAMMATORY SYNDROME (IRIS)

D89.3

DESCRIPTION

Clinical deterioration can occur after starting ART due an improvement in the immune system response to organisms already causing infection, e.g.

- *M bovis BCG,*
- *M. tuberculosis (MTB),*
- *M. avium complex,*
- *M. leprae,*
- *P. jiroveci,*
- CMV,
- JC virus.
- *C. neoformans,*
- Aspergillus,
- *C. albicans,*
- Human Herpes viruses,
- Human Papilloma virus,
- Hepatitis B and C viruses (HBV, HCV),

There are 2 manifestations of IRIS:

1. Unmasking occurs when a previously unsuspected condition manifests.
2. Paradoxical, i.e. known condition on appropriate treatment becomes worse.

DIAGNOSTIC CRITERIA

» Exclude other active or inadequately treated diseases (including MDR TB).

» Presentation:
  - > Usually during the first 6 weeks after starting ART.
  - > Clinical presentation depends on the causative organism and the organ-system involved, e.g. TB presents with fever, lymphadenopathy, worsening of the original tuberculous lesion, and/or deteriorating chest radiographic manifestations such as miliary pattern or pleural effusion.

TREATMENT
Treat underlying disease aggressively.

Antimicrobial therapy for specific infections.

In severe reactions:
- Hydrocortisone, IV, 3–6 mg/kg/dose 3–6 hourly on first day.

Follow with:
- Prednisone, oral, 1–2 mg/kg/dose 24 hourly for at least 7 days.

Continue cART, and manage underlying condition.

9.3.4 WASTING SYNDROME
B22.2

This syndrome appears to be a combination of the direct effects of advanced HIV infection and the occurrence of opportunistic infections.

TREATMENT
Nutritional advice. See section 2.4: Malnutrition.
cART may reverse some of the features of HIV wasting syndrome.
Exclude chronic infection, e.g. tuberculosis and *M. avium complex*, malabsorption and malignancy.

9.4 POST EXPOSURE PROPHYLAXIS FOLLOWING ALLEGED PENETRATIVE SEXUAL ABUSE

See Standard Treatment Guidelines and Essential Medicines List for Primary Health Care.

9.5 HIV IN ADOLESCENCE
B20-24

DESCRIPTION
Adolescence encompasses the period of physical and psychological development from the onset of puberty to maturity. HIV in adolescents may be due to:
1. Vertical infection in infancy that presents as long term non-progressors; or
2. Sexually acquired HIV from unprotected intercourse.

Increasing numbers of perinatally infected infants are surviving to adolescence.

Adolescence is a high risk period for non-adherence to therapy.
Mood disorders, denial, peer pressure, self-esteem and suicide risk are more common and patients may need to be referred for psychological support.

Education about sexual and reproductive health should be commenced early. Every encounter with the adolescent needs to be maximally utilised to discuss condom and contraception use to protect against unplanned pregnancies and STI transmission including HIV is essential. Schools should be taking an active role in this education. Sexually active youth need to be screened for STI symptoms and managed appropriately.

Consent
For testing and treatment, the current acts and regulations should be followed.

Disclosure
All adolescents need to be aware of their HIV status. This should be handled sensitively. In addition, disclosure of diagnosis has ramifications for adherence. Disclosure should be planned with the caregiver and usually takes place over 2–3 visits. Intervention by a social worker is useful. Determine what the adolescent already knows and discuss with the caregiver about who should disclose and where.

Dosage of ARVs
In children over the age of 15 years and over 40 kg use adult dosage regimes – consult ART guideline. Medicines are not routinely switched based on age.

Contraception in HIV-positive adolescents on ART

Hormonal contraceptives and IUCDs do not prevent sexually transmitted infections.<br>Additional use of condoms is required.

- IUCD

HIV is not a contraindication to IUCD use and may be used in adolescents on ART.

- 380mm^2 copper – standard type.

- Progestogen-only subdermal implant contraceptive e.g.:
- Levonorgestrel, 150mg, subdermal two-rod implant.

- Injectable contraception: e.g.
- Medroxyprogesterone acetate (long-acting), IM, 150 mg, 12 weekly.
  Note:
  It is unnecessary to shorten the dosage interval for women taking concomitant enzyme-inducing drugs, e.g. rifampicin, antiretrovirals and anticonvulsants.

Combined oral contraceptives (COCs) are indicated for motivated patients where adherence is more likely but are associated with drug-drug interactions.

CHAPTER 10

SURGICAL PROPHYLAXIS

DESCRIPTION

Surgical prophylaxis is the intra-operative administration of antibiotics to patients to reduce the risk of postoperative wound infection.
Specific epidemiological considerations may alter the choice of agents.

PRINCIPLES OF SURGICAL PROPHYLAXIS

» The need for prophylactic antibiotic therapy is based on the risk of wound contamination.
» The medication chosen should be active against the pathogens most likely to be associated with wound infections.
» Prophylaxis must be given within 30 minutes of the first incision, usually at induction of anaesthesia.

Risk factors for developing surgical site infection
Classification of degree of contamination likely to be present during operation:
» Class I: Clean procedures, only microorganisms from skin or external environment are likely to be introduced (includes operation for blunt trauma).
» Class II: Clean procedures with limited contamination, exposure to micro-organisms colonising the epithelial surfaces and/or lumen of respiratory, gastrointestinal, urinary or genital tract. No evidence of infection.
» Class III: Contaminated, open fresh accidental wounds, operations with major breaks (e.g. open cardiac massage or gross spillage from gastrointestinal tract) and incisions in which non-purulent inflammation is encountered.
» Class IV: Dirty and/or infected surgical site indicates that the organism causing postoperative infection was in the operation area before surgery, traumatic wounds with devitalised tissue not immediately attended to, and wounds that involve existing clinical infection or perforated viscera.

These guidelines cover prophylaxis and not therapy for infective conditions.
Other risk factors are:
» Prolonged duration of operation.
» Medical characteristics of the patient (nutritional status, immunosuppression and co-existent infection at remote body site).
Consider antibiotic prophylaxis for class II and if these other risk factors are present.

Recommendations for prophylaxis of surgery are limited to class II. For class III and IV, antibiotics are indicated for therapy rather than single dose prophylaxis.
Additional procedures for which antibiotic prophylaxis is required include the following:

» Head and neck: CSF shunt and grommet insertion.
» Cardiothoracic: cardiac pacemaker insertion, interventional cardiac catheter device placement.
» Gastrointestinal: insertion of percutaneous endoscopic gastrostomy.

The prophylactic dose is a single dose equal to the standard therapeutic dose.
A second dose is **ONLY** given if surgery is prolonged,
i.e. > 4 hours for cefazolin **OR** > 8 hours for metronidazole.

ANTIBIOTIC TREATMENT

- Cefazolin, IV, 25 mg/kg, 30 minutes before the procedure.
  - o Maximum dose: 1 000 mg.

AND

For lower limb am putation, colorectal, appendix, biliary, and pelv ic or oropharyngeal mucosa surgery:

- Metronidazole, IV, 7.5 mg/kg.

For eye surgery:

- Chloramphenicol ophthalmic drops, instil in the affected eye.

For ventilation tube insertion:

- Ofloxacin, ophthalmic drops, instil 1 drop, in t he affected ear. (Single dose topical antibiotic)

CHAPTER 11
MUSCULOSKELETAL SYSTEM

11.1 ARTHRITIS, SEPTIC (PYOGENIC)

M00.9

DESCRIPTION

Septic arthritis may occur as a result of haematogenous seeding of the synovium during transient periods of bacteraemia.
Septic or pyogenic arthritis is often part of a generalised septicaemia which may involve more than one joint and is caused by pyogenic micro-organisms. The organisms involved vary:

- » Neonates – *S. aureus*, Group B. Streptococci, *E. coli*, fungi.
- » Infants/children – *S aureus*, *H. influenzae*, Group A Streptococci, *S. pneumoniae, Kingella kingae.*
- » Adolescents (sexually active) – *N. gonorrhoea.*
- » Chronic septic arthritis – *Brucella*, tuberculosis, atypical mycobacteria, fungi and other uncommon organisms.

DIAGNOSTIC CRITERIA

The diagnosis is largely clinical and confirmed by finding pus in the joint space.

CAUTION
Do not carry out needle aspiration in haemophiliacs.

Clinical

- » Fever, local pain, loss of function and toxic looking child.
- » Subtle, non-specific signs of sepsis early in the course of the disease, especially in neonates.
- » Local tenderness, warmth, swelling at a joint with restriction of passive and active movement.
- » Malaise, irritability, feeding problems and pseudo-paralysis.
- » If lower extremities are involved, development of a limp or refusal to bear weight.

Investigations

- » Aspiration of pus from the joint space under ultrasound guidance, if possible.
- » Blood and aspirated pus for microscopy, Gram stain, culture and sensitivity.
- » Raised white-cell count and ESR and/or CRP.

GENERAL AND SUPPORTIVE MEASURES

» Septic arthritis of the hip (emergency) requires prompt open surgical drainage at the time of presentation, in consultation with an orthopaedic surgeon.
» Manage most infections of other sites by repeated aspiration or open drainage (not antibiotic instillation), if frank pus is obtained on initial diagnostic aspiration.
» Immobilise affected limb in position of function.
» Identify other effects of septicaemia or haematogenous spread and treat appropriately.
» Supportive and symptomatic care.

MEDICINE TREATMENT

Antibiotic therapy

Minimum duration of therapy: 4–6 weeks.

<u>IV antibiotics</u>

Initiate IV antibiotic treatment immediately.

Adjust antibiotic therapy based on culture results or if response to empiric antibiotic treatment is unsatisfactory.

Continue with IV antibiotics until there is evidence of good clinical response and laboratory markers of infection improve. Continue antibiotic therapy orally.

Neonates:

- Cloxacillin, IV, 50 mg/kg/dose.
  - If 1st week of life: 12 hourly.
  - If 2nd–4th week of life: 8 hourly.
  - If >4 weeks old: 6 hourly.

PLUS

- Cefotaxime, IV, 50 mg/kg/dose.
  - Preterm: 12 hourly.
  - If 1st week of life: 8 hourly.
  - If > 2 weeks old: 6 hourly.

Infants and children:

- Cloxacillin, IV, 50 mg/kg/dose 6 hourly.

PLUS

- Ceftriaxone, IV, 80 mg/kg/dose 12 hourly.

Special Circumstances

If MRSA is suspected, replace cloxacillin with vancomycin.

- Vancomycin IV, 15 mg/kg/dose administered over 1 hour given 6 hourly.

Adjust antibiotics once sensitivity results are obtained.

Oral antibiotics
Do weekly CRPs.
Continue with oral antibiotics until there are no signs of infection and white cell count/CRP are back to normal.

Antibiotics according to sensitivities:
- Clindamycin, oral, 6 mg/kg/dose 6 hourly.

OR

For children able to swallow a capsule:
- Flucloxacillin, oral, 12.5–25 mg/kg/dose, 6 hourly.

For pain and inflammation:
- Ibuprofen, oral, 5–10 mg/kg/dose, 6 hourly.

REFERRAL
» Multi-organ involvement.
» Failure to achieve progressive improvement on treatment.

11.2 ARTHRITIS, JUVENILE IDIOPATHIC
M08.0

See section 12.2: Juvenile idiopathic arthritis (JIA).

11.3 OSTEITIS/OSTEOMYELITIS, ACUTE
M86.1

DESCRIPTION
Most cases result from haematogenous deposition of organisms in the bone marrow after a transient bacteraemic episode. Osteomyelitis most commonly begins in the metaphyses of long bones which are highly vascular. The spread of infection through the epiphysis can result in septic arthritis. The organisms involved vary:
» Neonates: *S. aureus*, Group B. Streptococci, Gram negative (*E. coli*).
» Infants/children: *S. aureus*, *H. influenzae*, Group A Streptococci, *S. pneumoniae.*
» Traumatic direct infection: *P. aeruginosa* (penetrating foot wounds)
» Co-existing medical conditions e.g. diabetes, HIV, leucopaenia: *M. tuberculosis*, fungi.
» Sickle cell disease: Salmonella, pneumococcus.

DIAGNOSTIC CRITERIA

Clinical

- » Local pain and tenderness, loss of function, general toxicity and fever.
- » If lower extremities are involved (development of a limp or refusal to bear weight).
- » In neonates, early signs may be subtle or non-specific, e.g. irritability, feeding problems and pseudoparalysis.
- » Investigate for multi-organ disease, e.g. endocarditis, pericarditis and pneumonia.

Investigations

<u>Diagnostic</u>

- » Aspiration of pus for microscopy, Gram stain, culture and sensitivity.
- » Blood culture and full blood count.
- » Raised white-cell count, CRP.

The following may be helpful:

- » X-ray after 2 weeks.
- » Bone scan (Tc99*).
- » MRI.

GENERAL AND SUPPORTIVE MEASURES

- » Surgical drainage if:
  - > frank pus is aspirated from bone,
  - > clear evidence of progression to soft tissues,
  - > when a marked improvement has not occurred within 24–36 hours on adequate IV antibiotic treatment,
  - > coexisting septic arthritis.
- » Immobilise affected limb in position of function.
- » Supportive and symptomatic care.

MEDICINE TREATMENT

Antibiotic therapy

Minimum duration of therapy: 4–6 weeks.

<u>IV antibiotics</u>

Initiate IV antibiotic treatment immediately as diagnosis is made and blood and pus specimens have been collected.

Adjust antibiotic therapy based on culture results or if response to antibiotic treatment is unsatisfactory.

Where a single agent has been found to be sensitive, continue treatment on that single agent.

Continue with IV antibiotics until there is evidence of good clinical response and laboratory markers of infection improve (usually about 2 weeks). Oral antibiotics may then be considered.

Ongoing fever suggests an undrained focus of pus.

Neonates:

- Cloxacillin, IV, 50 mg/kg/dose.
  - If 1st week of life: 12 hourly.
  - If 2nd–4th week of life: 8 hourly.
  - If > 4 weeks old: 6 hourly.

PLUS

- Cefotaxime, IV, 50 mg/kg/dose.
  - Preterm: 12 hourly.
  - If 1st week of life: 8 hourly.
  - If > 2 weeks old: 6 hourly.

Infants and children:

- Cloxacillin, IV, 50 mg/kg/dose 6 hourly.

PLUS

- Ceftriaxone, IV, 50 mg/kg/dose 12 hourly.

Special Circumstances

If MRSA, replace cloxacillin with vancomycin.

- Vancomycin IV, 15 mg/kg/dose administered over 1 hour given 6 hourly.

Penetrating foot bone injuries: replace cefotaxime with ceftazidime plus aminoglycoside:

- Ceftazidime, IV, 50 mg/kg/dose 6 hourly.

PLUS

- Gentamicin, IV, 6 mg/kg once daily.

<u>Oral antibiotics</u>

Do weekly CRPs.

Continue with oral ant ibiotics until no s igns of inf ection and w hite cell count/ESR/CRP are back to normal.

Antibiotics according to sensitivities:

- Clindamycin, oral, 6 mg/kg/dose 6 hourly.

OR

For children able to swallow a capsule:

- Flucloxacillin, oral, 25 mg/kg/dose, 6 hourly.

<u>For pain and inflammation</u>:

- Ibuprofen, oral, 5–10 mg/kg/dose, 6 hourly.

REFERRAL

» Multi-organ involvement.

» Failure to achieve progressive improvement on treatment.

CHAPTER 12

CONNECTIVE TISSUE DISORDERS

12.1 HENOCH SCHÖNLEIN PURPURA (HSP)

D69.0

DESCRIPTION

Henoch Schönlein Purpura (HSP) is an acute leukoclastic vasculitis of small blood vessels usually involving skin, gastrointestinal tract, joints and the kidney. Aetiology is unknown.

Complications include:

- » acute severe abdominal pain, bowel infarction;
- » nephritis with renal impairment or nephrotic syndrome;
- » CNS involvement.

DIAGNOSTIC CRITERIA

Clinical

Syndrome consisting of:

- » Non-thrombocytopenic purpuric skin rash with a very typical distribution on lower extremities and buttocks. Trunk and upper extremities may be involved. It begins as a wheal or erythematous macule/papule, which develops into red-purple palpable purpura.
- » Arthralgia/arthritis in 60–70% of cases: mostly of large joints, i.e. knees and ankles.
- » Abdominal pain with colic: may develop haematemesis or intussusception or infarction.
- » Renal involvement in 25–50% manifesting with haematuria or proteinuria.
- » Angio-oedema of scalp, eyelids, lips and ears.
- » Rarely CNS involvement: seizures, paresis or coma.

Investigations

- » No diagnostic test.
- » FBC is usually normal but necessary to rule out other conditions with thrombocytopaenic purpura.
- » Coagulation studies are normal.
- » Urine dipstick to evaluate renal involvement. Serum urea, creatinine, electrolytes and albumin with renal involvement.
- » Check stools for occult or frank bleeding.

GENERAL AND SUPPORTIVE MEASURES

- » Short period of immobilisation during acute arthritis.
- » Soft diet for acute gastrointestinal involvement.

MEDICINE TREATMENT

For arthritis, oedema, fever, malaise:

- Ibuprofen, oral, 10 mg/kg/dose 6 hourly.
    - o Reduce dose interval to 8 hourly after pain is managed.

For complicated HSP (severe extrarenal symptoms or renal disease):

- Prednisone, oral, 1–2 mg/kg/dose once daily for 10 days in the morning.
    - o Reduce dose gradually over 2 weeks.

REFERRAL

HSP with complications, i.e. in patients with:

» persistent proteinuria, or
» persistent macroscopic haematuria for kidney biopsy to plan immunosuppressive treatment, or
» persistent abdominal pain, or
» progressive glomerulonephritis, or
» CNS involvement.

12.2 JUVENILE IDIOPATHIC ARTHRITIS (JIA)

M08.0

DESCRIPTION

Juvenile Idiopathic Arthritis (JIA) is defined as an arthritis of unknown origin for at least 6 weeks with onset before the age of 16 years. Other causes of arthritis must be excluded e.g. infections, malignancy, trauma, other autoimmune disease. Different clinical subgroups are recognised according to the pattern of on set that manifests within the first 6 months.

DIAGNOSTIC CRITERIA

Systemic onset

» Arthritis in one or more joints.
» Plus 2 weeks of daily (quotidian) fever.
» With one of the following:
  > erythematous macular rash, or
  > serositis, i.e. pericarditis and pleuritis, or
  > hepatosplenomegaly, or
  > generalised lymphadenopathy.

Oligoarthritis

» Arthritis affecting one to four joints for first 6 months of disease.
» Two categories are recognised:
  > Persistent oligoarthritis: affects ≤ 4 joints throughout disease course.
  > Extended oligoarthritis: affects > 4 joints after the first 6 months.
» Occurs more commonly in girls than in boys.

» Has early onset before 6 years of age.
» Usually asymmetric arthritis that affects mainly large joints.
» High risk of developing chronic iridocyclitis.
» 65–85% of patients are ANA positive.

Polyarthritis (Rheumatoid factor negative)
» Arthritis affecting ≥ 5 joints in first 6 months of disease.
» Negative rheumatoid factor polyarthritis includes 2 subsets:
  > one that is similar to adult onset RF negative rheumatoid arthritis characterised by a symmetric synovitis of large and small joints, onset at school age and absence of ANA expression;
  > another that resembles oligoarthritis apart from the number of joints affected in the first 6 months of the disease.

Polyarthritis (Rheumatoid factor positive)
» Arthritis affecting ≥ 5 joints in first 6 months.
» Positive rheumatoid factor on 2 separate occasions at least three months apart.
» Involves large and small joints.

Enthesitis related arthritis
» Arthritis and enthesitis **or,**
» arthritis or enthesitis **and** 2 of the following:
  > sacroiliac joint involvement,
  > HLA-B27 positive,
  > one 1st or 2nd degree relative with HLA-B27 associated disease,
  > arthritis in a boy after the age of 8 years,
  > anterior uveitis associated with pain, redness or photophobia.

Psoriatic Arthritis
» Arthritis plus psoriasis in a child, **or**
» Arthritis **and** 2 of the following:
  > dactylitis,
  > nail pitting,
  > psoriasis in a first degree relative.

Undifferentiated arthritis
» Arthritis not meeting criteria for one of the above categories or fitting more than one of the above groups.

Differential diagnosis
JIA is a clinical diagnosis and depends on the persistence of arthritis or typical systemic manifestations and by exclusion of other diseases:
» Pyogenic and tuberculous joint infection and osteomyelitis.
» Arthritis associated with other acute infectious illnesses.
» Acute leukaemia and other malignancies.
» Acute rheumatic fever.
» Auto immune disorders, SLE or mixed connective tissue disease.
» Reiter syndrome, i.e. arthritis, urethritis and conjunctivitis.
» Arthritis associated with primary inflammatory bowel disease.

Investigations

- » There is no single diagnostic test.
- » Full blood count: differential and platelet count.
- » C-reactive protein and erythrocyte sedimentation rate.
- » ALT for liver function screen before starting methotrexate.
- » Serum urea, creatinine and electrolytes.
- » Muscle enzymes, albumin, calcium, phosphate and alkaline phosphatase.
- » Auto-antibodies, complement C_3 and C_4, rheumatoid factor, IgG and IgA levels.
- » X–ray or ultrasound of affected joints.
- » Arthroscopy, synovial biopsies, ultrasound and CT scan or MRI, where necessary.
- » Eye screen for uveitis.

GENERAL AND SUPPORTIVE MEASURES

- » Occupational and physiotherapy programs may provide the following:
  - > exercises to increase range of movements of joints and to maintain muscle strength;
  - > hot water baths, swimming pool exercises;
  - > splints, e.g. nocturnal splints, for pain relief and prevention of contractures;
  - > shoe inserts/raises;
  - > aids for activities of daily living.
- » Orthodontic treatment if temporomandibular joints are involved.
- » Dental care to prevent periodontal disease.
- » Slit lamp examination 3–4 times yearly for at least the first 5 years of disease and depending on disease activity.

MEDICINE TREATMENT

There is no cure for JIA.
Goal of treatment is to eliminate active disease, to normalise joint function, to preserve normal growth, to prevent long term joint damage and disease complications. Outcome is improved with early aggressive therapy.

Oligoarthritis

NSAID, e.g.:

- Ibuprofen, oral, 10 mg/kg/dose 4–6 hourly.

Efficacy is determined within weeks unless there is aggressive progression or severe adverse effects, i.e. gastric irritation, peptic ulcer, hepatic toxicity, renal impairment or platelet dysfunction.

As soon as diagnosis confirmed:

- ▪ Intra-articular corticosteroid injection (Rheumatologist or orthopaedic specialist):
- Methylprednisolone acetate, 1 mg/kg with lignocaine 1%, 0.5 mL 1, if only 1–2 joints.
  - o If no response: repeat in 3 months.
  - o Young children may require light sedation with midazolam and ketamine.
  - o Large joints, if possible, should be aspirated at same time.

If no response:
ADD
- Prednisone, oral, 2 mg/kg as a single daily dose for 2 weeks. Specialist initiated.
    - o Reduce dose gradually over 4 weeks.

If not controlled after 3 months:
ADD
- Methotrexate, oral, 10-15 mg/m^2/week as a single dose on an empty stomach. Specialist initiated.
    - o Increase dose at monthly intervals up to 1 mg/kg/week until there is satisfactory response, continue maintenance at the same dose.
    - o Maximum dose: 25 mg/week.
    - o Adverse effects include: nausea, mood changes, raised liver enzymes, bone marrow toxicity and protein/haematuria. Monitor FBC, LFT, U&E and urine test strips monthly.

PLUS
- Folic acid, oral, 5 mg daily for the duration of the treatment.

If no r emission in 6 months **or** more joints involved: treat as for established polyarthritis and refer to a rheumatologist.

Note: Refer all patients early to screen for uveitis, especially if ANA positive.

Polyarthritis – early
Start NSAID as soon as possible.
- ▪ NSAID, e.g.:
- Ibuprofen, oral, 10 mg/kg/dose 4 – 6 hourly.

Efficacy is determined within weeks unless there is aggressive progression or severe adverse effects, i.e. gastric irritation, peptic ulcer, hepatic toxicity, renal impairment or platelet dysfunction.

Refer for initiation of disease-modifying antirheumatic drugs (DMARDs).

If no significant improvement in 1 month, or if severe, at onset, start disease modifying drug:
- Methotrexate, oral, 10–15 mg/m^2/week as a single dose on an empty stomach. (Specialist initiated)
    - o Maximum dose: 25 mg/week.

PLUS
- Folic acid, oral, 5 mg daily for the duration of the treatment.

Note:
Intra-articular steroids (IAS) may be used in conjunction with methotrexate.

In severe early disease, for induction and rapid relief of symptoms, consider

- Prednisone, oral, starting dose: 1 mg/kg/dose once daily.
  - Reduce dose gradually to 7.5 –5 mg daily, depending on response.

Established arthritis (oligo- or polyarthritis)

- Implies active disease for 6 months or more on above regimes.
- Change to, or start subcutaneous methotrexate, maximize dose as above.
- If no response within 3 months refer.

Systemic onset JIA

Systemic JIA is an aggressive systemic disease. Refer to a rheumatologist early. Initiate treatment after consultation with a rheumatologist.

- NSAID, e.g.:
- Ibuprofen, oral, 10 mg/kg/dose 4 – 6 hourly.

Efficacy is determined within weeks unless there is aggressive progression or severe adverse effects, i.e. gastric irritation, peptic ulcer, hepatic toxicity, renal impairment or platelet dysfunction.

For patients with mild disease begin with:

- Prednisone oral, 2 mg/kg as a single daily dose.
  - Once disease has become inactive, reduce dose gradually.

For critically ill patients with internal organ involvement, such as pleuritis, pericarditis, myocarditis or evidence of early macrophage activation syndrome:

- Methylprednisolone, IV, 30 mg/kg/day for 3 days.

Follow with:

- Prednisone oral, 2 mg/kg as a single daily dose until disease is controlled.
  - These patients may respond in the long term to methotrexate or cyclosporine but the response is not as good as other JIA patients. Refer early for treatment.

Psoriatic arthritis

Treat as for oligoarthritis if ≤ 4 joints, or polyarthritis if severe disease or >4 joints at onset.
Refer early as most children will require a DMARD.

Enthesitis related arthritis

Start NSAID as soon as possible.

- NSAID, e.g.:
- Ibuprofen, oral, 10 mg/kg/dose 4 – 6 hourly.

If severe disease:

- Prednisone, oral, 1–2 mg/kg as a single daily dose for 2 weeks and reduce dose rapidly.
  - If no remission in 2–4 months, refer.

Uveitis management
Manage in consultation with an ophthalmologist.
Use local and intra-ocular steroid first.

For poor response:
- Prednisone, oral, 1–2 mg/kg as a single daily dose for 2–3 months.

REFERRAL
» **Urgent:** uncontrolled systemic disease.
» All for confirmation of diagnosis.
» Established arthritis (oligo- or polyarthritis).
» Suspected JIA not responding to NSAID therapy.
» For slit lamp examination, if not locally available.
» Patients with iridocyclitis and uveitis.
» Adverse reaction to NSAID.
» For orthopaedic or orthodontic treatment, e.g. where intra-articular corticosteroids is indicated.
» All patients requiring DMARD to specialist.

12.3 KAWASAKI SYNDROME/MUCOCUTANEOUS LYMPH NODE SYNDROME
M30.3

DESCRIPTION
Kawasaki syndrome is an acute self-limiting systemic vasculitis of unknown aetiology occurring predominantly in c hildren. It i nvolves the small and medium arteries. Most serious complication is coronary artery aneurysms.

DIAGNOSTIC CRITERIA
Clinical
» There is no diagnostic test.
» Confirm diagnosis by the presence of fever for ≥ 5 days, lack of another known disease process to explain the illness and the presence of 4 of the 5 criteria listed below:
  1. bilateral bulbar conjunctival injection without exudates;
  2. changes of the lips and oral cavity: reddening of the oral mucosa, pharynx, lips, strawberry tongue, cracking of lips;
  3. polymorphous rash, primarily on the trunk;
  4. cervical lymphadenopathy (lymph nodes >1.5 cm diameter);
  5. changes of the extremities, including reddening of the palms and soles, oedema of the hands and/or feet and desquamation of the finger tips and toes.
» A high index of suspicion is required especially in younger children who may present without all of the above or may have incomplete/atypical Kawasaki.

» Important differential diagnosis:
  > aseptic/bacterial meningitis,
  > viral or drug eruption,
  > bacterial adenitis,
  > diseases mediated by staphylococcal or streptococcal toxins,
  > rickettsial diseases,
  > urinary tract infection.

Investigations
» C-reactive protein.
» FBC: increased white blood cell count and thrombocytosis.
» Urine dipstick: transient pyuria.
» ESR: elevated.
» Echocardiography to detect coronary artery aneurysms: 100% sensitivity, 97% specificity, done at beginning and 6 weeks after disease improvement.

GENERAL AND SUPPORTIVE MEASURES
» Routine supportive care.
» Tepid sponging for fever.
» Maintain hydration with oral fluids.

MEDICINE TREATMENT
As soon as diagnosed and preferably within first 10 days from onset of fever after specialist consultation.
- Immunoglobulin, IV, 2 g/kg as a single dose administered over 12 hours.
  - o Repeat dose, if necessary, if temperature doses not normalise or rash does not resolve within 24 hours.

If fever continues after 2 doses:
- Methylprednisolone, IV, 30 mg/kg/dose. Specialist consultation.

All children:
- Aspirin (high dose), oral, 20 mg/kg/dose 6 hou rly for 72 hrs or unti l fever settles.

Follow with:
- Aspirin, oral, 3–5mg/kg/day until ESR and platelet count are normal if there are no coronary artery aneurysms.
  - o Continue for at least 2 years if coronary artery abnormalities have resolved and for life if coronary artery aneurysms persist.

REFERRAL
» All patients for confirmation of diagnosis.
» For echocardiography to confirm presence of coronary artery aneurysms.

12.4 SYSTEMIC LUPUS ERYTHEMATOSUS

M32

DESCRIPTION

Systemic lupus erythematosus (SLE) is a multisystem inflammatory disease characterised by the presence of auto-antibodies directed against various cellular components, particularly DNA. It is often associated with antiphospholipid-antibody-mediated hypercoaguability. In children it predominantly targets the kidneys (in 50–80%), central nervous system, skin and joints.

Treatment of acute lupus depends on severity of illness, with more aggressive treatment for CNS, renal and haematologic involvement.

DIAGNOSTIC CRITERIA

Clinical

Diagnosis may be elusive due to its variations in presentation and is confirmed with at least 4 of 11 criteria:

1. malar rash-rash over cheeks, sparing nasal folds;
2. discoid rash-erythematous patches heal with scarring;
3. photosensitivity – skin rash as a result of unusual reaction to sunlight;
4. oral or nasopharyngeal ulcers;
5. non-erosive arthritis-tenderness, swelling or effusion;
6. pleuro-pericarditis;
7. renal disease, i.e. proteinuria and/or cellular casts;
8. neurologic disorder, i.e. seizures or psychosis in the absence of precipitating circumstances;
9. haematologic disorder: haemolytic anaemia, leukopaenia, lymphopaenia, thrombocytopaenia;
10. immunologic disorder:
    a) anti-dsDNA antibody,
    b) anti-Sm (Smith) antibody,
    c) positive antiphospholipid antibodies (anticardiolipin, lupus anticoagulant),
    d) false positive antitreponemal test;
11. positive anti-nuclear antibody (ANA) test.

Investigations

Lack of urinary sediment changes do not exclude active ongoing glomerulonephritis, especially interstitial nephritis.

» Urine dipstick: haematuria and proteinuria.
» Urine microscopy: cellular casts.
» FBC: differential and platelet count.
» Complement, antinuclear antibodies, anti-dsDNA antibodies.
» Screen for thyroid involvement.
» Serum urea, creatinine, electrolytes, albumin and cholesterol.
» Clotting profile, anti-phospholipid antibody and lupus anti-coagulant.
» Electrocardiography and chest X-ray.

GENERAL AND SUPPORTIVE MEASURES

» Counselling education and a team approach.
» Adequate rest and appropriate nutrition.
» Protect from sunlight, sunscreen, hats and avoidance of sunlight if unprotected.
» Physiotherapy to relieve arthralgia.
» Psychological support.
» Cosmetic management.
» Immunisation, especially pneumococcal vaccine.
» Prompt management of infections.
» Vitamin D and calcium supplementation.

MEDICINE TREATMENT

All children should be treated by a specialist.

All children:

- Chloroquine (as base), oral, 5 mg/kg/dose once daily.
  - o Maximum dose: 150 mg.
  - o 6-monthly eye examination necessary.

<u>Induction therapy</u>
General systemic disease, cutaneous disease, serositis or musculoskeletal disease:

- Corticosteroid treatment:
- Prednisone, oral, 2 mg/kg/day. Maximum daily dose, 60 mg.
  - o Reduce dose to 0.5 mg/kg/day once daily by 2 months.

Musculoskeletal or skin disease:

- Methotrexate, oral, 10–15 mg/m^2/week as a single dose on an empty stomach. Specialist initiated.
  - o Maximum dose 25mg/week.

PLUS

- Folic acid, oral, 5 mg daily for the duration of the treatment.

Major organ involvement, lupus nephritis class 3 or 4 or neuropsychiatric involvement, refer.

- **Additional immunosuppressive therapy**

Indicated for all patients with life threatening disease particularly lupus nephritis and cerebral lupus. Specialist initiated.

Lupus nephritis class 3 or 4 or neuropsychiatric disease
<u>Induction therapy</u>

- Methylprednisolone, IV, 30 mg/kg/dose (maximum 1000 mg) for 3 days.

Follow with:

- Prednisone, oral, 2 mg/kg/day.
  - o Reduce dose to 0.5 mg/kg/day once daily by 2 months.

AND

- Cyclophosphamide, IV, 500–750 mg/m^2/dose, administered over 2 hours
  - o Repeat once a month for 6 months.
  - o Cyclophosphamide must be given with pre-hydration and continue increased fluid intake for 24 hours after cyclophosphamide infusion,
  - o Monitor vital signs during administration of cyclophosphamide.

Maintenance treatment (steroid sparing treatment)

- Azathioprine, oral, 2–3 mg/kg/dose as single daily dose.
  - o Maximum dose: 150 mg.
  - o Refer if contraindication to azathioprine or if patient develops adverse effects with treatment.

REFERRAL

» All patients for confirmation of diagnosis and initiation/supervision of treatment.
» All patients receiving chloroquine treatment must be referred for ophthalmologic examination, if not locally available.
» Lupus nephritis class 5 or macrophage activation syndrome.
» For kidney biopsy if any evidence of renal disease.

12.5 TAKAYASU ARTERITIS

M31.4

DESCRIPTION

Takayasu arteritis is a chronic inflammatory disease involving large vessels, including the aorta and its main branches and the pulmonary vasculature. Lesions are typically segmental – obliterative and aneurysmal. Symptoms reflect end organ ischaemia.

DIAGNOSTIC CRITERIA

Clinical

Angiographic abnormalities of the aorta or its main branches plus at least one of the following:

» Hypertension with no obvious kidney disease.
» BP difference in limbs >10 mm Hg.
» Decrease in peripheral arterial pulses/absent pulses.
» Vascular bruits, particularly over aorta or main branches, carotids, subclavian, abdominal vessels.

May be associated with:

» Congestive cardiac failure associated with aortic regurgitation/dilated cardiomyopathy/hypertension.
» Neurologic signs secondary to hypertension/ischaemia.
» Any signs of unexplained inflammatory activity.
» Strongly positive TST.
» Discrepancy in kidney sizes.

Investigations
- » C-reactive protein.
- » ESR.
- » Plasma rennin.
- » Serum urea, creatinine and electrolytes.
- » TST to exclude tuberculosis.
- » Electrocardiography and chest X-ray.
- » Radio-isotope study of kidneys to demonstrate split renal function.

GENERAL AND SUPPORTIVE MEASURES

Acute hypertension:
- » Bed rest – Fowler's position.
- » Control fluid intake and monitor output with uncontrolled hypertension.
- » Restrict dietary sodium.
- » Manage end organ effects.

MEDICINE TREATMENT

Treat hypertension – refer to chapter 3 for management of hypertension.

CAUTION
Never use ACE inhibitor or angiotensin receptor blocker if bilateral renal artery stenosis is present.
Avoid ACE inhibitor if possible due to risk of acute renal failure.

Consider TB treatment if tuberculosis cannot be conclusively excluded.

- Aspirin soluble, oral, 5 mg/kg/day as single daily dose.

Induction therapy (Specialist initiated)
- Cyclophosphamide, IV bolus, 500–750 mg/m^2 as a single dose.

Continue maintenance treatment with:
- Methotrexate, oral, 10–15 mg/m^2/week. Specialist initiated
  - o Maximum 25mg/week.

PLUS
- Folic acid, oral, 5 mg daily for the duration of the treatment.

If disease activity is not controlled:
ADD
- Prednisone, oral, 2 mg/kg/day for maximum of 4 weeks.
  - o Reduce dose slowly over 12 weeks to 0.25 mg/kg on alternate days.

For children with extensive disease (arteritis on both sides of d iaphragm and continued activity of disease), continue with:
- Prednisone, oral, 0.25 mg/kg on alternate days for indefinite period.

REFERRAL

» All patients for confirmation of diagnosis with conventional angiography or magnetic resonance imaging angiography.

CHAPTER 13

THE NERVOUS SYSTEM

13.1 LUMBAR PUNCTURE

CONTRAINDICATIONS TO LUMBAR PUNCTURE

- Focal neurological signs and depressed level of consciousness.
- Clinical signs of raised intracranial pressure, or impending cerebral herniation:
  - deep coma, i.e. GCS < 13, or sudden deterioration of level of consciousness,
  - decerebrate or decorticate posturing,
  - neurogenic hyperventilation,
  - unequal dilated or poorly reactive pupils,
  - absent doll's eye reflex.
- Haemodynamic/respiratory unstable patients.
- Clinical meningococcaemia (septicaemia) with petechiae/purpura. (confirm with skin scrape, Gram stain and blood culture.)
- Skin sepsis or abnormalities over lumbar puncture site.

PROCEDURE

- Positioning and restraint are vital in determining the success of the procedure.
- The ability of the assistant in restraining is as important as the skill of the 'operator'.
- Preparation entails not only positioning, but attention to sedation/analgesia, 'patient comfort' and safety, as well as factors such as adequate lighting.
- Pay attention to the sterility of the operating field.
- Local analgesia with/without sedation may be required. See section 20.1: Management of pain.
- Ensure that all necessary equipment, e.g. needles, manometers and specimen tubes are close at hand.
- Only the interspaces below L3 (L3/L4 or L4/L5) are used in order to avoid damaging the conus medullaris.
- With the patient in the lateral recumbent position, the L3/L4 interspace is found at the level of the line joining the highest points of the two iliac crests.
- Turn the bevel of the needle (with stylet) to face the patient's side so as to avoid cutting the longitudinal dural fibres.

» As the ne edle is a dvanced, the firs t 'give' or lo ss of r esistance is encountered with the pierc ing of the lig amentum flavum. A sligh t 'popping' sensation is felt as the needle penetrates the dura.
  Remove the stylet to allow CSF to drain out passively. If no fluid appears, then rotate the needle a quarter turn (90°). If this does not help, replace the stylet and advance the needle a few millimetres and then check for fluid as before.
» Measure the opening pressure using a manometer, with the child relaxed in the lateral decubitus position. In a young relaxed child the opening pressure is in the range of 60–150 mm H_2O.
» At end of procedure, re-insert stylet before removing the needle completely.

Note:
If intracranial infection is suspected, initiate antimicrobial treatment immediately.
Remember to catch a few drops of CSF on a labstick to check for the glucose and white cells which may give an indication of an infection.

13.2 SEIZURES

R56.8

DESCRIPTION

A seizure is a change in movement, attention or level of awareness that is sustained or repetitive and occurs as a result of abno rmal and excessive neuronal discharges within the brain.

When unprovoked seizures are recurrent or ty pical of a specific syndrome, then the ter m epilepsy is used and specific management applies. See section 13.3: Epilepsy.

International League against Epilepsy
Classification of seizures is aetiological and clinical.

Aetiology
» Symptomatic causes with underlying pathology evident.
» Idiopathicor genetic.
» Unknown.

The causes of seizures are multifactorial.

The commonest seizures in children are febrile convulsions but the history, examination and subsequent investigation must be aimed at eliciting/excluding the following examples of differential diagnoses:

**Past perinatal conditions**	**Infections**	**Poisoning**
» congenital infection<br>» hypoxic-ischaemic damage<br>» trauma<br>» cerebral haemorrhage or thrombosis<br>» cerebral malformation or degeneration	» meningitis<br>» encephalitis<br>» brain abscess<br>» febrile convulsion	» accidental ingestion of medicines<br>» medicine withdrawal in infancy<br>» environmental toxins
**Metabolic conditions**	**Systemic disorders**	**Primary cerebral causes**
» hypoglycaemia<br>» hypocalcaemia<br>» hypomagnesaemia<br>» hyponatraemia<br>» hypernatraemia<br>» inborn errors of metabolism	» vasculitis<br>» hypertensive encephalopathy<br>» uraemia (renal failure)<br>» hyperammonaemia (liver failure)	» trauma<br>» haemorrhage<br>» thrombosis<br>» genetic/familial (syndromic)<br>» tumour<br>» idiopathic

Clinical
Within each of the above categories generalised, focal or syndromic seizures occur.

<u>Generalised seizures:</u>
The epileptic focus arises centrally and spreads to involve both hemispheres of the brain.
Generalised seizures may be:
- » tonic-clonic (grand-mal convulsion),
- » absence (typical or atypical),
- » clonic,
- » tonic or atonic,
- » myoclonic.

Generalised tonic-clonic seizures (GTCS) that continue or recur for more than 30 minutes without regaining consciousness are called Convulsive Status Epilepticus: See section 13.5: Status epilepticus (convulsive).

Focal seizures:
The epileptic activity arises from a particular focus within one hemisphere of the brain.
- » Simple focal seizure: A focal seizure with unaltered consciousness.
- » Complex focal seizure: A focal seizure with spread of the seizure to involve regions that subserve consciousness, memory, emotion and vision, resulting in an altered level of consciousness.
- » Partial seizures may progress to generalised tonic-clonic seizures and that is known as secondary generalisation

Epileptic Syndromes – See section 13.3: Epilepsy.

DIAGNOSTIC CRITERIA
Clinical
- » Obtain a history:
  - > eye witness account, aura,
  - > perinatal history, developmental history, school record, family history and environment.
- » Examine to exclude obvious aetiology, but in particular look for occult causes:
  - > general: skin abnormalities, e.g. Sturge-Weber syndrome and tuberous sclerosis complex;
  - > CNS examination for loss of consciousness, neck stiffness localising signs, head growth, developmental milestones and fundoscopy;
  - > CVS examination: check blood pressure.

Investigations
Investigations should be individualised according to clinical indication.

Always consider hypoglycaemia as a primary or aggravating cause of any seizure.

Basic investigations:
- » blood glucose in all children;
- » rapid test for malaria for those who have recently travelled to a malaria area;
- » electrolytes (Na, Ca, Mg) in sick and young children;
- » blood culture in febrile children;
- » FBC;
- » lumbar puncture: if m eningitis is suspected and f or first febrile generalised tonic-clonic seizures in children < 2 years old;

Note:
If the seizure has progressed to status epilepticus (i.e. lasted > 30 minutes), then lumbar puncture is contraindicated until raised intracranial pressure is excluded.

» CT-scan (brain): if persistently reduced coma score (GCS < 12/15) without known cause, raised intracranial pressure or focal intracranial pathology is suspected;
» EEG: is indicated for recurrent or syndromic seizures where diagnosis cannot be made on clinical grounds alone. Delay EEG for at least one week after the convulsive episode.

GENERAL AND SUPPORTIVE MEASURES

» Ensure an open airway and administer oxygen.
» Position to prevent aspiration of vomitus, i.e. recovery position.
» Check glucose during the seizure and blood pressure after the seizure.
» Obtain intravenous access if seizure duration > 5 minutes.
» Keep child nil per mouth and intravenous fluid volumes at maintenance rates.
» Control fever with tepid sponging.
» Aetiology will determine further management.

MEDICINE TREATMENT

(Of a first time **seizure)**

For fever:

- Paracetamol, oral, 15 mg/kg/dose 6 hourly, as required.
  - o In an unconscious child, administer paracetamol via a nasogastric tube.

Urgent medicine treatment is only indicated if the seizure is generalised and lasts more than 5 minutes or is causing systemic compromise. Treat as for Status epilepticus: See section 13.5: Status epilepticus (convulsive).

For the management of persistent or recurrent seizures that are not generalised, see section 13.3: Epilepsy.

13.3 EPILEPSY

G40.9

DESCRIPTION

A condition characterised by recurrent unprovoked seizures associated with abnormal and excessive paroxysmal neuronal discharges.

See International League against Epilepsy classification of seizures in section 13.2: Seizures.

When unprovoked seizures are recurrent, persistent or syndromic, then the child has epilepsy.

Besides the classification according to types there are also specific seizure syndromes which impact on management.
1. Absence epilepsy of childhood
2. Benign focal epilepsy of childhood
3. Infantile spasms
4. Lennox-Gastaut syndrome
5. Severe myoclonic epilepsy of infancy
6. Generalised epilepsy with febrile seizures plus (GEFS+)

Epileptic syndromes include:

Childhood absence epilepsy
» Short spells of sudden onset of motor arrest and impairment of consciousness lasting between 5 and 15 seconds.
» Little or no associated movements.
» No post-ictal effect.
» Onset 3 years until puberty.

Benign focal epilepsy of childhood
» Sleep related events of hemifacial clonic spasm.
» Inability to speak but retained awareness.
» Peak onset at ± 6–10 years.
» Usually resolves by late adolescence.

Infantile spasms (West syndrome)
» An infantile onset encephalopathy with epileptic spasms associated with hypsarrhythmia on the EEG and developmental regression.
» It is a neurological emergency. Do not delay diagnosis, treatment and referral. Early intervention reduces the subsequent neurodisability.
» Clinically, the child appears to stare, gives a sudden flexion of the trunk and head, with the limbs in extension or flexion but held in this tonic spasm for a few seconds.
» Events occur in runs and are most common when the infant is going to sleep or rousing.
» The episodes are distressing to the infant and he will often appear red in the face and may cry out.
» Events are often confused with colic.

Lennox-Gastaut syndrome (LGS)
» Combinations of GTCS, atypical absences, myoclonic seizures, tonic seizures, atonic drop attacks and occasionally complex partial seizures.
» May occur spontaneously but usually symptomatic.
» Onset between 2–3 years of age.
» Behavioural problems and neuroregression occurs.

Severe Myoclonic Epilepsy of Infancy (SMEI)

» Onset in children < 1 year of age with recurrent clusters of febrile convulsions, severe neuroregression and other non-febrile seizures by 2–3 years.

Generalised epilepsy with febrile seizures plus (GEFS+)

» Children with febrile convulsions which persist beyond 6 years.
» These children have epilepsy triggered by fever and may warrant anticonvulsant intervention.
» Often family history of febrile convulsions.

Note:
Infantile spasms, Severe Myoclonic Epilepsy of Infancy and Lennox-Gastaut syndrome are regarded as epileptic encephalopathies and are associated with neuroregression and behavioural problems.

DIAGNOSTIC CRITERIA

A child may be diagnosed:

» with a specific anatomical or systemic cause for the seizure type (see table of possible causes);
» as having an epileptic syndrome, i.e. a specific seizure type associated with a characteristic EEG, natural history, response to therapy and prognosis;
» with idiopathic epilepsy.

GENERAL AND SUPPORTIVE MEASURES

Acute

» Maintain an open airway.
» Place patient on side.
» Admit to high or intensive care, as necessary.
» If unconscious, consider urinary catheterisation.
» Monitor:
  > heart rate,
  > respiratory rate,
  > blood pressure,
  > electrolytes,
  > blood glucose,
  > osmolality.
  > acid–base status.
  > blood gases,
  > SaO_2,
  > neurological status,
  > fluid balance,
» Measure anticonvulsant blood levels if there are breakthrough seizures on medication, signs of toxicity, drug interactions or concerns about adherence.
» Control fever with tepid sponging and look for a possible cause of the fever and treat appropriately.
» Cardiovascular and/or respiratory support if the patient is unable to maintain blood gases and blood pressure within the normal physiological range.
» Ventilate to maintain $PaCO_2$ in the low normal range, i.e. 4–4.5 kPa.

LONG TERM

» Minimise the impact of the epilepsy by obtaining complete seizure control to maximise child's full potential.
» Educate the patient and caregiver about epilepsy and associated complications and comorbidities, i.e. learning difficulties and ADHD.

MEDICINE TREATMENT

For acute generalised tonic clonic seizures see section 13.5: Status epilepticus (convulsive).

To maintain SaO_2 of ≥ 95%:

- Oxygen 100 % by facemask or nasal cannulae.

Maintenance therapy

Monotherapy is preferred but combination therapy may be necessary.
Combination therapy should be specialist initiated.
As a general rule, start with small doses and titrate slowly up.
Aim for low to mid-therapeutic dose range and accept the lowest dose at which seizures are controlled.
If seizures continue, titrate to high therapeutic doses, if no unacceptable side-effects.
Measuring drug levels is rarely indicated unless there is concern about toxicity or adherence.

Maintenance medicine treatment choices for different types of epileptic seizures.

	**1st line**	**2nd line**
**Generalised tonic and/or clonic**	• Valproate<br>**OR**<br>• Phenobarbitone (< 6 months old)	• Lamotrigine (specialist advice)
**Partial**	• Carbamazepine	• Lamotrigine (specialist advice)
**Infantile spasms**	Refer all	
**Absence**	• Valproate	• Lamotrigine
**Myoclonic**	Refer all for specialist investigation and initiation of therapy with valproate	

- Valproate, oral, 5 mg/kg/dose, 8–12 hourly.
  - o Increase to 15–20mg/kg/dose 8–12 hourly.
  - o Maximum total daily dose: 40 mg/kg/day.
  - o Exclude liver dysfunction prior to initiating therapy (at least ALT), in children under 2 years or if clinical suspicion of liver dysfunction.
  - o Monitor at least clinically for hepatotoxicity.

- Carbamazepine, oral, 2 mg/kg/dose (starting dose).
  - o Increase slowly at 2 weekly intervals to 5–10 mg/kg/dose 8–12 hourly.
  - o Usual maintenance total daily dose: 10– 20 mg/kg.
  - o Maximum total daily dose: 20 mg/kg/day.
  - o Dosage intervals: syrup 8 hourly, tablets 12 hourly.
  - o Exacerbates myoclonic seizures and absence seizures.

- Lamotrigine, oral, 0.2 mg/kg/dose starting daily dose. (Specialist initiated)
  - o Increase slowly at 2 weekly intervals to 1–5 mg/kg/dose 12–24 hourly.
  - o Maximum dose when given with valproate: 5 mg/kg/day.
  - o Lamotrigine is given as add-on therapy for different seizure types and in drug-resistant paediatric epileptic syndromes, such as Lennox-Gastaut syndrome.
  - o Double the dose of lamotrigine when using carbamazepine or phenobarbitone and halve the dose when using valproate.

- Phenobarbitone, oral, 3–5 mg/kg/dose as single dose at night.
  - o May be used in children under six months of age.
  - o Is not recommended as maintenance therapy for children older than 2 years due to undesirable side effects such as sedation, behaviour disturbances, hyperkinesia and dependence, except in si tuations where there is poor adherence to other drugs.
  - o Exacerbates absence seizures.

REFERRAL

» Suspected but undiagnosed secondary cause.
» Partial seizures for neuro-imaging, if facilities or expertise not available.
» Generalised seizures other than typical febrile convulsions in children < 2 years.
» Seizures that are not controlled within 2 months on one agent with minimal side effects.
» Neuroregression.
» Mixed seizure types in one patient.
» All myoclonic seizures and infantile spasms at presentation.
» If need to add a second medicine.

13.4 SEIZURES, FEBRILE

R56.0

DESCRIPTION

Seizures occurring in children between the ages of 3 months and 6 years associated with a rapid rise in temperature at the beginning of an extracranial illness.
Febrile seizures can be simple or complex febrile seizures.

Simple febrile seizures:

» are generalised tonic-clonic seizures,
» are self limiting, usually less than 5 and always less than 15 minutes,
» cause no neurological deficit after the convulsion,
» have a good prognosis and very rarely develop into epilepsy,
» often consist of only one seizure which needs no specific treatment,
» there is often a family history of febrile seizures.

Complex febrile seizures – febrile seizures with one or more of the following:

» last longer than 15 minutes,
» are recurrent within the same febrile illness,
» have a focal (partial) onset,
» have post-ictal, focal neurological abnormalities.

Risk factors for recurrent febrile seizures include:

» seizure disorder in a first degree relative,
» onset before 12 months of age,
» complex initial seizures.

DIAGNOSTIC CRITERIA

Clinical

» Exclude intracranial, extracranial and biochemical causes.
» Signs of meningism are unreliable in children < 2 years of age.
» If raised intracranial pressure or meningitis cannot be excluded then the diagnosis of febrile seizures cannot be made. Treat children empirically for meningitis.

Investigations

» Lumbar puncture is indicated in:
  > children < 2 years of age for exclusion of intracranial infection even when signs of meningism are absent,
  > all children who have no focus of infection, particularly those who have received antibiotics prior to the event.
» In children > 2 years of age, where a focus of extracranial infection is present and intracranial infection has been excluded clinically, no further investigation is required.

- » All children with complex febrile seizures and persistent lethargy require neuro-imaging and then a lumbar puncture if raised intracranial pressure can be reliably excluded.
- » Investigate complex febrile seizures to rule out meningitis, focal brain lesions, epilepsy and other possible underlying conditions depending on the clinical picture.
- » An EEG is of no value in simple febrile seizures, but consider in complex febrile seizures.

GENERAL AND SUPPORTIVE MEASURES

- » Control fever with tepid sponging.
- » Reassure parents and caregivers.
- » Educate parents and caregivers regarding the management of future episodes of fever.

MEDICINE TREATMENT

For fever (temperature > 38.5˚C):

- Paracetamol, oral, 15 mg/kg/dose 6 hourly until fever subsides (administered by parents).
  - o Paracetamol has no effect on seizure prevention.

If convulsing:
See section 13.5: Status epilepticus (convulsive).

Continuous prophylactic therapy

Routine daily prophylaxis is not recommended for patients with simple febrile seizures.
For children with recurrent complex febrile seizures, consider prophylactic treatment after weighing the benefits against the risks and appropriate parental counselling.

REFERRAL

- » All patients with complex febrile seizures without an obvious cause of the seizure and/or not responding to initial management should be discussed with a specialist.
- » Developmental delay/regression.

13.5 STATUS EPILEPTICUS (CONVULSIVE)

G41.9

DESCRIPTION

Convulsive status epilepticus (SE) is a **medical emergency** defined as a generalised tonic-clonic convulsion that persists for 30 minutes or longer, or is repeated frequently enough to prevent recovery of consciousness between attacks.

After 30 minutes of generalised tonic-clonic seizures, the brain begins to suffer from hypoxia, acidosis, depletion of local energy stores, cerebral oedema and structural damage.

Complications include:

- » hyperpyrexia,
- » respiratory depression,
- » cerebral oedema,
- » disturbances of blood glucose,
- » renal failure,
- » acidosis,
- » blood pressure disturbances,
- » inappropriate antidiuretic hormone (ADH) secretion,
- » hypoxic, ischaemic damage to brain, myocardium and muscles.

DIAGNOSTIC CRITERIA

Clinical

- » Convulsive seizure lasting 30 minutes or longer.
  Manage convulsive seizures that have lasted for 5 minutes or more as for status epilepticus.
- » Convulsive status epilepticus may be:
  - > Idiopathic;
  - > secondary to an insult to the brain, e.g. encephalitis, hypoxic episode, trauma and complex febrile seizures;
  - > as a result of non-adherence and changes in anticonvulsant therapy.

GENERAL AND SUPPORTIVE MEASURES

- » Maintain an open airway.
- » Place patient on side.
- » Admit to high or intensive care, if possible.
- » If unconscious, consider catheterisation.
- » Monitor:
  - > heart rate,
  - > respiratory rate,
  - > blood pressure,
  - > electrolytes,
  - > blood glucose,
  - > anticonvulsant blood levels,
  - > acid–base status,
  - > blood gases,
  - > SaO_2,
  - > neurological status,
  - > fluid balance,
  - > osmolality.
- » Control fever with tepid sponging.
- » Cardiovascular and/or respiratory support if the patient is unable to maintain blood gases and blood pressure within the normal physiological range.
- » Ventilate to maintain $PaCO_2$ in the low normal range, i.e. 4– 4.5 kPa.

MEDICINE TREATMENT

To maintain $SaO_2 \geq 95\%$:

- Oxygen 100%, by facemask or nasal cannulae.

Status epilepticus
Follow ABCD approach.
See flow chart on next page for management of Status epilepticus.
For buccal midazolam and rectal diazepam, use the contents of an ampoule.

For the purpose of rationalising the management of convulsive status epilepticus, it helps to divide or classify it into different stages as below:
» Early SE (5-20 minutes)
» Established SE (20–60 minutes)
» Refractory SE (beyond 60 minutes)

Intravenous fluid:
- Dextrose 5% in sodium chloride 0.9%, IV.
  - o Avoid overhydration. Keep fluid volume at maintenance.
  - o Maintain normoglycaemia and electrolytes within the normal range.

Cerebral oedema
Treat when clinically suspected/proven.
If the patient has a serum osmolality < 320:
- Mannitol, IV, 250 mg/kg administered over 30–60 minutes.
  - o Do not exceed two doses without consulting with a specialist.

OR
Under specialist supervision:
- Sodium chloride 5%, IV, 2 mL/kg infused over 30 minutes.

Cerebral oedema with associated space occupying lesion
- Dexamethasone, IV, 0.5 mg/kg 12 hourly.

Other biochemical disorders
Correct abnormalities, if present, e.g. glucose, calcium and sodium.

For fever:
- Paracetamol, oral, 15 mg/kg/dose, 6 hourly as required via nasogastric tube.

Once fits are controlled consider maintenance therapy.

EARLY STATUS (5-20 MINUTES)

IN HOSPITAL

If IV access:
- Lorazepam, IV, 0.1 mg/kg

OR

If no IV access
- Lorazepam, IM, 0.1 mg/kg

OR
- Diazepam, rectal, 0.5 mg/kg

OR
- Midazolam, buccal, 0.5 mg/kg

AT PHC
- Diazepam, rectal, 0.5 mg/kg/dose

Repeat after 10 minutes if convulsions continue.
Maximum dose: 1 mg/kg in 1 hour

If no response after the 2nd dose of diazepam

- Phenobarbitone, oral by NG tube, 20 mg/kg/dose as a single dose.

If convulsing after 10 minutes

- Lorazepam, IV, 0.1 mg/kg.

If convulsing after 10 minutes

ESTABLISHED STATUS (20-60 MINUTES)

- Phenytoin, IV, 18 mg/kg infused over 20 minutes in glucose-free solution.

Cardiac monitoring is essential.
Monitor for bradycardia, arrhythmias, hypotension – if so, then stop infusion until stable then restart at 2/3 the starting rate.

OR IF CARDIAC MONITORING IS NOT POSSIBLE
- Phenobarbitone, IV, 20 mg/kg as a single dose.

If still convulsing after 20 minutes from starting either intravenous phenobarbitone or phenytoin treatment

- Phenobarbitone, IV, 20 mg/kg as a single dose.

If still convulsing

REFRACTORY STATUS (> 60 MINUTES)

Refer to ICU for consideration of:
a. midazolam infusion
b. intubation and thiopental sodium

Note:
Once intravenous access is attained take blood for glucose, blood gases, electrolytes, FBC and culture and anticonvulsant levels if patient is a known epileptic.

Monitor carefully for drug related respiratory depression.

Intubation, ventilation and administration of thiopental sodium infusion should only be performed in a centre with trained anaesthetists and a paediatric intensive care unit.

REFERRAL

Caution
Attempt to control seizures and stabilise the patient before referral.

- » Failure to control seizures within 1 hour.
- » Where the primary cause is unknown, or if the primary cause itself requires referral.

13.6 ANTITRETOVIRAL THERAPY AND ANTICONVULSANTS

Co-administration of anticonvulsant treatment in patients on antiretroviral therapy has not been well studied yet, and remains a therapeutic challenge. Drug interactions between anticonvulsants and antiretrovirals can arise from a number of mechanisms, including liver metabolism (increase or decrease), competition for protein binding and increase in viral replication. There is at present no strong evidence to guide clinicians.

The following points are important to remember when treating seizures and epilepsy in patients on ART:

- » The main area of caution is in drugs metabolised in the liver by the cytochrome P450 enzyme system as this may cause alterations in levels of both anticonvulsants and antiretrovirals leading to toxic or sub-therapeutic drug levels. This particularly pertains to the NNRTI's and more specifically to PI's.
- » If clinically indicated, monitor anticonvulsant levels in patients taking concurrent ART and anticonvulsant therapy.
- » Avoid prescribing carbamazepine, phenobarbital, and phenytoin for patients receiving NNRTIs or PIs, as there are serious P450 interactions involved. In this setting consider lamotrigine and valproate. See section 13.3: Epilepsy.

» Treat children on antiretrovirals presenting to casualty with acute seizures or in status epilepticus according to the existing standard status epilepticus or acute seizure protocols.
» Although benzodiazepines, phenytoin and phenobarbitone may interact with antiretroviral metabolism, the acute management of acute seizures or SE takes precedence in these instances.

13.7 HEADACHES

R51

DESCRIPTION

Headache is the most common pain syndrome in children of all ages. Recurrent headaches are a common health problem and can be:

» primary, e.g. migraine, or
» secondary/symptomatic, e.g. raised intracranial pressure.

The actual perception of headache varies according to age and is influenced by factors such as experience, memory and cultural environment.

International Classification of Headache Disorders (ICHD)
Migraine (without aura)

Five or more headaches lasting 1–48 hours (duration in children is often shorter, lasting a few hours only) fulfilling at least 2 of the following:

» bilateral or unilateral, frontal or parietal in location,
» pulsating in character,
» moderate or severe,
» aggravated by routine activity,
» nausea and/or vomiting plus photophobia and/or phonophobia during headache.

Migraine (with aura)

At least 2 attacks fulfilling at least 3 of the following:

» one or more reversible aura symptoms,
» at least one aura developing over > 4 minutes or 2 or more successive symptoms,
» no aura lasting > 1 hour,
» headache follows aura in less than 1 hour.

Episodic tension-type headache

At least 10 prior episodes, occurring less than 15 times per month and lasting 30 minutes to 7 days with at least 2 of the following:

» pressing or tightening quality,
» mild or moderate intensity,
» bilateral location,
» no aggravation by routine physical activity,
» no nausea or vomiting, and photophobia and phonophobia absent during headache.

Cluster headache

» Severe unilateral sharp headache associated with conjunctival injection and lacrimation.
» Rare in childhood.

Paroxysmal Hemicrania Continua

» Cluster headache of shorter duration.

Each of the above can occur in combination in any patient, i.e. mixed/comorbid headache.
Headaches can also be sub-classified according to temporal patterns, i.e. acute, acute recurrent, chronic progressive/non-progressive, episodic or constant.

DIAGNOSTIC CRITERIA

» Exclude secondary causes of headache, e.g. raised intracranial pressure.
» Red flags in childhood headaches:
  > change in pattern (e.g. "worst headache ever"),
  > progressive course over time,
  > age younger than 3 years,
  > nocturnal/wakes child from sleep,
  > early morning vomiting,
  > ataxia,
  > focal neurological signs,
  > alteration of level of consciousness.

GENERAL AND SUPPORTIVE MEASURES

» Environmental and lifestyle changes, e.g. Avoid precipitants such as bright lights, sleep deprivation and certain foods.
» Adequate hydration.
» Avoid skipping meals.
» Regular exercise.
» Stress alleviation and coping skills training where possible.
» Headache diary.

MEDICINE TREATMENT

Treat headaches early with analgesics.

- Paracetamol, oral, 15 mg/kg/dose 6 hourly as required.

For migraine:

- Ibuprofen, oral, 10 mg/kg/dose, 6 hourly.

Persistent vomiting and not tolerating oral feeds:

- Metoclopramide, oral, 0.15–0.3 mg/kg as a single dose.

OR

- Metoclopramide, IM/IV, 0.1 mg/kg as a single dose.

Migraine prophylaxis

Indicated when headaches occur frequently, impacting on the child's activity and requiring substantial relief medication.

Treat for six months then review.

- Propranolol, oral, 0.5–3 mg/kg/day in 2–3 divided doses.
  - o Contraindicated in asthma and heart block.
  - o Avoid in diabetes and depression.

In children who are unable to take propranolol, e.g. asthma:

- Topiramate, oral, 1–3 mg/kg/day in 1–2 doses. (Specialist initiated)
  - o Starting dose: 0.5 mg/kg/day.
  - o Titrate dose slowly every 1–2 weeks.
  - o Reinforce behavioural management before considering topiramate.

REFERRAL

» Secondary intracranial cause suspected.
» Failure to respond to first-line treatment.
» No response to treatment.

13.8 NEUROCYSTICERCOSIS

B69.0

DESCRIPTION

Neurocysticercosis is caused by the cysticercal form, i.e. larval form of the pork tapeworm, *T. solium*. The larvae may locate in the brain parenchyma, intraventricular and meningeal areas, spinal canal/cord and eye, or a combination of these regions. Viable cysticerci incite little inflammatory response, but dead cysticerci elicit an increased inflammatory response.

Cysticerci in the brain may remain dormant or may cause complications such as:

» headache,
» behavioural disorders,
» visual disturbances,
» seizures,
» meningo-encephalitis,
» focal neurological deficits,
» increased intracranial pressure,
» hydrocephalus,
» meningitis,
» spinal cord compression.

DIAGNOSTIC CRITERIA

Clinical

» Location and stage of the life cycle of the parasite in the brain determines the clinical features.

» Suspect if child from endemic area, i.e. pig farming area, p resents with neurological abnormalities such as:
  > Seizures,
  > raised intracranial pressure/hydrocephalus,
  > focal neurological deficits,
  > cranial nerve palsies,
  > meningo-encephalitis,
  > meningitis,
  > behavioural disorders,
  > headache.

Investigations

» Computed tomography (CT scan) and/or magnetic resonance imaging (MRI scan) of brain sho wing cysts, granulomas, peri-lesional oedema or calcification of cysts.

» MRI scan ma y identify more lesions and viable cystic lesions than the CT scan.

» Soft tissue radiolog y of muscles of lower limbs may demonstrate calcified cysticerci, i.e. rice grain calcifications in muscles.

» Follow-up CT scans and/or MRI scans may help to assess the response to therapy.

GENERAL AND SUPPORTIVE MEASURES

Prevention:

» Prolonged freezing or thorough cooking of pork to kill the parasite.

» Thorough washing of fresh fruit and vegetables in *T. solium* endemic areas.

» Attention to personal hygiene.

» Proper sanitation facilities and safe water.

» Avoid the use of human excreta as fertiliser.

» Look for Taenia ova in the stools of the family members.

MEDICINE TREATMENT

Calcified cysticerci and a single dying lesion visible on CT scan require no antihelminthic treatment.

Patients with multiple cysts usually have a mixture of live and dying cysts and are assumed to have active disease and require treatment.

- Albendazole, oral, 7.5 mg/kg/dose 12 hourly for 7 days.
  - o Maximum dose: 400 mg/dose.

Prevention of neurological manifestations
In massive infestations, cysticidal therapy may trigger an inflammatory response. Delaying antihelminthic therapy and adding corticosteroids may lessen the risk.

24 hours **prior** to albendazole therapy:
- Dexamethasone, IM, 0.15 mg/kg/dose 6 hourly.

Then follow with oral therapy as soon as possible:
- Prednisone 1mg/kg/day for the duration of albendazole therapy.

Seizure control
See section 13.3: Epilepsy.
Treat according to the type of seizure.
Anticonvulsant treatment for 6–12 months after neuro-imaging resolution.
Recurrent seizures require chronic treatment until seizure-free for 2 years.

REFERRAL
» All patients for CT scan. Repeat after 6 months to document resolution.
» Neurocysticercosis not responding to adequate therapy.
» Neurocysticercosis with complications, such as hydrocephalus.
» Intractable epilepsy.

13.9 NEUROMUSCULAR DISORDERS

13.9.1 INFLAMMATORY POLYNEUROPATHY (GUILLAIN-BARRÉ SYNDROME)
G61.0
* Notifiable condition

DESCRIPTION
Guillain-Barré syndrome is an acute autoimmune-mediated polyneuropathy which is precipitated by a preceding viral or other infection. It is the most common polyneuropathy in children.

Different forms or **variants of** Guillain-Barré syndrome are described depending on the clinical and/or neurophysiological characteristics.

Acute Inflammatory Demyelinating Polyradiculoneuropathy (AIDP)
» The most common, accounting for 80–90% of cases.
» Characterised mainly by:
  > symmetrical, ascending motor weakness,
  > areflexia, i.e. absence of tendon reflexes,
  > distal sensory alteration,
  > pain/dysesthesiae.

Acute motor axonal neuropathy (**AMAN**)

» Purely a motor form of GBS.
» It involves predominantly motor nerves and has an axonal pattern on electrophysiology (nerve conduction studies).
» Although there are similarities with AIDP, the clinical picture tends to be more severe with more patients suffering from respiratory failure.

Acute motor-sensory axonal neuropathy (**AMSAN)**

» Another axonal form of GBS but with sensory involvement.
» It is not frequently found in children.

Miller-Fischer Syndrome

» Patients have external ophthalmoplegia, sensory ataxia, weakness with areflexia.
» Electrophysiological and CSF studies are similar to AIDP.

Chronic Inflammatory Demyelinating Polyradiculoneuropathy (**CIDP**)

» May be considered a chronic variant of AIDP.
» Most often starts insidiously and progresses slowly, but can have onset like GBS.
» It is managed differently from GBS and should be referred.

DIAGNOSTIC CRITERIA

Clinical

» Preceding respiratory tract or gastrointestinal infection.
» Symmetrical, flaccid muscle weakness with early areflexia.
» The muscle weakness may ascend rapidly upwards to involve the trunk, arms, face and cranial nerves.
» Bulbar paralysis and respiratory failure may develop.
» Autonomic dysfunction.
» Relatively mild, or absence of, sensory signs.
» Exclude the following:
  > poliomyelitis, a rare cau se of hy potonia with abrupt onset of weakness (usually asymmetrical) in association with a febrile illness,
  > transverse myelitis:
    ▪ initial flaccid weakness and areflexia typically involving the lower limbs maximally,
    ▪ occasionally with pain at the onset, but rapidly progressing to spasticity and hyperreflexia,
    ▪ also a sensory level on trunk,
    ▪ bladder and rectal sphincter involvement.
  > Diphtheria,
  > Botulism.

Investigations

Screen for AFP

» Send two stool specimens taken 24–48 hours apart to the National Institute of Virology via the local laboratory.
» Send one stool specimen after 60 days.

CSF

» CSF findings after 1 week show elevated protein and no cells or only a few cells, i.e. albumino-cytological dissociation.
» CSF glucose is normal.

GENERAL AND SUPPORTIVE MEASURES

» Notify as Acute Flaccid Paralysis.
» Admit to a high or intensive care unit.
» Monitor respiratory and autonomic and respiratory functions closely:
  > peak expiratory flow rate,
  > respiratory rate,
  > forced vital capacity (FVC),
  > arterial blood gases.
  > blood pressure,
  > heart rate,
  > bulbar functions,
» Ventilation is recommended when:
  > PCO_2 levels start rising,
  > a progressive fall in the peak expiratory flow rate,
  > tachycardia and sweating occur,
  > inspiratory efforts are weak,
  > inability to talk.

Note:
These changes precede hypoxaemia detected on blood gas analysis, and ventilation should begin before frank hypoxaemia occurs. Respiratory care must be meticulous.

» Shoulder weakness, head-lag, weak cough and swallowing difficulties are an indication for respiratory support.
» To determine fluid losses from autonomic instability monitor urine output and degree of sweating.
» Provide adequate nutrition.
» Bladder and bowel care as well as pressure-point care.
» Routine physiotherapy for chest and limbs, keep ankles in neutral position (90^0) (may require foot/hand splints).
» Protect eyes and keep moist.
» Communicate with child as awareness is maintained. Staff should remember that children may be very frightened but unable to express their emotions and needs.

MEDICINE TREATMENT

Substantial pain is present (in up to 90%) in the severely affected patients.
Pain in this setting is often unrecognised and underestimated.
Pain management is essential. See section 20.1: Management of pain.

For neuropathic pain**:**

- Carbamazepine, oral, 5 mg/kg/dose 12 hourly.

For rapidly progressive ascending paralysis, respiratory dysfunction or loss of ambulation:

- Immunoglobulin, IV, 1 g/kg/day, slowly over 12–16 hours on two consecutive days **or** 0.4 g/kg as a single daily dose on 5 consecutive days early in the disease process.
    - o Use under specialist supervision in an intensive care setting.

REFERRAL

» Chronic inflammatory demyelinating polyradiculoneuropathy.
» Guillain-Barré syndrome with bulbar paralysis and/or early signs of respiratory failure.
» Patients who have lost or are losing ambulation for management in consultation with a paediatric neurologist.
» Patients with complex Guillain-Barré syndrome.

13.9.2 MYASTHENIA GRAVIS

G70.0

DESCRIPTION

An auto-immune disorder resulting in muscle fatigue. Mild cases involve the eyes alone, i.e. ptosis and ophthalmoplegia, and severe cases involve proximal muscle groups, respiratory and bulbar control.

DIAGNOSTIC CRITERIA

Clinical

» Muscle fatiguability with exercise and demonstration of this in the clinic setting:
  > Lid-lag test, i.e. failure to maintain upward gaze for 1 minute.
  > Arm-raising test, i.e. failure to maintain the arms at 90° from the trunk for 1 minute
  > <u>Neostigmine test</u> (consult with neurologist):
    - Best done in a high-care environment.
    - Attach cardiac monitor.
    - Atropine at hand, in case of bradycardia.
    - Give neostigmine bromide, IM, 0.04 mg/ kg, carefully whilst watching cardiac monitor.
    - Response is not as rapid as with edrophonium.
    - Wait for 15–30 minutes to assess response.

> <u>Ice-pack eye test:</u>
  - Put ice in a plastic bag and place it over the patients affected eye(s) for 1–2 minutes.
  - Positive test is when there is some improvement of the ptosis and or the opthalmoplegia.

Note:
Myasthenia gravis patients not uncommonly present in a myasthenic crisis, with bulbar and respiratory compromise. Sometimes this may be the first mode of clinical presentation.

GENERAL AND SUPPORTIVE MEASURES

» Occupational and physiotherapy.

MEDICINE TREATMENT

- Pyridostigmine, oral, 1–5 mg/kg/day in 4–6 divided doses. (Specialist initiated)

REFERRAL

» All for confirmation of diagnosis and initiation of treatment (consideration of steroids, immuno-modulation therapy).
» Myasthenic crisis.

13.10 SYDENHAM'S CHOREA

I102.9

DESCRIPTION

Rapid involuntary jerks affecting any part of the body often incorporated into a voluntary movement in an attempt to mask it. It is an acute post-streptococcal infection movement disorder and constitutes one of the major criteria for the diagnosis of rheumatic fever. Patient has the appearance of being restless with constant movement.

DIAGNOSTIC CRITERIA

Clinical

» Exclude drug reactions, hyperthyroidism, systemic lupus erythematosus and neurodegenerative disorders.

Investigations

» Cardiac screening, i.e. ECG, echocardiogram.
» ASOT, antiDNAse.
» Erythrocyte sedimentation rate.
» dsDNA, if clinically indicated.

GENERAL AND SUPPORTIVE MEASURES

» Emotional support
» School support
» Occupational therapy

MEDICINE TREATMENT

Movement disorders:

- Haloperidol, oral, 0.025 mg/kg/day in 2–3 divided doses.
  - o Increase dose slowly and incrementally to 0.05 mg/kg/day.

PLUS

If streptococcal infection:

- Phenoxymethylpenicillin, oral, 500 mg 12 hourly for 10 days.

THEN
Until 21 years of age:

- Benzathine benzylpenicillin (depot formulation), IM, 1.2 million units every 28 days.

OR

- Phenoxymethylpenicillin, oral, 250 mg 12 hourly.

REFERRAL

» All patients for specialist assessment.

13.11 CEREBROVASCULAR DISEASE/STROKE

I67.9

DESCRIPTION

Cerebrovascular disease can be ischaemic (thrombotic or embolic) or haemorrhagic, arterial or venous.
Arterial ischaemic stroke must always be considered in any child with sudden onset of hemiparesis or other focal neurological disturbance.
The clinical features of cerebral venous thrombosis (CVT) include headache, papilloedema, focal neurological signs, seizures (often focal), and alteration of consciousness.

Risk factors:

» cardiac disorders;
» infections, e.g. meningitis, varicella, HIV, etc.;
» prothrombotic disorders, e.g.. nephrotic syndrome, protein S/C deficiencies, etc.;
» haematologic disorders, e.g. sickle cell anaemia;
» vasculopathies e.g. vasculitis, HIV, moyamoya syndrome.

The initial evaluation in children includes the following:

- » Electrocardiography, echocardiography.
- » Full blood count, INR, PTT.
- » CSF analysis.
- » Infectious screening, including varicella, HIV, mycoplasma, TB.
- » Connective tissue and vasculitic screening.
- » Thrombophilia screening. See section 3.11: Venous thrombo-embolic disease.

GENERAL AND SUPPORTIVE MEASURES

Acute supportive and neuroprotective care directed at preserving damaged but salvageable brain tissue includes the following:

- » Maintain body temperature in the low to normal range.
- » Maintain euglycaemia.
- » Maintain O_2 saturation above 95%.
- » Maintain adequate cerebral perfusion and manage raised intracranial pressure.
- » Treat anaemia.
- » Treat acute seizures promptly.

Haemorrhagic stroke requires referral to a centre with neurosurgical expertise and facilities.

Early disability assessment and management, includes physiotherapy, speech therapy, occupational therapy, etc.

MEDICINE TREATMENT

Arterial ischaemic stroke without haemorrhage

All patients with confirmed arterial ischaemic stroke:

- Aspirin soluble, oral 1–5 mg/kg as a daily dose.
  - o Contraindicated in haemorrhagic stroke or bleeding tendency.

REFERRAL

- » All patients to specialist paediatrician for investigation.
- » Anticoagulation with enoxaparin and warfarin is be st done in a specialised setting under cardiologist, haematologist and neurologist supervision.

CHAPTER 14
PAEDIATRIC PSYCHIATRY

Principles for safe and effective prescribing of psychotropic medication for children and adolescents presenting with psychiatric symptoms

» Psychopharmacology is only part of a holistic biopsychosocial treatment plan. Safe and effective pharmacological management of psychiatric disturbances in children and adolescents should always be part of a comprehensive DSM-IV multi-axial diagnostic assessment and treatment plan, by a skilled clinician.

» Psychiatric problems in this age group are complex and require a multidisciplinary approach. There should be an awareness of co-occurring medical conditions as well as of other medications used. Psychiatric comorbidity is very common and often diagnoses are difficult to make as in many instances the illness may still be evolving.

» It is important to refer to a child psychiatrist for a diagnostic assessment, when there is diagnostic uncertainty and/or a poor response to treatment.

» There should be an awareness of the side effect profile of medications prescribed, as well as potential interactions with other medications. While psychotropic medications are generally well tolerated in children they can be associated with adverse effects. It is therefore important to use the lowest dosage possible and increase slowly, monitoring for side effects.

Common drugs used in psychiatry and their side effects:

Selective serotonin reuptake inhibitors (SSRI, e.g. fluoxetine, citalopram)

Adverse effects in children and adolescents

» Agitation, behavioural disinhibition or ‘activation’, headache, GIT disturbances (decreased appetite, nausea).

» **Less common but important to note: increased risk of suicidal thoughts or acts, associated with use of certain SSRIs in depressed children and adolescents (e.g. paroxetine).*

» **Less common but potentially serious side effect is Serotonin Syndrome, which presents (in increasing severity) as restlessness, tremor, shivering, myoclonus, confusion, convulsions and death.*

Special precautions/ investigations/monitoring

» Adverse events may be dose related, reduce where indicated.

» Check for :
  - > suicidal ideation,
  - > ‘manic switch’ (may precipitate mania in bipolar illness),
  - > symptoms of Serotonin Syndrome in patients where dosages have been exceeded or in whom there is simultaneous use of two SSRIs (‘cross-tapering’).

Tricyclic Antidepressants (e.g. amitriptyline)

<u>Adverse effects in children and adolescents</u>

» Sedation, anticholinergic, cardiac side effects.
» May be more cardiotoxic in children than in adults.

<u>Special precautions/ investigations/monitoring</u>

» Dangerous and potentially fatal in overdose. Avoid in children with pre-existing cardiovascular disease.
» Do not use in conjunction with other drugs which prolong the QT interval.
» Baseline and on-treatment ECGs should be performed in patients with pre-existing cardiovascular condition or positive family history.
» May precipitate mania in bipolar illness.

Stimulant medicines (e.g. methylphenidate)

<u>Adverse effects in children and adolescents</u>

» Loss of appetite, weight loss, insomnia, headache, abdominal pain.
» Dysphoria or depressed mood (higher doses).
» May worsen existing tics.
» May, at higher doses, lower seizure threshold and precipitate seizures in children suffering from epilepsy.

<u>Special precautions/ investigations/monitoring</u>

» Monitor BP, pulse, height and weight.
» Monitor for mood change and development of tics.
» Use with caution in children who suffer a psychotic illness.

'Atypical' antipsychotics (e.g. risperidone, olanzapine)

<u>Adverse effects in children and adolescents</u>

» Weight gain, metabolic syndrome, extrapyramidal side-effects (EPSE) such as acute dystonia and parkinsonism, akathisia and sedation at higher dosages (particularly risperidone).

<u>Special precautions/ investigations/monitoring</u>

» Monitor weight.
» Check prolactin level, glucose and cholesterol, particularly if family history.

'Typical' neuroleptics (e.g. haloperidol)

<u>Adverse effects in children and adolescents</u>

» EPSEs, acute dystonia, akathisia, tardive dyskinesia.
» *Life threatening side effect is Neuroleptic Malignant Syndrome (NMS) (fever, altered mental status, muscle rigidity, autonomic dysfunction).*

<u>Special precautions/ investigations/monitoring</u>

» Monitor for EPSEs.
» Avoid long term use as risk of tardive dyskinesia, which is irreversible.

Benzodiazepines (e.g. lorazepam, diazepam, clonazepam)

<u>Adverse effects in children and adolescents</u>

» Sedation, anomalous reaction of disinhibition, restlessness.

Special precautions/ investigations/monitoring

» Not for long term use.

14.1 SEDATION OF ACUTELY DISTURBED CHILD OR ADOLESCENT AWAITING PSYCHIATRIC EVALUATION

MEDICINE TREATMENT

Exclude organic causes, e.g. encephalopathy or other intracranial pathology, seizures, metabolic disease, medication adverse effects and intoxication.

For children under the age of six years

Sedation with psychotropic agents should only be considered in extreme cases and only after consultation with a specialist.

For children over the age of six years

- Lorazepam, oral/IM, 0.05–0.1 mg/kg/dose.
    - Onset of action: is 20–30 minutes.

If sedation is inadequate:

- Haloperidol, IM, 0.025–0.075 mg/kg/dose.

In case of an acute dystonic reaction secondary to haloperidol:

- Biperiden, IM/slow IV, 0.05–0.1 mg/kg.
    - 6–10 years: 3 mg
    - >10 years: 5 mg

14.2 ATTENTION DEFICIT HYPERACTIVITY DISORDER (ADHD)

F90.0/90.1

DESCRIPTION

Children with ADHD display developmentally inappropriate degrees of inattention, impulsiveness and hyperactivity.

There are 3 subtypes:

» predominantly inattentive,

» predominantly hyperactive-impulsive, and

» combined.

Inattention:

» Failing to give close attention to details,

» Careless mistakes,

» Not listening,

» Failure to complete tasks,

» Losing things,

» Distractibility.

Hyperactivity:

- » Fidgetiness.
- » Out of seat.
- » Running or climbing excessively.
- » Being "on the go", or like "driven by a motor".

Impulsivity:

- » Blurting out answers.
- » Difficulty waiting turn.
- » Interrupting or intruding on others.

Outcome

- » Hyperactivity symptoms decrease and inattention symptoms persist during adolescence.
- » Remission is achieved in up to two thirds (30–60%) of patients during adolescence.
- » Symptoms persist into adulthood in the remaining one third.
- » Symptom presentation in adults reflects persistence of problems with attention and concentration, as well as poor impulse control.
- » Common adult difficulties include problems in the workplace, relationship problems, high-risk behaviours including substance misuse as well as as sociated co-morbid conditions such as depression and anxiety.

DIAGNOSTIC CRITERIA

- » Diagnostic criteria are based on DSM IV.
- » Symptoms must present before age seven.
- » Symptom duration of at least six months.
- » Behaviour is inconsistent with the patient's developmental level and intellectual ability.
- » Presence of functional impairment in more than one setting.
- » Exclude other mental or physical disorders, e.g. anxiety disorders, mood disorders, psychotic disorders, hearing impairment.
- » Common co-morbid conditions include Oppositional Defiant Disorder, Conduct Disorder, Depression (particularly in girls) and Substance Use Disorders (SUDs), as well as HIV and epilepsy.
- » Certain conditions may 'mimic' ADHD s uch as, developmental disorders, motor coordination problems, intellectual disability, post-traumatic and post infectious encephalopathy.

Note:

- » Girls may more commonly present with inattentive-type ADHD. The diagnosis may therefore be missed.

GENERAL AND SUPPORTIVE MEASURES

- » Identify and treat comorbidities early, such as depressive disorders as this may prevent the onset of substance misuse (to 'self-medicate') and other risk behaviours during adolescence.

» Parent counseling:
  > rules and limit-setting,
  > positive reinforcement of pro-social behaviour,
  > consistent routine, and
  > restrictive diets are of no proven value.
» Behaviour-based therapy:
  > reward positive behaviour,
  > improve self-image, and
  > improve social awareness and adjustment.
» Social skills groups.
» Identify learning difficulties and refer to educational support services.

MEDICINE TREATMENT

There is no absolute contraindication for the concomitant use of methylphenidate with antiepileptic drugs (AEDs) or antiretroviral therapy (ART). However, exercise caution with the dosages prescribed and beware of potential drug-drug interactions. Monitor for potential adverse side effects.

For children under the age of six years
Refer for diagnostic assessment by a child psychiatrist or paediatrician.

For children over the age of six years
Initiate treatment using the short-acting methylphenidate formulation until effective dosage achieved. Reduce dose or withdraw methylphenidate if a paradoxical increase in symptoms occurs.

- Methylphenidate, short -acting, oral, 1 mg/kg/day (maximum 2 mg/kg)
  - o Initial dose: 5 mg 2–3 times daily, before breakfast and lunch, 3rd dose not later than 14h30 (approximately every 3 to 3½ hours).
  - o Increase the dose at weekly intervals by 5–10 mg until symptoms are controlled. Use the lowest effective dose.
  - o Maximum daily dose: 60 mg. Do not exceed 40 mg/day without consultation with a child psychiatrist or paediatrician.

If inadequate symptom control due to irregular usage or poor adherence, refer.

Contraindications:
Absolute**:**
» Hyperthyroidism,
» cardiac dysrhythmia,
» glaucoma,
» concomitant mono-amine oxidase inhibitor therapy.

Relative**:**
» hypertension,
» anxiety,
» agitation,
» epilepsy,
» tics.

Discontinuation of treatment

» If no objective improvement of symptoms is observed, e.g. Conners' Scales completed by teacher, after appropriate dosage adjustment over a one-month period.
» To establish whether on-going treatment is indicated in a child on long term stimulant therapy, trials of discontinued treatment (trials off treatment) should be part of the management plan.
» Indications for a trial off treatment:
  > treatment duration in excess of 2-3 years,
  > adolescent age (particularly late adolescence), and
  > a substantial reduction in core ADHD symptoms, evident in more than one setting.
» Trials off treatment should be planned and at times least disruptive to the child's academic and social functioning i.e. time the treatment withdrawal outside of major commitments such as examinations.
» Duration of treatment withdrawal can be for one week to a month, depending on whether stability is maintained.
» Treatment can be withdrawn abruptly, with no need to taper dosages.
» Obtain feedback from teachers and parents (verbal feedback, completion of parent and teacher Conners' Scales), before and during the trial off treatment.
» Assess the child and document the mental state (symptoms of ADHD), before and during the trial off treatment.
» Monitor 3 monthly for one year.
» Re-initiate treatment (at last dosage prescribed), if:
  > there is a significant re-emergence of symptoms after one week off treatment and/or during the month off medication, or
  > after a longer trial off medication, e.g. at 3 monthly follow up visits, there is evidence of symptom re-emergence.

Note:
Adolescents are more likely to present with poor concentration and inattentiveness, rather than hyperactivity.

REFERRAL

» No response to medicine treatment after 4–6 weeks.
» Presence of comorbid psychiatric conditions with severe functional impairment: oppositional defiant disorder, mood disorders, anxiety disorders, debilitating tics.
» Presence of uncontrollable seizures.
» HIV positive status.

14.3 MOOD DISORDERS

F30–F39

14.3.1 DEPRESSION IN CHILDHOOD AND ADOLESCENCE

F32

DESCRIPTION

The clinical presentation of depression includes:

- » symptoms of depressed mood,
- » decreased pleasure or interest,
- » neurovegetative symptoms,
- » sleep/appetite disturbance,
- » fatigue,
- » poor concentration,
- » psychomotor agitation/retardation,
- » guilty ruminations, and
- » thoughts of death and suicide.

Suicide is self-inflicted harm where the intention is to die.
Increased suicide risk is associated with the following:

- » male gender
- » adolescence
- » previous attempts and lethality of method used
- » family history of suicide
- » presence of a mental illness
- » social isolation and poor family support
- » associated substance abuse or physical aggression

The clinical picture of a child and adolescent with major depressive disorder is similar to that of adults except that there are some developmental differences i.e.:

- » mood is often irritable rather than sad,
- » somatic complaints, i.e. headache and abdominal pain,
- » behavioural and academic/school problems occur frequently in children,
- » withdrawal from social activities,
- » neurovegetative symptoms are less common than in adults, and
- » suicide attempts increase in number, tend to be more lethal and impairment of functioning worsens, with increasing age.

The first ep isode of bipolar disorder can present with depression in adolescents. Bipolar depression is often associated with a more sudden onset, psychomotor retardation and in some instances, psychotic symptoms.

A number of depressed children and adolescents have co-morbid psychiatric disorders. The most frequent co-morbid diagnoses are:

- anxiety disorders,
- ADHD,
- oppositional defiant disorder,
- conduct disorder, and
- substance misuse, particularly in adolescents.

Conduct problems may develop as a complication of the depression and persist after the depression remits. It is important to assess and manage conditions that occur together with depression.

DIAGNOSTIC CRITERIA

- The presence of at least five of the symptoms of depression for a period of up to two weeks.
- Symptoms should be of a severity to cause significant functional impairment and feelings of distress.
- Diagnosis is difficult in children, compared to adolescence, as children may 'deny' depressed mood or may not experience symptoms as 'ego dystonic'. Some children may require diagnostic assessment and treatment in an in-patient setting.

Consider the following in a child presenting with depressed mood:

- Exclude underlying medical conditions such as:
  - infections, e.g. HIV, cerebral cysticercosis, encephalitis and tuberculous meningitis;
  - neurological conditions, e.g. temporal lobe epilepsy, brain tumours;
  - endocrine disorders, e.g. thyroid conditions.
- Exclude medication-induced mood disturbances e.g. corticosteroids, antiretrovirals (zidovudine, efavirenz), high doses of stimulant medication and barbiturates.
- Exclude substance abuse such as alcohol and methamphetamine ('Tik').
- Assess for suicide risk.

GENERAL AND SUPPORTIVE MEASURES

- Psychological interventions are considered 'first line' for mild to moderate depression and should be administered by a suitably skilled clinician:
  - cognitive behavioural therapy (CBT): to address distorted, negative cognitions, maladaptive patterns of behaviour and communication;
  - psychodynamic/play therapy: to identify feelings, improve self esteem and social interactions.

» Additional interventions:
  > family counselling: to address family disharmony, stressors and provide psycho-education;
  > input to school: to address academic issues and psycho-education;
  > social worker: to investigate suspicion of child abuse or neglect.

MEDICINE TREATMENT

If there is a failure to respond to psychotherapeutic interventions after 4–6 weeks or if severity of symptoms increases, consider a trial of antidepressant medication, while still continuing with psychotherapy. Initiate treatment in consultation with a specialist.
Response to treatment should bring about a meaningful reduction in symptoms and improvement in functioning.
Once remission is achieved continue medication therapy for at least a further 6–12 months.

- Fluoxetine, oral, 0.5 mg/kg/day.
  - Dose range: 5–40 mg daily.
  - Recommended average dose: 10–20 mg/day.

If there is a poor response to fluoxetine after an adequate trial of treatment, i.e. 4–6 weeks; or if significant symptoms of anxiety are present; child is HIV positive; consider an alternative SSRI, in discussion with a child and adolescent psychiatrist:

- Citalopram, oral, 0.4 mg/kg/day.
  - Dose range: 10–60 mg daily.
  - Recommended average dose: 10–20 mg/day.

Note:
Be aware of potential side effects, discussed above.

A trial of treatment is considered to be ineffective if the patient presents with ongoing, significant depressive symptoms and/or suicidal ideation and where the patient has not achieved an improvement in overall level of functioning.

Be aware of the risk of bipolar 'switch' or precipitation of mania in patients with a family history of bipolar disorder.

Tricyclic antidepressants are not recommended as first line agents in children, due to insufficient evidence of efficacy, and potentially adverse cardiovascular side effects and lethality in overdose.

REFERRAL

» Poor response to an adequate trial of treatment i.e. medication trial of 6–8 weeks in combination with psychological treatment.
» Presence of co-morbid conditions.
» Psychotic symptoms such as delusions or hallucinations.
» Suicidal ideation or intent.

14.3.2 DISRUPTIVE MOOD DYSREGULATION DISORDER (DMDD)

F31

DESCRIPTION

Disruptive mood dysregulation disorder (Bipolar disorder) in children often presents with mixed mood states, with significant mood lability, rage attacks or 'affective storms', rather than discrete manic or depressive episodes. The picture therefore may not resemble the clinical picture observed in adults.

There is a risk of misdiagnosis or 'over-diagnosis' of this disorder, in children presenting with severe aggression and 'dysregulated' moods.

Manic Episode

A distinct period of an ab normally and pers istently elevated, expansive and/or irritable mood. This should represent a si gnificant change in t he patient's baseline mental status and must last for at least 1 week. During the period of mood disturbance, the pa tient should display the foll owing symptoms:

» Elated mood.
» Grandiosity.
» Decreased sleep.
» Hypersexuality.
» Racing thoughts and pressured speech.
» Increase in goal-directed activity.

Depressive episode

Similar to symptoms of major depressive episode except that onset may be more rapid, associated with psychomotor retardation, and/or psychotic symptoms.

Mixed mood state

Presence of both manic and depressive symptoms over a period of 1 week. This presentation is more common in children. Discrete manic and depressive phases are less evident than in adults.

The mood disturbance must cause marked impairment of functioning and should not be due to the direct effects of a substance.

MEDICINE TREATMENT

Refer patient with suspected manic episode immediately for spec ialist assessment and admission for containment and further management.

CAUTION

Beware of the ris k of ne uroleptic malignant syndrome, especially with the use of high doses in the first episode.

Acute treatment
Sedate before transfer. See section 14.1: Sedation of acutely disturbed child and adolescent awaiting psychiatric evaluation.

Maintenance treatment
If previously on maintenance therapy:
- Re-initiate treatment, in consultation with a psychiatrist.

If not previously on maintenance therapy:
- Risperidone, oral, 0.5–3 mg daily, depending on age, weight of child and clinical response. (Specialist initiated, or in consultation with a psychiatrist)
- Other medication such as mood stabilisers only to be initiated in consultation with a psychiatrist.

REFERRAL
- All for diagnostic assessment by a child and adolescent psychiatrist.

14.4 ANXIETY DISORDERS
F41.9

Separation Anxiety Disorder and Selective Mutism are diagnostic categories exclusive to the childhood population, while Post-Traumatic Stress Disorder (PTSD), Obsessive Compulsive Disorder (OCD), Social Phobia, Specific Phobia, Panic Disorder and Generalised Anxiety Disorder (GAD) present across the lifespan.

Medicine treatments do not form part of the primary management of Separation Anxiety Disorder and Selective Mutism, unless the child presents with additional symptoms which merit a diagnosis in one of the other anxiety disorder categories.

Anxiety in a child can be misdiagnosed as ADHD, as both conditions may present with increased levels of activity and problems with concentration.

Symptoms	ADHD	Anxiety
Hyperactivity	+	+
Inattention	+	+
Impulsivity	+	– (fearful, inhibited)
Emotional content/thoughts	» poor school performance,<br>» ‘in trouble’ with adults,<br>» peer rejection/social isolation,<br>» struggles to control aggression.	» fear,<br>» threat,<br>» worry,<br>» nightmares with anxiety themes.
Academic difficulties	More likely	Less likely
Poor sleep	+	+

14.4.1 POST TRAUMATIC STRESS DISORDER (PTSD)

F43.1

DESCRIPTION

The core features of experiences which place patients at risk of PTSD are:

» exposure to any traumatic event’
» there is a threat of serious injury or death,
» feelings of intense fear, helplessness and horror.

DIAGNOSTIC CRITERIA

According to DSM IV:

» Persistently re-experiencing:
  > recurrent thoughts, dreams,
  > flashbacks, reliving experiences.
» Marked avoidance (conversations, places):
  > numbing,
  > diminished interest,
  > detachment or restricted emotions.
» Hyperarousal (hypervigilance, poor sleep).
» Significant distress/impairment.

GENERAL AND SUPPORTIVE MEASURES

Debriefing in the immediate aftermath of the trauma is not recommended.
Psychological interventions, including:

» general supportive counseling,
» cognitive behavioural strategies,
» group and family interventions.

MEDICINE TREATMENT

- Fluoxetine, oral, 0.5 mg/kg/day.
  - o Recommended average dose: 5–20 mg.
  - o If poor response, consider higher doses in consultation with a specialist.

REFERRAL

» Persistent symptoms despite therapy.

14.4.2 GENERALISED ANXIETY DISORDER (GAD)

F41.1

DESCRIPTION

Excessive anxiety or worry, occurring on most days for at least 6 months.

DIAGNOSTIC CRITERIA

According to DSM IV, presence of 3 of the following:

- » restlessness or a feeling of being ‘on edge’,
- » poor concentration or ‘mind going blank’,
- » irritability,
- » muscle tension,
- » sleep disturbance,

GAD causes significant distress and impairment in functioning.
Exclude substance abuse or a medical condition.

GENERAL AND SUPPORTIVE MEASURES

These interventions should be performed by a suitably qualified clinician.

- » Cognitive behavioural therapy (CBT): aimed at changing pessimistic, anxiety-based cognitions and developing strategies to reduce anxieties and avoidant behaviour patterns.
- » Behaviour therapy: relaxation, desensitisation by imagining or exposure to anxiety-provoking situations.
- » Psychodynamic/supportive psychotherapy: aimed at promoting self esteem, assertiveness and autonomy.

MEDICINE TREATMENT

- Fluoxetine, oral, 0.5 mg/kg/day.
  - o Dose range: 5–40 mg daily.
  - o Recommended average dose: 10–30 mg.
  - o Treatment duration: 6–9 months after resolution of symptoms.

REFERRAL

- » Failure to respond after 6–8 weeks to an adequate trial of therapy and medication.
- » Adverse events with fluoxetine.

14.4.3 OBSESSIVE COMPULSIVE DISORDER (OCD)

F42.9

DESCRIPTION

<u>Obsessions</u>

Persistently recurring thoughts, impulses or images that are experienced as intrusive, inappropriate and not simply excessive worries about realistic problems. Children may not experience these as distressing but the obsessions may interfere with day-to-day functioning.

<u>Compulsions</u>

Repetitive behaviours or mental acts that a person feels driven to perform according to a rigidly applied rule in order to reduce distress or to prevent some dreaded outcome.

DIAGNOSTIC CRITERIA

According to DSM IV:

- The most common symptoms of OCD in childhood are:
  - contamination fears accompanied by compulsive washing and avoidance of "contaminated" objects,
  - repetitive checking and counting,
  - obsessive doubt,
  - compulsive reading and drawing,
  - symmetry concerns (e.g. right and left needs to be same).
- Comorbid conditions:
  - rheumatic fever,
  - streptococcal throat infection (paediatric autoimmune neuropsychiatric disorders associated with streptococcal infections (PANDAS)),
  - tic disorders.

GENERAL AND SUPPORTIVE MEASURES

- Provide Cognitive Behavioural Therapy (CBT), if available and appropriate.
- Exposure-based interventions, e.g. contact with "dirt" in a child with contamination fears, thought stopping techniques, "response prevention", i.e. blocking of rituals.

These interventions should be carried out by a suitably qualified professional.

MEDICINE TREATMENT

- Fluoxetine, oral, 0.5 mg/kg/day.
  - Dose range: 10–40 mg daily. Higher doses may be needed to effectively manage OCD symptoms.
  - Recommended average dose: 20–40 mg. Higher dose within the range may be required.
  - Duration of treatment: 6 months after resolution of OCD symptoms.

REFERRAL

- Poor response to adequate trial of cognitive behavioural therapy and medication i.e. persistence of obsessions and/or compulsions, with impairment in functioning.
- Co-morbid conditions.

14.5 CHILDHOOD PSYCHOSIS

F09

It is important to note that children who present with symptoms such as hallucinations, confusion and intensely aggressive or disturbed behavior may not all be psychotic or suffer from schizophrenia. Delirium should be the first diagnosis to consider, before a psychotic disorder is suspected. Failure to recognise a delirium may delay the diagnosis of the underlying medical condition or drug-related delirium and place the child at risk.

Delirium is a non-specific neuropsychiatric disorder which indicates global encephalopathic dysfunction in medically ill patients. The core features consist of attentional disturbances, an altered level of consciousness and diffuse cognitive deficits. It is fluctuating in nature and may present with perceptual disturbances, commonly visual hallucinations.

14.5.1 SCHIZOPHRENIA

F20.9

DESCRIPTION

Schizophrenia is a chronic psychotic disorder characterised by disturbances in thinking, perceptions, emotions and behavior, associated with significant degrees of impairment in functioning. Childhood Schizophrenia is rare. All children presenting with psychotic features merit further investigation.

- » Very Early Onset Schizophrenia (VEOS) is defined as onset before age 13 years.
- » Early Onset Schizophrenia is defined as onset before age 18 years.

Onset during childhood and adolescence confers a poorer prognosis for the illness, treatment refractoriness and significant impairment in functioning.

DIAGNOSTIC CRITERIA

According to DSM IV:

- » Two or more of the following symptoms need to be present for at least 6 months:
  - > delusions,
  - > hallucinations,
  - > disorganised speech,
  - > grossly disorganised or catatonic behaviour,
  - > ‘negative’ symptoms i.e. affective flattening and lack of volition.
- » The same diagnostic criteria for adults are used, but in children, delusions are not as bizarre or systematised as in adults. The clinical presentation in adolescents more closely resembles that in adults.
- » Exclude substance abuse (cannabis, methamphetamine) or a medical condition in a young person presenting with a psychotic episode.

GENERAL AND SUPPORTIVE MEASURES

- » Supportive individual and family counseling is an important part of the comprehensive treatment plan.
- » The aim of individual counseling is to develop healthier coping strategies and defence mechanisms and to provide structure and limit regression.
- » Family interventions focus on psycho-education, facilitating acceptance of the diagnosis to ensure adequate compliance and support for the patient.
- » Educational issues must be attended to such as transition back to school after a psychotic episode and academic support.

MEDICINE TREATMENT

Pharmacotherapy is the first line treatment for psychosis in children and adolescents.

Acute phase treatment (4–6 weeks)

Sedate before transfer. See section 14.1: Sedation of acutely disturbed child and adolescent awaiting psychiatric evaluation.

If previously prescribed antipsychotic medication:

- Re-initiate treatment, in consultation with a psychiatrist.

If not previously on therapy, while awaiting admission:

- Risperidone, oral.
  - o Initial dose: 0.5 mg daily.
  - o Use lowest effective dose to limit adverse long term side effects and to facilitate adherence.
  - o Increase dose by 0.25–0.5 mg daily every 1–2 weeks, depending on tolerability and age of the child.
  - o Refer if doses in excess of 3 mg are required.

Maintenance phase: (12–24 months)

Gradually lower the dose of risperidone from that needed to treat the acute psychotic phase to that needed to prevent relapse and to ensure adequate adherence.

Note:

» First generation or 'typical' antipsychotics (e.g. haloperidol) and second generation or 'atypical' antipsychotics (e.g. risperidone) have demonstrated equal efficacy for the treatment of psychotic symptoms.

» First generation antipsychotics are less favourable because of high rates of extrapyramidal side effects and worsening of negative symptoms in children and adolescence with long term use compared to adult populations.

» Exercise caution when prescribing high doses of haloperidol as this may increase the risk of neuroleptic malignant syndrome (NMS), which may be life threatening.

» In a patient with an identified cardiovascular risk, do an ECG before initiating therapy with risperidone.

» Monitor blood pressure, pulse, ECG, blood glucose and lipid profile in patients on long term treatment.

REFERRAL

» All children for assessment and for initiation of therapy

» Urgent: younger children

» Urgent: for admission, of behaviourally disturbed psychotic adolescent.

14.5.2 TIC DISORDERS

F95.9

DESCRIPTION

A tic is a sudden, rapid, recurrent, non-rhythmic stereotyped motor movement or vocalisation and includes the following subtypes:

» Chronic motor or vocal tic disorder.
» Transient tic disorder.
» Tourette's disorder.

Tourette's disorder is a chronic neuropsychiatric disorder that is characterised by both vocal and motor tics, and related somatosensory urges. It is commonly associated with a number of co-morbid conditions such as OCD, ADHD as well as disturbances of mood.

GENERAL AND SUPPORTIVE MEASURES

» Psycho-education of patient, parents, teachers and peers: to reduce the stigma and social consequences of tics.
» Supportive psychotherapy: to assist the individual to cope with the stigma/ teasing, improve self esteem and improve social skills.
» Family therapy: to assist the family in managing associated symptoms and to reduce stress.

MEDICINE TREATMENT

Medication to be used for short periods only to reduce severe symptoms. Haloperidol may be initiated as first line since medication treatment is not long term.

Note:
The natural course of tics is to 'wax and wane' or fluctuate. Therefore avoid long term use of neuroleptic medication.

For severe and frequent tics that seriously impact on child's functioning:

- Haloperidol, oral, 0.02–0.12 mg/kg/day.
  - Dose range: 0.25–1.75 mg 12 hourly.
  - Recommended average dose: 0.5–2 mg/day.
  - Monitor for extrapyramidal and anticholinergic side effects.

If poor response:

- Risperidone oral, 0.5 mg/day.
  - Dose range, 0.5–3 mg/day.
  - Recommended average dosage 1mg/day.

REFERRAL

» Tourette's syndrome not responding to therapy.
» Tourette's syndrome with comorbid psychiatric or medical conditions.

14.6 OTHER SITUATIONS OR CONTEXTS IN WHICH PSYCHOTROPIC DRUGS MAY BE PRESCRIBED

The following are particular conditions or common behaviours for which psychotropic medication forms part of the management plan.
These are:
» Psychiatric presentations associated with paediatric HIV.
» Behavioural problems associated with pervasive developmental disorders (PDDs).
» Substance use disorders in adolescence.
» Behavioural problems associated with Intellectual Disability (ID).

14.6.1 PSYCHIATRIC PRESENTATIONS IN HIV INFECTED CHILDREN AND ADOLESCENTS

DESCRIPTION
» HIV positive children and adolescents are at increased risk of psychopathology, such as ADHD, depression and anxiety disorders. Psychosis and mania is less common than in the adult population.
» The increased risk of psychopathology is due to the virus itself, side effects of antiretroviral therapy (ART) and those due to psychosocial stressors.
» Symptom presentation of psychiatric disorders in HIV positive children are the same as in the general childhood population.
» ADHD often co-occurs with significant learning difficulties, despite treatment with antiretroviral therapy (ART).
» Psychotic disorders are rare in HIV positive children. Consider a delirium or partial seizures if an HIV positive child presents with psychotic symptoms.

GENERAL AND SUPPORTIVE MEASURES
» Psychological interventions are similar to those for HIV negative children.
» Issues specific to the child's HIV status may need specific intervention, for problems related to disclosure of HIV status, stigma, grief counselling, adherence issues, orphanhood and living with a chronic illness.
» Refer to the hospital social worker to address social issues.

MEDICINE TREATMENT
Start all medications at lower doses and then titrate up slowly.
Initiate treatment according to guidance in this chapter.

Note:
Because of drug-drug interactions between fluoxetine and some antiretroviral medication, initiate treatment with citalopram.

REFERRAL

» All HIV positive children on ART who present with severe psychiatric symptoms such as severe depression, psychosis and/or mania for psychiatric evaluation and initiation of psychotropic medication.

14.6.2 PERVASIVE DEVELOPMENTAL DISORDERS (PDDS)

F84

DESCRIPTION

The Pervasive Developmental Disorders (PDDs) are neuropsychiatric disorders characterised by patterns of delay and deviance in the development of social, communication and cognitive skills. Onset is usually during the first few years of life and includes conditions such as:

» Autistic disorder.
» Asperger's disorder.
» Rett disorder.
» Childhood disintegrative disorder.

GENERAL AND SUPPORTIVE MEASURES

» Social skills and family interventions.
» Education and social placement.
» Behaviour modification, specifically adapted for autism spectrum disorders.
» Early intervention is important for optimal outcome.

MEDICINE TREATMENT

Not for core autistic symptoms.

For severe aggression and self-injurious behavior:

- Risperidone, oral, 0.125–3 mg daily in 2 divided doses.
  - o Average daily dose: 0.25–2 mg/day.

OR

- Haloperidol, oral, 0.002–0.12 mg/kg/day.
  - o Average daily dose: 0.25–2 mg/day.
  - o Dose range: 0.25–1.75 mg 12 hourly.
  - o Monitor for extrapyramidal and anticholinergic side effects.

14.6.3 SUBSTANCE ABUSE

F10–19

DESCRIPTION

Age of onset of substance abuse can be as early as 8 years.

Illicit drugs such as cocaine, amphetamines and cannabis, as well as alcohol abuse are associated with a greater risk for psychosis.

Behavioural disturbance in the context of a Substance Use Disorders (SUD) may be due to intoxication, withdrawal, or due to a substance induced mood or psychotic disorder.

Initial treatment of SUDs begins with medical stabilisation of the patient ideally in a medical facility.

About one third of youth with SUDs, present with a 'dual diagnosis', which is the presence of a co-occurring psychiatric disorder and SUD, in the same individual.

Be aware of the mental state changes associated with illicit drugs.

Drug	Symptoms of intoxication and physical signs	Withdrawal symptoms	Withdrawal duration	Time detectible in the urine (days)	Other substances which may give a positive result	Mental state examination (MSE)
**Amphetamine (Meth-amphetamine) (Tik)**	» raised BP,<br>» hyperthermia,<br>» tachycardia,<br>» tremor,<br>» agitation,<br>» anorexia.	» depression,<br>» hunger,<br>» excessive fatigue.	peaks at 7–34 hours<br>Duration: 5 days.	1–2	cough syrups and decongestants	» hallucinations (visual, auditory, tactile),<br>» elevated mood,<br>» paranoid ideation,<br>» impaired concentration.
**Cannabis (‘dagga’)**	» hypotension (postural)<br>» tachycardia,<br>» inco-ordination,<br>» blood-shot eyes.	» irritability,<br>» restlessness,<br>» anxiety,<br>» poor sleep.	depends on heaviness of use.<br><br>Up to one month and longer, if heavy use.	2–8 (acute)<br><br>14–42 (chronic use)	efavirenz	» psychosis,<br>» mania,<br>» perceptual disturbance,<br>» impaired memory and judgement.<br>Note: *2x increased risk of developing schizophrenia*

Drug	Symptoms of intoxication and physical signs	Withdrawal symptoms	Withdrawal duration	Time detectible in the urine (days)	Other substances which may give a positive result	Mental state examination (MSE)
**Cocaine**	» raised BP,<br>» tachycardia,<br>» headache,<br>» chest pain,<br>» tachypnoea,<br>» respiratory depression.	» decreased level of consciousness,<br>» lethargy.	12–18 hours	7–14	» codeine,<br>» ephedrine,<br>» pseudoephedrine.	» mania,<br>» psychosis,<br>» panic or anxiety,<br>» poor sleep,<br>» hyperarousal.
**Heroin**	» respiratory depression,<br>» pinpoint pupils,<br>» clammy skin.	» muscular aches,<br>» runny nose,<br>» diarrhoea,<br>» nausea,<br>» 'gooseflesh'.	1–2	1–2	» opiate analgesics,<br>» procaine,<br>» naltrexone.	» euphoric mood,<br>» hallucination,<br>» drowsiness.
**Methadone**	» pulmonary oedema,<br>» respiratory depression.	As above but milder, lasts longer.	peaks at 4–6 days, can last for 3 weeks.	up to 7 days with chronic use	» imipramine,<br>» pethidine, chlorpheniramine (high doses), cetirizine.	As above.

Drug	Symptoms of intoxication and physical signs	Withdrawal symptoms	Withdrawal duration	Time detectible in the urine (days)	Other substances which may give a positive result	Mental state examination (MSE)
**Methaqualone (mandrax)**	» slurred speech,<br>» poor coordination.<br>» nausea, vomiting,<br>» abdominal pain.	» anxiety,<br>» restlessness,<br>» nausea and vomiting,<br>» abdominal cramps,<br>» tremors,<br>» headache,<br>» seizures.	peaks at day 2–3	3–5 days		» elation,<br>» euphoria,<br>» aggression,<br>» mood swings,<br>» depressed mood,<br>» excessive sleep.
**Inhalants** (glue, petrol, 'thinners', varnish remover, methylated spirits)	» red face,<br>» inco-ordination,<br>» slurred speech.	No abstinence syndrome.	N/A	Not detectable.	N/A	» disinhibition,<br>» aggression,<br>» apathy,<br>» stupor,<br>» psychotic symptoms,<br>» dementia
	Note: Risk of cardiac arrhythmias, respiratory depression, neuropathy, cerebellar damage, deafness, dementia, leukaemia associated with chronic abuse of inhalants.					

Drug	Symptoms of intoxication and physical signs	Withdrawal symptoms	Withdrawal duration	Time detectible in the urine (days)	Other substances which may give a positive result	Mental state examination (MSE)
**Alcohol**	» slurred speech,<br>» poor coordination,<br>» drowsiness,<br>» agitation.	» restlessness,<br>» tremor,<br>» sweats,<br>» anxiety<br>» severe (delirium tremens),<br>» confusion,<br>» disorientation,<br>» agitation,<br>» tachycardia,<br>» raised BP,<br>» visual and auditory hallucinations.*<br>*rare in children and adolescents	peaks 24–48 hours<br><br>duration: 1 week to 10 days	Not detectable	N/A	» agitation,<br>» irritability,<br>» confusion,<br>» hallucinations,<br>» paranoid ideation.

DIAGNOSTIC CRITERIA (according to DSM IV-TR)

Substance abuse

- » recurrent use resulting in a failure to fulfil major obligations,
- » substance related legal problems,
- » persistent use of substances in physically hazardous situations,
- » ongoing use despite negative social or interpersonal consequences.

Substance dependence

- » substantial involvement with illicit substances (daily use, increasing frequency and amount, school dropout or failure, social withdrawal),
- » loss of control (unsuccessful efforts to cut down or control use),
- » withdrawal and tolerance.

Substance induced psychotic disorder

- » prominent hallucination or delusions,
- » symptoms occur during or within one month of proven substance abuse or intoxication,
- » a mental illness such as schizophrenia or a medical condition is not the cause of the psychosis,
- » the disturbance does not occur in the course of a delirium, which must be excluded.

Substance induced mood disorder

- » a significant and sustained disturbance in mood i.e. depressed, irritable, expansive or elevated,
- » symptoms occur during or within one month of proven substance abuse or intoxication,
- » a mental illness such as bipolar disorder or a medical condition is not the cause of the mood disturbance.

GENERAL AND SUPPORTIVE MEASURES

- » Conduct a me dical assessment (pulse, temperature, BP, ECG) and laboratory investigations (FBC, U&E, LFTs, urine toxicology), depending on the specific drug of abuse.
- » Manage withdrawal states, depending on substance of abuse.
- » Refer to the hospital social worker for evaluation of family circumstances and brief motivational interviewing.

MEDICINE TREATMENT OF WITHDRAWAL STATES

Consult with a psychiatrist or specialised referral unit.

Alcohol

Management of withdrawal:

- Diazepam, oral, 5–15 mg daily in 2–3 divided doses.
    - o Taper dose over 3–5 days.

Cannabis/ cannabis and mandrax
Management of withdrawal states is rarely required.

If symptomatic withdrawal:

- Diazepam, oral, 5–15 mg daily in 2–3 divided doses.
  - Taper dose over 3–5 days.

Stimulants (cocaine, methamphetamine)
Management of withdrawal states is rarely required.

If symptomatic withdrawal:

- Diazepam, oral, 5–15 mg daily in 2–3 divided doses.
  - Taper dose over 3–5 days.

Opioids
Mild withdrawal: symptomatic treatment:

- Diazepam, oral, 2.5–5 mg in four divided doses.
  - Taper off over 5–7 days for anxiety, cramps, agitation and cravings.
  - < 14 years: maximum dose – 10 mg/day.

Symptomatic treatment for stomach cramps, diarrhoea, muscle pain and flu-like symptoms.

Moderate to severe withdrawal:

- Admit to a medical facility.

Hallucinogens
No detoxification indicated.

Volatile Solvents
No detoxification indicated.

MEDICINE TREATMENT OF COMORBID PSYCHIATRIC CONDITIONS

- Treat according to the primary psychiatric condition, as per treatment guidelines. See section 14.1: sedation of acutely disturbed child or adolescent awaiting psychiatric evaluation; section 14.3: Mood disorders; and section 14.5: Childhood psychosis.
- Beware of adverse interactions between illicit drugs and psychotropic medication i.e. drug levels of both illicit drugs and psychotropic medications are altered.

REFERRAL

» All for psychotherapeutic interventions or drug rehabilitation.
» Outpatient treatment: refer to SANCA (South African National Council on Alcoholism and Drug Dependence)

Tel: 011 8923829 or toll free: 0861472622.

» In-patient treatment: refer for in-patient drug rehabilitation.
» Patients with severe behavioural disturbance, persistent psychotic and manic symptoms to an in-patient child and adolescent psychiatric facility, for ongoing containment and management of psychiatric symptoms.

Note:

» Detoxification is rarely required in children. Mild withdrawals can be managed on an out-patient basis. Complicated withdrawals require admission.
» Alcohol withdrawal is very rare. If alcohol withdrawal is suspected, refer and discuss with an addiction specialist.
» DSM-IV-TR diagnostic criteria have been adopted as a guide for adolescent SUD diagnoses. However, the criteria were developed for adults, and have not been validated in adolescents.
» Standard protocols for the management of withdrawal states have been developed for adults. No standard paediatric guidelines exist. Management should therefore be on a 'case by case' basis and discussed with paediatric specialists.
» Substance induced psychotic or mood disorders should resolve within one month of cessation of drug of abuse. Consider major mental illnesses such as schizophrenia or bipolar disorder if symptoms persist beyond one month.

14.6.4 BEHAVIOURAL PROBLEMS ASSOCIATED WITH INTELLECTUAL DISABILITY

F81.9

DESCRIPTION

Children with intellectual disabilities present with higher rates of behavioural problems than the general population. Common presentations of adverse behaviours are:

» aggression,
» impulsivity,
» irritability,
» hyperactivity,
» short attention span,
» self-injurious behavior, and
» inappropriate sexual behaviour.

ADHD commonly co-occurs with intellectual disability and responds to treatment with stimulant medications.
Autism more commonly co-occurs with intellectual disability.
Depression occurs at a rate similar to the general population and 25% of children and adolescents suffer significant anxiety.
Increased rates of medical conditions such as seizures, genetic disorders and dysmorphic syndromes are associated with intellectual disability.
Intellectual disability associated with comorbid epilepsy is associated with increased rates of ADHD, behavioural dyscontrol and psychosis.

DIAGNOSTIC CRITERIA

Diagnostic criteria for psychiatric disorders in children with intellectual disability are the same as those for the general population.
However symptom expression may vary with developmental stage or level of intellectual functioning.

GENERAL AND SUPPORTIVE MEASURES

» Exclude medical conditions in children presenting with behavioural disturbances, particularly in children who are not able to communicate symptoms verbally (e.g. seizures, dental caries, covert infections, poisoning, foreign bodies, space occupying brain lesions and drug side effects).
» Exclude emotional, physical or sexual abuse in a child presenting with persistent adverse behaviour and emotional distress (especially in non-verbal children).
» Parental guidance is an important part of the management of children presenting with behavioural problems (psycho-education, behavior management).
» Behaviour modification principles form the basis of psychotherapeutic intervention.

MEDICINE TREATMENT

Psychotropic medication treatment should occur as part of a multidisciplinary diagnostic and therapeutic intervention.
Treat according to the primary psychiatric condition, as per treatment guidelines.
Do baseline blood tests and ECGs, particularly in children with underlying medical conditions.
Start with the lowest doses possible.
Increase dosages cautiously as children with intellectual disability may be more susceptible to adverse effects such as extrapyramidal side effects (EPSEs), neuroleptic malignant syndrome (NMS) or the disinhibiting effects of benzodiazepines.

REFERRAL

- Children presenting with severe aggression, inappropriate sexual behavior or self-injurious behavior for a diagnostic assessment, or admission to a tertiary level child psychiatry service, or a dedicated intellectual disability psychiatric service (if such a service exists in the region).
- Children presenting with psychosis or mania for an assessment or admission to a psychiatric unit.
- To a social worker or child protection services if abuse is suspected.

CHAPTER 15

RESPIRATORY SYSTEM

15.1 CHRONIC LUNG INFECTIONS

15.1.1 BRONCHIECTASIS

J47

DESCRIPTION

Irreversible dilatation of the subsegmental airways, inflammatory destruction of bronchial and peribronchial tissue, and accumulation of exudative material in dependent bronchi that occurs as a result of recurrent bacterial infections and aspiration pneumonia. There is bronchial luminal obstruction; ciliary dyskinesia; thick, tenacious secretions and lung tissue damage.

Complications include cor pulmonale and respiratory failure.

Predisposing conditions include HIV, TB, cystic fibrosis, primary ciliary dyskinesia primary immunodeficiency syndromes.

DIAGNOSTIC CRITERIA

» Chronic cough, usually with mucopurulent sputum and occasional haemoptysis.
» A bout of coughing on physical activity or change in posture, particularly while reclining.
» Cyanosis, fever, malaise, anorexia, poor weight gain, halitosis and clubbing.
» Recurrent and persistent lower respiratory tract infections.
» Chest X-ray showing cystic dilatation and tram tracking.
» If diagnosis is uncertain or where localised disease on chest X-ray is suspected, perform high resolution computed tomography. Features include cystic dilatation, signet ring sign, tram tracking.

GENERAL AND SUPPORTIVE MEASURES

» Identify and treat the underlying disorder or bacterial source.
» Clear secretions effectively with postural drainage and physiotherapy.
» Eliminate all foci of infection.
» Nutritional support.
» Nebulise with sodium chloride 0.9% or sodium chloride 3% (hypertonic saline) to aid sputum expectoration.
  Mix 3 mL of 5% sodium chloride with 2 mL water to make 3% solution.
  Method of sputum induction
  Precaution: If undertaking procedure in acutely sick child with respiratory compromise, be prepared to manage acute bronchospasm as this may be an associated adverse effect.

Nebulise with a bronchodilator:

- Salbutamol, solution, 0.15–0.3 mg/kg/dose in 2–4mL of sodium chloride 3% delivered at a flow of 5 L/min ute with oxygen for 20 minutes.

» Perform physiotherapy.
» Encourage patient to cough up sputum or if infant or small child obtain nasopharyngeal aspirate post physiotherapy.
» Send sample for culture and cytology as indicated.

SURGICAL TREATMENT

Consider in localised severe disease or progressive disease despite adequate medical treatment.

MEDICINE TREATMENT

Empiric antibiotic therapy for acute lung infections:

- Ampicillin, IV, 25 mg/kg/dose, 6 hourly.

PLUS

- Gentamicin, IV, 6 mg/kg once daily.

Change antibiotics according to culture and sensitivity results.

If poor response and no culture to guide antibiotic choice, consider infection due to *S. aureus*, TB or fungal infection.

If there is evidence of good clinical response, change to:

- Amoxicillin/clavulanic acid, oral, 30 mg/kg/dose of amoxicillin component 8 hourly.

Total antibiotic duration of 14 days.
Note:
These antibiotic regimens **do not** apply to children with cystic fibrosis.

In the acute phase if wheeze is present:

- Salbutamol solution, 5 mg/mL, nebulise 4 hourly.
    - o 5 mg salbutamol in 2–4 mL sodium chloride 0.9%.

In chronic phase
» Theophylline, modified release, oral, 6 mg/kg/dose 12 hourly.
  - o Titrate dose for optimal response.
  - o Maximum dose 10 mg/kg/dose.

- Annual influenza vaccination.
- Pneumococcal vaccine (conjugated), 2 additional doses 8 weeks apart.

REFERRAL

» Poor response to therapy.
» For confirmation of the diagnosis.
» For early surgical intervention of localised disease.

15.1.2 LUNG ABSCESS

J85

DESCRIPTION

A suppurative process that results from destruction of the pulmonary parenchyma and formation of a cavity. The cavity may be single, e.g. after aspiration or multiple, e.g. staphylococcal disease and cystic fibrosis.

Lung abscess may follow pneumonia caused by:

- » *S. aureus,*
- » anaerobic organisms,
- » *H. influenza,*
- » *K. pneumoniae,*
- » *S pneumoniae,*
- » *M. tuberculosis.*

Metastatic lung abscesses due to septicaemia or septic emboli may also occur.

Complications include:

- » bronchiectasis,
- » rupture into the bronchial tree or pleural cavity or vessels,
- » bronchopleural fistula.
- » empyema,
- » pulmonary osteo-arthropathy,
- » brain abscess, and

DIAGNOSTIC CRITERIA

- » Intermittent or recurrent fever, malaise, weight loss, anorexia and clubbing.
- » Productive purulent cough with halitosis and haemoptysis.
- » Amphoric breathing over the cavity may be present.
- » Chest X-ray will confirm cavity/cavities with or without an air-fluid level.

GENERAL AND SUPPORTIVE MEASURES

- » Identify underlying cause.
- » Physiotherapy and postural drainage.
- » Correct anaemia.
- » Nutritional support.

MEDICINE TREATMENT

Empiric antibiotic therapy for at least 14 days.

- Ampicillin, IV, 25 mg/kg/dose, 6 hourly.

PLUS

- Gentamicin, IV, 6 mg/kg/day as a single daily dose.

PLUS

- Metronidazole, IV, 7.5 mg/kg/dose, 8 hourly.

Change antibiotics according to culture and sensitivity results.

Poor response and no culture to guide antibiotic choice:

ADD

- Cloxacillin, IV, 50 mg/kg/dose, every 6 hours.

If there is evidence of good clinical response, change to only:

- Amoxicillin/clavulanic acid, oral, 30 mg/kg/dose of amoxicillin component 8 hourly.

SURGICAL TREATMENT

Consider surgical drainage of abscess and/or resection if medical treatment fails.

REFERRAL

» Complicated lung abscess not responding to therapy.
» Lung abscess where the underlying cause has not been established.

15.1.3 TUBERCULOSIS, PERINATAL

P37.0
*Notifiable condition

DESCRIPTION

Tuberculosis acquired in the first 3 months of life. Perinatal tuberculosis may be acquired in one of the following ways:

» transplacental transmission, usually extrapulmonary or disseminated TB,
» via the passage of swallowed maternal blood or amniotic fluid during delivery, usually extrapulmonary TB, or
» inhalation of the bacilli during the neonatal period – usually pulmonary TB.

DIAGNOSTIC CRITERIA

» Hepatosplenomegaly, a suggestive chest X-ray, TB exposure via a mother or close contact with another source case.
» Positive smear or culture on any suitable sample e.g. gastric aspirate in the neonate or tissue histology suggestive of TB.
» Endometrial swabs or sputum samples in the mother positive for *M. tuberculosis*. See section 15.1.4: Tuberculosis, pulmonary.

GENERAL AND SUPPORTIVE MEASURES

» Check drug sensitivity of source. If resistant, refer.
» Check HIV status of mother and, if positive, test baby with HIV PCR.
» Screen all household contacts for tuberculous infection or disease.
» Monitor the nutritional status of the neonate.
» Do not give BCG vaccine at birth.

MEDICINE TREATMENT

Treatment

Newborn infant of mother with tuberculosis with newborn having any signs suggestive of illness.

<u>Intensive phase</u>

- Rifampicin, oral, 10 mg/kg/dose once daily for 2 months.

PLUS

- Isoniazid, oral, 10 mg/kg/dose once daily for 2 months.

PLUS

- Pyrazinamide, oral, 35 mg/kg/dose once daily for 2 months.

<u>Continuation Phase</u>

- Isoniazid, oral, 10–15 mg/kg/dose once daily for 4 months.

PLUS

- Rifampicin, oral, 10–15 mg/kg/dose once daily for 4 months.

Prophylaxis

All asymptomatic neonates:

- Isoniazid, oral, 10 mg/kg/dose once daily for 6 months.

Weight band	Daily isoniazid (INH) 100 mg tablet
2–3.4 kg	¼ tablet
3.5–4.9 kg	½ tablet
5–7.4 kg	¾ tablet

During prophylaxis monitor the infant for active TB disease. After 6 months and asymptomatic for HIV:

- BCG vaccine.

In severely immunosuppressed patients the tuberculin reaction test can be negative in the presence of active tuberculosis.

REFERRAL

» Patients not responding to adequate therapy.
» Perinatal TB with a drug resistant source.

15.1.4 TUBERCULOSIS, PULMONARY

A16.9
*Notifiable condition

DESCRIPTION

A chronic, granulomatous disease of the lungs caused by *M. tuberculosis*.

Most children acquire tuberculosis from infected adults by inhalation.

Malnourished, immunosuppressed (HIV and AIDS) and children < 3 years of age with pulmonary tuberculosis (PTB) are always regarded as having a very serious disease.

Complications include:

- » enlarged hilar and mediastinal lymphadenopathy with obstruction, e.g. tracheal or bronchial airway compression or occlusion with secondary atelectasis or hyperinflation;
- » local spread of infection, e.g. TB bronchopneumonia, pleural effusion or cavitation;
- » disseminated disease, e.g. miliary TB, TB meningitis and metastatic extrapulmonary involvement.

DIAGNOSTIC CRITERIA

Any child presenting with symptoms and signs suggestive of pulmonary TB is regarded as a case of TB if there is:

- » a chest X-ray suggestive of TB,

and/or

- » history of exposure to an infectious TB case and/or positive Tuberculin Skin Test (TST) e.g. Mantoux.

The diagnosis is supported by a positive smear microscopy and/or positive *M. tuberculosis* PCR e.g. GeneXpert®. Culture, usually on gastric aspirates or induced sputum, is a confirmatory test.

- » Signs and symptoms include:
  - > unexplained weight loss or failure to thrive,
  - > unexplained fever for ≥ 2 weeks,
  - > chronic unremitting cough for > 14 days,
  - > lymphadenopathy (especially cervical, often matted),
  - > hepatosplenomegaly,
  - > consolidation and pleural effusion.
- » The following may be evident on chest X-ray:
  - > Direct or indirect evidence of hilar or mediastinal adenopathy with or without parenchymal opacification and/or bronchopneumonia,
  - > miliary changes,
  - > pleural effusions.

  Note:

  Miliary pattern on chest X-rays of HIV infected children may also be suggestive of a diagnosis of lymphoid interstitial pneumonitis (LIP). (The miliary pattern of TB extends into the periphery of the lungs whereas LIP usually does not)
- » Exposure to an adult with pulmonary tuberculosis.

- Tuberculin skin test (TST) e.g. Mantoux.
  - A positive TST has an induration of ≥ 10 mm.
  - A TST may be falsely negative in the presence of:
    - malnutrition,
    - immunodeficiency, e.g. HIV and AIDS,
    - immunosuppression, e.g. steroid therapy, cancer chemotherapy,
    - following overwhelming viral infection, e.g. measles or post vaccination.

  In these circumstances a TST induration of ≥ 5 mm may be regarded as positive. Frequently, the TST will be non-reactive in these cases and a decision not to start TB treatment should not be based on a negative TST test.
- *M. tuberculosis* is suggested by positive microscopy (acid fast bacilli on smear) and confirmed by culture on the following specimens but most children will not have microbiological confirmation of TB:
  - early morning gastric aspirate (empty stomach, no oral food intake for ≥ 4 hours),
  - sputum (older children),
  - induced sputum,
  - CSF,
  - pleural and ascitic fluids,
  - fine needle aspirate biopsies of lymph nodes,
  - ear swabs for culture in chronic otorrhoea.
- **PCR sputum positive on molecular testing**.
  - The *M. tuberculosis* PCR has an inferior yield to liquid culture and therefore should not replace culture. Identification of rifampicin resistance would imply multidrug resistance and allow modification of therapy accordingly.
  - Where available, the molecular *M. tuberculosis* PCR test should be performed on sputum and gastric aspirates in preference to fluorescent smear for acid fast bacilli as it increases the diagnostic yield and allows early identification of rifampicin resistance.
- Microscopy and culture in all cases.

GENERAL AND SUPPORTIVE MEASURES

- Identify and treat the source case.
- In case of known contact with adult MDR TB case, the child requires referral for appropriate MDR TB prophylaxis or treatment.
- Screen all contacts for TB infection.
- Screen all contacts for TB infection.
- Monitor the nutritional status of the child to assess response to treatment.
- Only symptomatic pleural effusions should be drained via pleural aspiration. (In such cases consider adjunctive steroid therapy)
- Ensure household infection control practices.

MEDICINE TREATMENT

Tuberculosis control programme drug regimens (2009)

Directly observed therapy (DOT), short-course, using fixed medicine combinations is recommended to avoid the development of antimicrobial resistance.

Give treatment daily in both the intensive (initial) and the continuation phase.

HIV infected children with tuberculosis should be treated according to the standard treatment protocol with clinical, radiologic and microbiologic follow-up to determine response to treatment.

	**Recommended dose ranges in mg/kg**	
	**Daily**	**Max daily**
Isoniazid (H)	10-15	300 mg
Rifampicin (R)	10-20	600 mg
Pyrazinamide (PZA/Z)	30-40	2 g
Ethambutol (EMB/E)	15-25	1200 mg

Uncomplicated with low bacillary load

Children up to 8 years:

	**Intensive phase 2 months**			**Continuation phase 4 months**
**Weight**	RH	PZA		RH
	60/60 mg	Give one of the following:		60/60 mg
		150 mg* **OR** 150 mg/3 mL	500 mg	
2–2.9 kg	½ tablet	1.5 mL	expert advice on dose	½ tablet
3–3.9 kg	¾ tablet	2.5 mL	¼ tablet	¾ tablet
4–5.9 kg	1 tablet	3 mL	¼ tablet	1 tablet
6–7.9 kg	1½ tablet		½ tablet	1½ tablets
8–11.9 kg	2 tablets		½ tablet	2 tablets
12–14.9 kg	3 tablets		1 tablet	3 tablets
15–19.9 kg	3½ tablets		1 tablet	3½ tablets
20–24.9 kg	4½ tablets		1½ tablet	4½ tablets
25–29.9 kg	5 tablets		2 tablets	5 tablets

* For each dose, dissolve 150 mg dispersible (1 tablet) in 3 mL of water to prepare a concentration of 50 mg/mL (150 mg/3 mL)

PLUS
If HIV infected or malnourished:
- Pyridoxine, oral, daily for 6 months:
  - o < 5 years of age: 12. 5 mg daily,
  - o > 5 years of age: 25 mg.

<u>Children > 8 years of age and adolescent:</u>

	**Two months intensive phase given daily**	**Four months continuation phase given daily**	
**Weight**	RHZE (150/75/400/275)	RH (150/75)	RH (300/150)
30–37 kg	2 tablets	2 tablets	
38–54 kg	3 tablets	3 tablets	
55–70 kg	4 tablets		2 tablets
≥71 kg	5 tablets		2 tablets

PLUS
If HIV infected or malnourished:
- Pyridoxine 12.5 mg daily for 6 months.

Complicated TB, high bacillary load
All other forms of severe TB. i.e. extensive pulmonary TB, spinal or, osteo-articular TB or abdominal TB.

<u>Children up to 8 years of age:</u>
Intensive phase:
Standard dose 4–drug therapy daily (RHZE) for 2 months.

Follow with:
Continuation phase:
Standard dose 2 drug therapy daily (isoniazid + rifampicin).

	**Intensive phase 2 months**				**Continuation phase at least 4 months (up to 7 months***)**
Weight	RH	PZA<br>Give one of the following:		EMB	RH
	60/60	150 mg*<br>**OR**<br>150 mg/3 mL	500 mg	400 mg tablet<br>**OR**<br>400 mg/8 mL**<br>solution	60/60
2–2.9 kg	½ tablet	1.5 mL	Expert advice on dose	1 mL	½ tablet
3–3.9 kg	¾ tablet	2.5 mL	¼ tablet	1.5 mL	¾ tablet
4–5.9 kg	1 tablet	3 mL	¼ tablet	2 mL	1 tablet
6–7.9 kg	1½ tablet		½ tablet	3 mL	1½ tablets
8–11.9 kg	2 tablets		½ tablet	½ tablet	2 tablets
12–14.9 kg	3 tablets		1 tablet	¾ tablet	3 tablets
15–19.9 kg	3½ tablets		1 tablet	1 tablet	3½ tablets
20–24.9 kg	4½ tablets		1½ tablet	1 tablet	4½ tablets
25–29.9 kg	5 tablets		2 tablets	1½ tablets	5 tablets

*For each dose, dissolve 150 mg dispersible (1 tablet) in 3 mL of water to prepare a concentration of 50 mg/mL (150 mg/3mL).
Note:
**For each dose, crush 400 mg (1 tablet) to a fine powder and dissolve in 8 mL of water to prepare a concentration of 400 mg/8 mL. Discard unused solution.
***Continuation phase may be prolonged to 7 months in slow responders and children with HIV.

PLUS

If HIV infected or malnourished:

- Pyridoxine 12.5 mg daily for 6 months.

Children > 8 years and adolescent:

	Two months intensive phase given daily	Four months continuation phase given daily	
**Weight**	RHZE (150/75/400/275)	RH (150/75)	RH (300/150)
30–37 kg	2 tablets	2 tablets	
38–54 kg	3 tablets	3 tablets	
55–70 kg	4 tablets		2 tablets
≥71 kg	5 tablets		2 tablets

PLUS

If HIV infected or malnourished:

- Pyridoxine 12.5 mg daily for 6 months.

Adjust treatment dosages to body weight.
If calculating dosages, rather give ½ tablet more than ½ tablet less.

Treatment of children who were previously successfully treated for TB (Retreatment)

A child, who was previously successfully treated for pulmonary TB, is at increased risk for re-infection with TB. It is imperative to exclude drug-resistant TB by carrying out sputum *M. tuberculosis* PCR plus culture with drug susceptibility testing (DST), and also determine DST of any known TB source case. If the above does not indicate resistant TB, treat as drug susceptible TB (high bacillary load) with close monitoring of response. Consider an extension of the duration of the continuation phase of therapy in these retreatment cases.

Drug resistant TB

Drug resistant TB single drug, multidrug (MDR), extensive drug resistant (XDR) and total drug-resistant (TDR) is as infectious as drug susceptible TB. Drug resistance can be primary or acquired.

MDR-TB disease indicates resistance to both rifampicin and isoniazid with/without resistance to any other antituberculosis medicine(s).
XDR-TB disease is defined as MDR-TB and in vitro resistance to any of the fluoroquinolones and any second-line injectable medicine.

Suspect DR-TB when any of the features listed below is present:

1. A known source case (or contact) with drug resistant TB or high-risk source case, e.g. on TB therapy who was recently released from prison.
2. A smear positive case after 2 months of TB treatment who failed (or deteriorated on) first-line antituberculosis treatment to which they were adherent (treatment failure or relapse within 6 months of treatment).

3. Any severely ill child with TB that failed or got worse on TB treatment.

4. Defaulted TB treatment (> 2 months).
5. Treatment interruptions (< 1 month) or who relapsed while on TB treatment or at the end of treatment.
6. With recurrent TB disease after completion of TB treatment (retreatment case).

When DR-TB is suspected, submit appropriate microbiological specimens for genotypic drug sensitivity test **and** culture for phenotypic drug susceptibility testing. *M. tuberculosis* PCR tests for rifampicin resistance only while the line probe assay (LPA) tests for isoniazid and rifampicin susceptibility. Second-line LPA tests for other antimicrobial resistance including quinolones. All samples that test positive on molecular PCR testing must have samples submitted for culture and drug susceptibility testing but therapy for MDR-TB must be instituted while awaiting results. False positive results with both the *M. tuberculosis* PCR. and line probe assay have been recorded. Clinical and radiological correlation with molecular results must always be considered and discuss discordant results with an expert.

Manage confirmed DR-TB in a dedic ated MDR-TB unit w ith appropriate infection control measures to prevent nosocomial transmission. Initiate treatment in consultation with a designated expert while awaiting referral to the designated MDR-TB centre. An uni nterrupted medicine supply, direct supervision with proper education and counselling is necessary.

The standardised empiric treatment protocol for MDR-TB for children is 5 drugs for 6 months or more for at least 6 days a week during the intensive phase and 4 drugs for at least 6 days a week for 18 months or less during the continuation phase. Exact duration of therapy for the intensive phase is 4 months after the first date of sampling of a negative culture result while the total duration of therapy should be 18 months after the first date of sampling of a negative culture result.

<u>Children < 8 years with MDR-TB</u>
Intensive phase:

- Levofloxacin, oral.
  - o 15–20 mg/kg/dose once daily.
  - o Maximum dose: 1 000 mg.
- Amikacin, IV, 15–22.5 mg/kg daily.
- Terizidone, oral, 15–20 mg/kg daily.
- Ethionamide, oral, 15–20 mg/kg daily.
- Pyrazinamide, oral, 30–40 mg/kg daily

Continuation phase:
Same as initial phase but stop amikacin.

Children > 8 years with MDR-TB
Intensive phase:

- Moxifloxacin, oral, daily.
    - < 25 kg: 200mg
    - > 25 kg: 400 mg
- Amikacin, IV, 15–22.5 mg/kg daily.
- Terizidone, oral, 15–20 mg/kg daily.
- Ethionamide, oral, 15–20 mg/kg daily.
- Pyrazinamide, oral, 30–40 mg/kg daily.

Continuation phase:
Same as intensive phase but stop amikacin.

Other agents may be substituted in special situations and in consultation with a designated expert. Cases of DR-TB must be monitored clinically, with radiology and microbiologically for response to therapy. TB culture conversion occurs when 2 consecutive TB culture results on sputum/gastric aspirates taken 30 days apart are negative and thereafter remains negative.

Disseminated (Miliary) TB
Children < 8 years
A 6-month regimen of all 4 the following medicines:

- Rifampicin, oral, 20 mg/kg as a single daily dose.
    - Maximum dose, oral, 600mg daily.

PLUS

- Isoniazid, oral, 20 mg/kg as a single daily dose.
    - Maximum dose, oral, 400mg daily.

PLUS

- Pyrazinamide, oral, 40 mg/kg as a single daily dose.
    - Maximum daily dose: 2 000 mg.

PLUS

- Ethionamide, oral, 20 mg/kg as a single daily dose.
    - Maximum daily dose: 1 000 mg.

- Pyridoxine 25 mg daily for 6 months.

Note:
All cases of miliary TB should have a lumbar puncture (LP) preformed. Any abnormal CSF results or where a LP is not performed, should be treated as a patient with TBM. See section 8.13: Meningitis, tuberculosis (TBM).

Preventive therapy for TB exposure/infection
Screen all children in close contact with an infectious pulmonary TB case for TB disease. Screening includes clinical history/examination and, if available, chest X-ray and tuberculin skin test (TST). Give antituberculosis treatment if the diagnosis of TB disease is confirmed or suspected.

Indications for Isoniazid Preventive Therapy (IPT):
» All asymptomatic children < 5 years of age, or HIV-infected irrespective of age, i.e. clinically normal, normal chest X-ray and TST positive or negative, in close contact with an infectious pulmonary TB case should receive isoniazid preventive therapy (IPT).
» Children < 5 years of age, or HIV-infected irrespective of age, who have had no previous TB treatment or preventive therapy, are asymptomatic without a history of close contact with an infectious pulmonary TB case but found to have a positive TST.
» Previous isoniazid preventive therapy or treatment does not protect the child against subsequent TB exposure/infection. If there is re-exposure to an infectious pulmonary TB case after completion of 6 months of chemotherapy, children (< 5 years or HIV-infected) should receive IPT after each episode of documented TB exposure for 6 months. In cases of re-exposure to infectious source cases while the child is on IPT, the duration of IPT should continue for as long as the source case remains infectious.

- Isoniazid, oral, 10 mg/kg daily for 6 months.

Preventive therapy in case of drug-resistant TB contact:
Isoniazid monoresistance:
- Rifampicin, oral, 15 mg/kg daily for 4 months.

Rifampicin monoresistance:
- Isoniazid, oral, 10 mg/kg daily for 6 months.

MDR or XDR-TB:
Close follow-up for two years.
Ensure household infection control practices are observed.
Refer all cases.

REFERRAL
» Poor response to standard TB treatment.
» Failure to exclude MDR-TB.
» Adverse drug reactions (ADR) requiring single drug combinations.
» MDR or MDR-TB contact.

15.2 CONDITIONS WITH PREDOMINANT WHEEZE

15.2.1 ASTHMA ATTACK, ACUTE

J46

DESCRIPTION

Acute exacerbation of w heezing that is unresponsive to bronchodilator therapy that i s usually effective in a chi ld who had been prev iously diagnosed with asthma.

DIAGNOSTIC CRITERIA

Clinical signs include:

- » intense wheezing,
- » hyperinflation,
- » tachypnoea,
- » hypoxaemia,
- » restlessness,
- » difficulty or inability to talk or feed,
- » decreased air entry,
- » dyspnoea,
- » tachycardia,
- » anxiety,
- » palpable pulsus paradoxus,
- » reduced peak flow rate.

The following are danger signs in acute, severe asthma and require referral:

- » restlessness,
- » disturbance in level of consciousness,
- » rising $PaCO_2$,
- » silent chest with auscultation,
- » PEFR < 60% of predicted value,
- » decreasing oxygen saturation < 85%,
- » palpable pulsus paradoxus,
- » chest pain (air leaks).

Classification of Severity of Acute Asthma Exacerbations

	Mild	Moderate	Severe
Oxygen saturation	> 95%	92–95%	< 92%
PEFR*	70–90%	50–70%	< 50%
Arterial $PaCO_2$	< 35 mmHg	< 40 mmHg	> 40 mmHg
Pulsus paradoxus	< 10 mmHg	10–20 mmHg may be palpable	20–40 mmHg palpable
Wheezing	expiratory	expiratory and inspiratory	breath sounds soft
Respiratory rate	< 40	> 40	> 40

* Peak expiratory flow rate – as percentage of predicted value.

	Mild	Moderate	Severe
Additional signs		» speaks normally<br>» difficulty with feeding<br>» chest indrawing	» unable to speak<br>» confusion<br>» cyanosis<br>» use of accessory muscles
management	• Short-acting ß2 agonist, e.g. salbutamol, inhalation<br>**PLUS**<br>• Prednisone, oral	• Oxygen,<br>• Short-acting ß2 agonist, e.g. salbutamol, inhalation<br>• ± ipratropium bromide inhalation<br>• Prednisone, oral	• Oxygen,<br>• Short-acting ß2 agonist, e.g. salbutamol inhalation stat<br>• Ipratropium bromide inhalation,<br>• Hydrocortisone, IV<br>If no response:<br>± $MgSO_4$, IV bolus stat<br>**OR**<br>± Salbutamol, IV bolus stat<br>**AND**<br>consider ICU care

GENERAL AND SUPPORTIVE MEASURES

» Admit child to a high care unit, if available.

» Monitor:
  > heart rate,
  > respiratory rate,
  > PEFR,
  > pulse oximetry.
  > blood pressure,
  > acid-base status,
  > blood gases,

» Ensure adequate hydration:
  > Encourage intake of normal maintenance volume of oral fluids, avoid overhydration.

» If unable to drink, give 0.45% sodium chloride/5% dextrose. Patients with prolonged severe asthma may become dehydrated as a result of poor intake or vomiting. It is however inadvisable to overhydrate patients with acute asthma: do not exceed the recommended IV fluid volume in children, i.e. 50 mL/kg/24 hours.

Note:
Physiotherapy, antihistamines, antibiotics and sedation are not beneficial in the acute setting.
Agitation and restlessness are signs of severe hypoxia.

MEDICINE TREATMENT
Mild and moderate asthma
Bronchodilator, i.e. short-acting $ß_2$ agonist
- Salbutamol, inhalation, using a metered-dose inhaler with a spacer device.
  - 200–400 mcg (2–4 puffs) repeated every 20–30 minutes depending on clinical response.

OR
- Salbutamol, solution, 0.15–0.3 mg/kg/dose nebulise at 20 minute intervals for 3 doses.
  - Maximum dose: 5 mg/dose.
  - 5 mg salbutamol in 4 mL sodium chloride 0.9% delivered at a flow of 5 L/minute with oxygen.

PLUS
- Prednisone, oral, 1–2 mg /kg, daily immediately up to a maximum of:
  - 20 mg: children < 2 years for 5 days.
  - 30 mg: children 2–5 years for 5 days.
  - 40 mg: children 6–12 years for 5 days.

Moderate or severe asthma
Step 1:
To maintain arterial oxygen saturation ≥ 95%:
- Oxygen, 100%, at least 4–6 L/minute by facemask **or** 1–2 L/minute by nasal cannula.

PLUS
- Short-acting $ß_2$ agonist:
- Salbutamol, inhalation, using a metered-dose inhaler with a spacer device. Up to 10 puffs (1mg) per administration for severe asthma.
  - 400–600 mcg (4–6 puffs) up to 10 puffs repeated every 20–30 minutes depending on clinical response.

OR
- Salbutamol, solution, 0.15–0.3 mg/kg/dose nebulise at 20 minute intervals for 3 doses.
  - Maximum dose: 5 mg/dose.
  - 5 mg salbutamol in 4 mL sodium chloride 0.9% delivered at a flow of 5 L/minute with oxygen.

PLUS (if severe)

- Ipratropium bromide, solution, 0.25 mg, nebulise immediately.
  - If severe, follow with 0.25 mg every 20–30 minutes for 4 doses over 2 hours.
  - Maintenance dose: 0.25 mg 6 hourly.
  - 0.25mg (2 mL) ipratropium bromide in 2 mL sodium chloride 0.9%.
  - Ipratropium bromide may be mixed with a $ß_2$ agonist.

PLUS

- Prednisone, oral, 1–2 mg /kg, immediately up to a maximum of:
  - 20 mg: children < 2 years for 5 days.
  - 30 mg: children 2–5 years for 5 days.
  - 40 mg: children 6–12 years for 5 days.

Step 2:

Assess response to treatment in step 1 by using the following table:

	**Responder**	**Non-responder**
PEFR	improvement >20% OR > 80% (best/predicted)	improvement < 20% OR < 80% (best/predicted)
Respiratory rate	< 40/minute	> 40/minute
Retraction	absent	present
Speech	normal	impaired
Feeding	normal	impaired

Responder: patient who maintains an adequate response for at least 1 hour.
Non-responder: patient who fails to respond adequately to treatment in step 1.

Proceed to step 3.

Step 3:

<u>Responder:</u>

Review current treatment, possible precipitating or aggravating factors and commence:

- Prednisone, oral, 2 mg/kg as a single daily dose for 7 days.

If oral corticosteroids are not available:

- Hydrocortisone, IV, 2 mg/kg/dose, 6 hourly.

PLUS

- Short-acting $ß_2$ agonist:
- Salbutamol, inhalation, 200 mcg (2 puffs) as required using a metered-dose inhaler with a spacer device.

Review maintenance asthma therapy at follow up.

<u>Non-responder:</u>
Intensify treatment as follows:

- Short-acting $ß_2$ agonist:
- Salbutamol, solution, 0.15–0.3 mg/kg/dose nebulise at 20 minute intervals for 3 doses.
    - Maximum dose: 5 mg/dose.
    - 5 mg salbutamol in 4 mL sodium chloride 0.9% delivered at a flow of 5 L/minute with oxygen.

AND

- Ipratropium bromide, solution, 0.25 mg, nebulise immediately.
    - If severe, follow with 0.25 mg every 20–30 minutes for 4 doses over 2 hours.
    - Maintenance dose: 0.25 mg 6 hourly.
    - 0.25mg (2 mL) ipratropium bromide in 2 mL sodium chloride 0.9%.
    - Ipratropium bromide may be mixed with a ß2 agonist.

PLUS

Continue corticosteroid:

- Prednisone, oral, 2 mg/kg as a single daily dose.

OR

- Hydrocortisone, IV, 2 mg/kg/dose 6 hourly.

<u>Failure to respond - consult paediatrician</u>

- Magnesium sulphate, IV bolus, 25–75 mcg/kg administered over 20 minutes.

OR

- Salbutamol, IV, 15 mcg/kg as a single dose administered over 10 minutes.

Consider need for intensive care.

Step 4:

Assess response to treatment in Step 3.
If non-responsive, admit to intensive care unit for consideration of:

- Magnesium sulphate, IV bolus, 25–75 mcg/kg administered over 20 minutes (if not already given).

AND

- Salbutamol, IV.
    - Loading dose: 0.5 mcg/kg (do not give if stat dose already given).
    - Follow with: 0.2 mcg/kg/minute.
    - If necessary, increase dose by 0.1 mcg/kg every 15 minutes.
    - Maximum dose: 5 mcg/kg/minute.
    - Monitor electrolytes.

No further response

In cases of life threatening asthma in the intensive care unit:

- Aminophyline, IV, 5 mg/kg, loading dose administered over 20–30 minutes. Omit loading dose in children receiving maintenance oral theophylline.
  - o Follow with: 1 mg/kg/hour continuous infusion.
  - o ECG monitoring.

REFERRAL

» Acute exacerbation not responding to treatment.

15.2.2 ASTHMA, CHRONIC

J 45

DESCRIPTION

Asthma is a chronic inflammatory airways disease in which many cells and cellular elements play a role. Susceptible individuals present with recurrent episodes of wheezing, breathlessness, chest tightness and cough particularly in the early morning. There is widespread variable airflow obstruction that is reversible either spontaneously or with treatment. A variety of stimuli, e.g. allergens, viral infections, weather changes, emotional upsets or other irritants precipitate inflammation that is associated with increased bronchial hyper-responsiveness.

DIAGNOSTIC CRITERIA

» Chronic, persistent/recurrent cough and/or wheezing that responds to a bronchodilator.

» Objective evidence of reversible airway obstruction, as measured by > 15% improvement of the peak flow or > 12% improvement in the FEV_1 20 minutes after administration of an inhaled bronchodilator confirms the diagnosis. (FEV_1 = forced expiratory volume in 1 second)

» A family history of atopy, night or exercise-induced coughing and/or wheezing.

Control of asthma

The severity of asthma can vary with time and regular reassessments (at least every 3 months) are necessary.

On treatment chronic asthma is classified as:

» controlled,

» partially controlled, or

» uncontrolled.

The following criteria are used to classify control:

	Controlled	Partially controlled (Any present in any week)	Uncontrolled
Daytime symptoms	None (2 or less/week)	More than twice/week	3 or more features of partly controlled asthma present in any week
Limitations of activities	None	Any	
Nocturnal symptoms/ awakening	None	Any	
Need for rescue/ "reliever" treatment	None (2 or less/week)	More than twice/week	
Lung function (PEF or FEV_1)	Normal	< 80% predicted or personal best (if known) on any day	
Exacerbation	None	One or more/year.	

Partially controlled or uncontrolled cases requires escalation in therapy while cases controlled for > 4 months requires gradual reduction in therapy.

Assessment of severity and classification of chronic asthma

Before initiating treatment, classify the grade of severity of patient illness according to the presence of the most severe feature. This assists in choosing the most appropriate initial maintenance therapy.

Infrequent asthma: less than one acute exacerbation in 4–6 months.
Persistent asthma: mild, moderate or severe.

Criteria	Mild	Moderate	Severe
day time symptoms	2–4/week	> 4/week	continuous
night time symptoms	2–4/month	> 4/month	frequent
prior admission to hospital for asthma	None	one previous admission	> one previous admission or admission to ICU
PEFR*	> 80	60–80	< 60

* Peak expiratory flow rate – patient's best as percentage of predicted value.

GENERAL AND SUPPORTIVE MEASURES

» Environmental control, avoid triggers, e.g.:
  - > exposure to cigarette smoke,
  - > preservatives such as sulphites and benzoates,
  - > house pets such as cats and dogs,
  - > house dust mites sensitisation: use plastic mattress covers, and remove bedroom carpets.

» Wash bedding covers in hot water (> 70 °C).

» Educate children, parents, caregivers and teachers.

MEDICINE TREATMENT

Medicine delivery systems

Use spacer devices with a metered dose inhaler. Prime all spacers with 2 doses of inhaled medication prior to first use. The size of the spacer is dependent on tidal volume of the child:

	Spacer volume	Face mask/ mouthpiece	Valve
Infants	150–250 mL	facemask	mandatory
Children < 5 years<br>Children > 5 years	500 mL	facemask<br>mouthpiece	recommended
Adolescents	750 mL	mouthpiece	not necessary

The technique of using the spacer varies with age:

Infants and young children: use tidal breathing of 10 long, deep, slow breaths.

Older children and adolescents: breathe out fully, actuate the inhaler, then inhale the entire contents in one long slow breath. Hold breath for 10 seconds.

Inhaled corticosteroid use

Inhaled corticosteroids are indicated for all cases of persistent asthma. Spacer devices increase the efficacy of inhaled corticosteroids.
Rinse the mouth after inhalation of inhaled corticosteroids to reduce systemic absorption and adverse effects.
Wash face if a face mask is used.
Use the lowest possible effective dose of steroids.

15.2.2.1 INFREQUENT ASTHMA

To relieve symptoms:

▪ $ß_2$ agonist (short-acting), e.g.:

- Salbutamol, inhalation, 100–200 mcg, as required 3–4 times daily using a metered-dose inhaler with a spacer device until symptoms are relieved.

Note:
Failure to respond to 2 doses of an inhaled bronchodilator given 20 minutes apart is an indication of an **acute exacerbation** of asthma. See section 15.2.1: Asthma, acute attack.

15.2.2.2 PERSISTENT ASTHMA

Mild persistent asthma
When needed for acute exacerbations:
- $ß_2$ agonist (short-acting), e.g.:
- Salbutamol, inhalation, 100–200 mcg, as required 3–4 times daily using a metered-dose inhaler with a spacer device until symptoms are relieved.

PLUS
Low dose inhaled corticosteroids, e.g.:
- Beclomethasone **or** budesonide, inhalation, 50–100 mcg, 12 hourly using a metered-dose inhaler with a spacer device.

Moderate persistent asthma
To relieve symptoms:
- $ß_2$ agonist (short-acting), e.g.:
- Salbutamol, inhalation, 100–200 mcg, as required 3–4 times daily using a metered-dose inhaler with a spacer device until symptoms are relieved.

PLUS
Regular anti-inflammatory treatment with medium-dose inhaled corticosteroids:
- Budesonide, inhalation, 100–200 mcg, 12 hourly using a metered-dose inhaler with a spacer device.

OR
In children > 6 years with multiple allergies on other steroid formulations, low-dose inhaled corticosteroids plus long-acting beta agonist (LABA) e.g.:
- Fluticasone plus salmeterol by inhalation, 12 hourly. Specialist initiated.

Severe persistent asthma
To relieve symptoms:
- $ß_2$ agonist (short-acting), e.g.:
- Salbutamol, inhalation, 100–200 mcg, as required 3–4 times daily using a metered-dose inhaler with a spacer device until symptoms are relieved.

PLUS
Low-dose inhaled corticosteroids plus LABA, e.g.:
- Fluticasone plus salmeterol, inhaled, 12 hourly. Specialist Initiated.

REFERRAL

- After a life-threatening episode.
- Unstable or difficult to control asthma.
- Asthma interfering with normal life, despite treatment.
- Severe persistent asthma not responding to therapy.

Suggested reference peak expiratory flow (PEF) values for children:

Height (cm)	PEF		PEF	
	Caucasian		African	
	Male	Female	Male	Female
100	127	142	120	126
101	131	145	124	130
102	135	149	128	133
103	138	152	131	137
104	142	156	135	140
105	146	159	139	144
106	150	163	143	148
107	154	166	147	151
108	158	170	151	155
109	162	174	155	159
110	166	178	159	163
111	170	182	163	167
112	175	185	168	171
113	179	189	172	175
114	184	193	176	179
115	188	197	181	184
116	193	202	186	188
117	197	206	190	192
118	202	210	195	197
119	207	214	200	201
120	212	218	205	206
121	217	223	210	210
122	222	227	215	215
123	227	232	220	220
124	232	236	226	225
125	237	241	231	230
126	243	245	236	235
127	248	250	242	240
128	254	255	248	245
129	259	259	253	250
130	265	264	259	255
131	271	269	265	260
132	276	274	271	266
133	282	279	277	271
134	288	284	283	277
135	294	289	289	282
136	300	294	295	288
137	307	299	302	293

Height (cm)	PEF		PEF	
	Caucasian		African	
	Male	Female	Male	Female
138	313	304	308	299
139	319	309	315	305
140	326	315	322	311
141	332	320	328	317
142	339	325	335	323
143	345	331	342	329
144	352	336	349	335
145	359	342	356	342
146	366	348	363	348
147	373	353	371	354
148	380	354	378	361
149	387	365	386	368
150	395	371	392	374
151	402	377	401	381
152	410	382	409	388
153	417	388	417	395
154	425	394	425	402
155	433	401	433	409
156	440	409	441	416
157	448	413	442	423
158	456	419	458	430
159	464	426	466	437
160	473	432	475	445
161	481	438	484	452
162	489	445	492	460
163	498	451	501	468
164	506	458	510	475
165	515	465	520	483
166	524	471	529	491
167	533	478	538	499
168	542	485	548	507
169	551	492	557	515
170	560	499	567	523
171	569	506	577	532
172	578	513	587	540
173	588	520	597	548
174	597	527	607	557
175	607	534	617	566
176	617	541	627	574
177	626	549	638	583
178	636	556	648	592
179	646	563	659	601
180	657	571	670	610

For optimal control 80% of the predicted peak flow is required.

15.2.3 BRONCHIOLITIS

J21.9

DESCRIPTION

Bronchiolitis is an a cute viral infection of the small airways of low er respiratory tract affecting children between 4 months to 2 years of age.
The most common pathogen is the respiratory syncytial virus.
Recurrent episodes of wheeze associated with bronchiolitis may occur, and some of these children may develop asthma.

Assessment of severity

Signs of severe disease include:

- » infants under 3 months of age, especially premature babies;
- » respiratory failure;
- » nasal flaring;
- » distress when speaking or crying;
- » apnoea;
- » pneumothorax;
- » central cyanosis;
- » inability to feed;
- » lower chest wall indrawing;
- » grunting;
- » discomfort in breathing;
- » hypoxia;
- » convulsions and decreased level of consciousness.

Mild cases are managed as outpatients.

DIAGNOSTIC CRITERIA

- » Prodrome of viral infection: irritability and feeding difficulties.
- » A wheeze that is slowly responsive or non-responsive to bronchodilators.
- » Crepitations and signs of hyperinflation of the chest.
- » A chest X -ray is useful in confirming hyperinflation and associated segmental atelectasis.

GENERAL AND SUPPORTIVE MEASURES

- » Isolate from other infants, if possible.
- » Patients with signs of severe disease or associated complications or underlying cardiorespiratory disorders should be hospitalised for monitoring of:
  - > breathing pattern (apnoea monitoring if < 3 months of age),
  - > signs of respiratory failure,
  - > heart rate and respiratory rate,
  - > temperature,
  - > SaO_2,
  - > hydration and nutrition. IV maintenance fluid according to age, if oral/nasogastric feeds/fluids are not tolerated. Avoid overhydration.

MEDICINE TREATMENT

For all hospitalised patients

Only if saturation < 92%:

- Oxygen, humidified, 1–2 L/min via nasal prongs or nasal cannula.
  - o Ensure clear nasal passages and correctly position the nasal prongs.

For outpatient-based therapy nebulise with:

- Epinephrine (adrenaline) 1: 1 000, 1 mL diluted in 2–4 mL sodium chloride 3% immediately and every 2–4 hours.

For inpatient therapy nebulise with:

- Sodium chloride 3%, solution, 2–4 mL, 4–6 hourly.
  Mix 3 mL of 5% sodium chloride with 2 mL water to make 3% solution.

If there is poor response or deterioration, change to:

- Epinephrine (adrenaline) 1: 1 000, 1 mL diluted in 2–4 mL sodium chloride 3% immediately and every 2–4 hours.

PLUS

- Sodium chloride 3%, solution, 2–4 mL, 4–6 hourly.

Antibiotic therapy

Routine antibiotic therapy is not indicated. Only use antibiotics if there is:

» raised leukocyte count,
» persistent fever of ≥ 38.5°C, and/or
» a chest X-ray showing opacification suggestive of pneumonia.

<u>For secondary bacterial infection:</u>

- Amoxicillin, oral, 30 mg/kg/dose, 8 hourly for 5 days.

Note:

There is no evidence that β_2-agonists, anticholinergics, inhaled corticosteroids or leukotriene receptor antagonists have any benefit in these children. In severe disease requiring intensive care, oral corticosteroids may be considered after specialist consultation.

REFERRAL

» Bronchiolitis with signs of respiratory failure.

15.3 COUGH WITH PREDOMINANT FEVER AND TACHYPNOEA

15.3.1 PNEUMONIA

J18.9

DESCRIPTION

Infection of the lung parenchyma that is classified as non-severe, severe or very severe based on clinical features.

Empiric antibiotics are indicated in all cases of pneumonia, as delay in treatment is associated with poor outcome. Antibiotic choice is based on an assessment of severity and likely aetiology.

Common bacterial causes of pneumonia include:

Neonates:

- » Group B ß-haemolytic Streptococci.
- » *Klebsiella spp.*
- » *E. coli.*
- » Chlamydia.
- » *S. aureus.*

Children:

- » *S. pneumoniae.*
- » *H. influenzae.*
- » *S. aureus.*
- » *M. catarrhalis.*
- » *M. pneumonia.*

Common viral causes in infancy and early childhood include:

- » influenza virus,
- » measles virus,
- » respiratory syncytial virus,
- » para-influenza virus,
- » cytomegalovirus,
- » adenovirus.

Predisposing factors for pneumonia include:

- » aspiration,
- » septicaemia,
- » measles,
- » immunosuppression,
- » malnutrition,
- » whooping cough,
- » abnormalities in clearance of mucus/secretions (e.g. cystic fibrosis, foreign body and ciliary dysfunction).

Complications of pneumonia include:

- » respiratory failure,
- » empyema,
- » pleuritis,
- » pleural effusion,
- » pneumothorax,
- » bronchiectasis.

DIAGNOSTIC CRITERIA

» Tachypnoea: age dependent:

Age	Respiratory rate
< 60 days	> 60/minute
2–12 months	> 50/minute
1–5 years	> 40/minute

Non-severe pneumonia

» Cough and fast breathing (tachypnoea).

Severe pneumonia

Above plus one of the following:

» lower chest wall in-drawing;
» auscultatory signs i.e. decreased breath sounds, bronchial breathing, crackles, increased vocal resonance or pleural rub;
» dullness to percussion.

Very severe pneumonia

Above plus at least one of the following:

» central cyanosis,
» inability to feed,
» convulsions, lethargy or decreased level of consciousness,
» grunting,
» nasal flaring,
» < 60 days old.

Note:

All infants aged up to 60 days with pneumonia must be considered as having very severe disease.

Investigations

» Perform a chest X-ray when there is failure to respond to therapy, in cases with severe and very severe disease where complications or the diagnosis of TB is suspected. Perform a lateral and postero-anterior view. A chest X-ray is not essential in cases with non-severe pneumonia.
» TST.
» If facilities are available, blood, induced sputum, nasopharyngeal aspirates (viruses and PCP) and gastric aspirate (TB) should be sent for microscopy and culture, preferably before initiating antibiotics.

GENERAL AND SUPPORTIVE MEASURES

» Bed rest.
» Clear nasal and oral passages of thick secretions.
» Nurse neonates in a neutral thermal environment.

» Monitor:
  - > respiratory rate,
  - > heart rate,
  - > SaO_2,
  - > temperature,
  - > hydration,
  - > blood pressure,
  - > blood gases,
  - > acid-base status,
  - > hypercapnia and/or hypoxia are indications for ventilatory support.

» Maintain nutrition: Continue breast and oral feeds.
  - > Consider small frequent feeds by oro/nasogastric tube or IV fluids if respiratory rate > 60/minutes or enteral feeds are not tolerated.

SURGICAL TREATMENT

» To relieve a tension pneumothorax, do needle aspiration followed by chest tube placement.

» For symptomatic pleural effusion, do needle aspiration; if empyema, insert large bore chest tube drainage.

» Small or asymptomatic pneumothoraces in infants and children (excluding neonates) usually do not require treatment other than close observation, but identify and treat the underlying cause for the pneumothorax.

MEDICINE TREATMENT

- Oxygen, humidified, by nasal prongs is preferred.
  - o Continue oxygen until respiratory rate is < 60/minute.

To relieve discomfort:

- Paracetamol, oral/NGT, 15 mg/kg, 6 hourly as required.

If significant degree of wheezing is present:

- Salbutamol, inhalation, 100–200 mcg, as required using a metered-dose inhaler with a spacer device until symptoms are relieved.

Empirical antibiotic therapy

Choice of antibiotic depends on the severity of the condition and predisposing factors.
Reconsider choice of antibiotic when the results of cultures become available or the child does not improve.

<u>Non severe pneumonia</u>

- Amoxicillin, oral, 30 mg/kg/dose, 8 hourly for at least 3 days.

<u>Severe pneumonia:</u>

If hospitalisation is not required:

- Amoxicillin, oral, 30 mg/kg/dose, 8 hourly for 5 days.

OR
If hospitalisation is required:
- Ampicillin, IV, 25 mg/kg/dose 6 hourly.

If clinical condition allows use or change to:
- Amoxicillin, oral, 30 mg/kg/dose, 8 hourly to complete 5 days of therapy.

<u>Very severe pneumonia</u>
Assume child is HIV infected until proven otherwise.
See section 9.1: Human Immunodeficiency Virus Infections.

<u>Birth to 59 days</u>
- Ampicillin, IV, 25 mg/kg/dose 6 hourly.

PLUS
- Gentamicin, IV, 6 mg/kg as a single daily dose for 5–10 days.

Observe the response closely and reconsider choice of antibiotics.

<u>In children 60 days and older</u>
- Ceftriaxone, IV, 50 mg/kg/dose 24 hourly.

Switch to oral as soon as there is a response:
- Amoxicillin/clavulanate, oral, 30 mg/kg/dose of amoxicillin component 8 hourly.

15.3.2 PREDISPOSING CONDITIONS AND MODIFICATION OF ANTIMICROBIAL THERAPY

15.3.2.1 PNEUMONIA, VIRAL INFECTION
J12.9

DESCRIPTION
The commonest cause of pneumonia in ch ildren is viral infection. Respiratory syncyctial virus, adenovirus, cytomegalovirus, influenza, parainfluenza, adenovirus, herpes, human metapneumovirus and m easles are the common viruses responsible for infections of the respiratory tract, Children present with fever, cough, rhinorrhea and chest in-drawing. Scattered fine crackles may also occur.

DIAGNOSTIC CRITERIA
» Chest X-ray: usually patchy interstitial changes.
» Nasopharyngeal aspirate for respiratory viruses screen via immunofluorescence.

GENERAL AND SUPPORTIVE MEASURES

» Maintain nutrition.
» Maintain hydration.

MEDICINE TREATMENT

Only if saturation < 92%:

- Oxygen, humidified, 1–2 L/min via nasal prongs or nasal cannula.

If temperature is > 38.5°C:

- Paracetamol, oral, 15 mg/kg/dose 6 hourly.

There is no ro le for ro utine antiviral therapy. Refer children with viral pneumonia in respiratory failure for support with mechanical ventilation.

Monitor for secondary bacterial infection.

REFERRAL

» Deterioration of condition.

15.3.2.2 FUNGAL INFECTION

J17.2*/B49

DESCRIPTION

May occur in immunosuppressed patients and present with deep draining sinuses or associated fungal lesions in the larynx, trachea or mouth.

GENERAL AND SUPPORTIVE MEASURES

» Perform cultures.
» Screening blood test is β-glucans.

MEDICINE TREATMENT

- Amphotericin B, IV, 0.6–1.0 mg/kg as a single daily dose infused over 4 hours for at least 14 days.

Prehydration before administering amphotericin to prevent renal impairment:

- Sodium chloride 0.9%, IV, 20 mL/kg **plus** potassium chloride, 20 mmol/L infused over 2–4 hours.

OR

- Fluconazole, IV/oral, 10 mg/kg as a single daily dose for at least 14 days.

<u>Neonates < 2 weeks:</u>

- Amphotericin B, IV, 0.7–1.0 mg/kg every 72 hours infused over 4 hours for at least 14 days.

Prehydrate before administering amphotericin to prevent renal impairment:

- Sodium chloride 0.9%, IV, 20 mL/kg **plus** potassium chloride, 20 mmol/L infused over 2–4 hours.

OR

- Fluconazole, IV/oral, 10 mg/kg every 72 hours for at least 14 days.

<u>Neonates 2–4 weeks</u>

- Amphotericin B, IV, 0.7–1.0 mg/kg every 48 hours infused over 4 hours for at least 14 days.

Prehydration before administering amphotericin to prevent renal impairment:

- Sodium chloride 0.9%, IV, 20 mL/kg **plus** potassium chloride, 20 mmol/L infused over 2–4 hours

OR

- Fluconazole, IV/oral, 10 mg/kg every 48 hours for at least 14 days.

Note:
If screening test or culture-negative, discontinue antifungal treatment.
If culture positive, consider sensitivity results and adjust accordingly.

15.3.2.3 PNEUMONIA DUE TO ANAEROBIC INFECTION

DESCRIPTION

Often seen in comatose patients with aspiration syndromes.

MEDICINE TREATMENT

- Metronidazole, oral, 7.5 mg/kg/dose, 8 hourly for 5 days.

PLUS

- Ampicillin, IV, 25 mg/kg/dose, 6 hourly for 5 days.

PLUS

- Gentamicin, IV, 6 mg/kg as a single daily dose for 5 days.

15.3.2.4 PNEUMONIA, ATYPICAL DUE TO MYCOPLASMA OR CHLAMYDIAL INFECTION

J15.7/J16.0

DESCRIPTION

This condition is often seen in children of school going age.

Cases present with fever, arthralgia, headache, cough and crepitations.

DIAGNOSTIC CRITERIA
Clinical

» In the neonatal period, chlamydial pneumonia presents with a staccato cough and sticky eyes.

Investigations

» Chest X-ray show interstitial infiltrates, lobar consolidation and hilar adenopathy.

MEDICINE TREATMENT

Mild disease

- ▪ Macrolide, e.g.:
- • Clarithromycin, oral, 7.5 mg/kg/dose, 12 hourly for 7 days.

For severe atypical pneumonia

- ▪ Macrolide, e.g.:
- • Clarithromycin, IV, 7.5 mg/kg/dose, 12 hourly for 7 days.

15.3.2.5 PNEUMONIA, STAPHYLOCOCCAL

J15.2

DESCRIPTION

Child presents with a toxic illness, tachypnoea, chest wall in-drawing and cough.

DIAGNOSTIC CRITERIA
Investigations

» Chest X-ray shows a destructive pneumonia with pneumatocoele, pleural effusion or empyema.
» Markedly elevated white blood cells.

MEDICINE TREATMENT

- • Cloxacillin, IV, 50 mg/kg/dose every 6 hours.

If there is evidence of good clinical response, change to:

- • Flucloxacillin, oral, 12.5–25 mg/kg/dose 6 hourly for at least 21 days.

<u>MRSA pneumonia:</u>

- • Vancomycin, IV, 15 mg/kg/dose 6 hourly infused over 1 hour.

15.3.2.6 PNEUMONIA IN HIV EXPOSED OR INFECTED CHILDREN

DESCRIPTION

In additional to common bacterial, fungal and viral pathogens causing pneumonia, opportunistic micro-organisms in a 'polymicrobial mix' are common in these children. Many of these children may fail to respond to the standard antibiotic treatment for pneumonia. Micro-organisms commonly involved are:

- » *P. jiroveci* (PCP),
- » Mycobacteria, e.g. *M. tuberculosis*
- » *S. aureus,*
- » *S. pneumonia,*
- » cytomegalovirus,
- » Non-typhoidal Salmonella,
- » *Klebsiella pneumonia,*
- » Candida.

S. pneumonia, S. aureus and gram negative bacteria e.g. *Klebsiella pneumoniae* and Non-typhoid Salmonella cause a significant proportion of HIV-related pneumonia in early childhood.

P. jiroveci (PCP)

- » PCP is a common fungal infection of the lung in infants from 2–6 months.
- » Presents as an acute onset of respiratory distress with minimal/absent chest signs in a child who is HIV exposed.
- » Hypoxaemia and cyanosis are common features in severe disease.
- » Chest X-ray shows a range of abnormalities including bilateral perihilar interstitial changes.

Perinatal acquired cytomegalovirus associated pneumonia in HIV infected infants

- » Presents as an interstitial pneumonitis with acute hypoxic respiratory failure.
- » It may present as a multisystem sepsis-like syndrome, with hepatitis, neutropenia, pneumonitis, colitis and thrombocytopaenia.
- » Often occurs in children who are severely immunosuppressed (CDC Immune category 3) and carries a significant mortality.
- » Transmission occurs by blood transfusion, body fluids and via breast milk (reduced by freezing, pasteurisation, hand washing). Even though there is a high CMV IgG in breast milk, there is low rate of CMV transmission and therefore continue breast feeding.
- » CMV co-infection occurs commonly as polymicrobial infection with PCP and bacteria.

Tuberculosis in HIV infected children

- » Occurs in children at all ages.
- » The diagnosis is difficult to confirm.
- » A Mantoux test of $\geq$ 5 mm induration is indicative of tuberculosis infection.

<u>HIV positive children with chronic lung disease</u>

- Often presents with lymphoid interstitial pneumonitis and bronchiectasis.
- Secondary infection with bacteria similar to those seen in acute pneumonia are commonly isolated from these children.

DIAGNOSTIC CRITERIA

Investigations

- Chest X-ray.
- Screen for HIV infection:
  - In children < 18 months utilising HIV DNA PCR and quantitative HIV viral load.
  - In children > 18 months HIV ELISA (screening and confirmatory).
- Investigate for PCP:
  - Immunofluorescence and silver methenamine staining on induced sputum sample.
- Screen immediately for CMV using CMV viral load, where available.
  - A viral load of > 100 000 copies/mL suggests CMV disease: treat.
  - A viral load below 10 000 copies/mL is regarded as CMV infection: no therapy recommended.
  - CMV viral loads between 10 000 copies/mL and 100 000 copies/mL: treat if patient is clinical symptomatic.
  - Tissue biopsy: intranuclear inclusion bodies, typical 'owl eye' cells.

    Note:

    A positive urine culture for CMV or a positive serology does not confirm a diagnosis of CMV disease. Patient would not qualify for treatment solely on those results.
- Tuberculosis:
  - TST, MCS on induced sputum, *M. tuberculosis* PCR test on sputum.

GENERAL AND SUPPORTIVE MEASURES

- Avoid exposure to infectious agents.
- Adequate nutrition.
- Monitor oxygen saturations.
- Restrict fluid intake.

MEDICINE TREATMENT

If saturation < 92%:

- Oxygen, via nasal prongs or nasal cannula.

Treat for bacterial pneumonia. See section 15.3.1: Pneumonia.

In all infants between 2 and 6 months with pneumonia consider PCP.

ADD

- Co-trimoxazole, IV/oral, 5 mg trimethroprim/25 mg sulphamethoxazole/kg/dose, 6 hourly for 21 days.
    - o Continue co-trimoxazole prophylaxis at the end of this treatment period until CD4 counts recovers to normal.

Children who remain hypoxic on oxygen with proven or highly suspected PCP:

- Prednisone, oral, 1–2 mg, daily for 7 days.
    - o Taper dose over the next 7 days.

For confirmed CMV disease:

- Ganciclovir, IV, 5 mg/kg 12 hourly for 21 days (initial dose).
    - o Follow with: 5 mg/kg daily for 21 days (maintenance dose).

Note:
Immunoglobulins, interferon and CMV vaccine have no benefit.

For children not responding to standard therapy:
Exclude TB, *S. aureus* and gram negative bacteria.

If extremely ill consider empiric treatment with:

- Vancomycin, IV, 15 mg/kg/dose, 6 hourly infused over 1 hour.

PLUS

- Amikacin, IV, 15–20 mg/kg once daily.

For children with acute pneumonia or chronic lung disease:
Treat as above for pneumonia but investigate for TB, NTM, candida and non-typable *H. influenzae*.
Check immunisation status for pneumococcus and *H. influenzae*.

REFERRAL

» For commencement of combined ART, where indicated.
» In cases of CMV disease for follow up for hearing deficit.

15.3.2.5 PNEUMONIA, NOSOCOMIAL

J18.9

DESCRIPTION

Children acquiring pneumonia 48–72 hours after hospitalisation.
The common pathogens are:

» ß-lactamase producing pathogens,
» extended spectrum ß-lactamase producing *Klebsiella pneumoniae*,
» *P. aeruginosa*,
» multidrug resistant Acinetobacter species,

- » methicillin resistant *S. aureus*,
- » respiratory viruses e.g. respiratory syncytial virus, adenovirus, influenza, herpes, measles, parainfluenza.

GENERAL AND SUPPORTIVE MEASURES

- » Septic screen including blood and urine cultures.
- » Nasopharyngeal aspirates for antigens.
- » Molecular test for respiratory virus.

MEDICINE TREATMENT

Empirical antibiotic therapy

Broad spectrum antibiotics according to local susceptibility patterns.

Manage children with underlying predisposing factors according to the susceptibility of the most likely pathogen.

Review antibiotic choice once culture and sensitivity results become available.

<u>For bacterial infections</u>

Start with:

- Piperacillin/tazobactam, IV, 100 mg/kg/dose 8 hourly.

PLUS

- Amikacin, IV, 15 mg/kg/dose, daily.

Adjust therapy according to sensitivities.

For methicillin resistant *S. aureus* pneumonia:

- Vancomycin, IV, 15 mg/kg/dose 6 hourly infused over 1 hour.

15.4 PLEURAL DISEASE

15.4.1 EFFUSION AND EMPYEMA

J90

DESCRIPTION

A pleural effusion is an accumulation of an exudative or transudative fluid between the visceral and parietal pleura. Common causes for exudates are infections, inflammation and malignancy. Common causes of a transudate are cardiac failure, renal failure and hepatic failure. A straw coloured or haemorrhagic effusion is indicative of tuberculosis. A cloudy or frankly purulent fluid indicates an empyema.

DIAGNOSTIC CRITERIA

- » Decreased breath sounds and stony dull on percussion.
- » Pleural rub early in disease.
- » Chest X-ray shows uniform opaque opacities in a lamellar distribution at the costophrenic angles.

GENERAL AND SUPPORTIVE MEASURES

- » Treat small effusions conservatively.
- » Drain other effusions by either chest drain or needle aspiration.
- » Send samples for protein, glucose, cytology, microscopy and culture. If pus is identified or pH < 7.2, insert chest drain.
- » Transudates do not require drainage.
- » More aggressive surgical procedures such as open drainage or decortication are rarely indicated in children.

MEDICINE TREATMENT

For purulent effusion:

- Cloxacillin, IV, 50 mg/kg/dose every 6 hours.

PLUS

- Gentamicin, IV, 6 mg/kg as a single daily dose for 10 days.

If there is evidence of good clinical response, change to:

- Flucloxacillin, oral, 12.5–25 mg/kg/dose, 6 hourly for a total of 21 days.

If pathogens are cultured in blood from sanctuary sites e.g. bone, heart valves, etc. treat according to sensitivity for prolonged period of 21–42 days.

For straw coloured or haemorrhagic effusion:

- » Start antituberculosis therapy.

If no response, to any of the above therapy, consider fungal infection.

15.5 UPPER AIRWAY DISEASES

15.5.1 EPIGLOTTITIS

J05.1

DESCRIPTION

Life-threatening upper airway obstruction at the level of the supraglottic structures (epiglottis and arytenoids).

The condition is rare since *H. influenzae* type b v accination has been introduced.

DIAGNOSTIC CRITERIA

» Acute onset, high fever, sore throat, dysphagia, refusal to eat or swallow, drooling and muffled voice.
» Position of comfort to protect the upper airway: sitting upright, head forward, open mouth, neck in extension.

GENERAL AND SUPPORTIVE MEASURES

» Do not interfere with the protective mechanism of the patient. Allow the child to remain sitting up.
» Avoid all measures that could agitate the patient:
  > make no attempt to see the epiglottis,
  > do not routinely perform X-rays of neck and chest,
  > delay blood sampling and IV line insertion until after airway is secured.
» Monitor oxygen saturation (pulse oximeter).

Acute airway obstruction

> **Caution**
> Epiglottitis is an upper airway emergency.
> Total upper airway obstruction is imminent by the time stridor appears.
> Prepare equipment for bag-mask ventilation, endotracheal intubation, needle cricothyroidotomy and tracheostomy.

» If airway obstructs completely or respiratory arrest occurs, attempt to establish an airway: ventilate with bag and mask.
» If unable to ventilate: intubate.
» If unable to intubate: perform needle or surgical cricothyroidotomy.

Total airway obstruction may occur suddenly and quite unpredictably, the patient should ideally be intubated before referral. Intubation should preferably be performed under general anaesthesia in an operating theatre.

If intubation prior to referral is not possible, transfer patient as an emergency advising transfer staff to avoid lying the child down. Inform the receiving hospital before departure.

During transport, if the child decompensates, attempt bag and mask ventilation.

After an open airway has been secured:
» take blood for cultures,
» swab epiglottis for microscopy, culture and sensitivity,
» monitor heart rate, respiratory rate, blood pressure and SaO_2,
» ensure adequate nutrition and hydration.

MEDICINE TREATMENT

- Oxygen, humidified, if needed.

- Ceftriaxone, IV, 50 mg/kg/dose, once daily for 7 days.

REFERRAL

» All, once airway is secured.

15.5.2 LARYNGOTRACHEOBRONCHITIS, ACUTE VIRAL (CROUP)

J05.0

DESCRIPTION

Potentially life-threatening airway obstruction in children and one of the most common causes of stridor in children aged between 6 months and 2 years.
The most common viruses causing laryngotracheobronchitis (LTB) include:

» para-influenza virus (most common),
» measles,
» herpes simplex,
» adenovirus.

DIAGNOSTIC CRITERIA

Clinical

» a previously healthy child who, a day or two after the onset of an upper respiratory tract infection, develops progressive airway obstruction with a barking cough and stridor,
» a mild fever may be present,
» stridor becomes softer as airway obstruction becomes more severe.

The following features suggest a different diagnosis:

» acute onset of obstruction without prodromal features (foreign body or angioneurotic oedema),
» incomplete immunisation and a membrane in the upper airway (diphtheria),
» high fever, dysphagia, drooling or sitting position (epiglottitis, retro-pharyngeal abscess, bacterial tracheitis),
» recurrent upper airways obstruction (laryngeal papilloma).

Assessment of severity of airway obstruction in LTB

Severity	Inspiratory obstruction (Stridor)	Expiratory Obstruction (Stridor)	Pulsus paradoxus
Grade 1	+		
Grade 2	+	+<br>passive expiration	
Grade 3	+	+<br>active expiration using abdominal muscles	+
Grade 4	cyanosis, apathy, marked retractions, impending apnoea		

GENERAL AND SUPPORTIVE MEASURES

» Monitor the nutritional status and fluid requirements.
» Monitor oxygen saturation, heart rate and respiratory rate.
» Avoid arterial blood gas estimations. Clinical criteria are more effective in determining severity.
» Depending on severity, admit child to high care or intensive care ward.

MEDICINE TREATMENT

Grade 1 obstruction

- Prednisone, oral, 2 mg/kg as a single dose.

OR

- Dexamethasone, IV/IM, 0.5 mg/kg as a single dose.

Note:
Avoid steroids in patients with measles or herpes infection.

Grade 2 obstruction

As above
PLUS

- Epinephrine (adrenaline), 1:1000, nebulise with oxygen, every 15–30 minutes until expiratory obstruction is abolished.
  - o 1 mL epinephrine (adrenaline) 1:1 000 diluted in 1 mL sodium chloride 0.9%.

Grade 3 obstruction

As above:
» if improvement, treat as in grade 2 but reduce frequency of adrenaline (epinephrine) nebulisations with time,
» if no improvement within 1 hour, intubate, preferably under general anaesthetic,
» refer.

Grade 4 obstruction
As above and:
» continue steroids,
» continue with adrenaline (epinephrine) nebulisation with 100% warm humidified oxygen,
» emergency intubation or intubation under general anaesthesia, if circumstances permit,
» if unable to intubate, bag and mask ventilate and refer urgently.

For fever until fever subsides:
- Paracetamol, oral, 15 mg/kg/dose, 6 hourly.

For suspected herpes:
- Aciclovir IV, 10–15 mg/kg/dose 8 hourly for 5–7 days.

For suspected bacteria infection in children < 20 kg:
- Ampicillin, IV, 12.5–25 mg/kg/dose 6 hourly for 5–10 days.

For suspected bacteria infection in children > 20 kg:
- Ampicillin, IV, 250–500 mg, 6 hourly for 7 days.

AND
If bacterial tracheitis is suspected:
- Cloxacillin, IV, 50 mg/kg/dose 6 hourly for 7 days.

REFERRAL
Note:
» Intubate all children with grade 3 and 4 airway obstruction not responding within one hour to adrenaline nebulisations before referral.
» Children with an uncertain diagnosis.

CHAPTER 16
EYE CONDITIONS

16.1 EYE INFECTION, COMPLICATED (SEVERE EYE INFECTION)

H44

DESCRIPTION

Intensely painful, red eye with or without a discharge (excluding simple or non-painful conjunctivitis).

Assess clinically for:

- » Herpes conjunctivitis indicated by vesicles on skin next to eye.
- » Loss of vision.
- » Irregularity of pupil.
- » Haziness of the cornea.

Investigations

Swab eye for microbiological culture.

GENERAL AND SUPPORTIVE MEASURES

Patient education on personal hygiene to avoid spread.
Educate patient on correct application of ophthalmic drops.
Advise patient:

- » to wash hands thoroughly before applying ophthalmic ointment,
- » not to share ophthalmic ointments or drops,
- » not to rub eyes, and
- » never to use urine or milk to wash the eyes.

MEDICINE TREATMENT

If herpes infection suspected, treat as outlined in section 16.3: Herpes keratitis and conjunctivitis.

During the day:

- Gentamicin, ophthalmic drops, instill 1 drop 4–6 hourly.

OR

- Chloramphenicol 0.5%, ophthalmic drops, instill 1 drop 4–6 hourly.

AND

Apply at night:

- Chloramphenicol 1%, ophthalmic ointment.

REFERRAL

To ophthalmologist within 24 hours if associated with any of the following:

- » Reduced vision.
- » A cloudy cornea.
- » A corneal opacity or a staining corneal ulcer.
- » Pus and blood level in the anterior chamber (hypopion and hyphaema).
- » Cloudiness in the anterior chamber (poor view of iris details).
- » An irregular or dilated (including partially dilated) pupil.
- » A cloudy or poor view of the retina.
- » A poor or greyish red reflex.
- » Proptosis.
- » Restricted ocular movements.
- » Severe ocular pain.

Non urgent referral

- » A unilateral red eye for more than one day.
- » No improvement after 5 days of treatment.

16.2 CONJUNCTIVITIS, ALLERGIC

H10.1

See Standard Treatment guidelines and Essential Medicines List for Primary Health Care Level.

16.3 HERPES KERATITIS AND CONJUNCTIVITIS

H90.1*

DESCRIPTION

Herpes infection of the cornea and/or conjunctiva.

DIAGNOSTIC CRITERIA

There are three most common forms of this disease.

Blepharoconjunctivitis

Primary ocular infection involving the eyelids, and/or conjunctivae.
The condition is benign and self limiting.
May be associated with keratitis: tiny punctuate stains on the cornea when stained with fluorescein and viewed with the cobalt blue light of the direct ophthalmoscope.

Disciform keratitis
Immune response to herpes virus.
Decreased visual acuity and corneal sensation.
Round dull swollen area in the central cornea.
Refer to ophthalmologist.

Dendritic ulcer
A linear branching ulcer (dendritic ulcer) when stained with fluorescein and viewed with the cobalt blue light of the direct ophthalmoscope.
Decreased sensation when compared to the ot her eye. (Use a thread o f cotton from a cotton bud and touch the cornea from the side, away from the visual axis)

GENERAL AND SUPPORTIVE MEASURES
Pad the eye.

MEDICINE TREATMENT
- Aciclovir, ophthalmic ointment, applied five times per day for 10 days.

If cilliary spasm present:
- Cyclopentolate, ophthalmic drops, instil 1 drop 8 hourly.

REFERRAL
Urgent within 24 hours:
» If corneal lesion is not clean/clear or has whitish areas within the bed of the epithelial ulcer.
» If area of corneal staining is not smaller within 24 hours of treatment.
» If there is a history of recurrence.
» Disciform keratitis for assessment and treatment.

16.4 CYTOMEGALOVIRUS (CMV) RETINITIS
B25.8/H30.9

DESCRIPTION
Characteristic appearance: opacification of t he retina with areas of haemorrhage, exudate and necrosis.
Occurs in immunocompromised patients and could be an important cause of visual impairment in HIV infected patients.

DIAGNOSTIC CRITERIA
» Confirm retinitis with ophthalmological assessment.
» Confirm CMV disease with DNA PCR.

MEDICINE TREATMENT

- Ganciclovir, intravitreal, 2 mg once a week. (Opthalmologist treatment). Once immune function has been restored with antiretroviral therapy, i.e. CD4 > 100 ce lls/mm^3, maintenance ganciclovir can be stopped but monitor for recurrence.

REFERRAL

» All patients to confirm diagnosis and manage treatment.

16.5 CHEMICAL BURN TO THE EYE

T26.9

DESCRIPTION

Damage to one or both eyes caused by contact with irritating chemical substances, either alkali or acid.
Presentation:

» pain,
» inability to open eye,
» blurred vision,
» excessive tearing.

DIAGNOSTIC CRITERIA

» To assess extent of epithelial loss, after irrigating the eye/s, stain the cornea with fluorescein 2%.
Note:
If the entire cornea stains, then all the epithelium has been removed by the chemical substance. Compare fluorescein staining in the other eye.

GENERAL AND SUPPORTIVE MEASURES

Try to ascertain the exact nature of the chemical agent (without causing a delay in management and referral) by the checking of the pH of the conjunctival sac with litmus paper. (Alternatively the pH square of a urine dipstix strip may be used) Normal tear pH: 6.5–7.6.
Irrigate affected eye/s immediately and continuously with copious amounts of sterile water (at least 2L). Use an eye speculum and an IV fluid delivery set.

If chemical agent is alkaline, prolong irrigation.
Note: Do not attempt to neutralise alkali with acid or vice versa.

MEDICINE TREATMENT

Anaesthetise eye/s after rinsing the eye and before instilling fluorescein

- Amethocaine ophthalmic drops, instil 1 drop.
    - Repeat every 15 minutes, if necessary.

For pain:
- Paracetamol, oral, 15 mg/kg/dose, 6 hourly as required.

REFERRAL
Urgent

» Any severe chemical burn producing any epithelial loss or cloudiness of the cornea and/or conjunctival blanching.

16.6 PENETRATING EYE INJURY WITH/WITHOUT A FOREIGN BODY
S05.5/S05.6

DESCRIPTION
Penetration through the cornea or sclera to deeper structures with/without a foreign body still present.

DIAGNOSTIC CRITERIA
Urgently refer patient with a penetrating eye injury or a severely contused eye to an ophthalmic specialist to avoid endophthalmitis and loss of the eyeball**.**

GENERAL AND SUPPORTIVE MEASURES
Note:
Use only preservative-free sterile eye drops if there is a possibility of an open eye injury.
Apply a clean sterile eye shield that does not cause pressure on the globe and transfer patient to the nearest specialist eye unit. If no eye shield is available, the bottom $^{1}/_{3}$ of a paper cup may be used.

In cases of high velocity injury with radio-opaque material (metals, certain glass types), an orbital X-ray will reveal a suspected retained intra-ocular foreign body.

SURGICAL TREATMENT
Should be done by an ophthalmic specialist with an operating microscope.

REFERRAL
Urgent

» Any severe blunt trauma to the eye.
» A penetrating eye injury with/without foreign body.
» Corneal or scleral laceration.
» Distorted pupil.
» Flat, shallow or deep anterior chamber (comparative to the other eye).
» Blood inside the eye.

16.7 NON-PENETRATING EYE INJURY

S05.1

DESCRIPTION

An intact cornea and sclera, but severely contused eye.
Foreign body on or embedded in the cornea of an intact eye.

DIAGNOSTIC CRITERIA

Signs depend on site affected and nature of non-penetrating trauma.

<u>Corneal injury</u>
Contusion: hazy oedematous cornea.
Foreign body embedded on/in cornea.

<u>Iris injury</u>
Sphincter rupture: dilated or irregular pupil margin.
Hyphaema: blood in the ant erior chamber due to rupt ure of the blood vessels.

<u>Lens injury</u>
Cataract: reduced red reflex.

<u>Lens suspensory ligaments</u>
Subluxed or dislocated lens: abnormal lens position.

<u>Retinal injury</u>
Blood vessel injury: blood in vitreous, blood on/in the retina.
Retinal breaks and tears.

<u>Choroidal injury</u>
Choroidal break: blood visible under the retina.

<u>Optic disc</u>
Disc swelling or pallor.

MEDICINE TREATMENT

Corneal injury

A <u>superficial</u> corneal foreign body may be removed with a bud or hypodermic needle.

To anaesthetise the cornea for removal of foreign body:

- Amethocaine ophthalmic drops, instil 1 drop. Repeat every 15 minutes, if necessary.

To relieve discomfort caused by iris spasm:

- Cyclopentolate 0.5–1%, ophthalmic drops, 1 drop instilled immediately.

Until epithelialisation is complete:

- Chloramphenicol, ophthalmic ointment, applied 8 hourly for 5–10 days.

Iris injury

Sphincter rupture

Manage conservatively. Follow-up in four days to exclude hyphaema.

Hyphaema

Bed rest for five days.

Monitor for complications, i.e. increased intraocular pressure, corneal staining, secondary bleed.

- Atropine 1%, ophthalmic drops, instil one drop 12 hourly for 5 days.

PLUS

Topical steroid drops:

- Dexamethasone, ophthalmic drops, instil one drop 4 hourly for 5 days.

REFERRAL

» A deeply embedded or full thickness corneal foreign body.

» Hyphaema if unable to monitor for complications or if complications develop.

» Any eye with severe trauma and decreased visual acuity.

» Lens, retina and choroidal injuries – refer within 12 hours.

16.8 RETINOPATHY OF PREMATURITY (ROP)

H35.1

DESCRIPTION

ROP is a potentially preventable cause of blindness.

ROP is classified in five stages, ranging from mild (stage I) to severe (stage V):

Stage I – Mildly abnormal blood vessel growth.

» Many children who develop stage I improve with no treatment and eventually develop normal vision.

» The disease resolves on its own without further progression.

Stage II – Moderately abnormal blood vessel growth.

» Many children who develop stage II improve with no treatment and eventually develop normal vision.

» The disease resolves on its own without further progression.

Stage III – Severely abnormal blood vessel growth.

» The abnormal blood vessels grow toward the centre of the eye instead of following their normal growth pattern along the surface of the retina.
» Some infants who develop stage III improve with no treatment and eventually develop normal vision.
» However, when infants have a certain degree of Stage III and "plus disease" develops, treatment is considered.
» "Plus disease" means that the blood vessels of the retina have become enlarged and twisted, indicating a worsening of the disease.
» Treatment at this point has a good chance of preventing retinal detachment.

Stage IV – Partially detached retina.

» Traction from the scar produced by bleeding, abnormal vessels pulls the retina away from the wall of the eye.

Stage V – Completely detached retina and the end stage of the disease.

» If the eye is left alone at this stage, the baby can have severe visual impairment and even blindness.

TIMING OF SCREENING

» Gestational age less than 27 weeks: screen at 31 weeks gestational age.
» Gestational age more than 27 weeks: screen at 4–5 weeks post natal.

MEDICINE TREATMENT

Dilation of the pupils for ROP screening by ophthalmologist:

- Cyclopentolate 0.5%/phenylephrine 2.5%, ophthalmic drops, instil one drop every five minutes for three doses one hour before examination.

REFERRAL

» All neonates weighing less than 1 500 g or < 32 weeks gestational age to be screened for ROP by ophthalmological examination.

16.9 CONGENITAL GLAUCOMA

Q15.0

DESCRIPTION

Congenital glaucoma is caused by abnormal development of the draining angle of the eye.

DIAGNOSTIC CRITERIA

Symptoms:

» Tearing.
» Photophobia.
» Blepharospasm.

Signs:

» Enlarged eye (buphthalmos or "cow eye" appearance).
» Corneal haziness (due to corneal oedema or scarring).
» Optic disc cupping.
» Raised intraocular pressure.

REFERRAL

Urgent (to ophthalmologist):

» All patients.

16.10 LEUCOCORIA

H44.53

DESCRIPTION

Common causes of leucocoria (white pupil) include:

» retinoblastoma,
» cataract,
» persistent fetal vasculature, and
» end-stage ROP.

DIAGNOSTIC CRITERIA

» A white appearance of the pupil instead of the usual black colour.
» An absent or diminished red reflex of the fundus of the eye when examined with a direct ophthalmoscope or on a photograph of the child.

REFERRAL

Urgent (to ophthalmologist):

» All patients

16.11 STRABISMUS

H50.9

DESCRIPTION

Strabismus (squint) is a misalignment of the two eyes.

A non-paralytic squint (concomitant strabismus): will not h ave restrictions of ocular movements in any of the eye positions.

A paralytic squint (incomitant strabismus): will have a restriction in one or more of the six cardinal eye positions. Consider cranial nerve palsy (III, IV or VI). Do a full neurological examination.

Complications of strabismus

- Amblyopia: a sensory state of an eye where abnormal visual development occurs if that eye is not being used by the brain. Untreated amblyopia leads to permanent visual impairment.
- Diplopia: when a strabismus occurs after the development of binocularity, the child will perceive a sensation of double vision (diplopia). Binocularity develops during the 1st decade.

DIAGNOSTIC CRITERIA

- The corneal light reflex: Patient asked to fixate on light held by the examiner at a distance of 33 cm. The light glistening on the cornea is displaced relative to the pupil.
- The cover test: Cover one eye and then the other. This elicits a re-fixation movement of the non-fixating eye.

REFERRAL

- All children with a squint.
- Urgent: Any acute onset of strabismus.
- Within 24 hours: incomitant strabismus.
- Within 1 week: if complications of strabismus present.
- Within 1 month: concomitant strabismus.

16.12 LOSS OF VISION

H53.1

DESCRIPTION

Causes of sudden loss of vision in an outwardly normal eye include:

- retinal detachment,
- occlusion of the retinal artery or retinal vein/s,
- vitreous haemorrhage,
- optic and retrobulbar neuritis,
- choroiditis.

Causes of gradual loss of vision in an outwardly normal eye include:

- refractive errors,
- cataracts,
- retinopathies,
- malignancies,
- optic nerve and chiasmal disease.

Loss of vision may also be associated with trauma, inflammation or other abnormalities.

REFERRAL

» Urgent: all children with sudden visual loss for full ophthalmic assessment and management.
» As soon as possible: all children with gradual visual loss which is not fully corrected by refraction.

CHAPTER 17

EAR, NOSE AND THROAT

17.1 ABSCESS, RETROPHARYNGEAL

J39.0

DESCRIPTION

An infective process of the retropharyngeal space either due to:

- » lymphatic spread,
- » extension of infection from surrounding tissues, or
- » local injury.

Always consider cold abscess of TB as a possible cause.

DIAGNOSTIC CRITERIA

Clinical

- » Stridor and difficulty in breathing,
- » dysphagia and drooling,
- » extension of the neck, and
- » swelling on one side of posterior pharyngeal wall.

Investigations

- » Lateral X-ray of the neck may show the retropharyngeal space to be more than one half of the width of the adjacent vertebral bodies when the neck is extended, air may be seen in the retropharynx and there is loss of the cervical lordosis.

GENERAL AND SUPPORTIVE MEASURES

- » Surgical drainage of abscesses.
- » Protect the airway.
- » Ensure adequate hydration, IV fluids or by NG tube.

MEDICINE TREATMENT

Empirical antibiotic therapy

Initiate antibiotic treatment immediately even if transfer of the patient is anticipated.

Adjust antibiotic therapy based on culture results, if available.

Early complications may be treated with antibiotic therapy alone.

- ▪ Third generation cephalosporin, e.g.

Ceftriaxone, IV, 80 mg/kg/dose once daily.

PLUS

- • Metronidazole, IV, 7.5 mg/kg/dose 8 hourly.

As soon as there is a response and patient can tolerate oral medication:

- Amoxicillin/clavulanic acid, oral, 30 mg/kg/dose of the amoxicillin component 8 hourly.

Note:
S. aureus and *M. tuberculosis* are also etiological agents. Adjust antibiotics once sensitivity results are obtained.

Penicillin allergy
See section 23.4.1: Allergies to penicillins.

For pain and fever:

- Paracetamol, oral, 15 mg/kg/dose 6 hourly as required.

REFERRAL

» All children with suspected retropharyngeal abscess.

17.2 TONSILLITIS, COMPLICATED (PERITONSILLAR CELLULITIS, PERITONSILLAR ABSCESS)

J03.9

DESCRIPTION

An infective process involving the tonsils with spread of infection into the adjacent tissue. It must be differentiated from hypertrophy of the tonsils without infection.
Local complications include peritonsillar cellulitis and abscess (quinsy), and suppurative cervical adenitis.
Systemic complications include glomerulonephritis, rheumatic fever and bacterial endocarditis.

DIAGNOSTIC CRITERIA

Clinical

» Pyrexia, malaise.
» Sore throat, dysphagia, drooling, trismus.
» Enlarged, inflamed tonsils, often with superficial pus visible in crypts.
» Earache (referred to as otalgia).
» Tender and enlarged cervical lymph nodes.

Signs of peritonsillar abscess/cellulitis:

» Usually unilateral.
» Soft palate and uvula on the infected side are oedematous and displaced medially towards the uninvolved side.

Investigations

» Microscopy, culture and sensitivity.

GENERAL AND SUPPORTIVE MEASURES

» Drain abscesses surgically.
» Maintain the airway, if necessary.

MEDICINE TREATMENT

Empiric antibiotic therapy

Initiate antibiotic treatment immediately even if transfer of the patient is anticipated.
Adjust antibiotic therapy based on culture results, if available.
Early complications may be treated with antibiotic therapy alone.

▪ Third generation cephalosporin, e.g.:
- Ceftriaxone, IV, 80 mg/kg/dose once daily.

PLUS

- Metronidazole, IV, 7.5 mg/kg/dose 8 hourly

As soon as there is a response and patient can tolerate oral medication:

- Amoxicillin/clavulanic acid, oral, 30 mg/kg/dose of the amoxicillin component 8 hourly for 10 days.

Adjust antibiotics once sensitivity results are obtained.

Penicillin allergy
See section 23.4.1: Allergies to penicillins.

For pain and fever:

- Paracetamol, oral, 15 mg/kg/dose 6 hourly as required.

REFERRAL

» Tonsillitis with local complications not responding to adequate treatment.
» All cases where surgery may be required and is not available locally.

17.2.1 ACUTE BACTERIAL TRACHEITIS

J04.1

DESCRIPTION

An acute infective process characterised by marked subglottic oedema, with ulceration, erythema, pseudomembranous formation on the tracheal surface, and thick, mucopurulent secretion which frequently obstructs the lumen. Commonly due to *S. aureus*.

DIAGNOSTIC CRITERIA

Clinical

» Severely ill and toxic with airway obstruction and respiratory distress.
» Insidious onset, brassy cough, neck pain, dysphagia, no drooling.
» Associated co-infection, e.g. pneumonia.

Investigations

» Raised white cell count with left shift.
» Lateral neck X-ray: hazy tracheal air column.
» Upper airway endoscopy.
» Bacterial cultures on blood and pharyngeal secretions.

GENERAL AND SUPPORTIVE MEASURES

» Intubate and suction secretions if features of severe upper airway obstruction are present.
» Mechanical ventilation if associated pneumonia present.

MEDICINE TREATMENT

- Ceftriaxone, IV, 80 mg/kg once daily.

OR

If one month old or younger:

- Cefotaxime, IV, 50 mg/kg/dose, 6–8 hourly.

Adjust antibiotics according to sensitivity results.

For pain:

- Paracetamol, oral, 15 mg/kg/dose 6 hourly.

Give 3 doses of corticosteroids to intubated patients prior to extubation:

- Dexamethasone, IV, 0.15 mg/kg/dose 8 hourly.

REFERRAL

» All cases requiring intubation.

17.3 EPISTAXIS (NOSE BLEED)

R04.0

DESCRIPTION

Nose bleed may be caused by local or systemic diseases, or local trauma, especially nose picking. It occurs from an area anterior and inferior on the nasal septum. Recurrent nose bleeds should alert one to possible systemic diseases e.g. hypertension and bleeding tendency. Persistent or severe bleeds may require hospital care.

Complications include anaemia and hypovolaemic shock.

DIAGNOSTIC CRITERIA

» History of spontaneous and/or recurrent nose bleeds.
» Underlying problems include bleeding disorders and local intranasal pathology.
» Examine child for nasal lesions and signs of haematological disease and coagulopathies.

GENERAL AND SUPPORTIVE MEASURES

Digital pressure

» Squeeze the nasal wings (alae) of the nose between the thumb and forefinger to apply pressure to the nasal septum and maintain pressure for about 10 minutes.
» The child should sit up and lean forward so as not to swallow the blood, and should breathe through the mouth.
» If digital pressure fails, remove blood clots from the nose. The child may be able to do this by blowing his nose.

MEDICINE TREATMENT

Vasoconstrictor

If digital pressure fails:

- Oxymetazoline 0.025%, nose drops, instil 1–2 drops into the affected nostril(s) and repeat digital pressure as above.

Nasal pack

If bleeding continues and appears to originate from the anterior nasal cavity, pack the floor of the cavity (rather than the apex) with cotton gauze tape impregnated with:

- Bismuth iodine paste.

Topical anaesthesia prior to packing:

- Lidocaine spray 2% solution.
  - o Do not exceed 3 mg/kg dose.

Anaemia

If symptomatic anaemia:

» haemoglobin is less than 8 g/dL and/or haematocrit is < 25% with ongoing epistaxis, or
» there is an underlying disorder in which severe re-bleeding is likely,

- Packed red cells, IV, 10–15mL/kg.

Treat the underlying disorder appropriately.

REFERRAL

» Epistaxis caused by a serious underlying disorder.
» Epistaxis that is not controlled by the above measures.
» Recurrent epistaxis.

17.4 MASTOIDITIS

H70.9

DESCRIPTION

A serious condition involving infections of mastoid antrum that could spread to the adjacent brain and could occur secondary to an ear infection. Is usually due to bacterial or fungal infections but also consider tuberculosis in this condition.

DIAGNOSTIC CRITERIA

Clinical

- » Fever, severe pain, increasing hearing impairment, tenderness over mastoid antrum.
- » Swelling in post-auricular area. Pinna is pushed down and forward.
- » Tympanic membrane is usually perforated with otorrhoea.
- » Occasionally, pus breaks through the mastoid tip and forms an abscess in the neck (Bezold's abscess).
- » If neck stiffness and pain, exclude intracranial spread.

Investigations

- » X-rays of mastoid show opacity and air-cell coalescence.
- » CT scan can confirm the diagnosis.
- » Collect blood and pus for Gram stain, microscopy, culture and sensitivity tests before initiation of antibiotic therapy.

GENERAL AND SUPPORTIVE MEASURES

- » Dry mopping of the external auditory canal.

MEDICINE TREATMENT

Antibiotic therapy

Adjust antibiotic therapy based on culture results or if response to antibiotic therapy is unsatisfactory.
As soon as there is clinical improvement and patient can tolerate oral medication, change to oral antibiotics based on culture and sensitivity.
Total duration of therapy: at least 14 days.

- Ceftriaxone, IV, 80 mg/kg once daily.

OR

If one month old or younger:

- Cefotaxime, IV, 50 mg/kg/dose, 6–8 hourly.

As soon as there is a response and patient can tolerate oral medication:

- Amoxicillin/clavulanic acid, oral, 30 mg/kg/dose of amoxicillin component, 8 hourly.

For pain:

- Paracetamol, oral, 15 mg/kg/dose 6 hourly as required.

REFERRAL

Urgent

» To ENT surgeon after initiation of antibiotics.

17.5 OTITIS EXTERNA

H60.9

DESCRIPTION

Inflammation of the external ear.

GENERAL AND SUPPORTIVE MEASURES

» Exclude underlying chronic otitis media prior to treatment.

» Keep the ear clean and dry using a wick of rolled absorbent cloth.

MEDICINE TREATMENT

- Acetic acid 2% in alcohol, instil 3–4 drops 6 hourly into the cleaned and dried ear.

For pain:

- Paracetamol, oral, 15 mg/kg/dose 6 hourly as required.

17.6 OTITIS MEDIA, ACUTE

H66.9

DESCRIPTION

Inflammation of the middle ear that may be complicated by perforation and a purulent ear discharge, which usually resolves spontaneously within 14 days.

DIAGNOSTIC CRITERIA

» Acute purulent otorrhoea not due to otitis externa.

OR at least <u>one</u> of the following:

» Distinct fullness or bulging of the tympanic membrane.

» Marked redness of the tympanic membrane.

» New onset of ear pain, not due to referred pain.

GENERAL AND SUPPORTIVE MEASURES

» Avoid getting the inside of the ear wet.

MEDICINE TREATMENT

- Amoxicillin, oral, 30 mg/kg/dose 8 hourly for 5–10 days.

For pain:

- Paracetamol, oral, 15 mg/kg/dose 6 hourly as required.

17.7 OTITIS MEDIA, SUBACUTE WITH EFFUSION

H66.0

DESCRIPTION

Inflammation of the middle ear with a purulent or serous ear discharge that usually resolve spontaneously within 14 days. Where effusion does not resolve within 14 days and last up to 3 months, it is considered to be subacute otitis media with an effusion.

DIAGNOSTIC CRITERIA

» Bubbles or air-fluid interfaces.

OR at least <u>two</u> of following:

» Abnormal color of tympanic membrane: white, yellow, amber, blue.
» Opacification not due to scarring and retraction.
» Decreased or absent mobility.

GENERAL AND SUPPORTIVE MEASURES

» Avoid getting the inside of the ear wet.

MEDICINE TREATMENT

- Amoxicillin/clavulanic acid, oral, 30 mg/kg/dose of amoxicillin component, 8 hourly for 5–10 days.
- Chlorphenamine, oral, 0.1 mg/kg/dose 6 hourly.

REFERRAL

» Chronic otitis media.

17.8 OTITIS MEDIA, CHRONIC, SUPPURATIVE

H66.3

DESCRIPTION

A purulent discharge from the middle ear with perforation for more than two weeks and/or effusion persists beyond 3 months.

Note:

TB is an important cause of a chronic discharge from the ear. Persistent or chronic otitis media is also associated with HIV infection in children.

GENERAL AND SUPPORTIVE MEASURES

» Dry mopping is the most important part of the treatment. It should be demonstrated to the child's caregiver or patient if old enough.

» Continue with dry mopping for 4 weeks.
  > Roll a piece of clean absorbent cloth into a wick.
  > Carefully insert the wick into the ear with twisting action.
  > Remove the wick and replace with a clean dry wick.
  > Repeat this until the wick is dry when removed.
  > Soak a clean wick in acetic acid 1% in sodium chloride 0.9%.
  > Insert carefully into the ear.
  > Leave in place for 1 minute.
  > Remove the wick and replace with a clean dry wick.
  > Observe the patient or caregiver repeat this until the wick is dry when removed.
  > Dry the ear by wicking at home three to four times daily until the wick stays dry.
  > If bleeding occurs, drying the ear should be stopped temporarily.

» **Do not leave anything in the ear.**

» **Do not instil anything else in the ear.**

» **Avoid getting** the inside of the ear wet during swimming and bathing by using ear plugs only during these activities.

MEDICINE TREATMENT

▪ Fluoroquinolone eardrops, e.g.:

• Ofloxacin drops, instil 2 drops 8 hourly into the affected ear after dry mopping, for 4 weeks.

REFERRAL

Emergency

» All with suspected intracranial complication.

Elective

» Large central perforation.

» No improvement after 4 weeks.

17.9 RHINITIS, ALLERGIC

J30.4

DESCRIPTION

Recurrent inflammation of the nasal mucosa due to hypersensitivity to inhaled allergens. May present with a running, itchy nose and excessive sneezing (runner) and/or with nasal obstruction (blocker).

GENERAL AND SUPPORTIVE MEASURES

» Avoid allergens and irritants.

MEDICINE TREATMENT

- Corticosteroid aqueous nasal solution, e.g.:
- Beclomethasone, 50 mcg, 1 puff into each nostril 12 hourly.

AND/OR

- Cetirizine, oral, as a single dose at night:
  - o Children 3–12 years: 5 mg.
  - o Children older than 12 years: 10 mg.

17.10 SINUSITIS, ACUTE

J01

DESCRIPTION

Inflammation or infection of one or more of the sinuses that occurring most often after a viral infection or with allergic rhinitis.

GENERAL AND SUPPORTIVE MEASURES

» Steam inhalation to liquefy and remove secretions blocking the nose.

MEDICINE TREATMENT

For infection:

- Amoxicillin, oral, 30 mg/kg/dose 8 hourly for 10 days.

For pain:

- Paracetamol, oral, 15 mg/kg/dose 6 hourly as required.

- Oxymetazoline 0.025%, nose drops, instil 2 drops into each nostril 6–8 hourly.
  - o Do not use continuously for more than 5 days.

17.11 SINUSITIS, CHRONIC

J32

DESCRIPTION

Chronic purulent postnasal drip for more than two weeks, characterised by nasal congestion, headache, facial pain or percussion tenderness.

DIAGNOSTIC CRITERIA

Investigations

» X-ray or CT scan may show opacities and fluid levels.

GENERAL AND SUPPORTIVE MEASURES

- » Identify and treat the underlying cause, e.g. nasal allergy.
- » Hypertonic sodium chloride nose drops may improve outcome.

MEDICINE TREATMENT

There is no clear evidence that antibiotics improve the outcome.

For pain:

- Paracetamol, oral, 15 mg/kg/dose 6 hourly as required.

REFERRAL

- » Failure to achieve progressive improvement.

17.12 SINUSITIS, COMPLICATED

J32.9

DIAGNOSTIC CRITERIA

Clinical

- » Signs and symptoms of complications:
  - > Peri-orbital swelling and fever.
- » Signs of meningeal irritation:
  - > Neck stiffness, positive Kernig's and Brudzinski's signs.
- » Signs of increased intracranial pressure:
  - > Hypertension, bradycardia, papilloedema and headache.
- » Signs of involvement of orbital structures:
  - > Periorbital oedema, erythema, chemosis, proptosis, vision loss and ophthalmoplegia.
- » Signs of brain involvement:
  - > Neurological signs, ataxia, paresis, paralysis, convulsions and altered level of consciousness.

Investigations

- » X-ray or CT scan may show opacities and fluid levels.
- » CT scan will show if there is involvement of intracranial structures, e.g. brain abscess.
- » Pus, CSF and blood for culture and sensitivity tests. Microscopy and Gram-staining of pus and CSF specimens may give some indication of the micro-organism/s involved.

MEDICINE TREATMENT

Empiric antibiotic therapy

Initiate empiric antibiotic therapy and reassess as soon as culture and sensitivity results become available or if there is no improvement within 48–72 hours.

Total duration of therapy: 14 days.

- Ceftriaxone, IV, 50–80 mg/kg once daily.

OR

If one month old or younger:

- Cefotaxime, IV, 25–50 mg/kg/dose, 6–8 hourly.

As soon as there is a response and patient can tolerate oral medication:

- Amoxicillin/clavulanate, oral, 30 mg/kg/dose of amoxicillin component, 8 hourly.

<u>Penicillin allergy</u>

See section 23.4.1: Allergies to penicillins.

For pain:

- Paracetamol, oral, 15 mg/kg/dose 6 hourly as required.

REFERRAL

Urgent

» Spread of infection to eye/orbital structures or intracranial structures/brain.

17.13 SINUSITIS, UNCOMPLICATED

See the Standard Treatment Guidelines and Essential Medicines List for Primary Health Care Level.

CHAPTER 18

POISONING

18.1 POISONING

DESCRIPTION

Frequently encountered poisonings in children include:

- » analgesics,
- » hydrocarbons,
- » pesticides,
- » plant material,
- » household cleaning products.
- » vitamins and minerals,
- » anticonvulsants,
- » phenothiazines,
- » sedatives and antidepressants,

Suspect intentional ingestion in older children (adolescents).

DIAGNOSTIC CRITERIA

Clinical

Can be divided into 'toxidromes":

Cholinergic:

- » salivation,
- » lacrimation,
- » urination,
- » defaecation,
- » miosis (pinpoint pupils).
- » diarrhoea,
- » vomiting,
- » bronchorrhoea,
- » bradycardia,

Salicylism:

- » tachypnoea,
- » metabolic acidosis,
- » seizures.
- » agitation,
- » coma,

Anticholinergic:

- » fever,
- » ileus,
- » flushing,
- » tachycardia,
- » urinary retention,
- » dry/warm skin,
- » blurred vision,
- » mydriasis(dilated pupil),
- » coma,
- » hallucinations and seizures.

Sedative-hypnotic:

- » obtundation or coma.

Opiates:

- » miosis,
- » respiratory depression,
- » bradycardia,
- » hypotension.
- » decreased bowel sounds,
- » hypothermia,
- » altered (decreased) mental status,

<u>Dystonic reaction</u>**:**

» torticollis,
» opisthotonus,
» intermittent spasms and tongue thrusting.

<u>Sympathomimetic:</u>

» hypertension,
» tachycardia,
» hyperthermia,
» agitation,
» sweating,
» dilated pupils.

Sympathomimetic toxidrome resembles anticholinergic toxidrome, i.e. fight, flight and fright response.

<u>Toxic alcohols:</u>

» metabolic acidosis,
» increased osmolar gap,
» visual disturbances (methanol),
» depressed level of consciousness.
» hypoglycaemia,
» convulsions,
» renal failure (ethylene glycol),

TREATMENT

If the ingestion has definitely occurred, establish whether toxicity is expected and act accordingly.
If the possibility of ingestion was remote, only observation is necessary.

» Stabilise patient, if necessary.
» If there is the risk of toxicity, decontaminate patient.

1. Gastric lavage

» Indicated only if patient has ingested a potentially life-threatening poison and the procedure can be undertaken within 60 minutes of ingestion.
» Contraindications:
  - if a corrosive substance or volatile hydrocarbon has been ingested,
  - if patient is unconscious unless the airway is protected.

2. Activated charcoal

» Administer only if the patient has ingested a potentially toxic amount of a poison which is known to be adsorbed by charcoal up to one hour previously. There is insufficient data to support or exclude its use after one hour of ingestion.

- Activated charcoal, oral, given as a slurry:
  - o If < 6 years of age: 10 g in 50–100 mL water.
  - o If > 6 years of age: 20–50 g in 100–300 mL water.

  Placement of a nasogastric tube may be necessary for its prompt administration.

  Avoid activated charcoal in paracetamol poisoning, if only oral n-acetyl cysteine is available for treatment, as it will adsorb the antidote.

Poisons where charcoal is ineffective and should not be given	Poisons where charcoal may be particularly useful if poison taken in toxic dose
» ethanol<br>» methanol<br>» essential oils, including brake fluid<br>» petrol or paraffin<br>» iron salts<br>» lithium<br>» bleach and caustic alkalis<br>» boric acid<br>» mineral acids	» carbamazepine, barbiturates, phenytoin<br>» dapsone, quinine<br>» theophylline<br>» salicylates<br>» mushroom poisoning (Amanita phalloides)<br>» slow release preparations<br>» digoxin<br>» sotalol<br>» NSAIDs

3. Whole bowel irrigation

» Use only for poisoning due to iron, lithium, lead, and if no contra-indications exist.
- Polyethylene glycol balanced electrolyte solution, oral, 30 mL/kg/hour.
  - o Maximum dose: 1.8 L/hour.
  - o Continue until rectal effluent is clear.

» Antidote, if available.
» Enhance elimination, if possible.
» Monitor hydration status carefully.

REFERRAL

» Severely ill patient for ventilatory/circulatory support.
» Where relevant diagnostic testing is not available, e.g. paracetamol levels.
» Where relevant medication/antidotes are not available.
» Where dialysis/haemoperfusion is required.
» For psychiatric evaluation.

18.1.1 ANTICHOLINERGIC POISONING

Y13

DESCRIPTION

Various plant species and pharmaceutical preparations can cause anti-cholinergic toxicity.
Plants: *Datura stramonium*, e.g. ‘stinkblaar and malpitte”.
Medicines including atropine, diphenoxylate with atropine and diphenhydramine.
Other classes of medicines include antiparkinsonism agents, antispasmodics, antipsychotics, antihistamines and tricyclic antidepressants.

DIAGNOSTIC CRITERIA

Clinical

» Alteration of mental status, including delirium, hallucinations, agitation and seizures.

» Peripheral anticholinergic effects include:

> mydriasis,

> tachycardia and arrhythmias,

> flushing,

> urinary retention,

> decreased GIT motility,

> dry skin and mucous membranes.

Investigations

» ECG and continuous cardiac monitoring.

» Pulse oximetry.

GENERAL AND SUPPORTIVE MEASURES

» Stabilise patient, i.e. airway, breathing and circulation.

» Cooling for hyperthermia.

» Perform decontamination depending on route of exposure.

MEDICINE TREATMENT

- Activated charcoal.

For agitation:

- Diazepam, IV/oral, 0.1–0.2 mg/kg.
  - o Maximum dose: 10 mg.

For seizures:

See section 13.5: Status epilepticus (convulsive).

REFERRAL

» Cardiac dysrhythmia.

» No response to treatment.

18.1.2 ANTICOAGULANT POISONING

Y18

DESCRIPTION

Poisoning with warfarin and “super-warfarin” (long acting warfarin), marketed as pellets for rats and mice.

Over the counter pesticides containing warfarin may be accidentally ingested by toddlers or young children. Bleeding tendencies may be delayed.

Beware, some poisons may contain organophosphates.

DIAGNOSTIC CRITERIA
Clinical
» Signs and symptoms depend on the potency.
» Symptoms may range from the asymptomatic child, e.g. a small child who has tasted a rodenticide, to significant cases which present with bruising or bleeding, e.g. if the child has ingested “super-warfarin” containing pesticides.

Investigations
» Measure prothrombin time.
  > Obtain baseline INR if symptomatic.
  > Obtain a second INR in all cases at 24–32 hours post ingestion and then 48 hours thereafter as the onset of co-agulopathy may be delayed.

GENERAL AND SUPPORTIVE MEASURES
» Observe asymptomatic child.

MEDICINE TREATMENT
Deranged INR:
- Vitamin K_1, IV/oral, 1– 5 mg/dose administered slowly 6 hourly.
  - o Repeat if large doses were administered.

Oral vitamin K_1 is usually preferred to intravenous vitamin K_1 unless more rapid reversal is required (e.g. the patient is bleeding). Intravenous vitamin K_1 may cause hypersensitivity reactions.

Bleeding:
ADD
- Lyophilised plasma IV, 20 mL/kg.

OR
- Fresh frozen plasma, IV, 20 mL/kg.

OR
- Factor IX, intravenous, 25–50 units/kg (0.5–1 mL/kg).

Ingestion of long acting warfarin may be refractory to large doses of vitamin K_1 and therapy may be required for several weeks after ingestion.

18.1.3 ANTIDEPRESSANT (TRICYCLIC) POISONING
Y11

DESCRIPTION
Poisoning with tricyclic antidepressants represent a large portion of poisoning fatalities. There is a high risk of tricyclic antidepressant toxicity in children because of its narrow therapeutic index. Serious toxicity may occur with low doses in children.

DIAGNOSTIC CRITERIA

- » 10–20 mg/kg of tricyclic antidepressant medication will cause significant toxicity in most children.
- » Antidepressant poisoning cause anticholinergic syndromes.
- » Mainly affects the cardiovascular system, autonomic nervous system, and central nervous system, leading to:
  - > conduction delays,
  - > dysrhythmias,
  - > hypotension,
  - > altered mental status,
  - > seizures.

GENERAL AND SUPPORTIVE MEASURES

- » Gastric lavage for large ingestions or patients presenting within a few hours post ingestion, except if the patient is unconscious.
- » Circulatory and respiratory support.
- » Cardiac and ECG monitoring for 48 hours.

MEDICINE TREATMENT

- Activated charcoal, given as a slurry.
  - o If < 6 years of age: 10 g in 50–100 mL water
  - o If > 6 years of age: 20–50 g in 100–300 mL water

  Placement of a nasogastric tube may be necessary for its prompt administration.

For cardiac arrhythmias:

- Anti-arrhythmic agents. Only under specialist supervision.

For hypotension:

- Sodium chloride 0.9% or Ringer–Lactate, IV bolus, 20 mL/kg.

Alkalinisation for metabolic acidosis

Alkalinisation up to an arterial pH of 7.45:

- Sodium bicarbonate 4.25%, IV, 2 ml/kg as a bolus.
  - o May be repeated.
  - o Follow with a continuous infusion in consultation with specialist/poison centre.

For circulatory and respiratory support:
See section 1.1.4: Cardiorespiratory arrest.

REFERRAL

- » Any cardiac arrhythmia.

18.1.4 CAUSTIC OR CORROSIVE AGENTS, INGESTION

Y19

DESCRIPTION

Caustic agents, e.g. sodium hydroxide or potassium permanganate, corrosive agents, e.g. hydrochloric acid.
Acids and alkali do not differ in their severity.
Note:
Battery acid causes significant corrosive damage, whereas household bleach seldom has a corrosive effect.

DIAGNOSTIC CRITERIA

Clinical

» Chief symptom is pain.
» Young children may present with:
  > crying,
  > drooling, and
  > refusal to swallow,
  > vomiting.
» Stridor or hoarseness indicates laryngeal injury.
» The presence of oral or pharyngeal burns does not predict the presence of oesophageal or gastric injury.
» Oesophageal or gastric injury can cause perforation or subsequent fistula formation.
» Patients with no clinical signs or symptoms are unlikely to have significant oesophageal or other organ injury.

GENERAL AND SUPPORTIVE MEASURES

Asymptomatic

» Monitor for development of symptoms.
  > A 12 hour symptom-free period usually indicates that no intervention is necessary.

Symptomatic

» Gastric lavage/emesis is contraindicated in all cases.
» Keep patient nil per mouth.
» Airway injury may necessitate endotracheal intubation.
» Endoscopic evaluation for patient with caustic injury.

MEDICINE TREATMENT

Prophylactic antibiotics are not indicated.
Consider steroid therapy after endoscopy has shown a 2nd or 3rd degree oesophageal injury to reduce oedema and fibrosis. In the setting of GIT bleeding or perforation, steroids are contra-indicated.

For pain control:
See section 20.1: Management of pain.

REFERRAL

» All symptomatic cases for endoscopic evaluation.

18.1.5 VOLATILE SOLVENTS

Y16

DESCRIPTION

Inhalants include: spray paints, glues and paint thinners which may contain toluene and or n-Hexane. These may contain hydrocarbons which may be aspirated following ingestion, causing a chemical pneumonitis.

DIAGNOSTIC CRITERIA

» distinctive odour,
» discolouration around mouth/nose,
» palpitations,
» dizziness,
» cardiac arrhythmias,
» euphoria,
» headaches,
» progressive CNS depression,
» syncope,
» hypokalaemia,
» mucous membrane irritation, i.e. sneezing coughing and tearing,
» GIT complaints, i.e. nausea, vomiting and abdominal pain,
» distal renal tubular acidosis, i.e. hyperchloraemic metabolic acidosis with a normal anion gap,
» peripheral neuropathy and hepatotoxicity may be complications.

GENERAL AND SUPPORTIVE MEASURES

» Stabilise airway, breathing and circulation.
» Perform a chest X-ray and monitor patient for respiratory symptoms.
» Correct fluid and electrolyte abnormalities.

MEDICINE TREATMENT

For agitation:

- Diazepam, IV/oral, 0.1–0.2 mg/kg.
  - o Maximum dose: 10 mg.

For cardiac dysrythmias, e.g.: ventricular fibrillation see section 4.1: Cardiac dysrhythmias.

REFERRAL

» Cardiac dysrhythmia.

18.1.6 ETHANOL POISONING

Y15

DESCRIPTION

Ethanol is a selective CNS depressant at low concentrations, and a generalised depressant at high concentrations.

DIAGNOSTIC CRITERIA

Clinical

- » lack of coordination,
- » ataxia,
- » slurred speech,
- » gait disturbances,
- » drowsiness.
- » stupor,
- » coma,
- » hypoglycaemia,
- » convulsions,

Investigations

- » Monitor blood glucose levels.

MEDICINE TREATMENT

Obtunded patients:

- Dextrose 10%, IV, 2 mL/kg.
  If patients respond to glucose administration, perform serial glucose levels to detect recurrent hypoglycaemia.

18.1.7 IRON POISONING

Y14

DESCRIPTION

Iron is widely available as an over-the-counter product and is commonly ingested accidentally by toddlers.

DIAGNOSTIC CRITERIA

Clinical

- » Toxicity is related to the ingested dose of elemental iron.
- » Single dose of elemental iron > 20 mg/kg requires hospital assessment and management.

Elemental iron per preparation

Iron product	Strength	Elemental content	Elemental content Per mL or tablet
Ferrous gluconate elixir	350 mg/5 mL	40 mg elemental iron/5 mL	8 mg elemental iron per mL
Ferrous gluconate syrup	250 mg/5 mL	30 mg elemental iron per 5 ml	6 mg elemental iron per mL
Ferrous lactate drops	25mg/mL	25 mg elemental iron/ mL	1 mg elemental iron in 0.04 mL
Ferrous sulphate compound tablets	170 mg	± 65 mg elemental iron per tablet	± 65 mg elemental iron per tablet

Categories of iron toxicity:

Low risk	Medium risk	High risk
» No history of:<br>> abdominal pain,<br>> nausea,<br>> vomiting, or diarrhea.<br>» Asymptomatic for 6 hours.<br>» < 20 mg/kg of elemental iron ingested.	» Minimal gastrointestinal symptoms.<br>» Normal physical examination.	» Lethargic.<br>» Acidotic.<br>» Shocked.<br>» May have evidence of haematemesis or melaena.

» Low risk patients are unlikely to have ingested enough iron to lead to serious poisoning and can be discharged.
» Admit high and medium risk patients.

Investigations

Medium risk
» Abdominal X-ray.
» Arterial blood gas.
» Electrolytes.
» Serum iron levels within 2–6 hours after ingestion.
» If no clinical features are present and serum iron < 500 mcg/dL, patient is low risk.

High risk
» Arterial blood gas.
» Electrolytes.
» Serum iron levels within 2–6 hours after ingestion.

GENERAL AND SUPPORTIVE MEASURES

Medium risk
» If more than mild gastrointestinal symptoms or altered mental state, shock, or acidosis refer for chelation therapy.

High risk
» Manage airway.
» Refer for chelation therapy.

MEDICINE TREATMENT

Medium and high risk

Fluid resuscitation:
- Sodium chloride 0.9%, IV, 20 mL/kg as an initial bolus followed with maintenance therapy.

If no signs of gastrointestinal dysfunction e.g. perforation/haemorhage:
- Whole bowel irrigation.

Chelation therapy
For iron ingestion > 60 mg/kg of elemental iron:
- Desferrioxamine, IV, 15 mg/kg/hour as a continuous infusion until urine is no longer pink.
  - o Beware of hypotension.

REFERRAL
» Urgent: if unable to do the above, urgent transfer is vital.

18.1.8 NEUROLEPTIC POISONING
Y11

DESCRIPTION
Neuroleptic overdose may cause a depressed level of consciousness, hypotension, tachycardia and cardiac dysrhythmias and seizures. Commonly used neuroleptics include chlorpromazine, Haloperidol and phenothiazine antiemetics.

Acute dystonic reactions/extrapyramidal symptoms are distressing adverse reactions (sustained muscle spasms) occurring after an overdose or during chronic therapy with neuroleptics. A typical dystonic reaction includes overextension or overflexion of the limbs with abnormal posturing of the trunk. Other extrapyramidal symptoms may occur.

The neuroleptic malignant syndrome is uncommon following an overdose and is an idiosyncratic life threatening reaction presenting with:
» temperature dysregulation,
» autonomic instability,
» altered mental state,
» diaphoresis,
» musculoskeletal effects (pipe like rigidity).

DIAGNOSTIC CRITERIA
» Dystonic reactions.
» Other extrapyramidal symptoms.

GENERAL AND SUPPORTIVE MEASURES
» Observe asymptomatic patients for a minimum of 6 hours.
» Admit all symptomatic patients for continuous cardiac monitoring.
» Test the urine of patients who are hypotensive, have muscular rigidity or seizures for myoglobin (dipstix test for haemoglobin) and serum creatinine monitored because of the risk of rhabdomyolysis.

MEDICINE TREATMENT

- Activated charcoal.

For acute dystonic reactions:

- Biperidin, IV, slow injection.
  - o If < 1 year of age: 1 mg
  - o If 1–6 years of age: 2 mg
  - o If 6–10 years of age: 3 mg

  If concomitant significant anticholinergic findings are present, such as fever and dry skin and mucous membranes, a benzodiazepine is preferred.

REFERRAL

» Patients with neuroleptic malignant syndrome.
» Patient with conduction abnormalities (prolonged QT).

18.1.9 ORGANOPHOSPHATE POISONING

Y18
* Notifiable condition

DESCRIPTION

Organophosphates are potent inhibitors of acetylcholinesterase. Poisoning due to organophosphates is notifiable.

DIAGNOSTIC CRITERIA

Clinical

» Cholinergic toxidrome.
» Cholinergic symptoms include:
  Muscarinic effects:
  > diarrhoea,
  > urination,
  > miosis,
  > salivation.
  > vomiting.
  > lacrimation.
  > bronchorrhoea/bronchoconstriction,
  Central nicotinic effects:
  > confusion,
  > coma,
  > convulsions.
» Cardiac effects include bradycardia or tachycardia depending on whether muscarinic or nicotinic effects predominate.
» Signs depend on dose and route of exposure (vapour or liquid) as well as the time exposed (vapour).

Investigations

» Decreased levels of pseudocholinesterase.
  > Use for confirmation only.
  > Do not wait for levels before treating.

GENERAL AND SUPPORTIVE MEASURES

- » Ensure use of personal protective equipment.
- » Remove all patients' clothing and wash clothes thoroughly.
- » Ventilate, if necessary.
- » Wash affected skin with soap and water.
- » Suction secretions frequently.
- » Monitor respiratory function closely, as well as heart rate, pupillary size and level of consciousness.

MEDICINE TREATMENT

For bradycardia, bronchorrhoea or bronchospasm:

- Atropine, IV, 0.02–0.05 mg/kg.
  - o Follow with a repeat dose every 10–15 minutes until bronchial secretions are controlled.
  - o Titrate dose against the secretions.
  - o The therapeutic endpoint is the clearing of secretions and resolution of bronchospasm.

    Note:

    Atropine may need to be continued for prolonged periods.
  - o Many repeated doses of atropine may be required and large quantities may be needed. Beware of relapses.
  - o Tachycardia and mydriasis are not contra-indications for atropine.

Treat convulsions.
See section 13.5: Status epilepticus (convulsive).

REFERRAL

- » Where ICU facilities are not available.

18.1.10 OPIOID POISONING

Y12

DESCRIPTION

Codeine is a common drug of abuse.

The duration of action of morphine is 3–6 hours. Other oral agents, e.g. codeine and long acting morphine, demonstrate a delayed effect of up to 4–12 hours.

DIAGNOSTIC CRITERIA

- » Altered level of consciousness.
- » Classic triad of CNS depression, respiratory depression and miosis (pupillary constriction).
- » Hypotension, hypothermia, bradycardia and hyporeflexia.

- » Vomiting is common and exposes the patient to the risk of aspiration especially in patients with depressed consciousness.
- » Early symptoms: awake and alert presenting within 1–2 hours of ingestion.

GENERAL AND SUPPORTIVE MEASURES

- » Supportive care, ventilate with bag-mask device.
- » Monitor oxygen saturation constantly.
- » Observe for urinary retention.
- » Airway protection is a priority.

MEDICINE TREATMENT

- Activated charcoal.

- Naloxone, IV, 0.1mg/kg.
  - o If no response after 5 minutes, repeat dose and titrate according to response.
  - o Duration of action of naloxone is 20–30 minutes.
  - o If repeated doses are naloxone are necessary, a continuous IV infusion of naloxone can be instituted.

CAUTION
All patients treated with naloxone should be observed for at least 12 hours for relapse, especially if a long acting opioid has been ingested.

REFERRAL

- » Patients requiring multiple doses of naloxone.

18.1.11 PARACETAMOL POISONING

Y10

DESCRIPTION

Paracetamol poisoning in c hildhood is almost always intentional. The accidental ingestion of paediatric paracetamol elixir preparations by the toddler very rarely achieves toxicity. Adolescents are often not aw are that paracetamol ingestion can be lethal and may unknowingly take a lethal dose as a suicidal gesture.

Toxicity can be due to acute ingestions or repeated supratherapeutic ingestion (RSTI).

DIAGNOSTIC CRITERIA

» Doses in excess of 150 mg/kg per 24-hour period in healthy children are potentially toxic.
» Serum paracetamol concentration must be measured at least four hours following ingestion.
» Use nomogram to assess risk of toxicity.

Paracetamol treatment nomogram

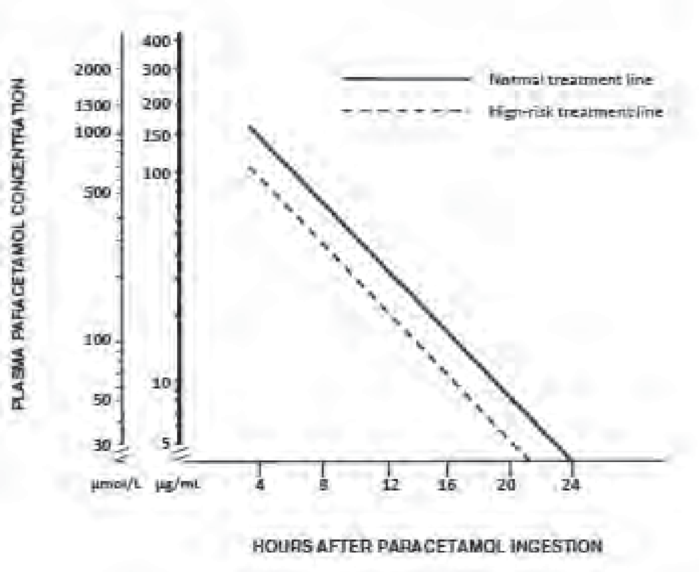

Nomogram reproduced from the Guidelines of Acute Paracetamol Overdose produced by the Medicines Information Centre, UCT. Published with kind permission.

This nomogram is not relevant for p oisoning with extended release formulations.

» Cautions for use of this chart:
  > The time co-ordinates refer to time of ingestion.
  > Serum levels drawn before 4 hours may not represent peak levels.
  > Use the graph only in relation to a single acute ingestion.
  > Not useful when there are co-ingestants, in delayed presentation > 24 hours post-ingestion.
  > The lower solid line 25% below the standard nomogram is included to allow for possible errors in paracetamol plasma assays and estimated time from ingestion of an overdose.

» If patients present > 8 hours post-ingestion, start on treatment without waiting for the paracetamol levels.
» If the time of ingestion is unknown, start treatment for any detectable level of paracetamol or any elevation of AST or ALT.
» Patients with normal LFTs and undetectable paracetamol levels four hours after ingestion do not require treatment.
» Administer without waiting for plasma paracetamol levels in substantial overdose, defined as ≥10 g (20 tablets) or ≥140 mg/kg, whichever is smaller. Discontinue if plasma levels are in the non-toxic range.
» Normal results at 48 hours excludes hepatic damage.
» Do:
  > Baseline urine and electrolytes.
  > Liver enzymes.
  > Coagulation profile.

The nomogram was not designed for use in overdoses with extended release paracetamol formulations. In this setting, administer N-acetylcysteine. Obtain a second paracetamol 4–6 hours after the original 4 hour concentration. If either value is above the treatment line of the nomogram, administer N-acetylcysteine.

GENERAL AND SUPPORTIVE MEASURES

» If the patient presents within one hour of ingestion do a gastric lavage.

MEDICINE TREATMENT

Only if patients presents within 1 hour of ingestion:

- Activated charcoal.

- Acetylcysteine, IV.

  First 24 hours
  - o Loading dose: 150 mg/kg in dextrose 5%, 5 mL/kg given over 15 minutes.
  - o 50 mg/kg in dextrose 5%, 5 mL/kg over the next 4 hours; then 100 mg/kg in dextrose 5%, 10 mL/kg over 16 hours.

  Second 24 hours
  - o 100 mg/kg in dextrose 5%, 10 mL/kg over 24 hours.

If IV not available:

- Acetylcysteine, oral, 140 mg/kg immediately as a single dose.
  - o Follow with 70 mg/kg every 4 hours for 17 doses.

REFERRAL

» Patients with severe hepatocellular damage.

18.1.12 PETROCHEMICAL POISONING

Y16

DESCRIPTION

Accidental ingestion of paraffin, particularly by toddlers, is common in South Africa.

DIAGNOSTIC CRITERIA

Clinical

» Paraffin is volatile and inhalation of the fumes or aspiration of liquid can cause serious chemical pneumonitis.
» Respiratory distress.
» CNS symptoms.

Investigations

» Chest X-ray.

GENERAL AND SUPPORTIVE MEASURES

CAUTION<br>**Do not attempt gastric lavage**.

» Observe patient for up to 12–24 hours if asymptomatic.
» Administer oxygen, if necessary.
» Education and counseling regarding prevention.

MEDICINE TREATMENT

If infection develops 48 hours after ingestion:

- Amoxicillin, oral, 30 mg/kg/dose 8 hourly for 5 days.

REFERRAL

» For ventilatory support.

18.1.13 SALICYLATE POISONING

Y10

DESCRIPTION

Salicylate poisoning may result from oral and/or topical exposure. Salicylate products vary widely in concentration e.g. oil of wintergreen is 100% methylsalicylate. As little as 4 mL of oil of wintergreen may be fatal in a child.

DIAGNOSTIC CRITERIA

Clinical

- » Doses less than 150 mg/kg will not cause toxicity except in a child with hepatic or renal disease.
- » Ingestion of 150–300 mg/kg may result in mild to moderate toxicity.
- » Ingestion of > 500 mg/kg should be considered a potentially lethal dose.
- » Features include:
  - > fever,
  - > epigastric pain,
  - > CNS depression,
  - > Hypoglycaemia,
  - > hyperventilation,
  - > renal failure,
  - > respiratory alkalosis (initially) followed by metabolic acidosis.
- » Monitor blood gases, urine output and urine and electrolytes.
- » Monitor salicylate level: toxic > 30 mg/dL.
- » Serial monitoring until declining levels are documented.
- » Monitor and treat hypoglycaemia.

GENERAL AND SUPPORTIVE MEASURES

- » Gastric lavage.
- » Correct hydration.

MEDICINE TREATMENT

After gastric lavage:

- Activated charcoal.
  - o May be used for up to 12 hours: due to delayed gastric emptying or if sustained-release/enteric-coated preparations were ingested.

Urine alkalinisation

If metabolic disturbances are present:

- Sodium bicarbonate, IV, 2 mmol/kg/day in maintenance fluid and administered over 3 hours.
  - o Increase, if necessary, to maintain urine pH above 7.5.

For hydration:

- ½ Darrows/dextrose 5%, IV.

For bleeding:

- Vitamin K_1 (phytomenodione), IM, 5 mg as a single dose.

REFERRAL

- » Severe cases for ICU care.

18.1.14 SEDATIVE-HYPNOTIC POISONING

Y11

DESCRIPTION

Young children or toddlers are typically involved in accidental exposure and ingest small amounts of sedatives.
Adolescents may ingest large amounts during suicide, suicidal gesture or for recreational use.
Examples of sedative-hypnotics include: benzodiazepines and diphenhydramine.

DIAGNOSTIC CRITERIA

Clinical

» Cardiorespiratory depression.
» Decreased level of consciousness.

Investigations

» Serum drug levels are of no value in the acute treatment phase.
» Urine test: may have medico-legal implications.

GENERAL AND SUPPORTIVE MEASURES

» If there is respiratory depression, intubate, ventilate and transfer.
» Gastric lavage.
» Supportive treatment only is necessary in most patients.

MEDICINE TREATMENT

If significant overdose is suspected:

- Activated charcoal.

REFERRAL

» Respiratory depression.

18.1.15 SULFONYLUREA

Y14

DESCRIPTION

First generation sulfonylureas include chlorpropamide, which is excreted renally. Second generation agents include glimepiride and glipizide and are excreted in the faeces.

DIAGNOSTIC CRITERIA

Clinical

» Coma and seizures.
» Profound hypoglycaemia, usually within 4 hours of ingestion.

Investigations

» Glucose monitoring is the mainstay of diagnostic testing.

GENERAL AND SUPPORTIVE MEASURES

» Observe for 24 hours even if a single tablet is ingested.
» Glucose containing fluid orally.

MEDICINE TREATMENT

- Activated charcoal.

- Dextrose 10% (2 mL/kg), IV boluses.
  - o Titrate until blood glucose is controlled.

Note:
Corticosteroids are not indicated.

REFERRAL

» Patients not responding to intravenous glucose.

18.1.16 SYMPATHOMIMETIC AGENT POISONING

Y13

DESCRIPTION

Pseudoephedrine in decongestants, methylphenidate and illicit drugs such as cocaine and amphetamines (Tik) are sympathomimetic agents.
These agents are frequently abused, especially as recreational drugs.

DIAGNOSTIC CRITERIA

Clinical

» Hypertension.
» Tachycardia.
» Tachypnoea.
» Psychosis.
» Mydriasis.
» Diaphoresis.
» Agitation.
» Hyperthermia: effects of sympathomimetics that predispose to hyperthermia include:
  > peripheral vasoconstriction and impaired cutaneous heat loss,
  > agitation,
  > seizures,
  > increased muscle activity,
  > impaired behavioral responses.
» With cocaine toxicity, cardiovascular manifestations predominate including:
  > supraventricular and ventricular dysrhythmias,
  > myocardial ischaemia.
» Neonates of mothers addicted to cocaine may present with withdrawal signs manifested by jitteriness.

Investigations

» ECG monitoring to evaluate dysrhythmias.

GENERAL AND SUPPORTIVE MEASURES

» Admit all seriously ill children to ICU.
» Maintain hydration.
» Cooling for hyperthermia.
» Mildly toxic patients require no specific treatment.

MEDICINE TREATMENT

- Activated charcoal.

For agitation and tachycardia:

- Diazepam, IV/oral, 0.1–0.2 mg/kg.
  - o Maximum dose of 10 mg.

For severe hypertension:
See section 4.11.1: Hypertension, acute severe.

For seizures:
See section See section 13.5: Status epilepticus (convulsive).

REFERRAL

» Status epilepticus requiring ICU.
» Hypertensive crisis.

18.2 ENVENOMATION

18.2.1 SCORPION STINGS

DESCRIPTION

Some scorpion species can cause serious systemic toxicity.
Thick-tailed scorpions with small pincers are extremely toxic resulting in both local and systemic features. Thin-tailed scorpions with large pincers are much less toxic and likely only to result in local symptoms.

DIAGNOSTIC CRITERIA

» Pain and paraesthesia occur immediately after envenomation.
» Autonomic and m otor findings may differentiate scorpion bites fro m other causes of pain.
» The pain can be exquisitely accentuated by tapping on the affected region, i.e. “tap test”.
» In severe cases cranial nerve dysfunction, blurred vision, pharyngeal muscle inco-ordination, drooling and respiratory compromise can occur.

» Excessive motor activity may present as restlessness, or uncontrollable jerking of extremities.
» Other serious effects include cardiac dysfunction, pulmonary oedema, pancreatitis, bleeding disorders and skin necrosis.
» Nausea, vomiting tachycardia and severe agitation can also occur.

GENERAL AND SUPPORTIVE MEASURES

» General supportive care.
» Monitor airway, breathing and circulation.

MEDICINE TREATMENT

For muscle cramps:

- Calcium gluconate 10%, IV, 0.5 mL/kg by slow intravenous injection.
  - o Give 0.5–1 mL/minute.
  - o Monitor ECG.

For pain:

- Paracetamol, oral, 15 mg/kg/dose, 6 hourly as required.

Very painful scorpion stings

- Lidocaine (lignocaine) 2%, 2 mL injected around the bite as a local anaesthetic.

If not immunised in the past 5 years:

- Tetanus toxoid, IM, 0.5 mL.

Complete course in previously unvaccinated patients.

Antivenom therapy

Antivenom therapy is recommended only in cases with systemic signs and is rarely required:

- Scorpion antivenom, slow IV, 10 mL administered over 3–5 minutes.

REFERRAL

» Severe cases requiring intensive care.

18.2.2 SNAKEBITE

T63.0

DESCRIPTION

The effects of snakebites may be cytotoxic, neurotoxic and/or haemotoxic. The overall effect is determined by the predominant toxin in the snake venom.

In the majority of cases, the species of snake is unknown. The patients can be divided into:

» no evidence of bite, no envenomation,
» evidence of bi te, minor envenomation, i.e. f ang marks, minimal pain, minimal swelling and no systemic signs,
» evidence of serious envenomation.

DIAGNOSTIC CRITERIA

Cytotoxic venom

» Puff adder, spitting cobra, gaboon adder.
» Venom causes severe local damage to t issues and vascular endothelium.
» Severe swelling and local necrosis occurs.

Neurotoxic venom

» Mamba, non- spitting cobra, rinkhals, berg adder.
» Venom causes a paresis and paralysis of skeletal muscles.
» Paralysis of respiratory muscles with respiratory failure may occur.
» Preceded by severe pain and paraesthesias.
» Ophthalmoplegia occurs when ocular muscles become paralysed.
» Speech and swallowing may be affected.
» Signs and symptoms start within 15–30 minutes.

Haemotoxic venom

» Boomslang, vine snake.
» Venom may cause:
  > haemolysis of red blood cells,
  > anaemia,
  > consumptive coagulopathy,
  > bruises,
  > ecchymosis,
  > epistaxis,
  > haemoptysis
  > haematuria.

GENERAL AND SUPPORTIVE MEASURES

» Patients with no evidence of bite and patients with evidence of bite but only minor envenomation should be admitted for observation. No anti-venom is indicated.
» Do not suck or cut the wound.
» Do not apply tourniquet.
» Where serious envenomation is suspected: **immediate treatment includes**
  > minimising movement of affected limb,
  > emergency treatment by bandaging affected limb with crepe bandage without compromising blood supply,
  > rapid transportation to a facility with available antivenom is the most important principle of pre-hospital care,
  > optimal therapy consisting of placing the patient at rest with the affected body part raised to the level of the heart,
  > stabilising circulation and blood pressure.

» For cytotoxic envenomation, surgical intervention, i.e. decompression surgery for established compartment syndrome and debridement of necrotic tissue should only be done when absolutely necessary and as conservatively as possible.
» For neurotoxic envenomation, ventilatory and cardiovascular support may be needed in an Intensive Care Unit.

MEDICINE TREATMENT

All patients not immunized within the past 5 years:

- Tetanus toxoid adsorbed vaccine, IM, 0.5 mL.

If children with penetrating wound and who are not completely immunised:

- Tetanus immunoglobulin, IM.
  - o If < 5 years of age: 75 IU.
  - o If 5–10 years of age: 125 IU.
  - o If > 10 years of age: 250 IU.

Cleanse wound:

- Chlorhexidine 0.05% solution in water.

Antivenom therapy

Indications:

» Consider antivenom in children who are persistently and severely affected even after the first day.
» Painful swelling of the whole hand/foot within 1 hour, spreading to elbow/knee in 3–6 hours.
» Swelling of head, neck or chest.
» Significant envenomation e.g. overt neurological signs or bite in close proximity to airway structures.
» Platelet count less than 100 x 10^9/L.
» Fibrinogen less than 100 mg/dL.
» Any confirmed black mamba or gaboon adder bite.

The dose of antivenom is the same for adult and children.

CAUTION
Never administer antivenom without being fully prepared to manage acute anaphylaxis.

Give a test dose of antivenom. If there is no untoward reaction, give the full dose. If a reaction develops following a test dose, give pre-treatment with epinephrine (adrenaline).

CAUTION

Polyvalent antivenom is only effective for the following snake bites:

» Cape cobra
» Mamba
» Puff adder
» Gaboon adder
» Rinkhals
» Spitting cobra

Boomslang requires specific antivenom.

Antivenoms are available from the South African Vaccine Producers (SAVP).
SAVP emergency number: 083 6520105.
Snakebite antivenoms may be available from specific hospitals in each province.

For cobras, mambas, rinkhals, puff adders and Gaboon viper:

- Polyvalent snake antivenom, IV.
  - o Dilute 10 mL in sodium chloride 0.9% 50 mL.
  - o Administer slowly over 15 minutes.
  - o If no reaction occurs, 60–120 mL antivenom diluted in sodium chloride 0.9%, 200 mL administered slowly over 30 minutes.

For boomslang bites:

- Boomslang antivenom, slow IV, 10 mL administered over 3–5 minutes.

OR

- Boomslang antivenom, IV infusion, 10–20 mL diluted in sodium chloride 0.9% or dextrose 5%, 50–100 mL administered over 5–10 minutes.
  - o After administration, observe patient.

Correct anaemia and bleeding tendency.

REFERRAL

» Snakebite with neurotoxic or haemotoxic manifestations may need intensive care.

18.2.3 SPIDER BITES (WIDOW SPIDERS)

T63.3

DESCRIPTION

The vast majority of spiders are no t harmful to humans. Widow spiders (*Lactrodectus*) are found in dark confined areas and the female can produce a potent venom that acts through a calcium mediated mechanism leading to the release of acetylcholine and noradrenaline from nerve terminals.

DIAGNOSTIC CRITERIA

» Bites are felt immediately as a pinprick sensation, followed by increasing local pain that may spread to include the entire extremity.
» Typical target lesions, i.e. erythematous ring surrounding a pale center.
» Cramp like spasms in large muscle groups, abdominal pain or rigidity, progressing to generalised pain involving the trunk and abdomen have been described.

GENERAL AND SUPPORTIVE MEASURES

» Supportive care of airway, breathing and circulation.

MEDICINE TREATMENT

For pain and muscle cramps:

- Calcium gluconate 10%, IV, 0.5 mL/kg by slow intravenous injection.
  - o Give 0.5–1 mL/minute.
  - o Monitor ECG and respiration.

For severe envenomation to resolve symptoms and shorten duration of illness:

- Spider antivenom, IV infusion, 5–10 mL diluted in sodium chloride 0.9% or dextrose 5%, 50–100 mL administered over 5–10 minutes.

18.2.3.1 SPIDER BITES, NECROTIC ARACHNIDISM

DESCRIPTION

Loxosceles spiders can produce local necrotic skin lesions that are mediated by enzymes.

DIAGNOSTIC CRITERIA

» Bites are initially painless.
» Skin lesions can vary from mildly erythematous lesions to severe local reaction, i.e. blistering, bluish discolouration progressing to frank necrosis.
» Systemic effects include nausea, vomiting, fever, chills, arthralgia, haemolysis, thrombocytopaenia, haemoglobinuria and renal failure.

GENERAL AND SUPPORTIVE MEASURES

» Supportive care.
» Surgical debridement once the clear margins around the necrotic lesions are established.

MEDICINE TREATMENT

For pain:

- Paracetamol, oral, 15 mg/kg/dose, 6 hourly as required.

- Morphine, IV, 0.1 mg/kg/dose 4 hourly.
    - o Monitor for respiratory depression.

Antibiotic therapy for septic lesions.

CHAPTER 19

PREMATURITY AND NEONATAL CONDITIONS

19.1 APNOEA, NEONATAL

P28.3

DESCRIPTION

A neonate presenting with episodes of cessation of breathing.
Apnoea episodes in a previously asymptomatic well neonate may be the first indication of a serious underlying disease.
Apnoea episodes in an already unwell neonate indicate deterioration in the condition of the neonate.

DIAGNOSTIC CRITERIA

» Cessation of respiration for longer than 20 seconds, with/without cyanosis, pallor or bradycardia.
» Cessation of respiration for less than 20 seconds with cyanosis, pallor and/or bradycardia.

Central apnoea

Causes include:

» IRDS,
» prematurity,
» hypoxia/hypercarbia,
» sepsis,
» acidosis,
» meningitis,
» temperature disturbances,
» hypotension,
» pneumonia,
» intraventricular haemorrhage,
» patent ductus arteriosus,
» hypoglycaemia,
» hypermagnesaemia,
» atypical convulsions,
» anaemia,
» rough or excessive handling, and
» medicines (sedatives, anticonvulsants, analgesics).

Obstructive apnoea

Neonates are obligatory "nose breathers". Obstruction of the nares make neonates prone to apnoea.
Causes of obstructive apnoea include:

» choanal atresia,
» micrognathia,
» gastro-oesophageal reflux,
» macroglossia,
» secretions (milk, meconium, blood, mucus) lodged in the upper airway, and
» neck flexion or extension.

Reflex apnoea or vagally mediated apnoea

Is due to:

- » endotracheal intubation,
- » gastro-oesophageal reflux,
- » suction of the pharynx or stomach.
- » passage of a nasogastric tube,
- » overfeeding, and

Mixed apnoea

Apnoea caused by a combination of the above causes.

GENERAL AND SUPPORTIVE MEASURES

For all forms of neonatal apnoea:

- » Identify and treat the underlying cause.
- » Frequent gentle physical stimulation e.g. rubbing of soles of feet.
- » Nurse premature neonates in the prone position.
- » Maintain ambient temperature at the lower range of neutral thermal environment.
- » Maintain axillary temperature or anterior abdominal wall temperature at 36.2–36.8°C.
- » Maintain haematocrit at 40%.
- » Maintain nasal CPAP of 4 cm water. (Nasal CPAP – not for central apnoea except for apnoea of prematurity.)
- » Monitor vital signs and parameters relating to the underlying cause.

MEDICINE TREATMENT

To maintain oxygen/haemoglobin saturation of 88–92% (for very low birth weight 88–90%) or an oxygen tension in the blood at 60–80 mmHg:

- Oxygen via nasal cannula, headbox, or mask.

Only for apnoea of prematurity (not term infants):

- Caffeine base, (anhydrous), oral.
  - o Loading dose: 10–12.5 mg/kg.
  - o Maintenance dose: 2.5–5 mg/kg/24 hours. Start maintenance dose 24 hours after the loading dose.
    (Caffeine citrate 20mg = caffeine base 10 mg)

OR

- Aminophyline, IV/oral.
  - o Loading dose: 8 mg/kg. (If IV infusion, administer over 30 minutes).
  - o Maintenance dose: 1.5–3 mg/kg/dose 8 hourly. Start maintenance dose 8 hours after loading dose

Maintain aminophylline blood levels at 10–12 mcg/mL.
If neonate responds favourably to caffeine/aminophyline continue until neonate is apnoea free for 7 days.

If central apnoea is unresponsive to therapeutic doses of caffeine or aminophyline:

- Doxapram, IV, 2 mg/kg/hour by continuous infusion.

REFERRAL

» Recurrent life-threatening episodes of apnoea, not responding to adequate treatment and requiring ventilation.

19.2 CYANOTIC HEART DISEASE IN THE NEWBORN

Q24.9

DESCRIPTION

Blue or grey discoloration of skin and tongue in room air, with an oxygen saturation of less than 85% in the presence of a cardiac lesion.

Note:

Strongly suspect cyanotic cardiac disease if centrally cyanosed, not in re spiratory distress and normotensive.

DIAGNOSTIC CRITERIA

» Rule out non-cardiac causes of central cyanosis:
  > Respiratory conditions, e.g. hyaline membrane disease, pneumonia and pneumothorax. Signs of re spiratory distress usually improve with oxygen administration. Chest X-ray may be helpful.
  > Central nervous system involvement, e.g. sed ation and asphyxia, which usually improves with oxygen administration.
  > $PaCO_2$ may be increa sed in cyanosis due to resp iratory and centra l nervous system causes.
  > Methaemoglobinaemia.

» Confirm cardiac cause:
  > Little or no improvement with oxygen administration. See hyperoxia test.
  > Tachypnoea, but usually no retraction.
  > Heart murmur (may be absent).

» Chest X-ray may show cardiomegaly or abnor mal cardiac silhouette and/or reduced pulmonary blood flow.
  > Confirm diagnosis with echocardiography.

» Hyperoxia Test (Nitrogen wash out test)
  > Administer 100% oxygen via a nasal cannula for 10 minutes.
  Unnecessary if saturation is under 85% in a head box or nasal cannulae delivering 100% oxygen.
  > Obtain arterial blood from the right radial artery (preductal flow).

PaO_2 mmHg	Interpretation
< 100	Most likely to be a cyanotic heart lesion, persistent fetal circulation or severe lung disease.<br>$PaCO_2$ will be increased with severe lung disease.
≥ 100–200	Unlikely to be cyanotic heart lesion.
≥ 200	Excludes cyanotic heart lesion.

GENERAL AND SUPPORTIVE MEASURES

» Nurse in neutral thermal environment.
» Monitor and maintain within physiological range for age:
  > heart rate,
  > respiration,
  > blood pressure,
  > body temperature,
  > electrolytes.
  > calcium, magnesium,
  > blood glucose,
  > blood gases,
  > acid-base status, and
» Provide adequate hydration and nutrition.

MEDICINE TREATMENT

To keep ductus arteriosus open if a duct dependent cyanotic heart lesion is suspected:

■ Prostaglandin therapy, i.e.:

- Alprostadil, IV, 0.05–0.1 mcg/kg/minute, initial dose.
  - o Maintenance dose : 0.01–0.1 mcg/kg/minute.

OR

- Dinoprostone, via naso/orogastric tube.
  - o For babies < 2.5 kg: 0.125 mg 1–2 hourly ($^1/_4$ tablet suspended in 2 mL sterile water), **or** 50 mcg/kg/dose 1–2 hourly.
  - o For babies > 2.5 kg: 0.25 mg hourly (½ tablet suspended in 2 mL water).

Continue with prostaglandin therapy until corrective or palliative surgery can be done or until patency of the duct is not deemed essential for survival of the infant.
Babies on prostaglandin therapy: inspiratory oxygen not more than 40 %.
An oxygen saturation of haemoglobin > 75% is acceptable.

**Serious side effects of prostaglandins to be aware of may include: Apnoea, fever, diarrhoea, hypotension and seizures**

If pH ≤ 7.2, correct metabolic acidosis:
» Sodium bicarbonate 4.2 %, IV.
  HCO_3 needed (mmol) = base excess x 0.3 x body mass (kg).
  2 mL sodium bicarbonate 4.2% = 1 mmol HCO_3.

SURGICAL TREATMENT

» Corrective or palliative surgery.

REFERRAL

» All cyanotic infants with an underlying cardiac cause for central cyanosis.

19.3 ENTEROCOLITIS, NECROTISING

P77

DESCRIPTION

Neonate presenting with the consequences of bowel wall injury or necrosis.

Risk factors include:

- prematurity,
- sepsis,
- early formula feedings,
- patent ductus arteriosus, and
- hypotension/shock,
- high feeding volumes,
- perinatal asphyxia (hypoxia),
- polycythaemia.

DIAGNOSTIC CRITERIA

- Early signs are often non-specific, i.e.:
  - feeding intolerance,
  - gastric aspirates,
  - vomiting,
  - body temperature instability,
  - apnoea and lethargy.
- Non-specific signs may progress to more specific signs including:
  - abdominal distention with ileus,
  - bloody stools,
  - peritonitis,
  - red-purple discolouration of the abdominal wall with abdominal wall cellulitis, and
  - bowel perforation.
- X-ray of abdomen may show:
  - distended loops of intestines,
  - bowel-wall thickening (oedema),
  - pneumatosis intestinalis,
  - hepatic portal venous gas, and
  - free intraperitoneal air due to perforation.
- Blood samples for culture and sensitivity testing before starting antibiotic therapy.

GENERAL AND SUPPORTIVE MEASURES

- Admit to neonatal high-care unit or intensive care unit.
- Nurse in neutral thermal environment.
- Insert oro/nasogastric tube and apply free drainage.
  - Suspected cases should be nil per mouth for 72 hours.
  - Confirmed cases should be nil per mouth for at least 7 days.
- Provide adequate parenteral nutrition (hyperalimentation) as soon as diagnosis is confirmed.
- Provide cardiovascular and ventilatory support, if necessary.

MEDICINE TREATMENT

Depending on age, weight and hydration status:

- Neonatal maintenance solution, IV.
  Add volume of gastric aspirates to daily maintenance fluid volume.

If co-agulopathy or septic shock:

- Plasma, IV, 20 mL/kg over 2 hours.

If haematocrit < 40%:

- Packed red cells, IV, 10 mL/kg.

Until blood pressure is stabilised:

- Dopamine, IV, 5–15 mcg/kg/minute.

Empiric antibiotic therapy

- Ampicillin, IV, 50 mg/kg/dose for 10 days.
  - If age < 7 days: 50 mg/kg 12 hourly.
  - If 7 days – 3 weeks of age: 50 mg/kg 8 hourly.
  - If > 3 weeks of age: 50 mg/kg 6 hourly.

PLUS

- Gentamicin, IV, 6 mg/kg once daily for 10 days.

PLUS

- Metronidazole, IV, for 7 days.
  - Loading dose: 15 mg/kg over 60 minutes.
  - Post natal age < 4 weeks: 7.5 mg/kg/dose 12 hourly.
  - Postnatal age ≥ 4 weeks: 7.5 mg/kg/dose 8 hourly.

Reassess choice of antibiotics when the culture and sensitivity results become available.

SURGICAL TREATMENT

Surgical intervention is required when there is progressive deterioration of the clinical condition despite maximal medical support and/or bowel necrosis with/without bowel perforation.

Prior to transport to a tertiary hospital for definitive surgery, insert/place a peritoneal drain in babies presenting with severe abdominal distension, due to free air and/or fluid in the peritoneal cavity, compromising respiration and/or blood pressure.
Perform the procedure in a theatre, intensive care or high care unit where facilities for monitoring vital signs, resuscitation, ventilation and temperature control of the environment are available.
Obtain consent to perform the surgical procedure.

Method of inserting/placing a peritoneal drain

- » Procedure is sterile; the doctor should be gowned and gloved.
- » Clean and drape the abdomen.
- » Administer an appropriate analgesic (e.g. ketamine, IV) immediately before the start of the procedure.
- » Identify a site in either one of the fossae iliaca, make sure it is lateral to the inferior epigastric artery.
- » At the intended surgical incision site, inject:
- • Lidocaine (lignocaine) 1%, SC, 0.5 mL.
- » Make a small skin incision over the "bubble" of lidocaine (no. 11 blade).
- » Use a mosquito forceps or clamp to dissect down to the peritoneum, pierce the latter with a gentle stab using the closed forceps and slightly stretch the peritoneal puncture site with the forceps.
- » Note what drains from the peritoneal cavity and send a sample for microscopy and culture.
- » Insert a pencil drain of ± 5 mm width with the mosquito clamps or forceps into the peritoneal cavity through the peritoneal stab wound. About 1.5–2 cm of the pencil drain should be inside the peritoneal cavity.
- » Fix the drain to the skin with a size 4 – 0 stitch (e.g. PDS).
- » Cover the drain with a gauze pad or urine collecting bag.

REFERRAL

- » All confirmed cases for specialist care.
- » Deterioration of clinical condition, despite adequate treatment.
- » Signs and symptoms of intestinal perforation and peritonitis requiring surgical intervention.
- » Recurrent apnoea episodes and/or signs of respiratory failure, requiring respiratory support.

19.4 HAEMORRHAGIC DISEASE OF THE NEWBORN

P53

DESCRIPTION

This is due to a deficiency of vitamin K-dependent clotting factors II, VII, IX and X. All newborns who did not receive vitamin K_1 at birth, especially premature babies and breastfed babies, are at risk.

Spontaneous bleeding may be from any site but is usually gastro-intestinal, producing haematemesis or melaena. Bleeding from the umbilical stump, epistaxis and a cephalohaematoma or subgaleal haemorrhage are also relatively common.

Complications may include anaemia, hypovolaemic shock and intracranial haemorrhage with neurological damage.

There are three forms of the disorder.

Early form: presents within 24 hours of birth in newborns of mothers on treatment with anticonvulsants, e.g. phenytoin and phenobarbitone, or oral anticoagulants.

Classical form: presents during the first week of life usually on the second to seventh day.
Late form: presents during the first to fourth month of life usually with intracranial haemorrhage in exclusively breastfed babies who did not receive vitamin K prophylaxis at birth.

DIAGNOSTIC CRITERIA
Special investigations
» Prolonged prothrombin time (PT).
» Normal partial prothrombin time (PTT).
» Increased international normalised ratio (INR) with a normal platelet count.
» Normal fibrinogen levels.
» Normal thrombin time.

Note:
Exclude other causes of bleeding in the neonate.
Exclude swallowed blood of mother during delivery in babies with melaena. (Apt test or haemoglobin electrophoresis).

GENERAL AND SUPPORTIVE MEASURES
» Nurse in neutral thermal environment.
» Provide adequate nutrition.
» Monitor:
  > blood pressure,
  > heart rate,
  > respiratory rate,
  > body temperature,
  > coagulation parameters.
  > hydration,
  > SaO_2,
  > haematocrit,
  > blood glucose, and

MEDICINE TREATMENT
- Oxygen, if needed.

- Fresh frozen plasma or lyophilised plasma, IV, 20 mL/kg over one hour.

If anaemic (haematocrit < 40% or Hb < 13 g/dL):
- Packed red cells, IV, 10 mL/kg over 1 hour.
  o May be repeated if necessary.

- Vitamin K_1, IM, 1 mg as a single dose.

Prophylaxis
- Vitamin K_1, IM, single dose at birth.
  Full term newborns: 1 mg
  Preterm newborns: 0.5 mg

Prophylaxis with oral vitamin K formulation is not recommended.

REFERRAL

- » Deterioration of clinical condition despite adequate treatment.
- » Suspected intracranial haemorrhage.

19.5 HEART FAILURE IN NEONATES

P29.0

DESCRIPTION

Clinical syndrome reflecting the inability of the myocardium to meet the oxygen and nutritional or metabolic requirements of the body. Heart failure may be acute or chronic.

The main causes of heart failure are:

- » Congenital heart abnormalities:
  - > Left-sided outflow obstruction, e.g. interrupted aortic arch, co-arctation of the aorta and aortic valve stenosis.
  - > Left to right shunts, VSD and PDA.
  - > Hypoplastic left heart.
  - > Complex congenital heart lesions.
- » Acquired conditions:
  - > fluid overload,
  - > hypoglycaemia,
  - > acidosis,
  - > dysrhythmias,
  - > pneumopericardium,
  - > hypertension.
  - > sepsis,
  - > hypoxia,
  - > severe anaemia,
  - > cardiomyopathy,
  - > hyperthyroidism,

DIAGNOSTIC CRITERIA

Diagnosis relies on history, physical examination and a chest X-ray.

Clinical

- » Acute heart failure may present with shock, i.e. cardiogenic shock.
- » Heart failure is usually associated with fluid retention and congestion.
- » History of recent onset of:
  - > poor feeding,
  - > tachypnoea (> 60 breaths/minute),
  - > sweating, and
  - > poor or excessive weight gain in excess of 30 g/24hours.
- » Physical findings:
  - > tachycardia (> 180 beats/minute),
  - > gallop rhythm (with/without a cardiac murmur),
  - > cardiomegaly,
  - > features of cardiogenic shock, i.e. cold wet skin, weak pulses, hypotension,
  - > reduced urinary output,
  - > pulmonary venous congestion and fluid retention,

  - systemic venous congestion,
  - hepatomegaly, and
  - signs and symptoms of underlying condition/disease.
- Always check the femoral pulses.

Special Investigations
- Radiology: cardiomegaly is almost always present, cardiothoracic ratio > 60%.
- Electrocardiogram may show evidence of hypertrophy of one or more heart chambers and/or dysrhythmias.
- Sonar or ultrasound may show a reduced ejection fraction or shortening fraction of left ventricle.

GENERAL AND SUPPORTIVE MEASURES
- Nurse in a neutral thermal environment.
- Restrict fluids but ensure adequate nutrition.
  - Administer 75% of daily requirements.
  - Use breast milk or low-salt milk formulae.
  - Tube feeding.
- Treat the underlying condition, e.g. sepsis and cardiac tamponade.

MEDICINE TREATMENT
First treat shock, if present.

To prevent hypoxia:
- Oxygen via face mask, nasal cannula or head box.

Combination medicine therapy is usually indicated,

Afterload reduction: ACE inhibitor or vasodilator
Monitor blood potassium levels and stop potassium supplements while patient is on an ACE inhibitor.
ACE inhibitors are contraindicated in renal failure, bilateral renal artery stenosis or a single functioning kidney.

Consider ACE inhibitors in persistent heart failure where left sided outflow obstruction has been excluded, other measures have failed and only after consultation with a paediatrician or paediatric cardiologist.

- Captopril, oral, 0.01–0.05 mg/kg/dose, 8–12 hourly, initially.
  - Adjust dose and interval based on response.
  - Administer 1 hour before feeding.
  - Continue as long as needed to control the heart failure

Diuretics
Continue diuretic therapy as long as needed to control heart failure.
Monitor blood potassium levels.

Potassium supplements may be necessary if furosemide is used without spironolactone.
Hypokalaemia and hypochloraemic alkalosis may increase digitalis toxicity.

- Furosemide, IV/oral, 1–3 mg/kg/24 hours as a single daily dose, or in 4 divided oral doses.

WITH/ WITHOUT

- Spironolactone, oral, 1–3 mg/kg/dose, once daily.

Digoxin
Consider in consultation with paediatrician or cardiologist
Monitor digoxin blood levels and ECG.

Digoxin is contraindicated in bradycardia, heart block, cardiac tamponade or hypertrophic cardiomyopathy.

- Digoxin, oral, 0.01 mg/kg/dose 8 hourly for 3 doses, then:
  - Maintenance: oral, 0.005 mg/kg/dose for as long as needed to control heart failure:
    For infants < 37 weeks gestational age: 24 hourly.
    For infants ≥ 37 weeks gestational age: 12 hourly.

When oral route is contraindicated:

- Digoxin, IV, 75% of oral dose.

Inotropic support
May help to stabilise patients with severe myocardial dysfunction, hypotension or low cardiac output.
May be lifesaving in severe myocarditis or cardiogenic shock.

- Dobutamine, IV infusion, 2.5–15 mcg/kg/minute.
  - Continue until myocardial function and blood pressure improve.
  - Ensure normovolaemia.
  - Monitor blood pressure.

Acute left-heart failure: acute pulmonary oedema or pulmonary venous congestion

- Oxygen 100%, via nasal cannula.

- Furosemide, IV, 1–3 mg/kg, immediately.

For patients not responding to furosemide:

- Morphine, IV, 0.1 mg/kg.

For patients not already on digoxin treatment:

- Digoxin, as above.

- Inotropic support, as above.
- Afterload reduction, as above.

To raise the alveolar pressure above pulmonary capillary pressure, intubate with intermittent positive ventilation.

SURGICAL TREATMENT
Palliative or corrective surgery for certain congenital heart lesions.

REFERRAL

» Deterioration despite adequate treatment.
» For determination of the underlying cause, initiation of treatment and stabilisation.

19.6 HYPOCALCAEMIA, NEONATAL

P71.1

DESCRIPTION

Acute symptomatic hypocalcaemia may present within the first 72 h ours of bir th (early hypocalcaemia) or after 72 hours of birth (late hypocalcaemia) with apnoea, irritability, seizures, jitteriness or prolonged QTc interval on ECG.

Causes of early hypocalcaemia include:
» Prematurity.
» Respiratory distress syndrome.
» Asphyxia/hypoxia.
» Neonate of diabetic mother.
» Sepsis.

Causes of late hypocalcaemia include:
» Maternal hyperparathyroidism.
» Congenital hypoparathyroidism.
» Renal failure.
» Hypomagnesaemia.
» High phosphate feeds.
» Vitamin D deficiency.

DIAGNOSTIC CRITERIA

» Total serum calcium < 1.8 mmol/L, or
» Ionised calcium < 0.7 mmol/L.

MEDICINE TREATMENT

Symptomatic hypocalcaemia:

- Calcium gluconate 10%, IV, 100–200 mg/kg/dose 6–8 hourly.

1 mL of calcium gluconate 10%	= 100 mg calcium gluconate
	= 10 mg elemental calcium
	= 0.23 mmol calcium

Correct hypomagnesaemia before administering 10% calcium gluconate.

- Magnesium sulphate 50%, IV, 0.25 mL/kg.
  - o Monitor levels until deficits are reduced.

Do not administer ceftriaxone, within 48 hours of administering calcium.

Exchange transfusion

- Calcium gluconate 10%, IV infusion, administered over 10 minutes.
  - o 100mg for every 100 mL citrated blood exchanged.

Acute hypocalcaemia with seizures

- Calcium gluconate 10%, IV infusion, 100–200 mg/kg, administered over 10 minutes. Repeat in 15 minutes if necessary.
  - o Dilute 1:1 with dextrose 5% or sodium chloride 0.9%.
  - o Do not use calcium chloride.

  Note: Rapid infusion causes bradycardia/dysrhythmias. Electrocardiographic monitoring is advised. Monitor the heart rate.

CAUTION
Do not mix calcium gluconate with bicarbonate or fluids containing phosphate as precipitation may occur.
Extravasation of calcium can cause tissue necrosis.
Do not give intra-arterially or via umbilical venous catheters placed near the heart or inside the liver.

REFERRAL

» Persisting or recurrent unexplained hypocalcaemia.

19.7 HYPOGLYCAEMIA, NEONATAL

P70.4

DESCRIPTION

Neonate presenting with a whole blood glucose below 2.6 mmol/L.

Risk factors include:

- prematurity,
- small for gestational age,
- neonate of diabetic mother,
- sepsis,
- hypothermia/hyperthermia,
- birth asphyxia,
- respiratory distress,
- rhesus iso-immunisation,
- hyperinsulinism,
- post maturity,
- feeding difficulties,
- polycythaemia, and
- hereditary defects in carbohydrate or amino acid metabolism.

DIAGNOSTIC CRITERIA

Clinical

Asymptomatic: Hypoglycaemia detected when screening neonates at risk.
Symptomatic:

- lethargy,
- hypotonia,
- apnoea,
- jitteriness,
- irritability,
- coma.
- poor feeding,
- respiratory distress,
- cardiac failure,
- convulsions,
- metabolic acidosis, and

Investigations

- Whole blood glucose (heel prick) < 2.6 mmol/L.

Monitor the blood glucose of all neonates who are at risk of hypoglycaemia regularly, at least 2 hourly, to prevent the development of hypoglycaemia.

GENERAL AND SUPPORTIVE MEASURES

- Determine and treat the underlying cause.
- Enteral feeding, oral or via oro/nasogastric tube, after exclusion of vomiting, ileus or obstruction.

MEDICINE TREATMENT

- Dextrose 10%, bolus IV, 2.5 mL/kg (250 mg/kg).
  Dextrose 10% = 10 g dextrose in 100 mL.
  Do not repeat dextrose bolus.

To raise heel prick blood glucose to a level of 2.6 mmol/L or more, follow with:

- Dextrose 10%, continuous IV infusion, 6–12 mg/kg/minute or more.

$$\text{Dextrose dose (mg/kg/min)} = \frac{(\%\ \text{dextrose solution x rate in mL/hour})}{(\text{weight x 6})}$$

If heel prick blood glucose remains below 2.6 mmol/L:

- Dextrose 15%, IV, 15 mg/kg/minute or more
  Dextrose 15% = 15 g dextrose in 100 mL.

If heel prick blood glucose is above 2.6 mmol/L after IV infusion has been started continue infusion at maintenance rate.

Monitor blood glucose at least 2 hourly until blood glucose level stabilises at 2.6 mmol/L or above. To avoid rebound hypoglycaemia, reduce IV dextrose infusion gradually.

Before the IV i nfusion is finally discontinued, the ne onate should receive all milk feeds orally or via nasogastric tube. If enteral feeds are not tolerated TPN should be given.

Suspect other serious underlying metabolic or biochemical abnormality if the neonate requires > 12 mg/kg/minute of dextrose to maintain a heel prick whole blood glucose > 2.6 mmol/L.
Use a central venous line for high concentrations of dextrose.

Prior to referral give the following, if available:
- Glucagon, IM/IV/SC, 0.2 mg/kg single dose.

REFERRAL
» Hypoglycaemia not responding to adequate treatment.
» Recurrent or persistent hypoglycaemia

See also section 7.6 Hypoglycaemia in children.

19.8 HYPOXIA/ISCHAEMIA OF THE NEWBORN (PERINATAL HYPOXIA/HYPOXIC-ISCHAEMIC ENCEPHALOPATHY)
P21.9

DESCRIPTION
Ischaemia and decreased oxygen delivery to the fetus/baby during the prepartum, intrapartum or immediate postpartum period, with hypoxic-ischaemic damage to the central nervous system and to other body systems.

Complications include:
» Cardiovascular: heart rate and rhythm disturbances, heart failure and hypotension.
» Pulmonary: respiratory distress/respiratory failure, pulmonary hypertension and pulmonary haemorrhage.
» Renal: renal failure, acute tubular/cortical necrosis and urinary retention.
» Gastrointestinal tract: ileus and necrotising enterocolitis.
» Central nervous system: increased intracranial pressure, cerebral oedema, encephalopathy, seizures, inappropriate antidiuretic hormone (ADH) secretion, hypotonia and apnoea.
» Metabolic: hypoglycaemia, hyperglycaemia, hypocalcaemia, hypomagnesaemia and metabolic acidosis.
» Body temperature: abnormal.
» Other: disseminated intravascular coagulation.

DIAGNOSTIC CRITERIA

» History of fetal distress and/or meconium stained amniotic fluid.
» Apgar scores:
  > one-minute Apgar score ≤ 3,
  > five-minute Apgar score of ≤ 6,
» Arterial blood lactate > 5 mmol/L.
» Severe mixed acidosis:
  > pH < 7.2,
  > base excess > –10,
  > $PaCO_2$ > 55 mmHg.
» Haematuria.
» TroponinT increased.

Stages of hypoxic-ischaemic encelphalopathy (HIE)

**Stage**	**Stage 1 mild**	**Stage 2 moderate**	**Stage 3 severe**
Prognosis	Good	Guarded<br>± 50% may have varying degree of neurological sequelae.	Poor<br>≥ 90% mortality with major neurological sequelae in survivors
Level of consciousness	» Hyperalert<br>» Irritable	» Lethargic<br>» Obtunded	» Stuporous<br>» Comatose
Neuromuscular control	» uninhibited<br>» over-reactive	» diminished<br>» spontaneous movement	» diminished/absent<br>» spontaneous movement
Muscle tone	» normal	» mild hypotonia	» flaccid
Posture	» mild distal flexion	» strong distal flexion	» intermittent decerebration
Tendon reflexes	» overactive	» overactive	» decreased/absent
Complex reflexes			
Suck	» weak	» weak/absent	» absent
Moro	» strong	» weak	» absent
Autonomic function	» general sympathetic	» general parasympathetic	» both systems depressed
Pupils	» mydriasis	» miosis	» mid-position, often unequal<br>» poor light reflex
Respirations	» spontaneous	» spontaneous<br>» occasional apnoea episodes	» periodic apnoea episodes
Heart rate	» tachycardia	» bradycardia	» variable, usually bradycardia

Stage	Stage 1<br>mild	Stage 2<br>moderate	Stage 3<br>severe
Bronchial and salivary secretions	» sparse	» profuse	» variable
Gastrointestinal motility	» normal or decreased	» increased	» variable<br>» ileus
Seizures	» none	» common	» uncommon, but prolonged if present decerebrate

Note: Newborns with stage 3 HIE should not be ventilated**.**

GENERAL AND SUPPORTIVE MEASURES

» Resuscitate.
» Avoid hyperthermia.
» Admit to neonatal high care or intensive care facility, if available.
» Whole body or head cooling should be done under supervision of a paediatrician.
  > Initiate within 6 hours of birth to maintain rectal (core) temperature at 33–34ºC for 72 hours.
  > Slowly rewarm at a rate of 0.5ºC/hour until axillary or skin temperature is at 36.5–36.8ºC.
  > Mild HIE: ambient temperature at lower range of neutral thermal environment.
  > Infants ≥ 36 weeks gestation with moderate HIE (stage 2): whole body or head cooling.
» Ventilatory support if PaO_2 < 60 mmHg and/or $PaCO_2$ > 55 mmHg in newborns with moderate HIE (stage 2).
» Maintain:
  > Blood glucose at 2.6–6 mmol/L.
  > Haematocrit at ≥ 40%.
  > Blood pressure at 70/35 mmHg in a term infant and 50/35 mmHg in a preterm infant. Mean blood pressure at least 5–10 mmHg more than the gestational age.
» IV fluids
  > Frequent assessment of fluid balance, i.e. intake and output.
  > Restrict fluids to 50–60 mL/kg in the first 24–48 hours.
  > Use dextrose water 10% or a neonatal maintenance solution potassium-free until the possibility of renal failure has been excluded.
» Maintain serum electrolytes, calcium, magnesium and acid-base status within normal physiological range.
» Nutrition:
  > No enteral feeds for at least the first 12–24 hours.
  > Enteral milk feeds only after ileus has been excluded.
  > Consider IV alimentation if enteral feeds are not possible after 24 hours.

» Monitor:
  - neurological status,
  - vital signs,
  - acid-base status,
  - blood gases,
  - SaO_2,
  - blood pressure,
  - fluid balance,
  - temperature,
  - blood glucose,
  - electrolytes,
  - calcium, magnesium,
  - renal function, and
  - brain function (aEEG), where available.

» Follow up for assessment of neurodevelopment, hearing and vision.

MEDICINE TREATMENT

To keep PaO_2 between 60 and 80 mmHg and saturation 88–92% (normal range):

- Oxygen.

Haematocrit <40%:

- Packed red cells, IV, 10mL/kg.

If infection is suspected or confirmed:

- Cefotaxime, IV, 50 mg/kg/dose.
  - o 12 hourly for first 7 post natal days and 8 hourly thereafter.
  - o If infection is excluded, antibiotics can be stopped in 72 hours.

Hypotension

- Sodium chloride 0.9%, IV, 20 mL/kg over 1 hour.

AND

- Dopamine, IV, 5–15 mcg/kg/minute.

AND/OR

- Dobutamine, IV, 5–15 mcg/kg/minute if cardiac dysfunction or failure is present.
  - o Continue with blood pressure support until blood pressure is stabilised.

Seizure Control

Administer anticonvulsants with monitoring of cardiorespiratory function.

- Phenobarbitone, IV:
  - o Loading dose: 20 mg/kg over 10 minutes.
  - o Refractory seizures: Additional 10 mg/kg up to 40 mg/kg.
  - o Maintenance: 4 mg/kg/day beginning 12–24 hours after the loading dose.

Do not use lidocaine if phenytoin was given.<br>Do not use lidocaine for seizures in newborns with congenital heart disease, dysrhythmias, acute heart failure and shock.

Admit neonates with seizures refractory to phenobarbitone to a high or intensive care unit.

Cardiorespiratory support is usually required in this category of infants.

For term normothermic neonates:

- Lidocaine (lignocaine), IV.
  - o Loading dose: 2 mg/kg over 10 minutes.
  - o Follow with a continuous infusion of:
    6 mg/kg/hour for 6 hours, then
    4 mg/kg/hour for 12 hours, followed by
    2 mg/kg/hour for 12 hours.
  - o If seizures are well controlled, taper slowly over 12 hours.

For preterm neonates:
A safe dose of lidocaine in preterm neonates has not been established but the following dosing schedule has been used.

- Lidocaine (lignocaine), IV.
  - o Loading dose: 2 mg/kg over 10 minutes.
  - o Follow with a continuous infusion of 3 mg/kg/hour for 3 days.
  - o Taper dose gradually over next 2 days.

» A safe lidocaine dosing regimen for term infants undergoing hypothermia treatment for HIE has not been established.
» Clearance of lidocaine is slower in hypothermic preterm infants and neonates and there is a risk of accumulation.
» Start tapering earlier than 3 days if seizures are well controlled.
» Continuous monitoring of ECG, heart rate and blood pressure is mandatory if lidocaine is used.
» Main adverse effects of lidocaine: dysrhythmias and bradycardia.
» Life threatening dysrhythmias may indicate lidocaine toxicity. Treat with:

- Lipid emulsion 20%, IV, 1.5 mg/kg over 1 minute.
  - o Follow with a continuous infusion of 0.25 mL/kg/minute for 30 minutes. Refer urgently.

Cardiac failure
Restrict fluid.

- Furosemide, IV/oral/nasogastric tube, 1 mg/kg/24 hours as a single daily dose.
- Dobutamine IV, 5–15 mcg/kg/minute.

Hypocalcaemia
Serum total calcium < 1.8 mmol/L or ionised calcium < 0.7 mmol/L.

- Calcium gluconate 10%, slow IV, 1–2 mL/kg over 15 minutes under ECG control.

Hypomagnesaemia
Serum magnesium < 0.6 mmol/L:

- Magnesium sulphate 50%, IV, 0.2 mL/kg as a single dose.

Hypoglycaemia
Blood glucose < 2.6 mmol/L:
- Dextrose 10%, bolus IV, 2.5–5 mL/kg (250–500 mg/kg).
  Dextrose 10% = 10 g dextrose in 100 mL.
  Do not repeat dextrose bolus.

Inappropriate ADH:
Moderate fluid restriction of 50–60 mL/kg/24hours for the first 24–48 hours.
Raise head of cot by 10–15 cm.

Cerebral oedema/raised intracranial pressure
Moderate hyperventilation to lower $PaCO_2$ to 35 mmHg, if ventilation facilities are available.

REFERRAL
» Neurological assessment of survivors at 3 months.
» Moderate HIE (gestational age ≥ 36 weeks) to reach referral hospital before 6 hours post birth.
» Lidocaine toxicity.

19.9 JAUNDICE, NEONATAL
P58

DESCRIPTION
Yellow discolouration of the skin and mucous membranes due to hyperbilirubinaemia.
Bilirubin is formed mainly from haem catabolism. Jaundice develops when there is an overproduction of bil irubin, defective bilirubin metabolism and/or defective excretion of bilirubin from the body.

DIAGNOSTIC CRITERIA
Jaundice may have a physiological and a pathological component.
Physiological jaundice
» Seldom appears before 24–36 hours after birth.
» Rarely lasts more than 10 days in the full term infant and 14 days in the pre-term infant.
» Only the unconjugated bilirubin fraction is increased.
» Total peak serum bilirubin concentration is usually below 275 micromol/L in the term infant.
» Total bilirubin concentration does not rise by more than 85 micromol/L/24 hours or 17 micromol/L/hour.
» The baby thrives and shows no signs of illness or anaemia.
» Treatment is unnecessary.

Pathological jaundice

- » Appears within the first 24 hours of birth but may also appear at any other time after birth.
- » Persists for longer than 10 days in the full term infant or 14 days in the pre-term infant.
- » The unconjugated and/or conjugated fractions of bilirubin are increased.
- » The conjugated bilirubin level exceeds 10% of the total bilirubin value, or the conjugated bilirubin fraction is 30 micromol/L or more.
- » Total bilirubin concentration ris es by more than 85 micromol/L/24 hours or 17 micromol/L/hour the total serum bilirubin level is above physiological level.
- » There are signs and symptoms of illness in the baby.
- » Stools are pale in obstructive jaundice.

BREASTFEEDING ASSOCIATED JAUNDICE

Increased unconjugated bilirubin levels during the first week of li fe in br eastfed babies is due to calorie and fluid deprivation and delayed passage of stools. Improves with increased frequency of breastfeeding.

19.9.1 HYPERBILIRUBINAEMIA, UNCONJUGATED

**Excessive haemolysis**	**Defective conjugation**
» ABO incompatability	» prematurity
» rhesus disease	» infection
» enclosed haemorrhages	» hypoxia
» polycythaemia	» hypoglycaemia
» infections*	» hypothyroidism*
» spherocytosis	» breast milk jaundice*
» G6PD deficiency	

* may cause prolonged neonatal jaundice

GENERAL AND SUPPORTIVE MEASURES

- » Treat the underlying cause.
- » Monitor the infant's body temperature and maintain within thermo neutral range.
- » Maintain adequate nutrition and hydration.
- » Correct factors known to inc rease the risk of br ain damage in ba bies with jaundice e.g.:
  - > hypoxia,
  - > hypoglycaemia,
  - > acidosis,
  - > haemolysis.
  - > prematurity,
  - > hypothermia,
  - > hypoalbuminaemia, and

PHOTOTHERAPY

Guideline for initiating phototherapy:

- » Terminate phototherapy when the unconjugated bilirubin level is lower than the recommended phototherapy initiating level, and the cause of the jaundice has been determined and adequately addressed.
- » The skin colour of a baby receiving phototherapy does not reflect the degree of jaundice (bilirubin blood level) or the efficacy of the phototherapy.
- » Undress the baby and cover the eyes with gauze pad or commercially available eye covers.
- » Position the phototherapy unit (fluorescent light bulbs of 400– 500 nm wavelength) not higher than 45 cm above the baby.
- » Check spectral irradiance of the fluorescent lights after every 200–300 hours of use to ensure that they are still effective (use radiometer, if available).
- » The spectral irradiance should be above 10 microwatt/cm^2/nm of wavelength. If spectral irradiance cannot be checked regularly, replace fluorescent light bulbs after 1 000 hours of continuous use.
- » A quartz halogen light source (400–500 nm wavelength) can also be used for phototherapy.
- » Phototherapy units with diodes emitting light in the blue spectrum or fibro-optic phototherapy units can be used instead of the fluorescent/quartz halogen units.
- » A rebound increase in bilirubin may follow termination of phototherapy.
- » Monitor bilirubin levels approximately 6 hourly after phototherapy has been stopped.

Guideline for exchange transfusion (See also the graphs attached):

- » Exchange transfusion is indicated when the risk of bilirubin encephalopathy and kernicterus is significant. Referral for exchange transfusion may be needed.

MEDICINE TREATMENT

Rh incompatibility (i.e. mother Rh negative, baby Rh positive).
ABO incompatibility (i.e. mother = O, baby = A, B or AB).
As soon as the diagnosis of Rh – or ABO incompatibility is confirmed, administer:

- Gammaglobulin, IV, 500 mg/kg over 1 hour.
  - o Can be repeated after 6–8 hours.

Mothers of babies with Rh incompatibility should receive:

- Anti D immunoglobulin, IM, 100 mcg as soon as possible after birth but within 72 hours of birth.

19.9.2 HYPERBILIRUBINAEMIA, CONJUGATED

Hepatocellular disease	Bile duct obstruction
» hepatitis*<br>» total parenteral nutrition*<br>» syphilis<br>» other congenital infections<br>» galactosaemia*	» bile duct hypoplasia/atresia*<br>» choledochal cyst<br>» cystic fibrosis

* May cause prolonged neonatal jaundice.

Conjugated hyperbilirubinaemia is due to intra/extrahepatic obstruction of bile ducts (cholestasis) and usually presents in the second week of life or later.
The baby has a green-yellow skin discolouration, dark bile stained urine and pale acholic stools. Hepatomegaly is commonly present and the infant often fails to thrive.

Neonatal hepatitis, prolonged TPN and biliary atresia or hypoplasia accounts for the majority of cases of conjugated hyperbilirubinaemia.

GENERAL AND SUPPORTIVE MEASURES

- » Treat the underlying cause.
- » Dietary modifications to counteract the malabsorption of fat and fat soluble vitamins (A, D, E and K) that may occur in patients with a prolonged conjugated hyperbilirubinaemia.
- » When galactosaemia is suspected avoid lactose containing feeds, i.e. breast milk and lactose containing formula.

MEDICINE TREATMENT

- Fat soluble vitamins (A, D, E and K).

SURGICAL TREATMENT

Conditions amenable to surgery e.g. biliary artresia.
Hepatoporto-enterostomy for biliary atresia should be done before 60 days of age for optimal outcome.

REFERRAL

- » All cases of jaundice persisting more than 2 weeks with conjugated bilirubin level >20% of total bilirubin, for diagnosis and initiation of treatment.

19.10 JAUNDICE, NEONATAL, PROLONGED

DESCRIPTION

Jaundice (static or a rising bilirubin) present for more than 10 days in a term infant and 14 days in a preterm infant. The usual causes are:

- » breast milk jaundice,
- » hypothyroidism,
- » hepatitis,
- » galactosaemia, and
- » infections, e.g. UTIs.

Breast milk jaundice may be confirmed by substituting breastfeeding with formula feeds for 24–48 hours. The bilirubin level will always drop to a lower level and increase again when breastfeeding is resumed. However, the level will not rise to the original high level. Breast milk jaundice is an unconjugated hyperbilirubinaemia and the infant is always well and thriving.

Abnormal thyroid function, increased TSH and decreased T_3 and T_4, indicates hypothyroidism. Unconjugated bilirubin fraction is raised and the infant may have clinical signs of hypothyroidism e.g.:

- » lethargy,
- » feeding difficulties,
- » poor cry,
- » nasal obstruction,
- » bradycardia.
- » constipation,
- » hypotonia,
- » umbilical hernia,
- » hypothermia, and

Infants with galactosaemia usually present with:

- » a conjugated hyperbilirubinaemia,
- » refusal to feed,
- » failure to thrive,
- » vomiting,
- » hepatomegaly,
- » Hypoglycaemia, and
- » encephalopathy and later cataracts.

DIAGNOSTIC CRITERIA

- » Hepatitis may be confirmed by abnormal liver function tests, i.e. raised values of:
  - > AST,
  - > ALT,
  - > gamma GT.
  - > ALP,
  - > bilirubin, mainly the conjugated fraction,
- » Hepatomegaly and/or hepatosplenomegaly.
- » If conjugated hyperbilirubiniaemia – See above.

Investigations

- » Syphilis. See section 19.18: Syphilis, early congenital.
- » Galactosaemia.
- » Thyroid function (see hypothyroidism) and urine for MCS (see urinary tract infection).
- » Suspect galactosaemia if urine is positive for reducing substances but negative for glucose in a baby receiving lactose-containing feeds. A galactose-1-phosphate uridyl transferase assay will confirm the diagnosis.

GENERAL AND SUPPORTIVE MEASURES

- » Monitor bilirubin levels.
- » Treat the underlying cause.
- » Dietary adjustment for prolonged conjugated hyperbilirubinaemia to counteract the malabsorption of fat and fat soluble vitamins (A, D, E and K)
- » Avoid lactose containing feeds, i.e. breast milk and lactose containing formulae, when galactosaemia is suspected.
- » Regular follow up until the underlying condition has been resolved.

MEDICINE TREATMENT

- Fat soluble vitamins (A, D, E and K).

REFERRAL

- » Pathological jaundice, unconjugated and/or conjugated, where the underlying cause cannot be identified.
- » Serum unconjugated bilirubin at exchange transfusion level.
- » Jaundice, unconjugated and/or conjugated, not improving on a dequate treatment.
- » Conjugated hyperbilirubinaemia due to conditions requiring surgical intervention e.g. biliary atresia.
- » Prolonged neonatal jaundice, excluding breast milk jaundice.

PHOTOTHERAPY

South African Neonatal Academic Hospital Guidelines: 2006

In presence of risk factors use one line lower (the gestation below) until <1000g.

If gestational age is accurate, rather use gestational age (weeks) instead of body weight

Infants > 12 hours old with TSB level below threshold, repeat TSB level as follows:
1- 20µmol/L below line:repeat TSB in 6hrs or start phototherapy and rept TSB in 12- 24hrs,
21 - 50 µmol/L below line: repeat TSB in 12 – 24hrs,
>50 µmol/L below line: rept TSB until it is falling and/or until jaundice is clinically resolving

Infants under phototherapy :
Check the TSB 12 – 24 hly but if TSB >30 µmol/L above the line , check TSB 4 – 6hly.
STOP phototherapy :
If TSB > 50 µmol/L below the line. Recheck TSB in 12 – 24hr.

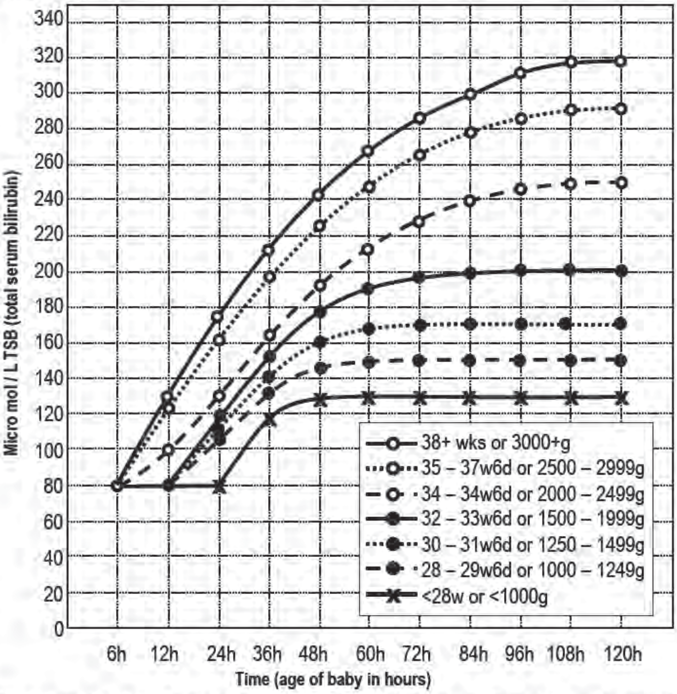

Start intensive phototherapy when the TSB is ≥ the line according to gestation or weight.
Published with permission from A Horn, P Henning, G Kirsten and SAMJ.
(SAMJ 2006;96:819-824)

EXCHANGE TRANSFUSION

South African Neonatal Academic Hospital Guidelines: 2006

In presence of sepsis, haemolysis, acidosis, or asphyxia,
use one line lower (gestation below) until <1000g
If gestational age is accurate, rather use gestational age (weeks) than body weight

Note: 1. Infants who present with TSB above threshold should have Exchange done if the TSB is not expected to be below the threshold after 6 hrs of intensive phototherapy.
2. Immediate Exchange is recommended if signs of bilirubin encephalopathy and usually also if TSB is >85 μmol/L above threshold at presentation
3. Exchange if TSB continues to rise >17 μmol/L/hour with intensive phototherapy

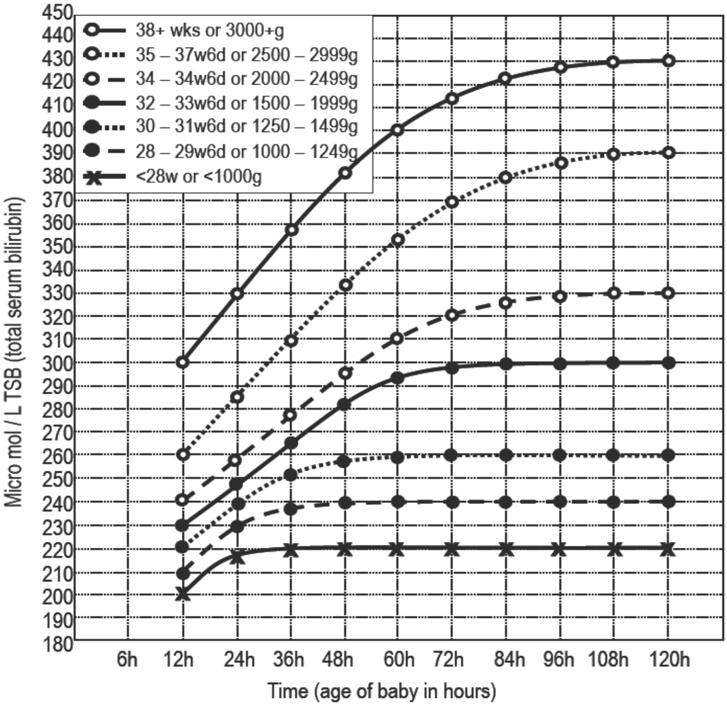

Published with permission from A Horn, P Henning, G Kirsten and SAMJ. (SAMJ 2006;96:819-824)

19.11 MENINGITIS BACTERIAL, NEONATAL

G01

DESCRIPTION

A bacterial infection of the meninges in the first month of life.

Consider meningitis in any neonate being evaluated for sepsis or infection, as most organisms implicated in neonatal sepsis also cause neonatal meningitis. The most common causative organisms are *Group B ß-haemolytic streptococcus type III* and Gram-negative organisms such as *E. coli* with K_1 antigen. Consider *S. epidermidis* and *S. aureus* as causative organisms with central nervous system anomalies such as open defects or with indwelling devices such as VP shunts.

Consider HIV infection in neonates with meningitis.

DIAGNOSTIC CRITERIA

Clinical

- » Clinical presentation is usually with one or more non-specific signs such as:
  - › temperature disturbances,
  - › lethargy,
  - › irritability,
  - › vomiting,
  - › feeding problems,
  - › vasomotor changes.
  - › altered level of consciousness,
  - › blood glucose disturbances,
  - › bulging/full fontanel,
  - › convulsions,
  - › apnoea, and
- » Complications include:
  - › cerebral oedema,
  - › raised intracranial pressure,
  - › vasculitis, with haemorrhage,
  - › ventriculitis,
  - › convulsions,
  - › hydrocephalus,
  - › subdural effusion,
  - › brain abscess,
  - › ischaemia and infarctions of the brain,
  - › inappropriate antidiuretic hormone (ADH) secretion.
- » Late complications include:
  - › neurological sequelae,
  - › deafness, and
  - › blindness,
  - › mental retardation.

SPECIAL INVESTIGATIONS

- » Lumbar puncture:
  - › CSF appears turbid to purulent.
  - › Protein concentration is increased.
  - › Leucocyte count is increased with a predominance of polymorphonuclear leucocytes.
  - › Glucose concentration is low, < $^{2}/_{3}$ of blood glucose.
  - › Counterimmune electrophoresis of CSF may provide a rapid diagnosis of some bacterial pathogens.
- » Gram stain, microscopy, culture and sensitivity of CSF. Rapid antigen tests on the CSF.
- » Blood cultures for microscopy, culture and sensitivity.

GENERAL AND SUPPORTIVE MEASURES

- Admit to high or intensive care unit, if available.
- Maintain a neutral thermal environment.
- Monitor, where indicated:
  - neurological status,
  - vital signs,
  - electrolytes,
  - haematocrit,
  - fluid balance (hydration),
  - calcium and magnesium,
  - acid-base status,
  - blood glucose,
  - serum and urine osmolality,
  - blood gases.
- Ensure adequate nutrition:
  - Enteral feeding where possible, use nasogastric tube, if necessary.
  - If enteral feeding is not possible, IV fluids, e.g. neonatal maintenance solution and parenteral nutrition under supervision by paediatrician.
- Limit total daily fluid intake, IV and oral:
  - Do not exceed the daily requirements for age.
  - Prevent fluid overload.

MEDICINE TREATMENT

Antibiotics, empirical

- Cefotaxime, IV, 50 mg/kg over 30 minutes, for 21 days.
  - If < 7 days of age: 50 mg/kg 12 hourly.
  - If 7 days – 3 weeks of age: 50 mg/kg 8 hourly.
  - If > 3 weeks of age: 50 mg/kg 6 hourly.

PLUS

- Ampicillin, IV, for 14 days.
  - If < 7 days of age: 50mg/kg 12 hourly.
  - If > 7 days – 3 weeks of age: 50 mg/kg 8 hourly.
  - If > 3 weeks of age: 50 mg/kg 6 hourly.

During the course of treatment a skull ultrasound should be done.
Repeat CSF examination after 48–72 hours to ensure there is a response to therapy.
Reconsider choice of antibiotic when the results of blood and CSF cultures become available or the child does not improve within 72–96 hours.

No response or intolerant to cephalosporins or ampicillin

For patients not responding to adequate antibiotic therapy where no organisms were identified or cultured, consider viruses, fungi and bacteria not usually causing meningitis.

Convulsions

See section 19.16: Seizure, neonate.

Raised intracranial pressure or cerebral oedema

Avoid fluid overload.
Limit total daily intake, IV and oral.
Do not exceed the maintenance requirements for age.

REFERRAL

» Meningitis not responding to adequate treatment.
» Meningitis with complications.
» Follow up is essential for assessing neurodevelopment, hearing and vision.

19.12 PATENT DUCTUS ARTERIOSUS (PDA) IN THE NEWBORN

Q25.0

DESCRIPTION

Patent ductus arteriosus (PDA) is the extra-uterine persistence of the normal fetal vessel that joins the pulmonary artery to the aorta.

DIAGNOSTIC CRITERIA

Clinical

Depending on size of PDA:

» systolic or continuous murmur at left heart base,
» hyperactive precordium with easily palpable bounding peripheral pulses.

Investigations

» Echocardiography will confirm the diagnosis.

Risk factors include:

» prematurity,
» hypoxia,
» fluid overload,
» anaemia, and
» pulmonary hypertension,
» sepsis,
» lung disease,
» congenital cardiac abnormalities.

Complications include cardiac failure, systemic hypotension, pulmonary haemorrhage and steal phenomena phenomena such as a decrease in mesenteric blood flow.

GENERAL AND SUPPORTIVE MEASURES

Preterm Infants

» Identify and treat underlying risk factors.
» Restrict fluid intake to 80–120 ml/kg/24 hours. Individualise volume to avoid over restriction of fluid and poor weight gain.
» Maintain haematocrit at ≥ 40% and Hb ≥ 13 g/dL.
» Monitor cardiac function, renal function and urinary output.
» Provide adequate nutrition.
» Nurse in neutral thermal environment.

MEDICINE TREATMENT

Cardiac failure

Diuretics

- Furosemide, IV/oral, 1 mg/kg/24 hours.

Closure of PDA in preterm infant less than 14 days of age

- Ibuprofen, oral
  First dose: 10 mg/kg. After 24 hours follow with 2 doses of 5 mg/kg 24 hours apart.
  Contraindications to ibuprofen therapy:
  - o Thrombocytopaenia (< 50 000/mm^3).
  - o Bleeding disorders.
  - o Impaired renal function.
  - o Jaundice approaching exchange transfusion levels.

SURGICAL TREATMENT

If medicine treatment is contraindicated or fails.

REFERRAL

» Patients with complications, e.g. c ardiac failure, pulmonary haemorrhage, ventilator dependence.
» PDA which remained patent despite adequate treatment.
» Term babies with symptomatic or persistent PDA.
» PDA in baby unable to take oral ibuprofen.

19.13 PREMATURITY/PRETERM NEONATE

P07.3

DESCRIPTION

Neonate born before 37 completed weeks of pregnancy.

GENERAL AND SUPPORTIVE MEASURES

» Admit unwell/unstable infants to neonatal high /intensive care facility.
» Temperature control:
  > Kangaroo mother care: Initiate if baby is well and vital signs are stable.
  > Provide a neutral thermal environment (incubator or infant crib with overhead heater) and keep ambient temperature at 26–28°C.
  > Keep infants temperature, axilla or skin of anterior abdominal wall, at 36.2–36.8°C.

Table for neutral thermal environment for age and body mass

Neutral Thermal Environment				
Age	Temperature for body mass range			
	< 1 200 g ± 0.5°C	≥ 1 200–1 500 g ± 0.5°C	≥ 1 500–2 500 g ±1°C	≥ 2 500 g ±1.5°C
0–12 hours	35	34.0	33.3	32.8
12–24 hours	34.5	33.8	32.8	32.4
2–4 days	34.5	33.5	32.3	32.0
4–14 days	33.5	32.1	32.0	
2–3 weeks	33.1	31.7	30.0	
3–4 weeks	32.6	31.4		
4–5 weeks	32.0	30.9		
5–6 weeks	31.4	30.4		

- Monitor to prevent or detect early the diseases/complications of prematurity:
  - respiratory rate,
  - haematocrit,
  - blood pressure,
  - bilirubin,
  - blood gas,
  - blood glucose,
  - acid-base status,
  - electrolytes,
  - calcium, magnesium,
  - hydration status, and
  - growth parameters.
- Nutritional support:
  - Give naso/orogastric tube feedings to infants with audible bowel sounds and no complications/diseases of prematurity.
  - Preferably use own mother's expressed breast milk, pasteurised donor breast milk or pre-term formula. Give small frequent bolus feeds, 1, 2 or 3 hourly or continuous naso/orogastric tube feeds (alternatives: cup, dropper, spoon, syringe).
  - Monitor gastric emptying by aspirating the stomach before each feed.
  - Consider stopping enteral feeding if:
    - aspiration of 3 mL or more of gastric contents before the next feed,
    - vomiting,
    - abdominal distension,
    - diarrhoea, or
    - ileus.
  - IV alimentation if enteral feeds are contraindicated or not tolerated.
- IV fluids to ensure adequate hydration, electrolyte and mineral intake, and normoglycaemia (blood glucose ≥ 2.6 mmol/L) until enteral (tube or oral) intake is satisfactory.
  - Discontinue IV fluids gradually to avoid reactive hypoglycaemia.
  - Discontinue the infusion when several oral feedings have been retained.
  - If renal function is compromised, use potassium-free solution.

Fluid requirements for a healthy premature infant	
Day of birth	mL/kg/24 hours
1	60
2	80
3	100
4	120
5	140
6 and onwards	160

Some infants may require fluid volumes up to 200 mL/kg/24 hours after day 6.

- Hospital discharge if:
  - clinically well,
  - able to breastfeed or formula feed,
  - able to maintain body temperature, and
  - usually > 1.8 kg.
- Follow-up visits to assess growth parameters, neurodevelopment, hearing and vision.

MEDICINE TREATMENT

To maintain haematocrit at 40% or Hb ±13 g/dL for the first 2 weeks of life:

- Packed red cells, IV, 10 mL/kg.

To maintain oxygen tension in the blood at 60–80 mm Hg:

- Oxygen, humidified via head box, or nasal cannulae.
  Oxygen therapy should be utilised to maintain oxygen saturation of haemoglobin at of 88–92%; and for babies less than 1 000 g, a saturation of 88–90%. Use pulse oximeter.

At birth

- Vitamin K, IM, 0.5–1 mg.
- Immunise according to EPI schedule.
- Iron and multivitamin supplementation from the third week of life.

Prophylaxis

- Iron (elemental), oral, 2–4 mg/kg/24/hours.
  - o Ferrous lactate 1 mL = 25 mg elemental iron.
  - o Multivitamin, oral, providing at least vitamin D, 400–800 IU and vitamin A, 1 250–5 000 IU per 24 hours.

Continue with iron and vitamin supplementation until the infant is on a balanced diet.

REFERRAL

Presence of one or more of the following complications that cannot be managed at the facility:

» Respiratory distress and/or apnoea attacks requiring ventilatory support.
» PDA with cardiac failure not responding to medical management.
» Necrotising enterocolitis requiring surgical intervention.
» Jaundice with serum unconjugated bilirubin level in the exchange transfusion zone.
» Septicaemic infants or infants with infections not responding to therapy.
» Pulmonary and/or intraventricular haemorrhage.
» Feeding difficulties where the underlying cause is unclear.
» Infants requiring hyperalimentation if parenteral nutrition is not available at the hospital.
» Convulsions not responding to treatment.
» Congenital abnormalities requiring surgical intervention.
» Hypoglycaemia not responding to treatment.
» For eye examination/hearing screening:
  - > infants less than 1.5 kg,
  - > infants < 32 weeks gestation,
  - > infants who received prolonged respiratory support/oxygen,
  - > infants with recurrent apnoea, and
  - > infants with an unstable clinical course.

19.14 RESPIRATORY DISTRESS IN THE NEWBORN

P22.9

DESCRIPTION

Newborn experiencing difficulty with breathing.
Causes of respiratory distress include:

PULMONARY CAUSES	EXTRAPULMONARY CAUSES
» hyaline membrane disease (surfactant deficiency),<br>» meconium aspiration,<br>» pneumonia,<br>» pneumothorax,<br>» wet lung syndrome,<br>» pulmonary haemorrhage,<br>» pulmonary hypertension,<br>» hypoplastic lungs, and<br>» diaphragmatic hernia.	» sepsis,<br>» cardiac failure irrespective of cause,<br>» hypothermia/hyperthermia,<br>» hypoglycaemia,<br>» anaemia,<br>» polycythaemia,<br>» hypovolaemic shock, and<br>» perinatal hypoxia.

DIAGNOSTIC CRITERIA

Clinical

» Pulmonary and/or extra pulmonary disorders presenting with two or more of the following signs in a newborn baby:
  - tachypnoea (≥ 60 breaths/minute),
  - expiratory grunting,
  - intercostal and sternal retractions (recession), and
  - central cyanosis while breathing room air.

Investigations

» Chest X-ray to determine underlying pathology.
» Echocardiography, if available, to exclude cardiac causes of respiratory distress.
» Haematocrit, blood glucose and temperature.
» Shake test to assess risk for hyaline membrane disease:
  - Within 15 minutes after birth place 0.5 mL gastric aspirate in a clean dry test tube.
  - Add 0.5 mL of sodium chloride 0.9% and replace the cap.
  - Shake well for 15 seconds.
  - Add 1 mL 95% alcohol to the 1 mL mixture of gastric aspirate and sodium chloride 0.9%.
  - Replace cap and shake well for 15 seconds.
  - Read at 15 minutes.
  - Interpretation of test:

Observation	Result	Risk
No bubbles on surface	Negative	High
Incomplete ring of bubbles on surface	Intermediate	Possible
Complete ring of bubbles or bubbles covering the entire surface	Positive	Very low

GENERAL AND SUPPORTIVE MEASURES

» Identify and treat underlying cause, e.g.:
  > Chest tube and underwater drainage of pneumothorax.
  > Isovolaemic dilutional exchange transfusion for symptomatic polycythaemia.
» Admit to neonatal high care/intensive care facility, if available.
» Handle neonate as little as possible.
» Nurse non-intubated infant in the prone position.
» Keep in a neutral thermal environment (incubator or infant crib with overhead heater). Keep room temperature, at 26–28°C, and anterior abdominal wall skin temperature at 36.2–36.8°C.
» Monitor:
  > blood pressure,
  > peripheral perfusion,
  > haematocrit,
  > blood glucose,
  > blood gases,
  > minerals and electrolytes and
  > respiratory rate,
  > heart/pulse rate,
  > acid-base status,
  > body temperature,
  > SaO_2,
  > fluid balance.
» Nutrition:
  > Provide adequate IV dextrose to maintain blood glucose ≥ 2.6 mmol/L.
  > Commence orogastric feeding after 12–24 hours if bowel sounds are audible and meconium has been passed.
  > If enteral feeding is not possible 24 hours after birth, start IV hyperalimentation.
» Ventilation (non invasive or invasive) is needed if:
  > An oxygen saturation of at least 90% or PaO_2 of at least 60mmHg cannot be maintained with an inspiratory oxygen concentration of ≥ 80% with or without nasal CPAP;
  > The PaCO2 rises to > 55 mmHg with uncompensated respiratory acidosis (pH ≤ 7.20), irrespective of oxygen saturation or PaO_2.
    (1kPa = 7.5 mmHg; 1 mmHg x 0.133 = 1 kPa)

MEDICINE TREATMENT

To eliminate central cyanosis and to maintain oxygen saturation of haemoglobin at 88–92%:

- Oxygen, warmed and humidified via head box, or nasal cannula.
  - o If a pulse oximeter or facility for blood gas analysis is available oxygen, humidified via head box, or nasal cannulae to maintain oxygen tension in the blood at 60–80 mmHg.
  - o If a pulse oximeter or facility for blood gas analysis is not available, regulate the inspired oxygen concentration in such a way that the least amount of oxygen that will prevent central cyanosis is used.
  - o Keep PaO_2 at 60–80 mmHg and $PaCO_2$ at 35–45 mmHg (arterial blood gas analysis).

Nasal CPAP is needed if the neonate has a good respiratory drive with a PCO_2 of ≤ 55 mmHg but unable to maintain a SaO_2 of 88–92% on an inspiratory oxygen concentration of ≥ 60% (F_1O_2) and pneumothorax has been excluded.
Administer nasal CPAP at 4– 6 cm H_2O and monitor SaO_2, blood gas and acid base status.

OR

- Oxygen/air mixture, hi-flow, warmed and humidified via nasal prongs. (Under specialist supervision)
  - o Do not exceed 6 L/minute. The flow/minute (L/min) approximates the pressure generated in cm water.

Stabilise circulation and blood pressure

- Neonatal maintenance solution, IV infusion, 60–80 mL/kg/24 hours (day 1 of birth) and adapt to daily maintenance requirements.

AND/OR

- Sodium chloride 0.9%, 10–20 mL/kg over 1–2 hours.
  - o For premature infants restrict to 10 mL/kg.

AND/OR

- Plasma, 10–20 mL/kg over 1–2 hours.

Inotropic support

- Dopamine, IV, 5 –15 mcg/kg/minute, continued until blood pressure has stabilised.
  - o Response to inotropic support will be unsatisfactory if the circulating blood volume is not corrected.

Anaemia

If anaemia is present, Hct < 40 % and Hb <13 g/dL:

- Packed red cells, IV, 10 mL/kg over 1–2 hours.

Metabolic acidosis

If pH ≤ 7.20 and the metabolic acidosis does not respond to normalisation of PaO_2, $PaCO_2$, blood pressure, volume expansion (hydration) and correction of anaemia:

- Sodium bicarbonate, 4.2 %, IV, administered slowly.

  1 mmol = 2 mL
  HCO_3 needed (mmol) = base excess x 0.3 x body mass (kg)
  (½ correct base deficit initially)

If blood gas and acid base analysis is not available and metabolic acidosis is suspected:

- Sodium bicarbonate, 4%, slow IV, 2 mL.

CAUTION
Do not administer Ca++ containing infusions with sodium bicarbonate solution

Polycythaemia
Treat with isovolaemic dilutional exchange transfusion using sodium chloride 0.9% if the venous haematocrit is Hct > 65%: Hb >22 g/dL and the baby is symptomatic. Perform under paediatrician's supervision.

$$\text{Volume to be exchanged (mL) if desired Hct} = 50: = \frac{[\text{Baby's Hct} - \text{desired Hct (i.e. 50)} \times \text{body mass (kg)}] \times 90}{\text{Baby's Hct}}$$

Hyaline membrane disease (Surfactant deficiency)
Supervision by paediatrician.
Shake test to assess risk for hyaline membrane disease and/or x-ray chest – see above.

If surfactant deficiency is suspected or present, provide respiratory support.
» Mild surfactant deficiency: nasal CPAP 4–6 cm H_2O.
» Moderate surfactant deficiency: "in-out" surfactant followed by nasal CPAP 4–6 cm H_2O. Intubate infant and administer surfactant via naso-or orotracheal tube. Ventilate for a few minutes with a Neopuff device or resuscitation bag with a CPAP generating device. Extubate baby and put on nasal CPAP 4–6 cm H_2O. Babies may be put on nasal CPAP directly after "in-out" surfactant administration, omitting the ventilation step following "in-out" surfactant.
» Severe surfactant deficiency: intubate baby and ventilate with a ventilator. Administer surfactant via the naso- or orotracheal tube. If a ventilator is not available the in-out surfactant followed by nasal CPAP can be used.

Semi-synthetic surfactant preparations are recommended while 100% synthetic preparations are not recommended.

Infection
If infection, e.g. bronchopneumonia, is present or suspected, give antibiotics after blood cultures have been taken.
Consider the antibiotic sensitivity profile of micro-organisms in a particular hospital when prescribing antibiotics.

▪ Aminoglycoside, e.g.:
- Gentamicin, IV, for 10 days in the first week of life.
  - If < 32 weeks gestation of age: 5 mg/kg/36 hours.
  - ≥ 32 weeks gestation of age: 5 mg/kg/24 hours.
  - After first week, 5 mg/kg/24 hours for all gestations.

PLUS
- Ampicillin, IV, for 10 days.
  - If < 7 days of age: 50 mg/kg 12 hourly.
  - If 7 days – 3 weeks of age: 50 mg/kg 8 hourly.
  - If > 3 weeks of age: 50 mg/kg 6 hourly.

Review after 72 hours. If infection is confirmed or very strongly suspected continue for 10 days.

REFERRAL

- » No improvement or deterioration despite adequate treatment.
- » Development of respiratory failure and need for ventilatory support.

19.15 RESUSCITATION OF THE NEWBORN

Be prepared!
Be at the delivery!
Check the equipment and emergency medicines!

Ask 3 questions to evaluate the infant:

1. Is the newborn breathing adequately and not just gasping?
2. Is the newborn's heart rate (HR) above 100 beats per minute?
3. Is the newborn centrally pink, i.e. no central cyanosis?

If the answer to all three questions is "yes", the newborn does not need resuscitation.
If the answer to all three questions is "no" the newborn needs resuscitation.

Assess the infant using the above 3 questions every 30 seconds during resuscitation. If the newborn is improving, then the intervention e.g. bagging can be stopped. Only if the baby is not responding or getting worse, is further intervention needed e.g. chest compressions (see algorithm).

Check that each step has been effectively applied before proceeding to the next step. The algorithm follows the assumption that the previous step was unsuccessful and the newborn is deteriorating.

Use the lowest inspiratory oxygen concentration to alleviate central cyanosis and restore a heart rate above 100 beats per minute. There is some evidence that resuscitation with 100% oxygen may be harmful to the baby.

An unsatisfactory response to resuscitation includes:

- » A sustained slow heart rate, usually less than, or equal to, 60/minute or a progressive decrease in heart rate until cardiac arrest occurs.
- » Episodes of cardiac arrest, with a progressively weaker response to chest compressions, positive pressure ventilation and medicines.
- » A decreasing blood pressure, increasing acidosis, severe hypotonia with central cyanosis or intense pallor.
- » Apnoea or weak, irregular and inefficient respiratory efforts.
- » Consider discontinuation of resuscitation if the unsatisfactory response to resuscitation persists for > 20 minutes and underlying conditions e.g. pneumothorax, diaphragmatic hernia have been excluded **or** > 10 min utes of u nresponsive cardiac arrest (asystole) and/or > 20 minutes of unsustainable respiration.
- » Admit newborns with a favourable response to resuscitation to a neonatal high or intensive care unit, if available, for post resuscitation care – See Section 19.8: Hypoxia/Ischaemia of the Newborn.

MEDICINES USED DURING NEONATAL RESUSCITATION

Medicine	Indications	Dosage	Effect
Epinephrine (adrenaline)	» asystole<br>» heart rate < 60/min	0.1 mL/kg of a 1:10 000 dilution, which may be repeated up to three times<br><br>ET, 1mL/kg of 1: 10 000 solution	↑Heart rate<br>↑Myocardial contractility<br>↑Arterial pressure
sodium bicarbonate -(4.2%) (1ml 4,2% = 0.5 mmol Soda Bic)	» life threatening metabolic acidosis<br>»pH < 7.2<br>»BE > –10 mmol/L<br>»$PaCO_2$ < 55 mmHg	slow IV, 2–4 mL/kg	Corrects metabolic acidosis. Improves cardiac output and peripheral perfusion.
naloxone	»maternal administration of opiates + apnoeic infant	ET/IV/SC/IM, 0.1 mg/kg	Corrects apnoea and/or hypoventilation.
Fluids<br>sodium chloride 0.9%	»hypovolaemia	slow IV, (5–10 min) 10–20 mL/kg	↑Blood pressure and improves tissue perfusion.
dextrose	»hypoglycaemia	IV, 250mg–500 mg/kg (2.5–5 mL/kg of 10% dextrose water)	Corrects hypoglycaemia.

Newborn Resuscitation Algorithm

The algorithm follows the assumption that the previous step was unsuccessful and the newborn is deteriorating)

Birth

Provide warmth
(Wrap preterm baby's body in plastic bag)
Clear airway if necessary
Dry and stimulate
Note the time

↓

30 seconds

Assess breathing / crying and/or heart rate

→ Crying/ breathing well and good tone → **Routine Care**
Maintain warmth.
Keep airway clear.
Ongoing evaluation

↓ Gasping, apnoeic or HR < 100

Start ventilating with room air (Rate: 30 - 40/min)
Connect to pulse oximeter, if available.
Give oxygen as necessary.
Ensure slight chest rise with each breath.

↓

30 seconds

Assess breathing and heart rate and sats/colour

↓ HR < 100

Continue ventilating with 100% oxygen.
(Bag connected to oxygen with flow at 5-10 L/minute)

↓

30 seconds

Assess breathing and heart rate and sats/colour

↓ HR < 60

Continue ventilating with 100% oxygen.
Start chest compressions (3 compressions: 1 breath).
Consider intubation or LMA.

↓

30 seconds

Assess breathing and heart rate and sats/colour

↓ HR < 60

Continue compressions and ventilations (3:1 ratio)
Give 0.1-0.3 mL/kg epinephrine IV (1:10 000 dilution)
(1 mL/kg epinephrine ET (1:10 000 dilution) ***only*** if no IV access)
May repeat epinephrine IV after 3 – 5 min.
Correct hypovolaemia if necessary (10 mL/kg IV over 5-10 min).
Consider pneumothorax.

If chest NOT moving:
M – Mask seal adequate?
O – Obstruction? (Secretions/Positional)
V – Ventilate more firmly?
I – Intubate if necessary?
N - Nasal choanal atresia?
G – Gastric distension?

Oxygen Administration
Use blended O2 if available to achieve targeted preductal sats (see below)

Alternatively:
- Bag with no O2 = ±21%
- Bag with O2 = ± 40%
- Bag with O2 ±reservoir = + 100%

Targeted pre-ductal sats after birth right hand or ear)

1 minute: > 60%
2 minutes: > 65%
3 minutes: > 70%
4 minutes: > 75%
5 minutes: > 80%
≥ 10 minutes: 85–95%

Published with kind permission from the Resuscitation Council of Southern Africa.

19.16 SEIZURES, NEONATAL

P90

DESCRIPTION

Neonatal seizures are usually secondary to a serum biochemical disorder or an underlying brain disturbance/injury/malformation. Seizures may be subtle due to the relatively underdeveloped cortex, and do not stop when limbs are flexed (as opposed to jitteriness).

The most likely causes are:

- » perinatal asphyxia,
- » birth trauma,
- » intracranial haemorrhage,
- » meningitis,
- » hypocalcaemia,
- » hypomagnesaemia,
- » hyponatraemia,
- » hypoglycaemia,
- » narcotic or alcohol withdrawal syndrome,
- » inborn errors of metabolism,
- » pyridoxine deficiency,
- » CNS developmental abnormalities.

DIAGNOSTIC CRITERIA

Categories of convulsions

- » Subtle seizures:
  - > tonic deviation of the eyes,
  - > 'swimming' movements of the arms,
  - > fluttering of the eyelids,
  - > 'cycling' movements of the legs,
  - > sucking and chewing movements,
  - > apnoea,
  - > vasomotor changes.
- » Tonic clonic movements.
- » Focal clonic movements.
- » Myoclonic movements.
- » Tonic movements/posturing.

GENERAL AND SUPPORTIVE MEASURES

- » Identify and treat the underlying cause, e.g. meningitis and hypoxic-ischaemic encephalopathy.
- » Ensure an open airway and administer oxygen, if necessary.
- » Nurse in neutral thermal environment.
- » Ensure adequate nutrition and hydration.
- » Monitor and maintain within accepted physiological range:
  - > respiration,
  - > heart rate,
  - > blood pressure,
  - > blood gases,
  - > SaO_2,
  - > body temperature.
  - > acid-base status,
  - > electrolytes,
  - > minerals,
  - > blood glucose,
  - > haematocrit,

MEDICINE TREATMENT

Seizure Control

Administer anticonvulsants with monitoring of cardiorespiratory function.

Phenobarbitone

- Phenobarbitone, IV.
  - Loading dose: 20 mg/kg administered over 10 minutes.
  - Refractory seizures: Additional 10 mg/kg/dose up to 40 mg/kg.
  - Maintenance: 4 mg/kg/day starting 12–24 hours after the loading dose.

> Do not use lidocaine if phenytoin was given.
> Do not use lidocaine for seizures in newborns with congenital heart disease, dysrhythmias, acute heart failure and shock.

Seizures refractory to phenobarbitone, should be admitted to a high or intensive care unit.

Cardiorespiratory support is usually required in this category of infants.

Lidocaine (lignocaine)

For term normothermic neonates:

- Lidocaine (lignocaine), IV.
  - Loading dose: 2 mg/kg administered over 10 minutes.
  - Follow with a continuous infusion of:
    6 mg/kg/hour for 6 hours, then,
    4 mg/ kg/hour for 12 hours, then,
    2 mg/kg for 12 hours.
  - If seizures are well controlled, slow taper lidocaine over 12 hours.

For preterm neonates:

A safe dose of lidocaine in preterm neonates has not been established but the following dosing schedule has been used.

- Lidocaine (lignocaine), IV.
  - Loading dose: 2 mg/kg administered over 10 minutes.
  - Follow with a continuous infusion of 3 mg/kg/hour for 3 days.
  - Gradually taper lidocaine over next 2 days.

> - A safe lidocaine dosing regimen for term infants undergoing hypothermia treatment for hypoxic ischaemic encephalopathy has not been established.
> - Clearance of lidocaine is slower in hypothermic neonates and preterm infants. There is a risk of accumulation.
> - Start tapering earlier than 3 days if seizures are well controlled.
> - Continuous monitoring of ECG, heart rate and blood pressure is mandatory if lidocaine is used.
> - Dysrhythmias and bradycardia are the main side effects of lidocaine. Life threatening dysrhythmias may indicate lidocaine toxicity.

Lidocaine toxicity:

- Lipid emulsion 20%, IV, 1.5 mg/kg administered over 1 minute.
  - o Follow with a continuous infusion of 0.25 mL/kg/minute for 30 minutes. (See Referral section.)

Pyridoxine deficiency:

- Pyridoxine, IV/IM, 20 mg/kg.

Maintenance anticonvulsant therapy

Maintenance anticonvulsant therapy is usu ally considered for neonates with underlying brain damage due to hy poxic ischaemic encephalopathy, meningitis, intracranial haemorrhage or birth trauma.

Continue until neonate is seizure-free for 2 weeks, then slowly taper to stop.

If seizures recur during tapering of an ticonvulsant therapy, continue with maintenance therapy.

Follow-up by medical practitioner or at clinic/hospital after discharge

Note:

Patients with head or whole body cooling should have an adj ustment of th e anticonvulsant doses.

Hypocalcaemia

Serum total calcium ≤ 1.8 mmol/L, or ionized calcium < 0.7 mmol/L.

- Calcium gluconate 10%, IV, 100–200 mg/kg/dose administered over 10 minutes.
  - o Dilute 1:4 with dextrose 5% water.
  - o Administer under ECG control over 5 minutes (preferred) or until seizure ceases. Repeat if necessary.
    (1 mL of 10% calcium gluconate = 100 mg calcium gluconate)

Hypoglycaemia

Serum glucose < 2.6 mmol/L.

- Dextrose, IV as bolus, 250–500 mg/kg.
  - o Follow with 6–12 mg/kg/minute or more until blood glucose is within the physiological range.
    (10% Dextrose = 10g dextrose/100mL)

Hypomagnesaemia

Serum magnesium < 0.6 mmol/L.

- Magnesium sulphate 50%, IV, 0.25 mL/kg administered slowly over 3 minutes as a single dose.

REFERRAL

» Seizures not responding to adequate therapy.
» Seizures where the underlying cause is unclear.
» Refractory cases for further treatment and aEEG monitoring.
» Lidocaine toxicity.

19.17 SEPTICAEMIA OF THE NEWBORN

P36.9

DESCRIPTION

Bacterial or fungal invasion of blood before or after birth, which may spread to involve other organs/systems, e.g. meninges (meningitis), lungs, (pneumonia), bone (osteomyelitis), and kidneys (pyelonephritis).

DIAGNOSTIC CRITERIA

Clinical

The baby usually presents with one or more non-specific clinical sign e.g.:

- vasomotor changes,
- feeding problems,
- lethargy,
- jaundice,
- diarrhoea,
- tachypnoea,
- temperature disturbances,
- apnoea attacks,
- sclerema,
- acidosis,
- abdominal distension,
- tachycardia,
- organomegaly,
- petechiae,
- convulsions,
- blood glucose disturbances,
- hypotonia,
- shock,
- anaemia,
- cyanosis.
- Complications include:
  - septic shock,
  - hypoglycaemia,
  - apnoea,
  - convulsions,
  - anaemia,
  - meningitis,
  - bronchopneumonia,
  - cardiac failure,
  - dehydration,
  - bleeding tendency,
  - DIC and/or thrombocytopenia,
  - metabolic acidosis,
  - osteomyelitis,
  - respiratory failure,
  - necrotising enterocolitis,
  - ileus,
  - renal failure,
  - multi-organ failure.

Investigations

- Blood and cerebrospinal fluid cultures.
- Blood count and differential count.
- C-reactive protein and procalcitonin, if available.

GENERAL AND SUPPORTIVE MEASURES

- Admit to neonatal high or intensive care facility, if available.
- Ensure a neutral thermal environment.
- Start infusion with appropriate IV fluid, e.g. neonatal maintenance solution.

» Ensure adequate nutrition:
  > enteral feeding where possible, via oro/nasogastric tube after ileus, obstruction, or other contraindications to enteral feeding have been excluded.
  > if enteral feeding is not possible, IV fluids, e.g. neonatal maintenance solution and parenteral nutrition under supervision by paediatrician
» Insert naso/orogastric tube.
» Oxygen to maintain PaO_2 at 60–80 mmHg or oxygen saturation of haemoglobin at 88–92%.
» Ventilatory support if $PaCO_2$ exceeds 55 mmHg.
» Monitor:
  > Body temperature 36.2–36.8° C (axillary or anterior abdominal wall).
  > Maintain blood glucose level of 2.6–6.8 mmol/L.
  > Acid-base status and maintain blood pH of 7.35–7.45.
  > Maintain a haematocrit of 40%.
  > Vital signs and respiration, and maintain blood electrolytes and minerals within their normal physiological ranges.
  > Clinical progress and for the emergence of complications.

MEDICINE TREATMENT

Antibiotic therapy

Reconsider choice of antibiotic when the results of blood and CSF cultures become available or the child does not improve within 72–96 hours.
Be aware of the antibiotic sensitivity/resistance profile of bacterial pathogens in your hospital/community.

Empiric treatment:

- ▪ Aminoglycoside, e.g.:
- Gentamicin, IV, for 10 days.
  - o If < 32 weeks gestation: 5 mg/kg/36 hours in the first week of life.
  - o If ≥ 32 weeks gestation: 5 mg/kg/24 hours in the first week of life.
  - o Monitor blood levels.

PLUS

- ▪ 3rd generation cephalosporin, e.g.:
- Cefotaxime, IV, for 10 days.
  - o If < 7 days of age: 50 mg/kg 12 hourly.
  - o If ≥ 7 days of age: 50 mg/kg 8 hourly.

Fungal infections

Where fungal septicaemia is demonstrated or suspected:

- Amphotericin B, IV, 1–1.5 mg/kg/24 hours infused over 2 hours for 14 days.
  - o Monitor renal function and serum potassium.

Anaerobic infections
Where anaerobic infection is likely, e.g. after gastro-intestinal surgery for sepsis, or where intra-abdominal sepsis is suspected:

- Metronidazole, oral/IV, for 10 days.
  - Loading dose, IV: 15 mg/kg administered over 60 minutes.
  - If ≤ 4 weeks of age: 7.5 mg/kg 12 hourly.
  - If ≥ 4 weeks of age: 7.5 mg/kg 8 hourly.

Inotropic support
Mean blood pressure should not be less than the gestational age (weeks) of the infant plus 5–10 mmHg.

If blood pressure is < $^{60}/_{40}$ mmHg in term infant or < $^{50}/_{35}$ mmHg in pre-term infant:

- Dopamine, IV, 5–15 mcg/kg/minute as a continuous infusion.
  - Continue with dopamine as long as it is necessary to maintain the blood pressure.

REFERRAL

» Septicaemia with complications.
» Septicaemia not responding to treatment.

19.18 SYPHILIS, EARLY CONGENITAL

A50.9
*Notifiable condition.

DESCRIPTION

Multi-organ infection caused by *T. pallidum* and acquired by vertical transmission via the transplacental route during pregnancy.

DIAGNOSTIC CRITERIA

Clinical

» Suspect if mother has syphilis or positive serology for syphilis and the baby a positive non-treponemal serological test at birth with a titre significantly higher than that of the mother.
» Large pale greasy placenta.
» The following signs may be present at birth or will develop within the first 3 months of life:
  - hydrops fetalis,
  - anaemia,
  - hepatosplenomegaly,
  - oedema,
  - condylomata,
  - hepatitis,
  - nephrosis/nephritis,
  - thrombocytopaenia,
  - lymphadenopathy,
  - jaundice,
  - hypoalbuminaemia,
  - pneumonia alba,
  - meningitis,
  - interstitial keratitis, and
  - transient bullous lesions, commonly on the hands and feet with later desquamation and an erythematous appearance of palms and soles.

- » A generalised reddish maculopapular rash which may also desquamate.
- » Rhinitis with mucopurulent bloodstained discharge excoriating the upper lip.
- » Other mucocutaneous lesions of the mouth, anus and genitalia, healing with scars, especially the corners of the mouth and on the chin.
- » Involvement of long bones with/without pseudoparalysis of one or more limbs and radiological findings.

Investigations:

If mother is positive for syphilis:

- » X-ray of long bones:
  - > translucent metaphyseal bands,
  - > osteochondritis,
  - > osteitits, and
  - > metaphysitis and periostitis.
- » Confirm syphilis with:
  - > Non-treponemal serological tests, i.e. RPR, VDRL.

  If there is reason to believe that RPR or VDRL is falsely negative or positive, then do a:
  - > *Treponema pallidum* haemoglutination test (TPHA), or
  - > Fluorescent Treponema antibody absorption test (FTA – ABS) for both IgG and IgM.

  Do not use umbilical cord blood at delivery for laboratory investigations.

GENERAL AND SUPPORTIVE MEASURES

- » Nurse infant in a neutral thermal environment.
- » Maintain adequate nutrition and hydration.
- » Monitor hepatic and renal function.

Pneumonia

To maintain oxygen saturation at 88–92% or PaO_2 at 60-80 mmHg:

- Oxygen via a head box or nasal cannulae.

  1 kPa = 7.5 mmHg

  1 mmHg x 0.133 = 1 kPa

Anaemia

If Haematocrit < 40% (Hb < 13 g/dL):

- Packed red cells, 10 mL/kg administered over 3 hours.

MEDICINE TREATMENT

Asymptomatic, well baby

Mother seropositive or result unknown, and mother has not been treated or was only partially treated:

- Benzathine benzylpenicillin (depot formulation), IM, 50 000 units/kg as a single dose into the antero-lateral thigh.

Symptomatic baby
- Procaine penicillin, IM, 50 000 units/kg daily for 10 days (not for IV use).

OR
- Benzylpenicillin (Penicillin G), IV, 50 000 units/kg 12 hourly for 10 days.

> **CAUTION**
> **Procaine penicillin and benzathine benzylpenicillin must not be given intravenously.**

Prevention
Screen pregnant women for syphilis at first visit and repeat during the second and/or third trimester.
Investigate and treat both parents, if necessary.

REFERRAL
» Symptomatic infant with complications, e.g. respiratory failure, hepatic failure, nephrotic syndrome and meningitis.

19.19 TETANUS, NEONATAL
A33

DESCRIPTION
Tetanus is an a cute spastic paralytic illness caused by tetanospasmin, the neurotoxin produced by *C. tetani*. Neonatal tetanus is the most common form of the disease, usually caused by umbilical stump infections or contamination.

The disease only occurs in infants of non-immunised mothers or mothers with insufficient levels of protecting antibody to tetanus toxin.

DIAGNOSTIC CRITERIA
Clinical signs
» Presents with difficulty in sucking and swallowing due to masseter spasm, i.e. trismus, usually on day three with associated hunger and crying.
» Temperature of 40–41 °C.
» Tenseness and rigidity of all muscles, including paraspinal and abdominal muscles.
» Fists clenched and the toes fanned.
» Opisthotonic spasms and clonic jerks following sudden stimulation by touch and noise:
  > spasms are painful,
  > not true seizures,
  > there is no loss of consciousness, and
  > laryngeal spasms may result in respiratory distress.
» Umbilicus may appear normal but there may be discha rge from, or di rt/dung on umbilicus.

- Complications include:

  Acute:

  - aspiration pneumonia,
  - pulmonary haemorrhage,
  - respiratory failure,
  - CNS haemorrhage,
  - urinary retention,
  - rhabdomyolysis, and
  - cardiac dysrhythmias,
  - unstable blood pressure,
  - asystole,
  - starvation,
  - bleeding into muscles,
  - iatrogenic paralytic ileus.

  Chronic:

  - contractures,
  - myositis ossificans,
  - peripheral paresis,
  - muscle weakness and atrophy, and
  - secondary neurologic sequelae due to hypoxic cerebral injury, including mental retardation, cerebral palsy etc.

Special Investigations

- Gram-stain of infected umbilical stump may reveal typical Gram positive bacilli.
- Anaerobic cultures are not necessary as attempts to culture *C. tetani* have a poor yield.
- Gram stain of cerebrospinal fluid may be required to rule out meningitis.

GENERAL AND SUPPORTIVE MEASURES

- Majority of cases will require admission to neonatal ICU, full intermittent positive pressure ventilation, muscle relaxation and sedation. If not available, nurse in quiet, cool and dark environment.
- If not intubated, suction the mouth and turn infant 30 minutes after each dose of sedative.
- Insert a nasogastric tube 30 minutes after sedative was given:
  - Start nasogastric tube feeds preferably after 24 hours of admission.
  - Give expressed breast milk in small feeds and augment with IV neonatal maintenance solution as required.
- Cut off umbilical stump if present and clean with solution of chlorhexidine and water 2 hours after human tetanus immunoglobulin (TIG) was given.
- Physiotherapy is important to prevent muscle atrophy and contractures. Sedate infant before physiotherapy. Limit unnecessary stimulation, i.e. sound, touch and therapeutic manipulation.
- Monitor and maintain body temperature.
- Cardiorespiratory monitoring is important due to involvement of respiratory muscles and sympathetic over activity, i.e. hypertension and tachycardia.
- Careful nursing attention to bladder and bowel function:
  - Bladder may successfully be emptied using Credé's method.
  - Urine retention may occasionally require bladder catheterisation.
  - Prevent constipation by giving expressed breast milk. If necessary, glycerine suppositories may be used, once muscle spasms become less frequent and always with prior treatment with sedatives and muscle relaxant (see Medicine Treatment).
- Place small balls of cotton wool in clenched fists and put splints on feet when muscle relaxants are given. Remove them daily to check for pressure sores.

MEDICINE TREATMENT

- Human anti tetanus immunoglobulin, IM, 500 units.
  - o Split the volume and administer at 2 different sites.

PLUS

- Metronidazole, IV, for 14 days:
  - o Loading dose: 15 mg/kg administered over 60 minutes
  - o Maintenance dose: < 4 weeks: 7.5 mg/kg/dose 12 hourly.
    ≥ 4 weeks: 7.5 mg/kg/dose 8 hourly.

PLUS

- Tetanus toxoid, IM, 0.5 mL into deltoid muscle.

For non-ventilated patient sedate with:

- Chlorpromazine, oral, 1 mg/kg/dose, 8 hourly via nasogastric tube.

AND

- Phenobarbitone, oral, 4 mg/kg once daily.

For intubated and ventilated patients:

- Diazepam, IV, 0.1 mg/kg/dose, 2–4 hourly, as necessary to control spasms in first few days.
  - o Treatment is sustained for 2–4 weeks and frequency of administration is decreased as patient improves.
  - o Titrate dose according to response.
  - o Change to oral as intravenous preparation can cause thrombophlebitis.

AND

- Vecuronium bromide, IV, 0.1–0.2 mg/kg, 2-4 hourly as necessary.
  - o Decrease frequency of administration as spasms become less frequent and less forceful, usually within 7 days.

AND

- Morphine, IV, 0.05–0.1 mg slowly, every 4–6 hours.

Once infant has improved, replace with

- Paracetamol, oral, 10 mg/kg/dose 4-6 hourly.

Constipation

- Glycerine, rectal, ¼ suppository every 2nd day.

Aspiration pneumonia

Treat as for nosocomial infections – See section 15.3.2.5: Pneumonia, nosocomial.

Preventive management

Prevention of neonatal tetanus can be accomplished by prenatal immunisation of the previously unimmunised mother.
Pregnant women who have not completed their primary immunisation series should do so before delivery, if possible.

All pregnant women:

- First pregnancy – three doses of tetanus toxoid:
  - **first dose** on first contact
  - **second dose** 4 weeks later
  - **third dose** 6 months later even if it is given in the post natal period (after birth)
- Subsequent pregnancy:
  - one **dose** during the antenatal period **(up to a total of 5 recorded doses)**

Active immunisation of the infant against tetanus should always be undertaken during convalescence, because the disease does not confer immunity.

REFERRAL

» All infants with complicated neonatal tetanus.
» Onset of neonatal tetanus within the first 3 days of life.

CHAPTER 20
PAIN CONTROL AND PALLIATIVE CARE IN PAEDIATRICS

20.1 MANAGEMENT OF PAIN

DESCRIPTION

Pain is a subjective unpleasant experience comprising sensory and emotional components.

Pain needs to be recognised and assessed before it can be managed appropriately.
Self-report of pain is the gold standard of pain assessment in children, but this is not possible in non- or pre-verbal children.
Parental report gives valuable information. Assessment of physiological and behavioural responses to pain is also important.
Physiologic features of pain and anxiety include tachycardia, hypertension and sweating. These may be absent in chronic pain.
Behavioural features in acute pain: crying, moaning, irritability, facial grimacing, thrashing, jerking, fisting, arching.
Behavioural manifestations in chronic pain are more subtle and are often overlooked: features include apathy, disinterest, depression and decreased activity level. If the pathology or procedure would cause pain in an adult, it is likely to be painful for a child.

PAIN SCALES TO ASSESS AND REVIEW PAIN MANAGEMENT

FLACC scale:

» For use in children under 3 years or older non-verbal children.
» Evaluate each item and arrive at a total score/10.

Item	0	1	2
**Face**	No particular expression or smile	Occasional grimace or frown, withdrawn disinterested	Frequent to constant frown, clenched jaw, quivering chin
**Legs**	Normal position or relaxed	Uneasy, restless, tense	Kicking, or legs drawn up
**Activity**	Lying quietly, normal position, moves easily	Squirming, shifting back and forth, tense	Arched, rigid or jerking
**Cry**	No cry (awake or asleep)	Moans or whimpers, occasional complaint	Crying steadily, screams or sobs, frequent complaints
**Consolability**	Content, relaxed, no need to console	Reassured by occasional touching, hugging or "talking to", distractible	Difficult to console or comfort

Revised Faces Pain Scale

» Use in children over 4 years.
» Ask them to point to the face that best depicts their level of pain.

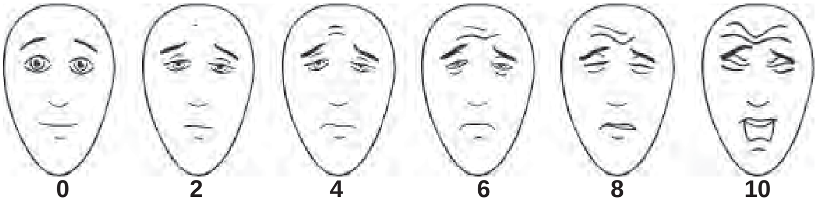

The primary aim is to alleviate pain.
Choose the appropriate pain relief according to the severity of pain.
Re-assess degree of response and adjust accordingly.
Do not hesitate to start with a strong analgesic in cases of severe pain.

Basic principles of pain management

» Treat the underlying cause.
» Determine the pathophysiology of the pain to determine the most suitable treatment (e.g. noiciceptive vs neuropathic).
» Use both medicine and non-medicine measures.
» Address associated psychosocial distress (e.g. separation anxiety).
» Continually re-evaluate pain and its response to treatment.

GENERAL AND SUPPORTIVE MEASURES

» Discuss the management with the family, including the child, as appropriate for development
» Address all factors that may contribute to pain and associated symptoms e.g. family stress, anxiety and sleep deprivation. Address parental anxiety.
» Where possible, allow a parent to room-in or stay with the child as long as possible.
» Make sure that the child is comfortable e.g. nappy is clean and dry, child is fed.
» Use non-medicine therapies and distraction e.g. massage, splints, music or play therapy and storybook reading, where appropriate.
» For babies up to three months of age: dummy moistened with sucrose 24% given 2 minutes before the painful procedure may provide some comfort.

Pain relief for painful procedures of short duration

For some procedures both local a naesthetic and systemic treatment is necessary to relieve anxiety and pain e.g. insertion of arterial line, placement of central venous line/intercostal drainage tube, needle pricks, etc.

If pain is likely to be on-going post procedure remember to prescribe continued analgaesia.
For some procedures, e.g. to remove an intercostal drain or deep wound drain or stitches, it is necessary to give sedation in combination with systemic pain treatment. Sedation should never be used on its own without analgesics for procedural pain.

For needle prick site/lumbar puncture, except in emergency situations

- Lidocaine/prilocaine cream, topical, applied at least 30 minutes before procedure.
  - Apply 1–1.5 cm length of cream over the needle puncture site.
  - Spread cream thinly over 1 cm radius on skin and cover with polyurethane film dressing.

Burns
Children with large burns require regular analgesia. (Refer to persistent pain below)
Provide analgesia cover at each dressing change. See section 1.2.1: Burns.
In children with major burns, dressings should be changed under general anaesthesia.

Short acting analgesics for invasive local procedures

Take standard precautions for respiratory arrest.

- Ketamine, oral, 5 mg/kg **or** IV, 1–2 mg/kg.

OR

- Midazolam, IV, 0.1–0.2 mg/kg or oral, 0.5 mg/kg or intranasal, 0.2 mg/kg.
  - Note: Midazolam has no analgesic effect.

Before administering the sedative/anxiolytic/analgesic drug

» Withhold food for 4 hours before planned procedure.
» Monitor child and have resuscitation equipment available.
» Put up an intravenous line with heparin lock in case an unexpected complication arises.
» Where appropriate, written informed consent should be obtained.

PAIN RELIEF FOR PERSISTENT PAIN

The correct use of the correct analgesic will relieve most pain in children.
Treatment should be individualised as pain experiences vary from child to child.

The broad principles of analgesic use in children:

» By the clock (regular rather than as required dosing).
» By the correct route for the type of pain (preferably oral, avoid IM injections).
» By the child (individualise treatment).

Non-opioid medicines

- Paracetamol, oral.
  - o Loading dose 20mg/kg/dose, then 15 mg/kg/dose 4–6 hourly.

OR

Where oral medication cannot be used:

- Paracetamol, suppositories, 6 hourly.
  - o If 3–12 months of age: 62.5–125 mg.
  - o If 1–5 years of age: 125–250 mg.
  - o If 6–12 years of age: 250–500mg.

Non-steroidal anti-inflammatory drugs (NSAIDS)

Where anti-inflammatory effect is required.

Can be used in combination with paracetamol or opioids.

- Ibuprofen, oral, 10 mg/kg/dose 8 hourly with meals.

Intermediate efficacy opioid

- Tilidine, oral, 1 drop per 2.5 kg of body weight (i.e. 1 mg/kg/dose).

Strong opioid

- Morphine, oral (Immediate release morphine (liquid))
  - o Starting dose:
    - o If 0 – 1 month of age: 0.05 mg/kg 6 hourly.
    - o If > 1–12 months of age: 0.1 mg/kg/dose 4 hourly.
    - o If > 12 months of age: 0.2–0.4 mg/kg/dose 4 hourly.
  - o Dosing is 4 hourly except in patients with delayed clearance, i.e. newborns, hepatic and renal dysfunction where prescribed 6 hourly.

  In non-palliative care, the increase in dose of morphine is limited by side-effects, i.e. hypotension and respiratory depression.

In palliative care the dose of morphine is titrated to the patient's pain control. Increase dose by 30–50 % with each dose if pain control is sub-optimal. There is no maximum dose of morphine.

  - o Breakthrough dose: 50–100% of the regular pain dose.
  - o Give regular and breakthrough doses at least one hour apart.
  - o If the child requires breakthrough doses less than an hour after the regular dose has been given then it is likely that the regular dose needs to be increased.
  - o At the end of a 24 hour period all the breakthrough doses should be added up and this value divided by 6 to determine the increase needed in the regular dose for the next day.
  - o Increase breakthrough dose as regular doses are increased.

If patient is unable to swallow or is vomiting:

- Morphine, IM, 4-hourly bolus.
  - Starting dose:
    - If > 1–6 months of age: 0.05 – 1 mg/kg.
    - If > 6 months of age: 0.1 – 0.2 mg/kg.

In all patients receiving morphine:

- Lactulose, oral, 2.5-10 mL 12 hourly.

Children receiving properly titrated doses of analgesics, including opioids, do not become addicted**.** There is a difference between tolerance, which is a need for escalating doses to achieve the same therapeutic effect, and addiction.

Withdrawal from opioids
This must be done for any child who has received morphine for more than 5–7 days. Wean by decreasing the daily dose by one third for three days.

Adjunctive analgesic therapy
Adjuvant analgesics are drugs that have weak or non-existent analgesic action when administered alone but can enhance analgesic actions when co-administered with known analgesic agents.

Steroids
Steroids may be used as an adjuvant in consultation with a specialist for the following indications:

» infiltration of bone/meninges,
» compression of nerves and spinal cord,
» visceromegaly,
» tumour invasion of organs,
» stretching of periosteum or peritoneum.

Stretching of periosteum or peritoneum:

- Prednisone, oral, 1–2 mg/kg/day as single dose or in 2 divided doses.

Neuropathic pain:

- Carbamazepine, oral, 5 mg/kg/dose 12 hourly.

REFERRAL

» Adequate analgesia will control pain in almost all cases. Discuss resistant cases with a specialist.

20.2 PALLIATIVE CARE

Icd10

DESCRIPTION

Palliative care is an approach that aims to improve the quality of life of patients and their families facing the problem associated with life-threatening illness, through the prevention and relief of suffering by means of early identification and impeccable assessment and treatment of pain and other problems, physical, psychosocial and spiritual.

A key component to relieving suffering is the management of distressing symptoms that include both pain and non-pain symptoms (e.g. nausea, anxiety, etc). There are certain key principles that should be applied when managing these symptoms, i.e.:

» Determine and treat underlying cause of the symptom including non-physical causes.
» Relieve the symptom without creating new symptoms or unwanted side effects.
» Consider different types of interventions: drug and non-drug interventions.
» Consider whether the treatment is of benefit to the individual patient.

Common non-pain symptoms and other problems in paediatric palliative care are described below.

Nausea and vomiting

Appropriate treatment depends on the cause of nausea and vomiting.

- Metoclopramide, oral, 0.1 mg/kg/dose, 6–12 hourly.

Use with caution as extrapyramidal side effects may occur (especially at higher doses)

OR

- Ondansetron, IV, 0.1 mg/kg.
  - o Maximum dose: 4 mg/day.

Anxiety

- ▪ Benzodiazepine, e.g.:
- Diazepam, oral, 8 hourly.
  - o If > 2–12 years of age: 2–3 mg.
  - o If >12–18 years of age: 2–10 mg.

Constipation

» Laxatives should be used prophylactically in all patients receiving morphine.
- Lactulose, oral, 2.5–10 mL 12 hourly.

Dyspnoea

» Reduce anxiety by addressing psychosocial factors e.g. parental separation.
» Consider home oxygen concentrator.
- Morphine, oral (Immediate release morphine (liquid))
  - o Starting dose:
    - o If 0–1 month of age: 0.05 mg/kg 6 hourly.
    - o If > 1–12 months of age: 0.1 mg/kg/dose 4 hourly.
    - o If > 12 months of age: 0.2–0.4 mg/kg/dose 4 hourly.

Muscle Spasms

» Where possible, refer for physiotherapy and/or occupational therapy.
▪ Benzodiazepine, e.g.:
- Diazepam, oral, 8 hourly.
    - o If > 2–12 years of age: 2–3 mg.
    - o If >12–18 years of age: 2–10 mg.

PLUS

- Morphine, oral (Immediate release morphine (liquid))
  - o Starting dose:
    - o If 0–1 month of age: 0.05 mg/kg 6 hourly.
    - o If > 1–12 months of age: 0.1 mg/kg/dose 4 hourly.
    - o If > 12 months of age: 0.2–0.4 mg/kg/dose 4 hourly.

Pruritus

- Promethazine, IV/oral, 0.1 mg/kg/dose 6 hourly.

Spasmodic abdominal pain

- Hyoscine butylbromide, IV/oral, 0.5 mg/kg/dose 6–8 hourly.

Oral care

- Zinc and castor oil cream, topical, applied to lips every 2 hours.

- Sodium chloride solution, gargle, to rinse mouth
  - o Dissolve 5 g sodium chloride in 1 L of water.

OR

- Chlorhexidine 0.2%, 10 mL as a mouthwash or gargle, 12 hourly.
  - o Do not swallow.

Apthous ulcers

See section: 2.1.5 Aphthous ulcers.

Mucositis

- Chorhexidine 2%/benzydamine, oral rinse, rinse or gargle 6–8 hourly.

Perineal mucositis/nappy rash

- Zinc and castor oil cream, topical, applied as needed
  - o If pain is a feature: mix with lidocaine 2% gel.

In no improvement within 3 days, suspect candida and:
ADD

- Clotrimazole 2% cream followed by zinc and castor oil ointment applied after each nappy change.

Secretions

» Suctioning and re-positioning is often helpful.
» Attention to oral hygiene is essential.
- Hyoscine butylbromide, IV/oral, 0.5 mg/kg/dose 6–8 hourly.

REFERRAL

Discuss with a specialist:
» Children with symptoms not described here.
» Children not responding to management.

CHAPTER 21

INTENSIVE CARE AND ANAESTHETICS

Healthcare professionals engaged in intensive care and anaesthetics must undergo appropriate training

21.1 SEDATION FOR INTENSIVE CARE PROCEDURES

21.1.1 ICU SEDATION, NEONATE

DESCRIPTION

Sedation is required for various procedures and situation in newborns in an intensive care setting to decrease discomfort and suffering, and to improve the management outcomes of the procedure/care that is being given.

GENERAL AND SUPPORTIVE MEASURES

In all situations appropriate control of the environment, provision of normal physiological requirements, monitoring of vital signs and provision of comforting care should be incorporated in the care of neonates and minimise the stress on the child.

MEDICINE TREATMENT

1. For controlled endotracheal intubation for ventilation

This skill should have been learnt in an appropriate learning situation. If not, endotracheal intubation should take place under supervision of a specialist.

Pre-oxygenate with bag-mask, T-piece or equivalent ventilator.
Maintain oxygen saturation between 88–92%.

- Propofol, IV, 2.5 mg/kg. Adjust as necessary.

OR

- Midazolam, IV, 0.1–0.2 mg/kg. Caution in premature babies.

OR

- Ketamine, IV, 1–2 mg/kg.

PLUS

- Succinylcholine, IV, 2 mg/kg. **Causes paralysis and apnoea.**
    - o Note: Avoid succinylcholine in patients with potassium disturbances and neuromuscular disease.

PLUS

If bradycardia or using ketamine:

- Atropine, IV, 0.02 mg/kg.
  - o Maximum dose: 0.6 mg.

If emergency with no IV line, consider:

- Ketamine, IM, 5–10 mg/kg.

2. Short term intubation (In-out endotracheal surfactant administration)
Nasal CPAP as required. If inadequate oxygenation on nasal CPAP, pre-oxygenate with bag-mask or T-piece ventilation to maintain saturation between 88-92%.
Intubate orally, give surfactant, follow with gentle manual ventilation or CPAP, as required, for 5 minutes.
Extubate and recommence nasal CPAP.

3. During continuous mechanical ventilation
For pain and sedation:

- Fentanyl, IV, 5–10 mcg/kg.
  - o If necessary, follow with IV infusion, 50 mcg/mL at 0.1–0.2 mL/kg/hour, i.e. 5–10 mcg/kg/hour.

OR

- Morphine, IV, 10–30 mcg/kg/hour.
  - o i.e. Morphine 1 mg/kg mixed with 50 mL dextrose 5% or sodium chloride 0.9% at 0.5–1.5 mL/hour.

OR

- Sucrose 24% solution, buccal as needed.

REFERRAL

» Inability to provide appropriate care.

21.1.2 ICU SEDATION, INFANT AND CHILD

DESCRIPTION

Sedation is required for various procedures and situations in infants and children in an intensive care setting to decrease discomfort and suffering and to improve the management outcomes of the procedure/care that is being given.

GENERAL AND SUPPORTIVE MEASURES

In all situations appropriate control of environment, provision of normal physiological requirements, monitoring of vital signs and provision of comforting care should surround the care of inf ants and children and minimises the stress on the child.

MEDICINE TREATMENT

This skill should have been learnt in an appropriate learning situation. If not, endotracheal intubation should take place under supervision of a specialist.

Pre-oxygenate with bag-mask, T-piece or equivalent ventilator.
Maintain oxygen saturation > 90%.

1. For endotracheal intubation

- Ketamine, IV, 1–2 mg/kg/dose.

OR

- Propofol, IV, 2.5 mg/kg. Adjust as necessary.

PLUS

If muscle relaxant necessary:

- Succinylcholine, IV, 2 mg/kg. **Causes paralysis and apnoea.**

2. During continuous mechanical ventilation

- Midazolam, IV, 1–4 mcg/kg/minute.
  - o Midazolam 3 mg/kg mixed with 50 mL dextrose 5% at 1–4 mL/hour.

PLUS

- Fentanyl, IV, 5–10 mcg/kg.
  - o If necessary, follow with IV infusion, 50 mcg/mL at 0.1–0.2 mL/kg/hour, i.e. 5–10 mcg/kg/hour.

OR

- Midazolam, IV, 1–4 mcg/kg/minute.
  - o Midazolam 3 mg/kg mixed with 50 mL dextrose 5% at 1–4 mL/hour.

PLUS

- Morphine, IV, 20–80 mcg/kg/hour.
  - o Morphine 1 mg/kg mixed with 50 mL dextrose 5% or sodium chloride 0.9% at (1–4 mL/hour).

For procedures (not ventilated)

Take standard precautions for respiratory arrest.

- Ketamine, oral, 5 mg/kg **or** IV, 1–2 mg/kg.

OR

- Midazolam, IV, 0.1–0.2 mg/kg **or** oral, 0.5 mg/kg **or** intranasal, 0.2 mg/kg. (No analgesic effect).

REFERRAL

» Inability to provide appropriate care.

21.2 PARENTERAL NUTRITION

» Parenteral nutrition (PN) is the intravenous administration of amino acids (proteins), lipids, carbohydrates, electrolytes, minerals, vitamins and trace elements necessary for metabolic requirements and growth.

» PN may be total, i.e. total parenteral nutrition (TPN) where all nutrients are administered via a central venous line until the infant is again ready to take enteral feeds.

» PN may also be partial i.e. partial parenteral nutrition (PPN) where it is used to supplement enteral feeds in infants who cannot yet tolerate their full complement of enteral feeds.

» Administer PN preferably via a central venous line, especially when it is expected that the infant will require TPN for more than 7 days. For partial parenteral nutrition a peripheral venous line may be used, especially where it is expected that the infant will require PPN for only a few days (< 7 days). Only PN solutions which contain lipids and with an osmolarity of < 1000 mOsm/L and non-lipid containing PN solutions with an osmolarity < 800 mOsm/L can be safely administered via a peripheral vein.

» Check peripheral vein infusion sites and patency of the line/catheter regularly for tissue infiltration.

» Transport and store PN solutions at 2–8ºC. Start administration of the PN solutions within one hour after removal from the refrigerator.

» Do not make additions to a PN bag or decant contents as the stability and/or the sterility may be compromised.

» Do not use the PN line to collect blood samples.

» Administer PN through a dedicated line and do not administer medications, blood, etc. through the PN line.

» Protect PN bags and lines against exposure to direct light (e.g. phototherapy). Cover the bag with a pouch and use amber lines.

» Use a 1.2 micron in-line filter for lipid containing PN solutions and a 0.2 micron filter for lipid-free PN solutions.

» Adhere to strict aseptic technique when administering PN solutions. Check solution and administration sets before starting the infusion. Use bags within 24 hours of starting infusion.

» PN should be prescribed and administered under the supervision of a paediatrician and dietician.

Complications of PN

» IV line/catheter complications e.g. extravasation, blockage or bacterial/fungal contamination;
» metabolic complications, e.g. hyperglycaemia, high ammonia, metabolic acidosis, electrolyte and mineral disturbances and hyperlipidaemia;
» infection/sepsis; and
» cholestatic hepatitis.

Monitor:

» vital signs and hydration;
» blood glucose 12 hourly, maintain blood glucose at 2.6–6 mmol/L;
» electrolytes, minerals and acid base on a daily basis or more regularly, if necessary;
» growth parameters and weight, once weekly;
» infection markers at least once weekly or more frequently, if necessary; and
» liver enzymes, bilirubin, ammonia, lipids, urea and creatinine once weekly or more frequently, as indicated by the condition of the infant.

PARENTERAL NUTRITION FORMULATIONS

Use the standard TPN formulations that are commercially available for paediatric use and that have been manufactured at a GMP (Good Manufacturing Practice) compliant site and have been regulated by the Medicines Control Council.

21.2.1 PARENTERAL NUTRITION, NEONATAL

DESCRIPTION

Parenteral nutrition (PN) should be considered within 24 hours in neonates where enteral feeds are not indicated, tolerated or contra-indicated due to medical or surgical conditions e.g. NEC, post intestinal surgery, ileus, bowel obstruction, malabsorption.

DOSE AND DURATION OF PN INFUSION

The volume of PN to be administered to the neonate depends on the age, weight and underlying disease of the neonate. Use the daily fluid requirements as a guide to determine the volume of PN solution to be administered.

The maximum volume of TPN for a neonate should not exceed 150 mL/kg/24 hours. The PN solution should be administered over 24 hours depending on the condition of the infant and volume to be administered.

The remainder of the daily fluid requirements to be made up by a neonatal maintenance solution.

Taper PN as the infant becomes able to tolerate enteral feeds.

> **Caution**
>
> **Extravasation of peripheral nutrition solutions causes severe tissue damage and necrosis.**
>
> **Do not infuse peripheral nutrition solutions into poorly running IV lines.**

	Premature newborn		Term newborn
	< 1500 g	≥ 1500 g	
The below figures are for stable full requirements after the transition phase (i.e. > day 5) – The slow increase in requirements during the transition phase is usually addressed by the incremental introduction of TPN with the parallel withdrawal of crystalloid infusion during this phase.			
Fluid mL/kg	140–180	140–160	140–170
Energy kcal/kg	110–120		90–100
Protein g/kg	1.5–4		1.5–3
CHO g/kg	6–12		10–18
Lipid g/kg	3–4		3–4

Adapted from ESPGHAN 2005.

Some infants may be intolerant to the total daily requirements of the different nutrients and may require slow up-titration.

REFERRAL

» No progress with the introduction of enteral feeds.
» Recurrent/serious complications.

21.2.2 PARENTERAL NUTRITION, PAEDIATRICS

DOSE AND DURATION OF PN INFUSION

The maximum volume of TPN for a child depends on the age, weight and underlying disease and is based on the total daily fluid requirements.

AVERAGE DAILY REQUIREMENTS

	Birth – 3 months	> 3 months –1 year	>1 – 3 years	> 3 – 6 years	> 6 – 12 years
Fluid mL/kg	120–150	120–150	80–100	80	60–80
Energy kcal/kg	90–100	90–100	75–90	75–90	60–75
Protein g/kg	1.5–3	1– 2.5	1–2.5	1– 2	1–2
CHO g/kg	18	18	14	14	12
Lipid g/kg	3–4	3–4	2–3 2–3		2–3

Adapted from ESPGHAN 2005.

AVERAGE DAILY REQUIREMENTS

The daily nutritional requirements are influenced by age, physical activity and underlying diseases/disorders e.g. burns, liver failure, etc.

REFERRAL

» No progress with the introduction of enteral feeds.
» Recurrent/serious complications.

21.3 ANAESTHETIC AND POST ANAESTHETIC CARE OF CHILDREN

21.3.1 LOCAL AND REGIONAL ANAESTHESIA

DESCRIPTION

Local anaesthesia is accomplished by either local infiltration of soft tissue or the instillation of local anaesthetic into potential or existing body spaces such as the epidural space, sub-arachnoid spinal spaces or around major nerves or plexuses.

Appropriate care is always used to limit the volumes. Use appropriate agents, avoid epinephrine (adrenaline) where end artery blood supply exists and ensure the agents are in the correct sites. This should be learnt under appropriate learning situations.

MEDICINE TREATMENT

Dental local anaesthesia

- Lidocaine (lignocaine) 2% with epinephrine (adrenaline) (1:80 000).
  - Maximum dose: 7 mg/kg lidocaine with epinephrine (adrenaline) (i.e. 0.35 mL/kg) per use.

Diffuse local soft tissue infiltration

Do not use adrenaline containing lidocaine in sites where vascular (end artery) compromise may result from vasoconstrictor use, i.e. fingers, toes, penis and eyes.

Sites where vascular (end artery) compromise is a risk.

- Lidocaine (lignocaine) 2% (**without** epinephrine (adrenaline)).
  - Maximum dose: 3 mg/kg of lidocaine (i.e. 0.15 mL/kg) per use.

Sites where vascular (end artery) compromise is not a risk:

- Lidocaine (lignocaine) 2% with epinephrine (adrenaline) (1:80 000).
  - Maximum dose: 7 mg/kg lidocaine (lignocaine) with epinephrine (adrenaline) (i.e. 0.35 mL/kg) per use.

Spinal anaesthesia

**Caution**<br>This is a specialist anaesthetic procedure.<br>Dangers include complete spinal block.

- Bupivacaine/dextrose 5/72.7 mg/mL (**without** epinephrine (adrenaline))
  - Maximum dose: 0.1–0.3 mg/kg of bupivacaine (i.e. 0.06 mL/kg). Use by an anaesthetist.

Intercostal nerve block/penile block/digital ring block/brachial (axillary approach) block

- Lidocaine (lignocaine) 2% (**without** epinephrine (adrenaline)).
  - Maximum dose: 3 mg/kg of lidocaine (i.e. 0.15 mL/kg) per use.

OR

- Bupivacaine 5 mg/mL (**without** epinephrine (adrenaline) and dextrose).
  - Maximum dose: 2 mg/kg of bupivacaine (i.e. 0.4 mL/kg).

21.3.2 GENERAL ANAESTHESIA

21.3.2.1 PREPARATION

DESCRIPTION

Premedication of children for anaesthesia is largely a sedative/anxiolytic intervention.

Recognition of the child's condition should guide in the choice of agent.

Other special medical interventions such as prevention of hypersecretion are ordered according to specific need or anticipated need.

Premedication care should be learnt in an appropriate learning situation.

Pre-operative starvation:
- » clear fluid: 2 hours,
- » breast milk: 4 hours,
- » solids, breast milk substitutes, non-human milk: 6 hours.

MEDICINE TREATMENT
Premedication:
- Midazolam, oral, 0.5 mg/kg, 15–30 minutes pre-operative.
  - o Maximum dose: 7.5 mg.

OR

For children over 2 years of age:
- Promethazine, oral, 0.5 mg/kg.
  - o Maximum dose: 25–100 mg.
  - o Decrease dose of any narcotics given.

21.3.2.2 INDUCTION

Induction should be learnt in an appropriate learning situation.

DESCRIPTION
Induction of anaesthesia is the critical part of the transition from consciousness to general anaesthesia.

All patient undergoing anaesthesia should be monitored with a minimum of:
- » clinical observation,
- » ECG,
- » blood pressure monitor,
- » pulse oximeter, and
- » temperature monitoring in children and infants.

It is desirable to do capnography for ventilated patients.

This is a period which requires highly attentive and skilled care.

MEDICINE TREATMENT
Endotracheal intubation

> **Caution**
> This procedure should be learnt under supervision.

The condition of the patient and the surgical requirements dictate airway management by way of face mask, supraglottic device or endotracheal tube.

Inhalational agents:
- Nitrous oxide.
- Oxygen.
- Halothane.

Intravenous agents:
- Propofol, IV, 2.5 mg/kg.

OR
- Ketamine, IV, 1–2 mg/kg/dose.

OR
- Thiopental sodium, IV, 2–5 mg/kg.
  - o Use smaller doses in neonates and child, higher dose in infants.

Muscle relaxant during induction for intubation:

> **Ventilate all patients receiving muscle relaxants.**

- Succinylcholine, IV, 2 mg/kg. **Causes paralysis and apnoea.**

OR
- Vecuronium bromide, IV, 0.1 mg/kg. **Causes paralysis and apnoea.**

Endotracheal tube sizes in anaesthesia (Children)				
Age	Weight (kg)	ETT	Oral (at lips)	Nasal (at nostril)
Prem	1	2.5	7	8.5
Prem	2	2.5-3	8	9.5
Term	3	3–3.5	9.5	11.5
2 months	4.5	3.5	11	12.5
1 year	10	4 12		14
18 month	12	4.5	13	15
2 years	15	5 14		16
4 years	17	5.5	15	17
6 years	21	6 16		19
8 years	25	6.5	17	20
10 years	31	7 18		21

21.3.2.3 MAINTENANCE

**Caution**
This procedure should be learnt under supervision.

DESCRIPTION

After induction transition occurs to maintenance of adequate level of pain prevention, amnesia and immobility to allow pain free and safe surgical care, i.e. adequate narcosis, analgesia and muscle relaxation.

Appropriate care is always required to monitor the patient, detect complications of, and depth of anaesthesia.

MEDICINE TREATMENT

Inhalation anaesthesia

- Oxygen.
- Nitrous oxide.
- Halothane.
- Isoflurane.

Intravenous medicine used during anaesthesia in children (not neonates):

- Fentanyl, IV, 1 –2 mcg/kg (under anaesthetist supervision up to 5–10 mcg/kg).
  - Maximum dose: 50–100 mcg if un-ventilated.

OR

- Morphine, IV, 20–80 mcg/kg/hour.
  - Morphine 1 mg/kg mixed with 50 mL dextrose 5% or sodium chloride 0.9% at (1–4 mL/hour).

Muscle relaxant during maintenance:

- Vecuronium bromide, IV.
  - Initial dose: IV bolus, 0.08–0.1 mg/kg.
  - Maintenance doses: IV, 0.03–0.05 mg/kg, as needed.

Reversal of muscle relaxant

- Neostigmine plus atropine, IV, 0.1 mL/kg of neostigmine/atropine solution below:
  - Neostigmine/atropine solution: 0.5 mL of neostigmine 2.5 mg/mL **plus** 0.5 mL atropine 0.6 mg/mL **plus** 0.5 mL sodium chloride 0.9%.

To reduce secretions:

- Atropine, IV, 0.02 mg/kg.
  - Maximum dose: 0.6 mg.

To protect the eyes from trauma and drying during anaesthesia, gently tape eyelids closed with thin light medical porous adhesive tape, or equivalent.

If eyes are required to be open:

- Hydroxypropylmethylcellulose 0.3%, ophthalmic drops.

21.3.3 POST OPERATIVE CARE

DESCRIPTION

After surgery adequate control of pain is required for comfort and also for the optimisation of outcome and minimisation of adverse effects of pain on recovery.
Pain relief should be adapted to the specific needs of each patient – according to the severity of pain, site of pain and type of pain.

GENERAL AND SUPPORTIVE MEASURES

Appropriate control of environment, provision of sedation (as above), normal physiological requirements, monitoring of vital signs and provision of comforting care should be incorporated in the care of infants and children during and after surgery.

MEDICINE TREATMENT

For pain (post operation): neonates
<u>Ventilated neonate:</u>

- Morphine, IV, 10–30 mcg/kg/hour (0.5–1 mL/hour).
    - o Morphine 1 mg/kg mixed with 50 mL dextrose 5% or sodium chloride 0.9%.

<u>Unventilated neonate:</u>

- Tilidine, oral, 1 drop per 2.5 kg (i.e.1 mg/kg/dose) 6 hourly.

Beware of respiratory depression.<br>**Monitoring of respiration is essential**.

PLUS

- Paracetamol, oral, 10–20 mg/kg as a single dose immediately.
    - o Follow with 15 mg/kg/dose 6 hourly.

PLUS

- Sucrose 24%, buccal, as needed.
    - o Preterm infants: 0.5–1 mL.
    - o Term infants: 2 mL.

For pain (post operation): older child (more than 3 months of age)

- Morphine, IV, 20–80 mcg/kg/hour
  - o Morphine 1 mg/kg mixed with 50 mL dextrose 5% or sodium chloride 0.9% at (1–4 mL/hour).

OR

- Morphine, IM/SC, 0.1 mg/kg 4–6 hourly as necessary.

OR

- Tilidine, oral, 1 drop per 2.5 kg (i.e.1 mg/kg/dose) 6 hourly. **Use with cardio-respiratory monitoring in infants.**

PLUS

- Paracetamol, oral, 20 mg/kg as a single dose immediately.
  - o Follow with 15 mg/kg/dose 6 hourly.

If oral cannot be used:

- Paracetamol rectal, 6 hourly.
  - o If 3–12 months of age: 60–125 mg.
  - o If 1–5 years of age: 125–250 mg.
  - o If 6–12 years of age: 250–500mg.

OR

- Ibuprofen, oral, 4–10 mg/kg/dose 6–8 hourly.
  Monitor renal function and ensure adequate hydration.

Regional anaesthesia

See section 21.3.1: Local and regional anaesthesia.

REFERRAL

» Inability to provide appropriate care.

21.3.4 MANAGEMENT OF ANAESTHETIC AND POSTANAESTHETIC COMPLICATIONS

DESCRIPTION

Various events may occur during and after anaesthesia which require management.

MEDICINE TREATMENT

Laryngospasm

Bag-mask ventilation and reintroduction of a volatile general anaesthetic agent may overcome laryngospasm without the need for succinylcholine.

- Succinylcholine, IV, 2 mg/kg. **Causes paralysis and apnoea.**

Bronchospasm

- Salbutamol nebulisation – administered in line (i.e. in circuit) or by mask.

See section 15.2.1: Asthma attack, acute.

OR

- Salbutamol, IV 5–10 mcg/kg/minute for 1 hour.
    - Follow with 1–2 mcg//kg/minute.

Hypersecretion

- Atropine, IV, 0.02 mg/kg.
    - Maximum dose: 0.6 mg.

Respiratory depression/apnoea from opiate overdose:

- Naloxone, IV, 0.01 mg/kg, repeated every 2 minutes, if required, up to 4 times.
    - Maximum dose: 0.4 mg.

      Note: All patients need to be kept under direct observation until the effect of the opiates has completely worn off. Further doses of naloxone may be needed as naloxone has a short duration of action.

Emergence delirium

- Midazolam, oral, 0.5 mg/kg, 15–30 minutes pre-operative.
    - Maximum dose: 7.5 mg.

Post operative nausea and vomiting

- Metoclopramide, IV/IM, 0.15–0.3 mg/kg 6 hourly.

Use with caution as extrapyramdal side effects may occur (especially at high doses)

OR

Children > 2 years of age:

- Ondansetron, slow IV, 0.2 mg/kg.
    - Maximum dose: 8 mg.

Malignant hyperthermia

- Dantrolene, IV, 1 mg/kg/minute until improvement.
    - Follow with 1–2 mg/kg 6 hourly for 1–3 days.
    - Maximum dose: 10 mg/kg.

Shock

See section 1.1.7: Shock.

Dysrhythmias

See section 4.1: Cardiac dysrhythmias.

Prevention of symptomatic hypocalcaemia during rapid large blood transfusion in babies with acid citrate anticoagulated blood:

- Calcium gluconate 10%, IV infusion.
  - 5–10 mL of calcium gluconate 10% added to 200 mL bag of compatible IV infusion fluid.
  - Infuse at maintenance IV fluid rate if blood transfusion volumes approach circulating volume of child (~80 mL/kg).

CHAPTER 22

ADOLESCENCE

Adolescence is a period of significant physical, emotional, and cognitive change and is the developmental phase of separation and individuation. The development of independence from family and the pressure to conform to peers can impose unique challenges in the management of chronic disease.

Despite the stronger identification with the peer group, adolescents with illnesses require more support from family and caregivers. Decreased focus on family and increased conformity with peer groups results in poor acceptance of a disease. Peer pressure and the need not to be stigmatised by a disease or its treatment dominates the emotional life of adolescents and may alter adherence to medicines.

Adolescence spans the period of pubertal development which manifests with physical changes which reflects the maturation of the gonads and hypothalamic pituitary gonadal axis. Generally accepted age for adolescence includes 10 to 19 years although changing views has extended the adolescent/youth period to age 24 years. Significant biologic (reflected in the different stages of puberty) and psychological changes occur during adolescence. Puberty is the time of somatic growth and sexual maturation, with females developing more fat and males more muscle mass. Irresponsible behaviour and tendency towards risk taking are features in adolescence that are related to the hormonal changes in puberty.

Distinct psychosocial features characterise early, mid- and late adolescence. Recognition of these stages impacts on issues such as adherence. In early adolescence, the individual is unable to think abstractly or plan ahead; in middle adolescence, concrete thinking in times of stress develops; in late adolescence abstract thinking and the ability to anticipate the future and plan develops.

TANNER STAGING OF PUBERTAL DEVELOPMENT

Tanner stage	Pubic hair	Breast development	Testicular and scrotal development	Penis
1.	No hair	Pre-adolescent	Pre-adolescent	Pre-adolescent
2.	Sparse, downy hair at base of symphysis pubis	Breast bud	Enlargement of scrotum and testis Skin of scrotum reddens, changes in texture	Little or no penis enlargement
3.	Sparse, coarse hair across symphysis pubis	Continued growth of breast	Further growth of testes and scrotum	Enlargement of penis mainly in length
4.	Adult hair quality, fills in pubic triangle, no spread to thighs	Areolar and papillae form secondary mound	Testes and scrotum larger; scrotal skin darkened	Increased size with growth in breadth and development of glans
5.	Adult quality and distribution of hair including spread to medial thighs	Mature female breast	Adult size and shape	Adult size and shape

Note: deviation from normal pubertal development may be primarily a disorder of the endocrine system, but more importantly, it may reflect the impact of another disease process on the endocrine system.

22.1 ADOLESCENT CHRONIC DISEASE: TRANSITION OF CARE

Z00.3

DESCRIPTION

Transition of care in adolescence is described as the purposeful, planned movement of a person with chronic medical conditions from a child-centered to an adult-orientated health care service.

Specialised programmes for transition improve adherence and outcomes, e.g. HIV, diabetes, epilepsy and rena l disease. Careful assessment of growth and dev elopment may determine an ind ividualised approach to transition.

Chronic disease during this period impacts on growth and development. HIV acquired perinatally as well as that due to unprotected sexual contact ("behaviourally infected") are encountered during adolescence.

GENERAL AND SUPPORTIVE MEASURES

» Promote adherence to medicine and follow up.
» Counselling and support.
» Manage and co-ordinate treatment through a multidisciplinary team including physicians and paediatricians.

MEDICINE TREATMENT

Titrate doses according to Tanner staging rather than strictly on b asis of age.

» Tanner 1 or 2 (early puberty): use paediatric schedules.
» Tanner stage 5 (late puberty): use adult schedules.

Puberty may be de layed in children with chronic disease, adding to discrepancies between Tanner stage-based dosing and age-based dosing.

During puberty doses may vary. Consult relevant package inserts for guidance of dosages.

Optimise therapy of certain medicines by monitoring drug levels, adjusting doses during puberty (particularly in diabetics) and with weight gain.

Be aware of medicine interactions, e.g. induction of oral contraceptive metabolism by rifampicin and recognising changes of drug disposition during puberty.

As psychological changes in puberty affect adherence, convenient medicine formulations and devices that contribute to better disease management may be considered.

Minimise the adverse impact of m edicines on cognition and brain development.

REFERRAL

» Refer patients with cognitive impairment and mental health problems to a psychiatrist.
» Adolescents with chronic disease for assessment by psychologist and mental health specialist for recognition of anxiety, depression, attention-deficit disorder and post traumatic stress disorder.

22.2 CONTRACEPTION, TEENAGE PREGNANCY AND TERATOGENICITY RISKS

Z30.9

DESCRIPTION

Adolescents are at risk for both sexually transmitted disease and unintended pregnancy. Health care workers need to be supportive of adolescents regardless of whether they are abstinent or sexually active.

The foetus in the pregnant adolescent taking medicine may be at risk for teratogenicity. Examples of potential teratogenic medicines include some members of the following classes e.g. anticonvulsants, antiretrovirals, anticoagulants, antithyroids, chemotherapy and radiation.

GENERAL AND SUPPORTIVE MEASURES

Offer counselling about sex education early in adolescence.
Counsel pregnant adolescent females about the risks of teratogenicity.
Refer pregnant adolescent for early foetal ultrasonography.

MEDICINE TREATMENT

For contraception refer to the Standard Treatment Guidelines and Essential Medicines List for Primary health Care.
Seek expert advice for pregnant teenagers on potential teratogenic medicine.

REFERRAL

» All pregnant teenagers with significant disease requiring chronic medicine.

22.3 HYPOGONADISM IN CHRONIC DISEASE

E23.0

DESCRIPTION

Hypogonadism can manifest as a complete lack of secondary sexual development or failure of normal pubertal progression. In addition to these features, females may present with menstrual irregularities.

Hypogonadism can be divided into:

» Hypogonadotrophic hypogonadism (due to hypothalamic or pituitary disorders), or
» Hypergonadotrophic hypogonadism (due primarily to abnormalities of the gonad).

DIAGNOSTIC CRITERIA

» Hypogonadotrophic hypogonadism is characterised by low levels of gonadotrophins.
  > chronic disease may be associated with hypogonadotrophic hypogonadism”. Pathogenic factors in HIV include malignancies and opportunistic infections which may lead to delayed puberty;
  > poorly controlled diabetics may also manifest with delayed puberty.

» Hypergonadotrophic hypogonadism is characterised by raised levels of gonadotrophins.

» Free testosterone and total testosterone levels may be normal as the sex hormone binding globulin levels may be elevated particularly in HIV infected patients.

» Symptoms of hypogonadism:
  > Reduced energy.
  > Low mood.
  > Reduced sense of well being.

» Objective signs of testicular failure include:
  > Low testicular volume due to testicular atrophy.
  > Gynaecomastia.

GENERAL AND SUPPORTIVE MEASURES

Reassure and support patients with constitutional delay of puberty and growth.

MEDICINE TREATMENT

The goal of therapy is the stimulation of normal pubertal progression and attainment of genetic potential for height.

Initiation of therapy should be under supervision of an endocrinologist, and maintenance therapy should be initiated by a specialist.

Males

- Testosterone cypionate, IM.
  - o Starting dose: 50 mg monthly for 6 months.
    Thereafter increase dose to 100 mg monthly for 6 months.
  - o If inadequate response continue to increase the testosterone dose by 50 mg until an adult replacement dose is achieved, i.e. 250 mg monthly.
  - o Adjusting dose intervals, if necessary.

Females

- Ethinyl oestradiol, oral.
  - o Starting dose: 2 mcg daily for 6 months.
  - o Then 5 mcg daily for 6 months; then 10 mcg daily for 6 months; then 15 mcg daily for 6 months; then 20 mcg daily for 6 months.

If there is vaginal spotting when the dose of ethinyl oestradiol is 20 mcg daily, or when the endometrial tissue is > 5 mm on pelvic ultrasound:
ADD

- Levonorgestrel, oral, 30 mcg daily from day 14 to 21 of the cycle.

> **CAUTION**:
> Women on long-term oestrogen replacement require regular breast examinations.

22.4 METABOLIC SYNDROME ASSOCIATED CARDIO-METABOLIC RISK

E88.81

DESCRIPTION

The metabolic syndrome is a cluster of cardiovascular and diabetes risk factors including abdominal (central) obesity, dyslipidaemia, glucose intolerance, and hypertension. The key factor in the pathogenesis of metabolic syndrome is insulin resistance, a situation arising in obesity. Other factors which increase cardiovascular risk of metabolic syndrome include:

» HIV,
» chronic kidney disease,
» smoking, and
» autoimmune inflammatory diseases.

DIAGNOSTIC CRITERIA

Increased body mass index (BMI) is used to classify obesity although increased waist circumference is considered to be a better sign of metabolic syndrome.
Adolescents with an increased BMI, and abnormalities in any two of the following may be deemed to have the metabolic syndrome:

» raised triglycerides > 1.69 mmol/L,
» low HDL-C,
» raised blood pressure, and
» type 2 diabetes/ finger prick fasting glucose > 5.6 mmol/L.

GENERAL MEASURES

Weight control and lifestyle modification.
Medical approach: Screening for at-risk groups to reduce the burden of disease e.g. hypertension, cardiovascular risk, and type 2 diabetes.
Public health approach: Health promotion at schools.

Targeted Lifestyle Behaviour Modification:
- » reduced intake of sugar-sweetened beverages,
- » consumption of > 5 servings of fruit and vegetables/day,
- » limit TV viewing to less than 2 hours/day,
- » advise family not to skip breakfast,
- » home prepared meals,
- » minimum of one hour of physical activity per day, and
- » regular uninterrupted sleep of at least 8 hours per day.

MEDICINE TREATMENT
If patient has risk factors and LDL > 5.4 mmol/L consider therapy.
See section 4.10: Dyslipidaemia.

REFERRAL
- » Morbid obesity not responding to life-style modification and medical therapy.
- » Development of complications e.g. obstructive sleep apnoea.

22.5 ACNE
L70.9

DESCRIPTION
Acne is a disease of sebaceous glands and is the commonest complaint affecting adolescents. Self image may be compromised by untreated acne.
See section 5.3.2: Acne.

22.6 DIABETES IN ADOLESCENCE
E10/11

DESCRIPTION
Glycaemic control deteriorates due to increase in growth hormone during puberty and decreased insulin sensitivity.

Poor metabolic control may be due to:
- » Family dynamics e.g. resistance to parental supervision (with increasing autonomy and drive for personal identity).
- » Oppositional interaction and emotional liability.
- » Risk-taking behaviour (e.g. recklessly neglecting to inject themselves and substance abuse).

Aggression and agitation may be features of poorly controlled diabetes.

Neurocognitive problems may be a consequence of hypoglycaemia. Repeated episodes of hypoglycaemia may affect spatial memory, poor cognitive abilities and delayed recall (which impact on self management).

Co-morbid psychiatric disorders and behavioural problems associated with diabetes

Adolescents with diabetes may have associated psychiatric disorders which could result in recurrent admissions.

Adjustment disorders characterised by anxiety, withdrawal, sadness, and dependency, manifest early and may become permanent if not recognised and treated.

Depressive symptoms may present atypically with vegetative symptoms such as fatigue, weight loss and impaired memory. Persistence of symptoms after achieving metabolic control should alert one to the diagnosis.

Anxiety disorders are common in diabetics and should be differentiated from hypoglycaemic and hyperglycaemic episodes.

Note: Differentiate persistent fears from physical symptoms such as palpitations and diaphoresis associated with hypoglycaemic episodes.

Eating disorders (anorexia and bulimia) risk taking behaviour and substance abuse are more common in diabetic patients.

GENERAL AND SUPPORTIVE MEASURES

Active involvement of parents in the management.

Co-morbid mental health illnesses should be treated by a psychologist.

Specific interventions such as cognitive behavioural therapy, coping skills training, and family based interventions may be necessary.

MEDICINE TREATMENT

Individualise insulin regimens to avoid hypo- and hyperglycaemia.

Insulin regimens that fit with an unrestricted life-style contribute to better control.

Insulin resistance occurs during puberty, being maximal in late puberty. Increase insulin dose in line with requirements and may reach 1.5–2.0 units/kg/day.

After puberty, the insulin requirements fall to prepubertal levels.

Failure to reduce insulin requirements in the late adolescent stages may result in excessive weight gain.

See section 7.5: Diabetes mellitus.

REFERRAL

» Uncontrolled diabetes.

22.7 OBESITY IN ADOLESCENCE

ICD10

See section 7.15: Obesity.

22.8 ANOREXIA NERVOSA, BULIMIA NERVOSA (EATING DISORDERS)

50.0/50.2/50.9

DESCRIPTION

The presence of eating disorders with pathological preoccupation with weight and body shape that are not fully accounted for by an underlying medical problem.

Early symptoms of eating disorders include:

- » growing interest in composition of food and its caloric content,
- » avoidance or skipping of main meals,
- » restriction to healthy foods, frequent weighing,
- » increased energy or physical restlessness,
- » discontent with weight and body shape,
- » increased achievement and
- » social isolation.

Eating disorders can be categorised as:

- » anorexia nervosa,
- » bulimia, and
- » eating disorders not otherwise specified.

Fear of weight gain dominates the life of adolescents with eating disorders. Self-esteem depends on maintaining slimness and the ability to control weight, and is based on their distorted body image.

In anorexia nervosa an exaggerated wish for thinness leads to weight loss. Anorexia nervosa can have serious medical consequences, including death. Untreated anorexia may be associated with delayed sexual maturation, regression of breast tissue and stunting.

Bulimia is a condition of self-induced vomiting after each eating episode. Recurrent vomiting (in the purging subtype) can result in Mallory-Weiss tears.

The eating disorders can impact significantly on physical, emotional and social aspects and may be associated with co-morbidity (endocrinopathies including amenorrhoea, and osteopaenia).

Patients may not seek advice for their eating disorder but may complain of hair loss, brittle nails, constipation, headache or fatigue.

REFERRAL

- » Urgently admit patients who develop oedema, cardiovascular signs, or who are purging.
- » All suspected patients must be routinely referred to a multidisciplinary team including physicians, endocrinologists, social workers, psychologists, and psychiatrists.

CHAPTER 23

DRUG ALLERGIES

23.1 DRUG ALLERGIES

T88.7

DESCRIPTION

Drug allergy is an immune-mediated reaction to the drug. Common drugs involved include penicillin, sulphonamides, and non-steroidal anti-inflammatory drugs.

Classification

Drug hypersensitivity reactions are classified as:

» immediate (≤ 1 hour after exposure): anaphylaxis, urticaria, angioedema; or
» delayed (≥ 6 hours): often involving rash with or without systemic symptoms.

DIAGNOSIS

Drug allergies are diagnosed clinically, based on symptoms and signs, and their timing relative to drug exposure; as well as exclusion of other potential causes.

In the acute setting, do laboratory tests based on the patient's condition to exclude, or to determine the severity of systemic involvement (for example full blood count or liver function tests).

Non-specific tests of drug allergy

An elevated serum tryptase concentration might help to confirm the diagnosis of anaphylaxis, but a normal concentration does not exclude it. If the diagnosis of anaphylaxis is unclear, take blood 1–4 hours after the onset of symptoms, and a follow-up sample after at least 2 days for comparison.

Identification of causative drug

Do tests to confirm the causative drug only if the benefit to the patient outweighs the risk, and only in consultation with a specialist.

» Skin tests: subcutaneous skin prick tests, intradermal tests, and patch tests:
  > Have variable sensitivity and specificity depending on the drug.
  > Perform skin and patch tests only in specialised units. Safety equipment must be available as they can provoke significant reactions.
» Specific IgE against suspected drug:
  > Available for a few drugs only.
  > Has low sensitivity but high specificity.

» Oral drug provocation tests:
  > Can be done for most drugs and are the gold standard proof of tolerance or allergy.
  > Perform only in specialised units. Safety equipment must be available as they can provoke significant reactions.

23.2 IMMEDIATE HYPERSENSITIVITY REACTIONS

23.2.1 DRUG RELATED ANAPHYLAXIS

T88.6

See Section 1.1.3: Anaphylaxis/anaphylactic reactions.

23.2.2 DRUG RELATED URTICARIA

L50.9

See Section 5.3.7: Urticaria.

23.2.3 DRUG RELATED ANGIOEDEMA

T78.3

DESCRIPTION

Local swelling of skin and/or mucosal tissue. May occur on its own or with urticaria or anaphylaxis. It must be distinguished from recurrent non pruritic angioedema which has a hereditary component and does not respond to the treatment below. Complement C4 and C1 esterase inhibitor levels are used to help to distinguish the two entities.

GENERAL AND SUPPORTIVE MEASURES

» Stop potentially causative drug(s).
» Monitor airway closely and intubate early if necessary.

MEDICINE TREATMENT

If symptoms and signs of anaphylaxis: treat as for anaphylaxis (above).

If angioedema in isolation:

- Chlorphenamine, oral, 0.1 mg/kg/dose 6 hourly.

- Prednisone, oral, 1–2 mg/kg daily for 1 week.

REFERRAL

» All cases after stabilisation for confirmation of diagnosis and long term management.
» Recurrent non pruritic angioedema.

23.3 DELAYED HYPERSENSITIVITY REACTIONS

See sections 5.3.1: Drug reactions and 5.2.1 Erythema multiforme/Stevens-Johnson Syndrome.

DESCRIPTION

Broad spectrum of clinical manifestations ranging from maculopapular or morbilliform rashes (most common presentation), to life-threatening cutaneous reactions such as Stevens-Johnson Syndrome/toxic epidermal necrolysis (SJS/TEN). Common drugs associated are antiretrovirals (efavirenz or nev irapine), anticonvulsants, anti-tuberculous therapy, penicillins and co-trimoxazole.

GENERAL AND SUPPORTIVE MEASURES

Stop the suspected causative medicine immediately. Give an a lternate agent.
If there are compelling reasons to continue with the suspected medicine, seek expert advice.

MEDICINE TREATMENT

Mild reactions without systemic or mucosal involvement may be treated symptomatically:
- Chlorphenamine, oral, 0.1 mg/kg/dose 6 hourly.

REFERRAL

» SJS/TEN for management in a centre with experience in treating burns.

23.4 SPECIFIC ALLERGIES

23.4.1 ALLERGIES TO PENICILLINS

Z88.0

DESCRIPTION

Patients may present with immediate (e.g. anaphylaxis, bronchospasm, angioedema) or delayed reactions (most commonly maculopapular rash without systemic involvement; rarely SJS/TEN or other systemic reactions).

GENERAL AND SUPPORTIVE MEASURES

Stop penicillin.

MEDICINE TREATMENT

If an antibiotic is still required, treat with a suitable antibiotic according to the condition.

Milder infections e.g. upper respiratory tract infections, impetigo, mild cellulitis:

- Macrolide, e.g.:
- Erythromycin, oral, 15 mg/kg/dose 6 hourly for 10 days.

Severe infections, e.g. osteomyelitis, pneumonia:

- Third generation cephalosporin, provided there is no history of immediate hypersensitivity (see below–Cross-reactivity of other β-lactams).

Alternative antibiotics for Gram positive infections:

- Clindamycin, oral, 6 mg/kg/dose 6 hourly.

OR

- Vancomycin, IV, 15 mg/kg 8 hourly.

Prophylaxis in acute rheumatic disease or post splenectomy for idiopathic thrombocytopaenic purpura, consider**:**

- Macrolide e.g.:
- Erythromycin, oral, 10 mg/kg/dose 12 hourly for life.

Cross-reactivity of other β-lactams in patients with penicillin allergy

Avoid cephalosporins in patients with a history of an immediate reaction to penicillin (anaphylaxis, urticaria, or angioedema).
In patients with a history of rash only, there is about a 5% risk of rash with cephalosporins, but no increased risk of anaphylaxis.
The risk of cross reactivity is lower with third generation cephalosporins such as ceftriaxone, which may be used if there are no suitable alternatives.
Risk of cross-reactivity is very low with carbapenems.

If no alternative antibiotic is available, consider desensitisation after consultation with a specialist.

Perform only in an ICU setting.<br>**A history of Stevens-Johnson syndrome, exfoliative dermatitis, or erythroderma is an absolute contra-indication to desensitisation.**

Discontinue all ß-adrenergic antagonists.
Ensure adequate IV access.
Monitor the patient with ECG and spirometry.
Oral route is preferred. 1/3 of patients develop a transient reaction during desensitisation or treatment, which is usually mild.

<table>
<tr><td colspan="3">A: Reconstitute phenoxymethylpenicillin 250 mg/5 mL</td></tr>
<tr><th>Step</th><th>Drug mg/mL</th><th>Amount to administer</th></tr>
<tr><td>Strictly every 15 minutes</td><td colspan="2">B: To make 0.5 mg/mL solution
Dilute 0.5 mL of reconstituted phenoxymethylpenicillin solution in 49.5 mL water.</td></tr>
<tr><td>1</td><td rowspan="7">0.5 mg/mL solution</td><td>0.1 mL</td></tr>
<tr><td>2</td><td>0.2 mL</td></tr>
<tr><td>3</td><td>0.4 mL</td></tr>
<tr><td>4</td><td>0.8 mL</td></tr>
<tr><td>5</td><td>1.6 mL</td></tr>
<tr><td>6</td><td>3.2 mL</td></tr>
<tr><td>7</td><td>6.4 mL</td></tr>
<tr><td></td><td colspan="2">C: To make 5 mg/mL solution
Dilute 1 mL of reconstituted phenoxymethylpenicillin solution in 9 mL water</td></tr>
<tr><td>8</td><td rowspan="3">5 mg/mL solution</td><td>1.2 mL</td></tr>
<tr><td>9</td><td>2.4 mL</td></tr>
<tr><td>10</td><td>4.8 mL</td></tr>
<tr><td></td><td colspan="2">D: Reconstituted phenoxymethylpenicillin
250 mg/5mL = 50 mg/mL</td></tr>
<tr><td>11</td><td rowspan="4">50 mg/mL</td><td>1.0 mL</td></tr>
<tr><td>12</td><td>2.0 mL</td></tr>
<tr><td>13</td><td>4.0 mL</td></tr>
<tr><td>14</td><td>8.0 mL</td></tr>
</table>

After the recommended treatment dose per kg is reached, observe for 30 minutes, then give the recommended dose, IV.

Note: Once desensitised, treatment must not lapse as risk of subsequent allergy increases.

REFERRAL

Consult a specialist:

» For alternative antibiotics in all patients with immediate reactions
» In cases where desensitisation is considered.

23.4.2 ALLERGIES TO SULPHONAMIDES

Z88.2

DESCRIPTION

The commonest sulphonamide allergies are related to co-trimoxazole, especially when used for *P. jirovecii* treatment and prophylaxis.

Patients may present with:

» a morbilliform or maculopapular rash only, usually within a few days of starting treatment (most common presentation),
» a rash with fever,
» with/without organ dysfunction, usually within 1–2 weeks,
» with SJS/TEN, or
» rarely with an immediate hypersensitivity reaction.

GENERAL AND SUPPORTIVE MEASURES

Stop the sulphonamide-containing drug.

MEDICINE TREATMENT

For *P. jirovecii* pneumonia prophylaxis in patients with history of rash:

- Dapsone, oral, 2 mg/kg daily.
  - o Maximum dose: 100 mg (1 tablet) daily.
  - o Note: Dapsone is a sulphone, not a sulphonamide, but there are concerns regarding cross-reactivity with sulphonamide allergy. Avoid dapsone if there is a history of anaphylaxis, SJS/TEN, or rash with systemic involvement.

If no alternative is available, consider desensitisation, as an inpatient, in patients with a history of rash, with or without fever. Consult a specialist for desensitisation of patients with a history of anaphylaxis.

Perform only in an ICU or high care setting

Discontinue all ß-adrenergic antagonists.
Ensure adequate IV access and immediate availability of all drugs for treatment of anaphylaxis.
Monitor the patient with ECG (looking for arrhythmias) and spirometry (looking for a drop in FEV_1).

Desensitisation is contra-indicated in patients with a history of SJS/TEN.

Dilute 1 mL of co-trimoxazole suspension (40/200 mg/5 mL) in 19 mL sodium chloride 0.9% or dextrose 5% solution.
1 mL dilute (1:20) solution = 0.4/2 mg co-trimoxazole

Day	Dose	Co-trimoxazole, administer orally
1	0.4/2 mg	1 mL of 1:20 solution
2	0.8/4 mg	2 mL of 1:20 solution
3	1.6/8 mg	4 mL of 1:20 solution
4	3.2/16 mg	8 mL of 1:20 solution
5	8/40 mg	1 mL of 40/200 mg/5 mL suspension
6	16/80mg	2 mL of 40/200 mg/5 mL suspension
7	32/160 mg	4 mL of 4/200 mg/5 mL suspension
8	64/320 mg	8 mL of 40/200 mg/5 mL suspension
9	80/400 mg	1 tablet (80/400 mg)
10	160/800 mg	1 tablet (160/800 mg)

After the recommended dose per kg is reached, continue with daily dosing at that dose.
Note: Once desensitised, treatment must not lapse as risk of subsequent allergy increases.

If patients require urgent co-trimoxazole treatment, a more rapid protocol may be used, stopping when the recommended dose per kg is reached:

Dilute 0.1 mL of co-trimoxazole suspension (40/200 mg/5 mL) in 200 mL sodium chloride 0.9% or dextrose 5% solution.
1 mL dilute solution = 0.004/0.02 mg co-trimoxazole

Hour	Dose	Co-trimoxazole, administer orally
0	0.004/0.02 mg	1 mL of dilute solution
1	0.04/0.2 mg	10 mL of dilute solution
2	0.4/2 mg	0.05 mL of syrup or 100 mL of dilute solution
3	4/20 mg	0.5 mL of syrup
4	40/200 mg	5 mL of syrup or ½ tablet
5	160/800 mg	2 single strength or 1 double strength tablet

Note:
Do **not** give antihistamines with this regimen.

REFERRAL

» Consult a specialist for alternative medicines in patients with immediate reactions or SJS/TEN.

GUIDELINES FOR THE MOTIVATION OF A NEW MEDICINE ON THE NATIONAL ESSENTIAL MEDICINES LIST

Section 1: Medication details

» Generic name
A fundamental principle of the Essential Drug Programme is that of generic prescribing. Most clinical trails are conducted using the generic name.

» Proposed indication
There will usually be many registered indications for the medication. However, this section should be limited to the main indication which is supported by the evidence provided in section 2.

» Prevalence of the condition in South Africa
This information is not always readily available. However, it is an important consideration in the review of a proposed essential medicine.

» Prescriber level
Here the proposed prescriber level should be included. If more than one level is proposed each relevant box should be ticked.

Section 2: Evidence and motivation

» Estimated benefit
- Effect measure: this is the clinical outcome that was reported in the clinical trial such as BP, FEV, CD_4, VL etc.
- Risk benefit: this should reported in the clinical trial and, in most cases, includes the 95% confidence level (95% CI). Absolute risk reduction, also termed risk difference, is the difference between the absolute risk of an event in the intervention group and the absolute risk in the control group.
- Number Need to Treat (NNT): gives the number of patients who need to be treated for a certain period of time to prevent one event. It is the reciprocal of the absolute risk or can be calculated using the formula below.

Calculations

	**Bad outcome**	**Good outcome**	**Total patients**
Intervention group	*a*	*c*	*a + c*
Control group	*b*	*d*	*b + d*

**Measure**	**Equation**
Absolute risk:	[b/(b+d)] – [a/(a+c)]
Number needed to treat	$\frac{1}{[b/(b+d)] - [a/(a+c)]}$
Relative risk	[a/(a+c)] ÷ [b/(b+d)]
Odds ratio	$\frac{[a/(a+c)] \div [c/(a+c)]}{[b/(b+d)] \div [d/(b+d)]} = (a/c) \div (b/d)$

Reference - Aust Prescr 2008;31:12–16)

» Motivating information (**Level of evidence based on the SORT system**)

– The National Essential Drug List Committee has endorsed the adoption of the SORT system for categorising levels of evidence. This system[1] contains only three levels:

Level I	Good quality evidence	Systematic review of RCTs with consistent findings<br>High quality individual RCT
Level II	Limited quality patient orientated evidence	Systematic review of lower quality studies or studies with inconsistent findings<br>Low quality clinical trial<br>Cohort studies<br>Case-control studies
Level III	Other	Consensus guidelines, extrapolations from bench research, usual practice, opinion, disease-oriented evidence (intermediate or physiologic outcomes only), or case series

A: Newer product: for most newer products, level 1 evidence such as high quality systematic reviews or peer-reviewed high quality randomised controlled trials should be identified and referenced in the space provided.
B: Older products: many of these products were developed prior to the wide use of randomised controlled trials. However, there maybe level 1 evidence where the product was used as the control arm for a newer product. If no level 1 evidence can be identified, then level II data from poorer quality controlled trials or high quality observational studies should be referenced in the space provided.

» Cost considerations

– Where a published reference supporting the review of cost is available comments should be made regarding its applicability to the South African public sector environment.
– Possible unpublished information that can be included:
  - o Cost per daily dose or course of therapy – for long term or chronic therapy such as hypertension the usual daily dose should be calculated (Dose x number of times a day) and converted into the number of dosing units e.g. tablets. This is then used to calculate the cost per day. For medications used in a course of therapy such as antibiotics this is then multiplied by the number of days in the course of therapy.
  - o Cost minimisation is used where there is evidence to support equivalence and aims to identify the least costly treatment by identifying all the relevant costs associated with the treatment.

[1] Ebell MH, Siwek J, Weiss BD, et al. Strength of recommendation taxonomy (SORT): a patient-centered approach to grading evidence in the medical literature. Am Fam Physician. 2004;69:550-6.

- Cost-effectiveness analysis is used to compare treatment alternatives that differ in the degree of success in terms of the therapeutic or clinical outcome. By calculating a summary measurement of efficiency (a cost-effectiveness ratio), alternatives with different costs, efficacy rates, and safety rates can be fairly compared along a level playing field.

Where any of these have been performed tick the relevant block and send as an attachment with all the calculations. If possible, the spread sheet should be supplied electronically.

Section 3: Motivator's Details

The receipt of all submission will be acknowledged. In addition, all decisions with supporting arguments will be communicated where appropriate. This section therefore forms a vital link between the motivator and the decision making process.

Motivation form for the inclusion of a new medication on the National Essential Medicines List

**Section 1: Medication details**			
Generic name (or International Nonproprietary Name):			
Proposed indication:			
Prevalence of condition (based on epidemiological data, if any):			
Prescriber level			
Primary Health Care<br>1	Medical Officer<br>2	Specialist<br>3	Designated Specialist<br>4

**Section 2: Evidence and motivation**		
**2.1 Estimated benefit**		
Effect measure		
Risk difference (95% CI)		
NNT		
**2.2: Motivating information (Level of evidence based on the SORT system)**		
**A. Newer product**: High quality systematic reviews or peer-reviewed high quality randomised controlled trials (Level I)		
Author	Title	Journal ref
**B. Older product with weaker evidence base**: Poorer quality controlled trials or high quality observational studies (Level II)		
Author	Title	Journal ref

**2.3: Cost-considerations**			
Have you worked up the cost?	YES		NO
	Daily cost	Cost minimisation	Cost-effectiveness analysis
Other relevant cost information if available:			

Author	Title	Journal ref
**2.4: Additional motivating comments.**		

**Section 3: Motivator's Details**	
**PTC Title:**	**Date submitted:**

GUIDELINES FOR ADVERSE DRUG REACTION REPORTING

National Pharmacovigilance Programme

The Medicines Control Council (MCC) has a responsibility to ensure the safety, efficacy and quality of all medicines used by the South African public. The National Pharmacovigilance Programme is coordinated by the MCC and has a dedicated Unit, The National Adverse Drug Event Monitoring Centre (NADEMC), in Cape Town, which monitors the safety of all registered medicines in South Africa.

What is Pharmacovigilance?

Pharmacovigilance is defined as the science and activities concerned with the detection, assessment, understanding and prevention of adverse reactions to medicines (i.e. adverse drug reactions or ADRs). The ultimate goal of this activity is to improve the safe and rational use of medicines, thereby improving patient care and public health.

What is an Adverse Drug Reaction (ADR)?

The Medicines Control Council (MCC) defines an Adverse Drug Reaction (ADR) as a response to a medicine which is noxious and unintended, including lack of efficacy, and which occurs at any dosage and can also result from overdose, misuse or abuse of a medicine.

Who should report Adverse Drug Reactions?

All health care workers, including doctors, dentists, pharmacists, nurses and other health professionals are encouraged to report all suspected adverse reactions to medicines (including vaccines, X-ray contrast media, traditional and herbal remedies), especially when the reaction is not in the package insert, potentially serious or clinically significant.

What happens to a report?

All ADR reports are entered into a national ADR database. Each report is evaluated to assess the causal relationship between the event and the medicine. A well-completed adverse drug reaction/product quality form submitted could result in any of the following:

» additional investigations into the use of the medicine in South Africa;
» educational initiatives to improve the safe use of the medicine;
» appropriate package insert changes to include the potential for the reaction, and
» changes in the scheduling or manufacture of the medicine to make it safer.

The purpose of ADR reporting is to reduce the risks associated with the use of medicines and to ultimately improve patient care.

Will reporting have any negative consequences on the health worker or the patient?

An adverse drug reaction report does not constitute an admission of liability or that the health professional contributed to the event in any way. The outcome of a report, together with any important or relevant information relating to the reaction, will be sent back to the reporter as appropriate. The details of a report are stored in a confidential database. The names of the reporter or any other health professionals named on a report and that of the patient will be removed before any details about a specific adverse drug reaction are used or communicated to others. The information is only meant to improve the understanding of the medicines used in the country.

Is the event possibly an ADR?

The following factors should be considered when an adverse drug reaction is suspected:

1. What exactly is the nature of the reaction? (*Describe the reaction as clearly as possible and where possible provide an accurate diagnosis.*)
2. Did the reaction occur within a reasonable time relationship to starting treatment with the suspected medicine? *(Some reactions occur immediately after administration of a medicine while others take time to develop.)*
3. Is the reaction known to occur with the particular medicine as stated in the package insert or other reference? *(If the reaction is not documented in the package insert, it does not mean that the reaction cannot occur with that particular medicine.)*
4. Did the patient recover when the suspected medicine was stopped? *(Some reactions can cause permanent damage, but most reactions are reversible if the medication is stopped.)*
5. Did the patient take the medicine again after the reaction abated (i.e. rechallenge). If so, did the same reaction occur again? *(In most situations it is not possible or ethical to rechallenge the patient with the same medicine. If such information is available or if such a rechallenge is necessary, recurrence of the event is a strong indicator that the medicine may be responsible.)*
6. Can this reaction be explained by other causes (e.g. underlying disease/s; other medicine/s; toxins or foods)? *(It is essential that the patient is thoroughly investigated to decide what the actual cause of any new medical problem is. A medicine-related cause should be considered, when other causes do not explain the patient's condition.)*

What types of reactions should be reported?

The following adverse drug reactions should be reported:

- All ADRs to newly marketed drugs or new drugs added to the EDL
- All serious reactions and interactions
- ADRs that are not clearly stated in the package insert.
- All adverse reactions or poisonings to traditional or herbal remedies

Report even if you are not certain that the medicine caused the event.

What Product Quality Problems should be reported?

The following product quality problems should be reported:

- suspected contamination;
- questionable stability;
- defective components;
- poor packaging or labeling;
- therapeutic failures.

How can ADRs be prevented from occurring?

Some ADRs are unavoidable and cannot be prevented. However, most ADRs can be prevented by following the basic principles of rational use of medicines.

How are adverse drug reactions reported?

An Adverse Drug Reaction/Product Quality Report Form is enclosed in this book and should be completed in as much detail as possible before returning it by fax or post to any of the addresses provided below. Additional forms can be obtained by contacting the MCC at these addresses. Report forms may also be accessed via the following website: *http://www.mccza.com*

1. The Registrar of Medicines

Medicines Control Council, Department of Health, Private Bag X828

Pretoria, 0001

Tel: (021) 395 8003/8176; Fax: (012) 395 8468

2. The National Adverse Drug Event Monitoring Centre (NADEMC)

C/o Division of Pharmacology, University of Cape Town,

Observatory, 7925

(021) 447 1618; Fax: (021) 448 6181

ADVERSE DRUG REACTION AND PRODUCT QUALITY PROBLEM REPORT FORM

(*Identities of reporter and patient will remain strictly confidential)*

health<br>Department:<br>Health<br>REPUBLIC OF SOUTH AFRICA	**NATIONAL ADVERSE DRUG EVENT MONITORING CENTRE NADEMC**<br>The Registrar of Medicines<br>Private Bag X 828<br>Pretoria 0001<br>In collaboration with the WHO International Drug Monitoring Programme

PATIENT INFORMATION

Name (or initials): ....

Patient Reference Number: ……………………………………………..

**Sex:**	**M**	**F**	**Age:**	**DOB:**	**Weight (kg)**	**Height (cm)**
			....	..... / ....../	....	....

ADVERSE REACTION/PRODUCT QUALITY PROBLEM (tick appropriate box)

**Adverse reaction**	☐	**and/or Product Quality problem**	☐	**Date of onset of reaction:**<br>........./........../............<br>**Time of onset of reaction:**<br>…........hour............min

Description of reaction or problem (Include relevant tests/lab data, including dates):

1. MEDICINES / VACCINES / DEVICES (include all concomitant medicines)

Trade Name and Batch No. (Asterisk Suspected Product)	Daily Dosage	Route	Date Started	Date Stopped	Reasons for use

ADVERSE REACTION OUTCOME (Check all that apply)

	death		life-threatening
	disability		hospitalisation
	congenital anomaly		Other...............
	required intervention to prevent permanent impairment/damage		

Reaction abated after stopping medicine:

	**Y**		**N**

Event reappeared on rechallenge:

	**Y**		**N**		**Rechallenge not done**

Recovered:

	**Y**		**N**

Sequelae:

	**Y**		**N**

Describe Sequelae:......................

COMMENTS: (e.g. Relevant history, Allergies, Previous exposure, Baseline test results/lab data)

2. PRODUCT QUALITY PROBLEM:

Trade Name	Batch No	Registration No	Dosage form & strength	Expiry Date	Size/Type of container

Product available for evaluation?: Y N

REPORTING HEALTHCARE PROFESSIONAL:

NAME: ....

QUALIFICATIONS:...

ADDRESS: ....

....Postal Code: ……………

TEL: (............)..

………………………….. …………………..
Signature Date

This report does not constitute an admission that medical personnel or the product caused or contributed to the event.

ADVICE ABOUT VOLUNTARY REPORTING

Report adverse experiences with:
- medications (drugs, vaccines and biologicals)
- medical devices (including in-vitro diagnostics)
- complementary / alternative medicines (including traditional, herbal remedies, etc)

Please report especially:
- adverse drug reactions to newly marketed products
- serious reactions and interactions with all products
- adverse drug reactions which are not clearly reflected in the package insert.

Report Product Quality Problems such as:
- suspected contamination
- questionable stability
- defective components
- poor packaging or labelling
- therapeutic failures

Report even if:
- you're not certain the product caused the event
- you don't have all the details

Important numbers:

Investigational Products and Product Quality Problems:
- fax: (012) 395-9201
- phone: (012) 395-9341

Adverse Events Following Immunisation:
- fax: (012) 395 8905
- phone: (012) 395 8914/5

Confidentiality: Identities of the reporter and patient will remain strictly confidential.

Your support of the Medicine Control Council's adverse drug reaction monitoring programme is much appreciated. Information supplied by you will contribute to the improvement of medicine safety and therapy in South Africa.

PLEASE USE ADDRESS PROVIDED BELOW - JUST FOLD IN THIRDS, TAPE and MAIL

Postage will be paid by the Addressee
Posgeld sal deur die geadresseerde betaal word

No Postage stamp necessary if posted in the Republic of South Africa
Geen posseël nodig nie indien in die Republiek van Suid-Afrika gepos

BUSINESS REPLY SERVICE
BESIGHEIDSANTWOORDDIENS
Free Mail Number: **BNT 178**
Vryposnommer:

DEPARTMENT OF HEALTH
DEPARTEMENT VAN GESONDHEID
REGISTRAR OF MEDICINES
REGISTRATEUR VAN MEDISYNE
PRIVATE BAG / *PRIVAATSAK* X828
PRETORIA
0001

DISEASE NOTIFICATION PROCEDURES

The disease reporting system in South Africa is based on government law (the Health Act, Act No. 61 of 2003), together with regulations on the reporting of specific diseases to the Local, Provincial and/or National Health Department.

Who should notify

The first health care professional to come into contact with a patient presenting with one of the prescribed Notifiable Medical Conditions is required by law to notify. This may include clinic personnel, infection control nurses, other hospital staff or private medical practitioners. In the event of deaths (or cases) in the community, a member of the community is obliged to notify the event.

Which diseases to notify

Currently 33 broad medical conditions are currently notifiable in South Africa (see List of Notifiable Medical Condition). Some conditions (e.g. tuberculosis and viral hepatitis) have been divided into various components, resulting in more than 40 notifiable medical conditions.

Notifiable medical conditions have been sub-divided into two categories according to type of disease:

a) **Category A:** these are medical conditions that require immediate notification to the regional/provincial or national Department of Health by telephone or fax upon initial diagnosis (presumptive or confirmed) with written notification form (GW17/5) to follow within five days.

Any health care professional identifying even a single case of a disease (presumptive or laboratory confirmed) contained in the Category A should make an immediate notification directly to the designated local health officer through fax or telephonically as rapidly as possible (within 24 hours). The local health officer must report to the Provincial health officer and/or to the National Department of Health. Where it is applicable, laboratory confirmation should be obtained at the earliest opportunity and also reported to the designated health

office. After reporting through a telephone/fax, it is still required of the health care provider to send a complete GW17/5 form to the designated local health authority within five days after telephonic reporting.

b) **Category B**: these are medical conditions that require written notification (GW17/5 form) only, within seven days of diagnosis.

The notification system is based on clinical notifications and, therefore, all suspected cases of a notifiable condition must be notified immediately.

Reporting a Notifiable Disease during an outbreak

During an outbreak of a notifiable disease, report all cases by phone, email or fax. Daily reporting by health facilities should be maintained through an Outbreak Case Line Listing Form as well through the notification form (GW17/5) to the local health authority that must report to the provincial health officials and the National Department of Health.

Priority Reporting of MDR & XDR-TB

Tuberculosis (TB) is one of 33 medical conditions, which is notifiable in terms of the National Health Act (Act 61 of 2003). The Directorate: Epidemiology and Surveillance have instituted a priority reporting for MDR and XDR TB. This means that all health care facilities, public and private, including clinics, hospitals, laboratories, general practitioners and private specialist doctors, are required to report all cases of MDR and XDR TB to the Department of Health within 24 hours.

How to notify

The initial notification of a medical condition is done on a case-based form (*GW 17/5*) with the relevant details by the health personnel e.g., clinic personnel, infection control nurses, other hospital staff, public or private medical practitioners. Initial notification makes tracing as easy as possible, since a disease notification demands action (follow-up) at the peripheral level.

The GW17/5 form makes provision for the notification of cases as well as deaths. Any person contracting a notifiable disease and then dies from the disease should be notified twice: first as a **“CASE”** and then later as a **“DEATH”.** This will ensure that when estimating the **“Case Fatality Rate”** (CFR%), all deaths in the numerator are also included in the denominator. Depending on the structural organization of the province, the completed **GW 17/5** forms is sent to the relevant local health authority, district health office or the provincial office.

National Department of Health
Cluster: Health Information, Evaluation & Research (HIER)
Directorate: Epidemiology & Surveillance
Private Bag X828
PRETORIA
0001
Tel: 012 395 8150/1

List of Notifiable Medical Conditions

Category A: *Immediate notification (within 24 hours) of diagnosis by the health care professional (telephone or fax) to the designated district or provincial health officer.*

Acute flaccid paralysis
Anthrax
Cholera
Crimean-Congo haemorrhagic fever
Other haemorrhagic fevers of Africa
Food poisoning
Measles
Meningococcal infection
Plague
Rabies, human
Yellow fever

Category B

Brucellosis
Congenital syphilis
Diphtheria
Haemophilus Influenza type B
Lead poisoning
Legionellosis
Leprosy
Malaria
Paratyphoid fever
Poisoning agricultural stock remedies
Poliomyelitis
Rheumatic fever
Tetanus
Tetanus neonatorum
Trachoma
Tuberculosis primary
Tuberculosis pulmonary
Tuberculosis of other respiratory organs
Tuberculosis of meninges
Tuberculosis of intestines, peritoneum
Tuberculosis of bones and joints
Tuberculosis of genito-urinary system
Tuberculosis of other organs
Tuberculosis miliary
Typhoid fever
Typhus fever (lice-borne)
Typhus fever (ratflea-borne)
Viral hepatitis type A (acute)
Viral hepatitis type B (acute)
Viral hepatitis non-A non-B (acute)
Viral hepatitis unspecified
Whooping cough

USING THE ROAD TO HEALTH BOOKLET

Check and update the Road to Health booklet at each consultation and on each admission and discharge.

The South African Road to Health Booklet is an extremely important document for the child and family,
It is designed to support and integrate the variouschild health strategies such as IMCI, EPI, TB and HIV care and the Integrated Nutrition Programme. It reminds health care workers to look for, respond to, and record important events and care given to the child.

OWNERSHIP OF THE BOOKLET
The Road to Health Booklet is the exclusive property of the parent (primary caregiver) and the child. This is important as the booklet contains information on the child's health including HIV status, and if the booklet is used for other purposes, mothers may hide the booklet or refuse to allow important information to be recorded in it. This can result in the child receiving less than optimal care

USE OF THE ROAD TO HEALTH BOOKLET
Issuing the Road to Health Booklet
At birth all children should be issued with a Road to Health Booklet – in which all vital information is recorded including:

» Name and date of birth – Page 1 (front cover)
» Details of child and family – Page 4
» Neonatal information – Page 5
» Immunisations at birth – Page 6
» PMTCT/HIV information – Page 7

Use at health service contacts

On the cover the booklet states:

> "IMPORTANT: always bring this booklet when you visit any health clinic, doctor or hospital"

To use the booklet effectively the attending nurse or doctor should ask, at each attendance, to see the Road to Health booklet both due to its intrinsic value as part of a child health consultation and to emphasise the importance of the booklet and its use to the mother.

On each visit complete/record appropriately

- » Well child visit routine care (incl. growth, TB status, PMTCT HIV status, feeding etc) – Pages 2 and 3.
- » Immunisations given – Page 6.
- » Information on the HIV status of the mother and child (if HIV-exposed) – Page 8.
- » Vitamin A and deworming – Page 9.
- » Weight for age, length/height for age and weight for length/height charting – Pages 14–19.
- » Any clinical notes (ideally using IMCI classification, treatment and follow up should be made in the clinical notes) – Pages 21–27.
- » Any hospital admissions should be recorded – Page 19.

During the health visit certain care given will depend on whether this is a scheduled well child visit, a follow-up visit, or a first attendance for a new illness.

<table>
<tr><th>Well child visit</th><th>Sick child consultation</th><th>Follow up consultation</th></tr>
<tr><td colspan="3">Greet mother and child</td></tr>
<tr><td>Ask why she has come and whether she has any concerns.</td><td>Ask why she has come and what her concerns are.</td><td>Ask how the child is and whether any further concerns have arisen.</td></tr>
<tr><td colspan="3">Ask for Road to Health Booklet and use it.</td></tr>
<tr><td>If the child has an illness, proceed to sick child consultation (IMCI) in addition to the well child consultation.</td><td>Proceed to sick child consultation (IMCI).
Ensure that promotive aspects of IMCI (nutrition, immunisations, HIV and TB status) are covered.</td><td>Carry out the follow-up process from IMCI, but also check the well child consultation.</td></tr>
<tr><td colspan="3">Check and record all due visit items – see above.</td></tr>
<tr><td>Carry out and record the well child visit.
Note and respond to any other problems identified.</td><td colspan="2">Manage the child according to IMCI classification.
Follow up as required.
Carry out and record the well child visit.
Note and respond to any other problems identified.</td></tr>
<tr><td colspan="3">Tell mother what has been done, what was found and what this means.
Ensure the mother knows when to follow up for the next well child visit, and when to come if the child is ill or for other scheduled follow up.</td></tr>
</table>

Boy's Weight-for-Age Chart

Write on the chart

- Any illness e.g. diarrhoea, ARI etc.
- Admission to hospital
- Solids introduced
- Breastfeeding stopped
- Birth of next child, etc

Like this:

ARI | Admitted to hospital Diarrhoea | Extra meals given | Worm medicine

Watch the direction of the curve showing the child's growth:

GOOD
Means the child is growing well

VERY DANGEROUS
Child may be ill needs extra care

DANGER SIGN
Not gaining weight
Find out why.

Refer child to hospital

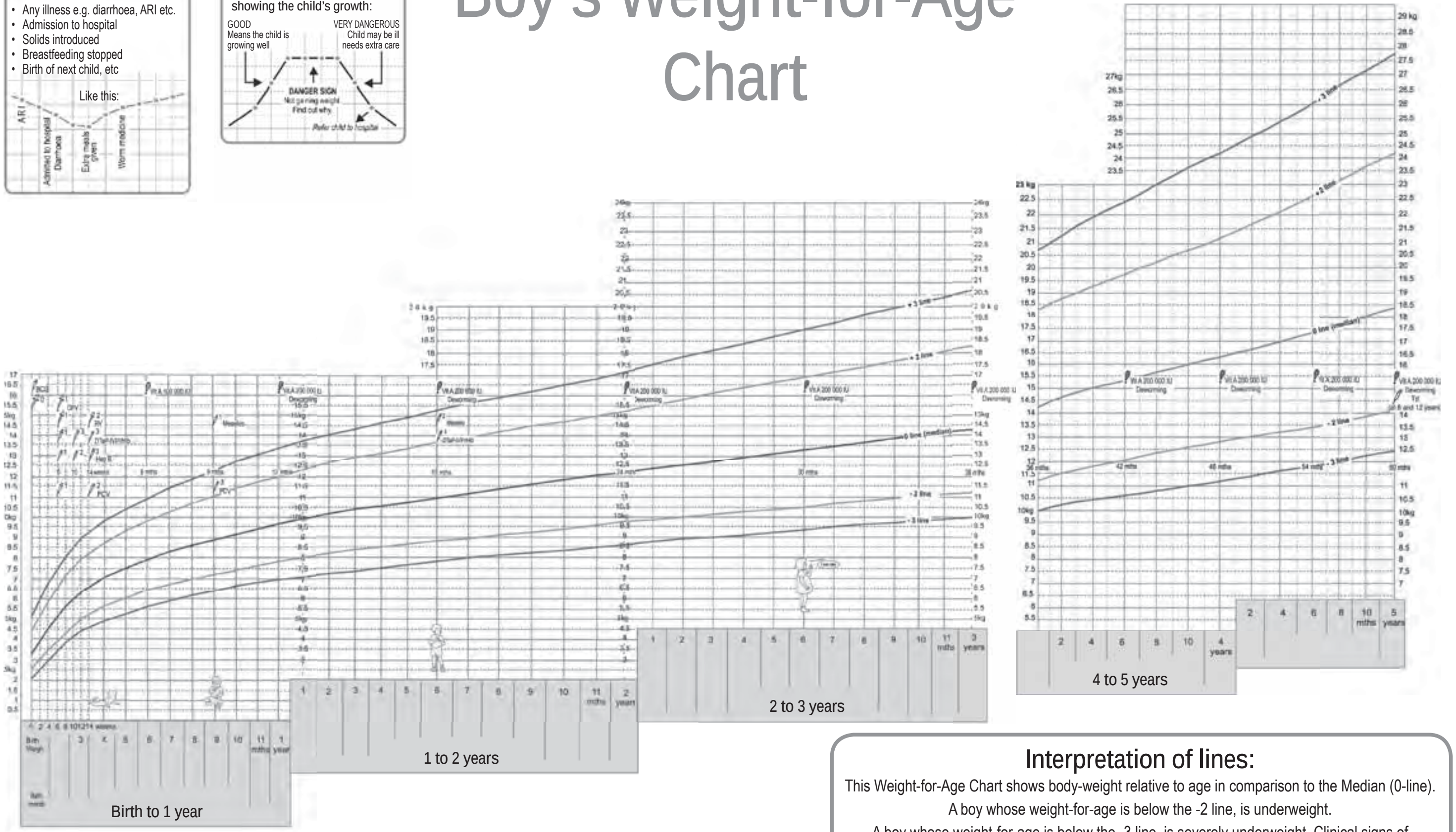

Interpretation of lines:

This Weight-for-Age Chart shows body-weight relative to age in comparison to the Median (0-line).
A boy whose weight-for-age is below the -2 line, is underweight.
A boy whose weight-for-age is below the -3 line, is severely underweight. Clinical signs of Marasmus or Kwashiokor may be observed.

If his line crosses a z-score line and the shift is away from the median, this may indicate a problem or risk of a problem.

If his line stays close to the median, occasionally crossing above or below it, this is fine.

Girl's Weight-for-Age Chart

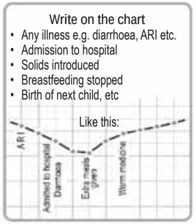

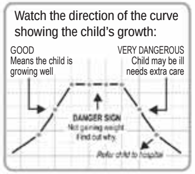

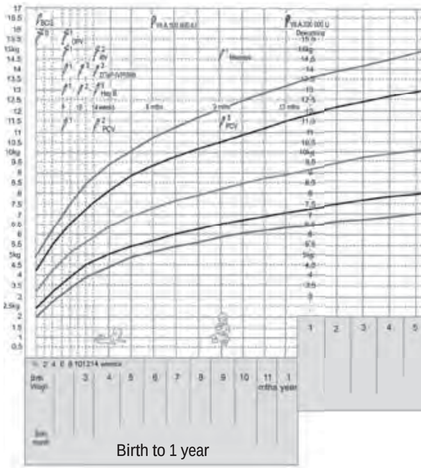

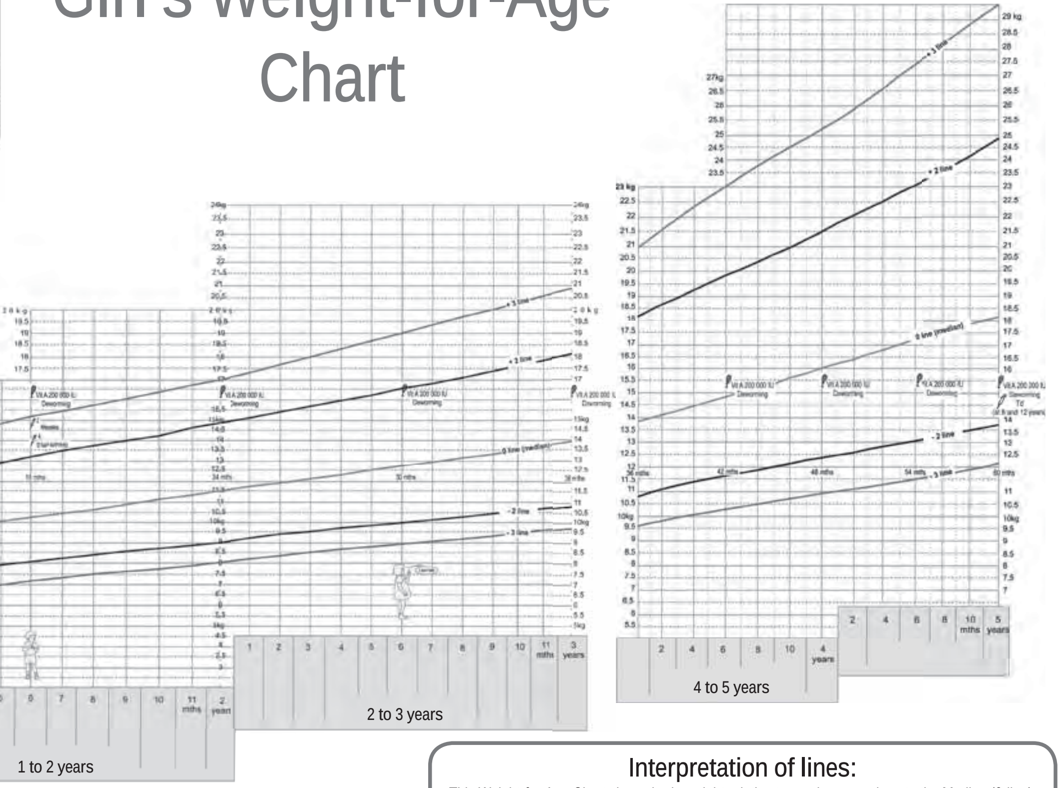

Interpretation of lines:

This Weight-for-Age Chart shows body-weight relative to age in comparison to the Median (0-line).

A girl whose weight-for-age is below the -2 line, is underweight.

A girl whose weight-for-age is below the -3 line, is severely underweight. Clinical signs of Marasmus or Kwashiokor may be observed.

If her line crosses a z-score line and the shift is away from the median, this may indicate a problem or risk of a problem.

If her line stays close to the median, occasionally crossing above or below it, this is fine.

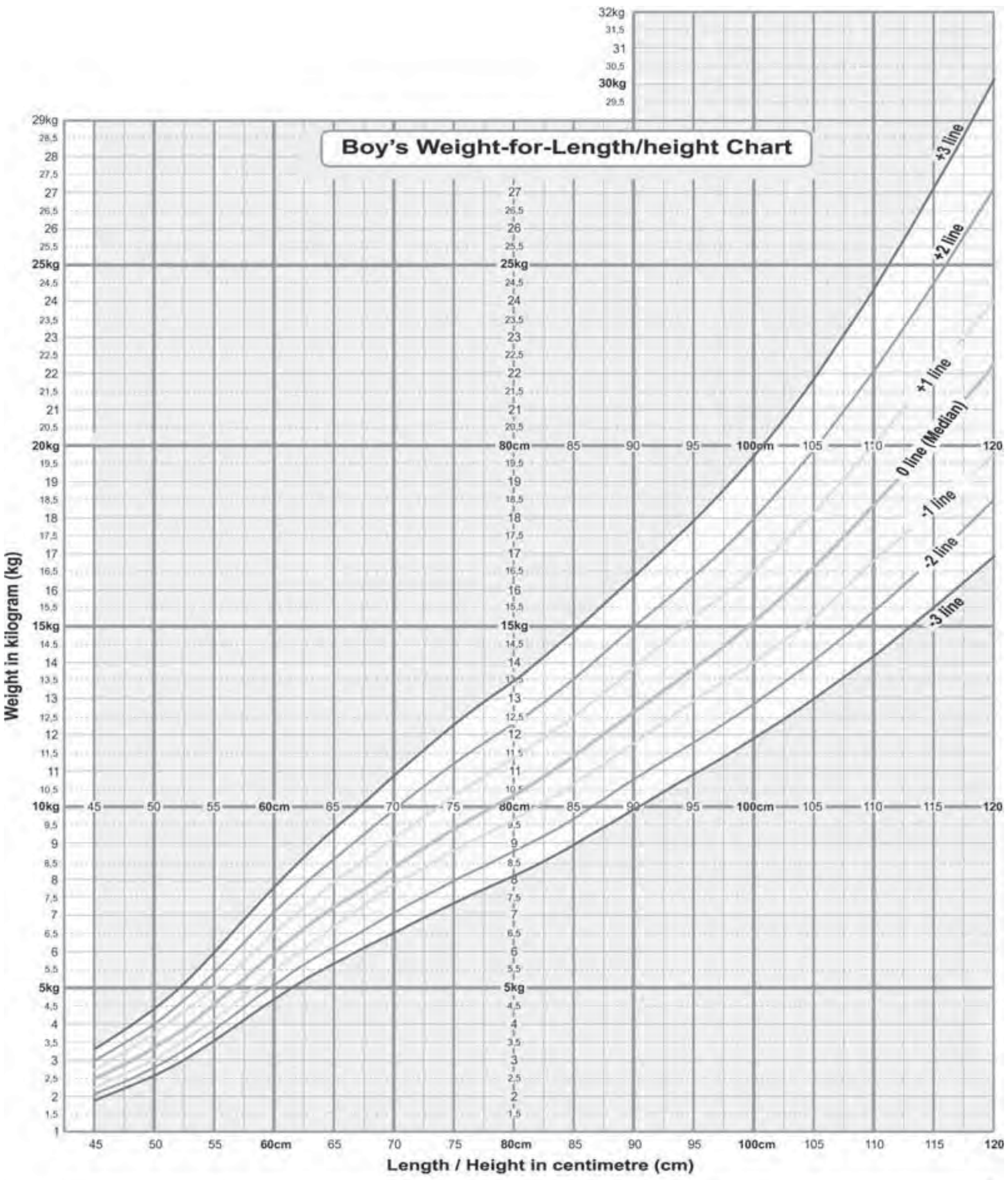

This **Weight-for-Length/height Chart** shows body-weight relative to length/height in comparison to the Median (the 0 z-score line).

A boy whose weight-for-length/height is above the +3 line, is **obese.**

A boy whose weight-for-length/height is above the +2 line, is **overweight**.

A boy whose weight-for-length/height is below the -2 line, is **wasted.**

A boy whose weight-for-length/height is below the -3 line, is **severely wasted. Refer for urgent specialised care.**

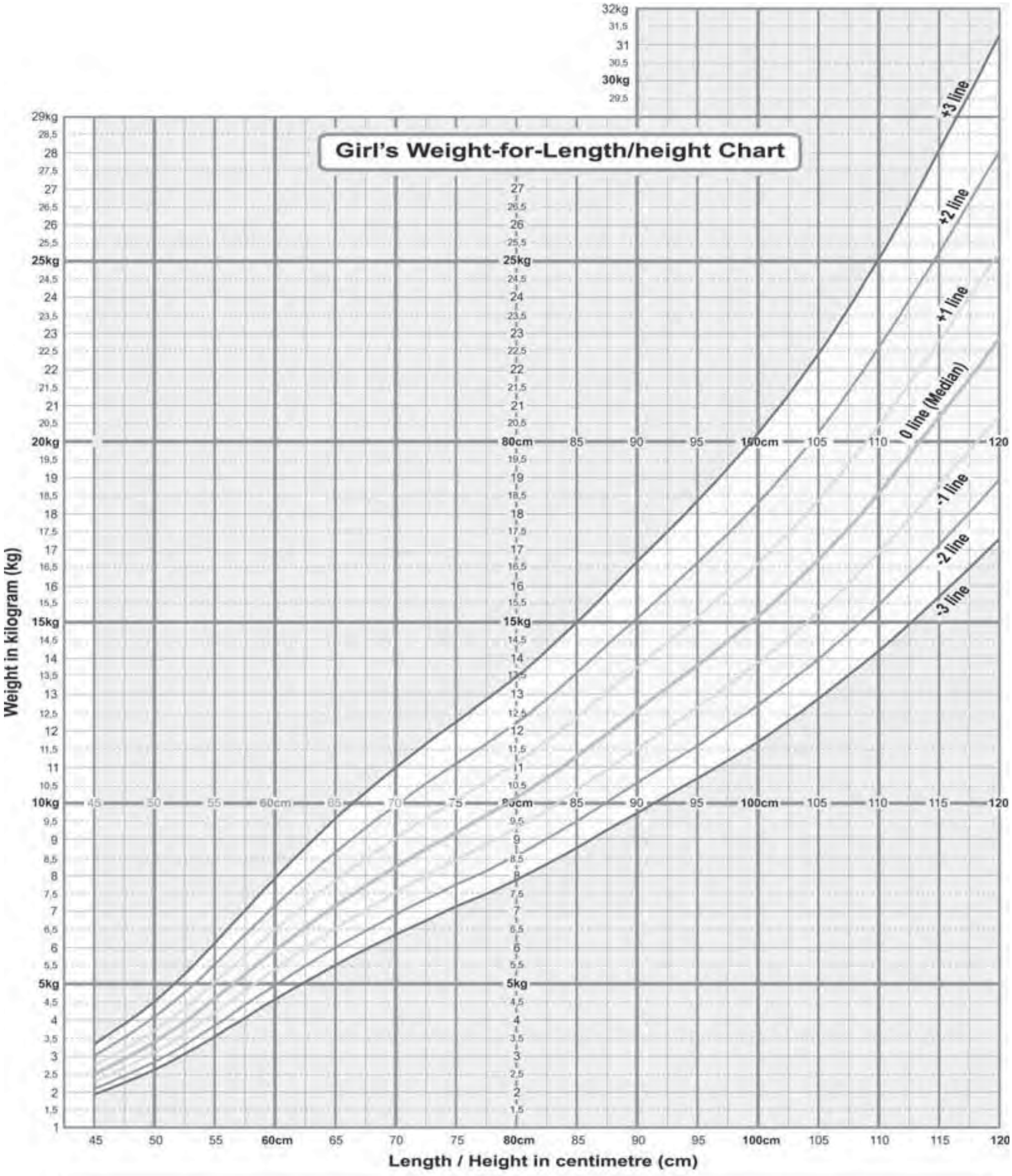

This **Weight-for-Length/height Chart** shows body-weight relative to length/height in comparison to the Median (the 0 z-score line).

A girl whose weight-for-length/height is above the +3 line, is **obese.**

A girl whose weight-for-length/height is above the +2 line, is **overweight.**

A girl whose weight-for-length/height is above the +1 line, shows possible risk of **overweight.**

A girl whose weight-for-length/height is below the -2 line, is **wasted.**

A girl whose weight-for-length/height is below the -3 line, is **severely wasted. Refer for urgent specialised care.**

INDEX OF CONDITIONS

Abscess, retropharyngeal	17.1
Acne	5.5
Acute bacterial tracheitis	17.3
Acute kidney injury (renal failure, acute)	6.14
Adolescent chronic disease: transition of care	22.2
Adrenal hyperplasia, congenital	7.2
Adrenal insufficiency, acute	7.3
Allergies to penicillins	23.3
Allergies to sulphonamides	23.5
Amoebiasis (entamoeba histolytica)	8.2
Anaemia of chronic disorders (infection or disease)	3.7
Anaemia, aplastic	3.1
Anaemia, haemolytic	3.2
Anaemia, iron deficiency	3.5
Anaemia, megaloblastic	3.4
Anaemia, sickle cell	3.8
Anaesthetic and post anaesthetic care of children	21.7
Anaphylaxis/anaphylactic reactions	1.5
Anorexia nervosa, bulimia nervosa (eating disorders)	22.9
Anticholinergic poisoning	18.3
Anticoagulant poisoning	18.4
Antidepressant (tricyclic) poisoning	18.5
Antiretroviral therapy and anticonvulsants	13.15
Anxiety disorders	14.11
Aphthous ulcers	2.3
Apnoea, neonatal	19.1
Approach to the resuscitation of the child	1.3
Arthritis, juvenile idiopathic	11.3
Arthritis, septic (pyogenic)	11.1
Ascites, due to hypoalbuminaemia and/or portal hypertension	2.30
Asthma attack, acute	15.15
Asthma, chronic	15.20
Asthma, infrequent	15.22

Asthma, persistent	15.23
Attention deficit hyperactivity disorder (ADHD)	14.3
Behavioural problems associated with intellectual disability	14.27
Bleeding oesophageal varices	2.28
Bronchiectasis	15.1
Bronchiolitis	15.26
Bullae	5.1
Burns	1.17
Candidiasis	5.8
Candidiasis, oral	2.3
Candidiasis, systemic and other	8.5
Cardiac dysrhythmias	4.1
Cardiorespiratory arrest	1.7
Caustic or corrosive agents, ingestion	18.7
Cellulitis and erysipelas	5.6
Cerebral oedema in diabetic coma	7.19
Cerebrovascular disease/stroke	13.25
Chemical burn to the eye	16.4
Childhood psychosis	14.14
Children with prosthetic heart valves	4.41
Cholera	2.5
Chronic bullous disease of childhood	5.2
Chronic kidney disease (renal failure, chronic)	6.19
Chronic lung infections	15.1
Cirrhosis	2.29
Conditions with predominant wheeze	15.15
Congenital glaucoma	16.8
Conjuctivitis, allergic	16.2
Constipation/faecal loading	2.6
Contraception, teenage pregnancy and teratogenicity risks	22.4
Convulsions, not febrile convulsions	1.10
Cough with predominant fever and tachypnoea	15.28
Cutaneous larva migrans/ancylostoma braziliense (dog hookworm)	8.3

Cyanotic congenital heart disease with hypoxaemic attacks/spells (hypercyanotic spells)	4.6
Cyanotic heart disease in the newborn	19.3
Cystic fibrosis	2.7
Cytomegalovirus (CMV) infection	8.7
Cytomegalovirus (CMV) retinitis	16.3
Delayed hypersensitivity reactions	23.3
Dental and oral disorders	2.1
Depression in childhood and adolescence	14.7
Diabetes in adolescence	22.7
Diabetes insipidus	7.4
Diabetes mellitus, type 2	7.29
Diabetes mellitus	7.6
Diabetes mellitus in adolescents	7.28
Diabetes mellitus, insulin dependent (type 1)	7.6
Diabetes mellitus, insulin dependent: acute complications	7.19
Diarrhoea, acute	2.9
Diarrhoea, persistent	2.19
Dilated cardiomyopathy	4.16
Diphtheria	8.8
Disorders of puberty	7.40
Disorders of sexual development (DSD)	7.1
Disruptive mood dysregulation disorder (DMDD)	14.10
Drug allergies	23.1
Drug reactions	5.4
Drug related anaphylaxis	23.2
Drug related angioedema	23.2
Drug related urticaria	23.2
Dysentery	2.23
Dyslipidaemia	4.24
Eczema	5.7
Effusion and empyema	15.38
Endocarditis, infective	4.8
Enterocolitis, necrotising	19.5

Enuresis	6.25
Envenomation	18.21
Epidermolysis bullosa	5.1
Epiglottitis	15.39
Epilepsy	13.5
Epistaxis (nose bleed)	17.4
Erythema and desquamation	5.2
Erythema Multiforme/Stevens-Johnson syndrome	5.2
Ethanol poisoning	18.8
Eye infection, complicated (severe eye infection)	16.1
Fungal infection	15.32
Gastrointestinal disorders	2.5
Gastro-oesophageal reflux disease (GORD)	2.24
General anaesthesia	21.8
General anaesthesia, induction	21.9
General anaesthesia, maintenance	21.11
General anaesthesia, preparation	21.8
Generalised anxiety disorder (GAD)	14.12
Gingivitis, uncomplicated	2.1
Growth disorders	7.32
Guidelines for management of diabetics on sick days	7.17
Haemophilia A and B	3.10
Haemorrhagic disease of the newborn	3.14
Haemorrhagic disease of the newborn	19.7
Headaches	13.16
Heart failure	4.20
Heart failure in neonates	19.9
Heart failure, acute	4.23
Heart failure, acute severe with pulmonary oedema and shock	4.22
Helminthiasis, intestinal	8.1
Henoch Schönlein purpura (HSP)	12.1
Hepatic disorders	2.28
Hepatitis B, chronic	2.34

Hepatitis, chronic, autoimmune	2.33
Hepatitis, toxin induced, acute	2.32
Hepatitis, viral, acute	2.31
Herpes gingivostomatitis	2.3
Herpes keratitis and conjuctivitis	16.2
HIV exposed infant	9.4
HIV in adolescence	9.26
HIV infected infant/child	9.8
HIV papular pruritic eruption	5.12
Human immunodeficiency virus infections	9.1
Hydatid disease	8.3
Hyperbilirubinaemia, conjugated	19.22
Hyperbilirubinaemia, unconjugated	19.21
Hyperglycaemic ketoacidosis	7.20
Hyperkalaemia	7.34
Hypertension in children	4.27
Hypertension, acute severe	4.37
Hypertension, chronic	4.38
Hyperthyroidism, Graves disease	7.38
Hypocalcaemia in children	7.33
Hypocalcaemia, neonatal	19.12
Hypoglycaemia in children	7.30
Hypoglycaemia in diabetics	7.26
Hypoglycaemia, neonatal	19.13
Hypogonadism in chronic disease	22.4
Hypokalaemia	7.34
Hypopituitarism	7.35
Hypothyroidism in older children and adolescents	7.37
Hypothyroidism, neonatal	7.36
Hypoxia/ischaemia of the newborn (perinatal hypoxia/hypoxic-ischaemic encephalopathy)	19.15
ICU sedation, infant and child	21.2
ICU sedation, neonate	21.1
Idiopathic thrombocytopaenic purpura (ITP)	3.14

Idiopathic thrombocytopenic purpura (ITP)	5.11
Immediate hypersensitivity reactions	23.2
Immune reconstitution inflammatory syndrome (IRIS)	9.25
Impetigo	5.13
Infections	5.11
Inflammatory bowel disease (IBD)	2.27
Inflammatory polyneuropathy (Guillain-Barré syndrome)	13.20
Inhalation, foreign body	1.10
Intra-osseous infusion in emergencies	1.16
Iron poisoning	18.9
Jaundice, neonatal	19.20
Jaundice, neonatal, prolonged	19.23
Juvenile idiopathic arthritis (JIA)	12.2
Kaposi's sarcoma	5.12
Kawasaki syndrome/mucocutaneous lymph node syndrome	12.7
Lactic acidosis	9.22
Laryngotracheobronchitis, acute viral (croup)	15.41
Leucocoria	16.9
Lipodystrophy	9.24
Liver failure, acute	2.35
Local and regional anaesthesia	21.7
Loss of vision	16.10
Lumbar puncture	13.1
Lung abscess	15.3
Macules and papules	5.4
Malaria	8.10
Malaria prophylaxis - self provided care	8.14
Malnutrition	2.38
Malnutrition, severe acute	2.38
Management of anaesthetic and post anaesthetic complications	21.13
Management of pain	20.1
Mastoiditis	17.6
Measles	8.15

Meningitis bacterial, neonatal	19.28
Meningitis, acute bacterial	8.17
Meningitis, cryptococcal	8.21
Meningitis, tuberculous (TBM)	8.22
Meningococcaemia	5.11
Meningo-encephalitis/encephalitis, acute viral	8.26
Metabolic syndrome associated cardio-metabolic risk	22.6
Mood disorders	14.7
Mumps	8.28
Myasthenia gravis	13.23
Mycobacterium avium complex (MAC) infection	8.29
Myocarditis	4.15
Necrotising periodontitis	2.2
Nephrological/urological disorders	6.1
Nephropathy	7.28
Nephrotic syndrome (NS)	6.8
Neurocysticercosis	13.18
Neuroleptic poisoning	18.11
Neuromuscular disorders	13.20
Non-penetrating eye injury	16.6
Non-typhoid salmonella (NTS)	8.40
Obesity	7.38
Obesity in adolescence	22.9
Obsessive compulsive disorder (OCD)	14.13
Opioid poisonong	18.13
Organophosphate poisoning	18.12
Osteitis/osteomyelitis, acute	11.3
Other situations or contexts in which psychotropic drugs may be prescribed	14.18
Otitis externa	17.7
Otitis media, acute	17.7
Otitis media, chronic, suppurative	17.8
Otitis media, subacute with effusion	17.8
P. falciparum malaria, non-severe, uncomplicated	8.11

P. falciparum malaria, severe, complicated (or if repeated vomiting)	8.12
P. ovale, *p vivax* and *p. malariae*	8.14
Paediatric emergencies	1.1
Palliative care	20.6
Paracetamol poisoning	18.14
Parenteral nutrition	21.4
Parenteral nutrition, neonatal	21.5
Parenteral nutrition, paediatrics	21.6
Patent ductus arteriosus (PDA) in the newborn	19.30
Penetrating eye injury with/without a foreign body	16.5
Peptic ulcer disease	2.26
Pericardial effusion	4.17
Pericarditis	4.19
Periodontitis	2.1
Pertussis	8.30
Pervasive developmental disorders (PDDS)	14.19
Petrochemical poisoning	18.17
Pleural disease	15.38
Pneumocystis jiroveci pneumonia (PCP)	8.31
Pneumonia	15.28
Pneumonia due to anaerobic infection	15.33
Pneumonia in HIV exposed or infected children	15.35
Pneumonia, atypical due to mycoplasma or chlamydial infection	15.33
Pneumonia, nosocomial	15.37
Pneumonia, staphylococcal	15.34
Pneumonia, viral infection	15.31
Poisoning	18.1
Poliomyelitis (acute flaccid paralysis)	8.31
Portal hypertension	2.30
Post exposure prophylaxis following alleged penetrative sexual abuse	9.26
Post operative care	21.12
Post streptococcal glomerulonephritis	6.1
Post traumatic stress disorder (PTSD)	14.12

Predisposing conditions and modification of antimicrobial therapy	15.31
Prematurity/preterm neonate	19.31
Psoriasis	5.9
Psychiatric presentations in HIV infected children and adolescents	14.18
Purpura	5.11
Rabies	8.32
Rapid triage of the child presenting with acute conditions in casualty/out patients	1.1
Recurrent abdominal pain	2.51
Respiratory distress in the newborn	19.34
Resuscitation of the newborn	19.38
Retinopathy of prematurity (ROP)	16.7
Rheumatic fever, acute	4.13
Rhinitis, allergic	17.9
Rickets	2.49
Salicylate poisoning	18.17
Schistosomiasis (bilharzia)	8.4
Schizophrenia	14.15
Scorpion stings	18.21
Sedation for intensive care procedures	21.1
Sedation of acutely disturbed child or adolescent awaiting psychiatric evaluation	14.3
Sedative-hypnotic poisoning	18.19
Seizures	13.2
Seizures, febrile	13.10
Seizures, neonatal	19.41
Sepsis (outside the neonatal period)	8.45
Septicaemia of the newborn	19.44
Shock	1.11
Sinusitis, acute	17.10
Sinusitis, chronic	17.10
Sinusitis, complicated	17.11
Sinusitis, uncomplicated	17.12
Skin and mucosal disorders in HIV	5.11

Snakebite	18.22
Special considerations in HIV infected children	3.19
Specific adverse events and complications	9.22
Specific allergies	23.3
Spider bites (widow spiders)	18.25
Spider bites, necrotic arachnidism	18.26
Staphylococcal scalded skin syndrome	5.1
Staphylococcal septicaemia	8.46
Status epilepticus (convulsive)	13.11
Strabismus	16.9
Substance abuse	14.19
Sulfonylurea	18.19
Sydenham's chorea	13.24
Sympathomimetic agent poisoning	18.20
Syphilis, early congenital	19.46
Systemic lupus erythematosus	12.9
Takayasu arteritis	12.11
Tetanus	8.36
Tetanus, neonatal	19.48
Tetralogy of fallot	4.7
Thrombocytopaenia	3.19
Tic disorders	14.17
Tick bite fever	8.38
Tonsilitis, complicated (peritonsillar cellulitis, peritonsillar abscess)	17.2
Toxoplasmosis	8.39
Trauma	1.17
Tuberculosis and HIV	9.21
Tuberculosis, perinatal	15.4
Tuberculosis, pulmonary	15.5
Typhoid	8.39
Upper airway diseases	15.39
Urinary tract infection (UTI)	6.4
Urticaria	5.10

Varicella (chickenpox)	8.42
Venous thrombo-embolic disease	3.17
Vesicles and pustules	5.11
Volatile solvents	18.8
Von Willebrand disease	3.13
Warts	5.13
Wasting syndrome	9.26
Worm bolus	2.50
Zoster	8.44

INDEX OF MEDICINES

ACE inhibitor	4.22, 4.23, 4.26, 4.40, 6.10, 6.24, 7.28, 19.10
Acetazolamide	8.25
Abacavir	9.13, 9.14, 9.15, 9.17, 9.22, 9.25
Abacavir/lamivudine	9.14
Acetic acid 2% in alcohol	17.7
Acetic acid 1% in sodium chloride 0.9%	17.9
Acetylcysteine	18.2, 18.16
Aciclovir	2.4, 8.27, 8.43, 8.44, 15.43, 16.3
Adenosine	4.4
Albendazole	2.21, 8.2, 8.3, 8.4, 13.19, 13.20
Albumin 20%	1.22, 6.10
Alcohol 95%	19.34
Aldosterone antagonist	4.23
Alfacalcidol	6.22, 7.34
Alpha-blocker	4.38, 4.41
Alprostadil	19.4
Amethocaine	16.4, 16.6
Amikacin	1.24, 3.2, 8.29, 15.12, 15.13, 15.37, 15.38
Aminoglycoside	11.5, 19.37, 19.45
Aminophylline	15.20, 19.2
Amiodarone	1.9, 4.5
Amlodipine	4.38, 4.40, 4.41, 6.3
Amoxicillin	2.26, 2.43, 4.12, 8.16, 15.27, 15.30, 15.31, 17.8, 17.10, 18.17
Amoxicillin/clavulanic acid	2.2, 6.6, 6.7, 8.34, 15.2, 15.3, 15.31, 17.2, 1 7.3, 17.6, 17.8, 17.12
Amphotericin B	8.7, 8.22, 15.32, 15.33, 19.45
Ampicillin	2.19, 2.37, 2.43, 4.12, 8.16, 15.2, 15.3, 15.31, 15.33, 15.43, 19.6, 19.29, 19.37
Anti-D immunoglobulin	19.22

Antiretroviral therapy	9.6, 9.9, 9.10, 9.11, 9.12, 9.13, 9.19, 9.21, 9.22, 9.23, 9.25, 9.27, 13.15
Aqueous cream (UEA)	5.8
Artemether/lumefantrine	8.11, 8.13
Aspirin	4.14, 12.8, 12.12, 13.26
Atenolol	4.40, 7.38
Atropine	4.5, 13.23, 16.7, 18.13, 21.2, 21.11, 21.14
Azathioprine	2.33, 12.11
ß$_2$ agonist, long acting	15.23
ß$_2$ agonist, short acing	15.16, 15.17, 15.18, 15.19, 15.22, 15.23
Bacillus Calmette-Guerin (BCG) Vaccine	9.11, 15.5
Beclomethasone	15.23, 17.10
Benzathine benzylpenicillin (depot formulation)	4.14, 6.2, 8.9, 13.25, 19.47
Benzodiazepine	20.6
Benzoyl peroxide	5.5
Benzylpenicillin (Penicillin G)	4.11, 8.9, 8.46, 19.48
Betamethasone	5.8, 5.12
Biperiden	14.3, 18.12
Bisthmus iodine paste	17.5
Boomslang antivenom	18.25
Budesonide	15.23
Bupivacaine 0.5% with dextrose without adrenaline	21.8
Bupivacaine 0.5% without adrenaline and dextrose	21.8
Caffeine	19.2
Calamine lotion (BP)	5.12, 8.43
Calcitriol	7.34
Calcium, oral	7.34
Calcium carbonate	6.10, 6.22
Calcium channel blocker	4.40, 4.41
Calcium gluconate	6.17, 7.33, 18.22, 18.26, 19.13, 19.19, 19.43, 21.15

Captopril	4.17, 4.22, 4.23, 4.26, 4.40, 19.10
Carbamazepine	8.44, 13.8, 13.9, 13.15, 13.23, 20.5
Carbimazole	7.38
Cefazolin	10.2
Cefotaxime	1.15, 2.21, 2.24, 2.37, 3.2, 3.10, 5.7, 8.41, 11.2, 11.5, 17.4, 17.6, 17.12, 19.18, 19.29, 19.45
Ceftazidime	4.12, 11.5
Ceftriaxone	1.15, 1.24, 2.18, 2.21, 2.24, 2.43, 3.2, 3.10, 4.12, 4.19, 4.20, 5.7, 6.6, 8.13, 8.19, 8.20, 8.40, 8.41, 8.46, 11.2, 11.5, 15.31, 15.41, 17.1, 17.3, 17.4, 17.6, 17.12
Cephalexin	5.4, 5.7, 5.8, 8.43
Cephalosporins	1.15, 3.10, 8.40, 17.1, 17.3, 19.45
Cetirizine	5.5, 5.8, 5.10, 17.10
Charcoal, activated	18.2, 18.4, 18.6, 18.12, 18.14, 18.16, 18.18, 18.19, 18.20
Chloramphenicol	8.17 10.2, 16.1, 16.7
Chlorhexidine	2.1, 2.2, 2.4, 5.4, 8.34, 18.24, 19.49, 20.7
Chlorhexidine/benzydamine	20.8
Chloroquine	8.14, 12.10
Chlorphenamine	1.6, 5.5, 5.10, 5.12, 8.43, 17.8, 23.2, 23.3
Chlorpromazine	19.50
Ciprofloxacin	2.6, 2.18, 2.24, 8.20, 8.40
Citalopram	14.9, 14.18
Clarithromycin	8.29, 8.30, 8.39, 15.34
Clindamycin	8.12, 8.13, 11.3, 11.5, 23.4
Clotrimazole	5.9, 8.7, 20.8
Cloxacillin	4.11, 4.19, 4.20, 5.1, 5.4, 5.7, 8.46, 8.47, 11.2, 11.5, 15.3, 15.34, 15.39, 15.43
Coal tar	5.9

Codeine phosphate	3.10, 3.11
Combination antiretroviral therapy	9.5, 9.6, 9.8, 9.9, 9.11, 9.20, 9.21, 9.26
Combined oral contraceptive	3.14, 9.28
Corticosteroids	2.33, 4.20, 5.8, 6.11, 12.4, 12.10, 15.18, 15.22, 15.23, 17.4, 17.10
Co-trimoxazole (Trimethoprim/sulfamethoxazole)	2.21 2.22, 9.8, 9. 10, 9.11, 15.37, 23.7
Cyclopentolate	16.3, 16.7
Cyclopentolate/phenylephrine	16.8
Cyclophosphamide	6.13, 12.11, 12.12
Dantrolene	21.14
Dapsone	23.6
Darrows half strength with dextrose 5%	1.22, 2.12, 2.15, 2.16, 2.17, 2.37, 6.17, 18.18
Desferrioxamine (deferoxamine)	18.11
Desmopressin	3.14, 6.26, 7.5
Dexamethasone	8.19, 8.20, 13.13, 13.20, 15.42, 16.7, 17.4
Dextrose	2.41, 19.35
Dextrose 10%	2.37, 2.41, 6.2, 6.16, 6.21, 6.22, 7.4, 7.24, 7.27, 7.31, 8.14, 18.9, 18.20, 19.14, 19.17, 19.20, 19.39, 19.43
Dextrose 15%	19.14
Dextrose 5%	6.2, 6.16, 6.21, 6.22, 8.13, 18.16, 18.25, 18.26, 19.13, 19.43,21.2, 21.3, 21.11, 21.12, 21.13, 23.7
Dextrose 5% in sodium chloride 0.45%	7.23, 15.16
Dextrose 5% in sodium chloride 0.9%	2.17, 6.17, 7.31, 13.13
Dextrose 50 %	2.17, 2.41, 6.17, 7.27, 7.31
Diazepam	2.37, 8.37, 13.13, 13.14, 14.2, 14.25, 14.26, 18.4, 18.8, 18.21, 19.50, 20.6, 20.7
Didanosine	9.22, 9.23
Digoxin	4.16, 4.23, 4.24, 19.11

Dinoprostone (prostaglandin E2)	19.4
Diuretics	4.23, 4.40, 4.41, 19.10, 19.30
Dobutamine	1.14, 1.15, 4.22, 19.11, 19.18, 19.19
Dopamine	19.6, 19.18, 19.36, 19.46
Doxapram	19.3
Doxycycline	5.5, 8.38
Efavirenz	9.13, 9.14, 9.15, 9.18, 9.22, 9.23
Emtricitabine/tenofovir/efavirenz	9.6
Lamivudine/tenofovir/efavirenz	9.6
Emulsifying ointment (UE)	5.8
Enalapril	4.23, 4.26, 4.40, 6.10, 6.24, 7.28
Epinephrine (adrenaline)	1.6, 1.8, 1.9, 1.14, 1.15, 4.5, 4.22, 15.27, 15.42, 15.43, 18.24, 19.39, 19.40
Erythromycin	2.21, 5.7, 6.2, 8.9, 23.4
Erythropoietin	6.23
Ethambutol (EMB/E)	8.29, 15.8, 15.10
Ethinyl oestradiol	22.5
Ethionamide	8.24, 15.12, 15.13
Factor IX	3.12, 3.13, 18.5
Factor VIII	3.12, 3.13, 3.14
Factor VIII inhibitor-bypassing activity (FEIBA)	3.12
Fentanyl	21.2, 21.3, 21.11
Ferrous gluconate	3.5, 3.6, 18.9
Ferrous lactate	3.6, 18.9, 19.33
Ferrous sulphate compound (BPC)	3.6, 18.9
Flucloxacillin	5.1, 11.3, 11.5, 15.34, 15.39
Fluconazole	5.9, 8.7, 8.22, 15.32, 15.33
Fludrocortisone	7.2
Fluorescein 2%	16.4
Fluoroquinolone	8.29, 17.9
Fluoxetine	14.9, 14.12, 14.13, 14.14, 14.18

Fluticasone/salmeterol	15.23
Folic acid	2.45, 3.3, 3.5, 4.7, 6.10, 6.22, 12.5, 12.10, 12.12
Fresh frozen plasma	2.36, 3.12, 18.5, 19.8
Furosemide	2.30, 2.45, 4 .15, 4.16, 4.22, 4.23, 4.38, 4.40, 4.41, 6.3, 6.10 , 6.18, 8.25, 19.11, 19.19, 19.30
Gammaglobulin	19.22
Ganciclovir	8.8, 15.37, 16.4
Gentamicin	2.19, 2.22, 2.36, 2.43, 4.11, 4.12, 6.7, 8.16, 11.5, 15.2, 15.3, 15.31, 15.33, 15.39, 16.1, 19.6, 19.37, 19.45
Glucagon	7.26, 7.27, 19.15
Glucose	7.26, 7.27, 7.30
Glycerine suppositories	19.49, 19.50
Haloperidol	13.25, 14.3, 14.17, 14.19
Halothane	21.10, 21.11
Heparin, unfractionated	3.18
Hepatitis B immunoglobulin (HBIG)	2.32
Hepatitis B vaccine (HepB)	2.32, 6.11, 6.25
Hydrochlorothiazide	2.30, 4.40, 4.41, 6.10, 7.5
Hydrocortisone	1.6, 1.15, 5.8, 5.10, 5.12, 7.2, 7.4, 7.31, 7.36, 9.26, 15.16, 15.18, 15.19
Hydroxypropylmethycellulose 0.3%	21.12
Hydroxyurea	3.10
Hyoscine butylbromide	20.7, 20.8
Ibuprofen	2.4, 3.10, 4.14, 4.20, 5.7, 7.5, 11.3, 11.5, 12.2, 12.4, 12.6, 13.17, 19.31, 20.4, 21.13
Imidazole	5.9, 8.7
Immunoglobulin, human normal	3.16, 8.17, 12.8, 13.23
Influenza vaccine	3.4, 3.10, 3.17, 8.20, 15.2
Insulin	6.17, 7.13, 7.18, 7.19, 7.24, 7.25, 7.29
Insulin, intermediate acting	7.12, 7.14, 7.15, 7.16, 7.17

Insulin, rapid	7.15, 7.17, 7.19
Insulin, short acting	7.12, 7.14, 7.15, 7.16, 7.17, 7.19, 7.25
Ipratropium bromide	15.16, 15.18, 15.19
Iron	1.20, 2.45, 3.6, 3.7, 3.8, 4.7, 19.33
Isoflurane	21.11
Isoniazid (H/INH)	8.24, 15.5, 15.8, 15.13, 15.14
Ispaghula husk	2.7
Ketamine	1.23, 4.6, 19.7, 20.3, 21.1, 21.2, 21.3, 21.4, 21.10
Labetalol	4.38, 6.3
Lactulose	2.7, 2.36, 20.5, 20.7
Lamivudine	9.13, 9.14, 9.15, 9.17
Lamotrigine	13.8, 13.9
Levoflocaxin	8.29, 15.12
Levonorgestrel	9.27, 22.6
Levothyroxine	7.37
Lidocaine (lignocaine)	19.19, 19.42
Lidocaine 1% (lignocaine)	4.18, 12.4, 19.7
Lidocaine 2%	18.22. 21.8
Lidocaine 2% (oral)	2.4, 17.5
Lidocaine 2% with epinephrine (adrenaline)	21.7, 21.8
Lidocaine, topical	20.8
Lidocaine/prilocaine	20.3
Lipid emulsion 20%	19.19, 19.43
Liquid paraffin	2.7
Lopinavir/ritonavir	9.13, 9.14, 9.15, 9.16, 9.18, 9.22, 9.23
Lorazepam	13.14, 14.2, 14.3
Macrolides	8.30, 15.34, 23.4
Magnesium sulphate	2.43, 7.34, 15.16, 15.19, 19.13, 19.19, 19.43
Maintenance, Neonatal solution	2.41, 19.6, 19.36, 19.44
Maintenance, Neonatal solution with dextrose	8.19, 8.21, 8.27

Maintenance, Neonatal, Potassium Free solution	19.17
Maintenance, Paediatric with dextrose	8.19, 8.21, 8.27
Mannitol	7.20, 8.20, 8.25, 8.27, 13.13
Measles vaccine	9.11
Mebendazole	2.44, 2.50, 3.7, 8.2
Medroxyprogesterone acetate	9.28
Methotrexate	12.5, 12.6, 12.10, 12.12
Methylphenidate	14.5
Methylprednisolone	3.16, 12.4, 12.6, 12.8, 12.10
Metoclopramide	13.18, 20.6, 21.14
Metronidazole	2.18, 2.21, 2.22, 2.24, 2.26, 8.3, 8.37, 10.2, 15.3, 15.33, 17.1, 17.3, 19.6, 19.46, 19.50
Miconazole	8.7
Midazolam	1.23, 2.37, 4.4, 4.5, 13.13, 13.14, 20.3, 21.1, 21.3, 21.4, 21.9, 21.14
Morphine	4.6, 4.22, 6.4, 6.18, 18.27, 19.11, 19.50, 20.4, 20.5, 20.7, 21.2, 21.3, 21.11, 21.12, 21.13
Moxifloxacin	15.13
Multivitamin	2.30, 2.45, 3.7, 3.8, 6.10, 6.22, 19.33
Naloxone	18.14, 19.39, 21.14
Neostigmine bromide	13.23, 21.11
Nevirapine	9.5, 9.6, 9.7, 9.13, 9.23
Nitrous oxide	21.10, 21.11
Non-nucleoside reverse transcriptase inhibitors	9.18, 9.20, 9.23, 13.15
Nystatin	8.6
Octreotide	2.28
Ofloxacin	10.2, 17.9
Omeprazole	1.23, 2.25, 2.26, 2.28, 2.36
Ondasetron	20.6, 21.14
Oral rehydration solution (ORS)	2.12, 2.13, 2.15, 2.22, 6.17
Oxybutynin	6.26

Oxygen	1.5, 1.6, 1.10, 1.12, 1.17, 1.20, 1.25, 3.9, 3.18, 4.6, 4.15, 4.16, 4.22, 4.23, 6.4, 6.18, 6.21, 7.21, 8.30, 13.5, 13.8, 13.12, 15.2, 15.16, 15.17, 15.27, 15.30, 15.32, 15.36, 15.41, 15.42, 15.43, 19.2, 19.3, 19.8, 19.10, 19.11, 19.18, 19.33, 19.35, 19.36, 19.38, 19.40, 19.45, 19.47, 21.10, 21.11
Oxymetazoline	17.5, 17.10
Packed red cells	1.13, 1.22, 2.37, 2.45, 3.1, 3.9, 4.7, 6.18, 6.23, 8.14, 8.40, 8.41, 17.5, 19.6, 19.8, 19.18, 19.33, 19.36, 19.47
Pancreatic enzymes (lipase/amylase/protease)	2.8
Paracetamol	1.22, 1.23, 2.1, 2.2, 2.3, 2.4, 3.10, 3.11, 4.11, 5.7, 6.7, 8.13, 8.16, 8.20, 8.27, 8.28, 8.30, 8.39, 8.43, 13.5, 13.11, 13.13, 13.17, 15.30, 15.32, 15.43, 16.5, 17.2, 17.3, 17.4, 17.7, 17.8, 17.10, 17.11, 17.12, 18.22, 18.27, 19.50, 20.4, 21.12, 21.13
Petrollium jelly	5.13
Phenobarbitone	2.17, 13.8, 13.9, 13.14, 19.18, 19.42, 19.50
Phenoxymethylpenicillin	3.4, 3.10, 3.17, 4.14, 6.2, 6.11, 13.25, 23.5
Phenylephrine	4.7
Phenytoin	13.14, 13.15, 13.16, 19.8, 19.42
Phosphate-containing enema	2.7
Piperacillin	4.12
Piperacillin/tazobactam	15.38
Plasma	19.6, 19.36
Plasma lyophilised	2.36, 3.12, 18.5, 19.8
Platelets	2.36, 3.2, 3.16
Pneumococcal vaccine (conjugated)	3.4, 3.9, 3.17, 15.2

Pneumococcal vaccine (polysacchiride)	3.4, 3.16, 3.17, 6.11, 6.24
Podophyllin resin	5.13
Polio vaccine	8.32
Polyethylene glycol, sodium sulphate, electrolytes oral solution	2.7, 2.36, 18.3
Polyvalent antiserum (snake)	
Polyvalent snake antivenom	18.25
Potassium chloride	2.16, 2.17, 2.19, 2.37, 2.42, 6.10, 7.23, 7.24, 7.35, 8.7, 8.22, 15.32, 15.33
Potassium hydroxide	5.9
Povidone iodine	1.23, 8.34
Praziquantel	8.5
Prazosin	4.38, 4.41
Prednisone	3.3, 3.16, 3.20, 4.19, 4.20, 5.10, 6.12, 6.13, 7.3, 8.25, 9.26, 12.2, 12.5, 12.6, 12.7, 12.10, 12.12, 13.20, 15.16, 15.17, 15.18, 15.19, 15.37, 15.42, 20.5, 23.2
Primaquine	8.14
Procaine penicillin	19.48
Promethazine	1.6, 5.5, 20.7, 21.9
Propofol	21.1, 21.10
Propranolol	2.28, 4.7, 4.8, 4.41, 6.3, 7.38, 13.18
Protease Inhibitors	9.10, 9.13, 9.20,13.15
Pyrazinamide (Z)	8.24, 15.5, 15.8, 15.10, 15.12, 15.13
Pyridostigmine	13.24
Pyridoxine (vit B6)	9.22, 15.9, 15.10, 15.11, 15.13, 19.43
Quinine	8.11, 8.12, 8.13
Rabies immunoglobulin (RIG)	8.34, 8.35
Rabies vaccine	8.34, 8.35
Ranitidine	1.23
Recombinant factor VIIa	3.12

Rifampicin (R)	8.20, 8.24, 9.13, 9.16, 15.5, 15.8, 15.13, 15.14
Rifampicin/isoniazid (RH)	15.8, 15.9, 15.10, 15.11
Rifampicin/isoniazid/pyrazinamide/ ethambutol (RHZE)	15.9, 15.11
Ringer-Lactate	2.5, 18.6
Risperidone	14.11, 14.16, 14.17, 14.19
Ritonavir	9.13, 9.16, 9.18, 9.22
Salbutamol	1.6, 6.17, 15.2, 15.16, 15.17, 15.18, 15.19, 15.22, 15.23, 15.30, 21.14
Salicylic acid 2% and coal tar in white soft paraffin	5.9
Salicylic acid 25%	5.13
Scorpion antivenom	18.22
Silver sulfadiazine	1.24
Simvastatin	4.26, 6.11, 6.24
Sodium bicarbonate	2.16, 4.6, 6.17, 6.18, 6.23, 18.6, 18.18, 19.4, 19.36, 19.39
Sodium chloride	2.1, 2.3, 7.3
Sodium chloride 0.45 %	4.38, 6.22, 7.24
Sodium chloride 0.9 % (Normal saline)	1.6, 1.8, 1.9, 1.13, 1.14, 1.16, 1.21, 1.22, 2.5, 2.11, 2.15, 2.17, 3.14, 4.4, 4.6, 5.8, 5.10, 6.9, 6.22, 7.4, 7.21, 7.22, 7.23, 7.24, 8.7, 8.13, 8.22, 8.23, 8.25, 8.34, 8.35, 15.1, 15.2, 15.17, 15.18, 15.19, 15.32, 15.33, 15.42, 18.6, 18.10, 18.25, 18.26, 19.13, 19.18, 19.34, 19.36, 19.39, 21.2, 21.3, 21.11, 21.12, 21.13, 23.7
Sodium chloride 3 %	15.1, 15.2, 15.27
Sodium chloride 5 %	7.24, 13.13, 15.1, 15.27
Sodium polystyrene sulfonate	6.17, 6.23
Spider antivenom	18.26
Spironolactone	2.30, 4.16, 4.23, 6.10, 19.11

SSS (Sugar and salt solution)	2.13
Statins	4.26, 6.11, 6.24
Stavudine	9.13, 9.16, 9.17, 9.25
Succinylcholine	21.1, 21.3, 21.10, 21.14
Sucrose	20.2, 21.2, 21.12
Sugar Solution	2.41
Sunscreen	5.6
Surfactant	19.37
Surfactant, semi-synthetic	19.37
Tenofovir	9.10
Terizidone	15.12, 15.13
Testosterone cypionate	22.5
Tetanus toxoid immunoglobulin	1.24, 8.37, 18.24, 19.49, 19.50
Tetanus toxoid adsorbed vaccine (ATT)	18.24
Tetanus toxoid vaccine (TT)	1.24, 8.34, 8.37, 18.22, 19.50, 19.51
Theophylline	15.2
Thiamine	9.23
Thiopental	13.14, 21.10
Tilidine	1.23, 20.4, 21.13
Topiramate	13.18
Trace elements mix	2.43, 2.45, 2.46
Tranexamic acid	3.13, 3.14
Tretinoin	5.5, 5.6
Valproate (sodium)	13.8, 13.9
Vancomycin	1.24, 4.12, 8.19, 8.47, 11.2, 11.5, 15.34, 15.37, 15.38, 23.4
Varicella-Zoster immunoglobulin (VZIG)	8.43
Varicella-Zoster vaccine	6.11, 6.24, 8.44
Vecuronium bromide	19.50, 21.10, 21.11
Vitamin A (Retinol)	2.44, 8.16, 19.24
Vitamin B_{12}	3.5
Vitamin D	2.49, 7.34, 12.10, 19.23, 19.24

Vitamin E	19.23, 19.44
Vitamin K_1 (Phytomenodione)	2.30, 2.36, 18.5, 18.18, 19.8, 19.23, 19.24, 19.33
Vitamins, fat soluble	19.23, 19.24
Warfarin	3.18, 3.19, 4.42
Zidovudine	9.10, 9.13, 9.16, 9.17
Zinc and Castor oil	20.7, 20.8
Zinc, elemental	2.6, 2.19, 2.23

ABBREVIATIONS

3TC	Lamivudine
ABC	Abacavir
ACE Inhibitor	Angiotensin-converting enzyme inhibitor
ACTH	Adrenocorticotropic hormone
ADA	Adenosine deaminase
ADH	Antidiuretic hormone
ADHD	Attention Deficit Hyperactivity Disorder
ADR	Adverse Drug Reaction
AED	Antiepileptic Drug
AFP	Acute Flaccid Paralysis
AIDP	Acute Inflammatory Demyelinating Polyneuropathy
AKI	Acute Kindney Injury
ALP	Alkaline phosphatase
ALT	Alanine aminotransferase
AMAN	Acute motor axonal neuropathy
AMSAN	Acute motor-sensory axonal neuropathy
ANA	Anti-nuclear antibody
Anti-HBc	Hepatitis B core antibody
Anti-HBs	Hepatitis B surface antibody
APSGN	Acute poststreptococcal glomerulonephritis
ARDS	Acute Respiratory Distress Syndrome
ART	Antiretroviral therapy
ASO-titre	Antistreptolysin O-titre
AST	Aspartate aminotransferase
ATT	Tetanus toxoid adsorbed
AVPU scoring	Alert, response to voice, response to pain, unconscious scoring
AZT	Zidovudine
BCG	Bacillus Calmette-Guérin
BMI	Body Mass Index
BP	Blood Pressure
BSA	Body Surface Area

Ca	Calcium
cART	combination Antiretroviral Therapy
CBT	Cognitive Behavioural Therapy
CIMT	Carotid intima
CIPD	Chronic Inflammatory Demyelinating Polyneuropathy
CKD	Chronic Kidney Disease
Cl	chloride
cm	centimetre
CMV	Cytomegalovirus
CNS	Central Nervous System
COC	Combined oral contraceptives
CPAP	Continuous positive airway pressure
CPR	Cardiopulmonary resuscitation
CrCl	Creatinine clearance
CRF	Chronic Renal Failure
CRP	C-Reactive Protein
CSF	Cerebrospinal fluid
CT	Computed Tomography
CVP	Central Venous Pressure
CVT	Cerebral Venous Thrombosis
D4T	Stavudine
DC cardioversion	Direct current cardioversion
ddI	Didanosine
DET	Diethyltoluamide
DIC	Disseminated intravascular coagulation
DKA	Diabetic Ketoacidosis
dL	Decilitre
DMARD	Disease-modifying anti-rheumatic drug
DMDD	Disruptive Mood Dysregulation Disorder
DOT	Directly Observed Therapy
DSD	Disorders of sexual development
DSM	Diagnostic and Statistical Manual of Mental Disorders

DST	Drug susceptibility testing
ECG	Electrocardiogram
eCrCl	estimated Creatinine Clearance
EEG	Electroencephalography
EFV	Efavirenz
EM	Eythema Multiforme
EMB or E	Ethambutol
EPI	Expanded Programme on Immunization
EPSE	Extrapyramidal side-effects
ESR	Erythrocyte Sedimentation Rate
ETAT	Emergency Triage Assessment and Treatment
ETT	Endotracheal tube
FBC	Full Blood Count
FEIBA	Factor VIII inhibitor-bypassing activity
FEV	Forced expiratory volume
FSGS	Focal segmental glomerulosclerosis
FTA-ABS	Fluorescent Treponema antibody absorption test
FTC	Emtricitabine
FVC	Forced vital capacity
g	gram
G6PD	Glucose-6-phosphate dehydrogenase
GAD	Generalised Anxiety Disorder
GBS	Guillain-Barré syndrome
GCS	Glasgow Coma Scale
GEFS+	Generalised epilepsy with febrile seizures plus
GFR	Glomerular Filtration Rate
GIT	Gastrointestinal tract
GMP	Good Manufacturing Practice
GOR	Gastro-Oesophageal Reflux
GORD	Gastro-Oesophageal Reflux Disease
GTCS	Generalised tonic-clonic seizures
H	Isoniazid

HACEK	Haemophilus; Actinobacillus, Cardiobacterium homonis; Eikenella corrodens; Kingella
Hb	Haemoglobin
HBIG	Hepatitis B Immune Globulin
HBsAg	Hepatitis B surface antigen
HCO_3	Bicarbonate
HDL	High Density Lipoprotein
HepB	Hepatitis B vaccine
Hib	Haemophilus influenzae type B
Hib	Haemophilus influenzae type B
HIE	Hypoxic-ischaemic encelphalopathy
HIV	Human Immunodeficiency Virus
HLA	Human Leukocyte Antigen
HMD	Hyaline membrane disease
HMG-CoA	3-hydroxy-3-methylglutaryl coenzyme-A
HSP	Henoch-Schönlein Purpura
HSR	Hypersensitivity reaction
IAS	Intra-articular steroids
IBD	Inflammatory Bowel Disease
ICHD	International Classification of Headache Disorders
ICP	Intracranial Pressure
ICU	Intensive Care Unit
IDL	Intermediate Density Lipoprotein
IE	Infective endocarditis
IgM anti-HBc	IgM antibody against hepatitis B core antigen
IM	Intramuscular
IMCI	Intergrated Management of Childhood Illness
INR	International Normalised Ratio
IPT	Isoniazid Preventive Therapy
IPV	Inactivated polio vaccine
IRIS	Immune Reconstitution Inflammatory Syndrome
ITP	Idiopathic Thrombocytopaenic Purpura

IUCD	Intrauterine Contraceptive Device
IV	Intravenous
JC virus	John Cunningham virus
JIA	Juvenile Idiopathic Arthritis
JVP	Jugular Venous Pressure
K	Potassium
kg	Kilogram
kJ	Kilojoule
LABA	Long-acting beta agonist
LDH	Lactate Dehydrogenase
LDL	Low Density Lipoprotein
LFT	Liver Function Tests
LGE	Lineal gingival erythema
LGS	Lennox-Gastaut Syndrome
LIP	Lymphoid interstitial pneumonitis
LMA	Lymphangioleiomyomatosis
LOC	Loss of consciousness
LP	Lumbar Puncture
Lp(a)	Apolipoprotein
LPA	Line probe assay
LPV/r	Lopinavir/ritonavir
LTB	Laryngotracheobronchitis
m2	metre squared
MAC	Mycobacterium Avium Complex
MCH	Mean Corpuscular Haemoglobin
MCNS	Minimal change nephrotic syndrome
MCS	Microbiological Culture and Sensitivity
MCUG	Micturating Cystourethrogram
MCV	Mean Corpuscular Volume
MDR	Multi-drug resistance
Meq	Milliequivalent
Mg	Magnesium

mg	milligram
mL	millilitre
mm^3	cubic millimitre
mmHg	milliliters mercury
mmol	millimole
mOsm	milliosmole
MRI	Magnetic resonance imaging
MRSA	Methicillin-resistant Staphylococcus aureus
MSE	Mental State Examination
MUAC	Mid upper arm circumference
Na	Sodium
NADEMC	National Adverse Drug Event Monitoring Centre
NEC	Necrotising enterocolitis
NG	Nasograstric tube
NICD	National Institute for Communicable Diseases
NIMART	Nurse initiation and management of antiretroviral therapy
NMS	Neuroleptic Malignant Syndrome
NNRTI	Non-nucleoside reverse transcriptase inhibitors
NS	Nephrotic Syndrome
NSAID	Non-Steroidal Antiinflamatory Drug
NTS	Non-Thyphoid Salmonella
NVP	Nevirapine
oC	degrees Celsius
OCD	Obsessive Complusive Disorder
ORS	Oral Rehydration Solution
PANDAS	Paediatric Autoimmune Neuropsychiatric Disorders associated with streptococcal infection
PCP	Pnuemocystis Jiroveci Pneumonia
PCR	Polymerase Chain Reaction
PCV	Pneumococcal conjugate vaccine
PDA	Patent ductus arteriosus
PDDS	Pervasive Developental Disorders

PEFR	Peak expiratory flow rate
PGL	Persistent generalised lymphadenopathy
pH	acidity (partial pressure of hydrogen)
PHC	Primary Health Care
PI	Protease inhibitor
PML	Progressive multifocal leukoencephalopathy
PMTCT	Prevention of mother to child transmission
PN	Parenteral Nutrition
PPN	Partial Parenteral Nutrition
PTB	Pulmonary tuberculosis
PTC	Pharmaceutical and Therapeutics Committee
PTSD	Post Traumatic Stress Disorder
PTT	Partial Thromboplastin Time
PZA or Z	Pyrazinamide
R	Rifampicin
RBC	Red Blood Cell
RF	Rheumatoid factor
RH	Rifampicin/Isoniazid
rHuEPO	Recombinant human erythropoietin
RHZE	Rifampicin/Isoniazid/Pyrazinamide/Ethambutol
RIG	Rabies Immunoglobulin
ROP	Retinopathy of prematurity
RPR	Rapid plasma reagin
RSTI	Repeated supratherapeutic ingestion
RTI	Respiratory Tract Infection
RUTF	Ready to Use Therapeutic Foods
SAM	Severe Acute Malnutrition
SANCA	South African National Council on Alcoholism and Drug Dependance
SAVP	South African Vaccine Producers
SC	Subcutaneous
SE	Status Epilepticus

SIADH	Syndrome of inappropriate antidiuretic hormone secretion
SJS	Stevens-Johnson Syndrome
SLE	Systemic Lupus Erythematosis
SMEI	Severe Myoclonic Epilepsy of Infancy
SSRI	Selective serotonin reuptake inhibitor
SSS	Sugar Salt Solution
STI	Sexual transmitted infections
SUD	Substance Use Disorders
T4	Thyroxine
TB	Tuberculosis
TBM	Tuberculous Meningitis
TDF	Tenofovir
TDR	Total drug-resistant
TEN	Toxic Epidermal Necrolysis
TIG	Tetanus immunoglobulin
TPHA	Treponema pallidum haemoglutination test
TPN	Total Parenteral Nutrition
TSH	Thyroid-stimulating hormone
TST	Tuberculin Skin Test
TT	Tetanus toxoid
URTI	Upper Respiratory Tract Infections
UTI	Urinary Tract Infection
VEOS	Very Early-Onset Schizophrenia
VL	Viral load
VLDL	Very Low Density Lipoprotein
VSD	Ventricular Septal Defect
VZIG	Varicella-Zoster immunoglobulin
WCC	White Cell Count
WHO	World Health Organization
XDR	Extensive drug-resistance
